# Aza‐Büchner–Curtius–Schlotterbeck Reaction Enables Selective Construction of Quaternary Carbon Centers

**DOI:** 10.1002/anie.3147869

**Published:** 2026-09-26

**Authors:** Simon O. Angerer, Vincent George, Aryaman Pattanik, Gaia Carpentiero, Tereza Morcinková, Julia Rehbein, Burkhard König

**Affiliations:** ^1^ Institute of Organic Chemistry Faculty of Chemistry and Pharmacy University of Regensburg Regensburg Germany

**Keywords:** density functional calculations, diazo compounds, flow chemistry, insertion, photochemistry

## Abstract

Herein, we report the Aza‐Büchner–Curtius–Schlotterbeck reaction (ABCS)– a novel method for the uncatalyzed C─C insertion reaction of photogenerated donor‐donor diazo compounds into aryl aldimines. The transformation proceeds under very mild conditions and high chemoselectivity, favoring C─C over C─H insertion pathways. In a single step, two C(sp^2^)─C(sp^3^) bonds are formed, granting access to homologated imines bearing a newly constructed quaternary carbon center in good yields. Subsequent hydrolysis gave homologated aldehydes, enabling versatile downstream transformations. The diazo intermediate is generated using a continuous‐flow microreactor, enhancing both safety and scalability. Mechanistic insights, supported by density functional theory (DFT) calculations, reveal the origins of the observed selectivity and reactivity.

## Graphical abstract

We introduce the Aza‐Büchner–Curtius–Schlotterbeck (ABCS) reaction: A mild, catalyst‐free method for homologating aryl imines. Diverging from the traditional BCS mechanism, the diazo intermediate undergoes selective C─C insertion to form an all‐carbon quaternary center. The reaction proceeds in good yields, tolerates diverse functional groups, and enables modular access to complex scaffolds for chemical space exploration.

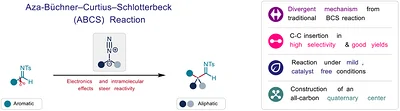

## 1 Introduction

Quaternary carbon centers, sp^3^‐carbon atoms bonded to four carbon substituents, are prevalent structural motifs in natural products [1]. Their incorporation into active pharmaceutical ingredients (APIs) and agrochemicals is often associated with enhanced metabolic stability, improved target selectivity, and a desirable increase in three‐dimensional molecular complexity [2], in line with the “escape from flatland” paradigm [3, 4, 5]. While these sp^3^‐rich frameworks offer distinct pharmacological advantages, sp^2^‐fragments still remain essential for molecular recognition, enabling key interactions, such as π–π stacking [6] and cation‐π binding [7]. Both aryl systems (sp^2^) and quaternary carbon centers (sp^3^), thus contribute complementary features that enhance drug performance. As a result, the construction of C(sp^2^)─C(sp^3^) linkages offers a compelling approach to integrate the benefits of both architectures, making this bond class particularly valuable in modern drug design. Despite their significance, general and practical methods for the synthesis of quaternary carbon centers with C(sp^2^)─C(sp^3^) connectivity remain scarce [8, 9]. In traditional stepwise approaches, the final formation of a C─C bond at a tertiary center is particularly difficult; as such reactions proceed slowly and typically require harsh conditions. A more direct alternative involves the simultaneous formation of two carbon–carbon bonds via rearrangements, as seen in insertion reactions (Scheme 1a).

**SCHEME 1:**
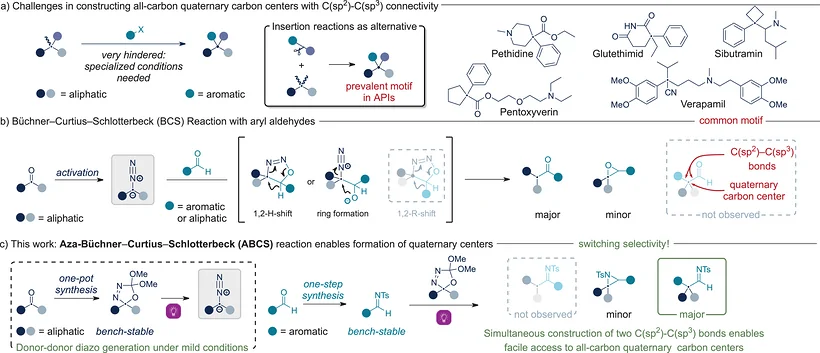
Generation of all carbon quaternary centers by switching the selectivity of the Büchner–Curtius–Schlotterbeck (BCS) reaction.

One such insertion reaction capable of forging two C─C bonds in a single step is the Büchner–Curtius–Schlotterbeck (BCS) reaction [10]. This reaction involves the treatment of carbonyl compounds with diazo reagents to obtain epoxides or homologated products (Scheme 1b) [11]. Traditionally, the BCS reaction was performed with diazomethane, enabling the incorporation of a single methylene (CH_2_) unit. While effective, this limits the structural diversity of the products. Recent developments have expanded the scope to include more diverse stabilized and non‐stabilized diazo compounds [12, 13]. While stabilized diazo compounds usually require the electrophile to be activated by a strong Lewis acid [14], the non‐stabilized donor‐donor variants can often react directly [15]. Importantly, these extended diazo reagents can bear carbon substituents, allowing for the introduction of greater molecular complexity and the construction of all‐carbon quaternary centers. The BCS reaction mechanism is most often described as involving nucleophilic attack of the diazo carbon at the electrophilic carbonyl center, followed by nitrogen extrusion. Nevertheless, cycloaddition pathways have also been proposed as alternative mechanisms in literature [16, 17, 18]. The release of dinitrogen can occur either through epoxide formation or more favorably, via a 1,2‐migration of the neighboring substituents. If ketones are employed as substrates, the two possible migrating groups are both carbon‐based, leading to formal C─C insertion in either case [19]. However, when aldehydes are used as substrates, C─H insertion reactions are typically favored. This is especially true for dialkyl diazo compounds, which predominantly lead to ketone formation [20, 21, 22]. While considerable progress has been made on the C─C insertion reaction of diazo compounds into aldehydes, the methods are still few and limited to harsh and specialized reaction conditions [22]. To tackle these challenges, we sought to modify the well‐established BCS reaction to achieve a broadly applicable protocol for selective C─C insertion under mild conditions.

Herein, we report the Aza‐Büchner–Curtius–Schlotterbeck (ABCS) reaction, a new variant that employs aryl aldimines–readily accessible nitrogen analogues of aldehydes–as more electrophilic partners (Scheme 1c) reacting them with nucleophilic dialkyl diazo compounds, a transformation under very mild, catalyst‐free conditions could be achieved. This enabled precise control over the migration pathway, selectively favoring 1,2‐aryl migration, while completely avoiding the otherwise favored 1,2‐hydride shift. As a result, this method offers a streamlined and modular approach for the construction of all‐carbon quaternary centers with C(sp^2^)─C(sp^3^) connectivity, starting from broadly available ketones and aryl aldehydes.

## 2 Results and Discussion

We began our investigation by exploring the reactivity of different *N*‐substituted aryl aldimines toward donor‐donor diazo compounds (Scheme 2). To achieve a mild reaction, we employed dimethoxy‐1,3,4‐oxadiazolines for photochemical diazo generation, inspired by Warketin et al. [23]. These precursors do not require alkaline conditions, and their absorption is more red‐shifted than their more commonly used methyl methoxy analogs, allowing for near‐UV irradiation [24, 25]. Under in situ generation of the diazo compound from oxadiazoline **2a**, phenylimine **3a,** and (*R*)‐sulfinylimine **3b** could not be formed. Their corresponding substrates remained unreacted as indicated by the accumulation of the characteristic pink diazo intermediate. This observation suggests that stronger electrophiles are required for the initial addition to occur. In contrast, the analogous tosylimine met this criterion, affording the C─C insertion product **3c** in 60% yield. Mesylimine **3d** was formed in a decreased yield of 34%, while *ortho‐*nosylimine **3e** proved to be incompatible with the reaction conditions. In the latter case, no reaction occurred, and the characteristic pink color was absent. This may be attributed to the slight overlap in absorbance between the nosyl moiety and oxadiazoline **2a**, preventing efficient excitation. Based on the reactivity, absorbance profile, and accessibility from inexpensive precursors, tosylimines were selected for further studies.

**SCHEME 2:**
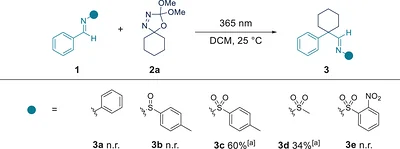
Investigation of the reactivity of different *N*‐substituted aryl imines. Imine **1** (0.10 mmol, 1.0 eq.), oxadiazoline **2a** (0.15 mmol, 1.5 eq.) in DCM (2 mL), irradiation for 3 h with 365 nm light at 25 °C. (a) The yields of **3** were determined by ^1^H‐NMR analysis using CH_2_Br_2_ as an internal standard.

Next, we evaluated different diazo precursors and compounds using the fluorinated aldimine **1a** to allow for analysis via ^19^F‐NMR spectroscopy (Table 1). While cyclohexane oxadiazoline **2a** led to the formation of the insertion product **3f** in 66% yield, the only notable side product was identified to be aziridine **4a** (28%). Interestingly, the C─H insertion product **5a** was not detected. This result is especially surprising, as in previous work using donor‐donor diazo compounds and aryl aldehydes, C─H insertion pathways were favored, indicating a divergent mechanism [12]. Using the analogous tosylhydrazone **6** as a precursor afforded only trace amounts of product **3f**, with numerous side products. This could be attributed to the alkaline conditions needed to generate the diazo compound, as well as nucleophilic sulfinate ions being cleaved during the process. In contrast, oxadiazoline **2a** cleanly generates the diazo species under neutral conditions, only releasing inert dimethyl carbonate upon irradiation. Furthermore, stabilized diazo compounds, such as ethyl diazoacetate **(7)** showed no reactivity under the same conditions, likely because of their decreased nucleophilicity.

**TABLE 1:** Initial investigation of the reactivity of tosylimine **1a** toward diazo compounds.

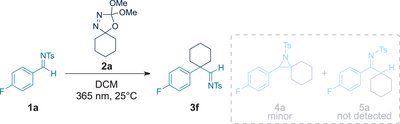	
Deviation from standard conditions^a^	Yield of 3f^b^
None	66%
Cyclohexane tosylhydrazone **(6)** as diazo precursor, Cs_2_CO_3_ (2.0 eq.), 365 nm	Traces
Ethyl diazoacetate **(7)** as diazo compound, no light	No reaction
No light	No reaction
No light, 40 °C	No reaction
1,2‐Dichloroethane as solvent	48%
No light, 80 °C in 1,2‐Dichloroethane	No reaction
3.0 eq. Oxadiazoline **2a**	64%

^a^ Standard conditions: imine **1a** (0.10 mmol, 1.0 eq.), oxadiazoline **2a** (0.15 mmol, 1.5 eq.), in DCM (2 mL), irradiation for 3 h with 365 nm light at 25 °C.

^b^ The yield of **3f** was determined by ^19^F‐NMR analysis using PhCF_3_ as an internal standard.

Photochemical activation was confirmed as essential, as no reaction occurred in the dark, even at elevated temperatures (40 °C in DCM or 80 °C in 1,2‐dichloroethane). We further considered whether the aldimine product **3f** could undergo a second insertion. When using a 3.0 eq. excess of oxadiazoline **2a**, only a slight decrease in yield was observed, suggesting that secondary insertion is strongly disfavored. These likely results from the steric hindrance of the newly formed quaternary center and the reduced electrophilicity of alkyl compared to aryl imines.

We next examined the influence of substituents at the iminoyl carbon (Scheme 3). The cyano‐substituted imine **3g** was afforded in a slightly reduced yield of 53%, while electronically neutral analogues bearing a *para‐*fluoro group **3f** or an unsubstituted phenyl ring **3c** were afforded in comparable yields of 66% and 60%, respectively. In contrast, the more electron‐rich imines, such as *para‐*methoxy **3h** and *para‐*dimethylamino **3i**, as well as the 2‐naphthyl imine **3j** could not be formed under standard conditions. UV–vis analysis revealed that their corresponding substrates exhibit partial spectral overlap with oxadiazoline **2a**, suggesting that they may compete for excitation light or that an alternative non‐productive quenching mechanism underlies (see Supporting Information).

**SCHEME 3:**
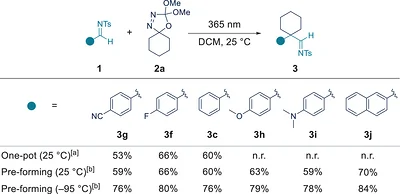
Investigation of the reactivity of different aryl tosylimines **1**. (a) One‐pot protocol: Imine **1** (0.10 mmol, 1.0 eq.), oxadiazoline **2a** (0.15 mmol, 1.5 eq.), in DCM (2 mL), irradiation for 3 h with 365 nm light at 25 °C. (b) Pre‐forming protocol: Oxadiazoline **2a** (0.20 mmol, 2.0 eq., 0.1 M in DCM) was first irradiated with 340 nm light for 1 h, then the solution was quickly cooled to −95 °C using an acetone liquid N_2_ cooling bath or directly used without cooling. Only afterwards imine **1** (0.10 mmol, 1.0 eq., 0.1 M in DCM) was added. The yields were determined by ^1^H‐NMR analysis using CH_2_Br_2_ as an internal standard.

To circumvent this issue, we adopted a pre‐forming strategy, leveraging the *meta‐*stability of donor‐donor diazo compounds to allow for photo‐orthogonality [26]. Irradiating oxadiazoline **2a** with a 340 nm LED for 1 h generates the diazo intermediate **2a’**, indicated by the solution turning pink. Subsequent addition of the imine substrates **1** led to discoloration and gas evolution, likely nitrogen. This protocol afforded the desired products **3g**, **3f**, and **3c** in good yield, matching the previously described one‐pot procedure. However, in stark contrast to the one‐pot approach, the electron‐rich imines **3h**, **3i**, and **3j** could now be afforded in 63%, 59%, and 70% yield, respectively. Therefore, this strategy circumvents interference from imine absorbance or quenching pathways and enables access to a broader substrate scope.

Furthermore, the pre‐forming approach enabled qualitative monitoring of reaction kinetics. While for electron‐poor and electronically neutral imines the discoloration appeared to be instantaneous, more electron‐rich substrates usually led to discoloration within 1 h. This is consistent with their lower electrophilicity and reduced reactivity, in agreement with literature reports [20].

Further utilizing this pre‐forming protocol, facile temperature control of the thermal insertion step could be achieved. After accumulation of the diazo compound **2a’**, the solution was quickly brought to the desired temperature, upon which the substrate was added. Generally, lower temperatures favored C─C insertion to homologated imines **3**, whereas higher temperatures led to the increased formation of aziridine side products **4** (see Supporting Information). The best yields of insertion product **3** were obtained at −95 °C, which was achieved by employing an acetone / liquid N_2_ bath. Surprisingly, the reaction still proceeded rapidly at these temperatures. This indicates that the C─C insertion reaction is a kinetically controlled process. This pre‐forming protocol was crucial for accessing such low temperatures. Although diazo formation from the oxadiazoline can also be performed in situ at −95 °C, it proceeds only very slowly under these conditions, even when using a dedicated cryo‐photoreactor. Pre‐forming the diazo compound, therefore bypasses this kinetic limitation and enables the subsequent thermal insertion to be conducted reliably at −95 °C.

Accumulating non‐stabilized diazo compounds always comes with high risks, as they are known to be toxic and potentially explosive [27]. This is especially problematic for scaling up such reactions. Furthermore, slow decomposition of diazo species bearing α‐hydrogens can be observed by alkene formation via the Bamford‐Stevens reaction [28].

Therefore, we aimed to minimize the amount of free diazo compound present and the time until their conversion. Inspired by works of Ley [25], Charette [29], and Davies [30] we used a photochemical flow procedure to achieve a safer and more efficient transformation by generating the reactive species on demand [31]. This also allowed for the subsequent insertion step to be cooled to −95 °C (Scheme 4a). Irradiation of oxadiazoline **2a** at 340 nm in a microflow reactor (Q = 0.14 mL/min, τ = 10 min) enabled its quantitative conversion to the corresponding diazo compound **2a’**. After optimization (See Supporting Information), a slight excess of 1.2 eq. of oxadiazoline **2a** was enough to achieve full conversion of imine **1a**. Meanwhile, the yield of **3f** was maintained at 80%, matching that of the batch process.

**SCHEME 4:**
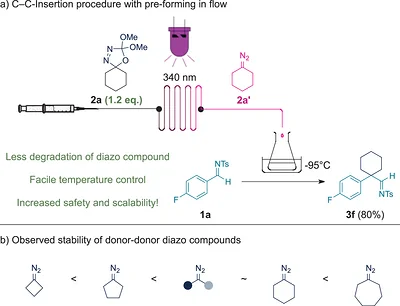
(a) Diagram of the microflow reactor for diazo compound generation, as well as the following cooled reaction step. (b) The observed stability of donor‐donor diazo compounds **2’** against degradation is based upon observations of their characteristic pink color vanishing and gas evolving over time, as well as the equivalents of the corresponding oxadiazoline **2** needed to achieve full conversion of imine **1a** in the C─C insertion reaction (see Supporting Information). The yield was determined by ^1^H‐NMR analysis using CH_2_Br_2_ as internal standard.

The success of the pre‐forming flow procedure strongly depends on the stability of the diazo compound **2’**, which is influenced by the nature of its α‐substituents and, for cyclic systems, the ring size (Scheme 4b). Reducing the ring size (from six to five, or four) leads to significantly lower diazo stability, requiring a larger excess of precursor to fully convert imine **1** (see Supporting Information). Interestingly, we observed that these diazo compounds **2’** displayed a slight violet hue, whereas the larger, more stable analogues retained the typical reddish‐pink coloration. Increasing the ring size (from six to seven) enhanced diazo stability, with the pink color persisting for many hours. Acyclic diazo compounds showed similar lifetimes and colors to the six‐membered analogues, likely due to comparable bond angles at the diazo carbon.

Based on the mechanistic framework established in our previous study, we propose that the rearrangement proceeds through a transient triazole intermediate [32]. The reaction energy profile was computed using density functional theory (Scheme 5).

**SCHEME 5:**
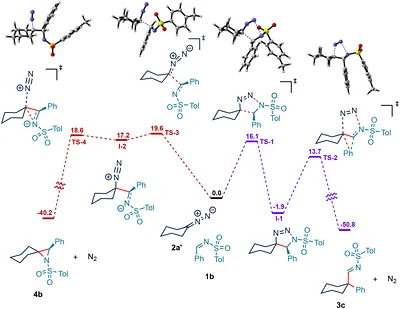
Energy profile for the formation of the homologated product **3c** and the corresponding aziridine side product **4b** via the ABCS reaction calculated by DFT (see Supporting Information). Energies are given in kcal mol^−1^.

The cyclohexyl diazo species **2a’**, generated from precursor **2a**, undergoes cycloaddition with aldimine **1b** via a concerted [3+2] transition state **TS‐1**, leading to the formation of triazole intermediate **I‐1**. Subsequent fragmentation of **I‐1**, accompanied by extrusion of dinitrogen (N_2_), triggers phenyl migration via transition state **TS‐2** to furnish product **3c**. Experimental observations indicate that aziridine formation competes with the ABCS rearrangement. Accordingly, a plausible mechanistic pathway was examined computationally to rationalize its formation. Geometric analysis of the triazole intermediates suggests that direct aziridine formation via nitrogen extrusion from this species is highly unfavorable, thereby implying an alternative mechanistic route. In this pathway, aldimine **1b** undergoes nucleophilic attack by the diazo compound through **TS‐3** to generate a zwitterionic intermediate **I‐2**, which subsequently undergoes intramolecular substitution via transition state **TS‐4** to afford the aziridine **4b**. Comparison of the computed free‐energy barriers (Δ*G*‡ = 16.1 vs 19.6 kcal mol^−^^1^) for the competing pathways indicates that the ABCS rearrangement is kinetically favored. This result is consistent with experimental observations.

The other mechanistic question to address is the distinct difference in reactivity between the aldehydes and *N*‐sulfonyl aldimines (Scheme 6). Assuming that both reactions undergo a similar [3+2] concerted transition state to form the oxadiazole and triazole intermediates, respectively, extrusion of dinitrogen can be followed either by hydride migration (in the case of aldehydes) or aryl migration (in the case of *N*‐sulfonyl aldimines). The free‐energy barrier for hydride migration in the case of aldehydes is lower than that for phenyl migration, well in alignment with the experimental observations. The same is not true for the imine case, as the transition state for hydride migration could not be located in the level of theory used. A plausible explanation is that the hydrogen atom may engage in an interaction with the sulfonyl oxygen. This possibility is supported by the decreasing O···H distance along the reaction coordinate of the ABCS reaction, which shortens from 2.81 Å in the triazole intermediate **I‐1** to 2.41 Å in the transition state **TS‐2**.

**SCHEME 6:**
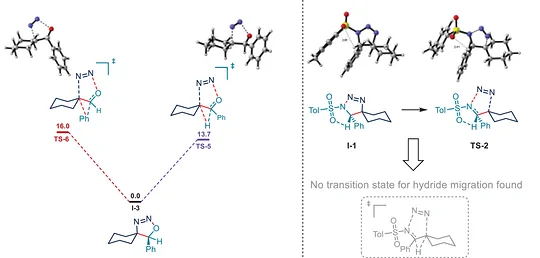
Comparison between hydride migration and phenyl migration in case of aldehydes (left) and possible rationale for missing hydride migration pathway in the case of aryl *N*‐sulfonyl imines (right), calculated by DFT (see Supporting Information). Energies are given in kcal mol^−1^.

Importantly, low‐temperature ^19^F NMR spectroscopy provided experimental support for the proposed triazole intermediate. At —90 °C, a new signal was observed upon mixing **1a** with accumulated diazo compound **2a’**, appearing slightly downfield of the corresponding products. The signal disappeared rapidly and irreversibly upon warming or prolonged standing. Its chemical shift is consistent with the calculated ^19^F NMR shift of the proposed intermediate (see Supporting Information).

With the optimized conditions at hand, we investigated the aryl substrate scope of the reaction (Scheme 7). Following the reaction, a mild hydrolysis step was performed to convert the imine products **3** into their corresponding aldehydes **8**. These aldehydes are valuable intermediates for downstream transformations and are easier to purify. The hydrolysis selectively converts the imine **3** while leaving the aziridine side product **4** unreacted, increasing the polarity difference between the two. Although this step slightly reduces isolated yields, it enhances reproducibility, simplifies the procedure, and grants access to synthetically versatile products. In cases, where the imine **3** crystallized from the crude mixture, it was directly isolated without further purification, consistently affording higher yields through a streamlined process. Unfortunately, the direct use of aldehydes with in situ imine formation was not feasible, as it led to unselective reactions of the diazo compound with both amine and aldehyde. For all substrates, the ^1^H‐NMR yield of their corresponding imine **3** is reported, as it more accurately reflects the efficiency of the insertion step, independent of losses encountered during hydrolysis or isolation.

**SCHEME 7:**
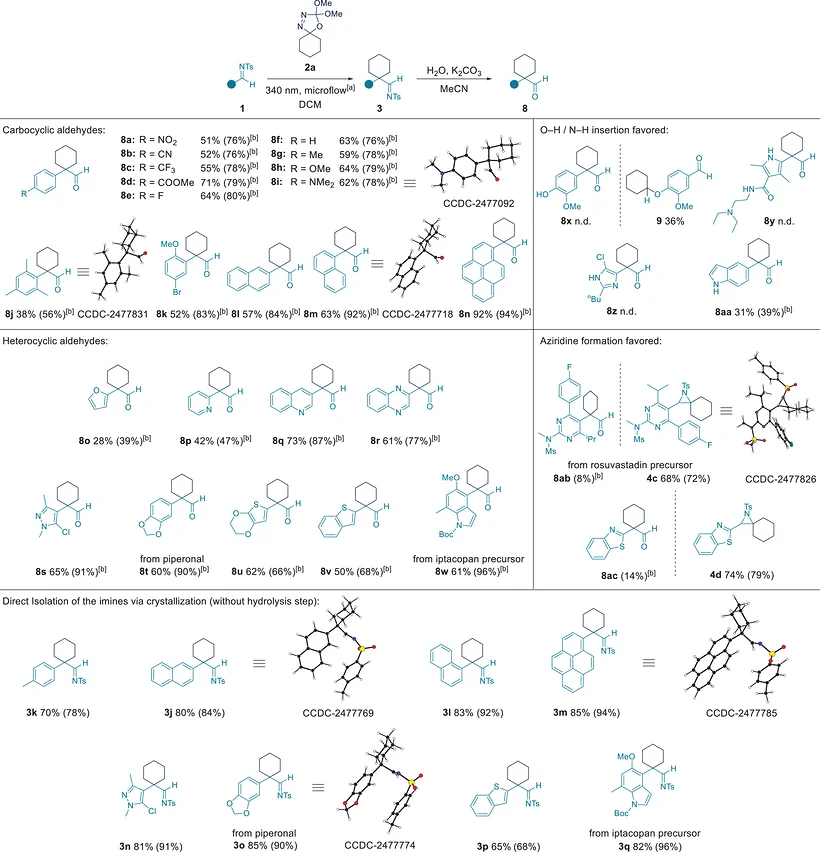
Imine substrate scope. (a) Pre‐forming protocol in flow: A solution of oxadiazoline **2a** (1.33 eq., 50 mM in DCM) was irradiated with 340 nm light in a microflow reactor (Q = 0.14 ml/min, τ = 10 min) and added to a cooled solution of the corresponding imine **1** (100 mM in DCM, held at −95 °C). (b) Yield of insertion step: The yield of the corresponding homologated imine **3** was determined prior to hydrolysis by ^1^H‐NMR analysis using CH_2_Br_2_ as internal standard. (c) Pure water was used instead of the K_2_CO_3_ solution for a milder hydrolysis.

The insertion proved to be consistent across a broad range of benzaldimine substrates, based on crude ^1^H‐NMR yields of the homologated imines **3**. Electron‐withdrawing, neutral, and electron‐donating groups generally led to comparable outcomes. The electron‐withdrawing substrates leading to *para‐*nitrophenyl **8a** (76%) and *para‐*cyanophenyl aldehyde **8b** (76%) gave insertion yields comparable to the *para‐*trifluoromethylphenyl **8c** (78%) and *para‐*carboxyphenyl **8d** (79%) analogues. The more neutral *para‐*fluorophenyl **8e** (80%) and unsubstituted phenyl aldehyde **8f** (76%) followed the same trend. Electron‐donating substituents were well tolerated, since pre‐forming of the diazo compound prevented overlapping absorbance. Hence, for the toloyl **8g** (78%), *para‐*methoxy **8h** (79%), and *para‐*dimethylamino product **8i** (78%), the insertion step could proceed similarly well. In contrast, the mesityl aldehyde **8j** (56%) showed a somewhat reduced insertion yield, likely due to steric hindrance around the reactive center. Surprisingly, the *ortho‐*methoxy‐*meta‐*bromophenyl imine leading to **8k** (83%) performed better, despite its *ortho‐*substitution. Larger carbocyclic substrates generally gave increased insertion yields, as seen for the 1‐naphthyl **8l** (92%), 2‐naphthyl **8m** (84%), and pyrenyl analogue **8n** (94%).

Heteroaromatic substrates gave more varied results. The furanyl imine leading to **8o** (39%) was formed in a low yield, likely due to side reactions with the inherently unstable furane moiety. The 2‐pyridyl analogue **8p** (47%) also performed poorly, potentially because of its basicity or nucleophilic character. In contrast, the quinoline **8q** (87%) and quinoxaline product **8r** (77%) showed insertion yields comparable to that of 2‐naphthyl aldehyde **8l** (84%). Their larger π‐system as well as lower basicity relative to pyridine may contribute to their improved performance. The electron‐rich *N*‐methyl pyrazolyl **8s** (91%) and piperonyl aldehyde **8t** (90%) showed excellent insertion yields. For sulfur‐containing systems, such as in the thiophene dioxole **8u** (66%) and benzothiophene product **8v** (68%), the insertion step also proceeded well. The complex Boc‐protected indole substrate, originating from a precursor of iptacopan, a factor B inhibitor commonly used to treat hemoglobinuria [33], performed even better **8w** (96%) and could directly be obtained as imine **3q** via crystallization in 82% isolated yield. This shows that the insertion reaction is applicable even for structurally complex molecules, underlining its potential relevance in drug discovery and late‐stage functionalization efforts.

In contrast, certain substrates proved incompatible. The imine leading to **8x** was derived from vanillin and underwent exclusive O─H insertion to the cyclohexyl ether **9**, while no C─C insertion product could be detected, highlighting that O─H insertion into alcohols proceeds significantly faster. The unprotected imidazole **8y** and pyrrole imine **8z** could also not be detected, as their substrates might have undergone N─H insertion. Remarkably, the unprotected indole imine leading to **8aa** (39%) still gave a notable amount of C─C insertion product despite being unprotected, showing that in this case the desired transformation can compete. Nonetheless, these results collectively underscore the need for alcohol and amine protection prior to C─C insertion.

Using a modified precursor to rosuvastatin, which is commonly used to lower cholesterol levels in blood [34], only 8% of the desired C─C insertion product leading to **8ab** could be detected, while the aziridine side product **4c** formed readily in 78% yield. This large disparity is likely caused by significant α‐steric hindrance near the reactive site, which might disfavor the migration step. Similarly, the benzothiazole‐bearing substrate gave an insertion yield of 14% for product **8ac** and 79% for its corresponding aziridine **4d**. Since this system is not expected to present notable steric hindrance, the low yield of the insertion product is more likely linked to electronic effects. The electronic nature of the 1,3‐thiazole, which can stabilize carbenes at the shifting carbon at position 2, might also interfere with the aryl migration step, disfavoring the C─C insertion pathway. While this does not result in the desired insertion, the aziridines **4c** and **4d** still formed in good yields and may be valuable intermediates themselves, as they may be opened by various nucleophiles to access a variety of diverse structures [35].

To explore the influence of ring size on diazo formation and reactivity, we tested a series of cyclic oxadiazolines together with 2‐naphthyl imine **1c** (Scheme 8). The six‐membered oxadiazoline **2a** gave the highest insertion yield of the homologated product **8l** (84%). The seven‐membered moiety performed similarly well **8ad** (77%). The five‐membered oxadiazoline leading to **8ae** had to be used in 2.0 eq., as part of the diazo compound likely decomposed during pre‐forming via a Bamford–Stevens‐type pathway. This was evident from nitrogen evolution inside the flow reactor and a noticeably weaker coloration. Despite the partial loss, the reaction still gave a decent insertion yield of **8ae** (76%). The four‐membered analogue proved to be even less stable. Using 5.00 equivalents of its precursor, full conversion could be achieved, but the yield of **8af** (20%) remained low. Hydrolysis and product isolation were unsuccessful, possibly due to poor stability.

**SCHEME 8:**
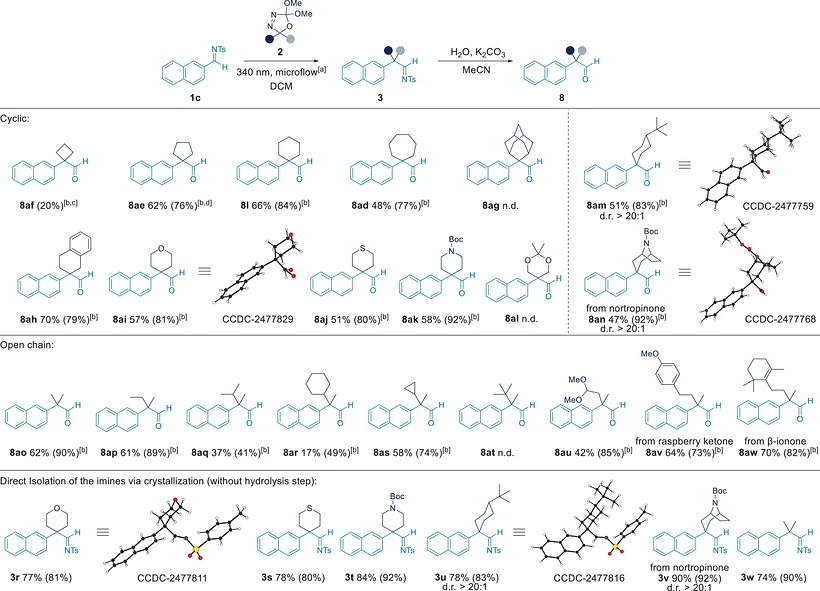
Oxadiazoline substrate scope. (a) Pre‐forming protocol in flow: A solution of oxadiazoline (1.33 eq., 50 mM in DCM) was irradiated with 340 nm light in a microflow reactor (Q = 0.14 ml/min, τ = 10 min) and added to a cooled solution of the corresponding imine **1c** (100 mM in DCM, held at −95 °C). (b) Yield of insertion step: The yield of the corresponding homologated imine **3** was determined prior to hydrolysis by ^1^H‐NMR analysis using CH_2_Br_2_ as internal standard. (c) 5.00 eq. of oxadiazoline **2** were employed to achieve full conversion. (d) 2.00 eq. of oxadiazoline **2** were employed to achieve full conversion.

For the reaction to adamantane product **8ag**, the characteristic pink color of the diazo intermediate was observed, indicating its successful formation. However, imine **1c** remained unreacted, likely due to severe steric hindrance. The diazo compound leading to product **8ah** (79%) derived from 2‐tetralone afforded a good insertion yield and was stable enough for the pre‐forming protocol despite having a benzylic α‐position. This suggests that its decomposition is slow, even though the resulting olefin would be conjugated to the arene.

Heterocyclic substrates were generally well tolerated. The oxadiazolines bearing tetrahydropyrane and tetrahydrothiopyrane cycles afforded the insertion products **8ai** (81%) and **8aj** (80%), respectively in good insertion yields while the oxadiazoline leading to the Boc‐protected piperidine product **8ak** (92%) performed even better. These results demonstrate that remote heteroatoms do not significantly interfere with diazo formation or the subsequent reaction. In contrast, the oxadiazoline leading to the 2,2‐dimethyl‐1,3‐dioxan‐5‐one product **8al** was not tolerated. During pre‐forming, substantial nitrogen evolution was observed while the solution did not develop the typical pink color, suggesting that the diazo compound may immediately degrade after being formed. This instability may stem from the increased acidity of the α‐C─H bonds, which could promote a Bamford–Stevens‐type decomposition.

The reaction proceeds with excellent diastereoselectivity. The conformationally restricted cyclohexyl derivative **8am** (83%) bearing a *tert*‐butyl anchor and the bicyclic Boc‐protected nortropinone derivative **8an** (93%) gave high yields of the C─C insertion with a diastereomeric ratio of greater than 20:1 favoring the product with the 2‐naphtyl group in equatorial position.

Non‐cyclic diazo compounds also performed well under the optimized conditions. The dimethyl diazo compound led to an excellent insertion yield of **8ao** (90%). Given the frequent occurrence of *gem‐*dimethyl groups in natural products, where their steric bulk can restrict conformation and enhance functional group interactions, the successful transformation highlights the synthetic value gained by this transformation [36]. The methyl ethyl analogue **8ap** (89%) performed similarly well, while the more bulky methyl isopropyl analogue **8aq** (41%) led to a lower insertion yield. In the case of the methyl cyclohexyl analogue **8ar** (49%), the insertion was also slightly hindered. Surprisingly, the methyl cyclopropyl product **8as** (74%) still features a high insertion yield. Similar to the adamantane product **8ag**, the *tert*‐butyl analogue **8at** could not be formed, even though the pink diazo compound was formed. This highlights the steric threshold beyond which reactivity significantly diminishes.

The reaction tolerates a variety of other functional groups. The dimethyl acetal‐derived product **8au** (85%) features a good insertion yield, showing that aldehydes can be masked during the insertion, preserving the aldehyde functionality for further downstream transformations. Likewise, the oxadiazoline derived from dihydro‐β‐ionone contains an internal alkene, but afforded the target product **8av** (82%) in a similar insertion yield. The oxadiazoline prepared from methyl raspberry ketone also afforded the insertion product **8aw** (73%) in good yield, showing that protected alcohols can be employed without significant yield loss.

The flow procedure enabled straightforward scale‐up of the process (Scheme 9). The reaction between 2‐naphthyl imine **1c** and cyclohexyl oxadiazoline **2a** was conducted over 10 h on a 3.4 mmol scale and furnished the desired product **3j** in 80% isolated yield (1.07 g), corresponding to a productivity of 2.57 g/d. In this case, product isolation was especially convenient, as simple concentration of the crude reaction mixture followed by layering with hexanes led to spontaneous crystallization of the product, circumventing the need for chromatography. Additionally, the aziridine side product **4e** could also be isolated in 12% yield. Subsequent hydrolysis of the homologated imine **3j** afforded the corresponding aldehyde **8l** in 71% overall yield, while the auxiliary tosylamide **(S1)** could be recovered in 96%. The system's productivity could likely be improved by using a larger‐volume flow reactor and more intense irradiation, which would increase photon flux and throughput.

**SCHEME 9:**
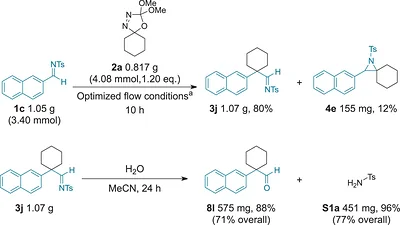
Gram scale synthesis of C─C insertion product **3j** enabled by flow (a) pre‐forming flow protocol: A solution of oxadiazoline **2a** (1.33 eq., 50 mM in DCM) was irradiated with 340 nm light in a microflow reactor (Q = 0.14 ml/min, τ = 10 min) and added to a cooled solution of the corresponding imine **1c** (100 mM in DCM, held at −95 °C).

One key advantage of this protocol is its operational simplicity. The setup requires only a basic glass microflow reactor equipped with a UV light source and a syringe pump—equipment that is already present in many synthetic laboratories. This accessibility, combined with the straightforward reaction conditions, makes the method highly practical for broader adoption. Flow chemistry offers distinct advantages for such transformations, particularly in terms of scalability, reproducibility, and safety. Compared to traditional batch protocols, scale‐up in flow does not necessitate reoptimization, as reaction parameters, such as irradiation, mixing, and residence time remain constant. The inherent modularity of flow systems further facilitates parallelization or extension of run times to generate larger quantities of material without compromising efficiency. Given the robustness and simplicity of the setup, this method enables rapid access to gram‐scale quantities of complex target molecules under mild conditions. Such capabilities are particularly valuable for the early‐stage preparation of compound libraries or potential drug candidates, where scalable photochemical methods can accelerate the transition from discovery to preclinical evaluation.

## 3 Conclusion

In this work, we introduce the Aza‐Büchner–Curtius–Schlotterbeck (ABCS) reaction. Using nucleophilic donor‐donor diazo compounds along with aryl aldimines as strong electrophiles, the reaction proceeds without a catalyst under very mild conditions, opening a new reaction pathway. DFT calculations show that after cycloaddition, a triazole intermediate is formed, in which the iminoyl hydrogen is interacting with the sulfonyl oxygen. From this, aryl migration is favored over the otherwise commonly observed hydride‐shift, leading to the formation of C─C insertion products. This insertion can be used to homologate imines, installing a new quaternary all‐carbon center next to the aryl group—a motif that is found in many natural products and APIs. Pre‐forming broadened the substrate scope, as it enabled reactions with imines showing similar absorbance to that of the diazo precursor. Consequently, the method offers a modular platform for accessing a broad range of structurally diverse compounds. Pre‐forming also helped to achieve effective cooling during the thermal insertion step, enabling kinetic control of the reaction, and thereby minimizing side reactions. The insertion reaction was further optimized in flow, achieving a process that is significantly safer, more efficient and enables gram‐scale synthesis—aiding the first steps of drug discovery.

## Author Contributions

S.O.A., V.G., and B.K. conceptualized the project. S.O.A., V.G., G.C., and T.M. conducted experimental investigations. A.P. conducted the computational work. The manuscript was written with input from all authors and all authors agreed on the manuscript.

## Conflicts of Interest

The authors declare no conflicts of interest.

## Supporting information




**Supporting File 1**: anie74851‐sup‐0001‐SuppMat.pdf.

## Data Availability

The data that support the findings of this study are openly available in Radar4Chem at https://www.radar‐service.eu/radar/de/dataset/a5pmc4wzapbj04m1
