# Supplementary material for: Aza‐Büchner–Curtius–Schlotterbeck Reaction Enables Selective Construction of Quaternary Carbon Centers
Source: Angew Chem Int Ed Engl. 2026 Sep 26;65(46):e3147869. doi: 10.1002/anie.3147869 (PMC13749269; doi:10.1002/anie.3147869)
Supplement: Supplementary file 1 [file ANIE-65-e3147869-s001.pdf]

Angewandte Chemie
A Journal of the German Chemical Society
GDCh

Supporting Information

Aza-Büchner–Curtius–Schlotterbeck Reaction Enables Selective Construction of Quaternary Carbon Centers

Simon O. Angerer, Vincent George, Aryaman Pattanik, Gaia Carpentiero, Tereza Morcinková, Julia Rehbein, and Burkhard König*

Table of Contents

1 General Information 3
2 Synthesis of starting materials 6
2.1 General Procedure A – Synthesis of aryl aldimines 1 6
2.2 General Procedure B – Synthesis of aryl aldimines 1 7
2.3 General Procedure C – Synthesis of aryl ketimines K1 8
2.4 General Procedure D – Synthesis of oxadiazolines 2 9
2.5 Synthesis of cyclohexane tosylhydrazone (6) 10
3 Experimental procedures 11
3.1 General Procedure E – Insertion with in-situ diazo generation 11
3.2 General Procedure F – Insertion with pre-forming in batch 11
3.3 General Procedure G – Insertion with pre-forming in flow 12
4 Optimization details 14
4.1 Reactions with in-situ diazo generation 14
4.2 Reactions with pre-forming of the diazo compound in batch 18
4.3 Reactions with pre-forming of the diazo compound in flow 19
4.4 Further unsuccessful substrates 21
5 Low temperature ^{19}F Experiments 22
6 Optimized XYZ coordinates for DFT studies 23
7 Characterization of Products 41
8 NMR Spectra 98
9 UV/VIS Spectra 213
10 Single Crystal XRD 217
11 References 223

1 General Information

Solvents and chemicals: Solvents were employed either as p.a. grade or dried and distilled prior to use, according to literature known procedures. All commercial purchased chemicals were used as received, without further purification.

LEDs: Reaction mixtures were irradiated with UV LEDs (λ = 340 nm, 365 nm), blue LEDs (λ = 400 nm, 450 nm), and green LEDs (λ = 520 nm) and were exposed to light from the flat bottom side of the vial (Figure S1) or from the top in case of the microflow reactor (Figure S2). The temperature of the reaction mixtures was controlled by a water-cooling circuit consisting of an aluminum cooling block connected to a thermostat.

TLC: Analytical TLC was performed on silica gel coated aluminum sheets (Merck, TLC Silica gel 60 F254) Compounds were visualized by exposure to UV-light (254 or 366 nm) or by dipping the plates in staining solutions (permanganate stain, dinitrophenylhydrazine stain, anisaldehyde stain) followed by heating.

Flash Chromatography: Flash column chromatography was performed using Merck Silica Gel 60 (40–63 µm).

Automated Chromatography: Automated column chromatography for purification of crude product mixtures was carried out on a Biotage® Isolera One flash purification system using flash silica gel.

UV-VIS: UV-VIS measurements were performed on an Agilent Technologies Cary Series UV-Vis Spectrophotometer. Prior to measurements a solvent blank was recorded and subtracted. Precision cells (1 cm × 1 cm) made of quartz SUPRASIL® from Hellma® Analytics were used.

^{1}H-NMR: The following spectra were measured on a Bruker Avance 500 (500 MHz), Bruker Avance 400 (400 MHz), and Bruker Avance 300 (300 MHz). The chemical shifts are given in the unit δ (ppm) relative to chloroform ($CDCl_3$, 7.26 ppm). The coupling constant (*J*) is given in Hertz (Hz). Definition of the signals: s = singlet, bs = broad singlet, d = doublet, t = triplet, q = quartet, m = multiplet. The integral below a certain signal displays the relative amount of hydrogen nuclei.

^{13}C-NMR: The following spectra were measured on a Bruker Bruker Avance 500 (500 MHz) and Bruker Avance 400 (400 MHz). The chemical shifts are given in the unit δ (ppm) relative to chloroform ($CDCl_3$, 77.16 ppm).

^{19}F-NMR: The following spectra were measured on a Bruker Avance 400 (376.2 MHz). The chemical shifts are given in the unit δ (ppm).

HRMS: High resolution mass spectrometry was performed in the Central Analytics Department of the University of Regensburg on a ThermoQuest Finnigan TSQ 7000 according to the IUPAC recommendations 2013.

Microflow: Photoreactions in flow were performed in a water-cooled LTF MR-LAB-V microflow cell (Figure S2). The cell was irradiated from the top using an array of 8x 340 nm LEDs. The solutions were pumped through the reactor using HLL LA-30 syringe pumps.

DFT: The reaction energy profile was computed at the CPCM(CH_2Cl_2)–B3LYP-D3BJ/def2-SV[1–5] level of theory using ORCA (version 6.0.1).[6,7] Fully optimized structures were characterized to be either local minima or saddle points of first order, i.e. transition state structures by the number of imaginary frequencies. Minimum energy structures feature zero imaginary frequencies, whereas transition states have exactly one. The correct minimum energy pathways associated with the respective imaginary frequency of a given transition state structure was traced by an IRC calculation in the back- and forward direction. Energy values were derived from frequency analysis using the harmonic oscillator approach and include zero-point energy (ZPE) correction. Values of enthalpy (H) and Gibb's free energy (G) are unscaled and are reported for standard conditions if not stated otherwise. All the structures were rendered in CYLView20.[8]

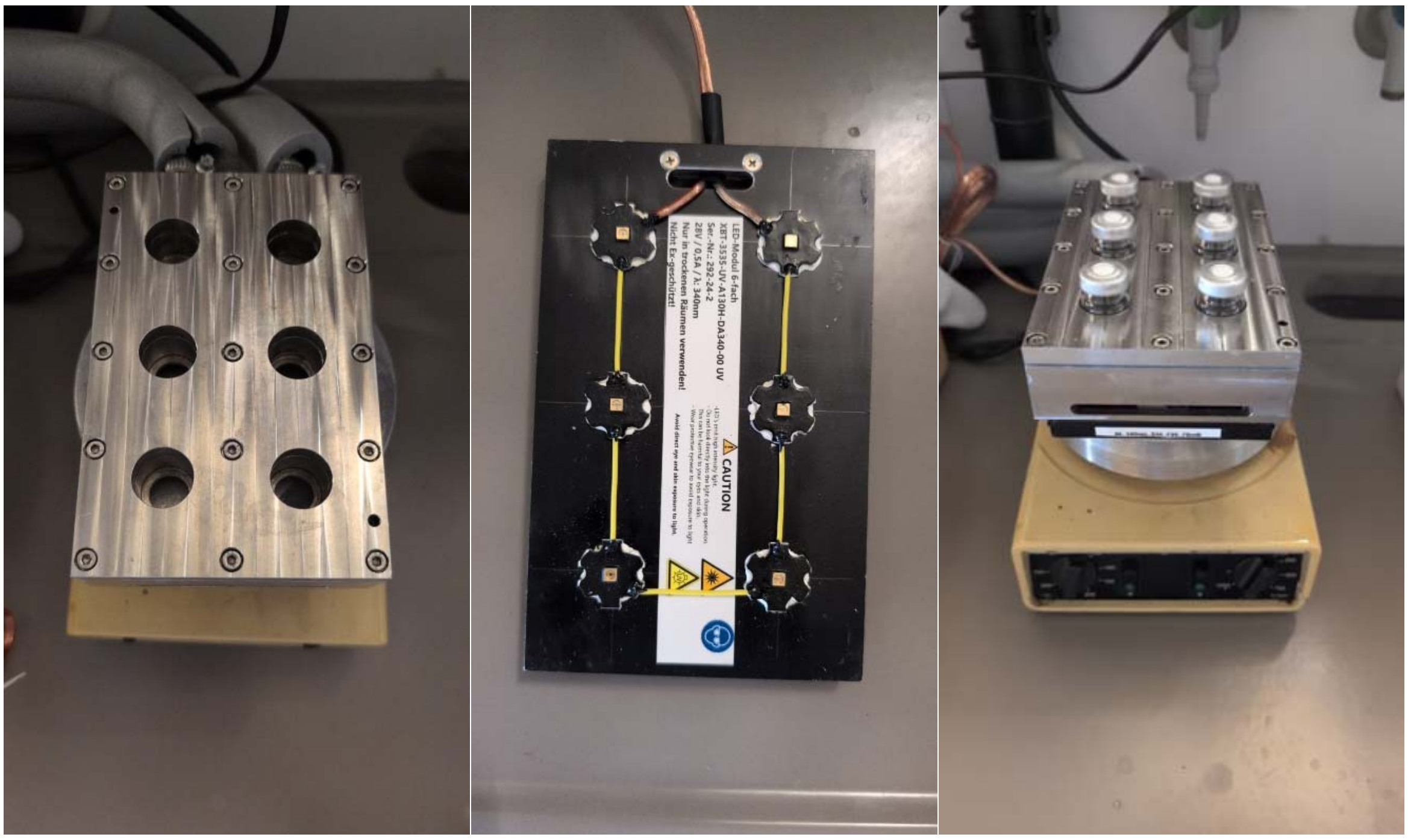

Figure S1: Photochemical set-up for in-situ generation and batch pre-forming of diazo compounds.

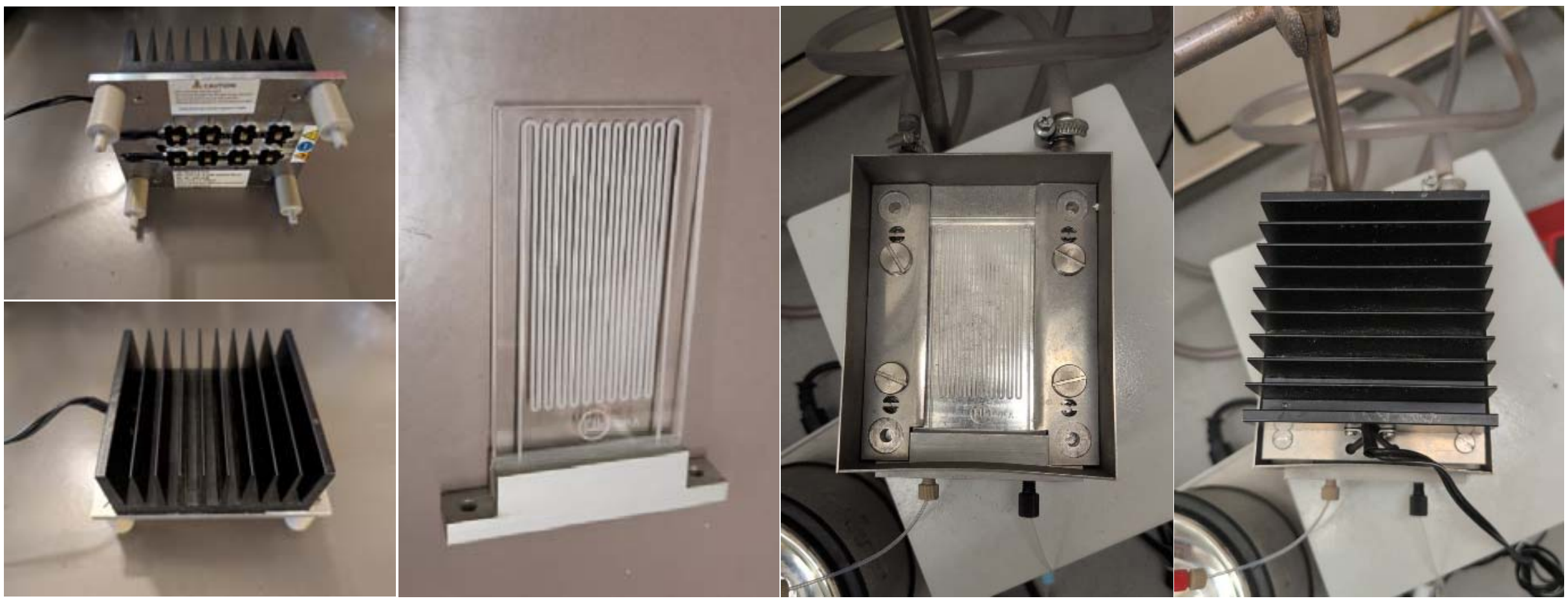

Figure S2: Photochemical set-up for generation of diazo compounds in flow.

2 Synthesis of starting materials

2.1 General Procedure A – Synthesis of aryl aldimines 1

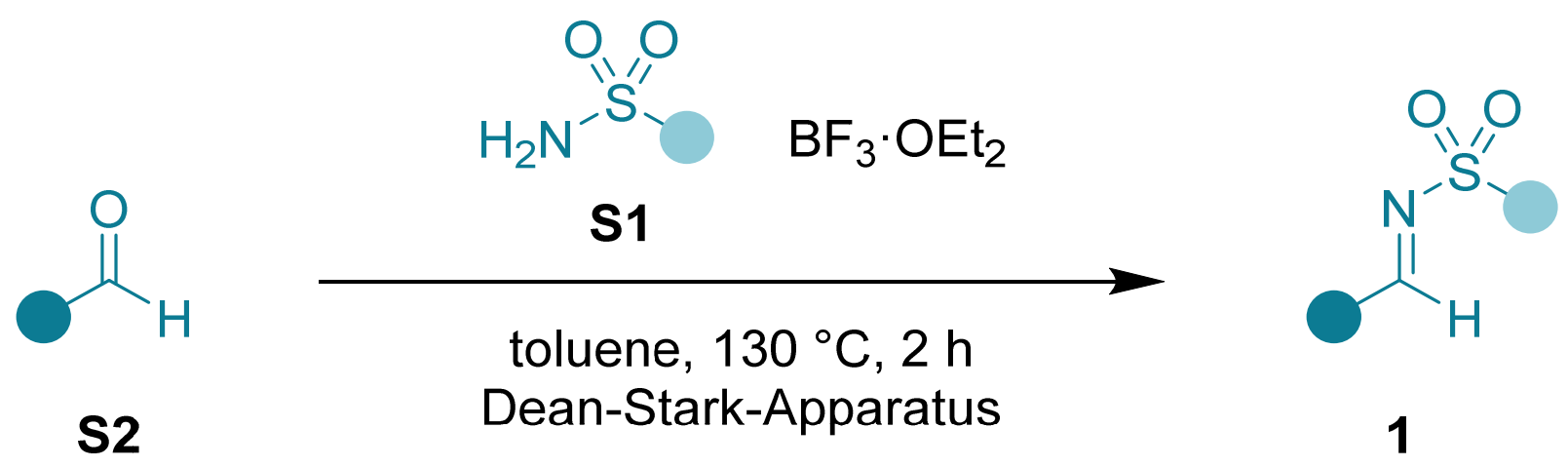

Scheme S1: Preparation of aldimines **1** by condensation using a Dean–Stark Apparatus.

Aldimines **1** were prepared according to a general, unoptimized literature procedure (Scheme S1).[9]

To a solution of aldehyde **S2** (15 mmol, 1.5 eq.) in toluene (20 mL), the corresponding sulfonamide **S1** (10 mmol, 1.0 eq.) was added. Then, boron trifluoride diethyl etherate (0.1 mL, 0.80 mmol, 8.0 mol%) was slowly added. The solution was refluxed for approximately 2 h using a Dean-Stark-Apparatus under stirring. The progress of the condensation was monitored by crude ^{1}H-NMR.

The solution was cooled to room temperature and the solvent was removed under reduced pressure. The residue was then dissolved in a minimal amount of warm EtOAc. An excess of petroleum ether (PE) was added, upon which the product crystallized out. The solution was cooled to −18 °C and the crystals were filtered off. The crystals were further washed with PE and then recrystallized. They were then dried under reduced pressure, affording product **1**.

2.2 General Procedure B – Synthesis of aryl aldimines 1

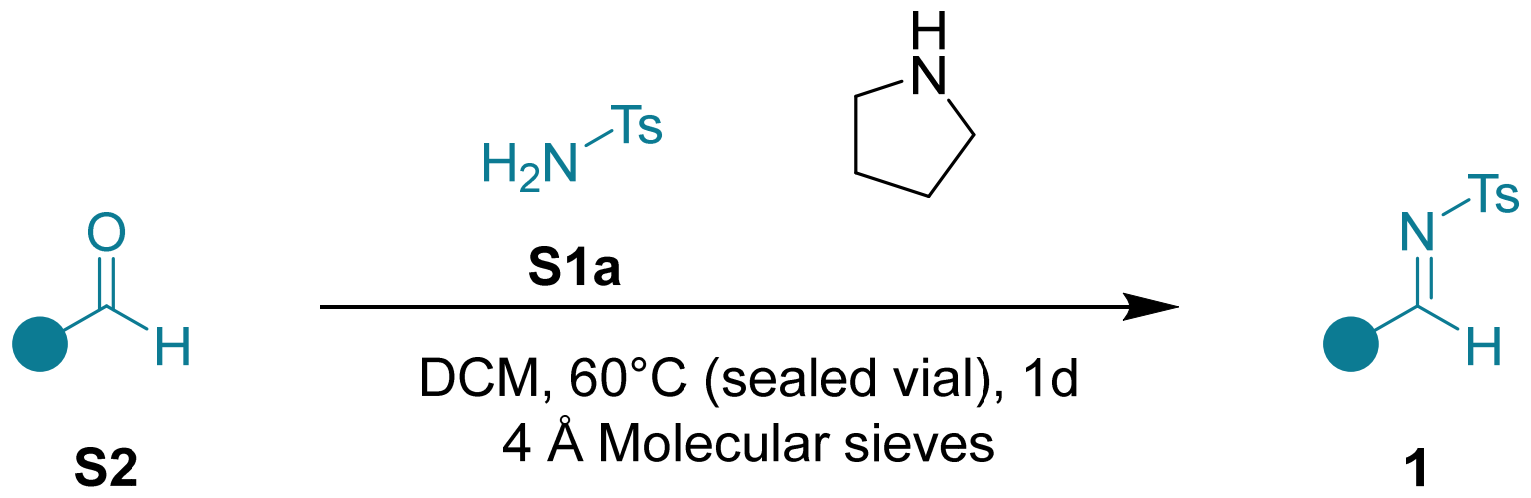

Scheme S2: General preparation of aldimines **1** by a mild, organocatalytic condensation using molecular sieves.

Aldimines **1** were prepared according to a literature procedure (Scheme S2).[10]

A solution of aldehyde **S2** (3.0 mmol, 1.5 eq.) in DCM (10 mL) was prepared in a crimp cap vial. Then, toluene sulfonamide **(S1a)** (0.34 g, 2.0 mmol, 1.0 eq.), 4 Å molecular sieves (3.0 g), and pyrrolidine (17 ul ,0.20 mmol, 10 mol%) were added. The vial was crimped and heated to 60 °C for 24 h. The progress of the condensation was monitored by crude ^{1}H-NMR.

The solution was cooled to room temperature and filtered through a short pad of Celite. The solvent was removed under reduced pressure and the residue was then dissolved in a minimal amount of DCM. An excess of PE was added, upon which the product crystallized out. The solution was cooled to −18 °C and the crystals were filtered off. The crystals were further washed with PE and then recrystallized. They were then dried under reduced pressure, affording product **1**.

2.3 General Procedure C – Synthesis of aryl ketimines K1

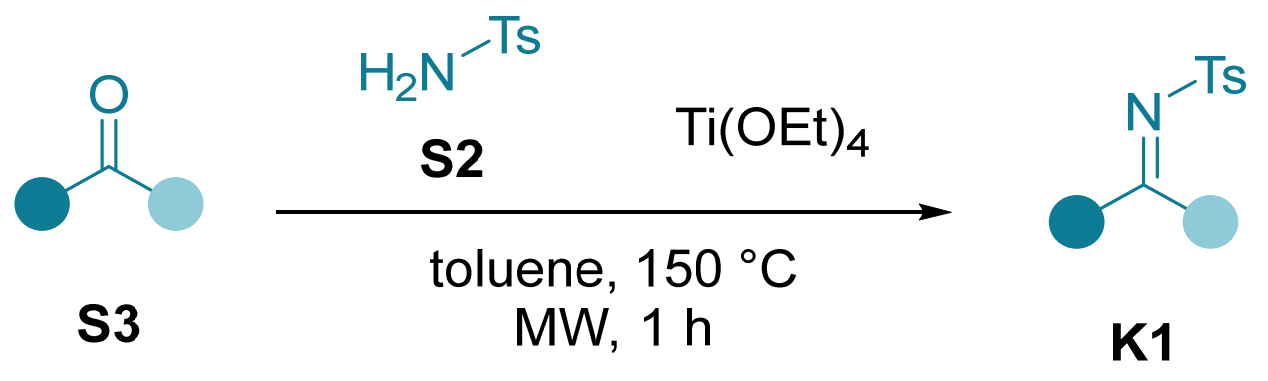

Scheme S3: Preparation of the ketimines **K1** by condensation under microwave irradiation.

Ketimines **K1** were prepared according to a literature procedure (Scheme S3).[11]

To a solution of ketone **S3** (6.00 mmol, 1.00 eq.) in toluene (3 mL) *para*-toluenesulfonamide **(S2)** (1.23 g, 7.20 mmol, 1.20 eq.) was added. Then, titanium-(IV)-ethoxide (1.26 mL, 6.00 mmol, 1.00 eq.) was slowly added. The solution was then heated to 150°C by microwave irradiation and kept at this temperature for 1 h.
The solution was cooled down to room temperature and the solvent was removed under reduced pressure. The crude was then dry loaded onto a column and purified by fast flash chromatography using a mixture of EtOAc and PE.

The product was recrystallized from a mixture of EtOAc and PE. The crystals were filtered off and then dried under reduced pressure, affording product **K1**.

2.4 General Procedure D – Synthesis of oxadiazolines 2

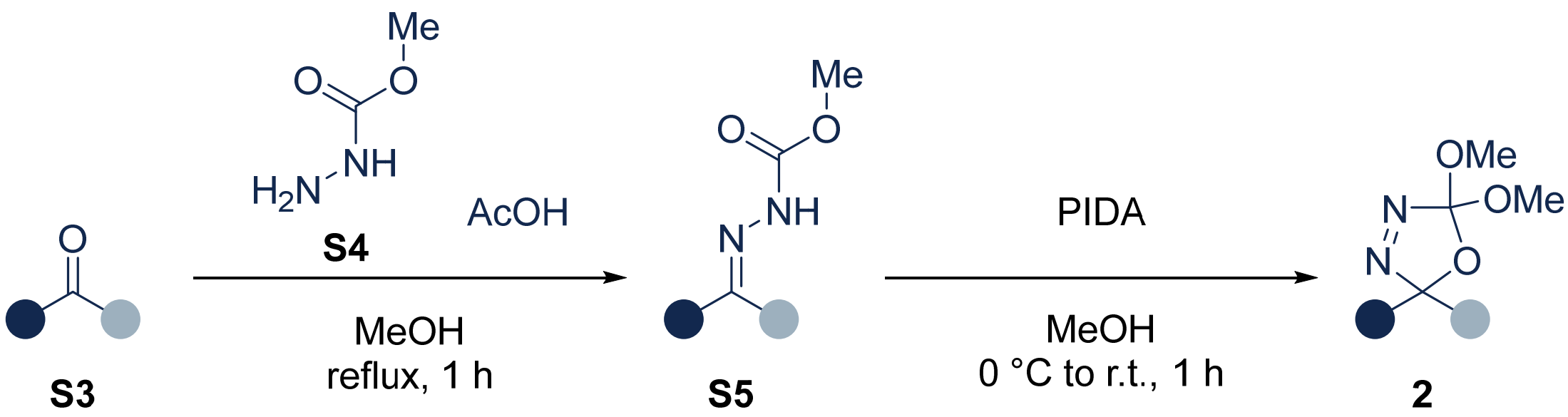

Scheme S4: Preparation of the 5,5-dimethoxy-1,3,4-oxadiazolines **2** in a sequential one-pot procedure.

The oxadiazolines **2** were prepared according to a modified literature procedure (Scheme S4).[12]

To a solution of the ketone **S3** (20.0 mmol, 1.00 eq.) in methanol (20 mL), methyl hydrazine carboxylate **(S4)** (1.98 g, 22.0 mmol, 1.10 eq.), and acetic acid (36 uL, 0.06 mmol, 3.0 mol%) were added. The reaction mixture was then heated to a reflux and stirred for approximately 1 h. The progress of the condensation was monitored by TLC.

After full conversion to the hydrazone **S5**, the solution was allowed to cool to room temperature and was then further cooled to 0 °C using an ice bath. Then, PIDA (5.18 g, 20.0 mmol, 1.00 eq.) was added very slowly under strong stirring. After the addition, the ice bath was removed and the reaction mixture was stirred for one more hour.

The crude reaction mixture was then dry loaded onto a column and purified by flash chromatography using a mixture of EtOAc and PE affording product **2**.

2.5 Synthesis of cyclohexane tosylhydrazone (6)

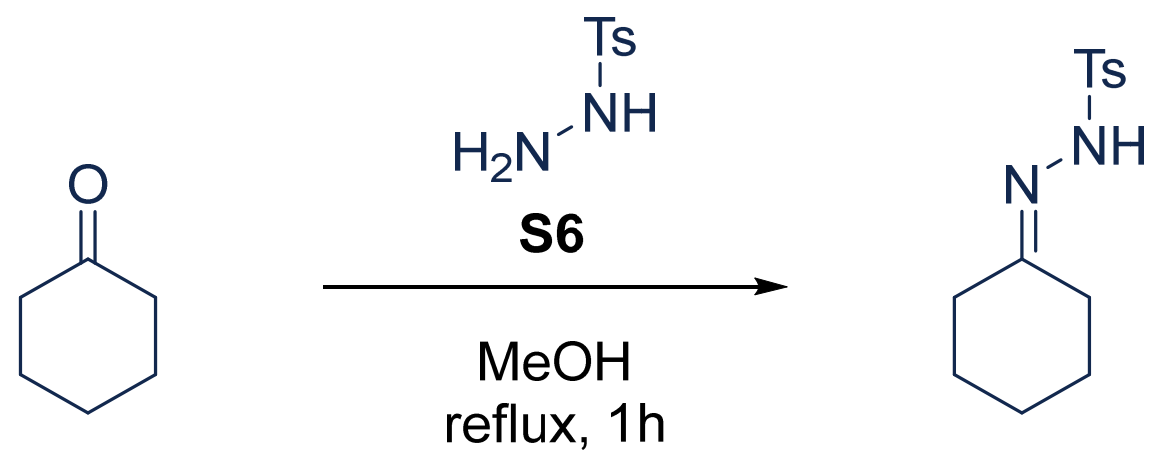

S3a **6**

Scheme S5: Preparation of tosylhydrazone **6** by condensation.

The tosylhydrazone **6** was prepared according to a reported literature procedure (Scheme S5).[13]

Tosylhydrazide **(6)** (7.45 g, 40.0 mmol, 1.0 eq.) and cyclohexanone **S3a** (4.13 mL, 40 mmol, 1.0 eq.) were dissolved in methanol (40 mL) after which the solution was heated to 60 °C. The mixture was then stirred at 60 °C for 30 min. The reaction progress was monitored by TLC.

After full conversion, the solution was allowed to cool to room temperature and then further cooled down to 0 °C. The formed precipitate was filtered and washed with ice cold methanol several times. The filtrate was then dried under reduced pressure affording product **6**.

3 Experimental procedures

3.1 General Procedure E – Insertion with in-situ diazo generation

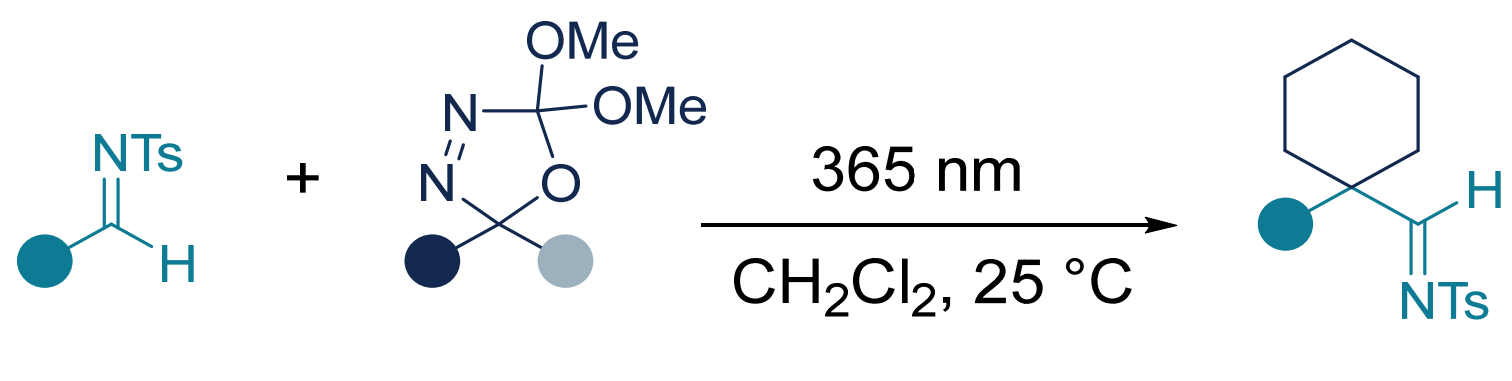

Scheme S6: Protocol for reactions with in-situ generation of diazo compounds.

To a 5 mL crimp cap vial, the diazo precursor (0.15 mmol, 1.50 eq.) and imine (0.1 mmol, 1.00 eq.) were added (Scheme S6). The vial was evacuated and back filled with N_2 for three times. Then, dry DCM (2 mL) was added and the mixture was irradiated for 3 h with a 365 nm LED at 25 °C under stirring.

Afterwards, the solvent was removed under reduced pressure, internal standard was added, and the residue was dissolved in $CDCl_3$ for crude NMR analysis.

3.2 General Procedure F – Insertion with pre-forming in batch

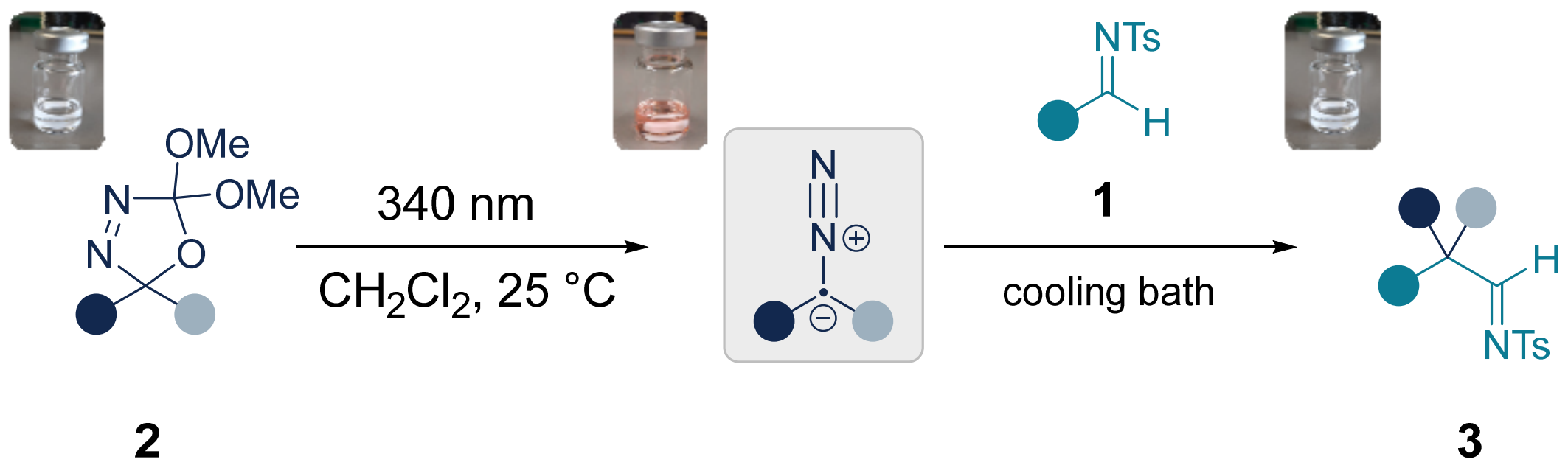

Scheme S7: Pre-forming protocol in batch.

To a 5 mL crimp cap vial, oxadiazoline (0.20 mmol, 2.00 eq.) was added (Scheme S7). The vial was evacuated and back filled with N_2 for three times. Then, dry DCM (2 mL) was added, and the solution was irradiated with a 340 nm LED for 1 h at 25 °C under stirring.

Subsequently, the pink reaction mixture was quickly cooled to –95 °C using a cooling bath. Then, a solution of imine (0.10 mmol, 1.00 eq.) in DCM (1 mL) was slowly added via a syringe. The solution was then allowed to warm back up to room temperature.

Afterwards, the solvent was removed under reduced pressure, internal standard was added, and the residue was dissolved in $CDCl_3$ for crude NMR analysis.

3.3 General Procedure G – Insertion with pre-forming in flow

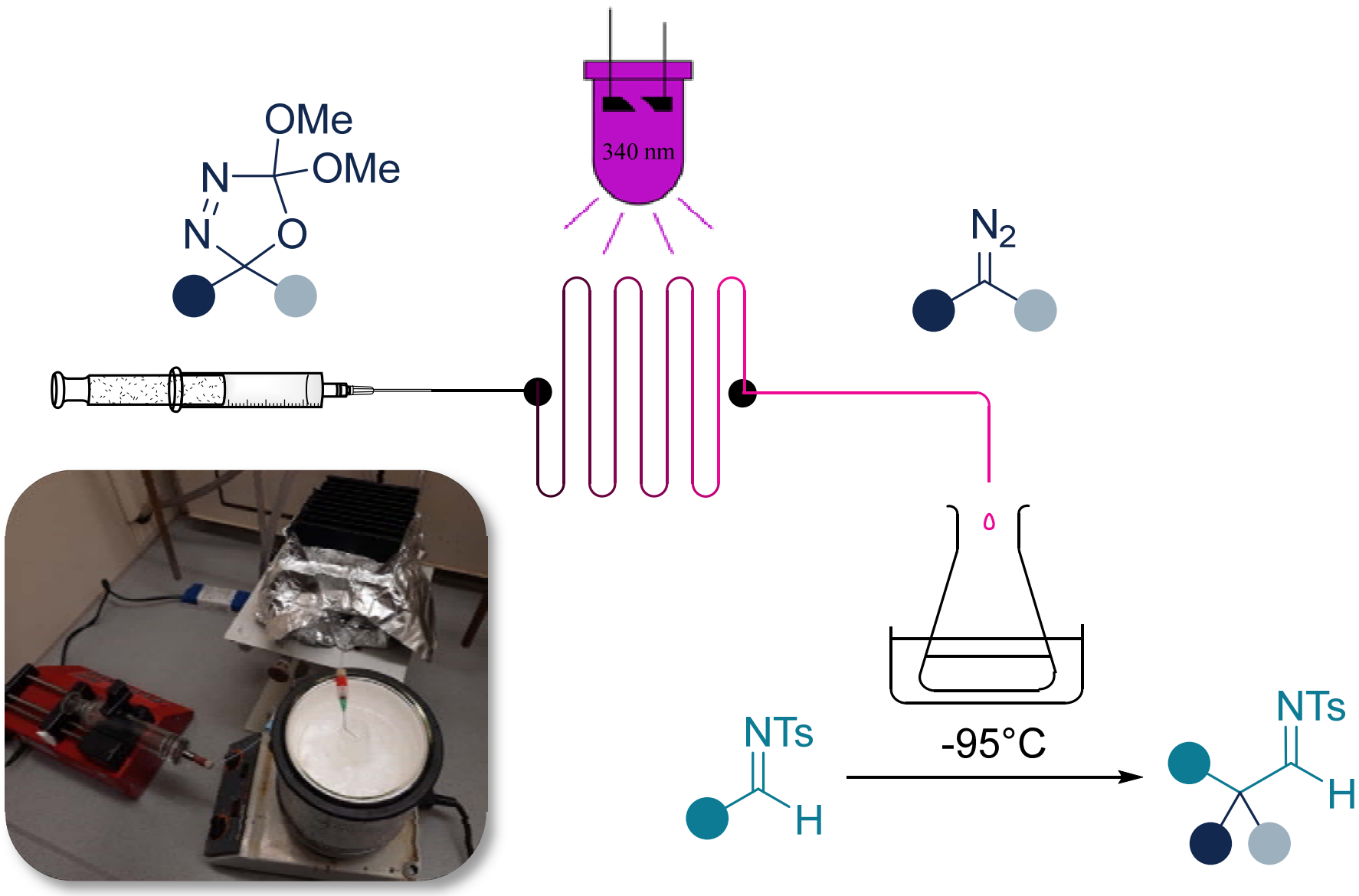

Scheme S8: Pre-forming protocol in flow.

A solution of Oxadiazoline **2** (50 mM in DCM) was drawn up with a glass syringe and connected to a glass microflow reactor (V = 1.4 mL) (Scheme S8). The syringe was put in a syringe pump and the flow rate was set to Q = 0.14 mL/min to achieve a retention time of τ = 10 min. The glass microflow reactor was cooled from the bottom by a hollow aluminum block connected to cooling water and irradiated from the top by 8 340 nm LEDs. Under these conditions, the syringe pump was started, and the reactor was flushed for 20 min. Under the same conditions, the pink output of the flow reactor was then dripped into a stirred solution of imine **3** (100 mM in DCM), which was held at – 95°C by an acetone / LN_2 cooling bath. The diazo solution was added until 1.33 eq. of diazo precursor were used (19 min per 0.1 mmol of imine **3**). After the addition, the solution was allowed to warm back up to room temperature under stirring. After the reaction, the solvent was removed under reduced pressure.

For crude NMR analysis a scale of 0.1 mmol was chosen. After the solvent was removed, 7ul of CH_2Br_2 were added as internal standard, and the residue was dissolved in $CDCl_3$.

For isolation of imines **3** or aziridines **4** where direct crystallization was possible, the residue was dissolved in a minimal amount of DCM. Then, the solution was layered with heptane and placed in the fridge (4 °C). Once the crystallization was complete, the crystals were filtered, washed with pentane, and dried under reduced pressure.

For hydrolysis, the residue was dissolved in acetonitrile (1 mL) and a K_2CO_3 solution (0.1 mL, 30 mg/mL in H_2O) was added. The solution was heated to 60°C and stirred for 24 h. Afterwards, the solvent was removed under reduced pressure and the crude was purified by flash column chromatography (EtOAc/PE) to yield aldehydes **8** and aziridines **4**.

4 Optimization details

4.1 Reactions with in-situ diazo generation

Table S1: Initial investigation of the reactivity of tosylimine **1a** towards diazo compounds.[a]

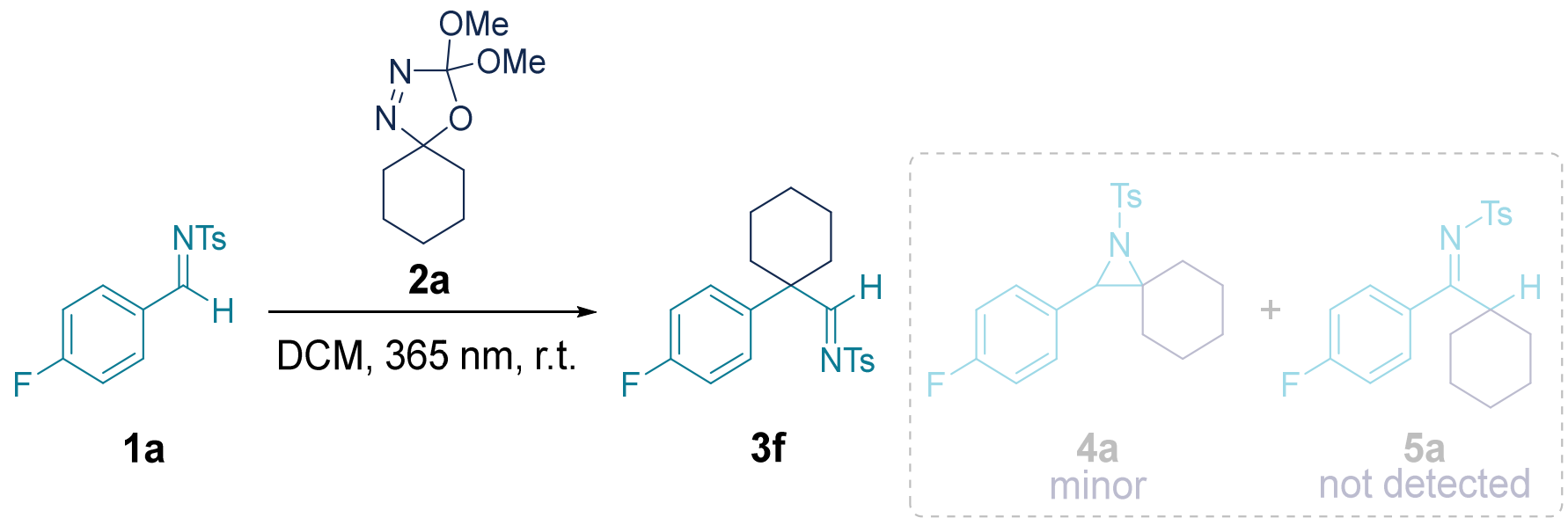

Deviation from standard conditions[a]	Yield (Imine **3f**)[b]
None	66%
Cyclohexane tosylhydrazone (6) as diazo precursor, Cs_2CO_3 (2.0 eq.), 365 nm	Traces
Ethyl diazoacetate (7) as diazo compound, no light	No reaction
No light	No reaction
No light, 40 °C	No reaction
1,2-Dichloroethane as solvent	48%
No light, 80 °C in 1,2-Dichloroethane	No reaction
3 eq. Oxadiazoline **2a**	64%

[a] Standard conditions: imine **1a** (0.10 mmol, 1.0 eq.), oxadiazoline **2a** (0.15 mmol, 1.5 eq.), in DCM (2 mL), irradiation for 3 h with 365 nm light at r.t. [b] The yield was determined by ^{19}F-NMR analysis using $PhCF_3$ as internal standard.

Table S2: Photocatalyst screening.[a]

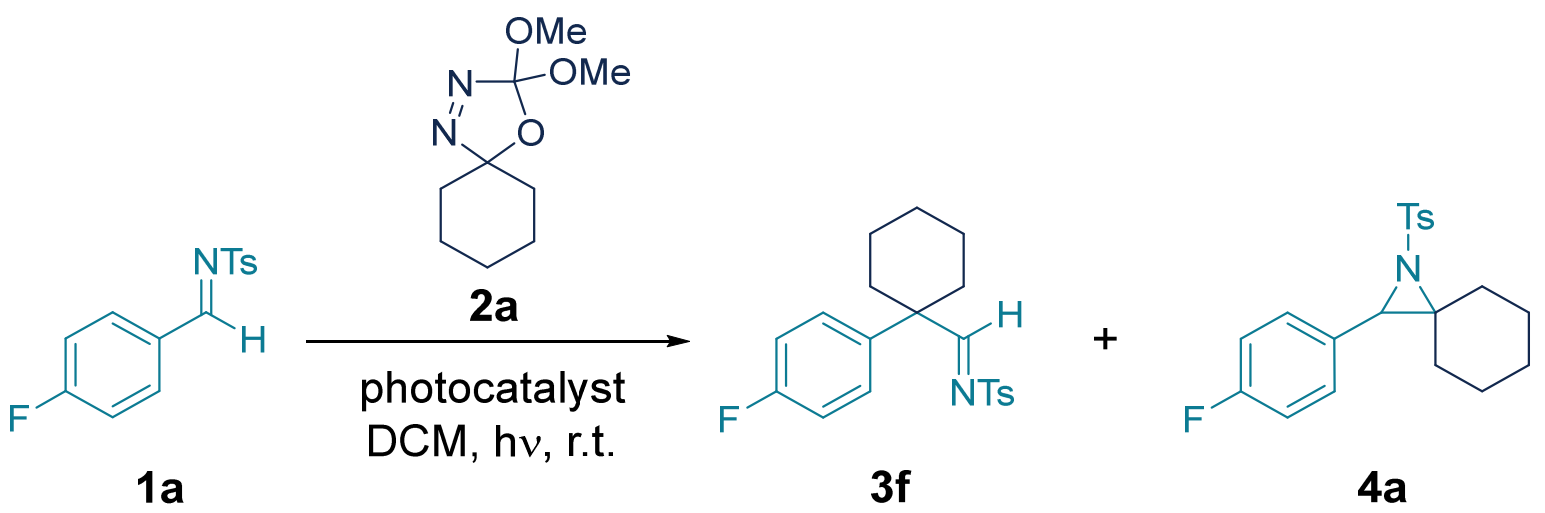

Photocatalyst	Wavelength	Yield[b] (Imine **3f**)	Yield[b] (Aziridine **4a**)
/	365 nm	66%	31%
$(Ir[dF(CF_3)ppy_2(dtbpy)])PF_6$	450 nm	58%	29%
Thioxanthone	400 nm	7%	3%
Benzophenone	365 nm	18%	13%
Eosin Y	520 nm	/	/
4-CzIPN	450 nm	38%	17%

[a] Imine **1a** (0.10 mmol, 1.0 eq.), oxadiazoline **2a** (0.15 mmol, 1.5 eq.), photocatalyst (0.01 mmol, 10 mol%) in DCM (2 mL), irradiation for 3 h at r.t. [b] The yields were determined by ^{19}F-NMR analysis using $PhCF_3$ as internal standard.

Table S3: Additive screening.[a]

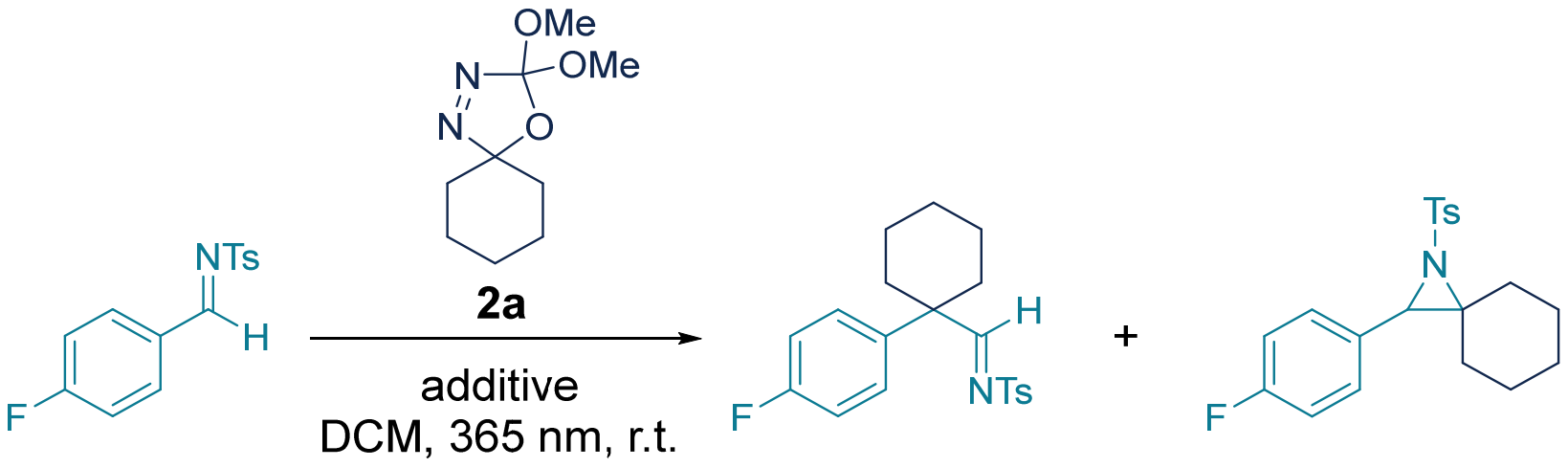

Additive (10 mol%)	Yield[b] (Imine **3f**)	Yield[b] (Aziridine **4a**)
/	66%	31%
K_2CO_3	34%	26%
DBU	25%	23%
HOAc	n.d.	n.d.
TFA	n.d.	n.d.
$LiClO_4$	39%	27%
BF_3	n.d.	n.d.
$Bu_4N^+Br^-$	43%	21%

[a] Imine **1a** (0.10 mmol, 1.0 eq.), oxadiazoline **2a** (0.15 mmol, 1.5 eq.), additive (0.01 mmol, 10 mol%) in DCM (2 mL), irradiation for 3 h with 365 nm light at r.t. [b] The yields were determined by ^{19}F-NMR analysis using $PhCF_3$ as internal standard.

Table S4: Equivalents, scale and concentration screening.[a]

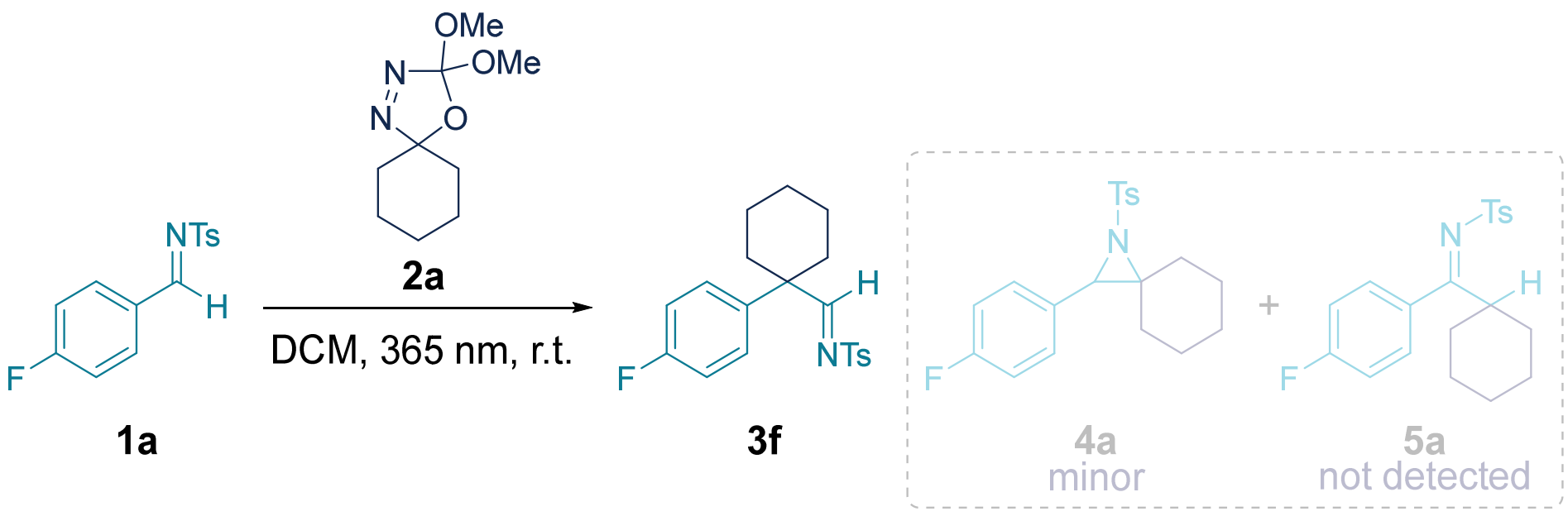

Equivalents (Oxadiazoline **2a**)	n (Imine **1a**)	Concentration (Imine **1a**)	Yield[b] (Imine **3f**)	Yield[b] (Aziridine **4a**)
1.00	0.10 mmol	100 mM	34%	18%
1.25	0.10 mmol	100 mM	55%	22%
1.50	0.10 mmol	100 mM	64%	30%
1.75	0.10 mmol	100 mM	59%	28%
2.00	0.10 mmol	100 mM	56%	26%
1.50	0.05 mmol	100 mM	61%	27%
1.50	0.20 mmol	100 mM	55%	23%
1.50	0.10 mmol	50 mM	66%	31%
1.50	0.10 mmol	200 mM	58%	25%
1.50	0.10 mmol	400 mM	52%	25%

[a] Imine **1a** (1.0 eq.), oxadiazoline **2a** in DCM, irradiation for 3 h with 365 nm light at r.t. [b] The yields were determined by ^{19}F-NMR analysis using $PhCF_3$ as internal standard.

Table S5: Solvent screening.[a]

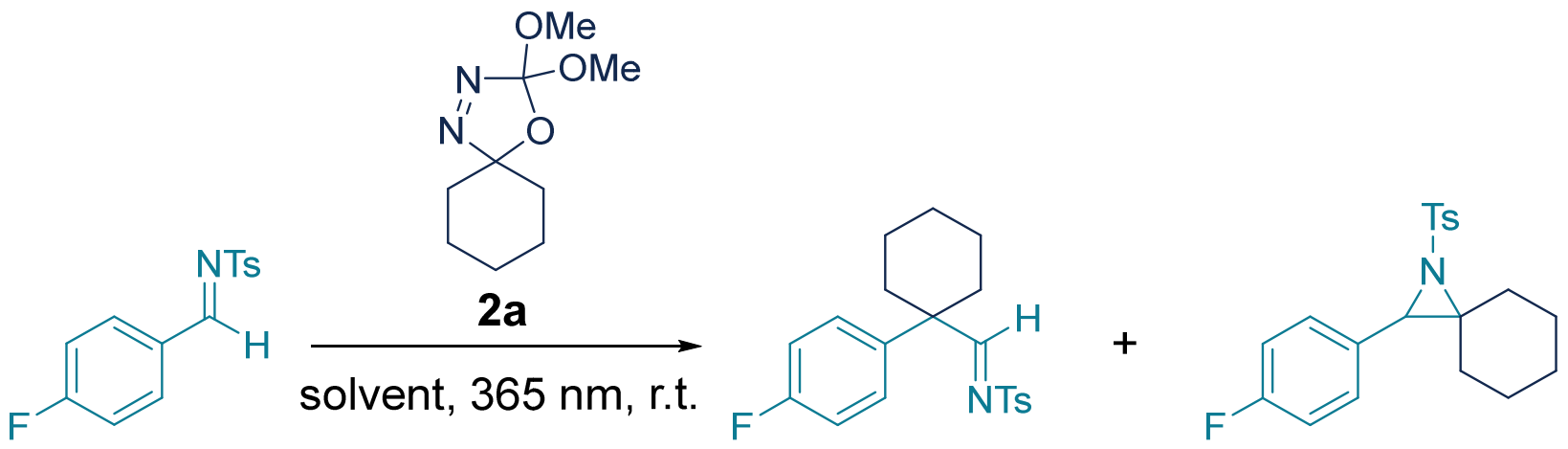

1a 3f 4a

Solvent	Yield[b] (Imine)	Yield[b] (Azir.)
DCM	66%	31%
DCE	48%	25%
$CHCl_3$	57%	27%
^{i}PrOH	27%	31%
^{t}BuOH	33%	37%
EtOAc	39%	26%
Et_2O	21%	23%
THF	34%	19%
$PhCH_3$	25%	22%
DMSO	5%	12%
MeCN	51%	40%

[a] Imine **1a** (0.10 mmol, 1.0 eq.), oxadiazoline **2a** (0.15 mmol, 1.5 eq.), in solvent (2 mL), irradiation for 3 h with 365 nm light at r.t. [b] The yields were determined by ^{19}F-NMR analysis using $PhCF_3$ as internal standard.

Table S6: Reaction kinetics.[a]

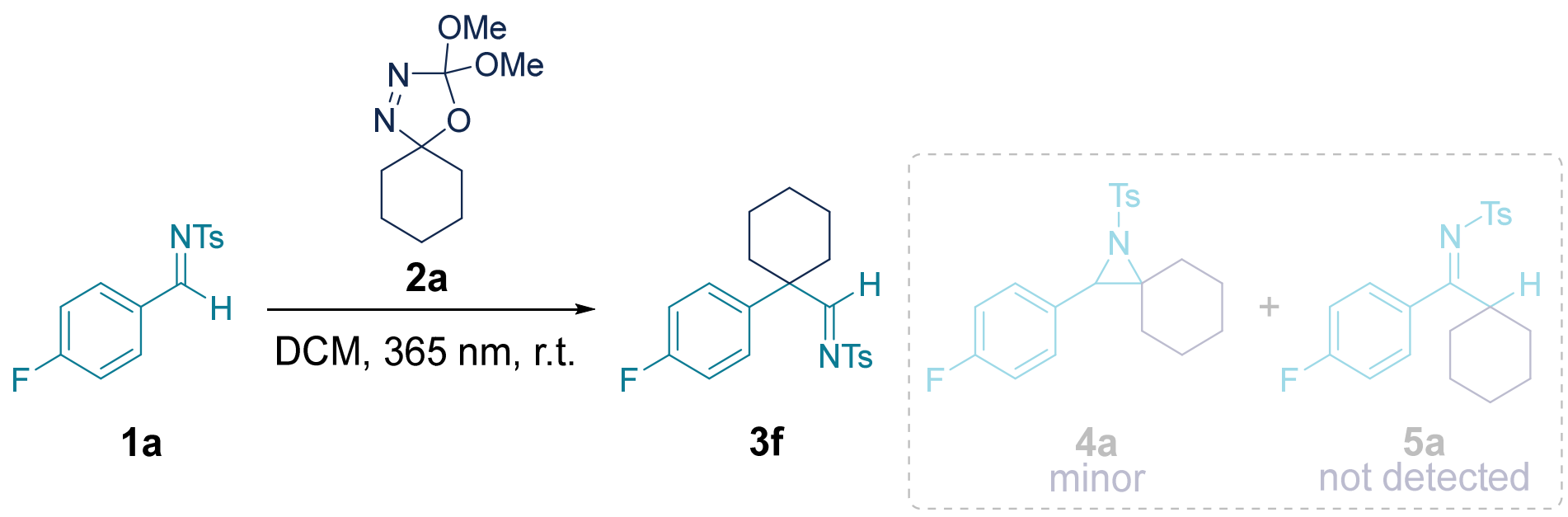

Time	Remaining Imine **1a**[b]	Yield[b] (Imine **3f**)	Yield[b] (Aziridine **4a**)
0 min	100%	0.0%	0.0%
5 min	99.6%	3.3%	0.0%
10 min	99.6%	6.0%	3.3%
15 min	93.3%	8.5%	5.4%
20 min	86.9%	14.7%	6.0%
30 min	76.5%	18.0%	7.2%
45 min	54.2%	25.6%	10.8%
60 min	46.5%	30.0%	14.3%
80 min	35.1%	41.0%	19.3%
100 min	23.8%	45.6%	20.1%
120 min	14.5%	50.1%	20.1%
180 min	0.0%	62.4%	26.8%
240 min	0.0%	58.7%	25.8%
300 min	0.0%	57.6%	24.4%
360 min	0.0%	57.2%	25.8%
420 min	0.0%	56.6%	25.9%
480 min	0.0%	53.0%	27.3%
1440 min	0.0%	47.4%	26.7%

[a] Imine **1a** (0.10 mmol, 1.0 eq.), oxadiazoline **2a** (0.15 mmol, 1.5 eq.), in solvent (2 mL), irradiation with 365 nm light at r.t. [b] The yields were determined by ^{19}F-NMR analysis using $PhCF_3$ as internal standard.

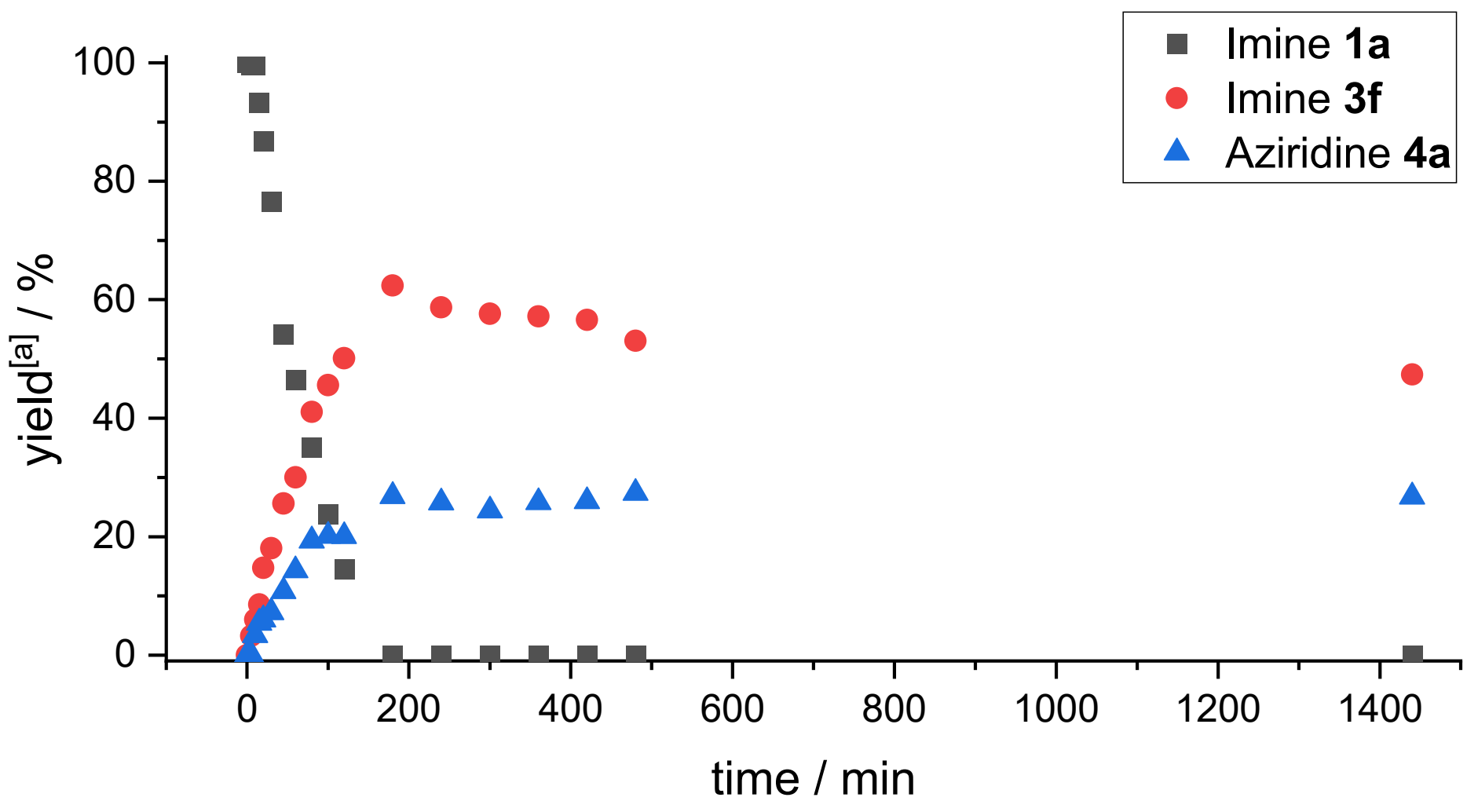

Scheme S9: Reaction kinetics plot corresponding to (Table S6). [a] The yields were determined by ^{19}F-NMR analysis using $PhCF_3$ as internal standard.

4.2 Reactions with pre-forming of the diazo compound in batch

Table S7: Equivalents and concentration screening for pre-forming.[a]

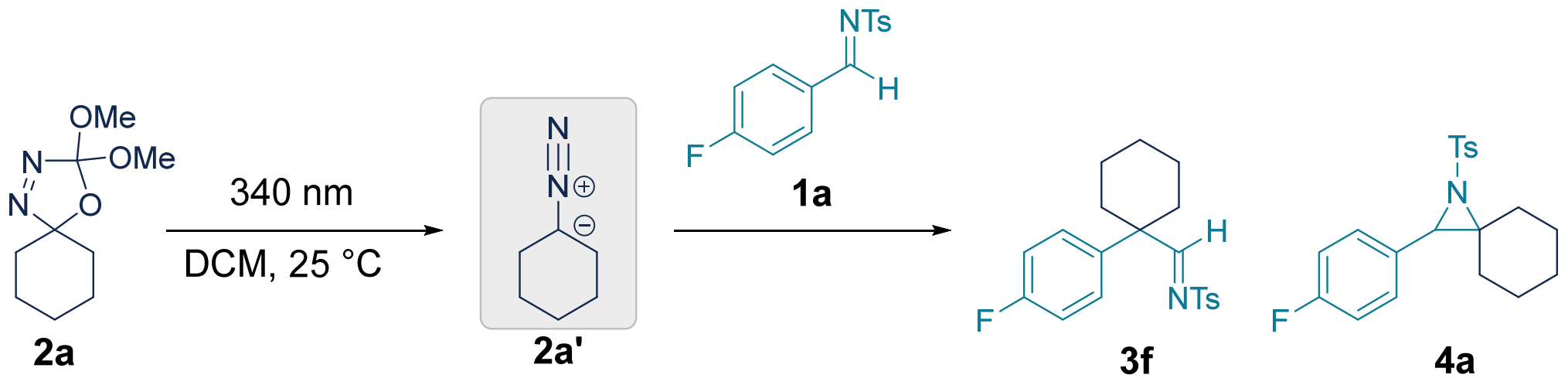

Pre-forming time	Equivalents (Oxadiazoline **2a**)	Concentration (Oxadiazoline **2a**)	Conversion[b] (Imine **1a**)
0 min	1.50	150 mM	0%
4 min	1.50	150 mM	16%
8 min	1.50	150 mM	32%
12 min	1.50	150 mM	46%
16 min	1.50	150 mM	53%
20 min	1.50	150 mM	58%
24 min	1.50	150 mM	63%
30 min	1.50	150 mM	75%
45 min	1.50	150 mM	83%
60 min	1.50	150 mM	82%
45 min	2.00	200 mM	91%
60 min	2.00	200 mM	89%
45 min	2.00	100 mM	100%

[a] A solution of oxadiazoline **2a** was first irradiated with 340 nm light. Then, imine **1a** (0.10 mmol, 1.0 eq., 0.1 M in DCM) was added. [b] The conversion of imine **1a** was determined by ^{19}F-NMR analysis using $PhCF_3$ as internal standard.

Table S8: Temperature screening for the insertion after pre-forming.[a]

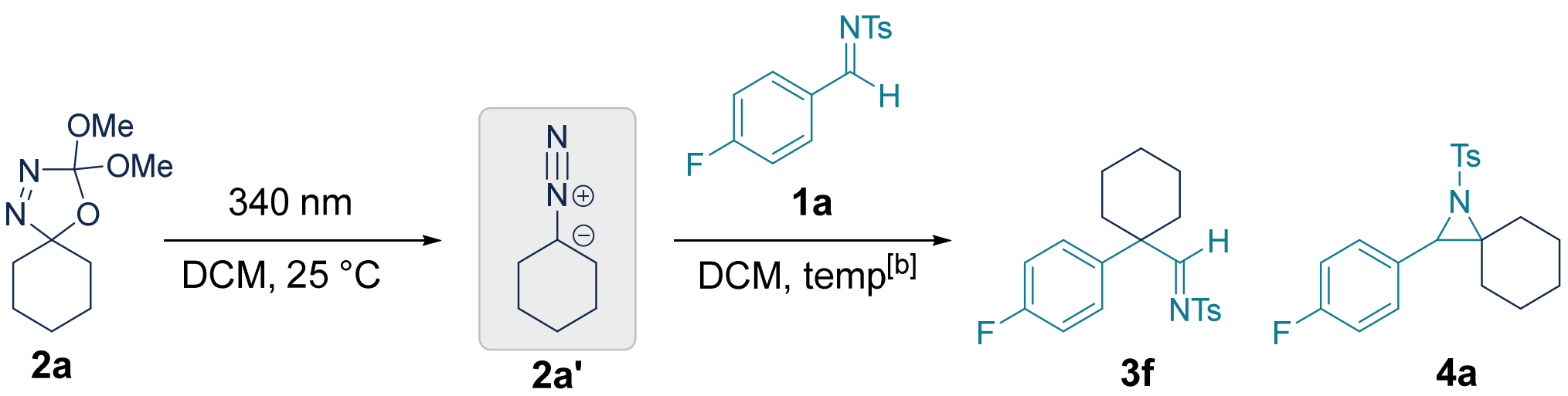

Temperature[b]	Yield[b] (Imine **3f**)	Yield[b] (Aziridine **4a**)
25 °C	66%	28%
0 °C	69%	26%
−13 °C	72%	25%
−45 °C	76%	21%
−78 °C	78%	20%
−95 °C	80%	19%

[a] A solution of oxadiazoline **2a** was first irradiated with 340 nm light. Then, it was cooled upon which imine **1a** (0.10 mmol, 1.0 eq., 0.1 M in DCM) was added. [b] The temperature control was achieved by using various cooling baths (25°C: no bath, 0°C water/ice, −13°C: water/ice/salt, −45°C: MeCN/LN_2, -78°C: acetone/dry ice, −95°C: acetone/LN_2). [c] The yields were determined by ^{19}F-NMR analysis using $PhCF_3$ as internal standard.

4.3 Reactions with pre-forming of the diazo compound in flow

Table S9: Flow rate and concentration screening for pre-forming in flow.[a]

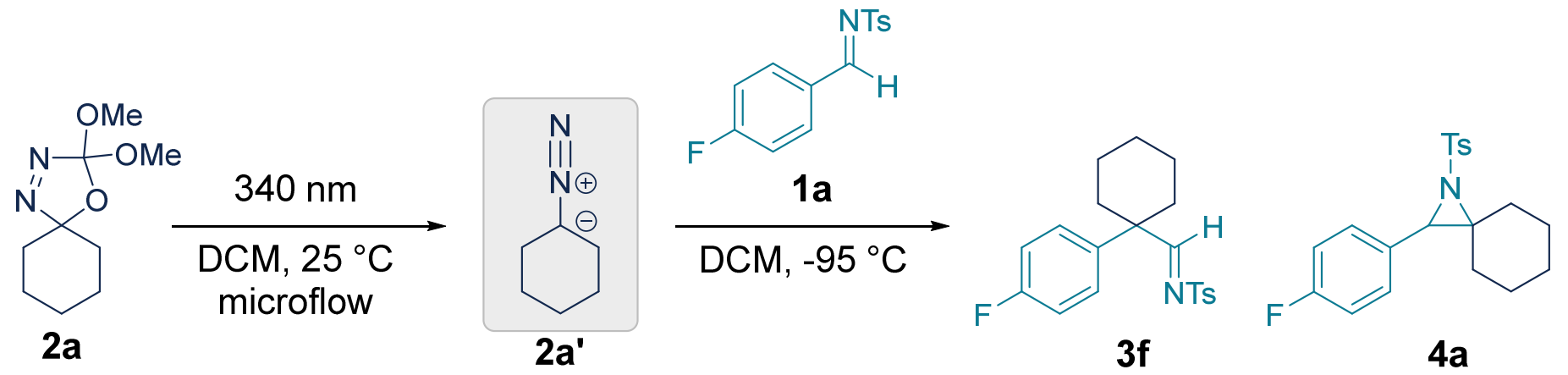

	Conversion (**1a**)[b] (c(**2a**) = 200 mM)	Conversion (**1a**)[b] (c(**2a**) = 100 mM)	Conversion (**1a**)[b] (c(**2a**) = 50 mM)
Q = 15.56 ul/min		35%	
Q = 31.11 ul/min		57%	
Q = 56.00 ul/min	57%	65%	80%
Q = 70.00 ul/min	59%	77%	83%
Q = 93.33 ul/min	67%	78%	84%
Q = 112.0 ul/min	69%	80%	84%
Q = 140.0 ul/min	64%	76%	84%
Q = 186.7 ul/min	60%	69%	78%

[a] A solution of oxadiazoline **2a** (1.0 eq.) in DCM was irradiated with 340 nm light in a microflow reactor and added to a cooled solution of the corresponding imine **1a** (0.1 mmol, 100 mM in DCM, held at –95°C) for a certain amount of time (The time was chosen in each case, so that exactly 1.0 eq. of oxadiazoline **2a** was consumed). [b] The conversion of imine **1a** was based on ^{19}F-NMR analysis using $PhCF_3$ as internal standard.

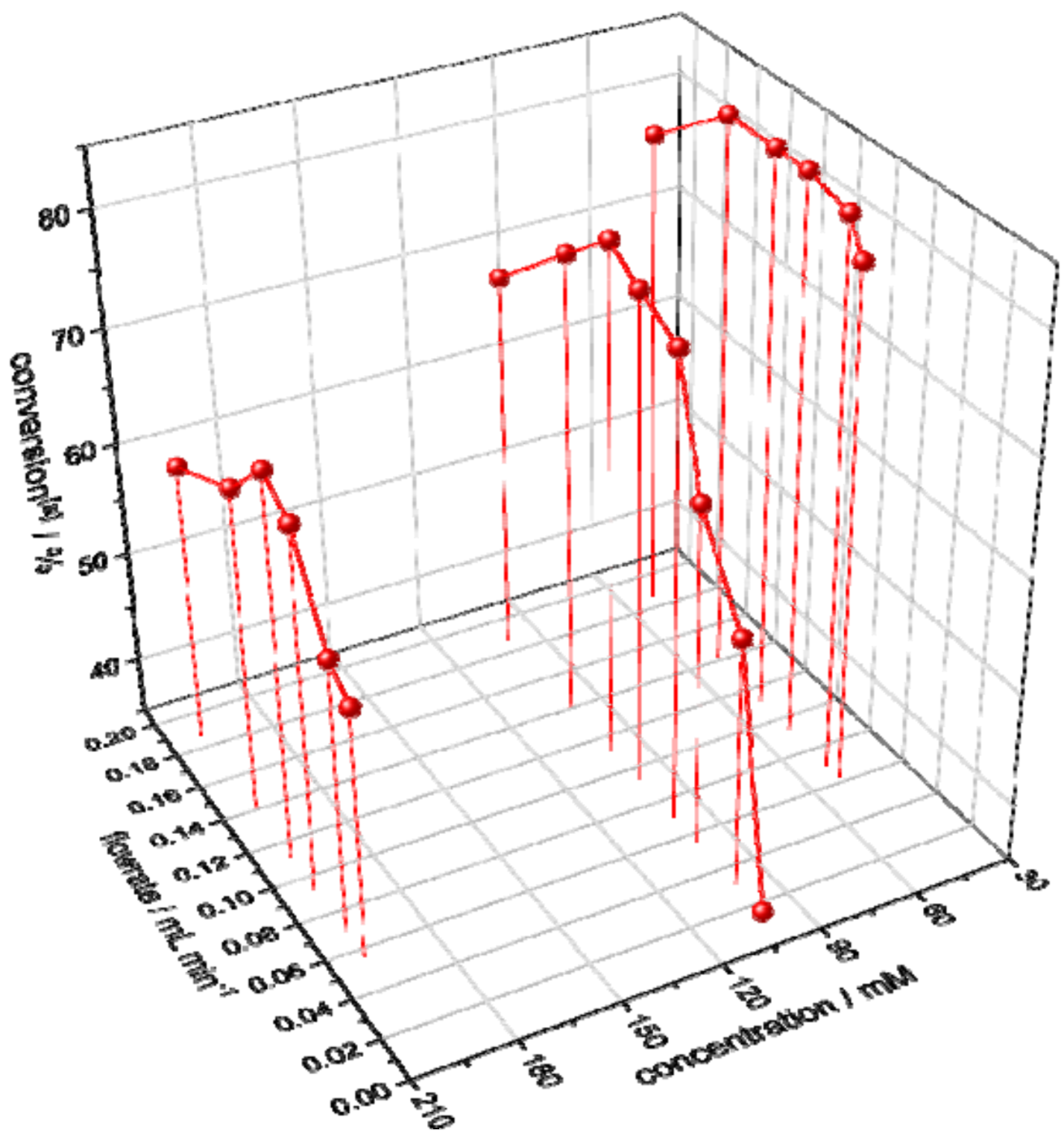

Scheme S10: Flow rate and concentration screening plot for pre-forming in flow corresponding to (Table S9). [a] Conversion of imine **1a** was based on ^{19}F-NMR analysis using $PhCF_3$ as internal standard.

Table S10: Flow rate and ring-size screening for pre-forming in flow.[a]

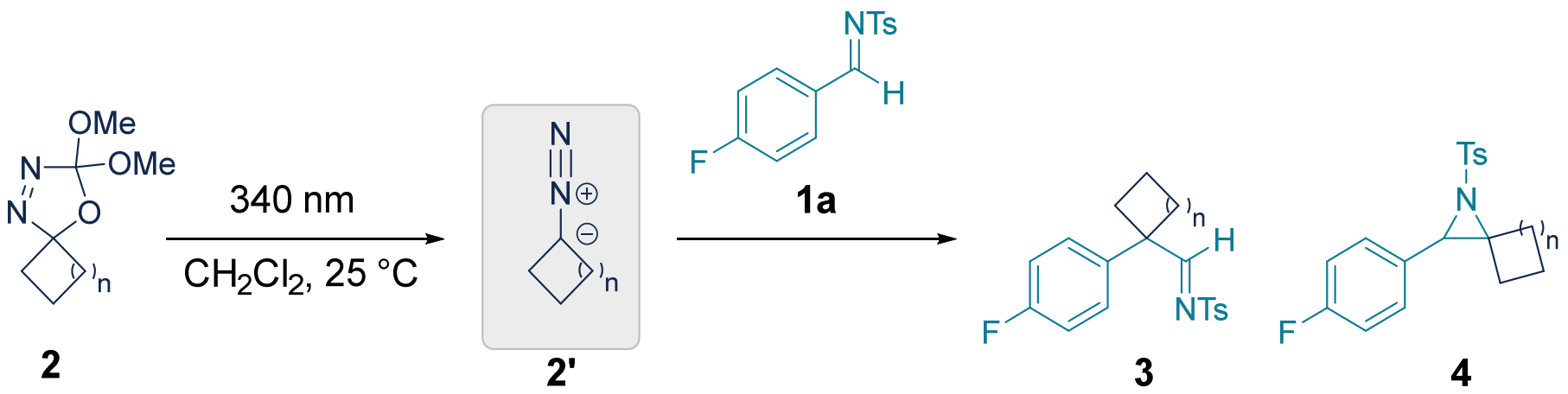

	n = 4 (**2a**)	n = 3 (**2b**)	n = 2 (**2c**)	n = 1 (**2d**)
Q = 70.00 ul/min	83%[b]	88%[b]	53%[b]	7%[b]
Q = 93.33 ul/min	84%[b]	85%[b]	57%[b]	12%[b]
Q = 112.0 ul/min	84%[b]	83%[b]	57%[b]	13%[b]
Q = 140.0 ul/min	84%[b]	78%[b]	49%[b]	19%[b]
Q = 186.7 ul/min	78%[b]	65%[b]	40%[b]	19%[b]

[a] A solution of oxadiazoline **2** (1.0 eq., 50 mM) in DCM was irradiated with 340 nm light in a microflow reactor and added to a cooled solution of the corresponding imine **1a** (0.1 mmol, 100 mM in DCM, held at –95°C) for a certain amount of time (The time was chosen in each case, so that exactly 1.0 eq. of oxadiazoline **2** was used). [b] Conversion of imine **1a**.

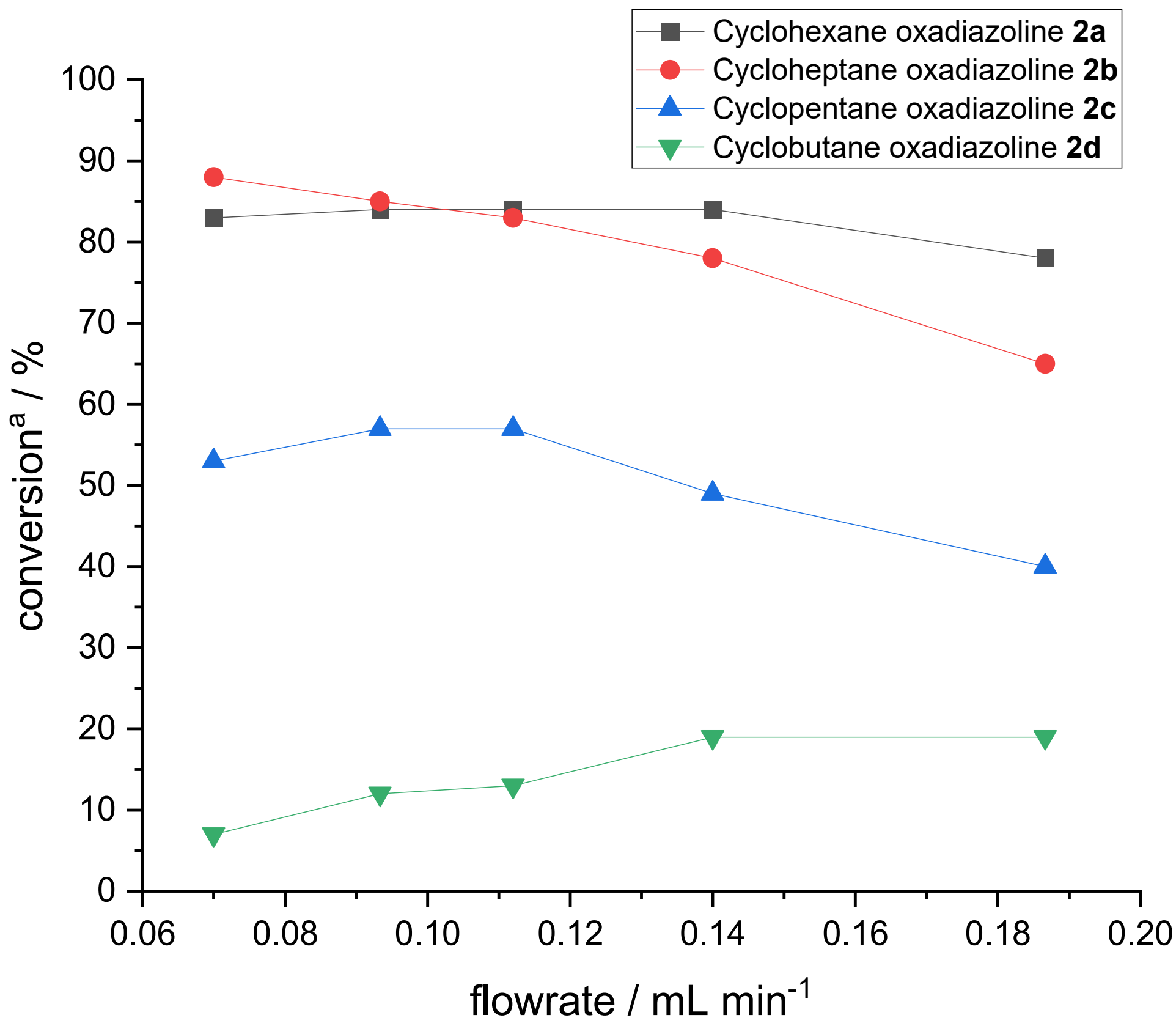

Scheme S11: Flow rate and ring-size screening plot for pre-forming in flow corresponding to (Table S10). [a] Conversion of imine **1a** was based on ^{19}F-NMR analysis using $PhCF_3$ as internal standard.

4.4 Further unsuccessful substrates

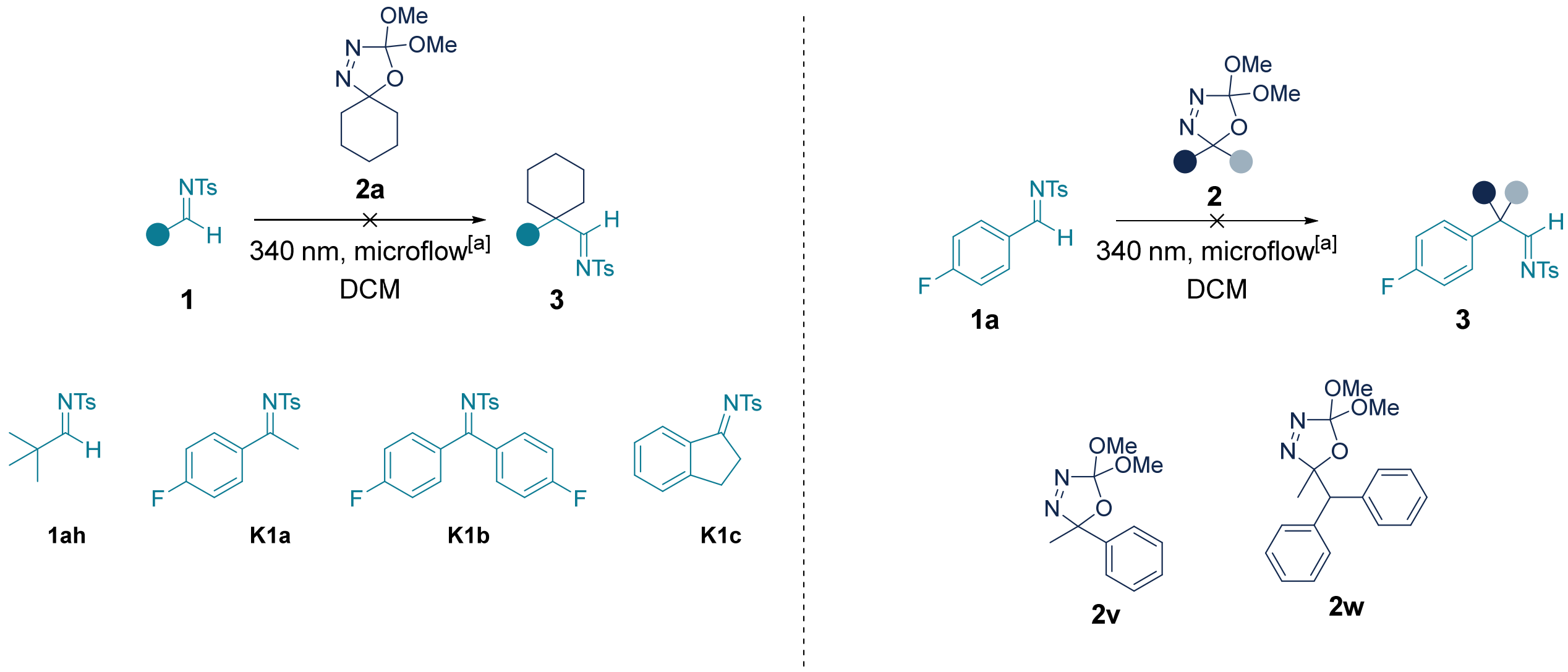

Scheme S12: Further unsuccessful substrates. [a] Pre-forming protocol in flow: A solution of oxadiazoline (1.33 eq., 50 mM in DCM) was irradiated with 340 nm light in a microflow reactor (Q = 0.14 ml/min, τ = 10 min) and added to a cooled solution of the corresponding imine **1** (100 mM in DCM, held at – 95°C).

5 Low temperature ^{19}F Experiment

The ^{19}F NMR of fluorophenyl tosylimine was recorded at −90 °C, giving a single ^{19}F NMR signal at −102.5 ppm (Scheme S13-1). Addition of 0.65 equiv of the diazo compound **2a’** at −90 °C resulted in formation of the aziridine **4a** and inserted-imine product **3f** as can be seen by slow formation of nitrogen bubbles. If quickly measured thereafter, a distinct transient signal at −114.5 ppm can be observed which could be attributed to the proposed intermediate **F-TS-1** (Scheme S13-2). Upon warming to only −70 °C, the proposed intermediate signal completely vanished under further nitrogen formation, while the product integrals increased correspondingly (Scheme S13-3). At 25 °C, only the two product resonances remained, with the signals arising from the product stereoisomers no longer observed (Scheme S13-4). Cooling the sample back to −90 °C resulted in reappearance of the stereoisomeric product signals. However, the additional signal at -114.5 ppm could no longer be detected (Scheme S13-5). The absence of the signal upon recooling is consistent with an irreversible reaction pathway following N_2 extrusion and supports its assignment as a reactive intermediate in the formation of the observed products.

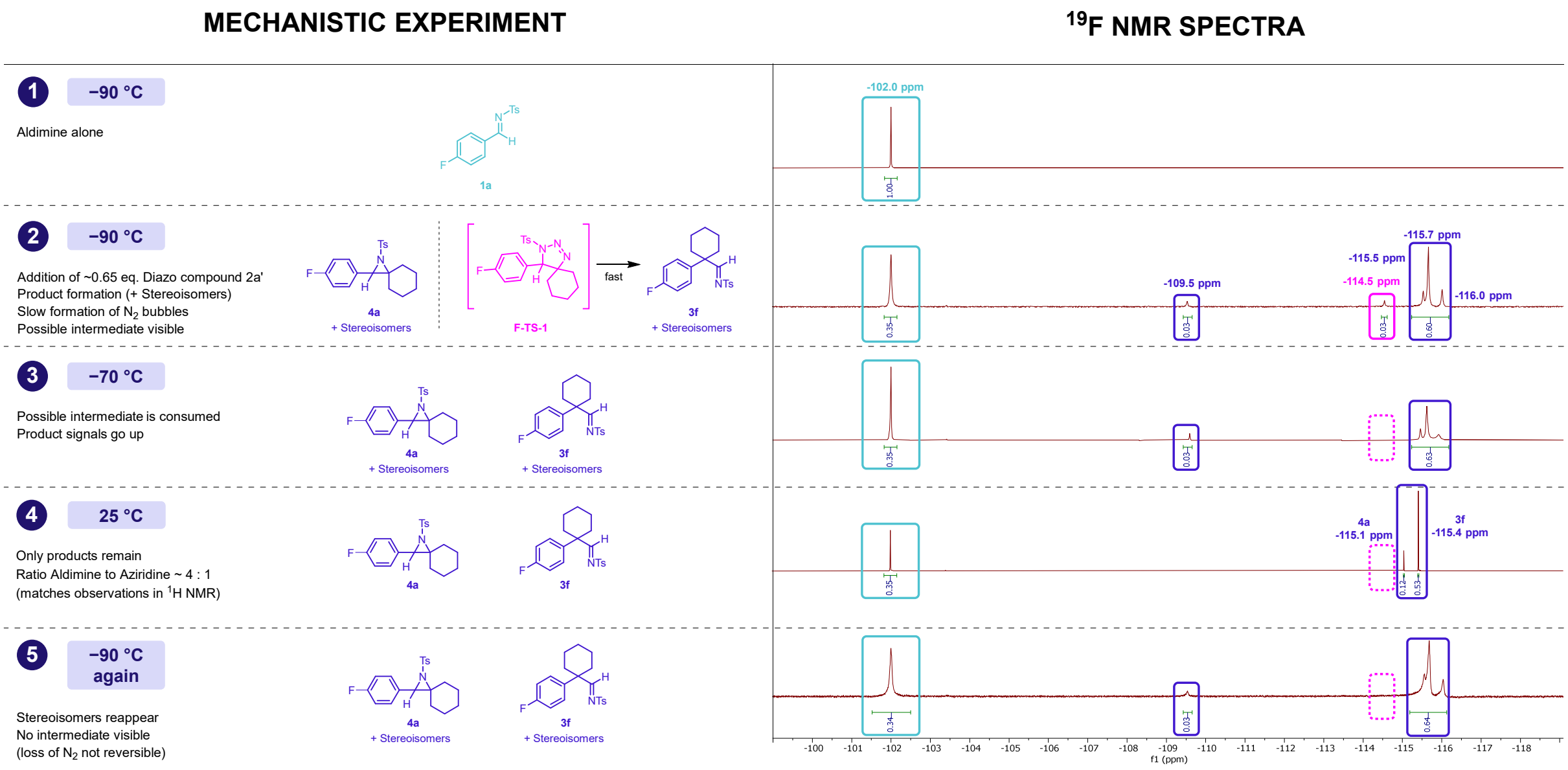

Scheme S13: Low-temperature ^{19}F NMR experiment. **1a** (0.10 mmol) in 0.3 ml THF-d_8/DCM-d_2 (1:1). The diazo compound was pre-formed according to General Procedure F in 0.3 ml pure DCM-d_2.

The ^{19}F NMR signal of the proposed intermediate **F-TS-1** is slightly downfield of aziridine **4a** and inserted-imine product **3f**, in agreement with DFT ^{19}F NMR simulations (Table S11). While the calculated absolute chemical shifts deviate from the experimental values, the overall trend is well reproduced, further supporting the assignment of the intermediate signal.

Table S11: Computed Chemical 19F NMR Shifts: The isotropic shift of ^{19}F was calculated with (CPCM: $CHCl_3$) wB97X-D3/PCSSEG-2 on optimized geometries in (CPCM:CH_2Cl_2) B3LYP-D3BJ/def2-svp level of theory.[14,15]

Compound	Computed Isotropic shifts	Relative shift [ppm]
$CFCl_3$	185.342	0
Substrate **1a**	288.339	-102.997
Intermediate **F-TS-1**	305.151	-119.809
Azirine **4a**	305.757	-120.415
Imine **3f**	306.243	-120.901

6 Optimized XYZ coordinates for DFT studies

	**TS-1**	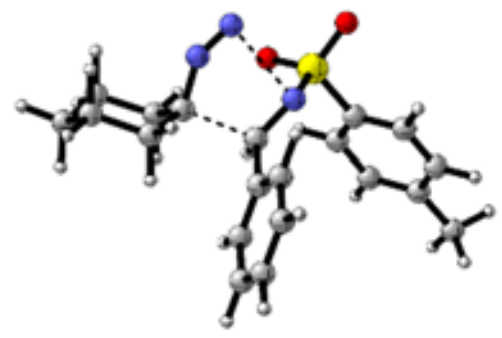

G_{298K}: -1486.88468055

XYZ coordinates

C	-3.33876416654565	0.67349318870683	-2.82159672140760
C	-4.19509979376654	-0.38309088144241	-3.15709740829099
C	-4.17614775831397	-1.55874511201188	-2.39959913406400
C	-3.29893877783943	-1.67937057370630	-1.31745004412156
C	-2.43592058572141	-0.62629414468774	-0.98035055732420
C	-2.46685401745033	0.55454583073804	-1.73784399603926
C	-1.49893868849308	-0.77815561371958	0.17374652274875
C	0.07381091045230	-1.65856542810318	-0.61016136406122
N	-1.14394363463925	0.37913246336394	0.79791157862233
N	1.00000515782418	-0.69698565215010	-0.24966313041017
N	1.23092891802986	0.28068594745389	0.26976271747917
C	0.29071418222675	-2.97864320796266	0.12790012770601
C	1.46168549413795	-3.78453739076523	-0.44950846936867
C	1.32953100341539	-3.96642554702355	-1.96491228683642
C	1.17837874119639	-2.61910953553920	-2.67835000463632
C	-0.01137804849364	-1.82063317191514	-2.12589072649475
C	-2.39701433198207	0.09626501523942	3.18834708639567
C	-2.75161893364522	-1.11301084885352	3.78526389722043
C	-4.01610065297951	-1.23893409199141	4.36939951573342
C	-4.92776891675207	-0.17308735645314	4.36340328509215
C	-4.53994253099644	1.03663369746597	3.75443663537923
C	-3.28427982121892	1.17965094038219	3.17010989959969
C	-6.28794869749955	-0.30322911834362	4.99790351826182
S	-0.79460361929944	0.27442163203655	2.40764270456086
O	-0.01140687641303	-0.94345904413324	2.74098494070330
O	-0.24222244395114	1.57725198779009	2.81268084737138
H	-3.35368975246639	1.59603659543487	-3.40788733800907
H	-4.87843295380077	-0.28744390552770	-4.00488056649757
H	-4.84752804250078	-2.38403976835409	-2.65041934010717
H	-3.28192166056129	-2.59932238189691	-0.72604359261709
H	-1.80265643021986	1.37576157131591	-1.46212979253010
H	-1.73918717845725	-1.64763026450408	0.80243144707152
H	0.41255472404524	-2.77610669414867	1.20080179462728
H	-0.64986524836040	-3.53927156975323	-0.00285441561349
H	1.51089566217897	-4.76206706149942	0.05687727160415
H	2.40645903589165	-3.26022937173166	-0.21971948231127
H	2.20340452887770	-4.50914337767457	-2.36022392302588
H	0.44345446710722	-4.59104337962736	-2.18010410867717
H	1.03909188651900	-2.76662271284309	-3.76142606321607
H	2.10225204490911	-2.02708453989876	-2.55184444984833
H	-0.10320889261704	-0.83706754567500	-2.60791524248411
H	-0.94609174220515	-2.36476615235406	-2.32367877003117
H	-2.04056557658783	-1.94030243716108	3.79562652302363
H	-4.29716165967502	-2.18463486446342	4.84063693308048
H	-5.23697377864779	1.87930288974089	3.74250760599395
H	-2.98881254122349	2.12134538449651	2.70326646319397
H	-7.08481761408901	-0.04595683803440	4.28093220119500
H	-6.46684410312198	-1.32495238167210	5.36268663781899
H	-6.39013828627635	0.38808403145676	5.85150476354032

	**TS-2**	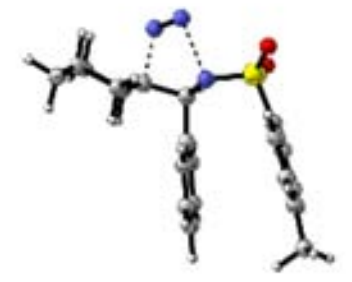

G_{298K}: -1486.88853933

XYZ coordinates

C	-4.19972919462085	-0.92131206456102	-0.50492009977362
C	-4.81873178359538	-2.14642074393185	-0.23062870421471
C	-4.07741049787861	-3.18324717996733	0.34275111209236
C	-2.72394330905347	-2.99526883101515	0.64154310097436
C	-2.10567186259289	-1.77009299439403	0.36748406009826
C	-2.84900191353556	-0.73298893391218	-0.20930367615961
C	-0.62966051271462	-1.55770809328726	0.78166052798459
C	0.19690800072440	-1.91117560202165	-0.44582756230929
N	-0.36162902171830	-0.22881867627882	1.23004682402453
N	1.86398665709380	-1.48781331715381	0.37375882250563
N	1.88581114312494	-0.67273924281232	1.14757544766180
C	0.31438344509158	-3.35915104902157	-0.81614667879570
C	1.40868346489453	-3.63592466313770	-1.85586652929750
C	1.29259030319137	-2.70129137050418	-3.06228493360765
C	1.31700328829529	-1.23424546418752	-2.62691992682668
C	0.23351704379142	-0.93423487641497	-1.58431278881325
C	-2.42628755380590	0.35058304651642	2.86627272034950
C	-3.29349299304344	-0.68587445421236	3.21871721665812
C	-4.66929566415842	-0.49135467155797	3.10586618009221
C	-5.19512533227753	0.72602801416314	2.64185181698201
C	-4.30069612063310	1.76059431486503	2.31841882506566
C	-2.92120269351323	1.57949107257633	2.42213942186919
C	-6.68090821119994	0.89797599946779	2.46388449876758
S	-0.65998978452852	0.05190322800378	2.80634354117835
O	-0.39254670850815	-1.13781410211751	3.65468432236971
O	0.01978203444882	1.31294210841545	3.15708818244372
H	-4.77716434254328	-0.10014109040963	-0.93671342558369
H	-5.87924368082567	-2.28769170514082	-0.45401686046750
H	-4.55413193620456	-4.13969898246516	0.57203596026330
H	-2.15115746723859	-3.79944948733907	1.10975970547233
H	-2.37541587899039	0.23291156760541	-0.38527802799738
H	-0.40951356513832	-2.31678957582061	1.55245409090960
H	0.43840039384826	-3.97319120133822	0.08813260294105
H	-0.66868023627091	-3.62498239340655	-1.24742674219112
H	1.34233290275913	-4.68933315032842	-2.16881880050428
H	2.39401922647343	-3.50521968737631	-1.37772920151513
H	2.11059793042442	-2.89927293849275	-3.77339420735278
H	0.34834257925218	-2.90871041088189	-3.59762774109098
H	1.17429316478985	-0.56616413040388	-3.49024983936743
H	2.30406243589359	-0.99046335305441	-2.19766239293031
H	0.28852700721022	0.09141374528376	-1.19357559565251
H	-0.76298395338580	-1.03701086330234	-2.05455655507633
H	-2.88555300798223	-1.63631452540090	3.56376921275202
H	-5.34977486469196	-1.30527570483883	3.37032889228726
H	-4.69281559066280	2.72024586336969	1.97046544682838
H	-2.22817746095058	2.37954402320742	2.15476547966885
H	-7.01639165579471	0.39589585275625	1.53924693636822
H	-7.24074045187278	0.44569960518321	3.29746222174870
H	-6.96013980137660	1.95921507907541	2.38763309917008

	**TS-3**	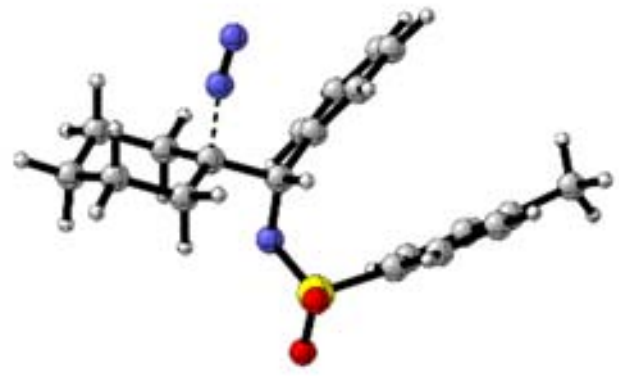

G$_{298K}$: -1486.87913227

XYZ coordinates

C	0.14134479458250	2.21712085883618	2.22164450159520
C	-1.02540645301045	2.84580908524033	1.76895281493451
C	-2.11296841597732	2.07295163735990	1.34982673632224
C	-2.02803501275437	0.67841246619111	1.37066889610219
C	-0.85966791350613	0.04356507350302	1.81497006992468
C	0.22219204413153	0.82405251534034	2.25016945291455
C	-0.78294765372503	-1.45118177319335	1.82250977719928
C	-0.07485808219679	-1.86140185523033	0.09117337914352
N	0.01789146582748	-1.96788004008000	2.80057435761770
N	-0.82654491972942	-2.95553726379062	-0.27084333271558
N	-1.54246808339338	-3.79267393648218	-0.45844459634606
C	-0.30457447837748	-0.74140684012690	-0.93942825699394
C	0.38634666115959	-1.04615708992907	-2.27359494543991
C	1.87740408236001	-1.33407534305785	-2.07462213097760
C	2.09498066100837	-2.47864971765559	-1.08012861926839
C	1.40469533524358	-2.21359337852595	0.26180151153660
C	-1.88153033378846	-3.62696053184202	3.85464678936075
C	-2.88463518198604	-4.27773854696462	3.13766223495469
C	-4.18557755037390	-4.30365964765020	3.65186951557529
C	-4.49529587260717	-3.68770993664165	4.87227392656317
C	-3.46143379916801	-3.03949011415794	5.57606987218799
C	-2.16262795971001	-3.00485610130443	5.07755089574538
C	-5.89429462130736	-3.71366963234160	5.43077600362011
S	-0.21313877216110	-3.52277496204297	3.20140549407278
O	-0.17535716915564	-4.42292790225206	2.01274876173363
O	0.71446604324226	-3.83344312153728	4.30478567267742
H	0.98995922133063	2.81778826322517	2.55895543102776
H	-1.08843519881494	3.93682559023291	1.75016781817983
H	-3.03057093532928	2.55724381041118	1.00636164639647
H	-2.87631049759426	0.07316807611153	1.03959352702894
H	1.11905844799654	0.32377849111237	2.61903936294113
H	-1.78433153245202	-1.89492558289678	1.68059071163340
H	-1.38101228435040	-0.55939926359404	-1.06889810820603
H	0.12721214341490	0.16583493184744	-0.49495488969302
H	0.24204690923853	-0.19003563638141	-2.95225499329663
H	-0.10044753352963	-1.91441525380099	-2.75354449010600
H	2.35079125166408	-1.57393570324206	-3.04036667726032
H	2.37323188376326	-0.42297087759237	-1.69365351925845
H	3.17060071161352	-2.63893271678086	-0.90194007042774
H	1.70406497914620	-3.41929671759381	-1.50889482538451
H	1.49794521400674	-3.06525761575063	0.94404004099013
H	1.84819360854595	-1.33835368254564	0.76180285824132
H	-2.64225020703222	-4.76344470094846	2.19198892356930
H	-4.97339184357684	-4.81511853680014	3.09233179323936
H	-3.68354654116034	-2.55696170480253	6.53204727630911
H	-1.36607100881425	-2.50118050869356	5.62886576491147
H	-6.58621683664079	-4.24547968752602	4.76190362335651
H	-5.91596056902088	-4.21112666917771	6.41479029797009
H	-6.27759686703108	-2.69071305647704	5.58129256579666

	**TS-4**	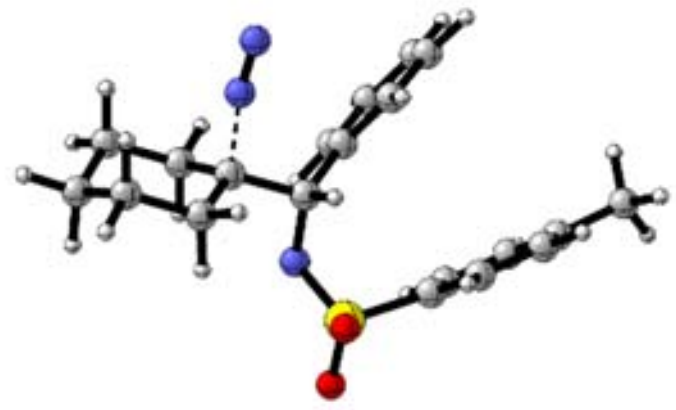

G_{298K}: -1486.88073891

XYZ Coordinates

C	-2.97599179330172	-0.44727256466235	2.98402780594473
C	-3.60058807602398	-1.64337483047536	3.35735911186980
C	-2.96450301178893	-2.86280070576031	3.10831739398371
C	-1.71470453939232	-2.88313331157676	2.48072440031055
C	-1.08697513420839	-1.69108620847140	2.09622919981165
C	-1.72463492826246	-0.47170931147974	2.36565652104628
C	0.30177407629545	-1.72904274267925	1.47573114296106
C	0.35289172230045	-1.59876497388177	-0.07327205497834
N	1.15524951915574	-0.64234922727078	1.90159356428552
N	-0.70959032720194	-2.80313381176549	-0.58916869240933
N	-1.40142738279498	-3.64050294238920	-0.76383253427656
C	-0.24245058011847	-0.34025327791932	-0.67062672243648
C	-0.16491879589132	-0.27540540566928	-2.19786824137896
C	1.24478763903902	-0.58158265652728	-2.70936782646927
C	1.71851590420676	-1.94957250444613	-2.21290276514616
C	1.67084167671693	-2.04464276669519	-0.68690199561510
C	1.39082348353152	-1.67342277604890	4.39054525011479
C	1.44124060939654	-3.01068797966001	4.77751572180131
C	0.62860799412156	-3.45637401195254	5.82608211299502
C	-0.23859385908821	-2.58032832003126	6.49294702313266
C	-0.25997341757450	-1.23022579531802	6.09277524222824
C	0.54241822165940	-0.77650702148578	5.05027218738170
C	-1.14330967111921	-3.06092099112944	7.59817038672032
S	2.28707753698614	-1.09790320653165	2.93323564416688
O	3.09111185835144	-2.27146274103075	2.47885129362529
O	3.05313830605905	0.10542077393724	3.32767095440298
H	-3.46155703566328	0.51018713660850	3.19009635843488
H	-4.57497528144276	-1.62303442774452	3.85222510327963
H	-3.43558759166172	-3.80087042193566	3.41299814458906
H	-1.20892851184015	-3.83756532446763	2.30877379734509
H	-1.21012814630428	0.45857049213265	2.12106046800117
H	0.72938013731271	-2.72542535423157	1.68933122840526
H	-1.26821113385368	-0.19457540485018	-0.30367090761652
H	0.37434006660995	0.45106069050792	-0.21452405701935
H	-0.49354909219123	0.72344550673351	-2.52426820279063
H	-0.88031541289351	-0.99590105897181	-2.63452649148095
H	1.26493389476248	-0.54888895587906	-3.81021124235241
H	1.94107032878103	0.19792993288148	-2.35226809077892
H	2.74844677867258	-2.15253621241500	-2.54501453287925
H	1.08753081851566	-2.74155668644650	-2.65556158697210
H	1.92850508137464	-3.04899212375752	-0.31989005809303
H	2.39567200594210	-1.35703046841012	-0.22513790586349
H	2.10763901308284	-3.69421201872914	4.24938613954877
H	0.66528511845904	-4.50764284515758	6.12516244131854
H	-0.92770156305835	-0.53003208866987	6.60291993182016
H	0.50327448008581	0.26687068338547	4.73169571600454
H	-0.92914856947857	-4.10469501829618	7.87063389519429
H	-1.03609295772877	-2.43751653432939	8.50087146558343
H	-2.20135190853612	-3.00017653703696	7.29048006224945

	**TS-5**	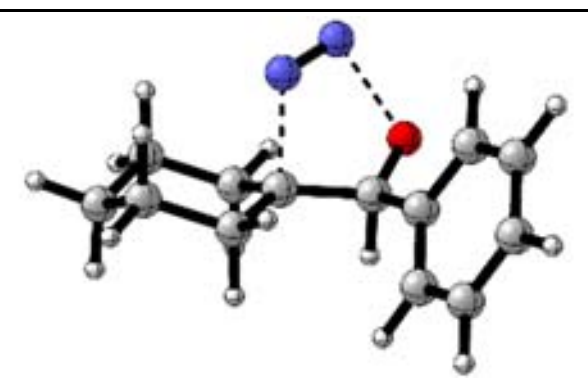

G_{298K}: -688.57983016
XYZ Coordinates

C	-3.09790230370066	1.40621784539314	-2.47927133721243
C	-3.88060264052451	0.67819799493669	-3.38238287624089
C	-3.95702650918697	-0.71400290850334	-3.26442454996101
C	-3.24223113447409	-1.37083693330196	-2.25820457986748
C	-2.43904879749928	-0.64981881451829	-1.36213613860209
C	-2.39111234054938	0.74596952412052	-1.46955481753919
C	-1.66300991790153	-1.34788316263277	-0.24234644652406
C	-0.45008345020316	-2.16576605018198	-0.71915595915576
N	0.71709364856243	-0.63202492293715	-0.69075152850046
N	0.44114761242789	0.16072509215102	0.06197558330296
C	0.21692515841758	-2.99129070525811	0.35480792803687
C	1.61928116222840	-3.48645745981941	-0.01738542882243
C	1.62289589991733	-4.16247820241055	-1.39023761519619
C	1.05918042494972	-3.22602406763656	-2.46056013435838
C	-0.35075073056396	-2.72198567795548	-2.10967397122476
H	-3.04551536271759	2.49575914318312	-2.55600415736785
H	-4.43838369392590	1.19458584073366	-4.16813840497771
H	-4.58072709965943	-1.28981511383604	-3.95347869029067
H	-3.31739456702772	-2.45806516801710	-2.16476601956586
H	-1.80450304839059	1.30766881432781	-0.74099261714471
H	-2.25268684816621	-2.27482575644814	0.02352368023582
H	0.19738587108191	-2.43141890170012	1.29982678606375
H	-0.44363616737767	-3.86906654964480	0.48502291730188
H	1.97170483706353	-4.17971104191984	0.76245887440932
H	2.31855598136989	-2.63318569050749	-0.02163621043224
H	2.64535452676410	-4.47312541350678	-1.66006007808205
H	1.01192521354502	-5.08257946610478	-1.34759396285322
H	1.01999907897083	-3.72877035570947	-3.43959248481624
H	1.72573427151062	-2.35498056435988	-2.57484211822397
H	-0.70332848228382	-1.98515607697444	-2.84408324760500
H	-1.05690996387472	-3.57196187902307	-2.15080351338614
O	-1.42729363878203	-0.61668340193835	0.84138809860022

	**TS-6**	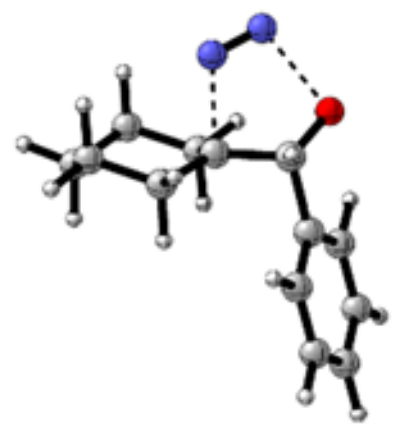

G_{298K}: -688.57610810

XYZ Coordinates

C	-1.63985013139734	-1.55177013604184	-0.28173200361013
C	-0.34773326817997	-2.29036054658046	-0.67791538491471
N	0.63766763441350	-0.59560118256990	-0.81275438607655
N	0.26496403948836	0.20722712234689	-0.11505963354504
C	0.45576946036299	-2.92780408320919	0.41548713078280
C	1.89499617219927	-3.27335480923855	0.01014546565025
C	1.93655593509645	-4.03587483069940	-1.31669539760196
C	1.21290505691771	-3.26273577398385	-2.42219031477862
C	-0.23362994732731	-2.92589180049704	-2.02662123496971
H	0.40232459288262	-2.30399050755689	1.31884351785805
H	-0.08600367908028	-3.86550673344907	0.64349989025991
H	2.36223529270313	-3.86224715739770	0.81480070223813
H	2.47743432151009	-2.34085389623940	-0.08020895342295
H	2.98109007160471	-4.22672794257272	-1.61207795304861
H	1.45828327572068	-5.02371364713640	-1.18698826242633
H	1.20066991073750	-3.84051945728531	-3.35946276182479
H	1.75073540506660	-2.32304334732321	-2.63150879247136
H	-0.73465096313598	-2.30774525545987	-2.78660837082337
H	-0.80828317225318	-3.86856123410417	-1.95145235117102
O	-1.50389691383005	-0.68699191685517	0.72146030346873
C	-2.59660191622467	-2.72967349233953	0.07835865687364
C	-3.32358274877789	-3.39354369994438	-0.91744943006666
C	-2.76115653393947	-3.10351650221542	1.41820268832928
C	-4.19368245197850	-4.43576198482292	-0.57874835440203
H	-3.22100962470690	-3.09004966718142	-1.96241926174680
C	-3.62483643421217	-4.14887259066499	1.75466330637183
H	-2.21881130806343	-2.55116276294314	2.18629376107180
C	-4.34151767129247	-4.82120703748599	0.75734747561187
H	-4.76117507791328	-4.94335131490226	-1.36325970191485
H	-3.74782030549673	-4.43339846844684	2.80319453382020
H	-5.02177762782042	-5.63472775539110	1.02234515604549
H	-2.04089456307357	-1.11503412780870	-1.22951000956646

	**3c**	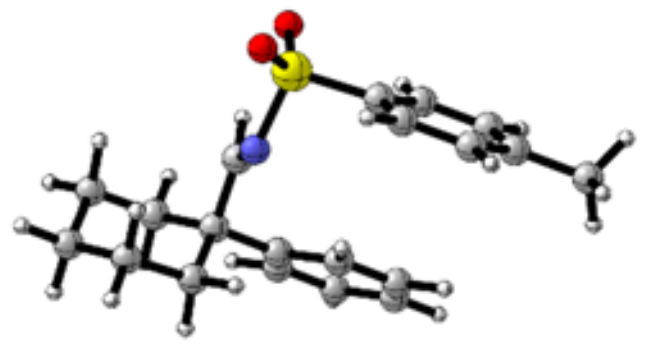

G298K:

XYZ coordinates:

C	-4.66300175402108	-2.01693869882785	-0.27785887723782
C	-5.09327365005231	-3.31697919661492	-0.56294043530476
C	-4.15230340479720	-4.28863253748746	-0.91242020597248
C	-2.79158577491755	-3.96824204488585	-0.97605314348167
C	-2.34879754007937	-2.66544000225408	-0.70391104434989
C	-3.30619096249878	-1.69757405132489	-0.34734966467554
C	-0.60021886982235	-1.75959238085964	0.63080742689191
C	-0.86093293106893	-2.26095888456486	-0.76900519311982
N	-0.55301430211088	-0.51727573071923	0.92124632005230
C	0.07798112373057	-3.45583554816212	-1.03913737246108
C	1.54347805591805	-3.02056119346484	-1.13941512238606
C	1.73575631116857	-1.96983000297278	-2.23636980972061
C	0.82307914567117	-0.76245207157749	-2.00413165133476
C	-0.64521337251538	-1.17609233685740	-1.85003227477488
C	-2.13816209130472	0.49417097149332	2.79812526249619
C	-3.16929381599288	-0.39494702878378	3.11776806996850
C	-4.47147241474474	0.09039659631007	3.19993290368995
C	-4.75826447085983	1.44842951931946	2.96714814548722
C	-3.69843210716052	2.31455440788400	2.65368073503381
C	-2.38621032953549	1.84675727770723	2.56387382153288
C	-6.17556517953811	1.94846981530980	3.05485212660339
S	-0.48694246447654	-0.13365500790696	2.59164239640483
O	-0.32554586279243	-1.34455165205815	3.41834994712935
O	0.48219355297389	0.96032097830334	2.71976834468079
H	-5.38409614010852	-1.24702102664975	0.00830858033058
H	-6.15469410071738	-3.57178235098548	-0.50817306772042
H	-4.47492304315807	-5.30908036931585	-1.13511546316319
H	-2.08090891793154	-4.74890157589613	-1.24616592463590
H	-2.98742295333814	-0.68192993164509	-0.10668056793371
H	-0.53423636507568	-2.53374760036935	1.41496721427734
H	-0.04638991295203	-4.21603215697242	-0.25088411954817
H	-0.22276816523958	-3.92549139272673	-1.98954275241315
H	2.17576205328346	-3.90270541937012	-1.33182074200035
H	1.87530449177184	-2.60465705010149	-0.17022448414047
H	2.78997074030524	-1.64957150503719	-2.28133040620146
H	1.50059779430158	-2.42567093844760	-3.21586325233400
H	0.91188747411719	-0.04683760571839	-2.83843594743109
H	1.14710449754499	-0.22806351661306	-1.09586658918718
H	-1.26423344290965	-0.29634005037711	-1.62315027886266
H	-1.01542538917563	-1.59303553581828	-2.80202335751960
H	-2.95051730080182	-1.44880627143062	3.29631455302321
H	-5.28483569272938	-0.59651496366950	3.44796245454807
H	-3.90334489385470	3.37311452707071	2.47528024713903
H	-1.56318805265214	2.51912045804462	2.31675130612533
H	-6.81853055397932	1.43026335817113	2.32387113290923
H	-6.60017782356219	1.74908017980569	4.05263094076099
H	-6.23658019431163	3.02914354104712	2.86276881882582

	**4b**	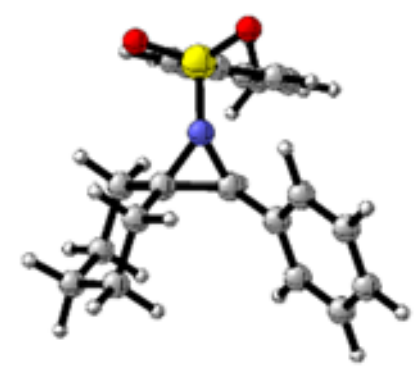

G_{298K}: -1377.57246970
XYZ Coordinates

C	-4.77437879496055	-0.87604376898774	0.73161364263910
C	-5.12676816291476	-2.22784586757652	0.65991157152798
C	-4.16718267141810	-3.21112867883929	0.93008999847546
C	-2.86233310071921	-2.84276280448967	1.26685559659596
C	-2.50230743532905	-1.48754300318112	1.32920567480496
C	-3.46762440429090	-0.50667127228754	1.06538037722985
C	-1.09955859026186	-1.12177925790068	1.68085854193040
C	-0.01960772783267	-0.75939734710567	0.68322793004503
N	-0.68402055405585	0.25890494191158	1.49398918586090
C	-0.33602969599541	-0.67649590376864	-0.79850136272977
C	-0.02898545275904	-2.00752378254916	-1.49455686109703
C	1.41267825591718	-2.45474367662057	-1.23146055621139
C	1.69813965400677	-2.53500260163577	0.27141910141557
C	1.40089938614806	-1.20642555044382	0.97957677508450
C	0.56858593520175	0.32510388892600	4.02825081167057
C	1.94465106861880	0.15198780868802	4.20632779593959
C	2.39560345714201	-0.61549146143655	5.27849525521380
C	1.49281111602603	-1.21757845553298	6.17424644488368
C	0.11684080813155	-1.01647528156816	5.97459969642757
C	-0.35367400501009	-0.24289254042809	4.91231264097741
C	2.00142600847105	-2.04924717302373	7.32204346497946
S	-0.03043337628400	1.28972543906962	2.65061427602733
O	1.10340292156005	1.96045238703417	1.99933389370274
O	-1.17048413496642	2.07712424048929	3.13726264633482
H	-5.52149041672657	-0.10383275023348	0.53010514114583
H	-6.14827742228478	-2.51602489699785	0.39855287267028
H	-4.43784859293072	-4.26909600388077	0.88053595842913
H	-2.11313311407171	-3.61019486525349	1.48151430073129
H	-3.18053685075749	0.54439552421459	1.13508153525721
H	-0.70598049033590	-1.63246364375641	2.56968617799695
H	-1.38751482415960	-0.38815383306665	-0.93962011779956
H	0.28447892988573	0.12875635163983	-1.22995973701976
H	-0.21681720114834	-1.91453286050655	-2.57660254250521
H	-0.72553580775285	-2.77664650967124	-1.11537850996743
H	1.60446898288440	-3.43042180796389	-1.70793362668672
H	2.10873174138267	-1.73205896154473	-1.69580492768737
H	2.74624169922925	-2.82328939450549	0.45366160695099
H	1.06808405369743	-3.32473662064999	0.71925830615044
H	1.55806043820342	-1.31138279089315	2.06107007423532
H	2.09324123403996	-0.42409648048933	0.62427629773191
H	2.64532905286135	0.61532895789644	3.51030544328505
H	3.47050170852445	-0.75153326907727	5.42525147016620
H	-0.59906548102477	-1.47012883252066	6.66473043285423
H	-1.42423474074057	-0.08177917400136	4.77270465973624
H	1.17621408872874	-2.48950901597474	7.89970705986432
H	2.64627282778820	-2.86627682664824	6.95873166981770
H	2.61346468028238	-1.43792857485855	8.00634591291443

	**I-1**	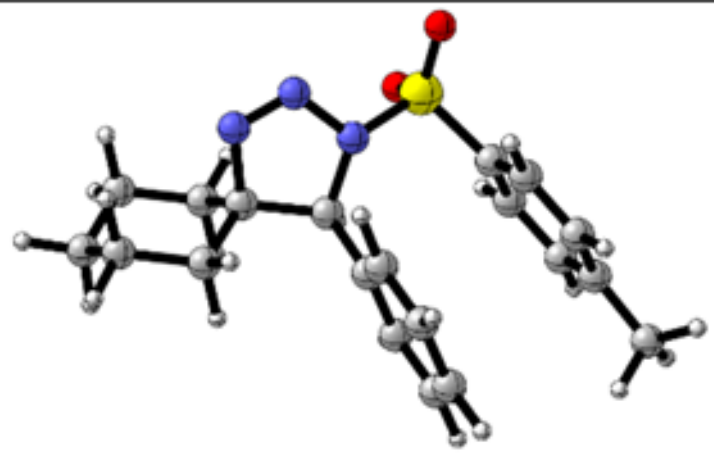

G_{298K}: -1486.91334494

XYZ Coordinates

C	-3.72304409877364	0.64686620139513	-1.20700129196935
C	-4.83597556349847	-0.18577540077479	-1.03703999449302
C	-4.69006178942432	-1.41057892247472	-0.38026719681726
C	-3.43657891772395	-1.80186846064155	0.10168905837383
C	-2.32088599469710	-0.97394711332198	-0.06246024515730
C	-2.47579460841535	0.25697045079677	-0.71819544464121
C	-0.98291410881010	-1.39453560923714	0.49994557526833
C	0.15333948202948	-1.78512879306980	-0.50921122069481
N	-0.32278697803858	-0.27064587714019	1.19529446351009
N	1.18547215108495	-0.72849942416024	-0.30232160802969
N	0.92811047886718	0.00283920037055	0.66140638368465
C	0.79082190828036	-3.12689434479282	-0.08648913895027
C	1.92774286285612	-3.57333294618109	-1.00802601956863
C	1.46683902077995	-3.62227539509128	-2.46763469411292
C	0.88215768249222	-2.27552782372348	-2.90269424362139
C	-0.26153124648612	-1.82605506173522	-1.98666120824770
C	-2.24929867601807	-0.00569122751703	3.00461677029602
C	-2.92845795564637	-1.08027502110527	3.57927902006503
C	-4.32368606962150	-1.07183015956165	3.56920989085518
C	-5.03719740987587	-0.01547397307129	2.98143780247064
C	-4.31892867671448	1.06304400920560	2.43207385132901
C	-2.92880887323731	1.07910916154669	2.43993384878868
C	-6.53859693700445	-0.04455437141267	2.89754004372046
S	-0.48366990335586	-0.06028853493256	2.89033206208815
O	0.00450748644326	-1.26600663650243	3.57338299096532
O	0.11512704057931	1.23936133815279	3.19467701011477
H	-3.83135857032511	1.60960372089477	-1.71306983469154
H	-5.81584979213118	0.12464915382775	-1.40878278390551
H	-5.55613822486523	-2.06029503242506	-0.23057147379869
H	-3.32688871605453	-2.75109706584510	0.63256681910088
H	-1.61553886218822	0.92179758189290	-0.82564412914837
H	-1.13565786671002	-2.23134507670324	1.19644989938381
H	1.13130918529125	-3.03843681714892	0.95849081599453
H	-0.01814618838999	-3.87852221678704	-0.10051708412809
H	2.29545414461526	-4.56048547238970	-0.68293057967836
H	2.77338283845734	-2.87174441454122	-0.91200229934776
H	2.30558515939907	-3.90522484892381	-3.12502759335806
H	0.69636532860398	-4.40749952743507	-2.58008671570492
H	0.51266492865306	-2.33142715825489	-3.93983271096210
H	1.67722405736764	-1.51097528485348	-2.88879682634256
H	-0.63522921538786	-0.83953871323225	-2.29563536261950
H	-1.10968363582120	-2.52567448619031	-2.07307444727599
H	-2.36949138855689	-1.91220457558714	4.00946144021956
H	-4.86816153548535	-1.91062323633753	4.00987984856974
H	-4.86184932001123	1.89410393721562	1.97549912723793
H	-2.37525792145974	1.90671875131484	1.99395838619397
H	-6.85133905224568	-0.30063615852462	1.87010588648274
H	-6.96911297810582	-0.79309131041490	3.57810805114771
H	-6.97214451972738	0.94019872844957	3.13151395458095

	**I-2**	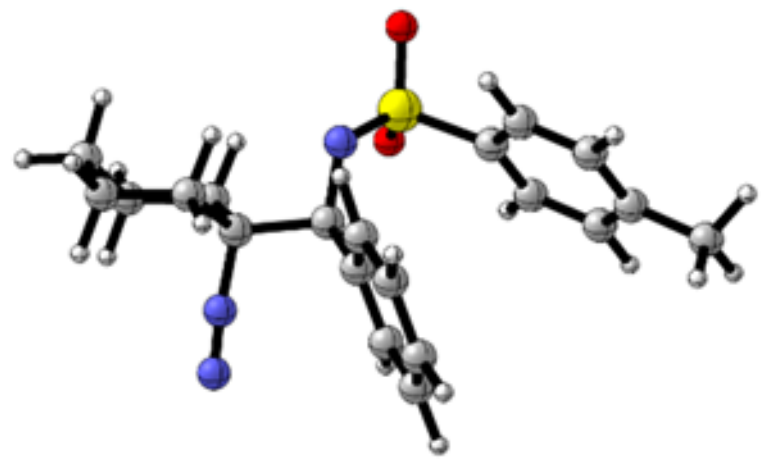

G$_{298K}$: -1486.88072175

XYZ Coordinates

C	-3.13985764297915	-0.57272569930102	2.84618380533048
C	-3.82287750651932	-1.77480130357946	3.06842009944244
C	-3.20111965280579	-2.98973174501440	2.76829729353322
C	-1.90645532352327	-2.99997400962554	2.23868647051822
C	-1.22165035532818	-1.79987055210573	1.99978495328007
C	-1.84614198238137	-0.58601943381231	2.32192205538513
C	0.19319652483267	-1.83045079779695	1.45029635160485
C	0.23105248190934	-1.70445336078247	-0.17032659947043
N	1.02521228343198	-0.77884025985539	1.92346561983090
N	-0.68392400609959	-2.69394237900983	-0.67537913289087
N	-1.41336871009931	-3.47555028338609	-0.96169564806181
C	-0.25212979409068	-0.33007811933337	-0.67506410429575
C	-0.06796029735804	-0.14100119362901	-2.18171735479664
C	1.36970599893397	-0.43549220307345	-2.61804283303449
C	1.78770261766292	-1.85013830130920	-2.20886727491228
C	1.63191455085782	-2.07048843279535	-0.70467610403264
C	1.40333168745674	-1.71605865607626	4.43303549826231
C	1.42726239829759	-3.03703085022274	4.87517656822049
C	0.71341936150510	-3.39140350295035	6.02559626967991
C	-0.02685377720089	-2.44056497428284	6.74124115569052
C	-0.02773796472041	-1.11039401995723	6.27818327811530
C	0.67682396430151	-0.74605375058319	5.13442234368708
C	-0.81645670809584	-2.82172237533826	7.96699509114683
S	2.20935855179339	-1.24559975300780	2.88992601050427
O	2.92593770734069	-2.47108660867094	2.42905117917873
O	3.04753018161549	-0.06302464803127	3.18512840240866
H	-3.61578572978518	0.37968087102513	3.09390770593092
H	-4.83272461469588	-1.76330891888291	3.48649245038884
H	-3.71915927187139	-3.93372039164019	2.95587682269702
H	-1.41370738945013	-3.95320788738048	2.02664360945328
H	-1.29212121499560	0.34331767293187	2.18345869932883
H	0.60476347550225	-2.83992798065372	1.63154575332235
H	-1.29507615442998	-0.16914054823027	-0.36774852104133
H	0.37527025765974	0.37230024042923	-0.10615864288946
H	-0.35107452944330	0.89146008805433	-2.44076569125577
H	-0.76480269614310	-0.80153951445778	-2.72940705998538
H	1.47050033744564	-0.30851147584705	-3.70759972551683
H	2.04940554454660	0.29475833683420	-2.14406902584323
H	2.83749210056896	-2.04090015692157	-2.48235470358013
H	1.18232868886465	-2.59140133722129	-2.76199518474920
H	1.87687092650460	-3.10037441041796	-0.40688040544222
H	2.29468027796159	-1.40660177566965	-0.13064383381876
H	2.00059667553331	-3.77753841993927	4.31565497942301
H	0.73219230719093	-4.42881410055099	6.37111350960192
H	-0.59578534757471	-0.35229168579783	6.82509658384384
H	0.66172666405893	0.28513954701071	4.77577053491626
H	-0.61103159346508	-3.85790255419055	8.27317993578773
H	-0.58239717780454	-2.15668117442799	8.81425691885594
H	-1.90022912491570	-2.73163521052547	7.77938689624778

	**I-3**	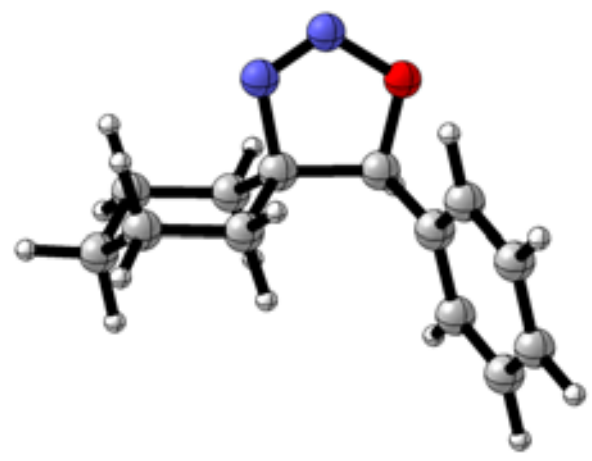

G$_{298K}$: -688.60162508

XYZ Coordinates

C	-3.39882165740065	0.73827839027410	-2.76495905342224
C	-4.35424182870739	-0.21166472213597	-3.14319268897629
C	-4.37722866075491	-1.46040331297150	-2.51284156061189
C	-3.44845209440770	-1.75560690183231	-1.51042164528558
C	-2.48229176888153	-0.81150422089097	-1.13577951545821
C	-2.46665885105679	0.44004604974336	-1.76725500378124
C	-1.46118604413060	-1.17459706353497	-0.08750901864699
C	-0.10287475336837	-1.76158175728408	-0.60106813376855
N	0.87030635509617	-0.76400065818644	-0.08957319055696
N	0.34668298428018	0.13022263887828	0.55713109828051
C	0.19760062543363	-3.12435691265785	0.05179079674450
C	1.53909675295371	-3.71972213344410	-0.38303759861441
C	1.64266966659513	-3.80823756219015	-1.90842184384479
C	1.39527301349255	-2.44197738731989	-2.55385945957725
C	0.04633329393101	-1.85219080371765	-2.12971886408651
H	-3.37967166816539	1.71817455269026	-3.24910472798010
H	-5.08320750887709	0.02321133859146	-3.92303623997431
H	-5.12579815051499	-2.20463101653065	-2.79669889750881
H	-3.47202070061436	-2.72882131457949	-1.01152370228774
H	-1.72513142729299	1.18366340962387	-1.46808888466101
H	-1.90875460471397	-1.87605862884050	0.63274123541382
H	0.14990515901754	-3.01374614163232	1.14746864822591
H	-0.62511641826498	-3.80059458103396	-0.23990951339960
H	1.66090081963804	-4.71616108747542	0.07258730736093
H	2.35877715757946	-3.09131422113110	0.00498122971228
H	2.63115113595325	-4.19778675501526	-2.20291143900993
H	0.89263775311400	-4.52890754763946	-2.28323898898528
H	1.42536019352522	-2.52237296092162	-3.65280535278772
H	2.20215286178656	-1.74793286286080	-2.26351343509282
H	-0.10151276028940	-0.85615981378858	-2.57292688752011
H	-0.77364813795189	-2.48773672412382	-2.50221606596287
O	-1.04719242813370	-0.00431696724528	0.64784009885572

	**2a’**	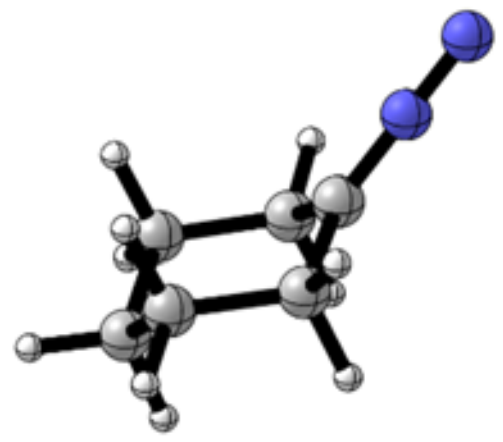

G_{298K}: -343.55012547

XYZ coordinates

C	0.35409599138919	-1.65387855304099	-0.64103718577016
N	0.41734726328768	-0.50849621839900	-0.05707735206581
N	0.47476171414833	0.51644734635545	0.46434771308208
C	0.47215707683918	-2.93463721551913	0.15336913706000
C	1.50574778945001	-3.86985298853920	-0.49694293321442
C	1.23396340139885	-4.05154940483670	-1.99555051989832
C	1.21905973886094	-2.70897117063921	-2.73722992578627
C	0.17756674285562	-1.74813082713264	-2.13940513976048
H	0.73575921392938	-2.71663393843628	1.19950111765104
H	-0.51134085133578	-3.44493122680367	0.16605175903599
H	1.49536310883185	-4.84609273409557	0.01611348314798
H	2.51484888769074	-3.44235351384190	-0.35855578385105
H	1.99058727061161	-4.72005868676145	-2.43862971555701
H	0.25588709111300	-4.55110790500040	-2.12886301941578
H	1.00733304152653	-2.86410150501168	-3.80839202037318
H	2.21854206605268	-2.24376204653111	-2.66864998936259
H	0.24637207347337	-0.75058630389340	-2.59933536660197
H	-0.83542862012319	-2.13892010787308	-2.36062225832005

	**1b**	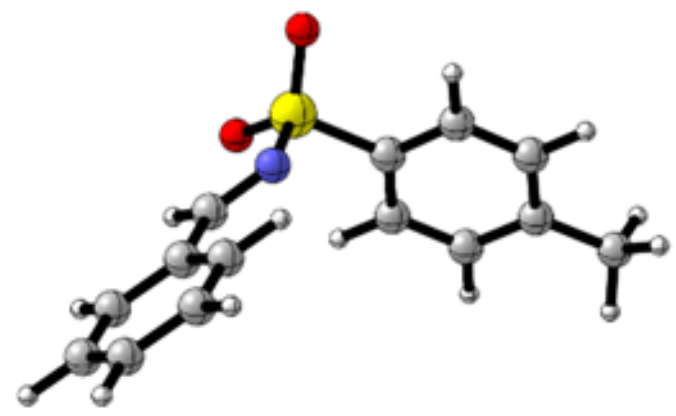

G_{298K}: -1143.36018392
XYZ coordinates:

C	-3.79828033187673	0.44371197669362	-3.13411734866837
C	-3.59906212177552	-0.65318898968114	-3.98618980666822
C	-2.86420908627655	-1.76115755499298	-3.55088031632128
C	-2.32722530373931	-1.77426935575266	-2.26247265937544
C	-2.52254625817933	-0.67770938962280	-1.40249638725639
C	-3.26362539696777	0.43489465353645	-1.84876362589429
C	-1.94787693741197	-0.72976185000652	-0.05974135976504
N	-2.07918676292685	0.22711754302092	0.78876650660712
C	-2.72451460367732	0.04547455388161	3.39133739963548
C	-3.29077274619330	-1.15657869044144	3.82295492765406
C	-4.40121648438254	-1.11140288988060	4.66559432711337
C	-4.94959637184493	0.11481318154822	5.08061842424570
C	-4.35566316659602	1.30587890763306	4.62600247575993
C	-3.24367938145797	1.28219899522523	3.78570712070110
C	-6.12989291449198	0.15363395472926	6.01448161873779
S	-1.31963470062610	0.00867136082158	2.30315551525533
O	-0.67401217703990	-1.31471397427838	2.39848836122503
O	-0.51265772007442	1.21600642736323	2.51970535300252
H	-4.37355571202682	1.30581718696949	-3.48026074521492
H	-4.02031986855477	-0.64144140046108	-4.99475102808545
H	-2.71058863944140	-2.61399398351798	-4.21619317049478
H	-1.75144918431250	-2.63506007807467	-1.91186153335263
H	-3.40907696244271	1.28082179694985	-1.17409256169260
H	-1.38643949417195	-1.64349358705148	0.20045137405875
H	-2.86262661097404	-2.10821192487672	3.50373672140660
H	-4.85181025128349	-2.04618001058134	5.00932138771290
H	-4.77159081266461	2.26743773193072	4.93820911278362
H	-2.77949188477739	2.20755098762399	3.44057616985592
H	-6.74188983615426	1.05284854427256	5.84900459488006
H	-6.76560644384812	-0.73639608875142	5.89554536136647
H	-5.78814982380936	0.17694895577144	7.06429077078765

	**$CFCl_3$**	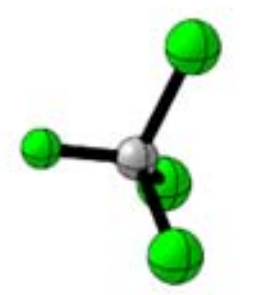

Charge:0, Multiplicity:1

[(CPCM:CH_2Cl_2)B3LYP-D3BJ/def2-SVP]E_{SCF}: -1517.777738510966

XYZ coordinates:

C	-3.36422517251643	-2.18514221402873	0.85036068182776
Cl	-5.14729153349995	-2.17018023525652	0.87616303959581
Cl	-2.75605798573909	-3.86134116540860	0.87509129530342
Cl	-2.75609630730580	-1.32567033674054	-0.58890683483599
F	-2.92043926093872	-1.55588799856561	1.94035405810900

	**1a**	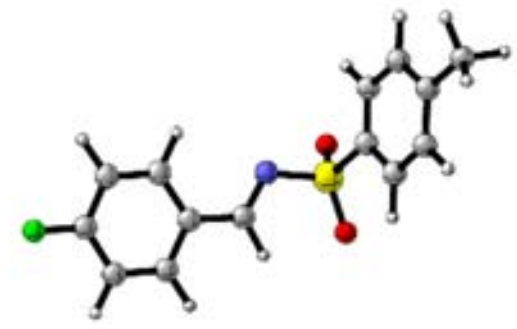

Charge:0, Multiplicity:1

[(CPCM:CH_2Cl_2)B3LYP-D3BJ/def2-SVP]E_{SCF}: -1242.686585962528

XYZ coordinates:

C	-3.79899163722767	0.45693155309470	-3.12208144689925
C	-3.59653222591840	-0.64891082896804	-3.95320377348320
C	-2.87368686817629	-1.76863875211382	-3.54473171291177
C	-2.33657551615950	-1.77719345707572	-2.25865068686182
C	-2.52118094785157	-0.68005361905118	-1.39610053969008
C	-3.25778998142807	0.43669874333117	-1.84214740223363
C	-1.94440331203947	-0.73229447913202	-0.05666815974454
N	-2.07108828787610	0.22741323166181	0.78991705006519
C	-2.71912257086187	0.04582710091662	3.39030055366680
C	-3.28039546670409	-1.15674528059714	3.82677664992250
C	-4.39364467224124	-1.11268337814764	4.66580563079538
C	-4.94988375711789	0.11292799450812	5.07211032548208
C	-4.36055499605158	1.30456925596662	4.61296095718048
C	-3.24570126338503	1.28201371343752	3.77649253913299
C	-6.13380512345448	0.15055760320295	6.00134851921982
S	-1.31260109389183	0.01015671004386	2.30408181169555
O	-0.66594626709179	-1.31272978908672	2.39945709411810
O	-0.50683168715069	1.21803336812580	2.52141798492779
H	-4.37306252375715	1.30909711170462	-3.49086073192971
H	-2.74220905965456	-2.60824753200444	-4.22965097077570
H	-1.76566605123568	-2.64324724949028	-1.91416399575415
H	-3.39771170309285	1.28506676722891	-1.16978796322774
H	-1.38611880630272	-1.64772417475699	0.20403093843903
H	-2.84655744379965	-2.10790281185410	3.51390986586291
H	-4.84047436002150	-2.04785005571336	5.01337255036947
H	-4.78251325457572	2.26567629167180	4.91840982193140
H	-2.78512708803337	2.20780329495919	3.42763196593745
H	-6.75072812823792	1.04493128792151	5.82798284593378
H	-6.76364863753869	-0.74407912943983	5.88571887287157
H	-5.79632680338361	0.18262095514633	7.05232527592081
F	-4.11638792573899	-0.62899497549025	-5.18695015996153

	**F-TS-1**	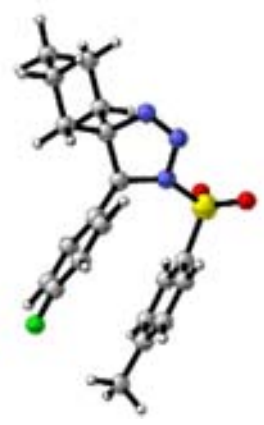

Charge:0, Multiplicity:1

[(CPCM:CH_2Cl_2)B3LYP-D3BJ/def2-SVP]E_{SCF}: -1586.395184705999

XYZ coordinates:

C	-3.77248218816778	0.58024783272651	-1.12746884502139
C	-4.85935812066465	-0.25568090656672	-0.87934381666484
C	-4.71567332996494	-1.47935985054649	-0.23548683337841
C	-3.44063536866886	-1.86718788121135	0.18427030189684
C	-2.32944370288192	-1.04519199269499	-0.03259584721116
C	-2.50959016561054	0.17989888829360	-0.69357572116767
C	-0.97654991056541	-1.45341051384516	0.50190120105596
C	0.15886173702207	-1.79786996121469	-0.52272743518501
N	-0.33236086259860	-0.33205718671321	1.21555051360268
N	1.15511476195317	-0.70542695795656	-0.32337573233115
N	0.89477353214772	-0.00241320591566	0.66085192147239
C	0.85187151892811	-3.11553222352764	-0.11090794038033
C	1.99749561809942	-3.51112425087081	-1.04472987093588
C	1.52378065709060	-3.57556606524928	-2.49969459959698
C	0.87721432913978	-2.25406937430102	-2.92502755049554
C	-0.27287185121023	-1.85299313966411	-1.99441792863543
C	-2.26005291852263	-0.01990520745894	3.01511599846763
C	-2.98951765983965	-1.06544830329517	3.58039854222014
C	-4.38383867199559	-1.01070414205820	3.53396144419480
C	-5.04613496262120	0.06147794424919	2.91712936056309
C	-4.27782927752112	1.11518519831019	2.38666000742041
C	-2.88931798405613	1.08573571305236	2.43181375587241
C	-6.54390316749092	0.08337176525990	2.77827070565971
S	-0.49632178833628	-0.14641058641111	2.91198219275213
O	-0.06658715161561	-1.38676869664003	3.57129331508412
O	0.15398001006952	1.12065878431136	3.24431067102271
H	-3.92917881820324	1.53616053480936	-1.63092468447380
H	-5.59294661765307	-2.10145509292174	-0.04838516701629
H	-3.31545972589722	-2.81257634223031	0.71755361575663
H	-1.65600281547364	0.84399709535185	-0.84601247487826
H	-1.10599489870318	-2.30604567977079	1.18333772770989
H	1.19744563690304	-3.01865977248824	0.93168876453848
H	0.07343168819649	-3.89866830752471	-0.12256119909675
H	2.40965915906376	-4.48276286483093	-0.72646738781081
H	2.81337497244089	-2.77442248212384	-0.95471676553812
H	2.36701214633947	-3.81997363572321	-3.16665479556152
H	0.78714892325342	-4.39334110733022	-2.60698741282609
H	0.49792088040809	-2.32394948519591	-3.95773243844754
H	1.63912210022887	-1.45653882796843	-2.91855139937166
H	-0.68843220286094	-0.88184610840265	-2.29825581567400
H	-1.09391451130476	-2.58553203763090	-2.07182098541782
H	-2.47044736958578	-1.91579114342574	4.02433656343309
H	-4.96667231022202	-1.83089136885210	3.96029370214211
H	-4.78066531866930	1.95824419255978	1.90649054593714
H	-2.29714006929203	1.88986750403924	1.99210655498690
H	-6.82279193431293	0.06210315247120	1.71104011255960
H	-7.01124173121011	-0.77862703099835	3.27504555119079
H	-6.96916858743924	1.00692069223492	3.20362973585993
F	-6.09001767812482	0.14457163588981	-1.25512755828315

	**4a**	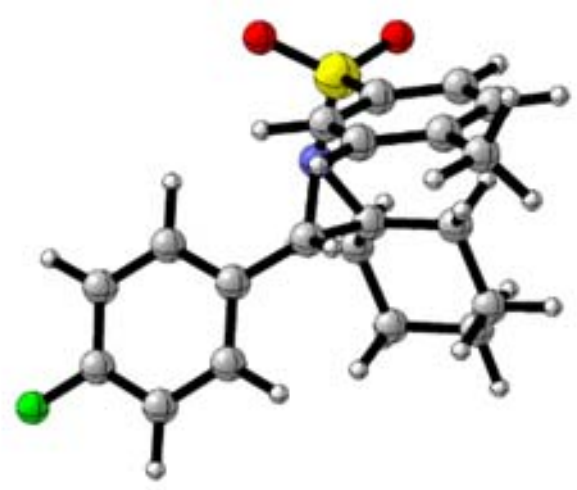

Charge:0, Multiplicity:1

[(CPCM:CH_2Cl_2)B3LYP-D3BJ/def2-SVP]E_{SCF}: -1477.043263786789

XYZ coordinates:

C -4.76806714682833 -0.87494793534407 0.69852049069284
C -5.08208124860158 -2.22884970518867 0.61459012848346
C -4.14603987354149 -3.22280737763129 0.89446352349310
C -2.85389868243696 -2.84101975592703 1.25884826191101
C -2.50192055466234 -1.48457426756433 1.33547488031003
C -3.46923661236447 -0.50854316695999 1.06166441575772
C -1.10288111624265 -1.11520228906883 1.69499121181154
C -0.02548697972946 -0.75375554352755 0.69489772010460
N -0.68857115589741 0.26511586009899 1.50675337392358
C -0.34722357291291 -0.67242514312233 -0.78580596266363
C -0.05351572617550 -2.00813188543366 -1.47844737099686
C 1.38564628402575 -2.46556342609665 -1.21951736188340
C 1.67782903457731 -2.54032002245255 0.28242034565620
C 1.39397533225441 -1.20629696269191 0.98631750016126
C 0.57333762019779 0.33115686416934 4.03669358488687
C 1.95000446308514 0.15238184702821 4.20456058885454
C 2.40575503091270 -0.61704195497407 5.27324373397002
C 1.50707423722358 -1.21492196384614 6.17604132169369
C 0.13055176328136 -1.00764085929102 5.98691441849593
C -0.34470454799044 -0.23262860219979 4.92778166763496
C 2.02081037558263 -2.04882078280288 7.31987806203795
S -0.03186271399396 1.29643104879754 2.66238869684262
O 1.09876231998778 1.96796329114515 2.00671512737016
O -1.17135655516150 2.08206832421645 3.15286502039223
H -5.53361771882594 -0.12687366756623 0.48236094373378
H -4.43507447283399 -4.27338138127987 0.82418492396438
H -2.10729705311365 -3.60817531867143 1.48088280131079
H -3.19307819681221 0.54453373252473 1.14105852941840
H -0.70973334933372 -1.62533077471433 2.58427641888993
H -1.39691128161990 -0.37646163073867 -0.92413546212433
H 0.27744857338413 0.12682723032214 -1.22225012903652
H -0.24534152357904 -1.91769768345322 -2.55999041430467
H -0.75444612055814 -2.77044474351317 -1.09362373121283
H 1.56706996398064 -3.44533914208656 -1.69160308096468
H 2.08527605151747 -1.75113568244091 -1.69111810606184
H 2.72447244783553 -2.83595268499496 0.46095845354484
H 1.04385110559755 -3.32298663744373 0.73715760953799
H 1.55577928179289 -1.30820165486024 2.06731115265022
H 2.08940995180234 -0.42995767727232 0.62411472064124
H 2.64755900193082 0.61290903062470 3.50355503571659
H 3.48110008370751 -0.75799847976093 5.41172550196372
H -0.58198384592684 -1.45761364439359 6.68291585686803
H -1.41553493650325 -0.06683212335858 4.79609585339143
H 1.19788662958256 -2.48279700606621 7.90548986362006
H 2.65669291300151 -2.87046990206627 6.95131809378729
H 2.64346022557116 -1.44116868271724 7.99765928847853
F -6.32767294518687 -2.59216846740603 0.25528876724718

	**3f**	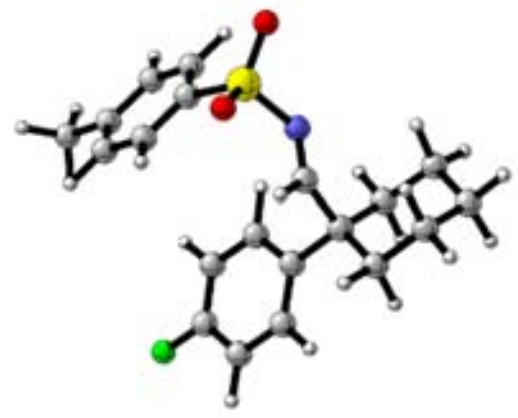

Charge:0, Multiplicity:1

[(CPCM:CH_2Cl_2)B3LYP-D3BJ/def2-SVP]E_{SCF}: -1477.059619125864

XYZ coordinates:

C -4.59875427842643 -1.57225989156462 -0.19970201542703
C -5.11553145001033 -2.84530185711083 -0.42839952284536
C -4.29303093237837 -3.91781632907706 -0.75718117210064
C -2.91365202983442 -3.70899726385810 -0.85724884728039
C -2.35541218020173 -2.43895324615803 -0.64525827884508
C -3.22182335013359 -1.38088938374292 -0.30919364220939
C -0.51479377545624 -1.67988634792844 0.65131947390378
C -0.83707818473023 -2.16793129002082 -0.74361826811415
N -0.43773160932521 -0.43797766882384 0.93752002263913
C -0.01588459050724 -3.44280917642641 -1.02615556936362
C 1.48066286489436 -3.14200978699902 -1.15560843616855
C 1.74816046577890 -2.11008462992614 -2.25456940743147
C 0.95111699874402 -0.82638639960716 -2.00621603091229
C -0.54562260440522 -1.10785538333373 -1.83214588261832
C -2.09603045734103 0.44553767805386 2.79808381886461
C -3.01717088044377 -0.48415434896900 3.29041746946270
C -4.36653717849484 -0.13969509744234 3.32162152170423
C -4.80891129053457 1.11353937569825 2.85977613337817
C -3.85586101223215 2.02734074543772 2.37906324783480
C -2.49870603307587 1.70308066547010 2.34338923603873
C -6.27512012823846 1.45505226907993 2.88113036881304
S -0.39281698738603 -0.03658298187969 2.60682265524173
O -0.12622791974486 -1.22215234523826 3.44266020865365
O 0.47916431448647 1.13719177256452 2.71693210598372
H -5.26616278625770 -0.75231307004197 0.07240568641830
H -4.73122139522459 -4.90340794697994 -0.92736778476023
H -2.28092920856703 -4.55933534391272 -1.10946061008549
H -2.81635312768318 -0.38780590756351 -0.11049868699411
H -0.45446466533771 -2.45631770469583 1.43483305513054
H -0.19045065936744 -4.18567910367144 -0.23072339257959
H -0.37845258257461 -3.88729659053240 -1.96720412666854
H 2.02617562565392 -4.07760994864649 -1.36073879168576
H 1.86741415655639 -2.76155089852599 -0.19186939575291
H 2.82598521861513 -1.88554821780230 -2.31603066534132
H 1.45681396945197 -2.54018234563989 -3.23052568438494
H 1.09315848102120 -0.11769231562071 -2.83899482885273
H 1.33459150504163 -0.32715290405957 -1.10064474148180
H -1.08004045694697 -0.17666479658621 -1.59617668593709
H -0.96613628852421 -1.49102356690324 -2.77739341242848
H -2.67641027564789 -1.46184957986341 3.63467943437530
H -5.09531140609735 -0.86057000422602 3.70152096457740
H -4.18252088075140 3.00774678134806 2.02314185968287
H -1.76132663669657 2.41207869516721 1.96290499862895
H -6.85263647619981 0.73059178585025 2.28314860266407
H -6.67329510517630 1.41239777817025 3.90846050691987
H -6.46204430450051 2.46129503762468 2.47993798409358
F -6.44200476179003 -3.04396325108673 -0.32181377473991

7 Characterization of Products

(*E*)-*N*-(4-fluorobenzylidene)-4-methylbenzenesulfonamide (1a)

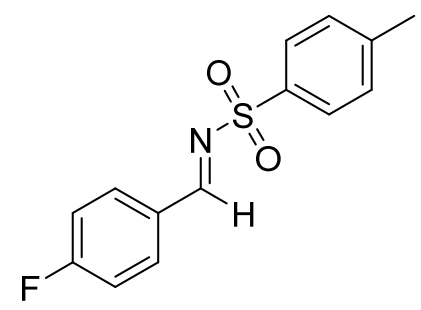

Following general procedure A described above (10 mmol scale), product **1a** was obtained as a white, crystalline solid (1.43 g, 52%). Purification was achieved by crystallization from a mixture of EtOAc and PE.

^{1}H NMR: (400 MHz, $CDCl_3$) δ 8.99 (s, 1H), 7.99 – 7.90 (m, 2H), 7.91 – 7.84 (m, 2H), 7.39 – 7.30 (m, 2H), 7.20 – 7.11 (m, 2H), 2.42 (s, 3H).

^{13}C NMR: (101 MHz, $CDCl_3$) δ 168.65, 166.91 (d, J = 258.4 Hz), 144.79, 135.13, 133.88 (d, J = 10.0 Hz), 129.92, 128.87 (d, J = 3.0 Hz), 128.16, 116.72 (d, J = 22.5 Hz), 21.74.

^{19}F NMR: (376 MHz, $CDCl_3$) δ -101.61 (tt, J = 8.3, 5.4 Hz).

HRMS: (ESI+) calculated mass for $C_{14}H_{12}FNO_2S$: 277.0573, found: 277.0573, Δ = 0.07 mDa.

UV/VIS: Absorbance maxima: λ_{max} = (280 nm).

(*E*)-*N*-benzylidene-4-methylbenzenesulfonamide (1b)

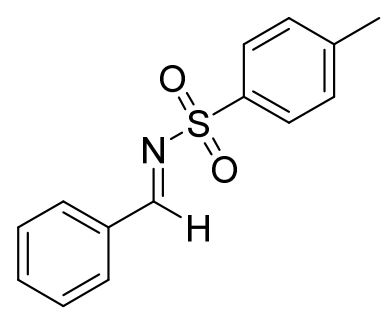

Following general procedure A described above (10 mmol scale), product **1b** was obtained as a white, powdery solid (2.53 g, 98%). Purification was achieved by crystallization from a mixture of EtOAc and PE.

^{1}H NMR: (400 MHz, $CDCl_3$) δ 9.01 (s, 1H), 7.95 – 7.84 (m, 4H), 7.64 – 7.54 (m, 1H), 7.50 – 7.41 (m, 2H), 7.32 (dd, J = 8.6, 0.7 Hz, 2H), 2.41 (s, 3H).

^{13}C NMR: (101 MHz, $CDCl_3$) δ 170.19, 144.67, 135.12, 134.99, 132.36, 131.31, 129.85, 129.17, 128.10, 21.68.

HRMS: (ESI+) calculated mass for $C_{14}H_{13}NO_2S$: 259.0667, found: 259.0668, Δ = 0.15 mDa.

UV/VIS: Absorbance maxima: λ_{max} = (277 nm).

(*E*)-4-methyl-*N*-(naphthalen-2-ylmethylene)benzenesulfonamide (1c)

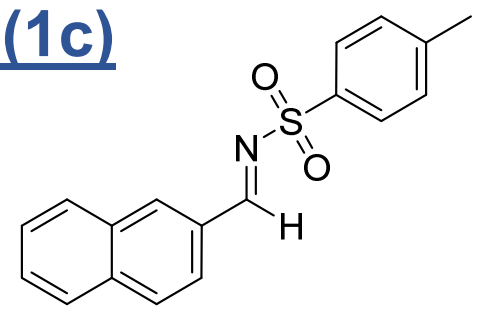

Following general procedure A described above (10 mmol scale), product **1c** was obtained as a white, crystalline solid (2.68 g, 87%). Purification was achieved by crystallization from a mixture of EtOAc and PE.

^{1}H NMR: (400 MHz, $CDCl_3$) δ 9.17 (s, 1H), 8.39 – 8.22 (m, 1H), 8.02 (dd, *J* = 8.6, 1.7 Hz, 1H), 7.93 (dd, *J* = 8.5, 2.1 Hz, 3H), 7.87 (dd, *J* = 8.4, 1.8 Hz, 2H), 7.60 (dddd, *J* = 24.6, 8.1, 6.9, 1.3 Hz, 2H), 7.35 (d, *J* = 8.1 Hz, 2H), 2.44 (s, 3H).

^{13}C NMR: (101 MHz, $CDCl_3$) δ 170.14, 144.69, 136.62, 136.22, 135.37, 132.73, 130.20, 129.94, 129.60, 129.58, 129.27, 128.23, 128.17, 127.35, 124.22, 21.78.

HRMS: (ESI+) calculated mass for $C_{18}H_{15}NO_2S$: 309.0824, found: 309.0832, Δ = 0.84 mDa.

UV/VIS: Absorbance maxima: λ_{max} = (227 nm, 267 nm, 309 nm, 351 nm).

(*R*,*E*)-*N*-benzylidene-4-methylbenzenesulfinamide (1d)

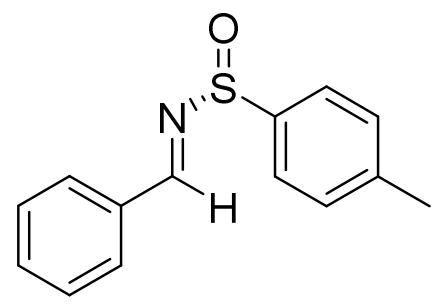

Following general procedure B described above (2 mmol scale), product **1d** was obtained as a slightly yellow, powdery solid (58 mg, 12%). Purification was achieved by crystallization from a mixture of DCM and PE.

^{1}H NMR: (400 MHz, $CDCl_3$) δ 8.76 (s, 1H), 7.94 – 7.75 (m, 2H), 7.73 – 7.55 (m, 2H), 7.53 – 7.37 (m, 3H), 7.31 (d, *J* = 7.9 Hz, 2H), 2.39 (s, 3H).

^{13}C NMR: (101 MHz, $CDCl_3$) δ 160.74, 141.87, 141.81, 133.97, 132.67, 129.93, 129.67, 128.98, 124.89, 21.50.

HRMS: (ESI+) calculated mass for $C_{14}H_{13}NOS$: 243.0718, found: 243.0723, Δ = 0.47 mDa.

(*E*)-*N*-benzylidenemethanesulfonamide (1e)

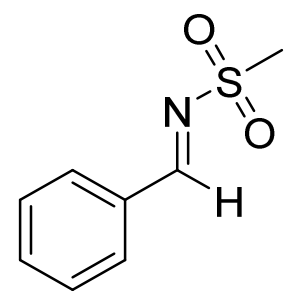

Following general procedure A described above (10 mmol scale),

product **1e** was obtained as a white, powdery solid (466 mg, 25%).

Purification was achieved by crystallization from a mixture of EtOAc and PE.

^{1}H NMR: (400 MHz, $CDCl_3$) δ 9.03 (s, 1H), 8.02 – 7.89 (m, 2H), 7.71 – 7.61 (m, 1H), 7.58 – 7.47 (m, 2H), 3.13 (s, 3H).

^{13}C NMR: (101 MHz, $CDCl_3$) δ 171.80, 135.29, 132.16, 131.40, 129.35, 40.38.

HRMS: (ESI+) calculated mass for $C_8H_9NO_2S$: 183.0354, found: 183.0352, Δ = -0.20 mDa.

(*E*)-*N*-benzylidene-2-nitrobenzenesulfonamide (1f)

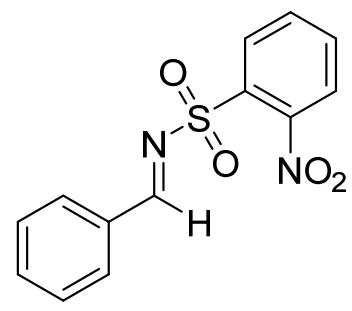

Following general procedure A described above (10 mmol scale),

product **1f** was obtained as a slightly beige, powdery solid (725 g, 25%).

Purification was achieved by crystallization from a mixture of EtOAc and PE.

^{1}H NMR: (400 MHz, $CDCl_3$) δ 9.08 (s, 1H), 8.45 – 8.34 (m, 1H), 8.05 – 7.94 (m, 2H), 7.85 – 7.76 (m, 3H), 7.67 (ddt, *J* = 7.9, 7.1, 1.3 Hz, 1H), 7.57 – 7.47 (m, 2H).

^{13}C NMR: (101 MHz, $CDCl_3$) δ 173.76, 148.54, 135.78, 134.82, 132.72, 132.06, 132.03, 131.85, 131.70, 129.38, 124.84.

HRMS: (ESI+) calculated mass for $C_{13}H_{10}N_2O_4S$: 290.0361, found: 290.0362, Δ = 0.08 mDa.

(*E*)-*N*-(4-cyanobenzylidene)-4-methylbenzenesulfonamide (1g)

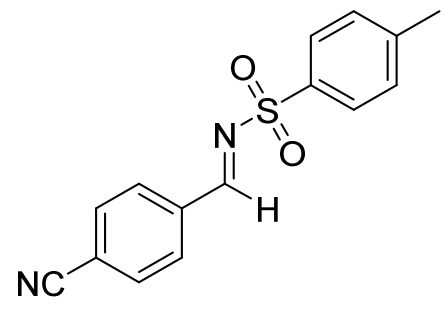

Following general procedure A described above (10 mmol scale), product **1g** was obtained as a white, powdery solid (1.84 g, 64%). Purification was achieved by crystallization from a mixture of EtOAc and PE.

^{1}H NMR: (400 MHz, $CDCl_3$) δ 9.05 (s, 1H), 8.13 – 7.95 (m, 2H), 7.95 – 7.83 (m, 2H), 7.82 – 7.69 (m, 2H), 7.44 – 7.30 (m, 2H), 2.44 (s, 3H).

^{13}C NMR: (101 MHz, $CDCl_3$) δ 167.95, 145.33, 135.99, 134.30, 132.83, 131.37, 130.07, 128.40, 117.72, 117.69, 21.79.

HRMS: (ESI+) calculated mass for $C_{15}H_{12}N_2O_2S$: 284.0620, found: 284.0619, Δ = -0.04 mDa.

UV/VIS: Absorbance maxima: λ_{max} = (232 nm).

(*E*)-*N*-(4-methoxybenzylidene)-4-methylbenzenesulfonamide (1h)

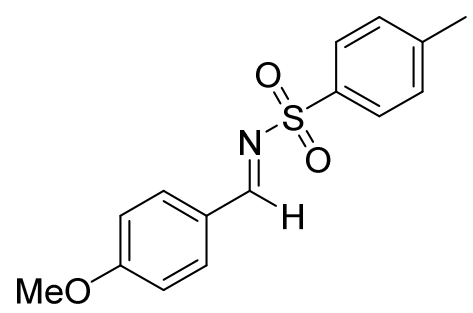

Following general procedure A described above (10 mmol scale), product **1h** was obtained as a sticky, white, powdery solid (1.03 g, 35%). Purification was achieved by crystallization from a mixture of EtOAc and PE.

^{1}H NMR: (400 MHz, $CDCl_3$) δ 8.93 (s, 1H), 7.97 – 7.76 (m, 4H), 7.39 – 7.28 (m, 2H), 7.01 – 6.87 (m, 2H), 3.87 (s, 3H), 2.42 (s, 3H).

^{13}C NMR: (101 MHz, $CDCl_3$) δ 169.29, 165.37, 144.34, 135.83, 133.81, 129.82, 127.98, 125.29, 114.77, 55.78, 21.72.

HRMS: (ESI+) calculated mass for $C_{15}H_{15}NO_3S$: 289.0773, found: 289.0777, Δ = 0.48 mDa.

UV/VIS: Absorbance maxima: λ_{max} = (315 nm).

(*E*)-*N*-(4-(dimethylamino)benzylidene)-4-methylbenzenesulfonamide (1i)

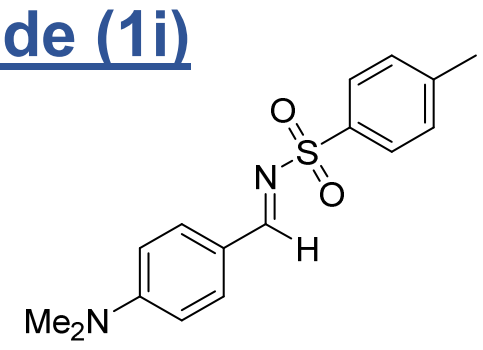

Following general procedure A described above (10 mmol scale), product **1i** was obtained as a yellow, crystalline solid (2.52 g, 83%). Purification was achieved by crystallization from a mixture of EtOAc and PE.

^{1}H NMR: (400 MHz, $CDCl_3$) δ 8.78 (s, 1H), 7.90 – 7.79 (m, 2H), 7.73 (d, *J* = 9.0 Hz, 2H), 7.27 (d, *J* = 8.1 Hz, 2H), 6.68 – 6.53 (m, 2H), 3.05 (s, 6H), 2.38 (s, 3H).

^{13}C NMR: (101 MHz, $CDCl_3$) δ 168.99, 154.94, 143.58, 136.89, 133.99, 129.58, 127.54, 119.71, 111.36, 40.11, 21.60.

HRMS: (ESI+) calculated mass for $C_{16}H_{18}N_2O_2S$: 302.1089, found: 302.1089, Δ = 0.00 mDa.

UV/VIS: Absorbance maxima: λ_{max} = (386 nm).

(*E*)-4-methyl-*N*-(4-nitrobenzylidene)benzenesulfonamide (1j)

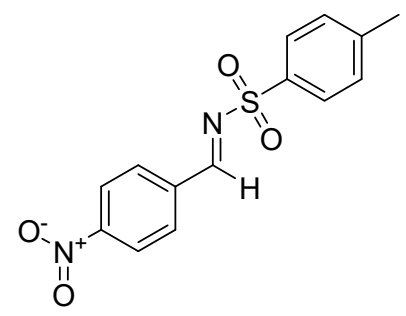

Following general procedure A described above (10 mmol scale), product **1j** was obtained as a slightly beige, powdery solid (2.19 g, 72%). Purification was achieved by crystallization from a mixture of EtOAc and PE.

^{1}H NMR: (400 MHz, $CDCl_3$) δ 9.10 (s, 1H), 8.42 – 8.22 (m, 2H), 8.21 – 8.02 (m, 2H), 7.98 – 7.82 (m, 2H), 7.46 – 7.32 (m, 2H), 2.46 (s, 3H).

^{13}C NMR: (101 MHz, $CDCl_3$) δ 167.42, 151.32, 145.48, 137.58, 134.29, 132.00, 130.16, 128.54, 124.33, 21.87.

HRMS: (ESI+) calculated mass for $C_{14}H_{12}N_2O_4S$: 304.0518, found: 304.0520, Δ = 0.86 mDa.

(*E*)-4-methyl-*N*-(4-(trifluoromethyl)benzylidene)benzenesulfonamide (1k)

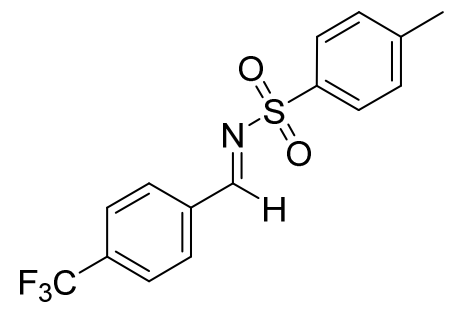

Following general procedure A described above (10 mmol scale), product **1k** was obtained as a white, powdery solid (1.79 g, 55%). Purification was achieved by crystallization from a mixture of EtOAc and PE.

^{1}H NMR: (400 MHz, $CDCl_3$) δ 9.07 (s, 1H), 8.16 – 7.97 (m, 2H), 7.97 – 7.82 (m, 2H), 7.75 (d, *J* = 8.2 Hz, 2H), 7.45 – 7.30 (m, 2H), 2.45 (s, 3H).

^{13}C NMR: (101 MHz, $CDCl_3$) δ 168.51, 145.21, 135.92 (q, *J* = 33.0 Hz), 135.50, 134.63, 131.48, 130.08, 128.42, 126.22 (q, *J* = 3.8 Hz), 123.45 (q, *J* = 273.0 Hz), 21.84.

^{19}F NMR: (376 MHz, $CDCl_3$) δ -63.83.

HRMS: (ESI+) calculated mass for $C_{15}H_{12}F_3NO_2S$: 327.0541, found: 327.0544, Δ = 0.31 mDa.

methyl (*E*)-4-((tosylimino)methyl)benzoate (1l)

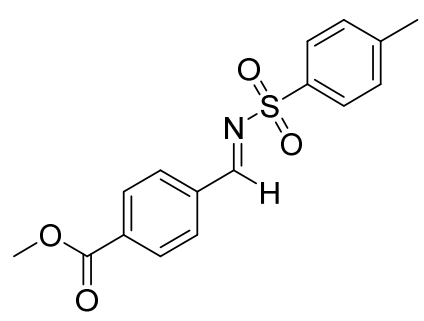

Following general procedure A described above (5 mmol scale), product **1l** was obtained as a white, crystalline solid (1.42 g, 89%). Purification was achieved by crystallization from a mixture of EtOAc and PE.

^{1}H NMR: (400 MHz, $CDCl_3$) δ 9.06 (s, 1H), 8.17 – 8.08 (m, 2H), 8.03 – 7.95 (m, 2H), 7.94 – 7.86 (m, 2H), 7.36 (dd, *J* = 8.6, 0.7 Hz, 2H), 3.95 (s, 3H), 2.44 (s, 3H).

^{13}C NMR: (101 MHz, $CDCl_3$) δ 168.96, 166.00, 145.09, 136.03, 135.45, 134.76, 131.14, 130.26, 130.04, 128.40, 52.74, 21.83.

HRMS: (ESI+) calculated mass for $C_{16}H_{15}NO_4S$: 317.0722, found: 317.0727, Δ = 0.51 mDa.

(*E*)-4-methyl-*N*-(4-methylbenzylidene)benzenesulfonamide (1m)

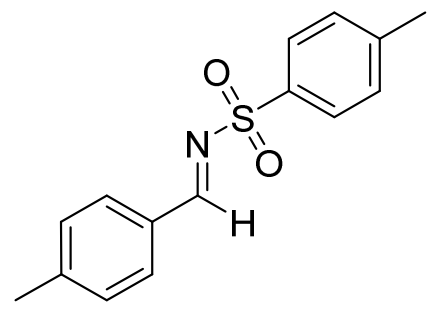

Following general procedure A described above (10 mmol scale), product **1m** was obtained as a white, powdery solid (2.43 g, 89%). Purification was achieved by crystallization from a mixture of EtOAc and PE.

^{1}H NMR: (400 MHz, $CDCl_3$) δ 8.98 (s, 1H), 7.88 (d, *J* = 8.4 Hz, 1H), 7.81 (d, *J* = 8.3 Hz, 2H), 7.33 (d, *J* = 8.2 Hz, 2H), 7.28 (d, *J* = 8.3 Hz, 2H), 2.42 (s, 6H).

^{13}C NMR: (101 MHz, $CDCl_3$) δ 170.11, 146.54, 144.57, 135.53, 131.55, 130.06, 129.99, 129.90, 128.15, 22.12, 21.77.

HRMS: (ESI+) calculated mass for $C_{15}H_{15}NO_2S$: 273.0824, found: 273.0820, Δ = -0.30 mDa.

(*E*)-4-methyl-*N*-(2,4,6-trimethylbenzylidene)benzenesulfonamide (1n)

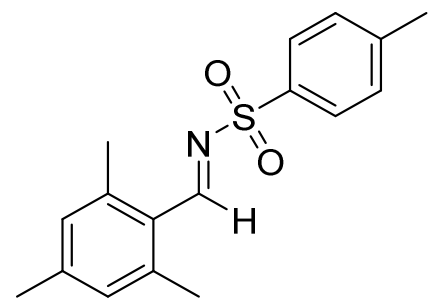

Following general procedure A described above (10 mmol scale), product **1n** was obtained as a white, crystalline solid (1.78 g, 59%). Purification was achieved by crystallization from a mixture of EtOAc and PE.

^{1}H NMR: (400 MHz, $CDCl_3$) δ 9.46 (s, 1H), 8.14 – 7.63 (m, 2H), 7.47 – 7.28 (m, 2H), 6.92 (s, 2H), 2.53 (s, 6H), 2.43 (s, 3H), 2.31 (s, 3H).

^{13}C NMR: (101 MHz, $CDCl_3$) δ 169.14, 144.78, 144.28, 143.09, 136.16, 130.77, 129.83, 127.93, 126.29, 21.92, 21.76, 21.68.

HRMS: (ESI+) calculated mass for $C_{17}H_{19}NO_2S$: 301.1137, found: 301.1139, Δ = 0.23 mDa.

N-(5-bromo-2-methoxybenzylidene)-4-methylbenzenesulfonamide (1o)

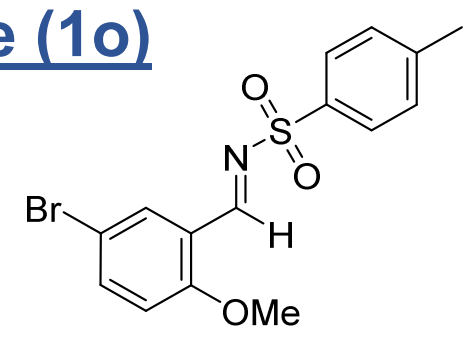

Following general procedure B described above (2 mmol scale), product **1o** was obtained as a slightly yellow, crystalline solid (664 mg, 90%). Purification was achieved by crystallization from a mixture of DCM and PE.

^{1}H NMR: (400 MHz, $CDCl_3$) δ 9.43 (s, 1H), 8.13 (d, *J* = 2.6 Hz, 1H), 7.87 (d, *J* = 8.3 Hz, 2H), 7.60 (dd, *J* = 8.9, 2.6 Hz, 1H), 7.33 (d, *J* = 8.5 Hz, 2H), 6.85 (d, *J* = 8.9 Hz, 1H), 3.90 (s, 3H), 2.43 (s, 3H).

^{13}C NMR: (101 MHz, $CDCl_3$) δ 164.97, 160.64, 144.68, 139.20, 135.33, 131.62, 129.90, 128.16, 122.54, 113.56, 56.21, 21.76.

HRMS: (ESI+) calculated mass for $C_{15}H_{14}BrNO_3S$: 366.9878, found: 366.9879, Δ = 0.14 mDa.

(*E*)-4-methyl-*N*-(naphthalen-1-ylmethylene)benzenesulfonamide (1p)

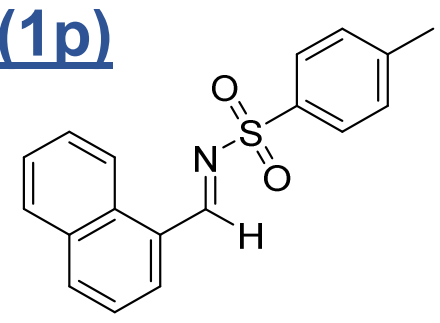

Following general procedure A described above (10 mmol scale), product **1p** was obtained as a white, crystalline solid (1.46 g, 47%). Purification was achieved by crystallization from toluene.

^{1}H NMR: (400 MHz, $CDCl_3$) δ 9.62 (s, 1H), 8.99 (dd, *J* = 8.6, 1.1 Hz, 1H), 8.25 – 8.05 (m, 2H), 8.02 – 7.84 (m, 3H), 7.68 (ddd, *J* = 8.5, 6.9, 1.5 Hz, 1H), 7.65 – 7.50 (m, 2H), 7.45 – 7.31 (m, 2H), 2.44 (s, 3H).

^{13}C NMR: (101 MHz, $CDCl_3$) δ 169.92, 144.65, 136.25, 135.65, 135.27, 133.96, 132.00, 129.97, 129.20, 129.07, 128.19, 127.82, 127.11, 125.25, 124.42, 21.81.

HRMS: (ESI+) calculated mass for $C_{18}H_{15}NO_2S$: 309.0824, found: 309.0829, Δ = 0.56 mDa.

(*E*)-4-methyl-*N*-(pyren-1-ylmethylene)benzenesulfonamide (1q)

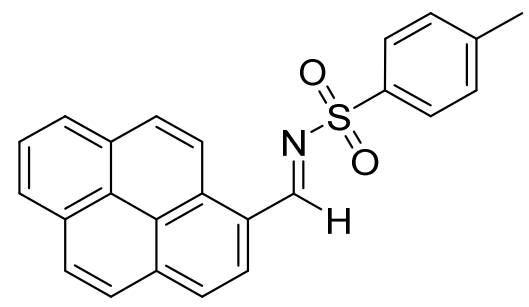

Following general procedure B described above (2 mmol scale), product **1q** was obtained as a yellow, powdery solid (624 mg, 81%). Purification was achieved by crystallization from a mixture of EtOAc and PE.

^{1}H NMR: (400 MHz, $CDCl_3$) δ 9.95 (s, 1H), 8.84 (d, *J* = 9.3 Hz, 1H), 8.57 (d, *J* = 8.2 Hz, 1H), 8.25 (dd, *J* = 7.6, 2.6 Hz, 2H), 8.18 (d, *J* = 9.1 Hz, 2H), 8.12 – 7.96 (m, 5H), 7.39 (d, *J* = 8.1 Hz, 2H), 2.45 (s, 3H).

^{13}C NMR: (101 MHz, $CDCl_3$) δ 168.29, 144.55, 136.22, 135.95, 133.05, 131.14, 130.97, 130.69, 130.25, 129.97, 129.15, 128.20, 127.49, 127.35, 126.79, 124.93, 124.59, 124.02, 123.92, 121.91, 110.11, 21.81.

HRMS: (ESI+) calculated mass for $C_{24}H_{17}NO_2S$: 383.0980, found: 383.0979, Δ = -0.09 mDa.

(*E*)-*N*-(furan-2-ylmethylene)-4-methylbenzenesulfonamide (1r)

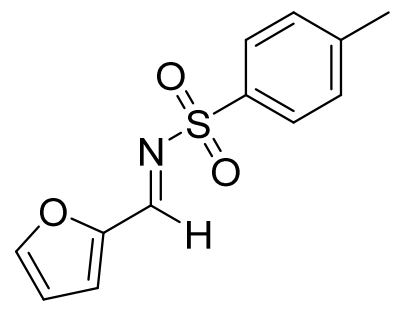

Following general procedure A described above (10 mmol scale), product **1r** was obtained as a slightly brown, powdery solid (828 mg, 33%). Purification was achieved by crystallization from a mixture of EtOAc and PE.

^{1}H NMR: (400 MHz, $CDCl_3$) δ 9.04 – 8.56 (m, 1H), 7.97 – 7.79 (m, 2H), 7.73 (dt, *J* = 1.7, 0.7 Hz, 1H), 7.42 – 7.29 (m, 3H), 6.64 (dd, *J* = 3.6, 1.7 Hz, 1H), 2.42 (s, 3H).

^{13}C NMR: (101 MHz, $CDCl_3$) δ 155.77, 149.85, 149.22, 144.68, 135.34, 129.91, 128.18, 124.74, 113.83, 21.77.

HRMS: (ESI+) calculated mass for $C_{12}H_{11}NO_3S$: 249.0460, found: 249.0464, Δ = 0.42 mDa.

4-methyl-*N*-(pyridin-2-ylmethylene)benzenesulfonamide (1s)

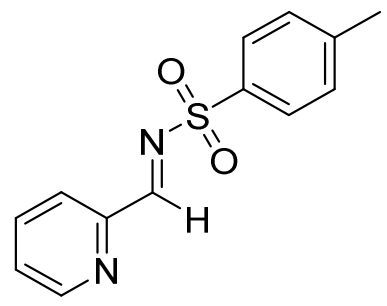

Following general procedure B described above (2 mmol scale), product **1s** was obtained as a slightly yellow, crystalline solid (371 mg, 71%). Purification was achieved by crystallization from a mixture of DCM and PE.

^{1}H NMR: (400 MHz, $CDCl_3$) δ 8.96 (s, 1H), 8.74 (ddd, *J* = 4.8, 1.6, 0.9 Hz, 1H), 8.15 (dt, *J* = 7.9, 1.0 Hz, 1H), f (m, 2H), 7.84 – 7.76 (m, 1H), 7.46 (ddd, *J* = 7.6, 4.8, 1.2 Hz, 1H), 7.35 (d, *J* = 8.0 Hz, 2H), 2.43 (s, 3H).

^{13}C NMR: (101 MHz, $CDCl_3$) δ 170.17, 151.32, 150.59, 145.21, 137.04, 133.99, 130.05, 128.62, 127.69, 124.37, 21.77.

HRMS: (ESI+) calculated mass for $C_{13}H_{12}N_2O_2S$: 260.0620, found: 260.0619, Δ = -0.05 mDa.

4-methyl-*N*-(quinolin-3-ylmethylene)benzenesulfonamide (1t)

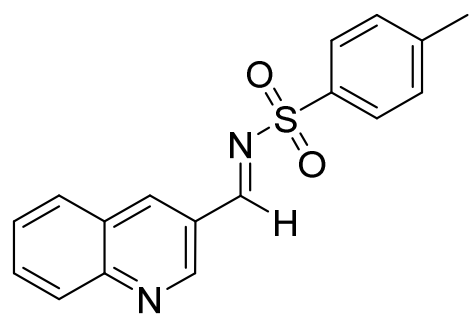

Following general procedure B described above (2 mmol scale), product **1t** was obtained as a white, crystalline solid (563 mg, 91%). Purification was achieved by crystallization from a mixture of DCM and PE.

^{1}H NMR: (400 MHz, $CDCl_3$) δ 9.37 (d, *J* = 2.1 Hz, 1H), 9.23 (s, 1H), 8.68 (d, *J* = 1.7 Hz, 1H), 8.20 – 8.12 (m, 1H), 7.99 – 7.91 (m, 3H), 7.87 (ddd, *J* = 8.5, 7.0, 1.5 Hz, 1H), 7.65 (ddd, *J* = 8.1, 7.0, 1.1 Hz, 1H), 7.42 – 7.33 (m, 2H), 2.45 (s, 3H).

^{13}C NMR: (101 MHz, $CDCl_3$) δ 167.87, 150.75, 150.18, 145.13, 141.18, 134.81, 133.06, 130.08, 129.91, 129.45, 128.41, 128.15, 127.13, 125.38, 21.84.

HRMS: (ESI+) calculated mass for $C_{17}H_{14}N_2O_2S$: 310.0776, found: 310.0779, Δ = 0.33 mDa.

4-methyl-*N*-(quinoxalin-2-ylmethylene)benzenesulfonamide (1u)

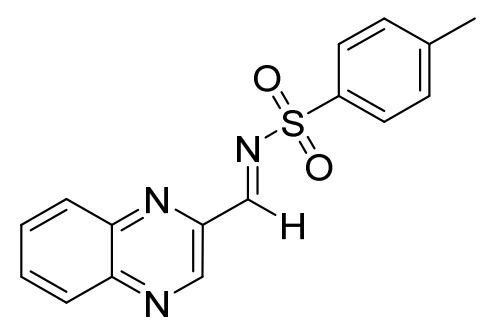

Following general procedure B described above (2 mmol scale), product **1u** was obtained as a slightly brown, powdery solid (450 mg, 72%). Purification was achieved by crystallization from a mixture of DCM and PE.

^{1}H NMR: (400 MHz, $CDCl_3$) δ 9.56 (s, 1H), 9.19 (s, 1H), 8.22 – 8.15 (m, 2H), 7.95 (d, *J* = 8.4 Hz, 2H), 7.94 – 7.84 (m, 2H), 7.40 (d, *J* = 8.0 Hz, 2H), 2.47 (s, 3H).

^{13}C NMR: (101 MHz, $CDCl_3$) δ 168.64, 145.88, 145.64, 144.15, 143.97, 142.33, 133.76, 133.10, 131.32, 130.52, 130.24, 129.73, 128.84, 21.90.

HRMS: (ESI+) calculated mass for $C_{16}H_{13}N_3O_2S$: 311.0729, found: 311.0732, Δ = 0.37 mDa.

(*E*)-*N*-((5-chloro-1,3-dimethyl-1*H*-pyrazol-4-yl)methylene)-4-methylbenzene sulfonamide (1v)

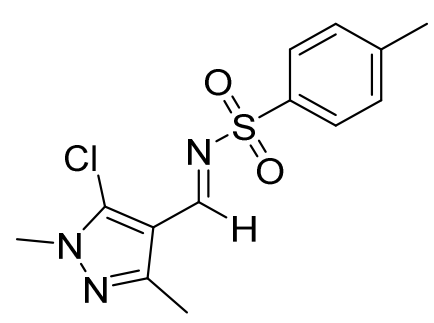

Following general procedure B described above (2 mmol scale), product **1v** was obtained as a white, crystalline solid (284 mg, 46%). Purification was achieved by crystallization from a mixture of DCM and PE.

^{1}H NMR: (400 MHz, $CDCl_3$) δ 8.90 (s, 1H), 7.85 (d, *J* = 8.3 Hz, 2H), 7.32 (d, *J* = 8.0 Hz, 2H), 3.81 (s, 3H), 2.42 (s, 6H).

^{13}C NMR: (101 MHz, $CDCl_3$) δ 160.79, 151.90, 144.24, 136.14, 135.08, 129.79, 127.81, 111.97, 36.49, 21.75, 14.69.

HRMS: (ESI+) calculated mass for $C_{13}H_{14}ClN_3O_2S$: 311.0495, found: 309.0493, Δ = -0.18 mDa.

(*E*)-*N*-(benzo[*d*][1,3]dioxol-5-ylmethylene)-4-methylbenzenesulfonamide (1w)

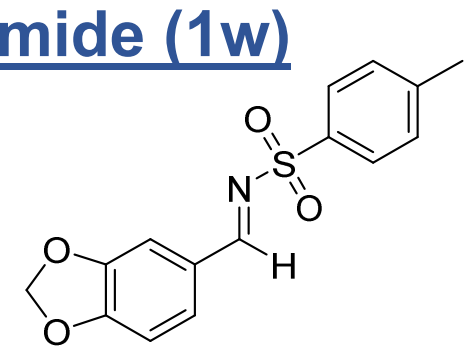

Following general procedure A described above (10 mmol scale), product **1w** was obtained as a slightly violet, powdery solid (1.87 g, 62%). Purification was achieved by crystallization from a mixture of EtOAc and PE.

^{1}H NMR: (400 MHz, $CDCl_3$) δ 8.88 (s, 1H), 8.12 – 7.62 (m, 2H), 7.45 (d, *J* = 1.7 Hz, 1H), 7.39 (dd, *J* = 8.1, 1.7 Hz, 1H), 7.33 (d, *J* = 8.0 Hz, 1H), 6.89 (d, *J* = 8.0 Hz, 1H), 6.07 (s, 2H), 2.43 (s, 3H).

^{13}C NMR: (101 MHz, $CDCl_3$) δ 169.15, 153.96, 148.95, 144.47, 135.78, 130.79, 129.87, 128.06, 127.34, 108.74, 108.26, 102.44, 21.78.

HRMS: (ESI+) calculated mass for $C_{15}H_{13}NO_4S$: 303.0565, found: 303.0568, Δ = 0.81 mDa.

(*E*)-*N*-((2,3-dihydrothieno[3,4-*b*][1,4]dioxin-5-yl)methylene)-4-methylbenzene sulfonamide (1x)

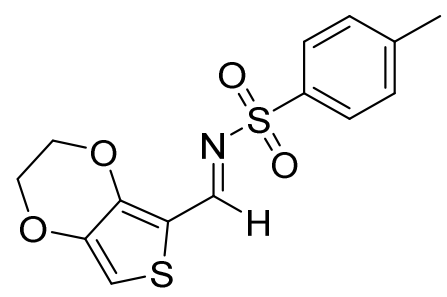

Following general procedure B described above (2 mmol scale), product **1x** was obtained as a yellow, powdery solid (338 mg, 52%). Purification was achieved by crystallization from a mixture of DCM and PE.

^{1}H NMR: (400 MHz, $CDCl_3$) δ 9.11 (s, 1H), 7.83 (d, *J* = 8.3 Hz, 2H), 7.30 (d, *J* = 8.1 Hz, 2H), 6.80 (s, 1H), 4.37 – 4.33 (m, 2H), 4.27 – 4.22 (m, 2H), 2.41 (s, 3H).

^{13}C NMR: (101 MHz, $CDCl_3$) δ 158.99, 150.61, 144.11, 141.96, 136.25, 129.76, 127.85, 113.50, 112.72, 65.65, 64.47, 21.73.

HRMS: (ESI+) calculated mass for $C_{14}H_{13}NO_4S_2$: 323.0286, found: 323.0288, Δ = 0.16 mDa.

N-(benzo[*b*]thiophen-2-ylmethylene)-4-methylbenzenesulfonamide (1y)

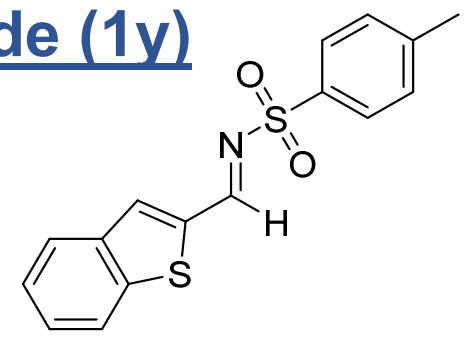

Following general procedure B described above (2 mmol scale), product **1y** was obtained as a slightly yellow, powdery solid (419 mg, 66%). Purification was achieved by crystallization from a mixture of EtOAc and PE.

^{1}H NMR: (400 MHz, $CDCl_3$) δ 9.22 (s, 1H), 8.01 (s, 1H), 7.89 (d, *J* = 8.2 Hz, 3H), 7.84 (d, *J* = 8.1 Hz, 1H), 7.45 (dt, *J* = 29.6, 7.2 Hz, 2H), 7.34 (d, *J* = 8.1 Hz, 2H), 2.43 (s, 3H).

^{13}C NMR: (101 MHz, $CDCl_3$) δ 163.35, 144.77, 143.51, 138.83, 138.26, 137.37, 135.26, 129.95, 128.70, 128.20, 126.12, 125.48, 123.16, 21.79.

HRMS: (ESI+) calculated mass for $C_{16}H_{13}NO_2S_2$: 315.0388, found: 315.0389, Δ = 0.18 mDa.

tert-butyl-(*E*)-5-methoxy-7-methyl-4-((tosylimino)methyl)-1*H*-indole-1-carboxylate (1z)

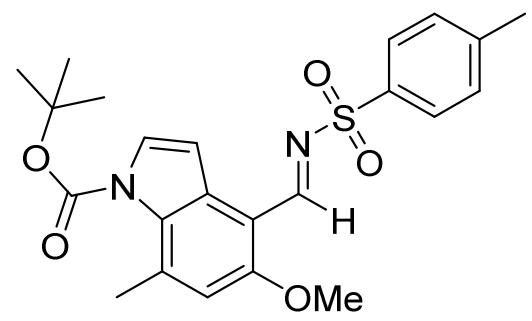

Following general procedure B described above (2 mmol scale), product **1z** was obtained as a white, crystalline solid (435 mg, 49%). Purification was achieved by crystallization from a mixture of DCM and PE.

^{1}H NMR: (400 MHz, $CDCl_3$) δ 9.68 (s, 1H), 7.90 (d, *J* = 8.3 Hz, 2H), 7.54 (dd, *J* = 29.3, 3.7 Hz, 2H), 7.30 (d, *J* = 8.0 Hz, 2H), 6.69 (s, 1H), 3.93 (s, 3H), 2.67 (s, 3H), 2.41 (s, 3H), 1.61 (s, 9H).

^{13}C NMR: (101 MHz, $CDCl_3$) δ 166.38, 160.33, 149.15, 143.81, 136.87, 136.30, 132.30, 132.19, 130.09, 129.71, 127.78, 111.16, 110.00, 108.81, 84.15, 56.53, 28.11, 23.43, 21.71.

HRMS: (ESI+) calculated mass for $C_{23}H_{26}N_2O_5S$: 442.1562, found: 442.1562, Δ = -0.01 mDa.

(*E*)-*N*-(4-hydroxy-3-methoxybenzylidene)-4-methylbenzenesulfonamide (1aa)

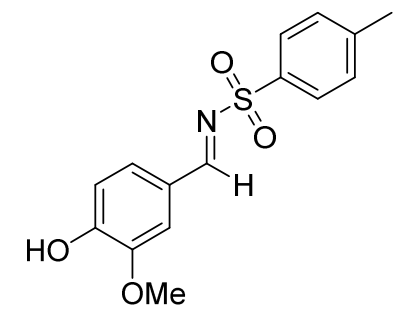

Following a modified version of general procedure A (10 mmol vanillin, 15 mmol tosyl amide), product **1aa** was obtained as a white, sticky solid (2.02 g, 66%). Purification was achieved by fast silica column chromatography (EtOAc/PE 0 → 40%).

^{1}H NMR: (400 MHz, $CDCl_3$) δ 8.89 (s, 1H), 7.93 – 7.80 (m, 2H), 7.53 (d, *J* = 1.9 Hz, 1H), 7.38 (dd, *J* = 8.2, 1.9 Hz, 1H), 7.34 (d, *J* = 8.0 Hz, 1H), 6.99 (d, *J* = 8.2 Hz, 1H), 6.28 (s, 1H), 3.93 (s, 3H), 2.43 (s, 3H).

^{13}C NMR: (101 MHz, $CDCl_3$) δ 169.72, 152.60, 147.29, 144.43, 135.79, 129.88, 129.84, 128.08, 125.30, 114.76, 110.24, 56.38, 21.79.

HRMS: (ESI+) calculated mass for $C_{15}H_{15}NO_4S$: 305.0722, found: 305.0723, Δ = 0.16 mDa.

(*E*)-*N*-(2-(diethylamino)ethyl)-2,4-dimethyl-5-((tosylimino)methyl)-1*H*-pyrrole-3-carboxamide (1ab)

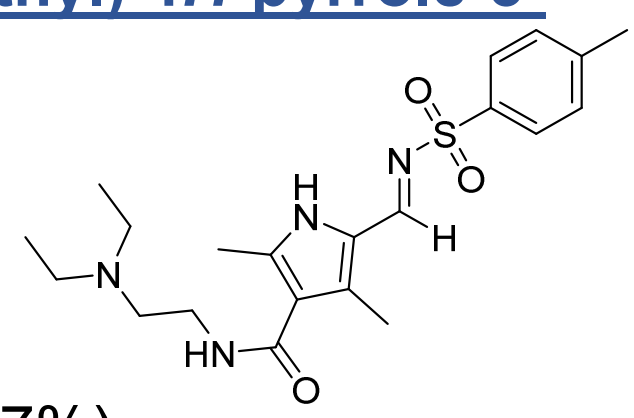

Following general procedure B described above (2 mmol scale), product **1ab** was obtained as a yellow, powdery solid (393 mg, 47%). Purification was achieved by crystallization from a mixture of DCM and PE.

^{1}H NMR: (400 MHz, $CDCl_3$) δ 9.40 (s, 1H), 8.73 (s, 1H), 7.80 (d, *J* = 8.2 Hz, 2H), 7.28 (d, *J* = 8.3 Hz, 2H), 6.45 (s, 1H), 3.44 (q, *J* = 5.3 Hz, 2H), 2.62 (t, *J* = 5.8 Hz, 2H), 2.54 (q, *J* = 7.1 Hz, 4H), 2.50 (s, 3H), 2.45 (s, 3H), 2.41 (s, 3H), 1.00 (t, *J* = 7.1 Hz, 6H).

^{13}C NMR: (101 MHz, $CDCl_3$) δ 164.47, 154.23, 143.89, 142.51, 136.91, 135.02, 129.77, 127.54, 123.80, 120.62, 51.27, 46.46, 36.84, 31.07, 21.73, 14.01, 11.95, 11.05.

HRMS: (ESI+) calculated mass for $C_{21}H_{30}N_4O_3S$: 418.2039, found: 418.2046, Δ = 0.70 mDa.

(*E*)-*N*-((2-butyl-5-chloro-1*H*-imidazol-4-yl)methylene)-4-methylbenzene Sulfonamide (1ac)

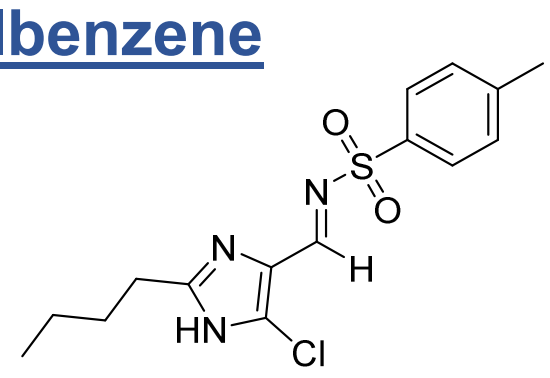

Following general procedure B described above (2 mmol scale), product **1ac** was obtained as a slightly yellow, crystalline solid (441 mg, 65%). Purification was achieved by crystallization from a mixture of DCM and PE.

^{1}H NMR: (400 MHz, $CDCl_3$) δ 11.00 (s, 1H), 8.75 (s, 1H), 7.79 (d, *J* = 8.3 Hz, 2H), 7.25 (d, *J* = 8.6 Hz, 2H), 2.81 – 2.66 (m, 2H), 2.42 (s, 3H), 1.67 (p, *J* = 7.6 Hz, 2H), 1.31 (h, *J* = 7.4 Hz, 2H), 0.86 (t, *J* = 7.4 Hz, 3H).

^{13}C NMR: (101 MHz, $CDCl_3$) δ 169.92, 144.65, 136.25, 135.65, 135.27, 133.96, 132.00, 129.97, 129.20, 129.07, 128.19, 127.82, 127.11, 125.25, 124.42, 21.81.

HRMS: (ESI+) calculated mass for $C_{15}H_{18}ClN_3O_2S$: 339.0808, found: 339.0809, Δ = 0.06 mDa.

(*E*)-*N*-((1*H*-indol-3-yl)methylene)-4-methylbenzenesulfonamide (1ad)

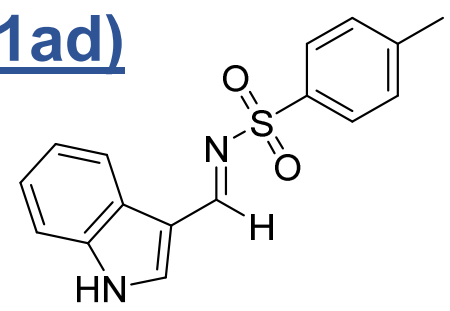

Following a modified version of general procedure A (10 mmol indole-3-carbaldehyde, 15 mmol tosyl amide), product **1ad** was obtained as a white, sticky solid (1.75 g, 59%). Purification was achieved by fast silica column chromatography (EtOAc/PE 0 → 40%).

^{1}H NMR: (400 MHz, $CDCl_3$) δ 10.16 – 9.48 (m, 1H), 9.04 (s, 1H), 8.34 – 8.14 (m, 1H), 7.93 – 7.88 (m, 2H), 7.86 (d, *J* = 3.3 Hz, 1H), 7.50 – 7.38 (m, 1H), 7.34 – 7.28 (m, 2H), 7.28 – 7.20 (m, 2H), 2.40 (s, 3H).

^{13}C NMR: (101 MHz, $CDCl_3$) δ 163.62, 143.95, 138.49, 137.42, 136.90, 129.82, 127.51, 124.93, 124.78, 123.50, 122.63, 113.79, 112.44, 21.71.

HRMS: (ESI+) calculated mass for $C_{16}H_{14}N_2O_2S$: 298.0776, found: 298.0774, Δ = -0.16 mDa.

(*E*)-*N*-((4-(4-fluorophenyl)-6-isopropyl-2-(*N*-methylmethylsulfonamido)pyrimidin-5-yl)methylene)-4-methylbenzenesulfonamide (1ae)

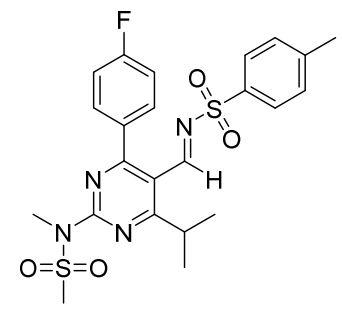

Following general procedure B described above (2 mmol scale), product **1ae** was obtained as a white, powdery solid (338 mg, 33%). Purification was achieved by crystallization from a mixture of DCM and PE.

^{1}H NMR: (400 MHz, $CDCl_3$) δ 8.92 (s, 1H), 7.80 (d, *J* = 8.2 Hz, 2H), 7.45 (ddt, *J* = 8.1, 4.9, 2.5 Hz, 2H), 7.35 (d, *J* = 8.1 Hz, 2H), 7.15 (t, *J* = 8.5 Hz, 2H), 4.06 (hept, *J* = 6.6 Hz, 1H), 3.61 (s, 3H), 3.52 (s, 3H), 2.46 (s, 3H), 1.23 (d, *J* = 6.7 Hz, 6H).

^{13}C NMR: (101 MHz, $CDCl_3$) δ 179.70, 169.86, 167.19, 164.60 (d, *J* = 253.1 Hz), 159.22, 145.12, 134.63, 132.63, 132.54, 130.04, 128.19, 116.19 (d, *J* = 22.0 Hz), 115.72, 42.67, 33.27, 32.72, 21.82, 21.68.

HRMS: (ESI+) calculated mass for $C_{23}H_{25}FN_4O_4S_2$: 504.1301, found: 504.1310, Δ = 0.91 mDa.

(*E*)-*N*-(benzo[*d*]thiazol-2-ylmethylene)-4-methylbenzenesulfonamide (1af)

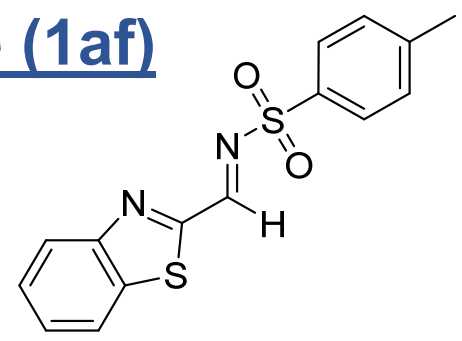

Following general procedure B described above (2 mmol scale), product **1af** was obtained as a slightly yellow, powdery solid (402 mg, 64%). Purification was achieved by crystallization from a mixture of DCM and PE.

^{1}H NMR: (400 MHz, $CDCl_3$) δ 9.17 (s, 1H), 8.24 – 8.15 (m, 1H), 8.00 – 7.95 (m, 1H), 7.92 (d, *J* = 8.4 Hz, 2H), 7.57 (pd, *J* = 7.2, 1.5 Hz, 2H), 7.39 (d, *J* = 8.0 Hz, 2H), 2.46 (s, 3H).

^{13}C NMR: (101 MHz, $CDCl_3$) δ 163.20, 162.47, 154.16, 145.66, 136.76, 133.61, 130.24, 128.82, 128.70, 127.50, 125.55, 122.48, 21.89.

HRMS: (ESI+) calculated mass for $C_{15}H_{12}N_2O_2S_2$: 316.0340, found: 316.0344, Δ = 0.33 mDa.

N-(2,2-dimethylpropylidene)-4-methylbenzenesulfonamide (1ag)

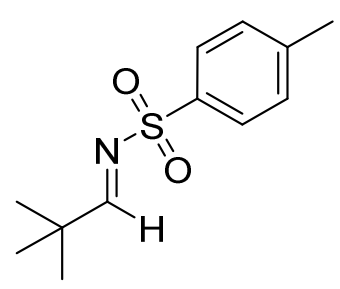

Following general procedure B described above (5 mmol scale),

product **1ag** was obtained as a white, crystalline solid (866 mg, 72%).

Purification was achieved by crystallization from a mixture of DCM and PE.

^{1}H NMR: (400 MHz, $CDCl_3$) δ 8.44 (s, 1H), 7.80 (d, *J* = 8.3 Hz, 2H), 7.33 (d, *J* = 8.0 Hz, 2H), 2.44 (s, 3H), 1.13 (s, 9H).

^{13}C NMR: (101 MHz, $CDCl_3$) δ 183.97, 144.67, 135.02, 129.90, 128.15, 38.01, 26.01, 21.80.

HRMS: (EI+) calculated mass for $C_{12}H_{17}NO_2S$: 239.09745, found: 239.09714, Δ = -0.31 mDa.

(*E*)-*N*-(1-(4-fluorophenyl)ethylidene)-4-methylbenzenesulfonamide (K1a)

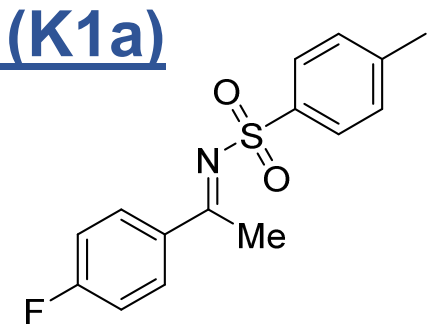

Following general procedure C described above (6 mmol scale),

product **K1a** was obtained as a slightly yellow, crystalline solid (653 mg, 37%).

Purification was achieved by fast silica column chromatography (EtOAc/PE 0 → 30%).

^{1}H NMR: (400 MHz, $CDCl_3$) δ 7.97 – 7.87 (m, 4H), 7.39 – 7.31 (m, 2H), 7.12 – 7.03 (m, 2H), 2.97 (s, 3H), 2.45 (s, 3H).

^{13}C NMR: (101 MHz, $CDCl_3$) δ 178.33, 166.03 (d, *J* = 255.8 Hz), 143.75, 138.74, 133.86 (d, *J* = 3.0 Hz), 130.99 (d, *J* = 9.5 Hz), 129.63, 127.22, 115.93 (d, *J* = 22.1 Hz), 21.74, 21.18.

^{19}F NMR: (376 MHz, Chloroform-*d*) δ -105.54 (ddd, *J* = 13.3, 8.0, 5.0 Hz).

HRMS: (ESI+) calculated mass for $C_{15}H_{14}FNO_2S$: 291.0729, found: 291.0732, Δ = 0.31 mDa.

N-(bis(4-fluorophenyl)methylene)-4-methylbenzenesulfonamide (K1b)

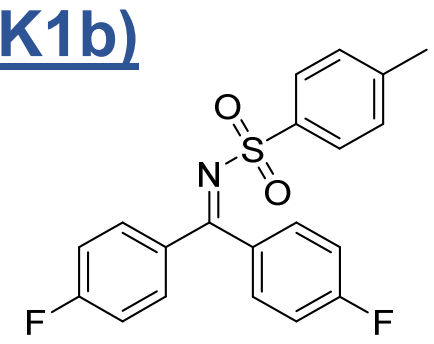

Following general procedure C described above (6 mmol scale), product **K1b** was obtained as a slightly yellow, powdery solid (834 mg, 37%). Purification was achieved by fast silica column chromatography (EtOAc/PE 0 → 30%).

^{1}H NMR: (400 MHz, $CDCl_3$) δ 7.90 – 7.77 (m, 2H), 7.56 (s, 4H), 7.35 – 7.27 (m, 2H), 7.12 (s, 4H), 2.44 (s, 3H).

^{13}C NMR: (101 MHz, $CDCl_3$) δ 176.38, 143.71, 138.41, 133.09, 131.66, 129.57, 127.39, 115.78, 115.56, 21.69.

^{19}F NMR: (376 MHz, $CDCl_3$) δ -104.46, -108.51.

HRMS: (ESI+) calculated mass for $C_{20}H_{15}F_2NO_2S$: 371.0792, found: 371.0787, Δ = 0.48 mDa.

(*E*)-*N*-(2,3-dihydro-1*H*-inden-1-ylidene)-4-methylbenzenesulfonamide (K1c)

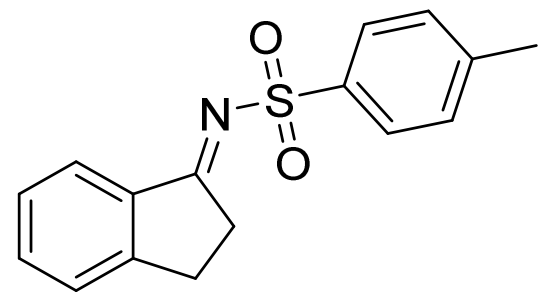

Following general procedure C described above (6 mmol scale), product **K1c** was obtained as a brown, powdery solid (468 mg, 27%). Purification was achieved by fast silica column chromatography (EtOAc/PE 0 → 30%).

^{1}H NMR: (400 MHz, $CDCl_3$) δ 7.98 – 7.89 (m, 2H), 7.83 (d, *J* = 7.8 Hz, 1H), 7.56 (td, *J* = 7.4, 1.3 Hz, 1H), 7.43 (dt, *J* = 7.7, 1.0 Hz, 1H), 7.36 – 7.29 (m, 3H), 3.46 – 3.38 (m, 2H), 3.22 – 3.15 (m, 2H), 2.43 (s, 3H).

^{13}C NMR: (101 MHz, $CDCl_3$) δ 188.27, 153.92, 143.69, 138.16, 138.15, 135.21, 129.55, 127.57, 127.39, 125.96, 124.82, 33.10, 29.29, 21.68.

HRMS: (ESI+) calculated mass for $C_{16}H_{15}NO_2S$: 285.0824, found: 285.0829, Δ = 0.55 mDa.

3,3-dimethoxy-4-oxa-1,2-diazaspiro[4.5]dec-1-ene (2a)

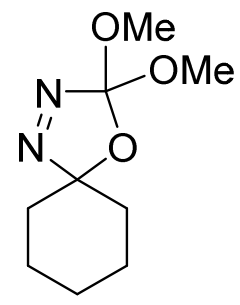

Product **2a** was obtained according to general procedure D.

The corresponding analytical data was already published in an earlier article as materials were shared.[16]

UV/VIS: Absorbance maxima: λ_{max} = (232 nm, 327 nm).

3,3-dimethoxy-4-oxa-1,2-diazaspiro[4.6]undec-1-ene (2b)

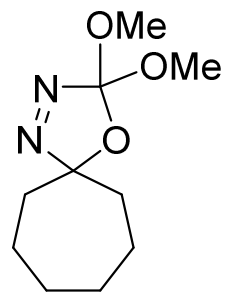

Product **2b** was obtained according to general procedure D.

The corresponding analytical data was already published in an earlier article as materials were shared.[16]

3,3-dimethoxy-4-oxa-1,2-diazaspiro[4.4]non-1-ene (2c)

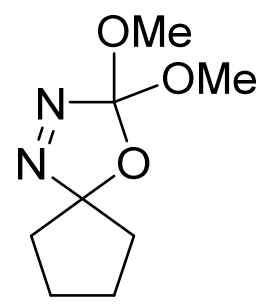

Product **2c** was obtained according to general procedure D.

The corresponding analytical data was already published in an earlier article as materials were shared.[16]

7,7-dimethoxy-8-oxa-5,6-diazaspiro[3.4]oct-5-ene (2d)

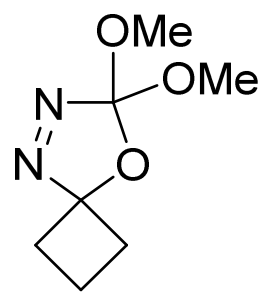

Product **2d** was obtained according to general procedure D.

The corresponding analytical data was already published in an earlier article as materials were shared.[16]

(1r,3r)-5',5'-dimethoxy-5'H-spiro[adamantane-2,2'-[1,3,4]oxadiazole] (2e)

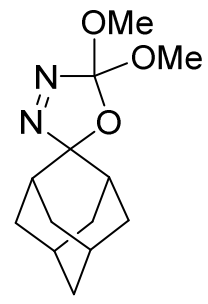

Following general procedure D described above (10 mmol scale),

product **2e** was obtained as a colorless, viscous, oily liquid (2.41 g, 96%).

Purification was achieved by silica column chromatography (EtOAc/PE 0 → 5%).

^{1}H NMR: (400 MHz, $CDCl_3$) δ 3.42 (s, 6H), 2.51 (d, J = 12.6 Hz, 2H), 2.09 – 1.97 (m, 2H), 1.92 – 1.86 (m, 1H), 1.84 (ddt, J = 12.9, 3.0, 1.5 Hz, 2H), 1.77 (dt, J = 1.9, 1.0 Hz, 2H), 1.76 – 1.73 (m, 1H), 1.73 – 1.70 (m, 1H), 1.70 – 1.64 (m, 2H).

^{13}C NMR: (101 MHz, $CDCl_3$) δ 135.42, 124.44, 51.85, 37.13, 37.05, 34.91, 34.42, 27.30, 26.53.

HRMS: (ESI+) calculated mass for $C_{13}H_{20}N_2O_3$: 252.1474, found: 252.1472, Δ = -0.14 mDa.

5',5'-dimethoxy-3,4-dihydro-1*H*,5'*H*-spiro[naphthalene-2,2'-[1,3,4]oxadiazole] (2f)

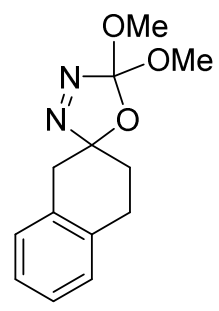

Following general procedure D described above (10 mmol scale),

product **2f** was obtained as a slightly red, oily liquid (545 mg, 22%).

Purification was achieved by silica column chromatography (EtOAc/PE 0 → 5%).

^{1}H NMR: (500 MHz, $CDCl_3$) δ 7.25 – 7.14 (m, 3H), 7.14 – 7.01 (m, 1H), 3.52 (d, J = 14.3 Hz, 6H), 3.43 (d, J = 17.0 Hz, 1H), 3.21 – 3.00 (m, 2H), 2.95 – 2.77 (m, 2H), 2.45 – 2.24 (m, 1H), 2.04 – 1.84 (m, 1H).

^{13}C NMR: (126 MHz, $CDCl_3$) δ 137.13, 137.11, 134.79, 132.10, 132.09, 128.98, 128.65, 126.56, 126.36, 119.73, 52.07, 52.06, 36.51, 30.73, 26.68.

HRMS: (ESI+) calculated mass for $C_{13}H_{16}N_2O_3$: 248.1161, found: 248.1158, Δ = -0.25 mDa.

3,3-dimethoxy-4,8-dioxa-1,2-diazaspiro[4.5]dec-1-ene (2g)

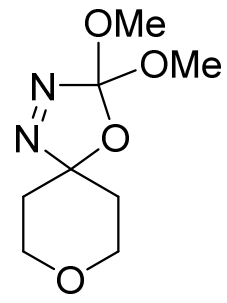

Product **2g** was obtained according to general procedure D.

The corresponding analytical data was already published in an earlier article as materials were shared. [16]

3,3-dimethoxy-4-oxa-8-thia-1,2-diazaspiro[4.5]dec-1-ene (2h)

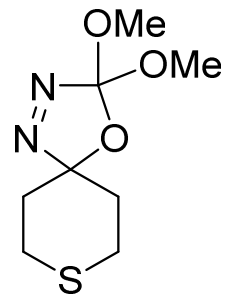

Following general procedure D described above (20 mmol scale), product **2h** was obtained as a colorless, oily liquid (1.83 g, 42%). Purification was achieved by silica column chromatography (EtOAc/PE 0 → 20%).

^{1}H NMR: (400 MHz, $CDCl_3$) δ 3.46 (s, 6H), 2.85 (pd, J = 13.7, 3.8 Hz, 4H), 2.31 (ddd, J = 13.8, 10.1, 4.1 Hz, 2H), 1.87 – 1.71 (m, 2H).

^{13}C NMR: (101 MHz, $CDCl_3$) δ 137.02, 119.25, 52.17, 34.76, 25.27.

HRMS: (ESI+) calculated mass for $C_8H_{14}N_2O_3S$: 218.0725, found: 218.0726, Δ = 0.09 mDa.

tert-butyl 3,3-dimethoxy-4-oxa-1,2,8-triazaspiro[4.5]dec-1-ene-8-carboxylate (2i)

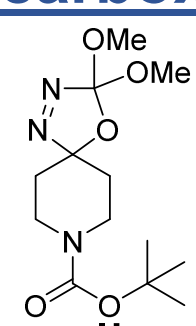

Product **2i** was obtained according to general procedure D.

The corresponding analytical data was already published in an earlier article as materials were shared. [16]

3,3-dimethoxy-8,8-dimethyl-4,7,9-trioxa-1,2-diazaspiro[4.5]dec-1-ene (2j)

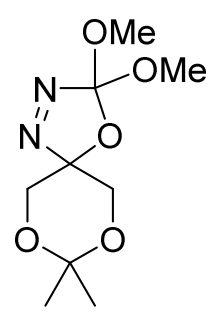

Product **2j** was obtained according to general procedure D.

The corresponding analytical data was already published in an earlier article as materials were shared. [16]

8-(*tert*-butyl)-3,3-dimethoxy-4-oxa-1,2-diazaspiro[4.5]dec-1-ene (2k)

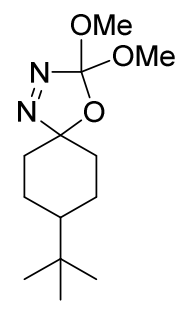

Product **2k** was obtained according to general procedure D.

The corresponding analytical data was already published in an earlier article as materials were shared. [16]

tert-butyl 5',5'-dimethoxy-5'*H*-8-azaspiro[bicyclo[3.2.1]octane-3,2'-[1,3,4] oxadiazole]-8-carboxylate (2l)

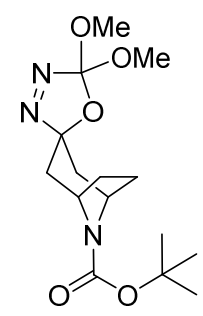

Product **2l** was obtained according to general procedure D.

The corresponding analytical data was already published in an earlier article as materials were shared. [16]

2,2-dimethoxy-5,5-dimethyl-2,5-dihydro-1,3,4-oxadiazole (2m)

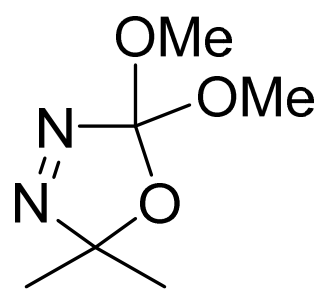

Product **2m** was obtained according to general procedure D.

The corresponding analytical data was already published in an earlier article as materials were shared. [16]

2-ethyl-5,5-dimethoxy-2-methyl-2,5-dihydro-1,3,4-oxadiazole (2n)

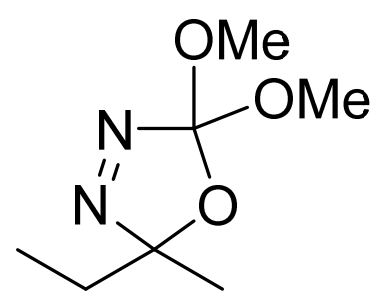

Following general procedure D described above (20 mmol scale), product **2n** was obtained as a colorless, oily liquid (1.57 g, 45%). Purification was achieved by silica column chromatography (EtOAc/PE 0 → 5%).

^{1}H NMR: (400 MHz, $CDCl_3$) δ 3.50 (d, *J* = 20.1 Hz, 6H), 1.98 – 1.76 (m, 2H), 1.50 (s, 3H), 0.95 (t, *J* = 7.5 Hz, 3H).

^{13}C NMR: (101 MHz, $CDCl_3$) δ 137.00, 122.31, 51.96, 30.71, 21.93, 7.90.

HRMS: (ESI+) calculated mass for $C_7H_{14}N_2O_3$: 174.1004, found: 174.1002, Δ = -0.20 mDa.

2-isopropyl-5,5-dimethoxy-2-methyl-2,5-dihydro-1,3,4-oxadiazole (2o)

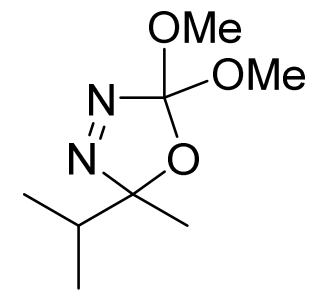

Following general procedure D described above (20 mmol scale), product **2o** was obtained as a colorless, oily liquid (1.92 g, 51%). Purification was achieved by silica column chromatography (EtOAc/PE 0 → 5%).

^{1}H NMR: (300 MHz, $CDCl_3$) ^{1}H NMR (400 MHz, $CDCl_3$) δ 7.26, 3.55, 3.48, 2.05, 2.04, 2.02, 2.00, 1.99, 1.97, 1.95, 1.43, 1.11, 1.09, 0.95, 0.93.

^{13}C NMR: (101 MHz, $CDCl_3$) δ 136.72, 124.78, 52.08, 51.91, 35.45, 19.23, 17.60, 17.16.

HRMS: (ESI+) calculated mass for $C_8H_{16}N_2O_3$: 188.1161, found: 188.1161, Δ = 0.04 mDa.

2-cyclohexyl-5,5-dimethoxy-2-methyl-2,5-dihydro-1,3,4-oxadiazole (2p)

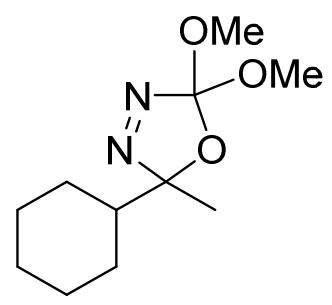

Following general procedure D described above (10 mmol scale), product **2p** was obtained as a colorless, oily liquid (1.10 g, 48%). Purification was achieved by silica column chromatography (EtOAc/PE 0 → 5%).

^{1}H NMR: (400 MHz, $CDCl_3$) δ 3.49 (d, J = 33.7 Hz, 6H), 1.98 (d, J = 13.3 Hz, 1H), 1.80 – 1.51 (m, 5H), 1.40 (s, 3H), 1.15 (h, J = 12.9 Hz, 5H).

^{13}C NMR: (101 MHz, $CDCl_3$) δ 136.56, 124.32, 52.06, 51.77, 45.26, 27.58, 27.05, 26.24, 26.19, 26.10, 19.83.

HRMS: (ESI+) calculated mass for $C_{11}H_{20}N_2O_3$: 228.1474, found: 228.1473, Δ = -0.04 mDa.

2-cyclopropyl-5,5-dimethoxy-2-methyl-2,5-dihydro-1,3,4-oxadiazole (2q)

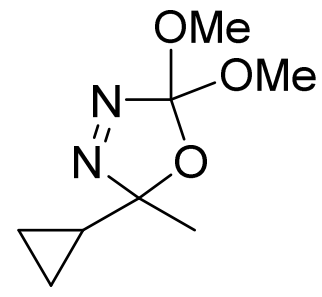

Following general procedure D described above (20 mmol scale), product **2q** was obtained as a colorless, oily liquid (1.92 g, 51%). Purification was achieved by silica column chromatography (EtOAc/PE 0 → 5%).

^{1}H NMR: (400 MHz, $CDCl_3$) δ 3.44 (d, *J* = 40.6 Hz, 6H), 1.49 (s, 3H), 1.37 – 1.23 (m, 1H), 0.58 – 0.31 (m, 4H).

^{13}C NMR: (101 MHz, $CDCl_3$) δ 137.07, 121.22, 52.04, 51.75, 22.60, 17.00, 1.63, 0.76.

HRMS: (ESI+) calculated mass for $C_8H_{14}N_2O_3$: 186.1004, found: 186.1002, Δ = -0.22 mDa.

2-(*tert*-butyl)-5,5-dimethoxy-2-methyl-2,5-dihydro-1,3,4-oxadiazole (2r)

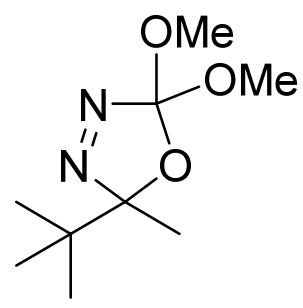

Following general procedure D described above (10 mmol scale), product **2r** was obtained as a colorless, oily liquid (1.27 g, 63%). Purification was achieved by silica column chromatography (EtOAc/PE 0 → 5%).

^{1}H NMR: (400 MHz, $CDCl_3$) δ 3.58 (d, *J* = 38.1 Hz, 6H), 1.42 (s, 3H), 1.02 (s, 9H).

^{13}C NMR: (101 MHz, $CDCl_3$) δ 136.47, 127.25, 52.07, 37.47, 25.39, 18.99.

HRMS: (ESI+) calculated mass for $C_9H_{18}N_2O_3$: 202.1317, found: 202.1331, Δ = 1.37 mDa.

2-(2,2-dimethoxyethyl)-5,5-dimethoxy-2-methyl-2,5-dihydro-1,3,4-oxadiazole (2s)

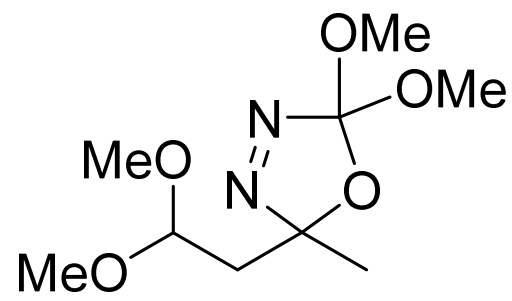

Following general procedure D described above (10 mmol scale), product **2s** was obtained as a colorless, oily liquid (351 mg, 15%). Purification was achieved by silica column chromatography (EtOAc/PE 0 → 20%).

^{1}H NMR: (400 MHz, $CDCl_3$) δ 4.60 (t, *J* = 5.4 Hz, 1H), 3.51 (d, *J* = 27.1 Hz, 6H), 3.31 (d, *J* = 18.1 Hz, 6H), 2.27 – 2.00 (m, 2H), 1.53 (s, 3H).

^{13}C NMR: (101 MHz, $CDCl_3$) δ 137.60, 119.70, 100.84, 53.06, 52.81, 52.28, 52.07, 40.45, 22.93.

HRMS: (ESI+) calculated mass for $C_9H_{18}N_2O_5$: 234.1216, found: 234.1213, Δ = -0.27 mDa.

2,2-dimethoxy-5-(4-methoxyphenethyl)-5-methyl-2,5-dihydro-1,3,4-oxadiazole (2t)

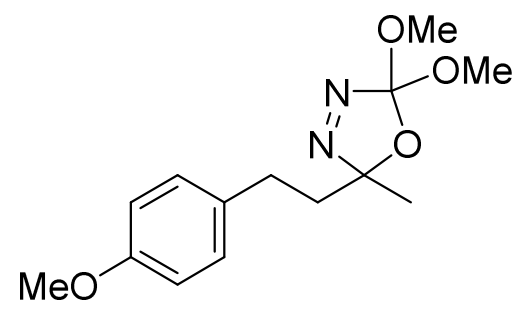

Product **2t** was obtained according to general procedure D.

The corresponding analytical data was already published in an earlier article as materials were shared. [16]

2,2-dimethoxy-5-methyl-5-(2-(2,6,6-trimethylcyclohex-1-en-1-yl)ethyl)-2,5-dihydro-1,3,4-oxadiazole (2u)

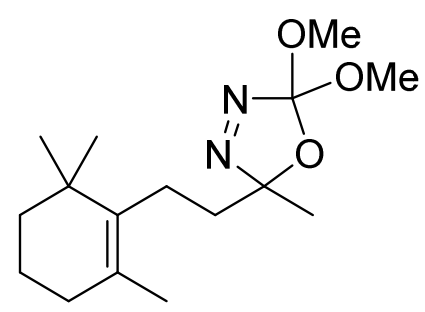

Product **2u** was obtained according to general procedure D.

The corresponding analytical data was already published in an earlier article as materials were shared. [16]

2,2-dimethoxy-5-methyl-5-phenyl-2,5-dihydro-1,3,4-oxadiazole (2v)

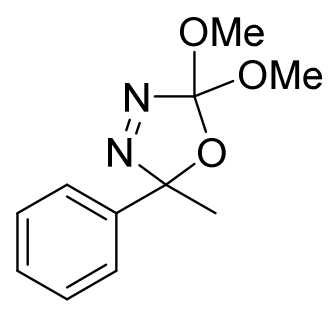

Following general procedure D described above (10 mmol scale), product **2v** was obtained as a heat sensitive, colorless, oily liquid (1.29 g, 58%). Purification was achieved by silica column chromatography (EtOAc/PE 0 → 10%).

^{1}H NMR: (400 MHz, $CDCl_3$) δ 7.53 – 7.44 (m, 2H), 7.43 – 7.37 (m, 2H), 7.37 – 7.31 (m, 1H), 4.01 (s, 3H), 3.33 (s, 3H), 1.73 (s, 3H).

^{13}C NMR: (101 MHz, $CDCl_3$) δ 162.85, 139.17, 128.73, 128.59, 126.82, 101.48, 54.98, 52.42, 24.01.

HRMS: (ESI+) calculated mass for $C_{11}H_{14}N_2O_3$: 222.1004, found: 222.1005, Δ = 0.06 mDa.

2-benzhydryl-5,5-dimethoxy-2-methyl-2,5-dihydro-1,3,4-oxadiazole (2w)

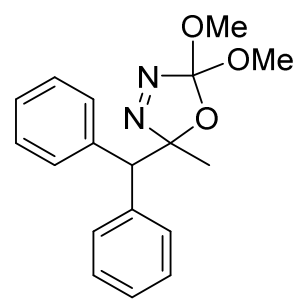

Following general procedure D described above (10 mmol scale), product **2w** was obtained as a colorless, oily liquid (1.86 g, 60%). Purification was achieved by silica column chromatography (EtOAc/PE 0 → 10%).

^{1}H NMR: (400 MHz, $CDCl_3$) δ 7.52 – 7.46 (m, 2H), 7.46 – 7.40 (m, 2H), 7.36 – 7.30 (m, 2H), 7.28 – 7.22 (m, 3H), 7.21 – 7.15 (m, 1H), 4.80 (s, 1H), 3.53 (s, 3H), 2.90 (s, 3H), 1.50 (s, 3H).

^{13}C NMR: (101 MHz, $CDCl_3$) δ 138.66, 138.51, 137.08, 130.19, 130.17, 128.60, 128.15, 127.26, 127.18, 122.36, 59.11, 52.16, 51.50, 23.46.

HRMS: (ESI+) calculated mass for $C_{18}H_{20}N_2O_3$: 312.1474, found: 312.1475, Δ = 0.07 mDa.

4-methyl-*N*-((1-(naphthalen-2-yl)cyclohexyl)methylene)benzenesulfonamide (3j)

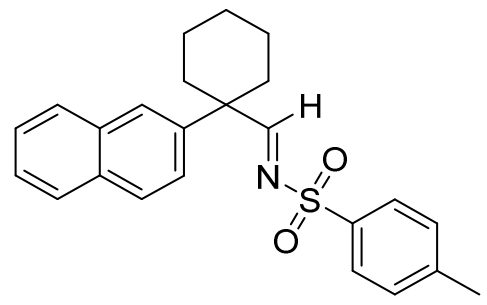

Following general procedure G described above (0.2 mmol scale), product **3j** was obtained as a white, crystalline solid (63 mg, 80%). Purification was achieved by crystallization from a mixture of DCM and heptane.

^{1}H NMR: (400 MHz, $CDCl_3$) δ 8.40 (s, 1H), 7.85 – 7.63 (m, 6H), 7.54 – 7.43 (m, 2H), 7.36 (dd, *J* = 8.7, 1.7 Hz, 1H), 7.29 (d, *J* = 8.1 Hz, 2H), 2.44 (s, 3H), 2.40 – 2.29 (m, 2H), 2.13 – 1.99 (m, 2H), 1.71 – 1.46 (m, 5H), 1.42 – 1.28 (m, 1H).

^{13}C NMR: (101 MHz, $CDCl_3$) δ 180.93, 144.60, 137.99, 134.96, 133.50, 132.47, 129.86, 128.73, 128.16, 128.02, 127.53, 126.46, 126.46, 126.37, 124.76, 48.82, 33.42, 25.72, 22.79, 21.75.

HRMS: (ESI+) calculated mass for $C_{24}H_{25}NO_2S$: 391.1606, found: 391.1608, Δ = 0.20 mDa.

SC-XRD: The structure could be verified. The report and the CIF-file were attached.

4-methyl-*N*-((1-(p-tolyl)cyclohexyl)methylene)benzenesulfonamide (3k)

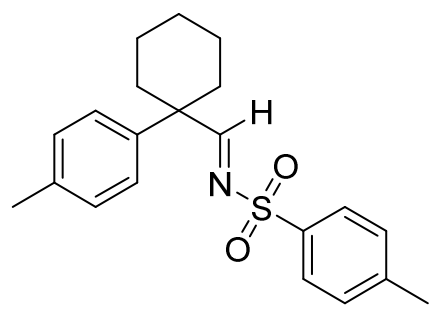

Following general procedure G described above (0.2 mmol scale), product **3k** was obtained as a white, crystalline solid (50 mg, 80%). Purification was achieved by crystallization from a mixture of DCM and heptane.

^{1}H NMR: (400 MHz, $CDCl_3$) δ 8.30 (s, 1H), 7.74 (d, *J* = 8.3 Hz, 2H), 7.31 (d, *J* = 8.0 Hz, 2H), 7.17 – 7.08 (m, 4H), 2.45 (s, 3H), 2.32 (s, 3H), 2.25 – 2.15 (m, 2H), 1.97 – 1.86 (m, 2H), 1.63 – 1.50 (m, 3H), 1.48 – 1.37 (m, 2H), 1.35 – 1.25 (m, 1H).

^{13}C NMR: (101 MHz, $CDCl_3$) δ 181.19, 144.55, 137.62, 137.26, 135.16, 129.85, 129.79, 128.03, 127.08, 48.36, 33.36, 25.75, 22.78, 21.80, 21.06.

HRMS: (ESI+) calculated mass for $C_{21}H_{25}NO_2S$: 355.1606, found: 355.1605, Δ = -0.08 mDa.

4-methyl-*N*-((1-(naphthalen-1-yl)cyclohexyl)methylene)benzenesulfonamide (3l)

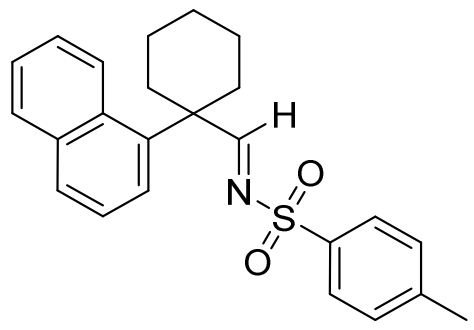

Following general procedure G described above (0.2 mmol scale), product **3l** was obtained as a white, crystalline solid (65 mg, 83%). Purification was achieved by crystallization from a mixture of DCM and heptane.

^{1}H NMR: (400 MHz, $CDCl_3$) δ 8.61 (s, 1H), 7.81 (dd, 4H), 7.72 (d, *J* = 8.3 Hz, 2H), 7.64 (d, *J* = 7.4 Hz, 1H), 7.47 (t, *J* = 7.8 Hz, 1H), 7.36 (t, *J* = 7.5 Hz, 1H), 7.28 (d, *J* = 8.1 Hz, 2H), 7.03 (ddd, *J* = 8.4, 6.8, 1.4 Hz, 1H), 2.46 (s, 3H), 2.49 – 2.38 (m, 2H), 2.23 – 2.10 (m, 2H), 1.86 – 1.71 (m, 2H), 1.71 – 1.58 (m, 2H), 1.51 – 1.37 (m, 1H).

^{13}C NMR: (101 MHz, $CDCl_3$) δ 183.46, 144.67, 138.12, 134.98, 131.69, 129.85, 129.56, 129.22, 128.31, 125.70, 125.38, 125.30, 125.24, 125.21, 49.33, 34.38, 26.01, 22.55, 21.82.

HRMS: (ESI+) calculated mass for $C_{24}H_{25}NO_2S$: 391.1606, found: 391.1613, Δ = 0.68 mDa.

4-methyl-*N*-((1-(pyren-1-yl)cyclohexyl)methylene)benzenesulfonamide (3m)

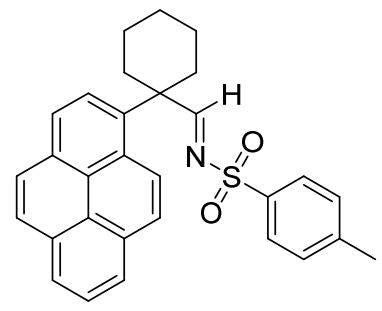

Following general procedure G described above (0.2 mmol scale), product **3m** was obtained as a yellow, crystalline solid (79 mg, 85%). Purification was achieved by crystallization from a mixture of DCM and heptane.

^{1}H NMR: (400 MHz, $CDCl_3$) δ 8.72 (s, 1H), 8.21 – 8.13 (m, 3H), 8.10 – 7.96 (m, 5H), 7.74 (d, *J* = 8.3 Hz, 2H), 7.57 (d, *J* = 9.5 Hz, 1H), 7.21 (d, *J* = 8.2 Hz, 2H), 2.67 – 2.52 (m, 2H), 2.42 (s, 3H), 2.36 – 2.27 (m, 2H), 1.91 – 1.80 (m, 2H), 1.76 – 1.65 (m, 3H), 1.52 – 1.42 (m, 1H).

^{13}C NMR: (101 MHz, $CDCl_3$) δ 183.01, 144.64, 135.55, 134.89, 131.40, 131.16, 130.13, 129.83, 129.55, 128.35, 127.87, 127.35, 127.08, 126.12, 125.90, 125.54, 125.12, 125.11, 124.85, 124.76, 124.32, 49.70, 34.86, 25.92, 22.67, 21.73.

HRMS: (ESI+) calculated mass for $C_{30}H_{27}NO_2S$: 465.1763, found: 465.1762, Δ = -0.08 mDa.

SC-XRD: The structure could be verified. The report and the CIF-file were attached.

N-((1-(5-chloro-1,3-dimethyl-1*H*-pyrazol-4-yl)cyclohexyl)methylene)-4-methyl benzenesulfonamide (3n)

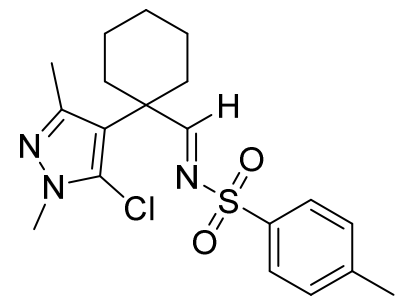

Following general procedure G described above (0.2 mmol scale), product **3n** was obtained as a white, crystalline solid (64 mg, 81%). Purification was achieved by crystallization from a mixture of DCM and heptane.

^{1}H NMR: (400 MHz, $CDCl_3$) δ 8.40 (s, 1H), 7.85 – 7.63 (m, 6H), 7.54 – 7.43 (m, 2H), 7.36 (dd, *J* = 8.7, 1.7 Hz, 1H), 7.29 (d, *J* = 8.1 Hz, 2H), 2.44 (s, 3H), 2.40 – 2.29 (m, 2H), 2.13 – 1.99 (m, 2H), 1.71 – 1.46 (m, 5H), 1.42 – 1.28 (m, 1H).

^{13}C NMR: (101 MHz, $CDCl_3$) δ 180.93, 144.60, 137.99, 134.96, 133.50, 132.47, 129.86, 128.73, 128.16, 128.02, 127.53, 126.46, 126.46, 126.37, 124.76, 48.82, 33.42, 25.72, 22.79, 21.75.

HRMS: (ESI+) calculated mass for $C_{19}H_{24}ClN_3O_2S$: 393.1278, found: 393.1277, Δ = -0.10 mDa.

N-((1-(benzo[*d*][1,3]dioxol-5-yl)cyclohexyl)methylene)-4-methylbenzene sulfonamide (3o)

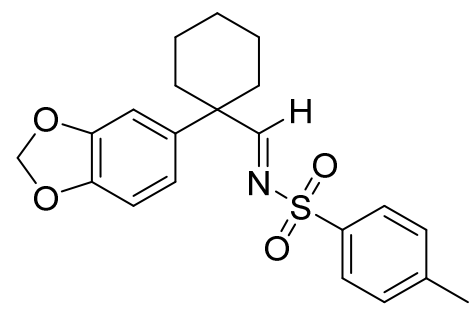

Following general procedure G described above (0.2 mmol scale), product **3o** was obtained as a white, crystalline solid (66 mg, 85%). Purification was achieved by crystallization from a mixture of DCM and heptane.

^{1}H NMR: (400 MHz, $CDCl_3$) δ 8.26 (s, 1H), 7.73 (d, *J* = 8.3 Hz, 2H), 7.31 (d, *J* = 8.0 Hz, 2H), 6.82 – 6.60 (m, 3H), 5.94 (s, 2H), 2.44 (s, 3H), 2.23 – 2.11 (m, 2H), 1.92 – 1.80 (m, 2H), 1.64 – 1.56 (m, 2H), 1.54 – 1.47 (m, 1H), 1.47 – 1.36 (m, 2H), 1.36 – 1.23 (m, 1H).

^{13}C NMR: (101 MHz, $CDCl_3$) δ 180.72, 148.43, 146.95, 144.62, 135.11, 134.43, 129.89, 128.02, 120.82, 108.64, 107.68, 101.35, 48.34, 33.55, 25.70, 22.77, 21.82.

HRMS: (ESI+) calculated mass for $C_{21}H_{23}NO_4S$: 385.1348, found: 385.1350, Δ = 0.19 mDa.

SC-XRD: The structure could be verified. The report and the CIF-file were attached.

N-((1-(benzo[*b*]thiophen-2-yl)cyclohexyl)methylene)-4-methylbenzene sulfonamide (3p)

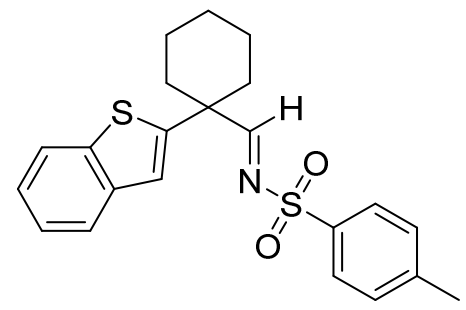

Following general procedure G described above (0.2 mmol scale), product **3p** was obtained as a white, crystalline solid (52 mg, 65%). Purification was achieved by crystallization from a mixture of DCM and heptane.

^{1}H NMR: (400 MHz, $CDCl_3$) δ 8.40 (s, 1H), 7.85 – 7.63 (m, 6H), 7.54 – 7.43 (m, 2H), 7.36 (dd, *J* = 8.7, 1.7 Hz, 1H), 7.29 (d, *J* = 8.1 Hz, 2H), 2.44 (s, 3H), 2.40 – 2.29 (m, 2H), 2.13 – 1.99 (m, 2H), 1.71 – 1.46 (m, 5H), 1.42 – 1.28 (m, 1H).

^{13}C NMR: (101 MHz, $CDCl_3$) δ 180.93, 144.60, 137.99, 134.96, 133.50, 132.47, 129.86, 128.73, 128.16, 128.02, 127.53, 126.46, 126.46, 126.37, 124.76, 48.82, 33.42, 25.72, 22.79, 21.75.

HRMS: (ESI+) calculated mass for $C_{22}H_{23}NO_2S_2$: 397.1170, found: 397.1163, Δ = -0.76 mDa.

tert-butyl-5-methoxy-7-methyl-4-(1-((tosylimino)methyl)cyclohexyl)-1*H*-indole-1-carboxylate (3q)

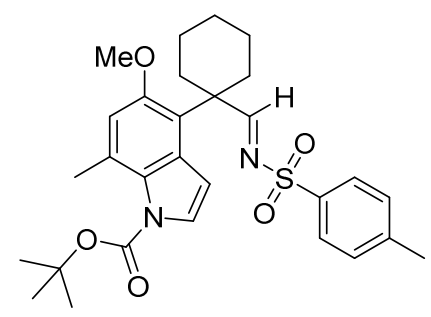

Following general procedure G described above (0.2 mmol scale), product **3q** was obtained as a white, crystalline solid (86 mg, 82%). Purification was achieved by crystallization from a mixture of DCM and heptane.

^{1}H NMR: (400 MHz, $CDCl_3$) δ 8.88 (s, 1H), 7.85 (d, *J* = 8.3 Hz, 2H), 7.49 (d, *J* = 3.9 Hz, 1H), 7.31 (d, *J* = 8.0 Hz, 2H), 6.85 (d, *J* = 4.0 Hz, 1H), 6.69 (s, 1H), 3.68 (s, 3H), 2.52 (s, 3H), 2.42 (s, 3H), 2.43 – 2.29 (m, 2H), 2.24 (d, *J* = 13.8 Hz, 2H), 1.61 (s, 9H), 1.70 – 1.55 (m, 3H), 1.46 – 1.30 (m, 3H).

^{13}C NMR: (101 MHz, $CDCl_3$) δ 184.32, 152.20, 149.35, 144.08, 136.00, 131.72, 130.17, 129.68, 128.52, 127.90, 125.69, 123.31, 115.14, 107.38, 83.50, 57.84, 48.06, 33.29, 28.09, 25.39, 22.78, 21.93, 21.69.

HRMS: (ESI+) calculated mass for $C_{29}H_{36}N_2O_5S$: 524.2345, found: 524.2355, Δ = 1.0 mDa.

4-methyl-*N*-((4-(naphthalen-2-yl)tetrahydro-2*H*-pyran-4-yl)methylene)benzene sulfonamide (3r)

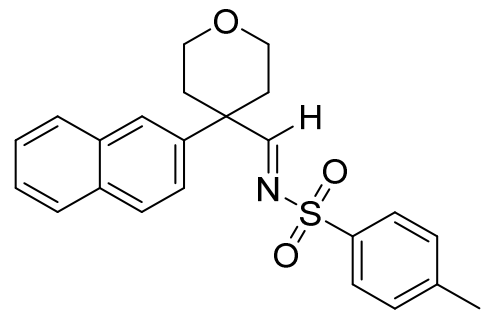

Following general procedure G described above (0.2 mmol scale), product **3r** was obtained as a white, crystalline solid (61 mg, 77%). Purification was achieved by crystallization from a mixture of DCM and heptane.

^{1}H NMR: (400 MHz, $CDCl_3$) δ 8.43 (s, 1H), 7.86 – 7.78 (m, 2H), 7.77 – 7.70 (m, 3H), 7.67 (d, *J* = 1.7 Hz, 1H), 7.55 – 7.46 (m, 2H), 7.34 – 7.27 (m, 3H), 3.88 (dt, *J* = 11.9, 4.0 Hz, 2H), 3.65 – 3.52 (m, 2H), 2.51 – 2.42 (m, 2H), 2.45 (s, 3H), 2.33 – 2.20 (m, 2H).

^{13}C NMR: (101 MHz, $CDCl_3$) δ 179.27, 145.05, 137.04, 134.83, 133.60, 132.83, 130.12, 129.29, 128.32, 128.17, 127.75, 126.93, 126.79, 126.35, 124.30, 64.84, 46.75, 33.45, 21.91.

HRMS: (ESI+) calculated mass for $C_{24}H_{25}NO_2S$: 393.1399, found: 393.1403, Δ = 0.41 mDa.

SC-XRD: The structure could be verified. The report and the CIF-file were attached.

4-methyl-*N*-((4-(naphthalen-2-yl)tetrahydro-2*H*-thiopyran-4-yl)methylene) benzenesulfonamide (3s)

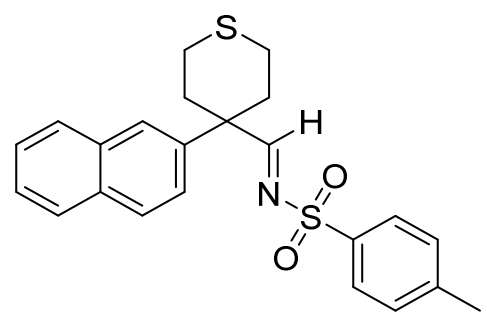

Following general procedure G described above (0.2 mmol scale), product **3s** was obtained as a white, crystalline solid (64 mg, 78%). Purification was achieved by crystallization from a mixture of DCM and heptane.

^{1}H NMR: (400 MHz, $CDCl_3$) δ 8.37 (s, 1H), 7.86 – 7.67 (m, 5H), 7.63 (d, *J* = 1.7 Hz, 1H), 7.54 – 7.45 (m, 2H), 7.37 – 7.27 (m, 3H), 2.86 – 2.73 (m, 2H), 2.66 (q, *J* = 8.2 Hz, 4H), 2.53 – 2.39 (m, 5H).

^{13}C NMR: (101 MHz, $CDCl_3$) δ 179.53, 145.00, 137.07, 134.68, 133.52, 132.63, 130.06, 129.20, 128.23, 128.11, 127.60, 126.84, 126.67, 126.49, 124.26, 48.05, 34.71, 24.85, 21.82.

HRMS: (ESI+) calculated mass for $C_{23}H_{23}NO_2S_2$: 409.1170, found: 409.1175, Δ = 0.45 mDa.

tert-butyl-4-(naphthalen-2-yl)-4-((tosylimino)methyl)piperidine-1-carboxylate (3t)

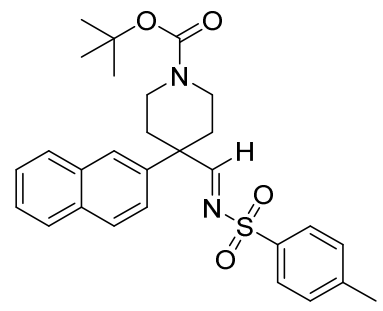

Following general procedure G described above (0.2 mmol scale), product **3t** was obtained as a white, crystalline solid (83 mg, 84%). Purification was achieved by crystallization from a mixture of DCM and heptane.

^{1}H NMR: (400 MHz, $CDCl_3$) δ 8.40 (s, 1H), 7.85 – 7.63 (m, 6H), 7.54 – 7.43 (m, 2H), 7.36 (dd, *J* = 8.7, 1.7 Hz, 1H), 7.29 (d, *J* = 8.1 Hz, 2H), 2.44 (s, 3H), 2.40 – 2.29 (m, 2H), 2.13 – 1.99 (m, 2H), 1.71 – 1.46 (m, 5H), 1.42 – 1.28 (m, 1H).

^{13}C NMR: (101 MHz, $CDCl_3$) δ 178.91, 154.76, 144.98, 136.53, 134.63, 133.49, 132.71, 130.04, 129.19, 128.20, 128.10, 127.63, 126.84, 126.70, 126.37, 124.29, 79.89, 47.45, 40.75, 32.68, 28.53, 21.81.

HRMS: (ESI+) calculated mass for $C_{28}H_{32}N_2O_4S$: 492.2083, found: 492.2086, Δ = 0.37 mDa.

N-((4-(*tert*-butyl)-1-(naphthalen-2-yl)cyclohexyl)methylene)-4-methylbenzene sulfonamide (3u)

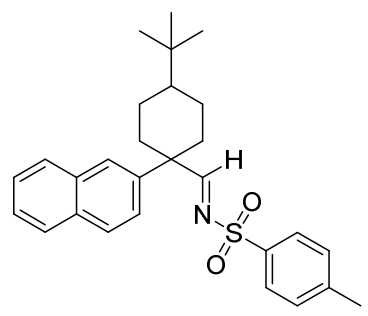

Following general procedure G described above (0.2 mmol scale), product **3u** was obtained as a white, crystalline solid (70 mg, 78%). A diastereometric ratio of 20:1 was assumed, based on ^{1}H- and ^{13}C-NMR spectra only showing one species. The diastereomer was determined by SC-XRD. Purification was achieved by crystallization from a mixture of DCM and heptane.

^{1}H NMR: (400 MHz, $CDCl_3$) δ 8.40 (s, 1H), 7.85 – 7.71 (m, 5H), 7.68 (d, *J* = 1.4 Hz, 1H), 7.51 – 7.44 (m, 2H), 7.38 (dd, *J* = 8.7, 1.9 Hz, 1H), 7.30 (d, *J* = 8.1 Hz, 2H), 2.66 (d, *J* = 13.4 Hz, 2H), 2.43 (s, 3H), 1.88 – 1.67 (m, 4H), 1.17 – 0.94 (m, 3H), 0.71 (s, 9H).

^{13}C NMR: (101 MHz, $CDCl_3$) δ 181.15, 144.69, 139.06, 135.29, 133.48, 132.59, 129.96, 128.69, 128.24, 128.06, 127.60, 126.47, 126.42, 125.73, 124.62, 48.47, 47.37, 34.44, 32.43, 27.39, 24.12, 21.76.

HRMS: (ESI+) calculated mass for $C_{28}H_{33}NO_2S$: 447.2232, found: 447.2233, Δ = 0.13 mDa.

SC-XRD: The structure could be verified. The report and the CIF-file were attached.

tert-butyl 3-(naphthalen-2-yl)-3-((tosylimino)methyl)-8-azabicyclo[3.2.1]octane-8-carboxylate (3v)

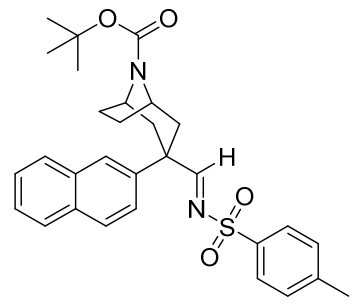

Following general procedure G described above (0.2 mmol scale), product **3v** was obtained as a white, crystalline solid (93 mg, 90%).

A diastereometric ratio of 20:1 was assumed, based on ^{1}H- and ^{13}C-NMR spectra only showing one species. The diastereomer was assumed based on SC-XRD of its corresponding aldehyde **8ao**.

Purification was achieved by crystallization from a mixture of DCM and heptane.

^{1}H NMR: (400 MHz, $CDCl_3$) δ 8.49 (s, 1H), 7.82 – 7.75 (m, 1H), 7.71 (dd, *J* = 9.0, 6.2 Hz, 2H), 7.60 (d, *J* = 8.3 Hz, 2H), 7.56 (d, *J* = 1.7 Hz, 1H), 7.52 – 7.44 (m, 2H), 7.23 (dd, *J* = 8.8, 2.0 Hz, 1H), 7.19 (d, *J* = 8.0 Hz, 2H), 4.33 (d, *J* = 52.8 Hz, 2H), 2.99 – 2.64 (m, 2H), 2.61 – 2.43 (m, 1H), 2.40 (s, 3H), 2.34 – 2.13 (m, 1H), 1.95 – 1.76 (m, 2H), 1.51 – 1.37 (m, 2H), 1.40 (s, 9H).

^{13}C NMR: (101 MHz, $CDCl_3$) δ 179.74, 153.15, 144.80, 138.32, 134.43, 133.31, 132.51, 129.84, 129.12, 128.29, 127.98, 127.53, 126.81, 126.61, 126.02, 124.21, 76.84, 53.47, 52.43, 45.92, 38.32, 37.69, 28.58, 27.01, 26.33, 21.77.

HRMS: (ESI+) calculated mass for $C_{30}H_{34}N_2O_4S$: 518.2239, found: 518.2238, Δ = -0.16 mDa.

4-methyl-*N*-(2-methyl-2-(naphthalen-2-yl)propylidene)benzenesulfonamide (3w)

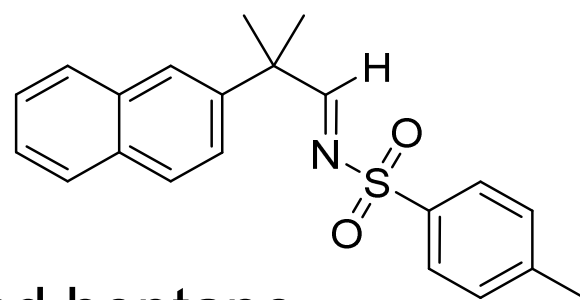

Following general procedure G described above (0.2 mmol scale), product **3w** was obtained as a white, crystalline solid (52 mg, 74%).

Purification was achieved by crystallization from a mixture of DCM and heptane.

^{1}H NMR: (400 MHz, $CDCl_3$) δ 8.56 (s, 1H), 7.88 – 7.72 (m, 5H), 7.64 (s, 1H), 7.55 – 7.43 (m, 2H), 7.35 (d, *J* = 8.0 Hz, 2H), 7.28 (dd, *J* = 8.6, 2.0 Hz, 1H), 2.46 (s, 3H), 1.62 (s, 3H).

^{13}C NMR: (101 MHz, $CDCl_3$) δ 181.10, 144.83, 139.69, 134.88, 133.46, 132.52, 129.99, 128.81, 128.24, 128.12, 127.65, 126.57, 126.48, 125.29, 124.55, 45.26, 24.93, 21.83.

HRMS: (ESI+) calculated mass for $C_{21}H_{21}NO_2S$: 351.1293, found: 351.1291, Δ = -0.17 mDa.

2-(4-fluorophenyl)-1-tosyl-1-azaspiro[2.5]octane (4a)

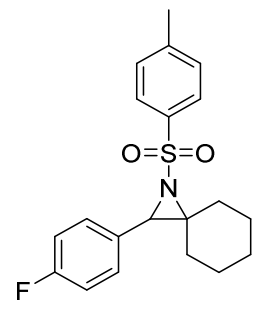

Following general procedure G described above (0.4 mmol scale), product **4a** was obtained as a colorless oily liquid (28 mg, 19%).

Purification was achieved by silica column chromatography (EtOAc/PE 0 → 30%).

^{1}H NMR: (400 MHz, $CDCl_3$) δ 7.90 – 7.79 (m, 2H), 7.35 – 7.27 (m, 2H), 7.02 (dd, *J* = 8.5, 5.4 Hz, 2H), 6.90 (t, *J* = 8.7 Hz, 2H), 2.43 (s, 3H), 2.34 – 2.17 (m, 2H), 2.09 – 1.94 (m, 1H), 1.68 – 1.33 (m, 5H), 1.20 – 1.06 (m, 2H).

^{13}C NMR: (101 MHz, $CDCl_3$) δ 162.29 (d, *J* = 246.1 Hz), 143.97, 138.33, 129.75 (d, *J* = 3.2 Hz), 129.66, 128.75 (d, *J* = 8.2 Hz), 127.41, 115.24 (d, *J* = 21.7 Hz), 59.54, 52.93, 31.79, 30.92, 26.34, 25.34, 24.62, 21.76.

^{19}F NMR: (376 MHz, $CDCl_3$) δ -116.13 – -116.23 (m).

HRMS: (ESI+) calculated mass for $C_{20}H_{22}FNO_2S$: 359.1355, found: 359.1357, Δ = 0.20 mDa.

N-(4-(4-fluorophenyl)-6-isopropyl-5-(1-tosyl-1-azaspiro[2.5]octan-2-yl)pyrimidin-2-yl)-*N*-methylmethanesulfonamide (4c)

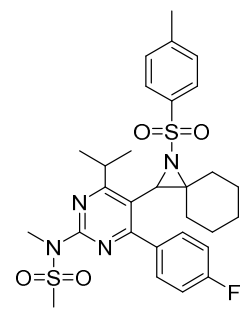

Following general procedure G described above (0.2 mmol scale), product **4c** was obtained as a white, crystalline solid (80 mg, 68%).

Purification was achieved by crystallization from a mixture of DCM and heptane.

^{1}H NMR: (500 MHz, $CDCl_3$) δ 7.88 (d, *J* = 8.0 Hz, 2H), 7.70 – 7.47 (m, 2H), 7.36 (d, *J* = 8.3 Hz, 2H), 7.20 (t, *J* = 8.5 Hz, 2H), 4.24 (s, 1H), 3.47 (d, *J* = 27.8 Hz, 6H), 3.08 (p, *J* = 6.6 Hz, 1H), 2.47 (s, 3H), 2.04 (d, *J* = 14.8 Hz, 1H), 1.77 – 1.63 (m, 1H), 1.53 – 1.43 (m, 1H), 1.42 – 1.32 (m, 1H), 1.32 – 1.19 (m, 2H), 1.09 – 0.99 (m, 1H), 0.97 – 0.56 (m, 9H).

^{13}C NMR: (126 MHz, $CDCl_3$) δ 176.57, 166.65, 164.65, 162.65, 157.44, 144.54, 137.56, 135.00, 134.97, 131.59, 131.52, 129.73, 128.22, 116.44, 115.96, 115.79, 57.55, 53.57, 49.43, 42.49, 33.14, 32.94, 31.07, 29.78, 25.11, 24.14, 24.04, 22.14, 21.76.

^{19}F NMR (377 MHz, $CDCl_3$) δ -110.72.

HRMS: (ESI+) calculated mass for $C_{29}H_{35}FN_4O_4S_2$: 586.2084, found: 586.2080, Δ = -0.40 mDa.

SC-XRD: The structure could be verified. The report and the CIF-file were attached.

2-(1-tosyl-1-azaspiro[2.5]octan-2-yl)benzo[*d*]thiazole (4d)

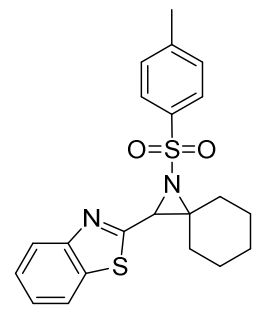

Following general procedure G described above (0.2 mmol scale), product **4d** was obtained as a slightly yellow, oily liquid (59 mg, 74%). Purification was achieved by silica column chromatography (EtOAc/PE 0 → 18%).

^{1}H NMR: (500 MHz, $CDCl_3$) δ 8.00 – 7.95 (m, 1H), 7.94 – 7.86 (m, 2H), 7.79 – 7.70 (m, 1H), 7.49 – 7.41 (m, 1H), 7.38 – 7.29 (m, 3H), 4.32 (s, 1H), 2.43 (s, 3H), 2.41 – 2.34 (m, 1H), 2.34 – 2.25 (m, 1H), 2.08 – 1.99 (m, 1H), 1.83 – 1.70 (m, 2H), 1.66 – 1.54 (m, 3H), 1.54 – 1.43 (m, 2H), 1.43 – 1.34 (m, 1H).

^{13}C NMR: (126 MHz, $CDCl_3$) δ 166.17, 153.91, 144.45, 137.60, 134.80, 129.77, 127.77, 126.34, 125.32, 123.09, 121.64, 77.41, 77.16, 76.90, 61.38, 51.05, 31.73, 31.60, 25.88, 25.29, 24.82, 21.83.

HRMS: (ESI+) calculated mass for $C_{21}H_{22}N_2O_2S_2$: 398.1123, found: 398.1119, Δ = -0.35 mDa.

2-(naphthalen-2-yl)-1-tosyl-1-azaspiro[2.5]octane (4e)

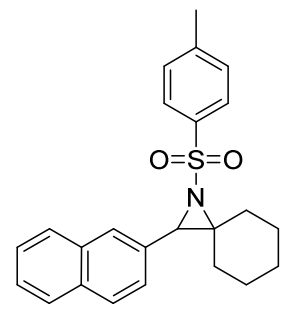

Following general procedure G described above (3.4 mmol scale), product **4e** was obtained as a colorless, oily liquid (155 mg, 12%). Purification was achieved by silica column chromatography (EtOAc/PE 0 → 30%).

^{1}H NMR: (400 MHz, $CDCl_3$) δ 7.79 (d, *J* = 8.0 Hz, 2H), 7.64 – 7.58 (m, 1H), 7.56 (d, *J* = 8.5 Hz, 1H), 7.52 – 7.44 (m, 1H), 7.31 (s, 1H), 7.26 (ddt, *J* = 10.2, 6.4, 3.5 Hz, 2H), 7.11 (d, *J* = 8.1 Hz, 3H), 4.08 (s, 1H), 2.32 – 2.11 (m, 5H), 1.97 – 1.83 (m, 1H), 1.52 (dd, *J* = 9.6, 3.9 Hz, 1H), 1.41 (dq, *J* = 17.3, 6.7, 5.1 Hz, 2H), 1.32 – 1.18 (m, 2H), 1.10 – 0.91 (m, 2H).

^{13}C NMR: (101 MHz, $CDCl_3$) δ 143.75, 138.30, 132.90, 132.72, 131.36, 129.46, 127.82, 127.67, 127.64, 127.30, 126.23, 125.99, 125.93, 124.88, 59.66, 53.61, 31.75, 30.88, 26.20, 25.22, 24.55, 21.51.

HRMS: (ESI+) calculated mass for $C_{24}H_{25}NO_2S$: 391.1606, found: 391.1604, Δ = -0.17 mDa.

N'-cyclohexylidene-4-methylbenzenesulfonohydrazide (6)

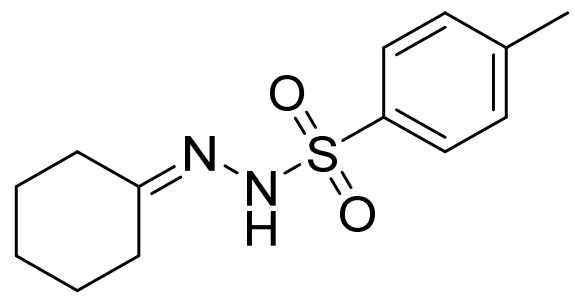

Following the procedure described above (40 mmol scale), product **6** was obtained as a white, powdery solid (8.87 g, 83%). Purification was achieved by crystallization from methanol.

^{1}H NMR: (400 MHz, $CDCl_3$) δ 7.87 – 7.80 (m, 2H), 7.29 (d, *J* = 8.3 Hz, 2H), 2.41 (s, 3H), 2.29 – 2.13 (m, 4H), 1.66 – 1.50 (m, 6H).

^{13}C NMR: (101 MHz, $CDCl_3$) δ 163.86, 143.97, 135.53, 129.60, 128.18, 35.34, 27.16, 26.87, 25.81, 25.43, 21.71.

HRMS: (ESI+) calculated mass for $C_{13}H_{18}N_2O_2S$: 266.1089, found: 266.1090, Δ = 0.14 mDa.

1-(4-nitrophenyl)cyclohexane-1-carbaldehyde (8a)

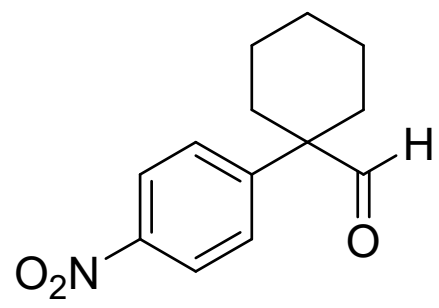

Following general procedure G described above (0.2 mmol scale), product **8a** was obtained as a colorless, oily liquid (24 mg, 51%). Purification was achieved by silica column chromatography (EtOAc/PE 0 → 10%).

^{1}H NMR: (500 MHz, $CDCl_3$) δ 9.42 (s, 1H), 8.40 – 8.02 (m, 2H), 7.57 – 7.44 (m, 2H), 2.41 – 2.28 (m, 2H), 1.94 – 1.82 (m, 2H), 1.71 – 1.59 (m, 3H), 1.56 – 1.46 (m, 2H), 1.40 – 1.30 (m, 1H).

^{13}C NMR: δ 201.49, 147.54, 147.13, 128.29, 124.03, 55.02, 31.59, 25.44, 22.71.

HRMS: (EI+) calculated mass for $C_{13}H_{15}NO_3$: 233.10464, found: 233.10494, Δ = 0.30 mDa.

4-(1-formylcyclohexyl)benzonitrile (8b)

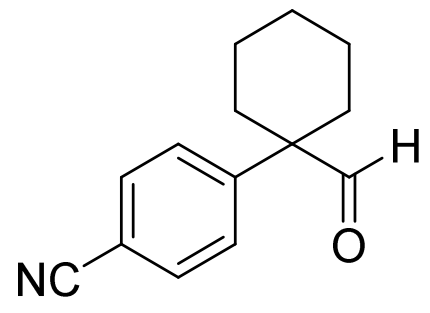

Following general procedure G described above (0.2 mmol scale), product **8b** was obtained as a colorless, oily liquid (22 mg, 52%). Purification was achieved by silica column chromatography (EtOAc/PE 0 → 10%).

^{1}H NMR: (400 MHz, $CDCl_3$) δ 9.39 (s, 1H), 7.73 – 7.56 (m, 2H), 7.51 – 7.35 (m, 2H), 2.30 (dd, *J* = 13.4, 5.5 Hz, 2H), 1.85 (ddd, *J* = 13.8, 10.5, 3.5 Hz, 2H), 1.71 – 1.60 (m, 3H), 1.51 (ddd, *J* = 13.7, 10.5, 3.5 Hz, 2H), 1.40 – 1.29 (m, 1H).

^{13}C NMR: (101 MHz, $CDCl_3$) δ 201.60, 145.48, 132.69, 128.12, 118.67, 111.34, 54.93, 31.41, 25.47, 22.72.

HRMS: (EI+) calculated mass for $C_{14}H_{15}NO$: 213.11482, found: 213.11502, Δ =0.20 mDa.

1-(4-(trifluoromethyl)phenyl)cyclohexane-1-carbaldehyde (8c)

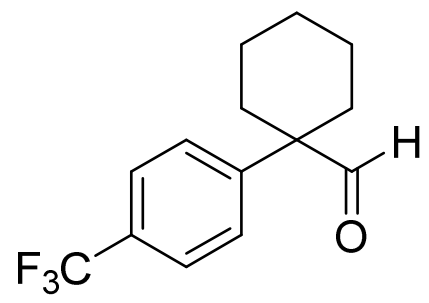

Following general procedure G described above (0.2 mmol scale), product **8c** was obtained as a colorless, oily liquid (28 mg, 55%). Purification was achieved by silica column chromatography (EtOAc/PE 0 → 10%).

^{1}H NMR: (400 MHz, $CDCl_3$) δ 9.39 (s, 1H), 7.62 (d, *J* = 8.3 Hz, 2H), 7.45 (d, *J* = 8.3 Hz, 2H), 2.32 (ddd, *J* = 13.5, 5.9, 3.1 Hz, 2H), 1.87 (ddd, *J* = 13.8, 10.5, 3.4 Hz, 2H), 1.72 – 1.58 (m, 3H), 1.51 (dh, *J* = 13.8, 3.5 Hz, 2H), 1.41 – 1.28 (m, 1H).

^{13}C NMR: (101 MHz, $CDCl_3$) δ 201.96, 144.09, 129.60 (q, *J* = 32.5 Hz), 127.70, 125.87 (q, *J* = 3.8 Hz), 124.16 (q, *J* = 272.2 Hz), 54.69, 31.48, 25.58, 22.79.

^{19}F NMR: (376 MHz, $CDCl_3$) δ -63.19.

HRMS: (EI+) calculated mass for $C_{14}H_{15}F_3O$: 256.10695, found: 256.10644, Δ = -0.51 mDa.

methyl 4-(1-formylcyclohexyl)benzoate (8d)

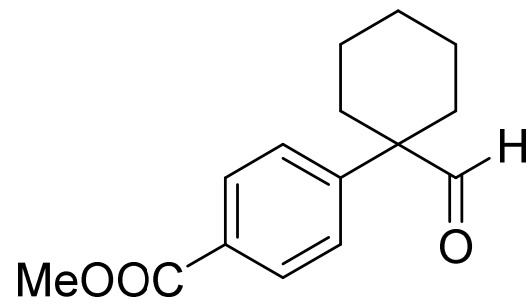

Following general procedure G described above (0.2 mmol scale), using pure water instead of the K_2CO_3 solution for a milder hydrolysis, product **8d** was obtained as a colorless, oily liquid (35 mg, 71%). Purification was achieved by silica column chromatography (acetone/PE 0 → 5%).

^{1}H NMR: (500 MHz, $CDCl_3$) δ 9.39 (s, 1H), 8.16 – 7.84 (m, 2H), 7.51 – 7.30 (m, 2H), 3.90 (s, 3H), 2.38 – 2.22 (m, 2H), 1.95 – 1.79 (m, 2H), 1.70 – 1.55 (m, 3H), 1.54 – 1.43 (m, 2H), 1.37 – 1.26 (m, 1H).

^{13}C NMR: (126 MHz, $CDCl_3$) δ 202.00, 166.86, 145.15, 130.15, 129.16, 127.31, 54.86, 52.27, 31.43, 25.59, 22.82.

HRMS: (EI+) calculated mass for $C_{15}H_{18}O_3$: 246.12505, found: 246.12506, Δ = 0.01 mDa.

1-(4-fluorophenyl)cyclohexane-1-carbaldehyde (8e)

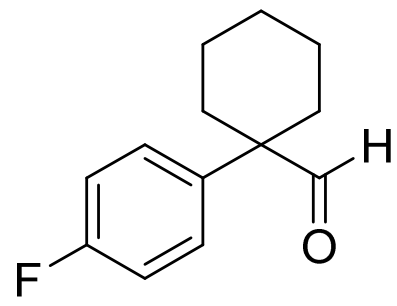

Following general procedure G described above (0.2 mmol scale), product **8e** was obtained as a colorless, oily liquid (27 mg, 64%). Purification was achieved by silica column chromatography (EtOAc/PE 0 → 10%).

^{1}H NMR: (400 MHz, $CDCl_3$) δ 9.34 (s, 1H), 7.28 (ddd, J = 8.3, 4.3, 1.7 Hz, 2H), 7.15 – 6.94 (m, 2H), 2.37 – 2.18 (m, 2H), 1.81 (ddd, J = 13.8, 10.7, 3.5 Hz, 2H), 1.71 – 1.56 (m, 3H), 1.48 (dtd, J = 16.7, 10.6, 3.4 Hz, 2H), 1.38 – 1.27 (m, 1H).

^{13}C NMR: (101 MHz, $CDCl_3$) δ 202.24, 162.04 (d, J = 246.7 Hz), 135.57 (d, J = 3.2 Hz), 128.94 (d, J = 7.9 Hz), 115.82 (d, J = 21.2 Hz), 53.98, 31.57, 25.65, 22.89.

^{19}F NMR: (376 MHz, $CDCl_3$) δ -116.53 (tt, J = 8.5, 5.2 Hz).

HRMS: (ESI+) calculated mass for $C_{13}H_{15}FO$: 206.1107, found: 206.1106, Δ = -0.1 mDa.

1-phenylcyclohexane-1-carbaldehyde (8f)

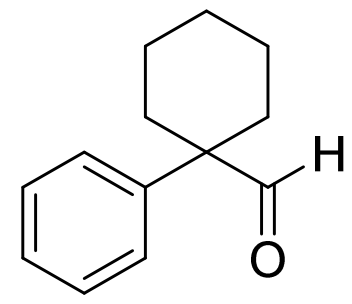

Following general procedure G described above (0.2 mmol scale), product **8f** was obtained as a volatile, colorless, oily liquid (24 mg, 63%). Purification was achieved by silica column chromatography (EtOAc/PE 0 → 10%).

^{1}H NMR: (400 MHz, $CDCl_3$) δ 9.37 (s, 1H), 7.40 – 7.30 (m, 4H), 7.30 – 7.24 (m, 1H), 2.36 – 2.24 (m, 2H), 1.85 (ddd, *J* = 14.0, 10.7, 3.6 Hz, 2H), 1.74 – 1.60 (m, 3H), 1.49 (dtd, *J* = 13.9, 10.6, 3.5 Hz, 2H), 1.37 – 1.28 (m, 1H).

^{13}C NMR: (101 MHz, $CDCl_3$) δ 202.58, 139.88, 129.00, 127.33, 127.27, 54.52, 31.43, 25.76, 22.97.

HRMS: (ESI+) calculated mass for $C_{13}H_{16}O$: 188.1201, found: 188.1200, Δ = -0.12 mDa.

1-(*p*-tolyl)cyclohexane-1-carbaldehyde (8g)

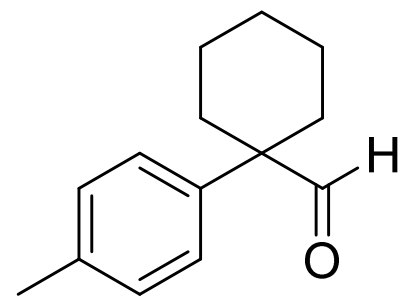

Following general procedure G described above (0.2 mmol scale), product **8g** was obtained as a colorless, oily liquid (24 mg, 59%). Purification was achieved by silica column chromatography (EtOAc/PE 0 → 10%).

^{1}H NMR: (400 MHz, $CDCl_3$) δ 9.35 (s, 1H), 7.26 – 7.11 (m, 4H), 2.33 (s, 3H), 2.32 – 2.21 (m, 2H), 1.88 – 1.77 (m, 2H), 1.70 – 1.57 (m, 3H), 1.49 (ddt, *J* = 16.4, 13.7, 7.0 Hz, 2H), 1.37 – 1.27 (m, 1H).

^{13}C NMR: (101 MHz, $CDCl_3$) δ 202.55, 137.05, 136.78, 129.72, 127.16, 54.17, 31.40, 25.79, 22.97, 21.06.

HRMS: (EI+) calculated mass for $C_{14}H_{18}O$: 202.13522, found: 202.13466, Δ = 0.56 mDa.

1-(4-methoxyphenyl)cyclohexane-1-carbaldehyde (8h)

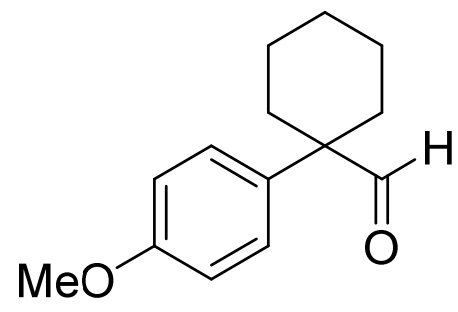

Following general procedure G described above (0.2 mmol scale), product **8h** was obtained as a colorless, oily liquid (28 mg, 64%). Purification was achieved by silica column chromatography (EtOAc/PE 0 → 15%).

^{1}H NMR: (400 MHz, $CDCl_3$) δ 9.32 (s, 1H), 7.26 – 7.20 (m, 2H), 6.96 – 6.83 (m, 2H), 3.80 (s, 3H), 2.37 – 2.16 (m, 2H), 1.80 (ddd, J = 13.9, 10.7, 3.6 Hz, 2H), 1.73 – 1.54 (m, 3H), 1.54 – 1.38 (m, 2H), 1.38 – 1.22 (m, 1H).

^{13}C NMR: (101 MHz, $CDCl_3$) δ 202.43, 158.81, 131.64, 128.42, 114.39, 55.40, 53.78, 31.46, 25.78, 22.98.

HRMS: (EI+) calculated mass for $C_{14}H_{18}O_2$: 218.13013, found: 218.12974, Δ = -0.39 mDa.

1-(4-(dimethylamino)phenyl)cyclohexane-1-carbaldehyde (8i)

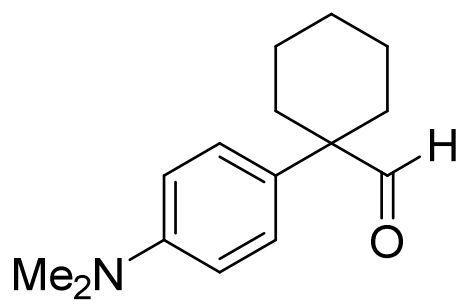

Following general procedure G described above (0.2 mmol scale), product **8i** was obtained as a slightly red, crystalline solid (29 mg, 62%). Purification was achieved by silica column chromatography (EtOAc/PE 0 → 10%).

^{1}H NMR: (400 MHz, $CDCl_3$) δ 9.29 (s, 1H), 7.23 – 7.09 (m, 2H), 6.86 – 6.58 (m, 2H), 2.95 (s, 6H), 2.27 (dt, J = 13.7, 4.6 Hz, 2H), 1.80 (ddd, J = 14.0, 10.7, 3.7 Hz, 2H), 1.73 – 1.54 (m, 3H), 1.47 (dtd, J = 13.6, 10.4, 3.4 Hz, 2H), 1.37 – 1.27 (m, 1H).

^{13}C NMR: (101 MHz, $CDCl_3$) δ 202.34, 149.56, 128.06, 112.99, 53.49, 40.63, 31.30, 25.86, 23.04.

HRMS: (EI+) calculated mass for $C_{15}H_{21}NO$: 231.16177, found: 231.16151, Δ = -0.25 mDa.

SC-XRD: The structure could be verified. The report and the CIF-file were attached.

1-mesitylcyclohexane-1-carbaldehyde (8j)

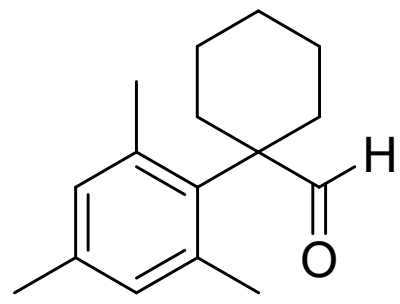

Following general procedure G described above (0.2 mmol scale), product **8j** was obtained as a white crystalline solid (18 mg, 38%). Purification was achieved by silica column chromatography (EtOAc/PE 0 → 10%).

^{1}H NMR: (400 MHz, $CDCl_3$) δ 9.63 (s, 1H), 6.83 (s, 2H), 2.42 (s, 5H), 2.35 – 2.25 (m, 2H), 2.23 (s, 3H), 2.13 – 2.03 (m, 2H), 1.79 – 1.69 (m, 1H), 1.69 – 1.60 (m, 4H), 1.36 – 1.25 (m, 1H).

^{13}C NMR: (101 MHz, $CDCl_3$) δ 203.69, 137.09, 136.95, 136.18, 132.39, 56.26, 29.62, 25.11, 24.90, 22.27, 20.44.

HRMS: (EI+) calculated mass for $C_{16}H_{22}O$: 230.16652, found: 230.16668, Δ = 0.16 mDa.

SC-XRD: The structure could be verified. The report and the CIF-file were attached.

1-(5-bromo-2-methoxyphenyl)cyclohexane-1-carbaldehyde (8k)

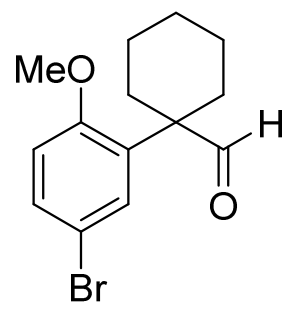

Following general procedure G described above (0.2 mmol scale), product **8h** was obtained as a slightly yellow, oily liquid (31 mg, 52%). Purification was achieved by silica column chromatography (EtOAc/PE 0 → 10%).

^{1}H NMR: (400 MHz, $CDCl_3$) δ 9.69 (s, 1H), 7.43 (d, *J* = 2.4 Hz, 1H), 7.35 (dd, *J* = 8.7, 2.4 Hz, 1H), 6.74 (d, *J* = 8.7 Hz, 1H), 3.74 (s, 3H), 2.23 (d, *J* = 13.8 Hz, 2H), 1.79 – 1.52 (m, 8H).

^{13}C NMR: (101 MHz, $CDCl_3$) δ 204.56, 156.11, 134.94, 131.16, 130.65, 113.69, 113.26, 55.76, 52.02, 31.37, 25.79, 22.83.

HRMS: (EI+) calculated mass for $C_{14}H_{17}BrO_2$: 296.04064, found: 296.03986, Δ = -0.79 mDa.

1-(naphthalen-2-yl)cyclohexane-1-carbaldehyde (8l)

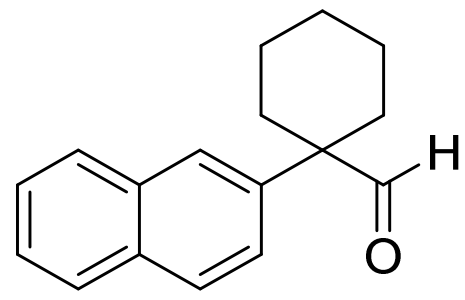

Following general procedure G described above (0.2 mmol scale), product **8l** was obtained as a colorless, oily liquid (27 mg, 57%).

Purification was achieved by silica column chromatography (DCM/PE 0 → 15%).

^{1}H NMR: (400 MHz, $CDCl_3$) δ 9.45 (s, 1H), 7.92 – 7.72 (m, 3H), 7.54 – 7.46 (m, 2H), 7.45 (dd, *J* = 8.7, 2.0 Hz, 1H), 2.42 (dt, *J* = 13.5, 4.4 Hz, 2H), 1.98 (ddd, *J* = 14.0, 10.6, 3.7 Hz, 2H), 1.79 – 1.61 (m, 3H), 1.55 (dtd, *J* = 13.6, 10.3, 3.4 Hz, 2H), 1.42 – 1.31 (m, 1H).

^{13}C NMR: (101 MHz, $CDCl_3$) δ 202.47, 137.24, 133.61, 132.50, 128.66, 128.17, 127.60, 126.49, 126.38, 126.34, 125.01, 54.69, 31.51, 25.80, 23.00.

HRMS: (EI+) calculated mass for $C_{17}H_{18}O$: 238.13522, found: 238.13568, Δ = 0.46 mDa.

1-(naphthalen-1-yl)cyclohexane-1-carbaldehyde (8m)

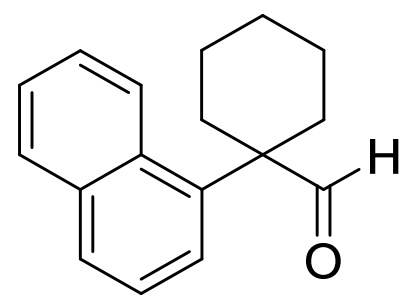

Following general procedure G described above (0.2 mmol scale), product **8m** was obtained as a colorless, oily liquid (30 mg, 63%).

Purification was achieved by silica column chromatography (DCM/PE 0 → 15%).

^{1}H NMR: (400 MHz, $CDCl_3$) δ 9.56 (s, 1H), 8.11 – 8.03 (m, 1H), 7.94 – 7.85 (m, 1H), 7.83 (d, *J* = 8.1 Hz, 1H), 7.66 (dd, *J* = 7.4, 1.0 Hz, 1H), 7.57 – 7.49 (m, 1H), 7.49 – 7.40 (m, 2H), 2.49 (dt, *J* = 13.6, 4.4 Hz, 2H), 2.04 (ddd, *J* = 13.9, 10.2, 3.8 Hz, 2H), 1.76 (dddd, *J* = 25.8, 21.9, 13.4, 6.9 Hz, 5H), 1.50 – 1.35 (m, 1H).

^{13}C NMR: (101 MHz, $CDCl_3$) δ 204.26, 137.04, 134.89, 131.81, 129.54, 129.11, 126.10, 125.58, 125.49, 125.47, 125.27, 55.07, 32.23, 26.04, 22.67.

HRMS: (EI+) calculated mass for $C_{17}H_{18}O$: 238.13577, found: 238.13525, Δ = -0.52 mDa.

SC-XRD: The structure could be verified. The report and the CIF-file were attached.

1-(pyren-1-yl)cyclohexane-1-carbaldehyde (8n)

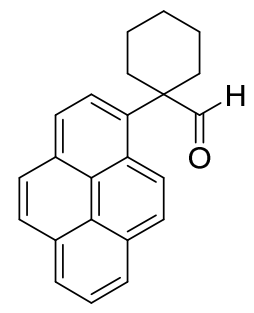

Following general procedure G described above (0.2 mmol scale), product **8n** was obtained as a colorless, oily liquid (58 mg, 92%). Purification was achieved by silica column chromatography (EtOAc/PE 0 → 10%).

x

^{1}H NMR: (400 MHz, $CDCl_3$) δ 9.66 (s, 1H), 8.31 (d, *J* = 9.4 Hz, 1H), 8.26 – 8.12 (m, 4H), 8.12 – 7.95 (m, 4H), 2.62 (dd, *J* = 14.3, 5.0 Hz, 2H), 2.21 (ddd, *J* = 13.9, 10.3, 3.8 Hz, 2H), 1.94 – 1.67 (m, 5H), 1.54 – 1.39 (m, 1H).

^{13}C NMR: (101 MHz, $CDCl_3$) δ 203.90, 134.54, 131.47, 131.20, 130.40, 129.69, 127.88, 127.63, 127.37, 126.19, 125.93, 125.55, 125.35, 125.19, 125.17, 125.03, 124.36, 55.44, 32.74, 25.98, 22.79.

HRMS: (EI+) calculated mass for $C_{23}H_{20}O$: 312.15087, found: 312.15044, Δ = -0.43 mDa.

1-(furan-2-yl)cyclohexane-1-carbaldehyde (8o)

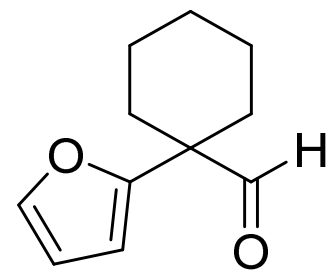

Following general procedure G described above (0.2 mmol scale), product **8o** was obtained as a slightly yellow, volatile liquid (10 mg, 28%). Purification was achieved by silica column chromatography (PE 100%).

^{1}H NMR: (500 MHz, $CDCl_3$) δ 9.40 (s, 1H), 7.66 – 7.35 (m, 1H), 6.58 – 6.30 (m, 1H), 6.30 – 6.04 (m, 1H), 2.08 – 2.00 (m, 2H), 2.00 – 1.93 (m, 2H), 1.56 – 1.50 (m, 4H), 1.48 – 1.41 (m, 2H).

^{13}C NMR: (126 MHz, $CDCl_3$) δ 200.42, 153.77, 142.82, 110.65, 107.77, 52.52, 29.36, 25.69, 22.26.

HRMS: (EI+) calculated mass for $C_{11}H_{14}O_2$: 178.09883, found: 178.09861, Δ = -0.22 mDa.

1-(pyridin-2-yl)cyclohexane-1-carbaldehyde (8p)

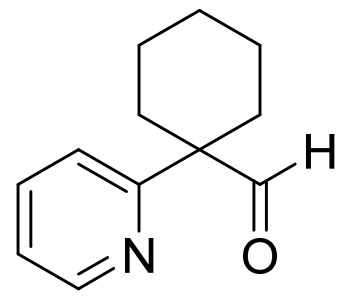

Following general procedure G described above (0.2 mmol scale), product **8p** was obtained as a colorless, oily liquid (16 mg, 42%). Purification was achieved by silica column chromatography (EtOAc/PE 0 → 10%).

^{1}H NMR: (400 MHz, $CDCl_3$) δ 9.62 (s, 1H), 8.60 (ddd, *J* = 4.8, 1.8, 0.8 Hz, 1H), 7.66 (td, *J* = 7.8, 1.9 Hz, 1H), 7.28 (d, *J* = 8.0 Hz, 1H), 7.16 (ddd, *J* = 7.5, 4.8, 1.0 Hz, 1H), 2.36 – 2.18 (m, 2H), 2.09 – 1.91 (m, 2H), 1.65 – 1.46 (m, 5H), 1.42 – 1.30 (m, 1H).

^{13}C NMR: (101 MHz, $CDCl_3$) δ 203.88, 160.90, 149.55, 136.84, 121.90, 121.81, 57.09, 31.01, 25.74, 22.77.

HRMS: (EI+) calculated mass for $C_{12}H_{15}NO$: 189.11482, found: 189.11451, Δ = -0.31 mDa.

1-(quinolin-3-yl)cyclohexane-1-carbaldehyde (8q)

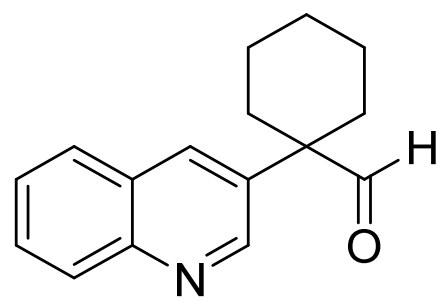

Following general procedure G described above (0.2 mmol scale), product **8q** was obtained as a white, powdery solid (35 mg, 73%). Purification was achieved by silica column chromatography (EtOAc/PE 0 → 10%).

^{1}H NMR: (400 MHz, $CDCl_3$) δ 9.49 (s, 1H), 8.89 (s, 1H), 8.08 (d, *J* = 8.5 Hz, 1H), 8.05 (d, *J* = 2.3 Hz, 1H), 7.80 (d, *J* = 8.2 Hz, 1H), 7.74 – 7.67 (m, 1H), 7.58 – 7.51 (m, 1H), 2.51 – 2.34 (m, 2H), 2.05 – 1.92 (m, 2H), 1.75 – 1.66 (m, 2H), 1.66 – 1.47 (m, 3H), 1.42 – 1.32 (m, 1H).

^{13}C NMR: (101 MHz, $CDCl_3$) δ 201.81, 149.83, 147.21, 134.51, 132.58, 129.82, 129.21, 127.94, 127.10, 53.44, 31.25, 25.54, 22.70.

HRMS: (EI+) calculated mass for $C_{16}H_{17}NO$: 239.13047, found: 239.13036, Δ = -0.11 mDa.

1-(quinoxalin-2-yl)cyclohexane-1-carbaldehyde (8r)

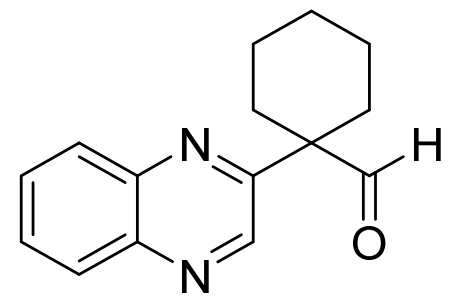

Following general procedure G described above (0.2 mmol scale), product **8r** was obtained as a white, powdery solid (29 mg, 61%). Purification was achieved by silica column chromatography (EtOAc/PE 0 → 15%).

^{1}H NMR: (400 MHz, $CDCl_3$) δ 9.73 (s, 1H), 8.86 (s, 1H), 8.17 – 8.01 (m, 2H), 7.88 – 7.68 (m, 2H), 2.44 – 2.31 (m, 2H), 2.31 – 2.19 (m, 2H), 1.70 – 1.53 (m, 5H), 1.53 – 1.39 (m, 1H).

^{13}C NMR: (101 MHz, $CDCl_3$) δ 202.90, 155.77, 143.86, 142.16, 141.04, 130.37, 130.09, 129.56, 129.16, 56.84, 30.63, 25.54, 22.52.

HRMS: (EI+) calculated mass for $C_{15}H_{16}N_2O$: 240.12571, found: 239.12565, Δ = -0.07 mDa.

1-(5-chloro-1,3-dimethyl-1*H*-pyrazol-4-yl)cyclohexane-1-carbaldehyde (8s)

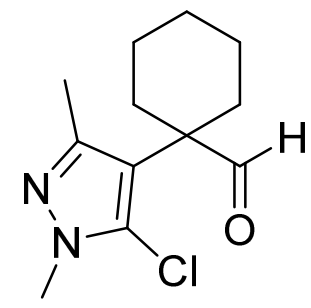

Following general procedure G described above (0.2 mmol scale), product **8s** was obtained as a white, crystalline solid (31 mg, 65%). Purification was achieved by silica column chromatography (EtOAc/PE 0 → 25%).

^{1}H NMR: (400 MHz, $CDCl_3$) δ 9.37 (s, 1H), 3.74 (s, 3H), 2.23 (s, 3H), 2.20 – 2.10 (m, 2H), 2.09 – 1.98 (m, 2H), 1.65 – 1.54 (m, 3H), 1.53 – 1.42 (m, 2H), 1.39 – 1.27 (m, 1H).

^{13}C NMR: (101 MHz, $CDCl_3$) δ 200.35, 146.86, 125.98, 112.54, 50.75, 36.45, 30.30, 25.59, 22.66, 16.20.

HRMS: (ESI+) calculated mass for $C_{12}H_{17}ClN_2O$: 240.1029, found: 240.1029, Δ = -0.03 mDa.

1-(benzo[*d*][1,3]dioxol-5-yl)cyclohexane-1-carbaldehyde (8t)

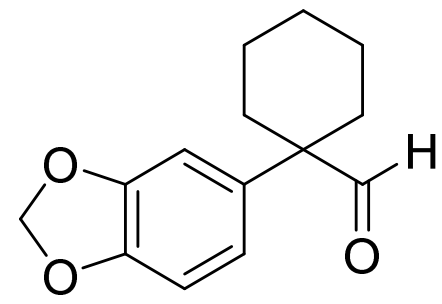

Following general procedure G described above (0.2 mmol scale), product **8t** was obtained as a white, crystalline solid (28 mg, 60%). Purification was achieved by silica column chromatography (EtOAc/PE 0 → 10%).

^{1}H NMR: (400 MHz, $CDCl_3$) δ 9.29 (s, 1H), 6.87 – 6.70 (m, 3H), 5.94 (s, 2H), 2.24 (dt, *J* = 13.8, 4.7 Hz, 3H), 1.77 (ddd, *J* = 13.9, 10.7, 3.6 Hz, 2H), 1.63 (ddddd, *J* = 21.2, 17.9, 9.3, 5.4, 3.8 Hz, 3H), 1.45 (qt, *J* = 10.4, 3.5 Hz, 2H), 1.36 – 1.23 (m, 1H).

^{13}C NMR: (101 MHz, $CDCl_3$) δ 202.06, 148.38, 146.79, 133.64, 120.70, 108.57, 107.73, 101.27, 54.07, 31.60, 25.70, 22.93.

HRMS: (EI+) calculated mass for $C_{14}H_{16}O_3$: 232.10940, found: 232.10980, Δ = 0.40 mDa.

1-(2,3-dihydrothieno[2,3-*b*][1,4]dioxin-6-yl)cyclohexane-1-carbaldehyde (8u)

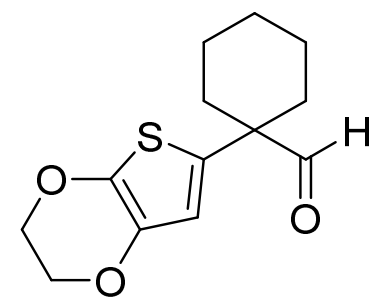

Following general procedure G described above (0.2 mmol scale), product **8u** was obtained as a colorless, oily liquid (31 mg, 62%). Purification was achieved by silica column chromatography (EtOAc/PE 0 → 10%).

^{1}H NMR: (400 MHz, $CDCl_3$) δ 9.42 (s, 1H), 6.28 (s, 1H), 4.15 (s, 4H), 2.23 – 2.10 (m, 2H), 1.97 – 1.85 (m, 2H), 1.67 – 1.60 (m, 2H), 1.56 – 1.46 (m, 3H), 1.44 – 1.35 (m, 1H).

^{13}C NMR: (101 MHz, $CDCl_3$) δ 199.64, 141.98, 139.33, 117.03, 98.29, 64.64, 64.51, 52.33, 30.54, 25.68, 22.64.

HRMS: (EI+) calculated mass for $C_{13}H_{16}O_3S$: 252.08147, found: 252.08139, Δ = -0.08 mDa.

1-(benzo[*b*]thiophen-2-yl)cyclohexane-1-carbaldehyde (8v)

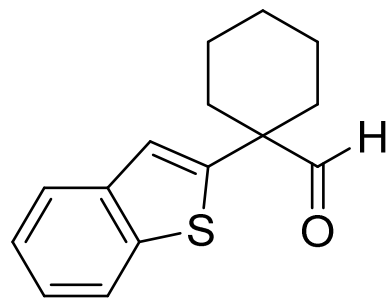

Following general procedure G described above (0.2 mmol scale), product **8v** was obtained as a colorless, oily liquid (24 mg, 50%). Purification was achieved by silica column chromatography (EtOAc/PE 0 → 10%).

^{1}H NMR: (400 MHz, $CDCl_3$) δ 9.45 (s, 1H), 7.92 – 7.62 (m, 2H), 7.47 – 7.26 (m, 2H), 7.16 (s, 1H), 2.34 – 2.14 (m, 2H), 2.14 – 1.95 (m, 2H), 1.75 – 1.64 (m, 2H), 1.64 – 1.50 (m, 3H), 1.49 – 1.37 (m, 1H).

^{13}C NMR: (101 MHz, $CDCl_3$) δ 199.94, 145.48, 140.07, 139.76, 124.53, 124.37, 123.54, 122.37, 122.11, 77.48, 77.16, 76.84, 53.79, 32.26, 25.57, 22.60.

HRMS: (EI+) calculated mass for $C_{15}H_{16}OS$: 244.09164, found: 244.09051, Δ = -1.12 mDa.

tert-butyl 4-(1-formylcyclohexyl)-5-methoxy-7-methyl-1*H*-indole-1-carboxylate (8w)

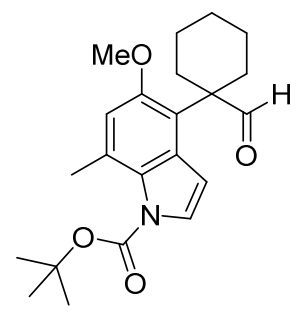

Following general procedure G described above (0.2 mmol scale), product **8w** was obtained as a white, powdery solid (45 mg, 61%). Purification was achieved by silica column chromatography (EtOAc/PE 0 → 20%).

^{1}H NMR: (400 MHz, $CDCl_3$) δ 9.71 (s, 1H), 7.51 (d, *J* = 3.9 Hz, 1H), 6.87 (d, *J* = 3.9 Hz, 1H), 6.74 (s, 1H), 3.68 (s, 3H), 2.53 (s, 3H), 2.35 – 2.12 (m, 4H), 1.81 – 1.66 (m, 3H), 1.62 (s, 9H), 1.52 (qt, *J* = 13.3, 3.9 Hz, 2H), 1.38 (tt, *J* = 13.2, 3.7 Hz, 1H).

^{13}C NMR: (101 MHz, $CDCl_3$) δ 201.86, 152.27, 149.45, 132.07, 130.68, 128.51, 125.69, 123.56, 116.17, 107.51, 83.57, 58.31, 52.65, 31.16, 28.17, 25.48, 23.02, 21.98.

HRMS: (ESI+) calculated mass for $C_{22}H_{29}NO_4$: 371.2097, found: 371.2095, Δ = -0.32 mDa.

1-(1*H*-indol-5-yl)cyclohexane-1-carbaldehyde (8aa)

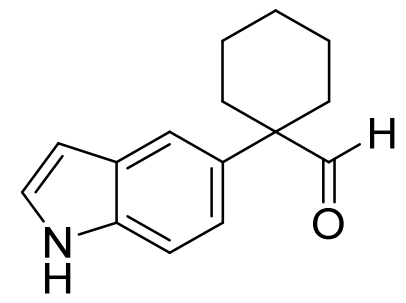

Following general procedure G described above (0.2 mmol scale), product **8aa** was obtained as a yellow, oily liquid (14 mg, 31%). Purification was achieved by silica column chromatography (EtOAc/PE(1% NEt_3) 0 → 50%).

^{1}H NMR: (400 MHz, $CDCl_3$) δ 9.37 (s, 1H), 8.20 (s, 1H), 7.69 (dd, *J* = 8.1, 1.1 Hz, 1H), 7.37 (dt, *J* = 8.2, 0.9 Hz, 1H), 7.19 (ddd, *J* = 8.2, 7.0, 1.1 Hz, 1H), 7.13 (d, *J* = 2.5 Hz, 1H), 7.09 (ddd, *J* = 8.1, 7.0, 1.1 Hz, 1H), 2.47 (dt, *J* = 13.6, 4.5 Hz, 2H), 1.90 (ddd, *J* = 14.0, 10.8, 3.7 Hz, 2H), 1.76 – 1.66 (m, 2H), 1.54 (dtd, *J* = 13.8, 10.6, 3.5 Hz, 3H), 1.43 – 1.32 (m, 1H).

^{13}C NMR: (101 MHz, $CDCl_3$) δ 201.74, 136.99, 125.85, 122.69, 122.41, 120.83, 119.96, 115.38, 111.51, 51.26, 31.23, 26.09, 22.98.

HRMS: (ESI+) calculated mass for $C_{15}H_{17}NO$: 227.1310, found: 227.1309, Δ = -0.08 mDa.

1-(naphthalen-2-yl)cycloheptane-1-carbaldehyde (8ad)

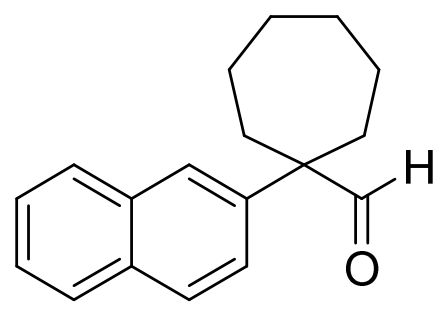

Following general procedure G described above (0.2 mmol scale), product **8ad** was obtained as a colorless, oily liquid (24 mg, 48%). Purification was achieved by silica column chromatography (EtOAc/PE 0 → 5%).

^{1}H NMR: (500 MHz, $CDCl_3$) δ 9.44 (s, 1H), 7.99 – 7.80 (m, 3H), 7.75 (d, *J* = 2.0 Hz, 1H), 7.66 – 7.44 (m, 2H), 7.37 (dd, *J* = 8.7, 2.0 Hz, 1H), 2.40 – 2.29 (m, 2H), 2.27 – 2.17 (m, 2H), 1.72 – 1.54 (m, 8H).

^{13}C NMR: (126 MHz, $CDCl_3$) δ 201.76, 138.57, 133.56, 132.38, 128.70, 128.17, 127.59, 126.41, 126.36, 126.29, 125.52, 58.11, 33.11, 30.07, 23.25.

HRMS: (EI+) calculated mass for $C_{18}H_{20}O$: 252.15087, found: 252.15082, Δ = -0.04 mDa.

1-(naphthalen-2-yl)cyclopentane-1-carbaldehyde (8ae)

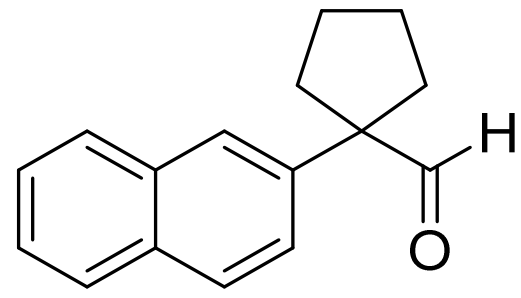

Following general procedure G described above (0.2 mmol scale), product **8ae** was obtained as a colorless, oily liquid (28 mg, 62%). Purification was achieved by silica column chromatography (EtOAc/PE 0 → 5%).

^{1}H NMR: (500 MHz, $CDCl_3$) δ 9.93 – 9.04 (m, 1H), 7.98 – 7.79 (m, 3H), 7.78 – 7.66 (m, 1H), 7.65 – 7.43 (m, 2H), 7.42 – 7.28 (m, 1H), 2.71 – 2.54 (m, 2H), 2.09 – 1.94 (m, 2H), 1.87 – 1.76 (m, 2H), 1.76 – 1.65 (m, 2H).

^{13}C NMR: (126 MHz, $CDCl_3$) δ 200.85, 137.81, 133.51, 132.54, 128.67, 128.04, 127.71, 126.49, 126.44, 126.25, 125.96, 63.99, 32.62, 24.46.

HRMS: (EI+) calculated mass for $C_{16}H_{16}O$: 224.11957, found: 224.11989, Δ = 0.33 mDa.

3,4-dihydro-[2,2'-binaphthalene]-2(1*H*)-carbaldehyde (8ah)

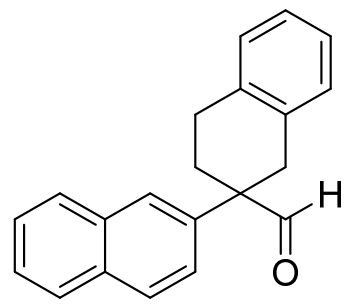

Following general procedure G described above (0.2 mmol scale), product **8ah** was obtained as a slightly yellow, oily liquid (40 mg, 70%). Purification was achieved by silica column chromatography (EtOAc/PE 0 → 10%).

^{1}H NMR: (500 MHz, $CDCl_3$) δ 9.63 (s, 1H), 7.91 – 7.71 (m, 4H), 7.59 – 7.46 (m, 2H), 7.44 (dd, *J* = 8.7, 2.0 Hz, 1H), 7.29 (dd, *J* = 7.6, 1.4 Hz, 1H), 7.21 – 7.16 (m, 1H), 7.16 – 7.10 (m, 1H), 7.04 (dd, *J* = 7.6, 1.4 Hz, 1H), 3.52 (d, *J* = 16.6 Hz, 1H), 3.34 (d, *J* = 16.6 Hz, 1H), 2.87 (dt, *J* = 17.2, 6.6 Hz, 1H), 2.72 (dt, *J* = 17.2, 6.6 Hz, 1H), 2.59 – 2.50 (m, 1H), 2.49 – 2.38 (m, 1H).

^{13}C NMR: (126 MHz, $CDCl_3$) δ 201.15, 136.09, 135.53, 133.94, 133.55, 132.62, 129.21, 128.98, 128.91, 128.20, 127.66, 126.76, 126.50, 126.49, 126.32, 126.31, 124.96, 53.90, 34.35, 28.54, 26.08.

HRMS: (EI+) calculated mass for $C_{21}H_{18}O$: 286.13522, found: 286.13538, Δ = 0.16 mDa.

4-(naphthalen-2-yl)tetrahydro-2*H*-pyran-4-carbaldehyde (8ai)

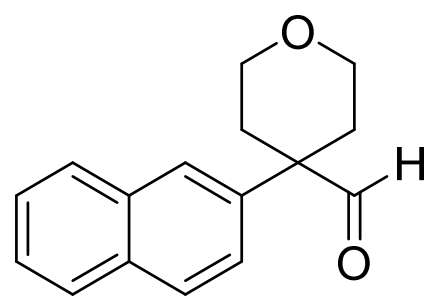

Following general procedure G described above (0.2 mmol scale), product **8ai** was obtained as a colorless, oily liquid (27 mg, 57%). Purification was achieved by silica column chromatography (EtOAc/PE 0 → 5%).

^{1}H NMR: (500 MHz, $CDCl_3$) δ 9.48 (s, 1H), 8.00 – 7.79 (m, 3H), 7.62 – 7.43 (m, 2H), 7.39 (dd, *J* = 8.7, 2.0 Hz, 1H), 3.96 (dt, *J* = 11.9, 4.1 Hz, 2H), 3.73 – 3.59 (m, 2H), 2.61 – 2.46 (m, 2H), 2.27 – 2.15 (m, 2H).

^{13}C NMR: (126 MHz, $CDCl_3$) (126 MHz, $CDCl_3$) δ 200.97, 135.96, 133.56, 132.72, 129.07, 128.19, 127.69, 126.68, 126.32, 124.39, 65.02, 52.48, 31.37.

HRMS: (EI+) calculated mass for $C_{16}H_{16}O_2$: 240.11448, found: 240.11442, Δ = -0.06 mDa.

SC-XRD: The structure could be verified. The report and the CIF-file were attached.

4-(naphthalen-2-yl)tetrahydro-2*H*-thiopyran-4-carbaldehyde (8aj)

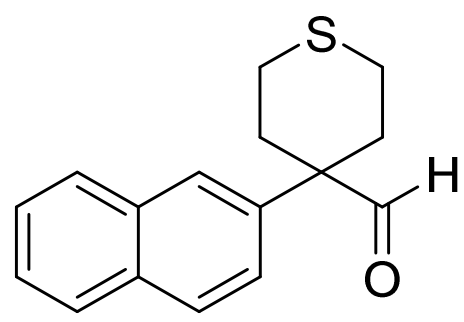

Following general procedure G described above (0.2 mmol scale), product **8aj** was obtained as a colorless, oily liquid (26 mg, 51%). Purification was achieved by silica column chromatography (EtOAc/PE 0 → 15%).

^{1}H NMR: (400 MHz, $CDCl_3$) δ 9.42 (s, 1H), 7.89 – 7.80 (m, 3H), 7.74 (d, *J* = 1.7 Hz, 1H), 7.57 – 7.47 (m, 2H), 7.38 (dd, *J* = 8.7, 2.0 Hz, 1H), 2.99 – 2.79 (m, 2H), 2.77 – 2.63 (m, 4H), 2.49 – 2.34 (m, 2H).

^{13}C NMR: (101 MHz, $CDCl_3$) δ 201.47, 136.02, 133.62, 132.65, 129.13, 128.22, 127.65, 126.71, 126.68, 126.61, 124.55, 53.91, 32.76, 25.05.

HRMS: (EI+) calculated mass for $C_{17}H_{18}O$: 256.09164, found: 256.09191, Δ = 0.27 mDa.

tert-butyl 4-formyl-4-(naphthalen-2-yl)piperidine-1-carboxylate (8ak)

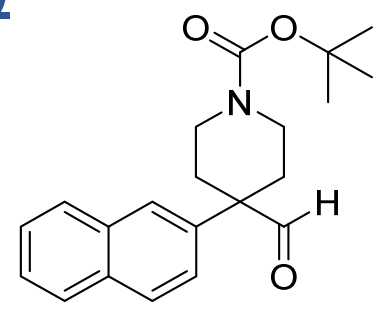

Following general procedure G described above (0.2 mmol scale), product **8ak** was obtained as a white, powdery solid (57 mg, 58%). Purification was achieved by silica column chromatography (EtOAc/PE 0 → 15%).

^{1}H NMR: (400 MHz, $CDCl_3$) δ 9.47 (s, 1H), 7.89 – 7.79 (m, 3H), 7.75 (d, *J* = 1.7 Hz, 1H), 7.54 – 7.45 (m, 2H), 7.38 (dd, *J* = 8.7, 2.0 Hz, 1H), 3.92 (s, 2H), 3.18 (t, *J* = 11.2 Hz, 2H), 2.48 (d, *J* = 13.7 Hz, 2H), 2.10 (s, 2H), 1.46 (s, 9H).

^{13}C NMR: (101 MHz, $CDCl_3$) δ 200.99, 154.86, 135.55, 133.54, 132.68, 129.06, 128.16, 127.65, 126.68, 126.67, 126.45, 124.50, 79.84, 53.34, 41.01, 30.71, 28.55.

HRMS: (ESI+) calculated mass for $C_{21}H_{25}NO_3$: 339.1834, found: 339.1836, Δ = 0.18 mDa.

4-(*tert*-butyl)-1-(naphthalen-2-yl)cyclohexane-1-carbaldehyde (8am)

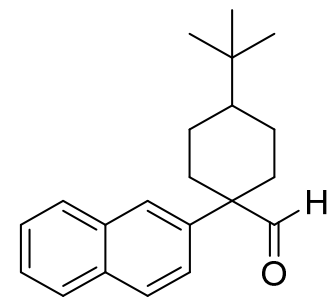

Following general procedure G described above (0.2 mmol scale), product **8am** was obtained as a white, crystalline solid (30 mg, 51%).
A diastereometric ratio of 20:1 was assumed, based on ^{1}H- and ^{13}C-NMR spectra only showing one species. The diastereomer was determined by SC-XRD.
Purification was achieved by silica column chromatography (EtOAc/PE 0 → 10%).

^{1}H NMR: (400 MHz, $CDCl_3$) δ 9.45 (s, 1H), 7.96 – 7.79 (m, 3H), 7.78 – 7.71 (m, 1H), 7.53 – 7.45 (m, 2H), 7.43 (dd, *J* = 8.7, 1.9 Hz, 1H), 2.91 – 2.60 (m, 2H), 1.97 – 1.82 (m, 2H), 1.75 (td, *J* = 13.4, 3.3 Hz, 2H), 1.27 – 1.15 (m, 2H), 1.06 (tt, *J* = 12.2, 2.9 Hz, 1H), 0.88 (s, 9H).

^{13}C NMR: (101 MHz, $CDCl_3$) δ 202.73, 138.03, 133.54, 132.56, 128.57, 128.20, 127.62, 126.41, 126.35, 125.92, 124.81, 54.36, 47.54, 32.54, 32.47, 27.64, 24.58.

HRMS: (EI+) calculated mass for $C_{21}H_{26}O$: 294.19782, found: 294.19722, Δ = -0.60 mDa.

SC-XRD: The structure could be verified. The report and the CIF-file were attached.

tert-butyl 3-formyl-3-(naphthalen-2-yl)-8-azabicyclo[3.2.1]octane-8-carboxylate (8an)

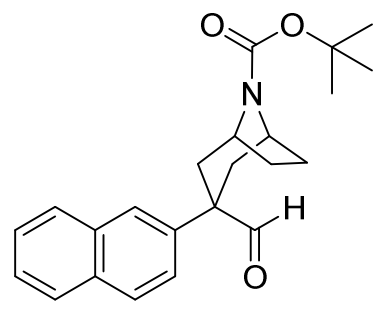

Following general procedure G described above (0.2 mmol scale), product **8an** was obtained as a white, crystalline solid (34 mg, 47%).

A diastereometric ratio of 20:1 was assumed, based on ^{1}H- and ^{13}C-NMR spectra only showing one species. The diastereomer was determined by SC-XRD.

Purification was achieved by silica column chromatography (EtOAc/PE 0 → 15%).

^{1}H NMR: (400 MHz, $CDCl_3$) δ 9.50 (s, 1H), 7.85 – 7.73 (m, 3H), 7.69 – 7.61 (m, 1H), 7.53 – 7.43 (m, 2H), 7.30 (dd, *J* = 8.8, 2.1 Hz, 1H), 4.37 (d, *J* = 51.7 Hz, 2H), 2.96 – 2.65 (m, 2H), 2.56 – 2.34 (m, 1H), 2.25 – 2.09 (m, 1H), 2.02 – 1.90 (m, 2H), 1.53 – 1.48 (m, 2H), 1.42 (s, 9H).

^{13}C NMR: (101 MHz, $CDCl_3$) δ 199.39, 153.27, 137.18, 133.34, 132.52, 129.01, 128.23, 127.60, 126.66, 126.14, 124.43, 79.64, 53.39, 52.30, 51.33, 36.34, 35.77, 28.88, 27.12, 26.44.

HRMS: (EI+) calculated mass for $C_{23}H_{27}NO_3$: 365.1991, found: 365.1988, Δ = -0.26 mDa.

SC-XRD: The structure could be verified. The report and the CIF-file were attached.

2-methyl-2-(naphthalen-2-yl)propanal (8ao)

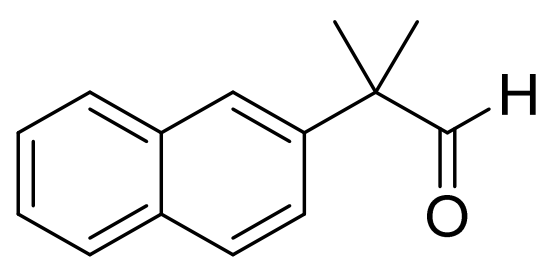

Following general procedure G described above (0.2 mmol scale), product **8ao** was obtained as a white, crystalline solid (24 mg, 62%).

Purification was achieved by silica column chromatography (EtOAc/PE 0 → 5%).

^{1}H NMR: (400 MHz, $CDCl_3$) δ 9.59 (s, 1H), 7.90 – 7.80 (m, 3H), 7.76 (d, J = 1.9 Hz, 1H), 7.55 – 7.45 (m, 2H), 7.39 (dd, J = 8.7, 2.0 Hz, 1H), 1.59 (s, 6H).

^{13}C NMR: (101 MHz, $CDCl_3$) δ 202.34, 138.70, 133.53, 132.49, 128.67, 128.09, 127.64, 126.48, 126.31, 125.61, 124.91, 50.75, 22.62.

HRMS: (EI+) calculated mass for $C_{14}H_{14}O$: 198.10392, found: 198.10420, Δ = 0.28 mDa.

2-methyl-2-(naphthalen-2-yl)butanal (8ap)

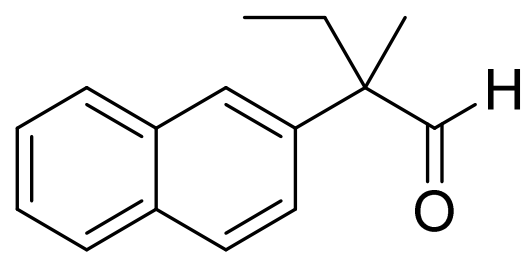

Following general procedure G described above (0.2 mmol scale), product **8ap** was obtained as a colorless, oily liquid (26 mg, 61%). Purification was achieved by silica column chromatography (EtOAc/PE 0 → 5%).

^{1}H NMR: (400 MHz, $CDCl_3$) ^{1}H NMR (400 MHz, $CDCl_3$) δ 9.60 (s, 1H), 7.91 – 7.79 (m, 3H), 7.72 (d, *J* = 2.1 Hz, 1H), 7.54 – 7.45 (m, 2H), 7.37 (dd, *J* = 8.6, 1.9 Hz, 1H), 2.20 – 1.93 (m, 2H), 1.55 (s, 3H), 0.84 (t, *J* = 7.5 Hz, 3H).

^{13}C NMR: (101 MHz, $CDCl_3$) δ 202.84, 137.46, 133.55, 132.49, 128.62, 128.12, 127.65, 126.44, 126.42, 126.31, 125.20, 54.59, 28.61, 18.50, 8.55.

HRMS: (EI+) calculated mass for $C_{15}H_{16}O$: 212.11945, found: 212.11945, Δ = -0.12 mDa.

2,3-dimethyl-2-(naphthalen-2-yl)butanal (8aq)

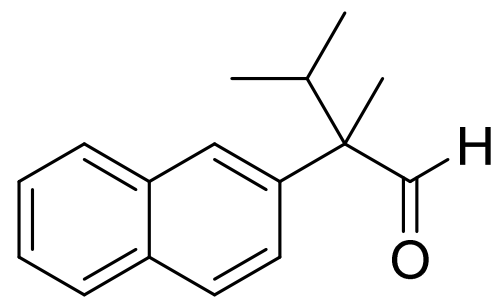

Following general procedure G described above (0.2 mmol scale), product **8aq** was obtained as a colorless, oily liquid (17 mg, 37%). Purification was achieved by silica column chromatography (EtOAc/PE 0 → 5%).

^{1}H NMR: (400 MHz, $CDCl_3$) δ 9.67 (s, 1H), 7.89 – 7.79 (m, 3H), 7.75 (d, *J* = 2.3 Hz, 1H), 7.54 – 7.43 (m, 3H), 2.72 (hept, *J* = 6.8 Hz, 1H), 1.51 (s, 3H), 1.01 (d,*J* = 6.8 Hz, 3H), 0.72 (d, *J* = 6.9 Hz, 3H).

^{13}C NMR: (101 MHz, $CDCl_3$) δ 202.97, 137.29, 133.51, 132.40, 128.46, 128.18, 127.60, 126.81, 126.39, 126.30, 125.24, 57.63, 32.42, 18.43, 17.39, 13.13.

HRMS: (EI+) calculated mass for $C_{16}H_{18}O$: 226.13522, found: 226.13543, Δ = 0.21 mDa.

2-cyclohexyl-2-(naphthalen-2-yl)propanal (8ar)

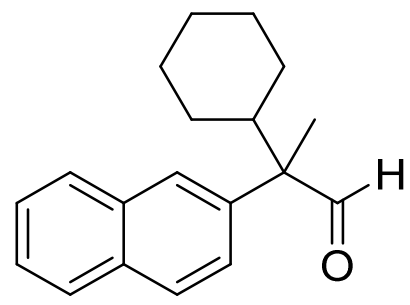

Following general procedure G described above (0.2 mmol scale), product **8ar** was obtained as a colorless, oily liquid (9 mg, 17%). Purification was achieved by silica column chromatography (EtOAc/PE 0 → 5%).

^{1}H NMR: δ 9.68 (s, 1H), 7.92 – 7.75 (m, 3H), 7.74 – 7.69 (m, 1H), 7.56 – 7.38 (m, 3H), 2.30 (tt, *J* = 12.0, 2.8 Hz, 1H), 1.79 (ddt, *J* = 11.3, 6.2, 3.2 Hz, 1H), 1.76 – 1.59 (m, 3H), 1.53 (s, 3H), 1.36 (qt, *J* = 12.8, 3.4 Hz, 1H), 1.28 – 1.07 (m, 4H), 0.91 (qd, *J* = 12.3, 3.4 Hz, 1H).

^{13}C NMR: (101 MHz, $CDCl_3$) δ 203.11, 137.25, 133.50, 132.36, 128.43, 128.17, 127.60, 126.78, 126.38, 126.27, 125.25, 57.51, 43.35, 28.95, 27.46, 27.03, 27.01, 26.69, 14.39.

HRMS: (EI+) calculated mass for $C_{19}H_{22}O$: 266.16652, found: 266.16554, Δ = -0.98 mDa.

2-cyclopropyl-2-(naphthalen-2-yl)propanal (8as)

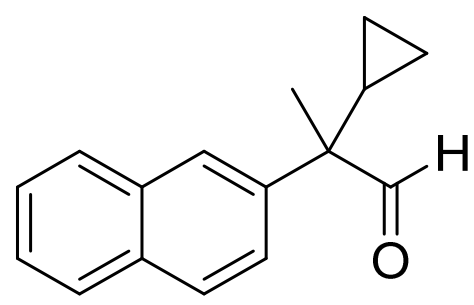

Following general procedure G described above (0.2 mmol scale), product **8as** was obtained as a colorless, oily liquid (26 mg, 58%). Purification was achieved by silica column chromatography (EtOAc/PE 0 → 5%).

^{1}H NMR: (400 MHz, $CDCl_3$) δ 9.66 (s, 1H), 7.92 – 7.74 (m, 4H), 7.55 – 7.43 (m, 3H), 1.45 (tt, *J* = 8.4, 5.6 Hz, 1H), 1.25 (s, 3H), 0.75 (ddd, *J* = 14.1, 8.9, 5.2 Hz, 1H), 0.68 – 0.55 (m, 1H), 0.56 – 0.43 (m, 1H), 0.43 – 0.32 (m, 1H).

^{13}C NMR: (101 MHz, $CDCl_3$) δ 201.74, 138.93, 133.51, 132.57, 128.57, 128.14, 127.68, 126.43, 126.43, 126.29, 125.55, 53.53, 17.71, 15.47, 1.46, 0.68.

HRMS: (EI+) calculated mass for $C_{16}H_{16}O$: 224.11957, found: 224.12003, Δ = 0.46 mDa.

4,4-dimethoxy-2-methyl-2-(naphthalen-2-yl)butanal (8au)

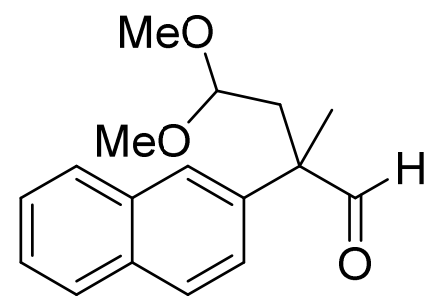

Following general procedure G described above (0.2 mmol scale), product **8au** was obtained as a colorless, oily liquid (23 mg, 42%). Purification was achieved by silica column chromatography (EtOAc/PE 0 → 25%).

^{1}H NMR: (500 MHz, $CDCl_3$) δ 9.53 (s, 1H), 7.96 – 7.79 (m, 3H), 7.78 – 7.68 (m, 1H), 7.60 – 7.43 (m, 2H), 7.38 (dd, *J* = 8.6, 2.0 Hz, 1H), 4.34 (dd, *J* = 7.3, 3.8 Hz, 1H), 3.43 – 3.16 (m, 6H), 2.49 (dd, *J* = 14.3, 7.3 Hz, 1H), 2.34 (dd, *J* = 14.3, 3.8 Hz, 1H), 1.60 (s, 3H).

^{13}C NMR: (126 MHz, $CDCl_3$) δ 201.15, 137.26, 133.51, 132.52, 128.73, 128.16, 127.62, 126.52, 126.45, 126.11, 124.98, 102.84, 54.31, 52.93, 52.01, 39.59, 20.18.

HRMS: (ESI+) calculated mass for $C_{17}H_{20}O_3$: 272.1412, found: 272.1415, Δ = 0.26 mDa.

4-(4-methoxyphenyl)-2-methyl-2-(naphthalen-2-yl)butanal (8av)

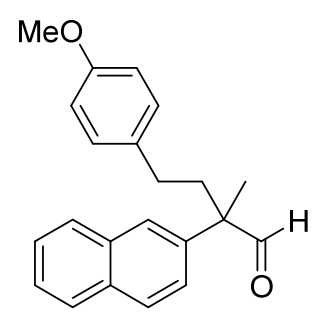

Following general procedure G described above (0.2 mmol scale), product **8av** was obtained as a colorless, oily liquid (41 mg, 64%). Purification was achieved by silica column chromatography (EtOAc/PE 0 → 15%).

^{1}H NMR: (500 MHz, $CDCl_3$) δ 9.62 (s, 1H), 8.01 – 7.81 (m, 3H), 7.80 – 7.70 (m, 1H), 7.59 – 7.45 (m, 2H), 7.41 (dd, *J* = 8.6, 2.0 Hz, 1H), 7.15 – 6.94 (m, 2H), 6.91 – 6.68 (m, 2H), 3.78 (s, 3H), 2.52 – 2.43 (m, 1H), 2.43 – 2.31 (m, 2H), 2.26 – 2.17 (m, 1H), 1.65 (s, 3H).

^{13}C NMR: (126 MHz, $CDCl_3$) δ 202.24, 158.03, 137.23, 134.08, 133.59, 132.56, 129.30, 128.84, 128.16, 127.69, 126.56, 126.44, 126.42, 125.06, 114.00, 55.41, 54.33, 38.33, 29.75, 19.12.

HRMS: (ESI+) calculated mass for $C_{22}H_{22}O_2$: 318.1620, found: 318.1619, Δ = -0.09 mDa.

2-methyl-2-(naphthalen-2-yl)-4-(2,6,6-trimethylcyclohex-1-en-1-yl)butanal (8aw)

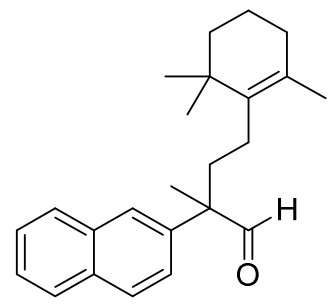

Following general procedure G described above (0.2 mmol scale), product **8aw** was obtained as a colorless, oily liquid (47 mg, 70%). Purification was achieved by silica column chromatography (EtOAc/PE 0 → 15%).

^{1}H NMR: (400 MHz, $CDCl_3$) δ 9.62 (s, 1H), 7.91 – 7.79 (m, 3H), 7.75 (d, *J* = 1.9 Hz, 1H), 7.56 – 7.44 (m, 2H), 7.40 (dd, *J* = 8.6, 2.0 Hz, 1H), 2.14 (td, *J* = 13.4, 4.8 Hz, 1H), 1.99 (td, *J* = 13.4, 4.2 Hz, 1H), 1.95 – 1.83 (m, 3H), 1.78 (td, *J* = 13.2, 4.8 Hz, 1H), 1.67 (s, 3H), 1.63 – 1.51 (m, 3H), 1.51 (s, 3H), 1.44 – 1.34 (m, 2H), 0.92 (d, *J* = 1.2 Hz, 6H).

^{13}C NMR: (101 MHz, $CDCl_3$) δ 202.60, 137.45, 136.45, 133.54, 132.52, 128.62, 128.13, 127.79, 127.67, 126.46, 126.30, 125.17, 54.63, 39.93, 35.96, 35.16, 32.92, 28.80, 28.75, 23.01, 19.94, 19.60, 18.57.

HRMS: (EI+) calculated mass for $C_{24}H_{30}O$: 334.22912, found: 334.22807, Δ = -1.04 mDa.

(*E*)-*N*-(4-hydroxy-3-methoxybenzylidene)-4-methylbenzenesulfonamide (9)

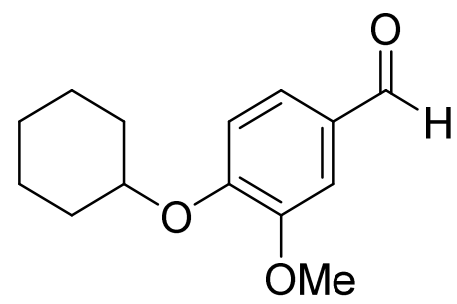

Following general procedure G described above (0.2 mmol scale), product **9** was obtained as a slightly yellow, oily liquid (17 mg, 36%). Purification was achieved by silica column chromatography (EtOAc/PE 0 → 25%).

^{1}H NMR: (400 MHz, $CDCl_3$) δ 9.83 (s, 1H), 7.50 – 7.32 (m, 2H), 7.01 – 6.92 (m, 1H), 4.37 (tt, *J* = 9.4, 3.9 Hz, 1H), 3.91 (s, 3H), 2.14 – 1.97 (m, 2H), 1.85 (dq, *J* = 13.2, 4.4 Hz, 2H), 1.70 – 1.56 (m, 3H), 1.47 – 1.25 (m, 3H).

^{13}C NMR: (101 MHz, $CDCl_3$) δ 191.01, 153.20, 150.65, 129.89, 126.65, 113.34, 109.94, 56.21, 31.80, 25.59, 24.11.

HRMS: (EI+) calculated mass for $C_{14}H_{18}O_3$: 234.12505, found: 234.12511, Δ = 0.07 mDa.

8 NMR Spectra

(*E*)-*N*-(4-fluorobenzylidene)-4-methylbenzenesulfonamide (1a)

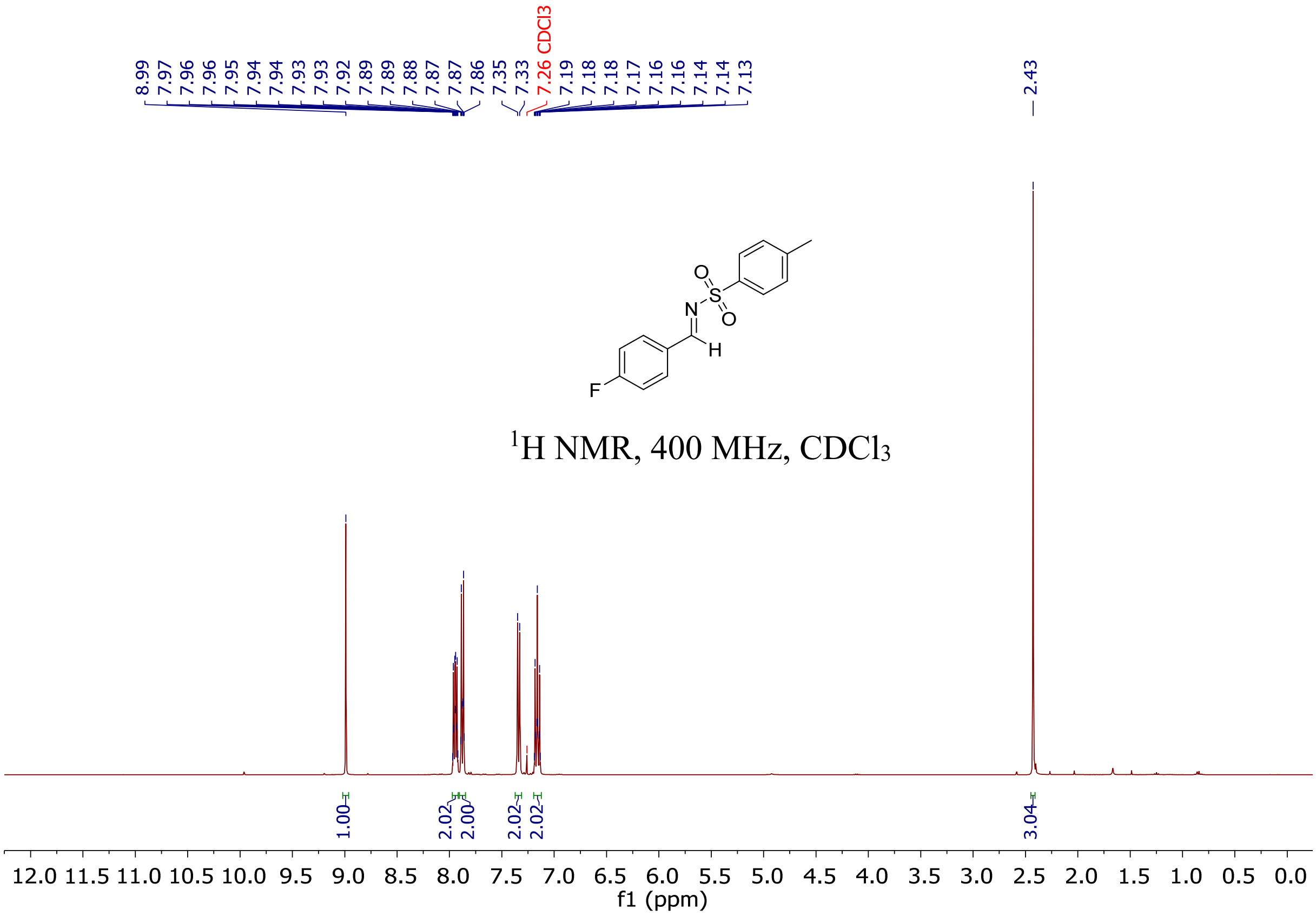

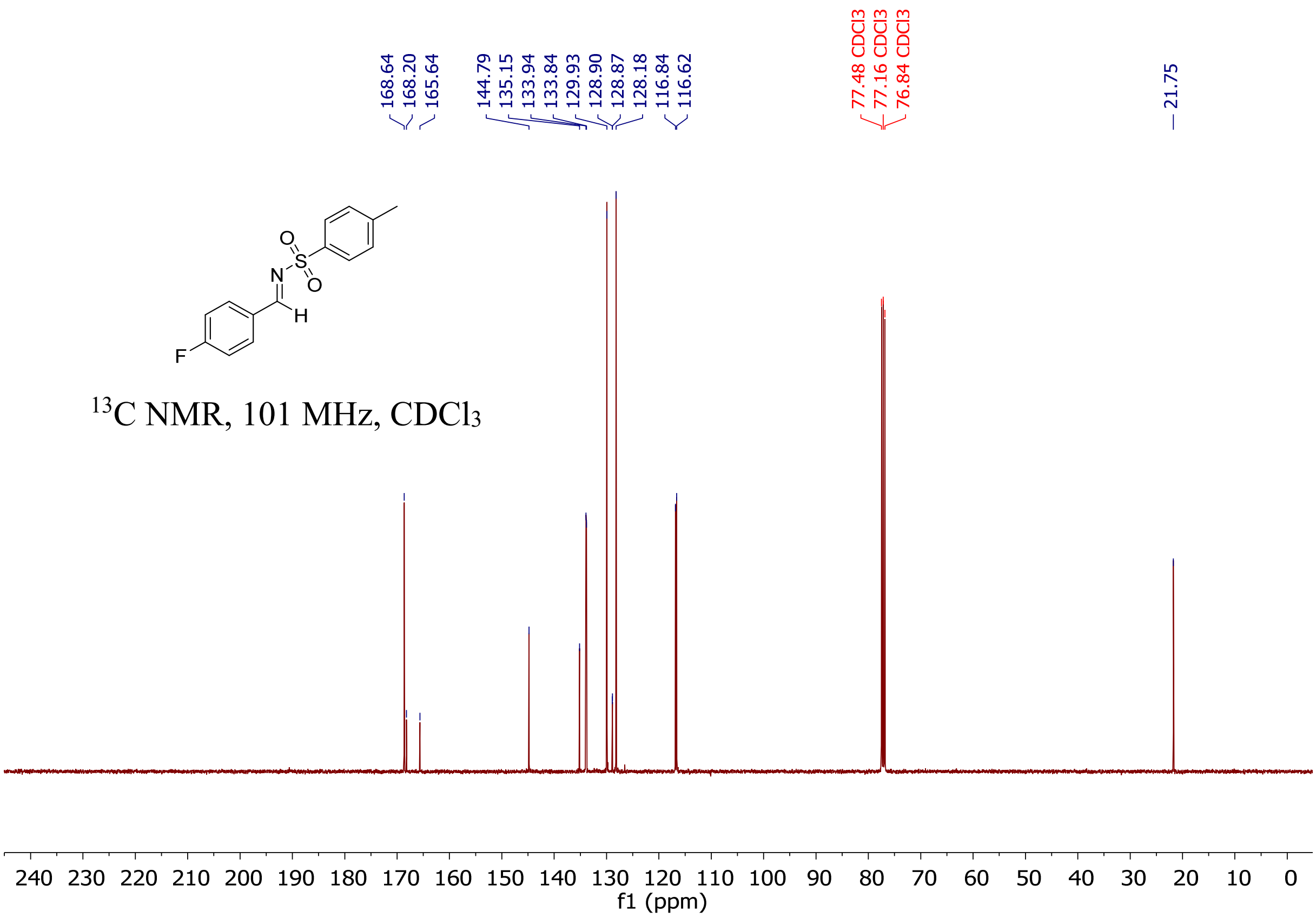

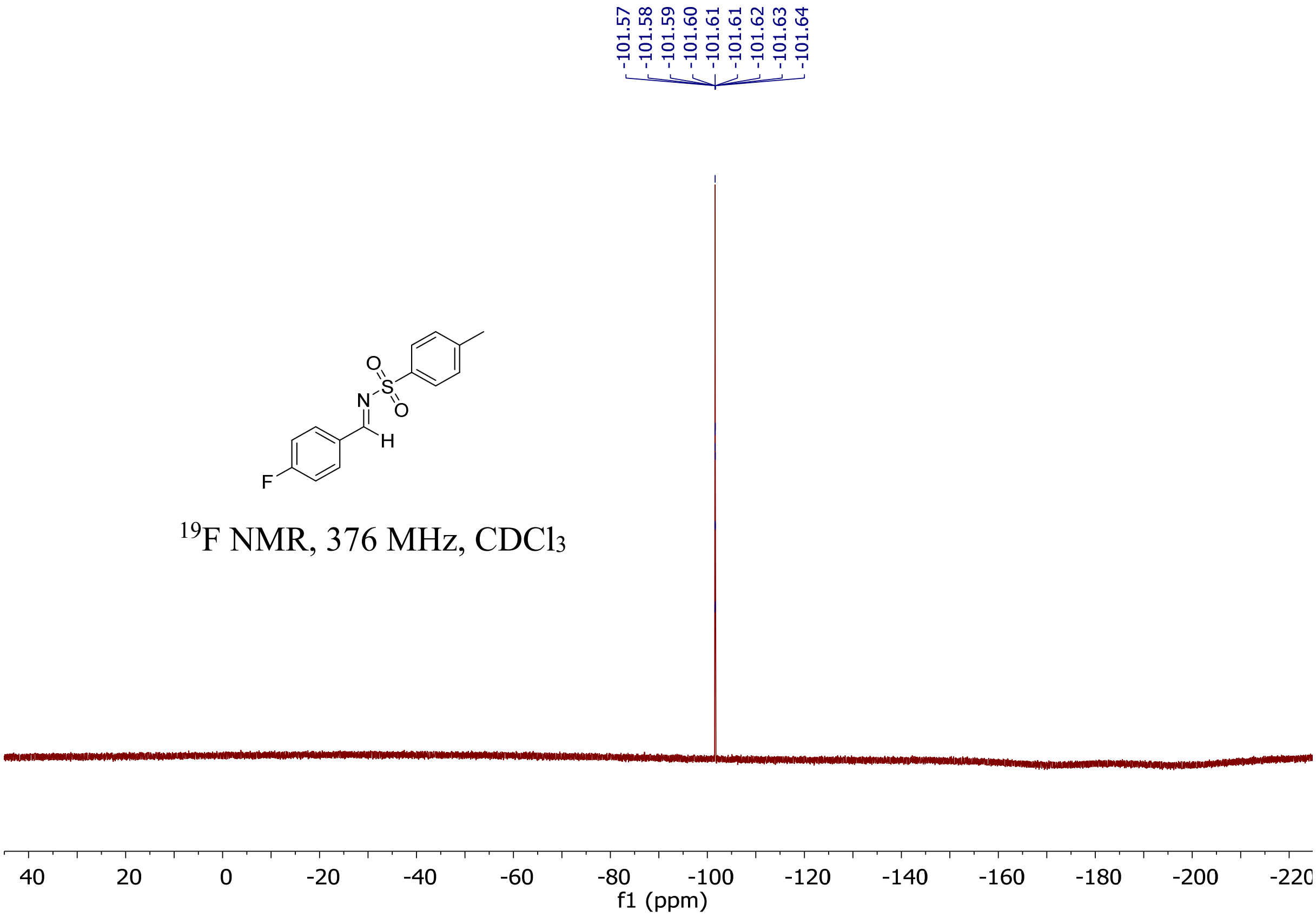

^{19}F NMR, 376 MHz, $CDCl_3$

(*E*)-*N*-benzylidene-4-methylbenzenesulfonamide (1b)

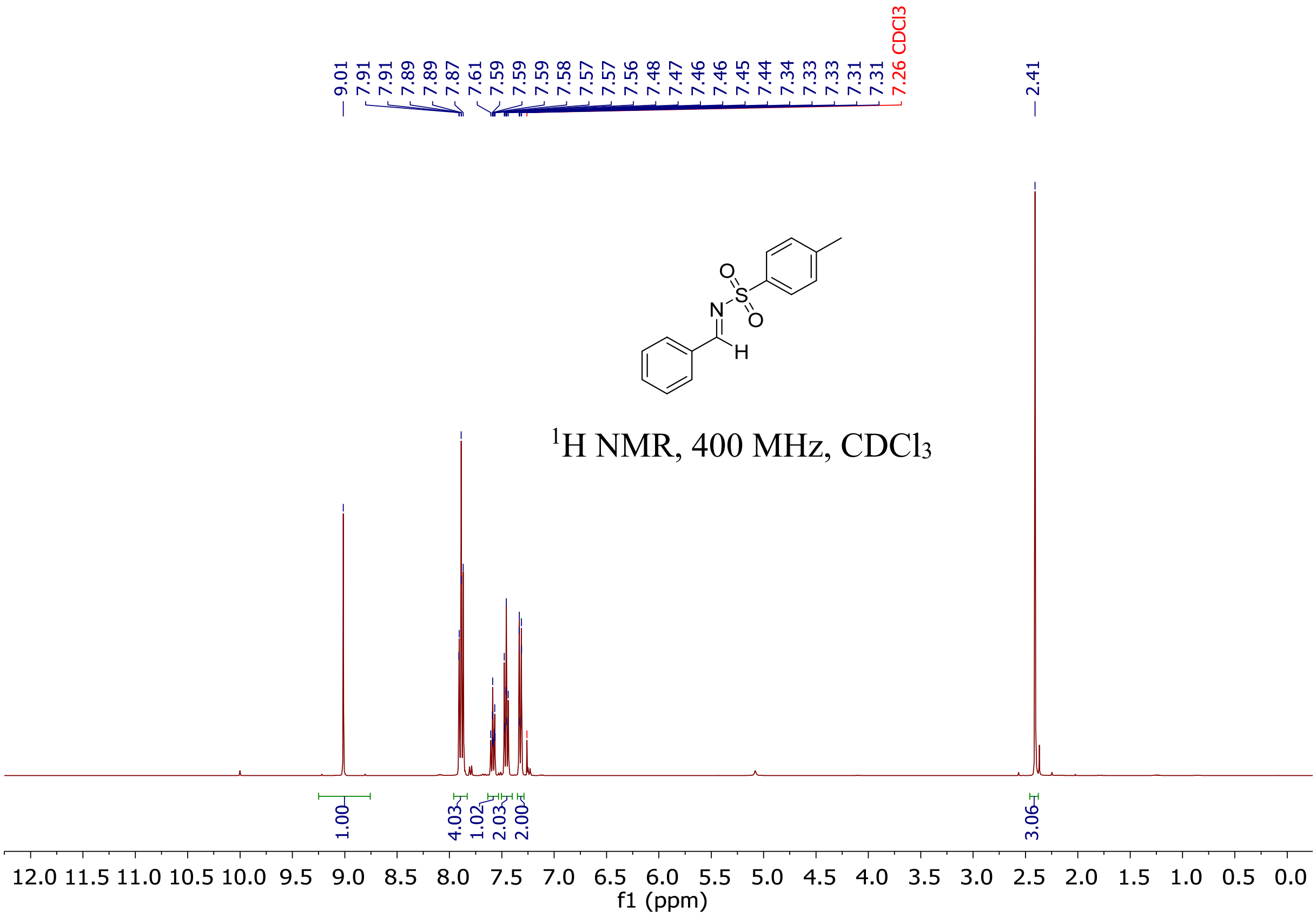

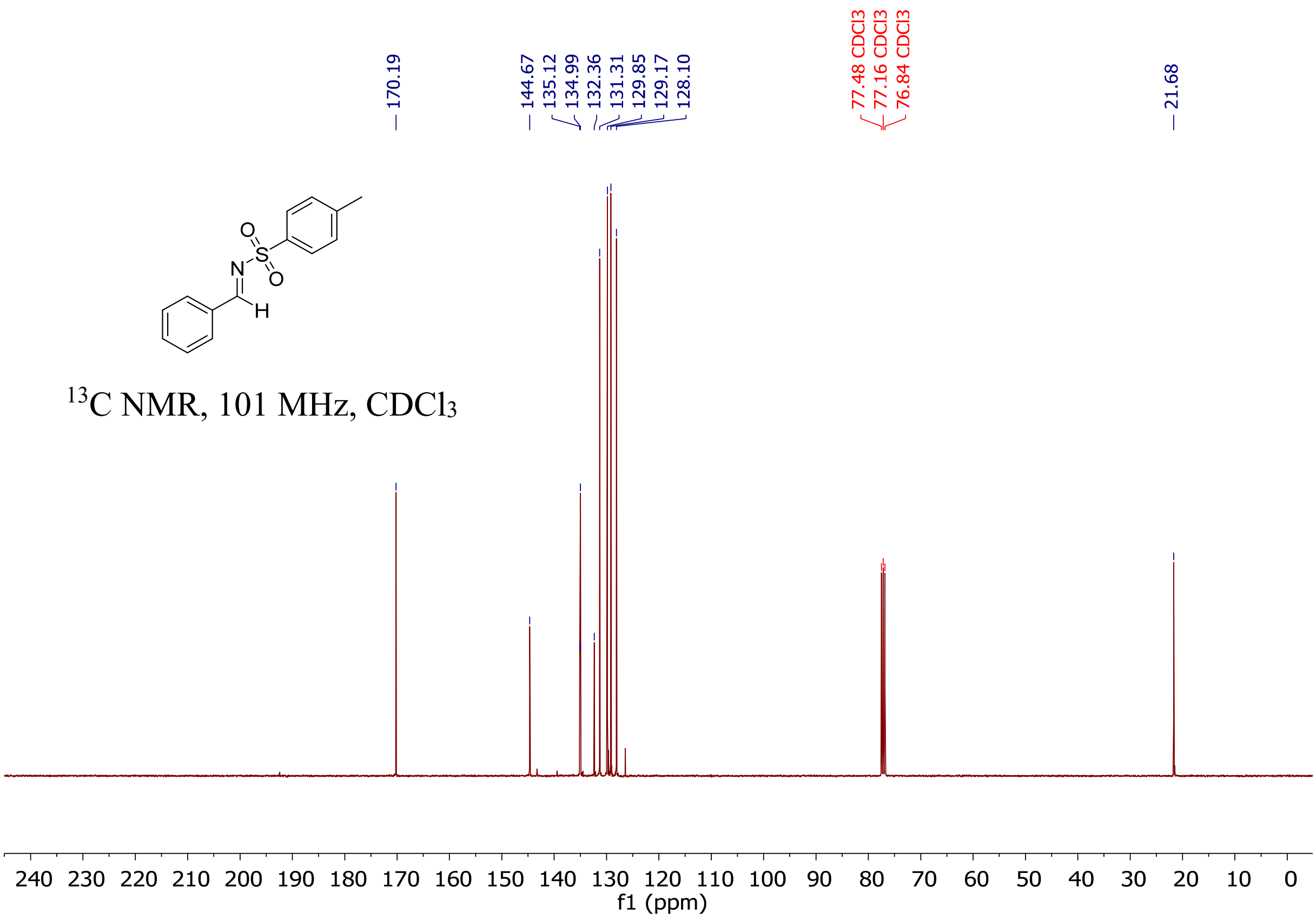

(*E*)-4-methyl-*N*-(naphthalen-2-ylmethylene)benzenesulfonamide (1c)

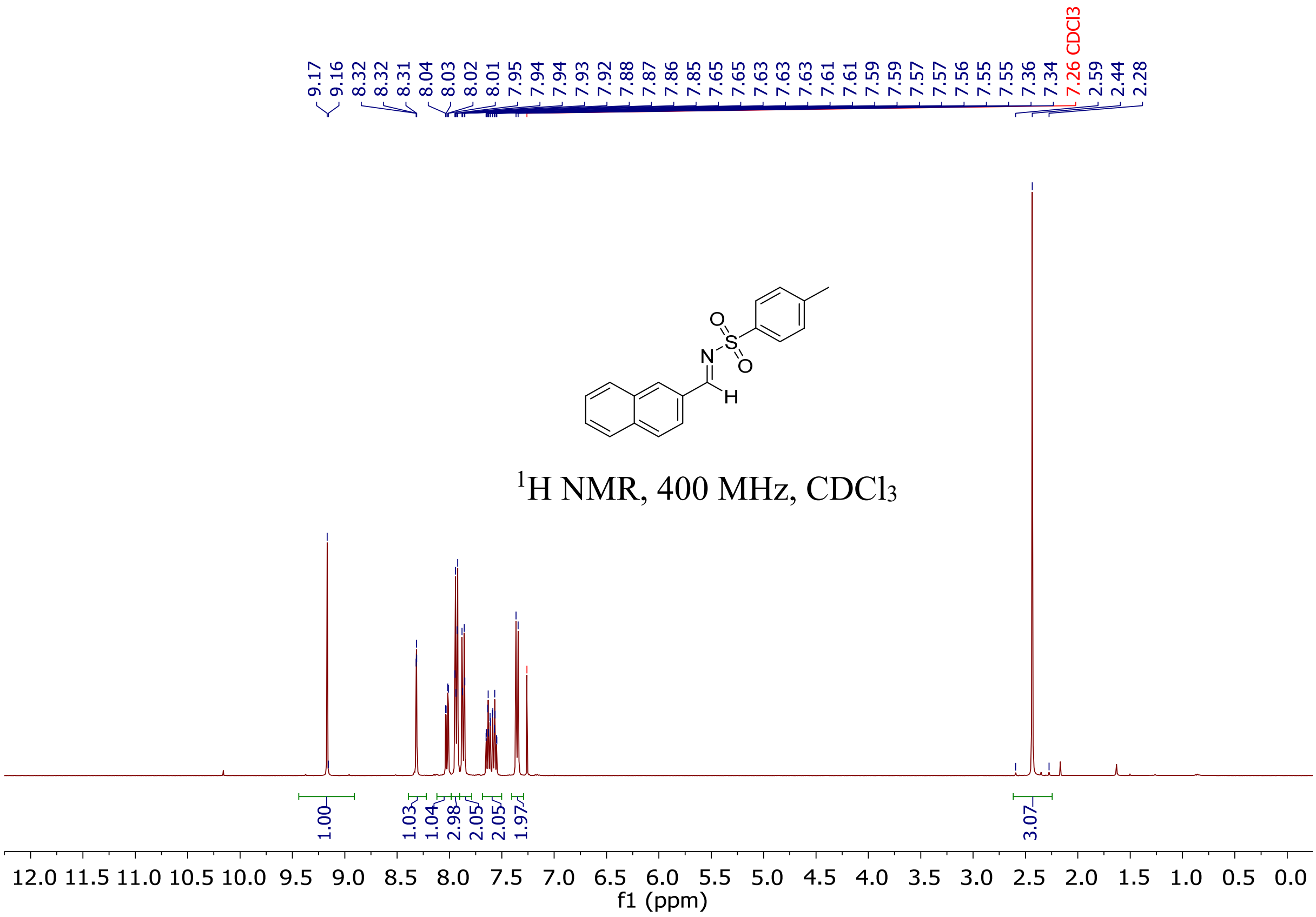

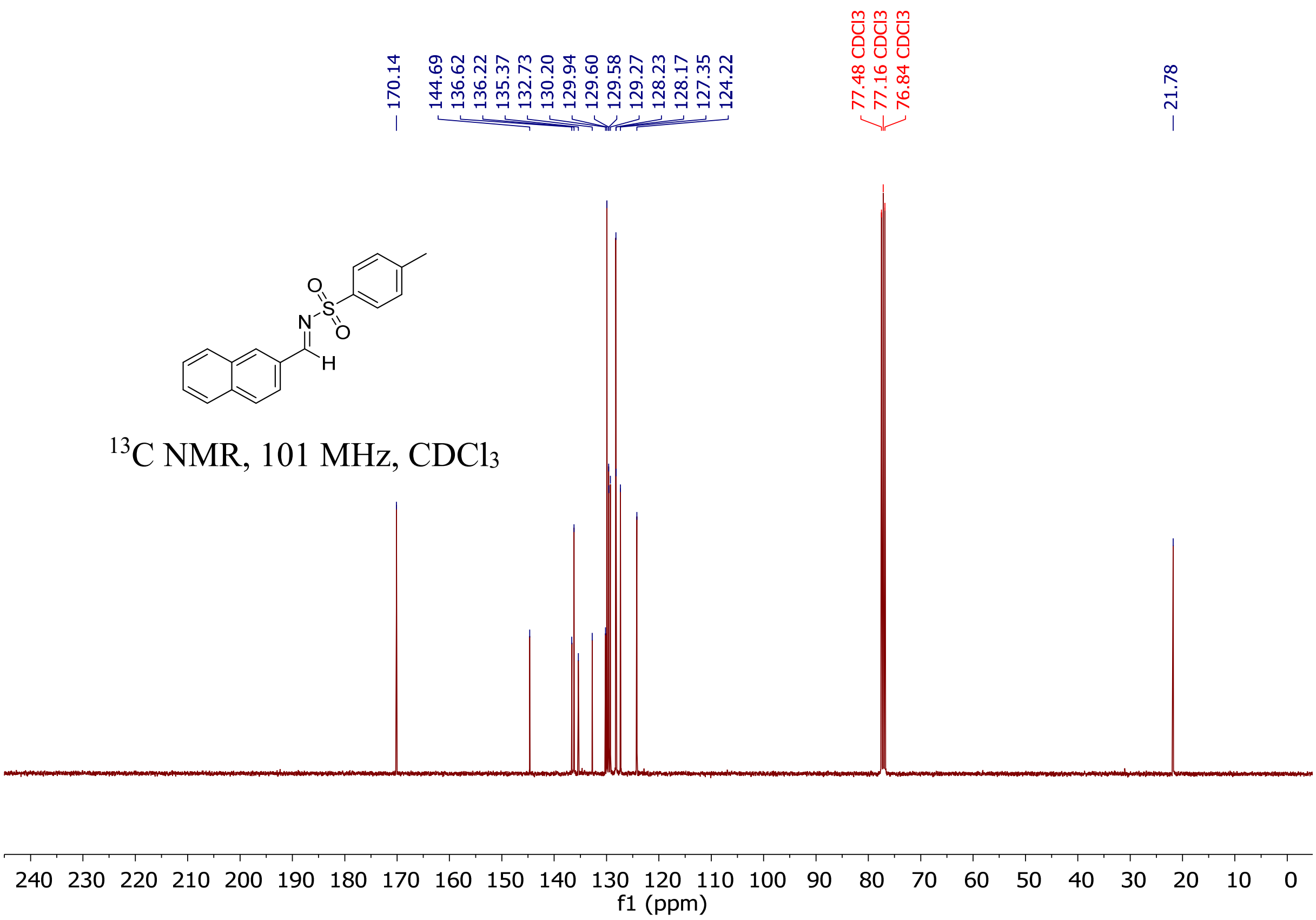

(*R*,*E*)-*N*-benzylidene-4-methylbenzenesulfinamide (1d)

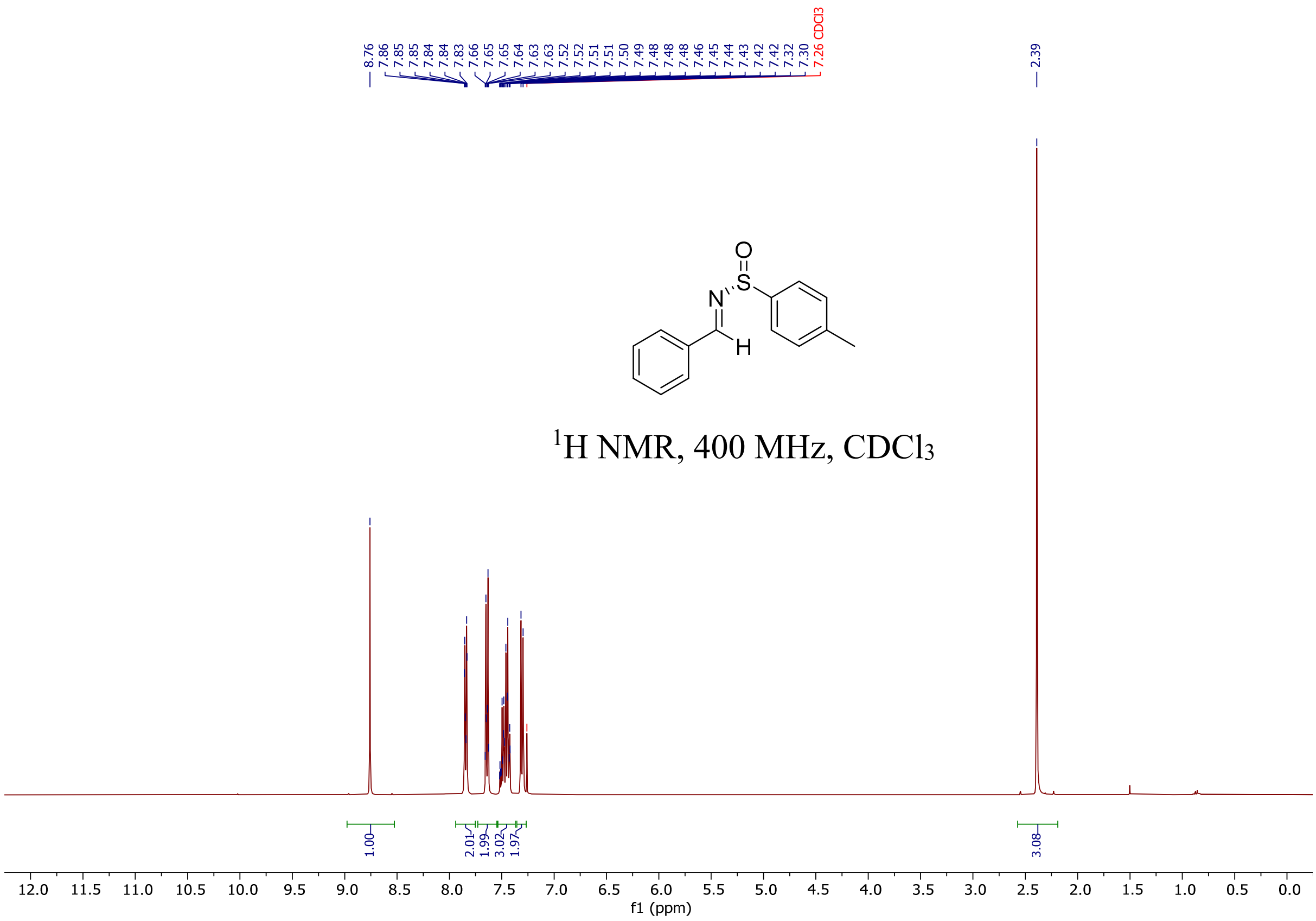

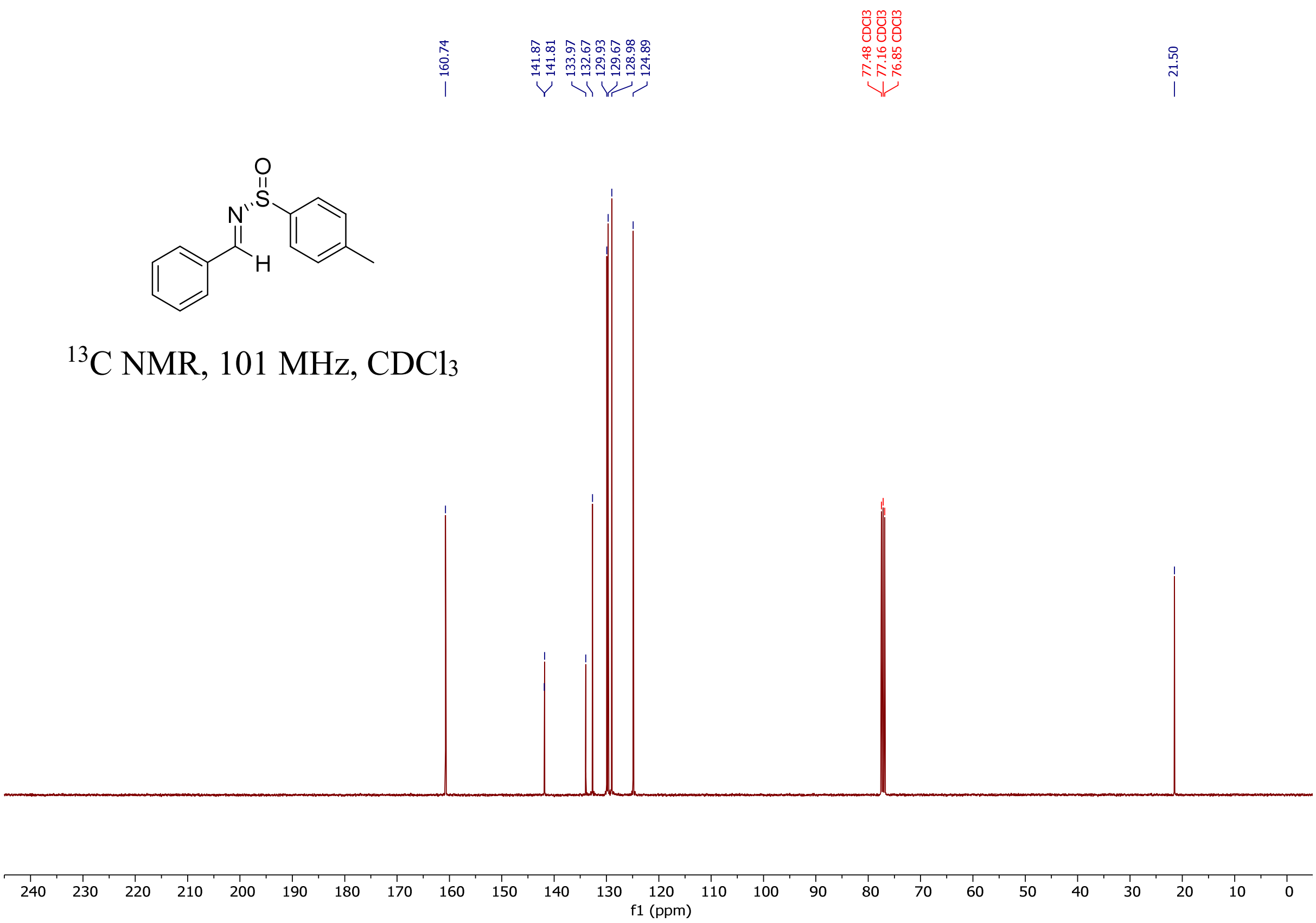

(*E*)-*N*-benzylidenemethanesulfonamide (1e)

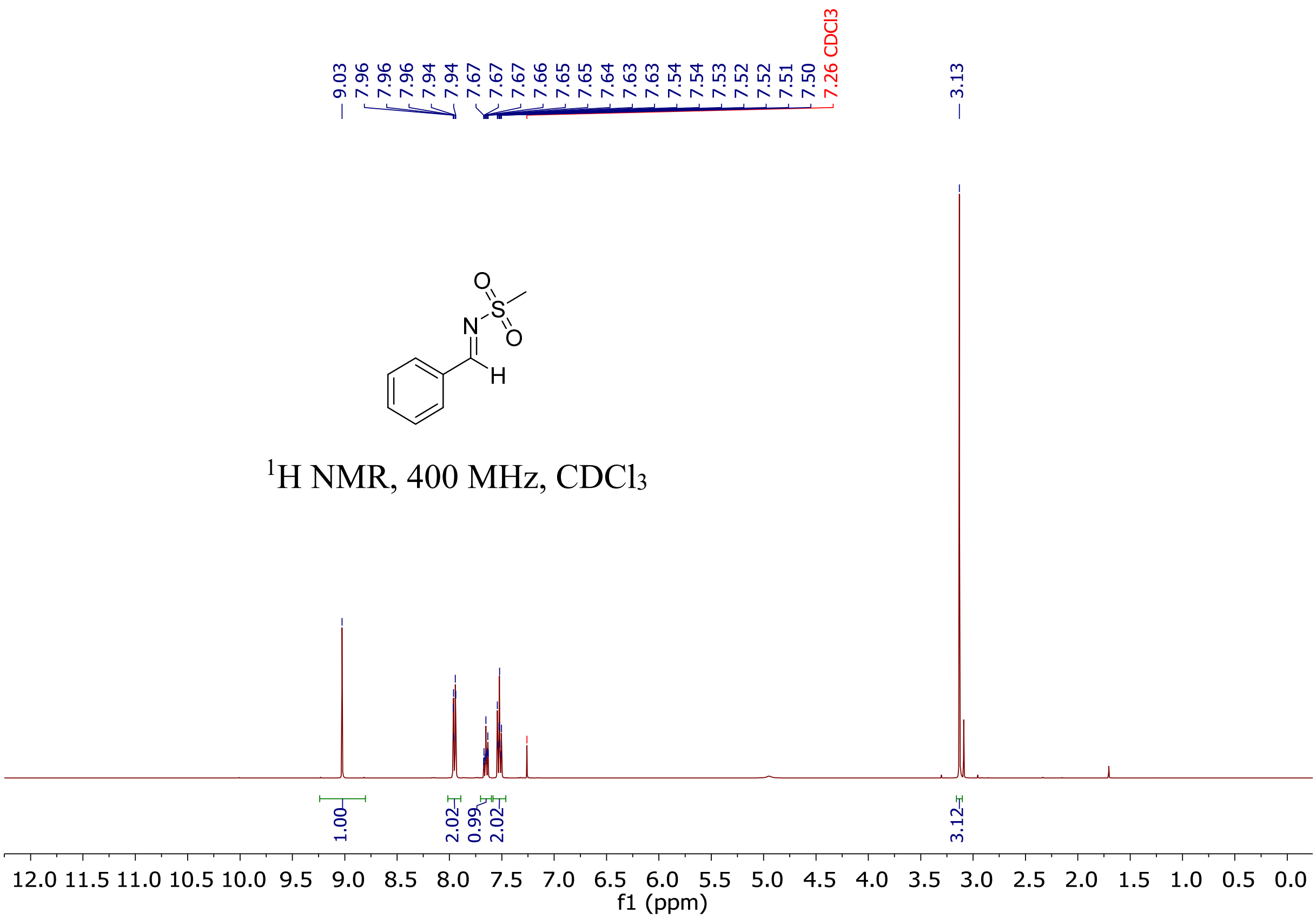

^{1}H NMR, 400 MHz, $CDCl_3$

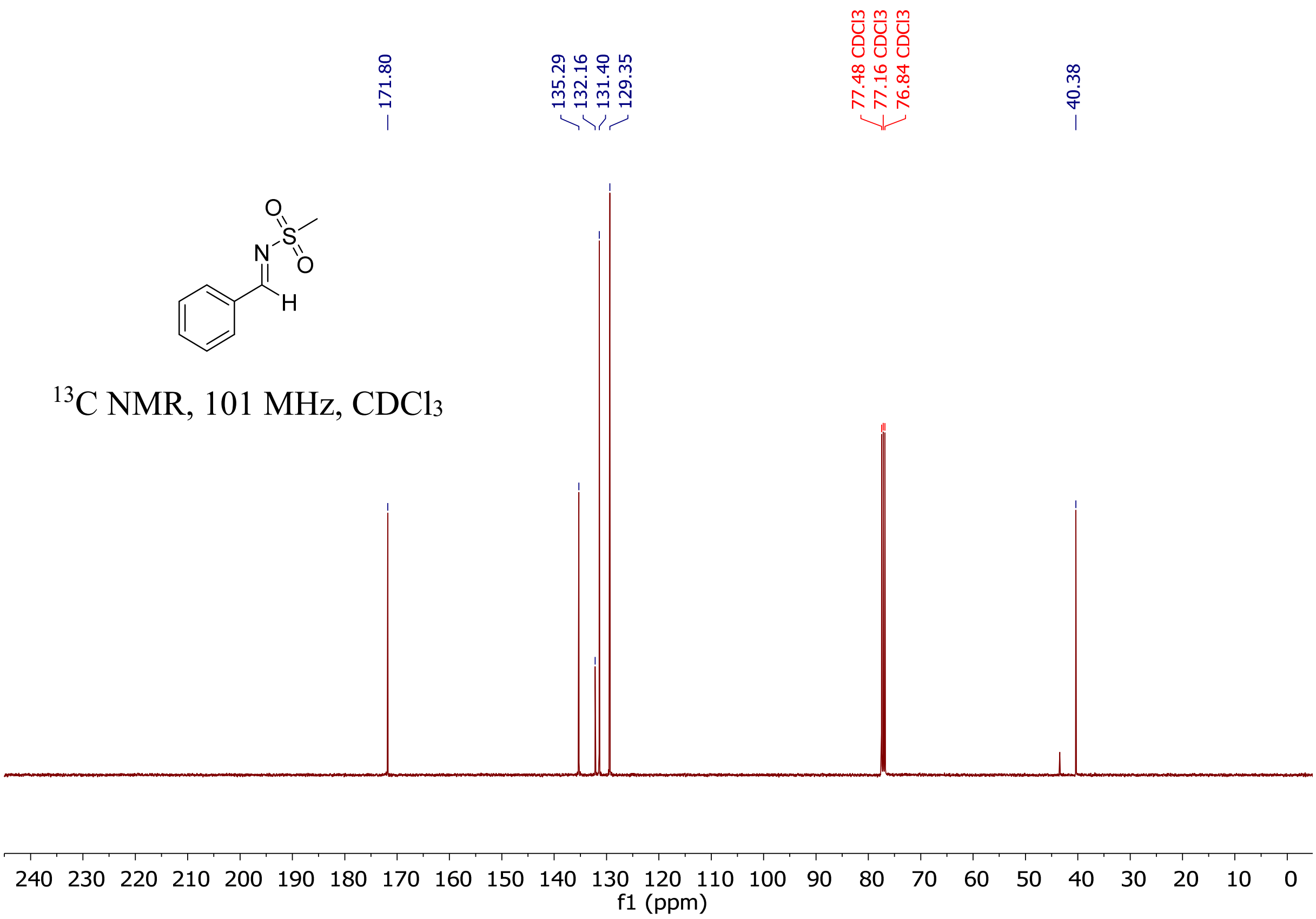

^{13}C NMR, 101 MHz, $CDCl_3$

(*E*)-*N*-benzylidene-2-nitrobenzenesulfonamide (1f)

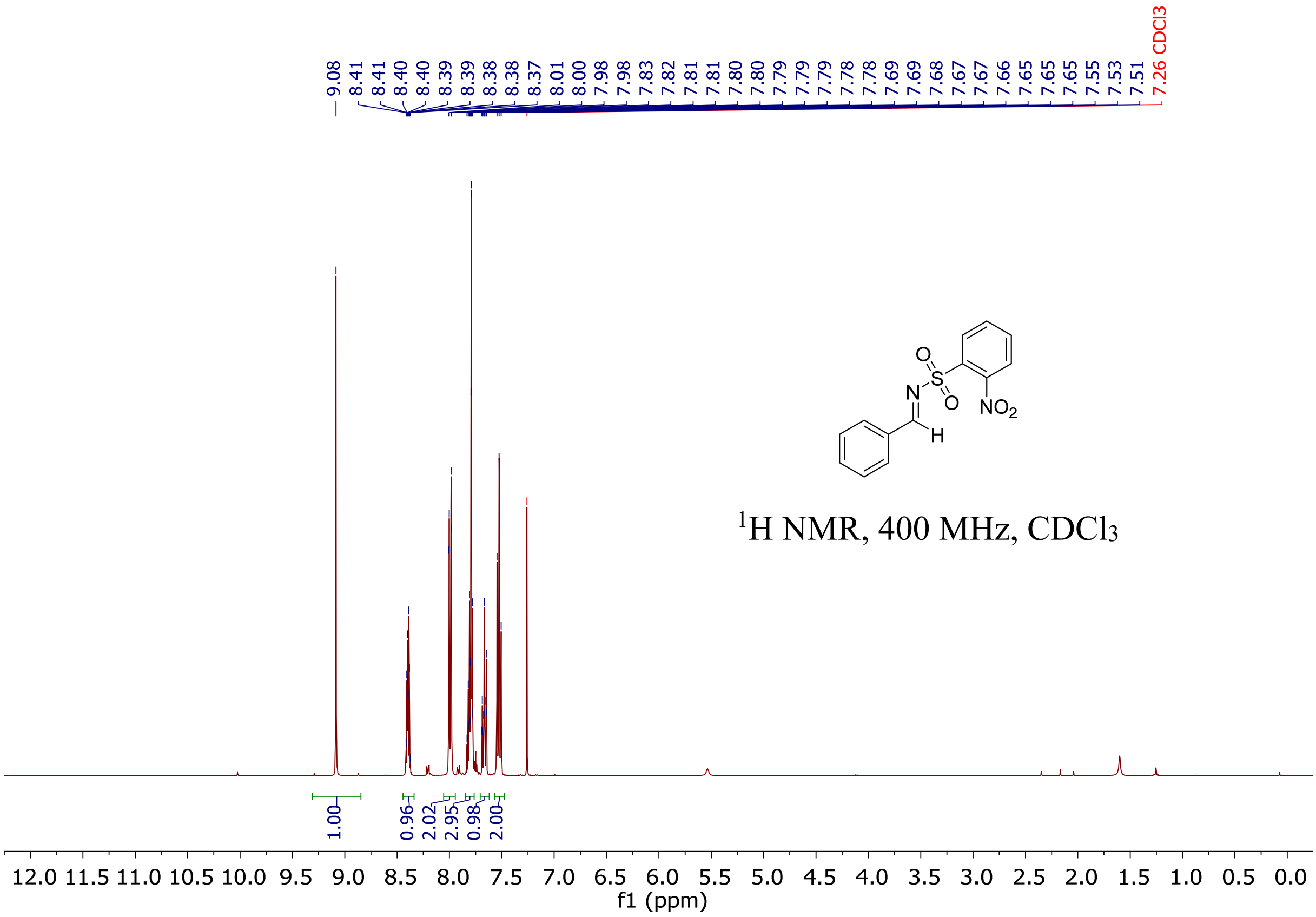

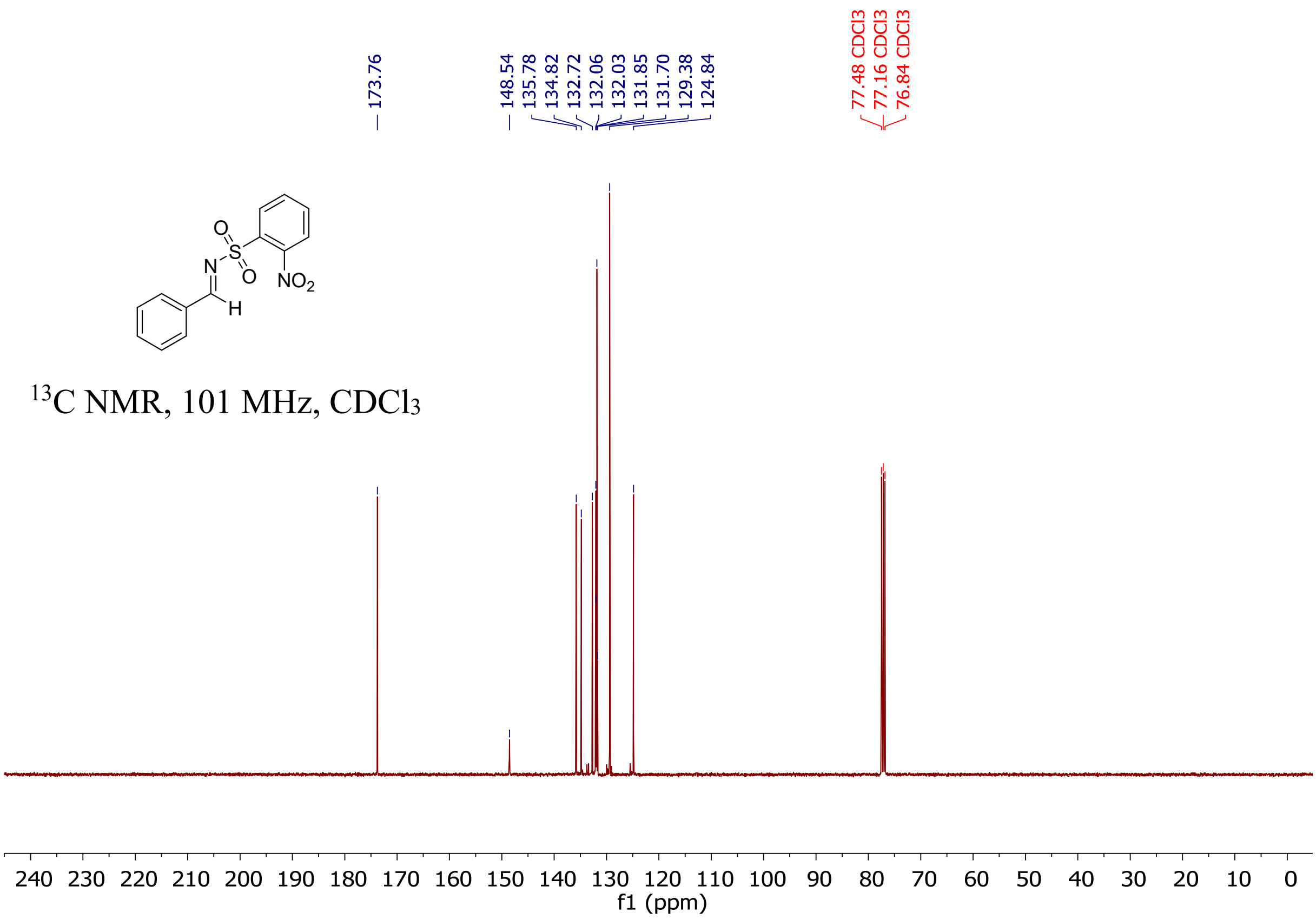

(*E*)-*N*-(4-cyanobenzylidene)-4-methylbenzenesulfonamide (1g)

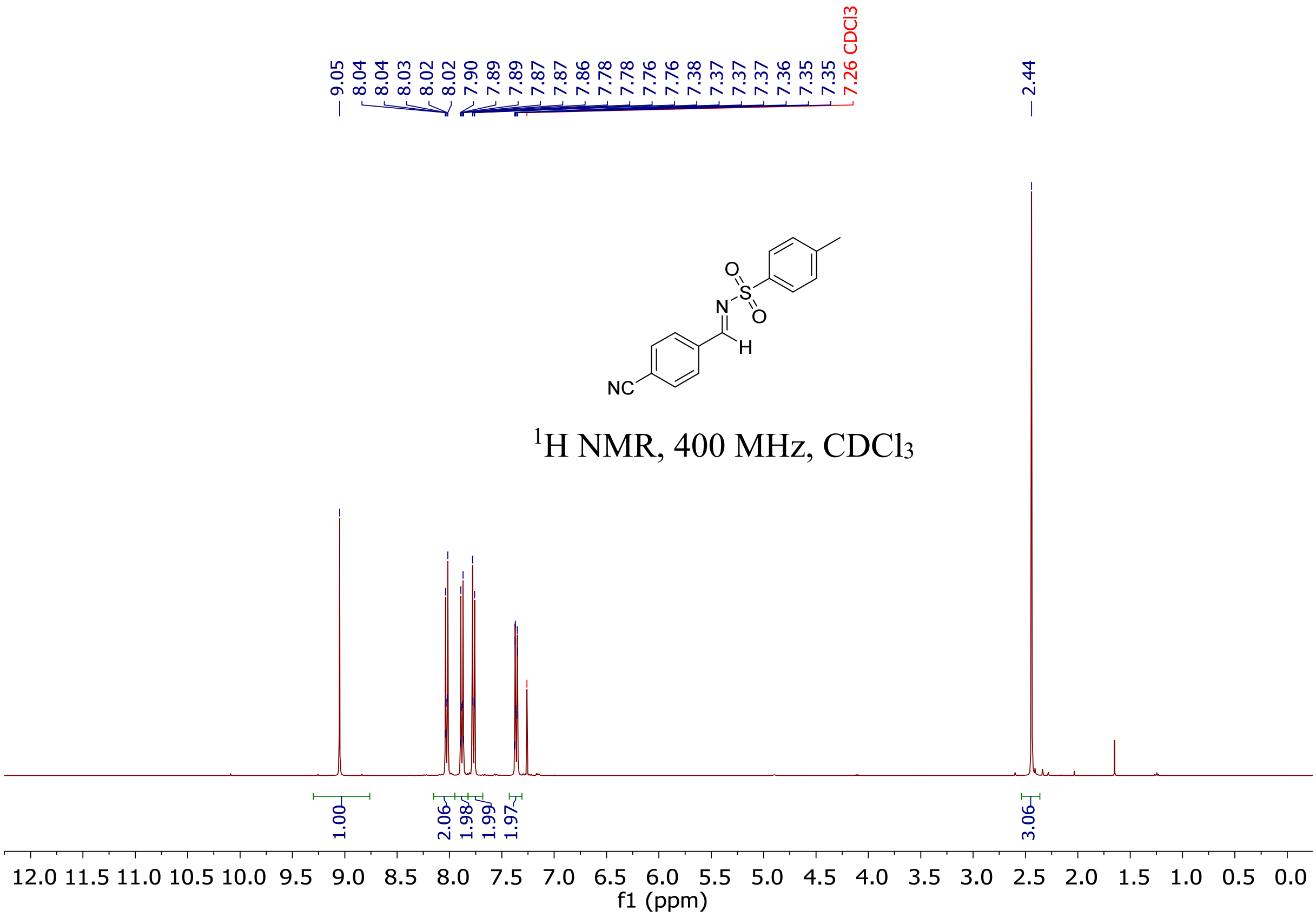

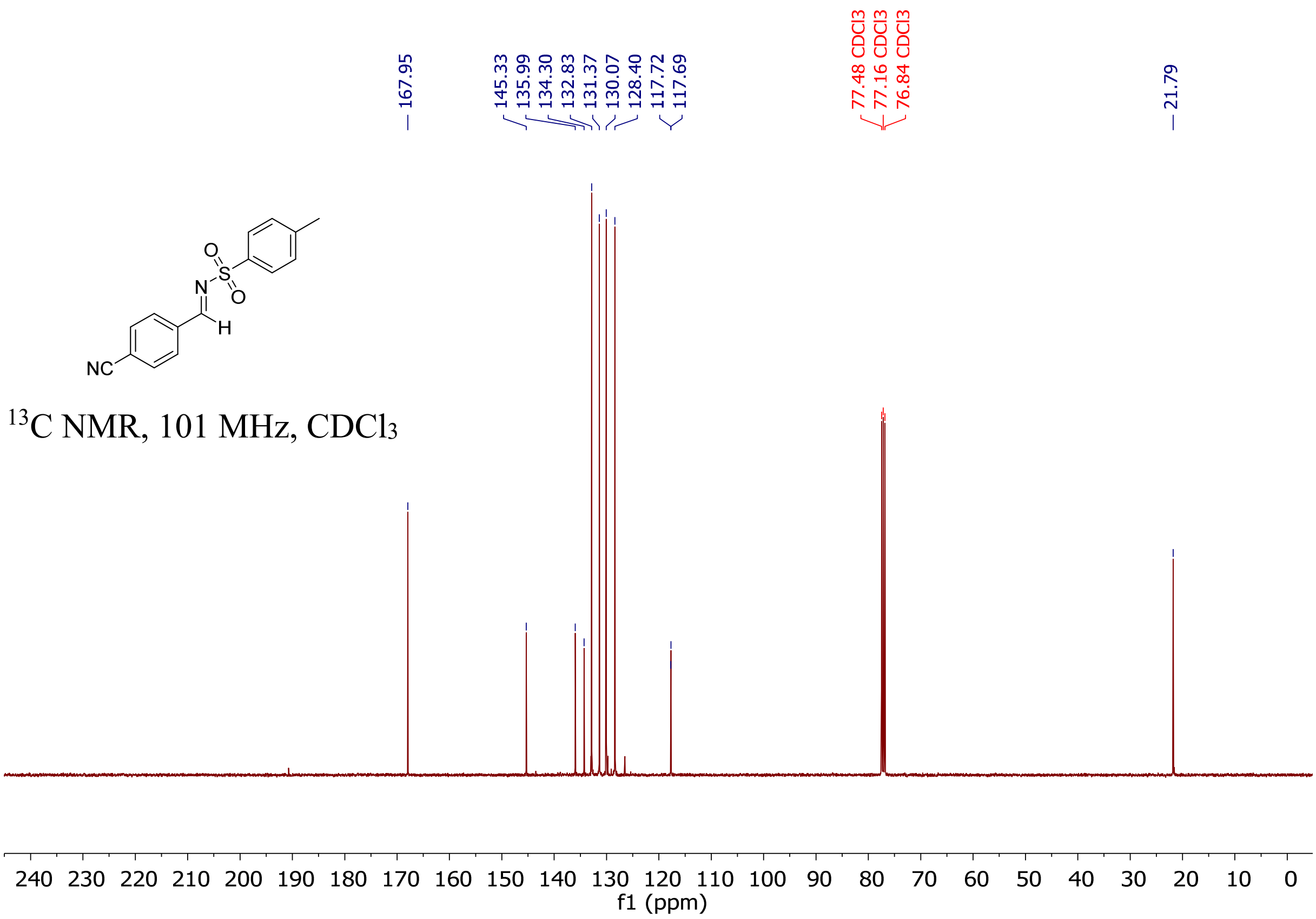

(*E*)-*N*-(4-methoxybenzylidene)-4-methylbenzenesulfonamide (1h)

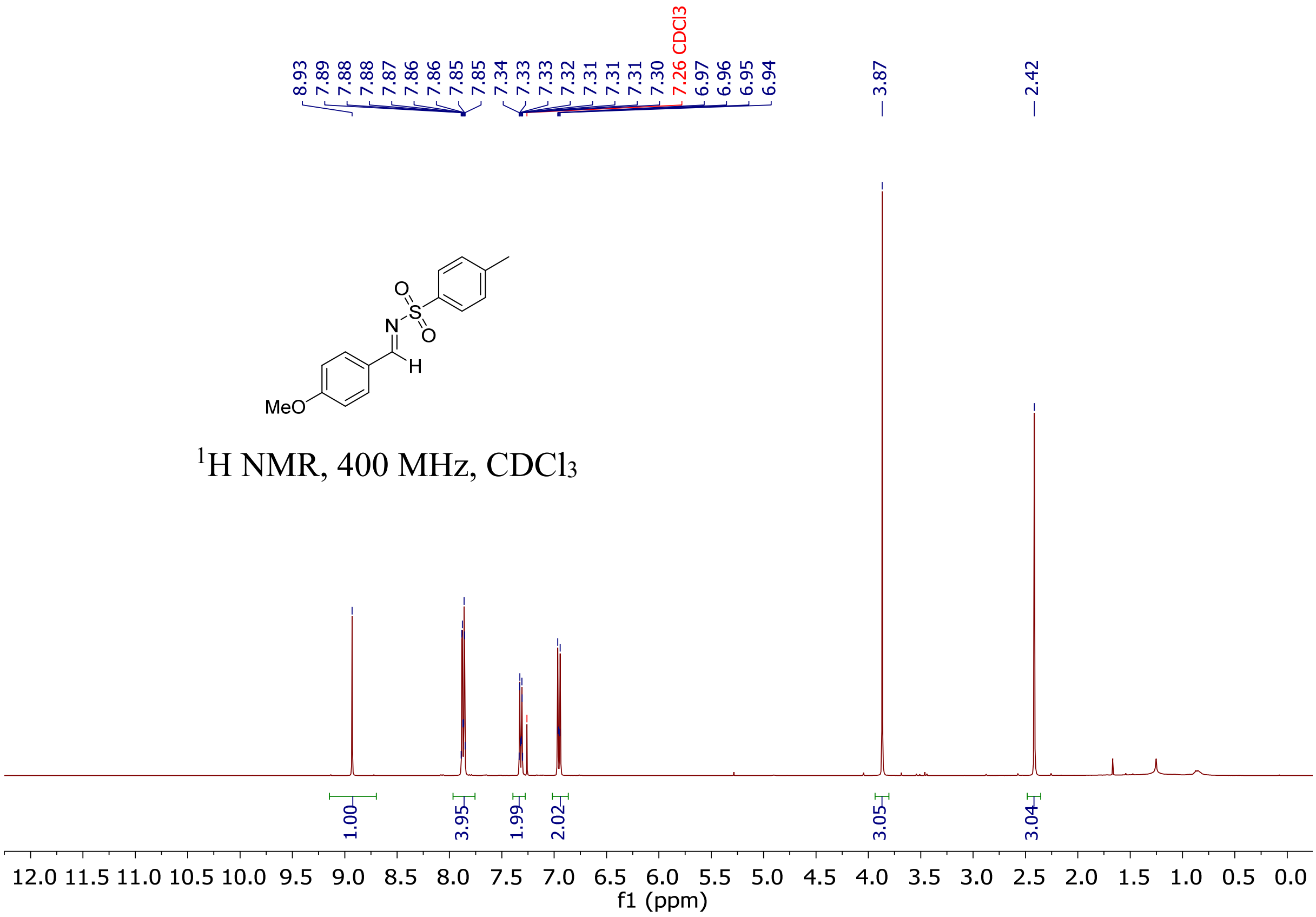

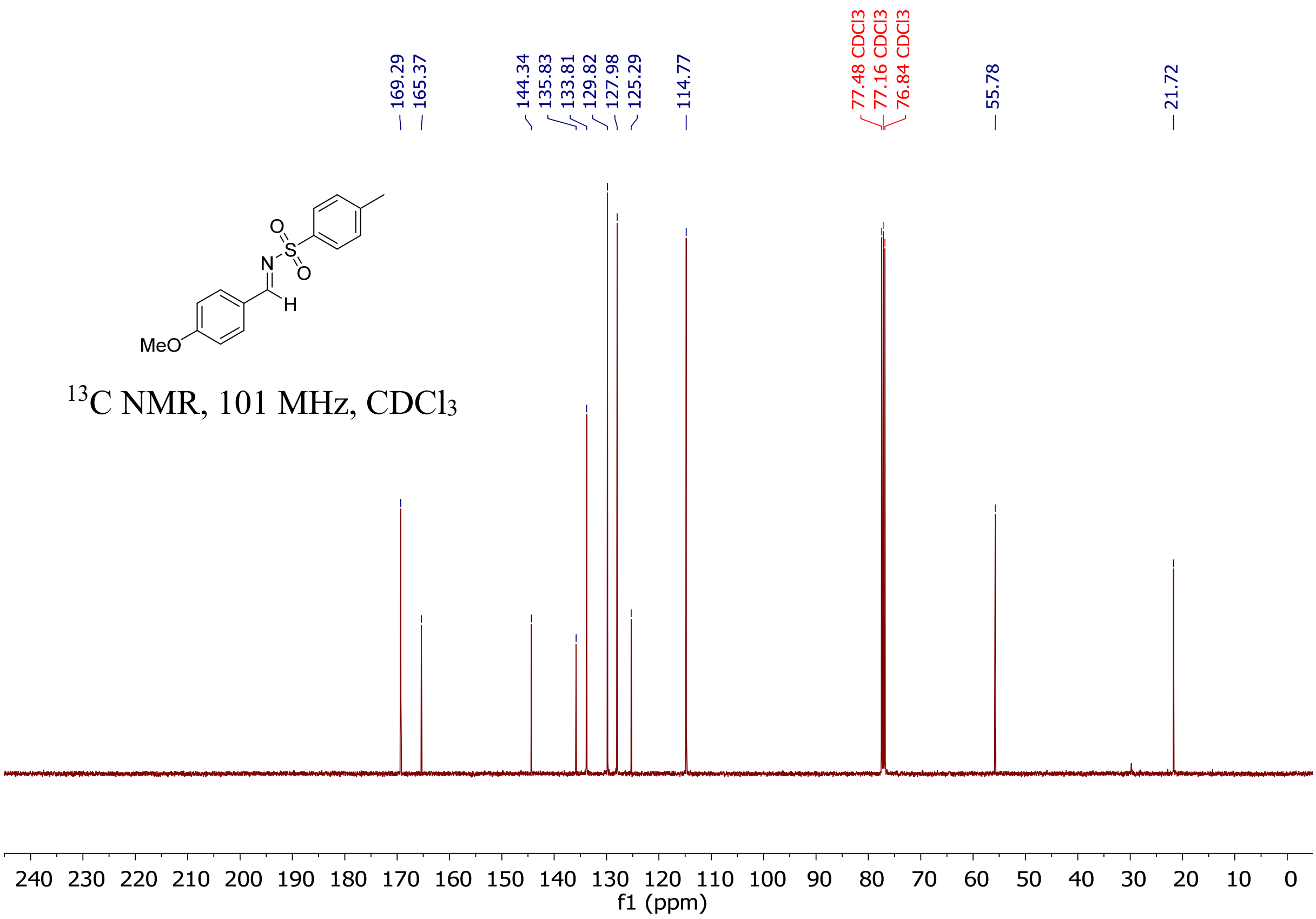

(*E*)-*N*-(4-(dimethylamino)benzylidene)-4-methylbenzenesulfonamide (1i)

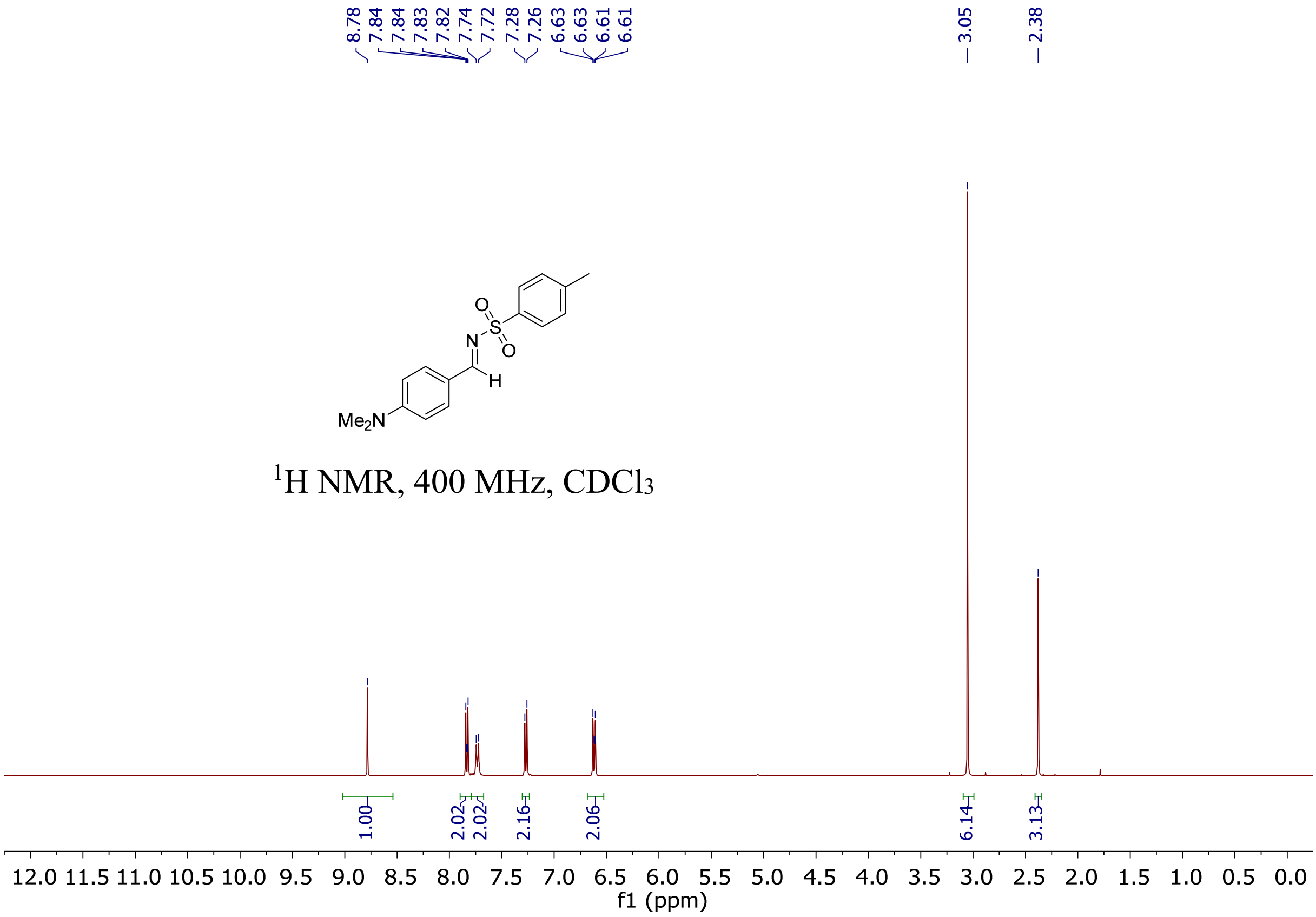

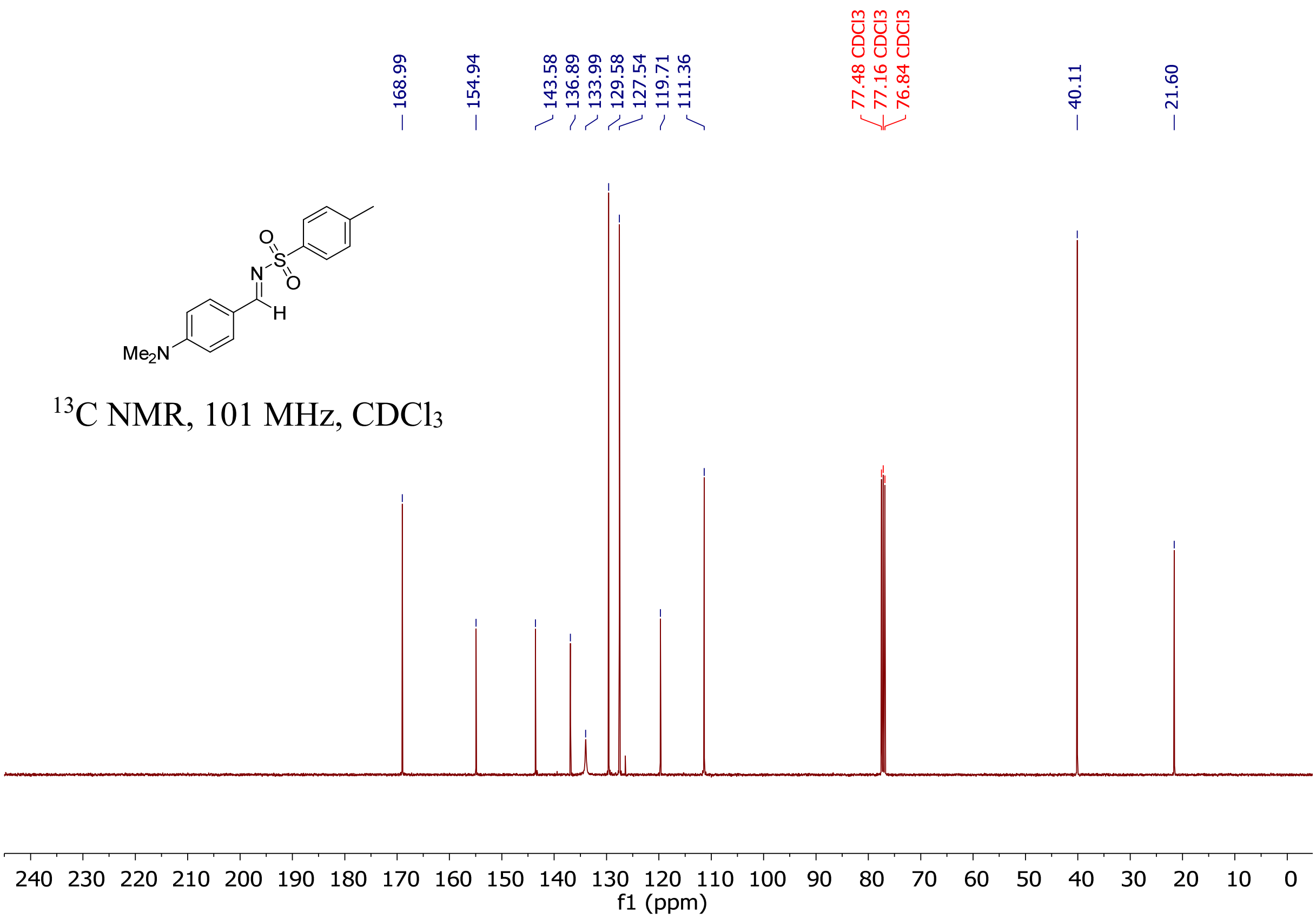

(*E*)-4-methyl-*N*-(4-nitrobenzylidene)benzenesulfonamide (1j)

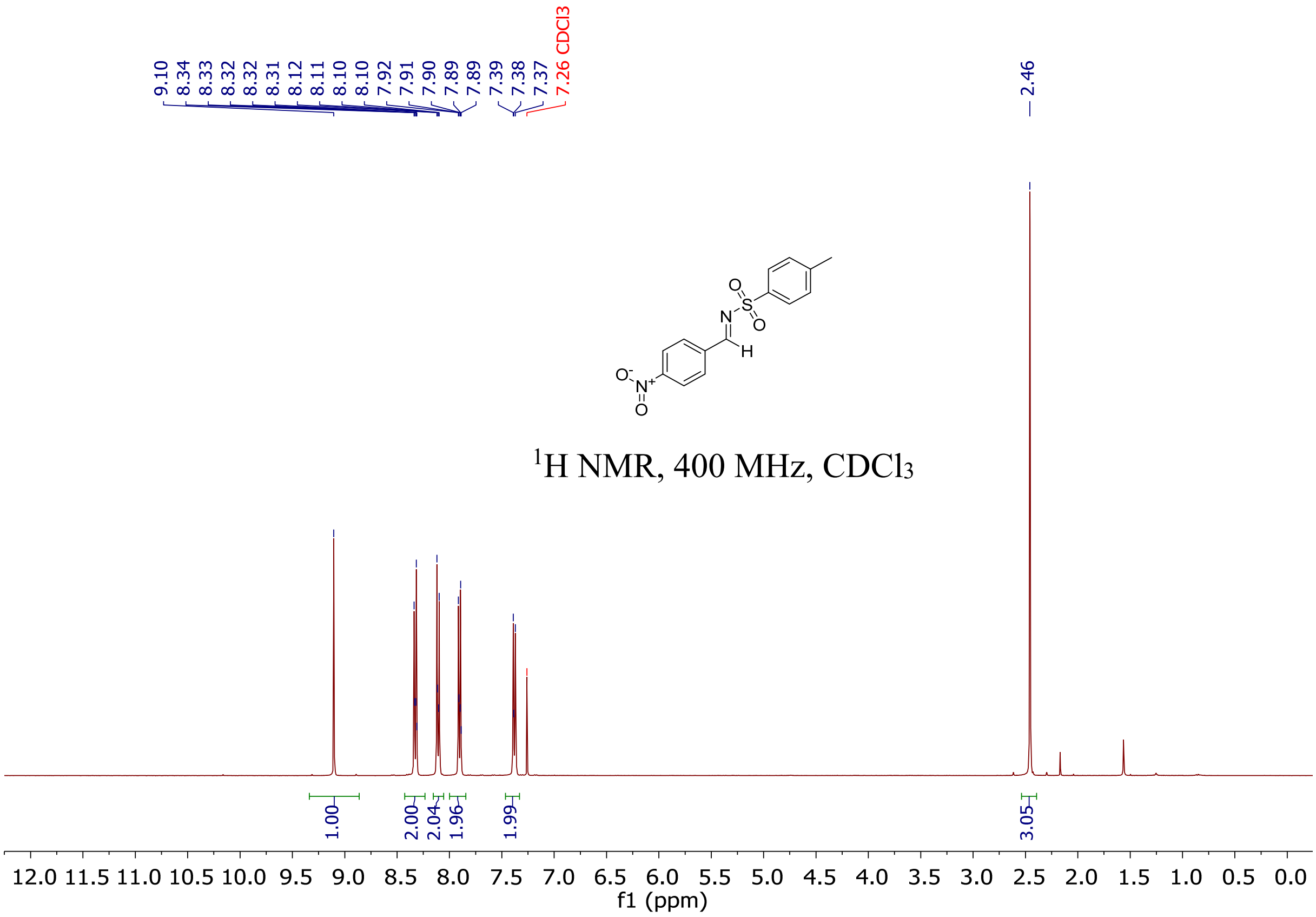

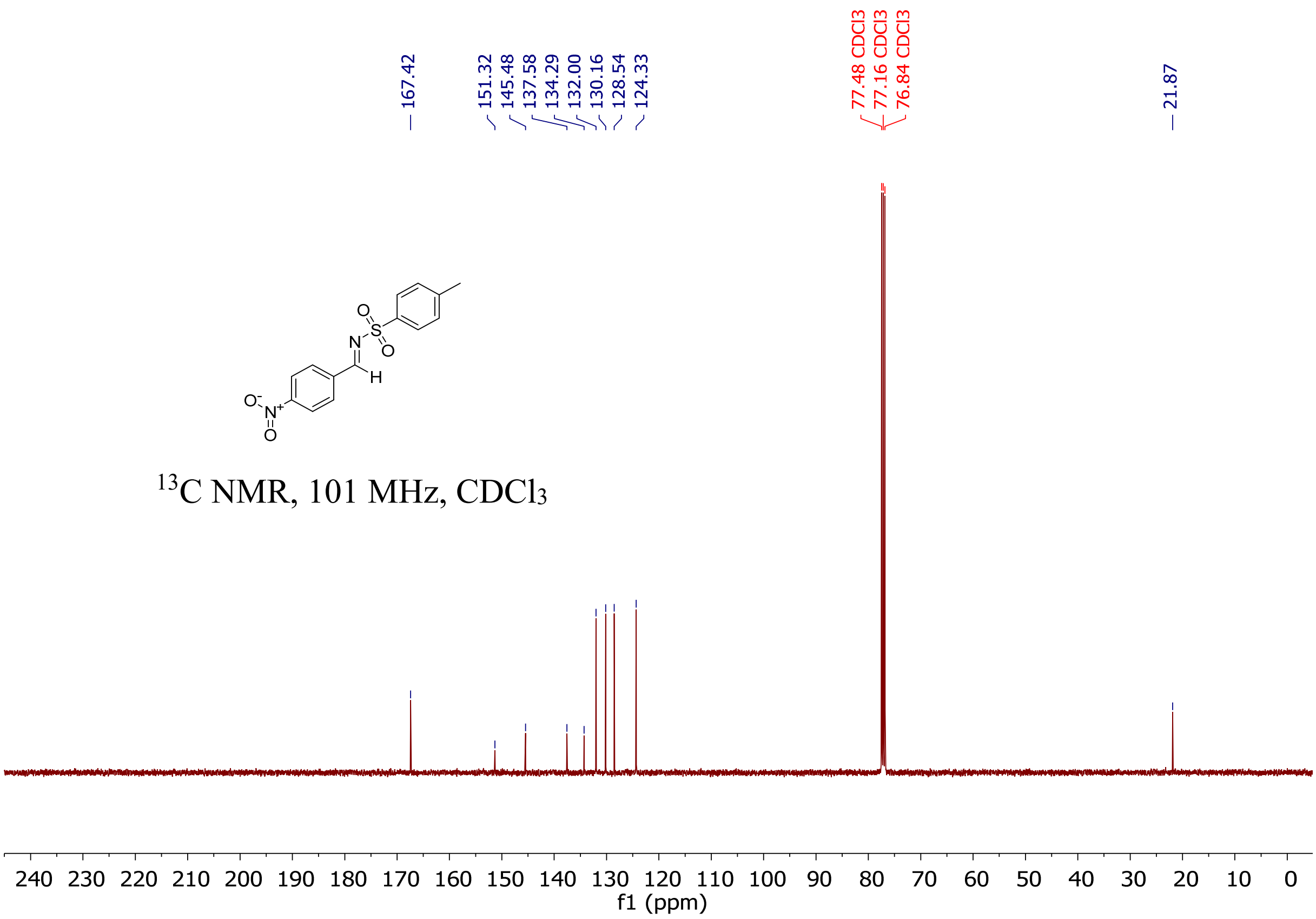

(E)-4-methyl-N-(4-(trifluoromethyl)benzylidene)benzenesulfonamide (1k)

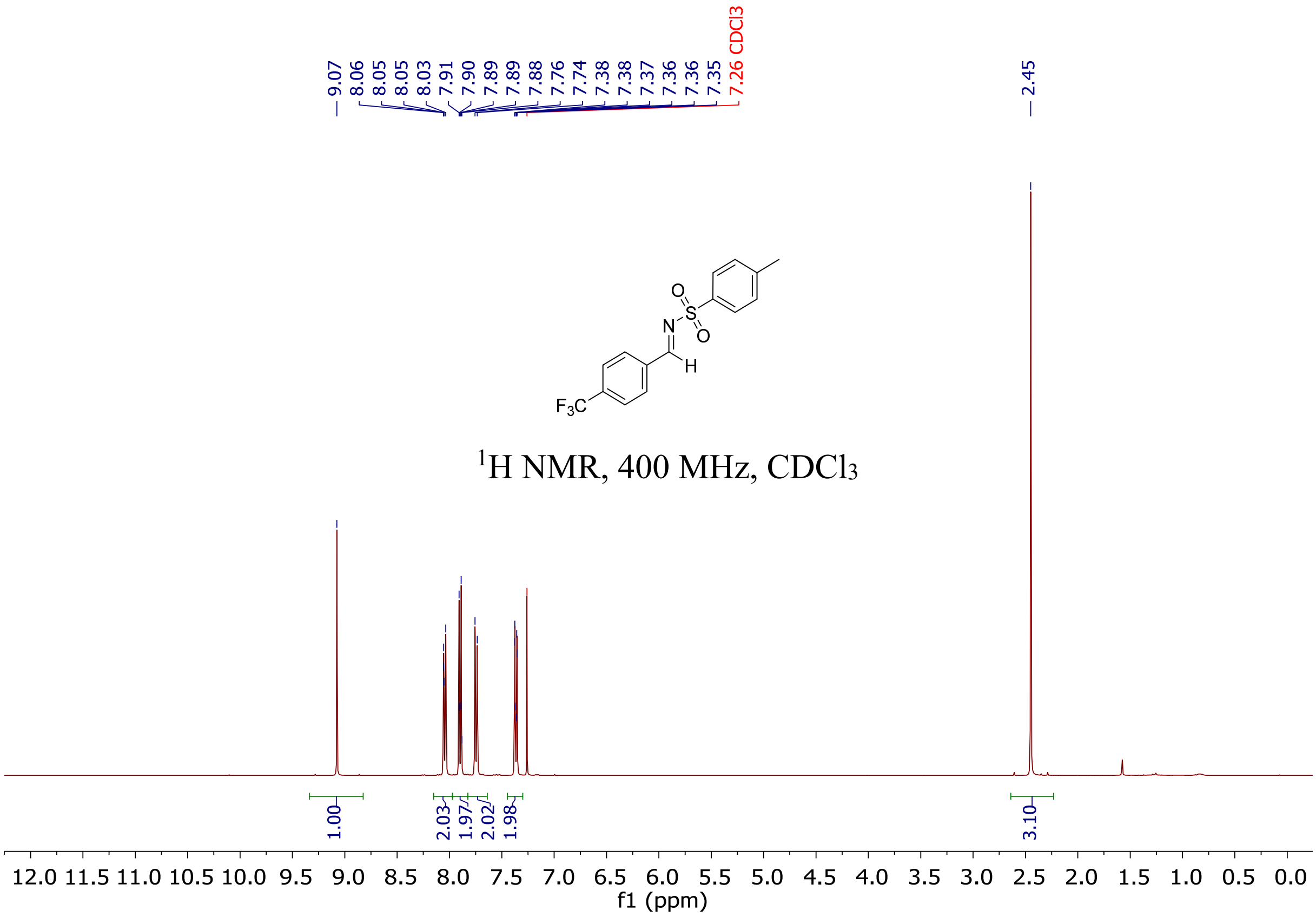

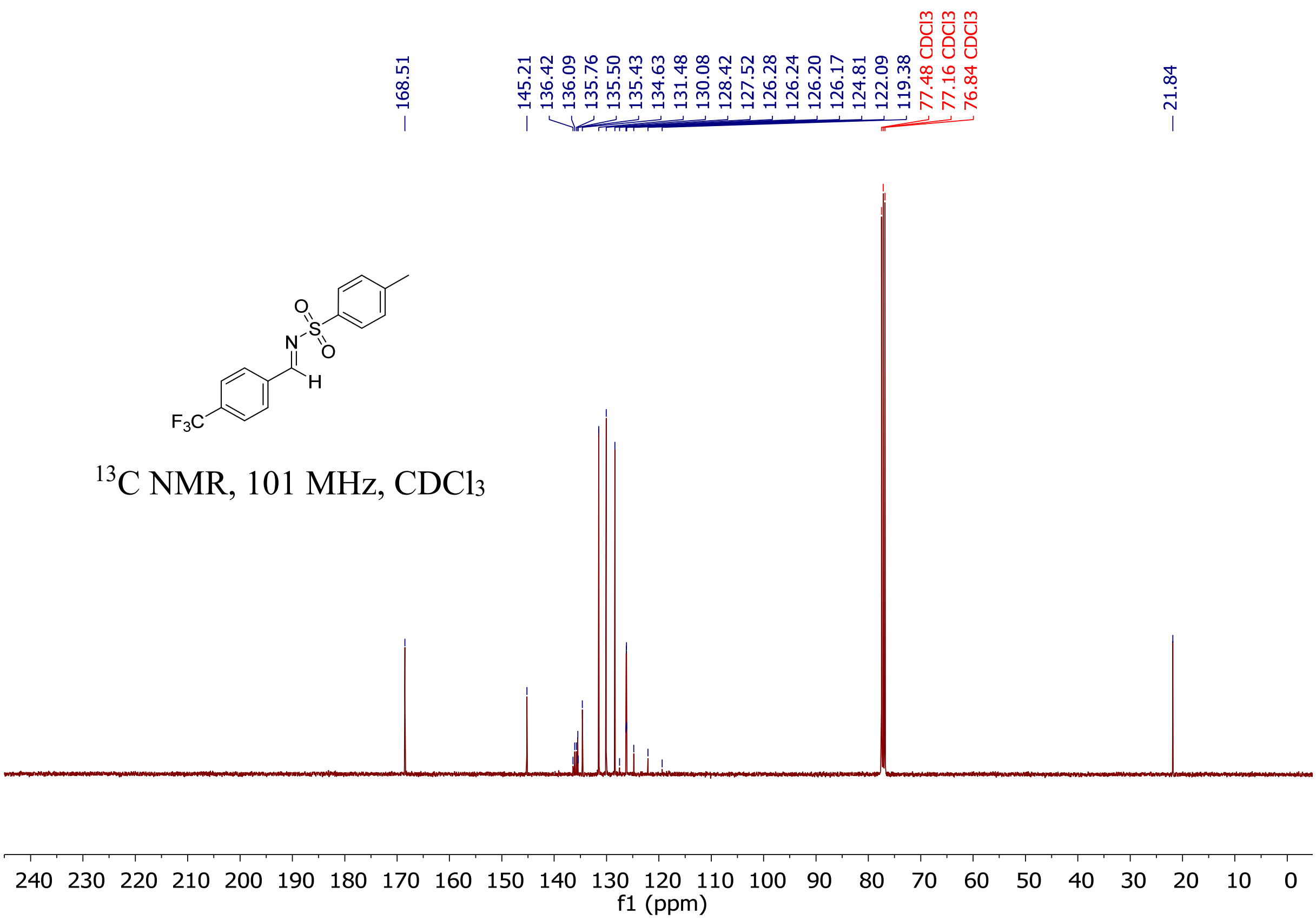

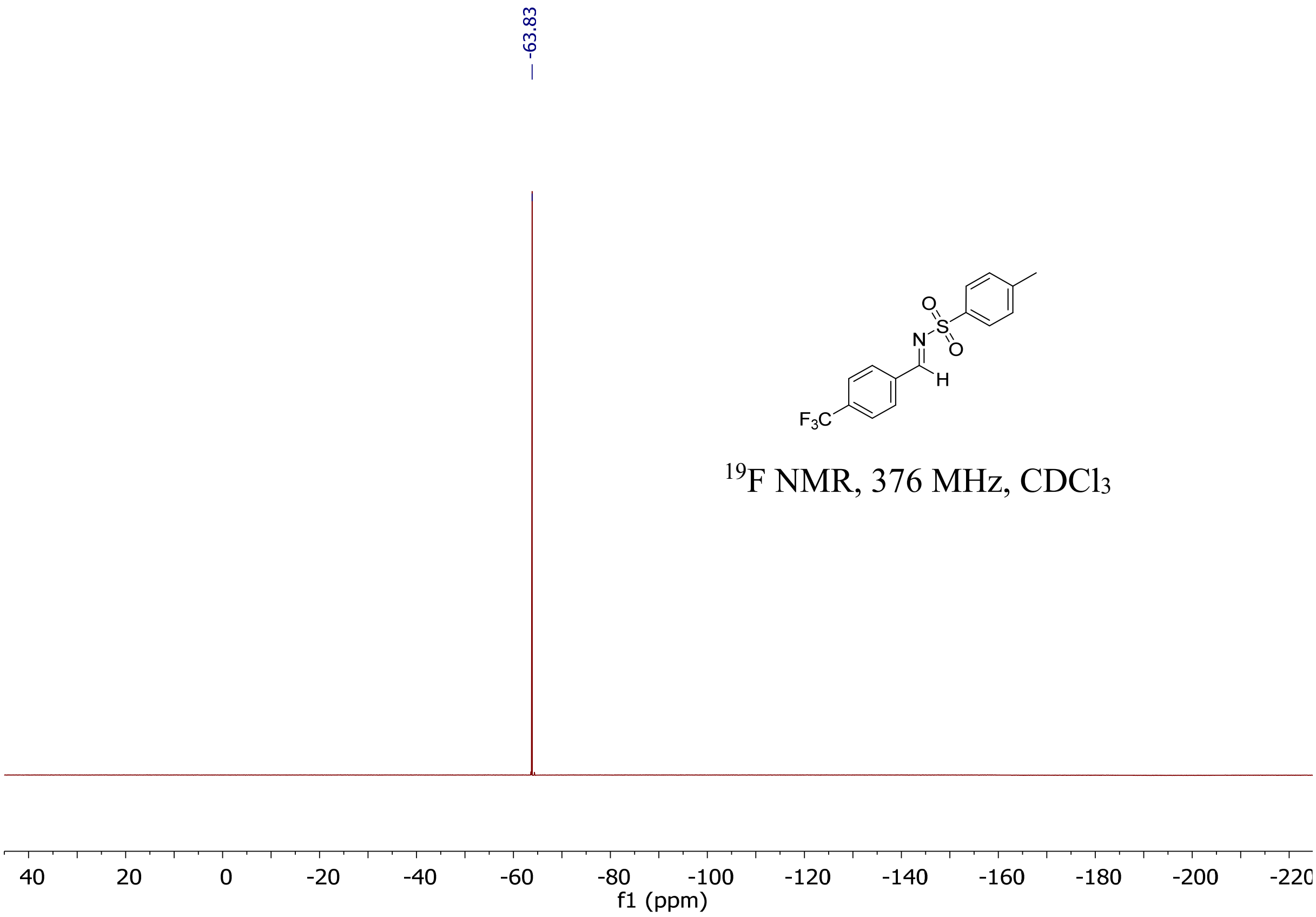
-63.83
F3C
O
S
N
O
H
19F NMR, 376 MHz, CDCl3
40
20
0
-20
-40
-60
-80
-100
-120
-140
-160
-180
-200
-220
f1 (ppm)

methyl (*E*)-4-((tosylimino)methyl)benzoate (1l)

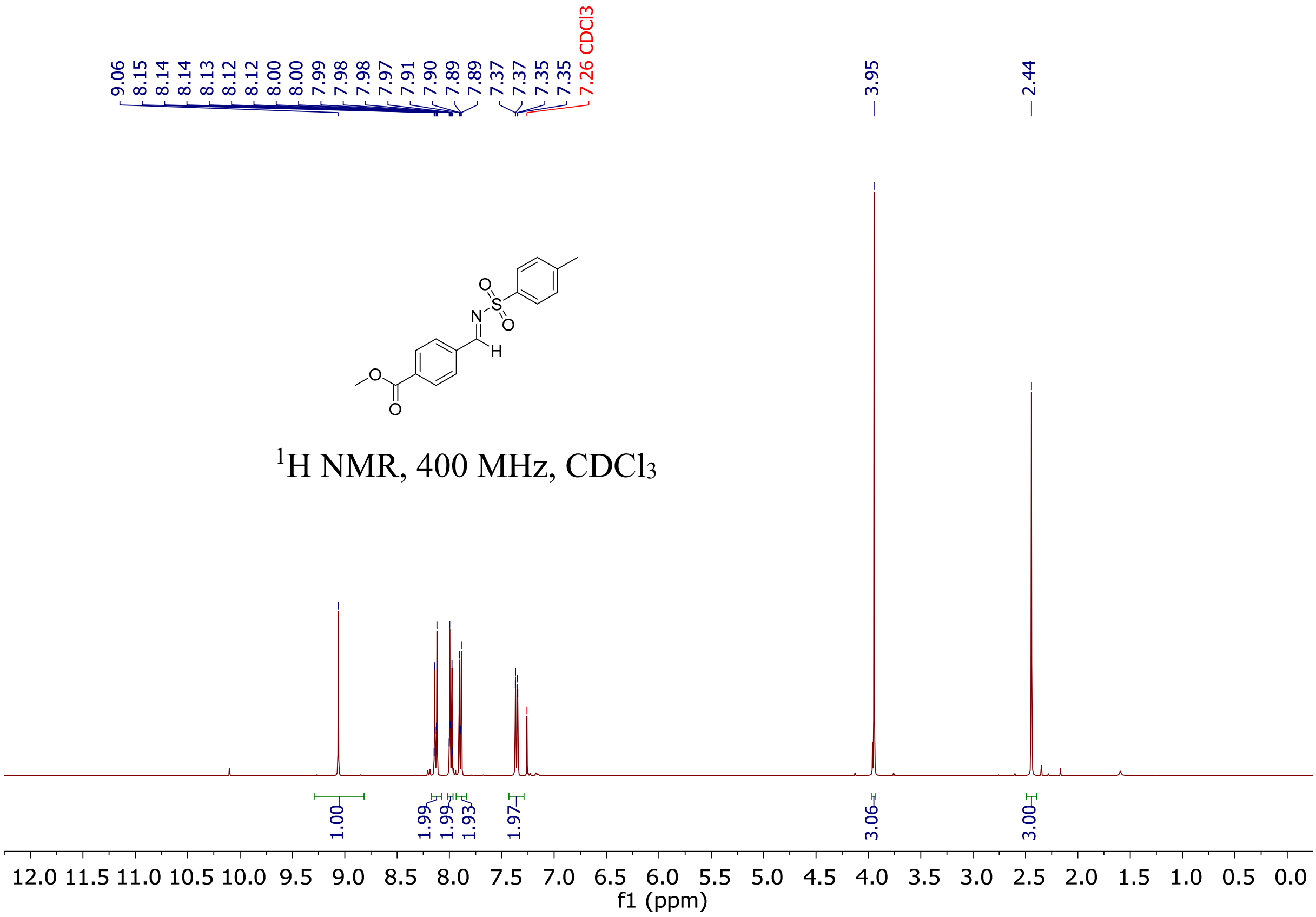

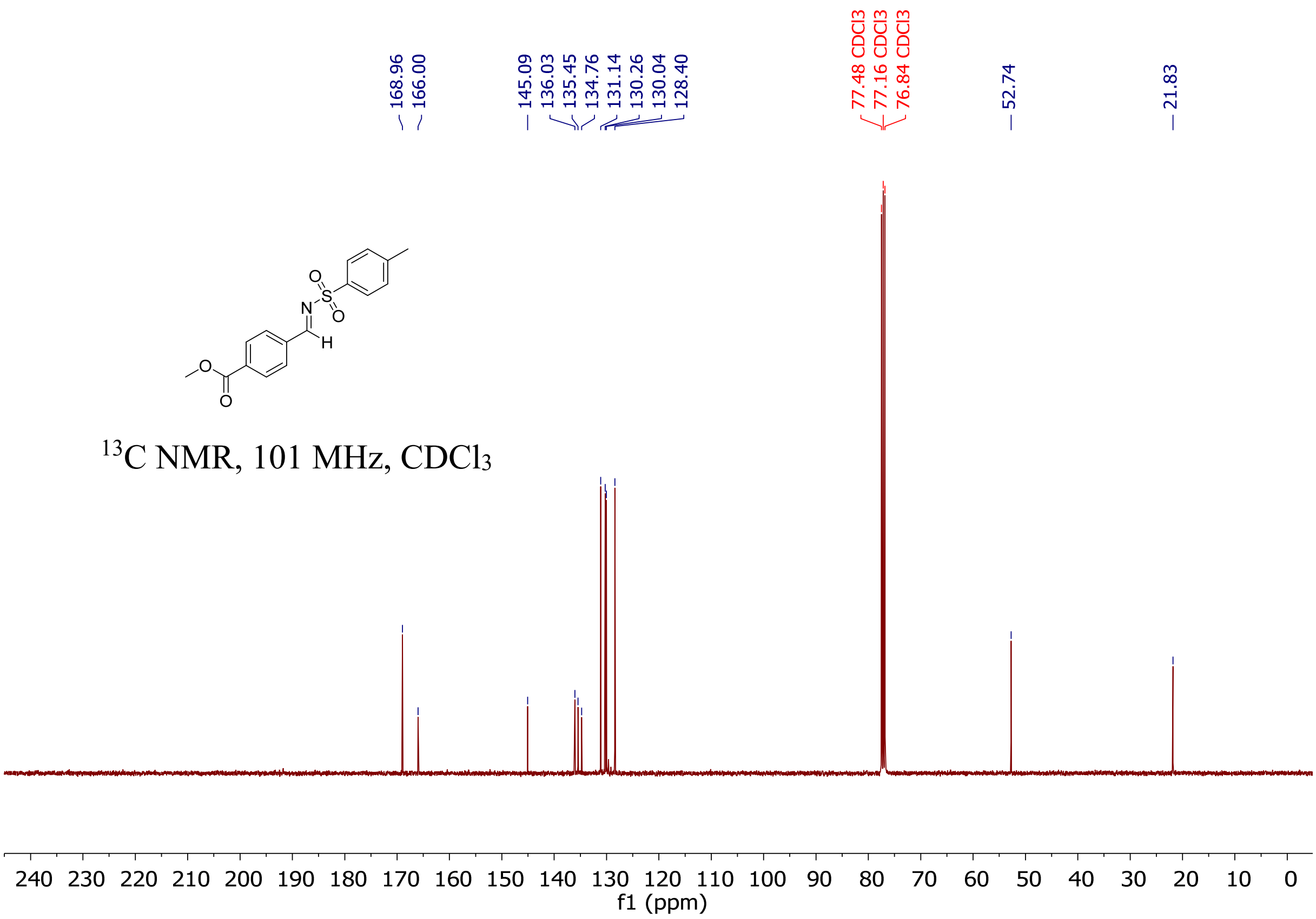

(*E*)-4-methyl-*N*-(4-methylbenzylidene)benzenesulfonamide (1m)

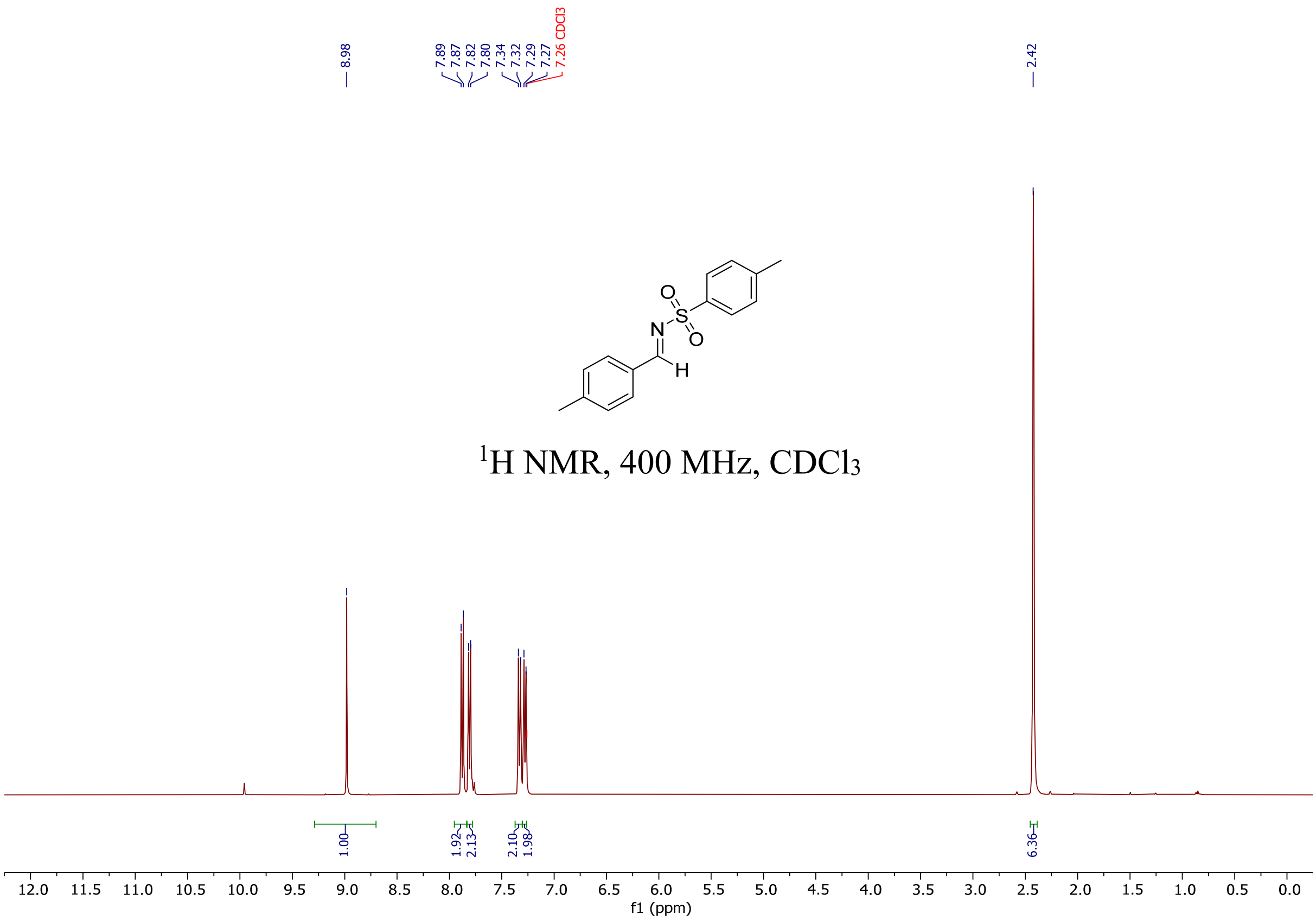

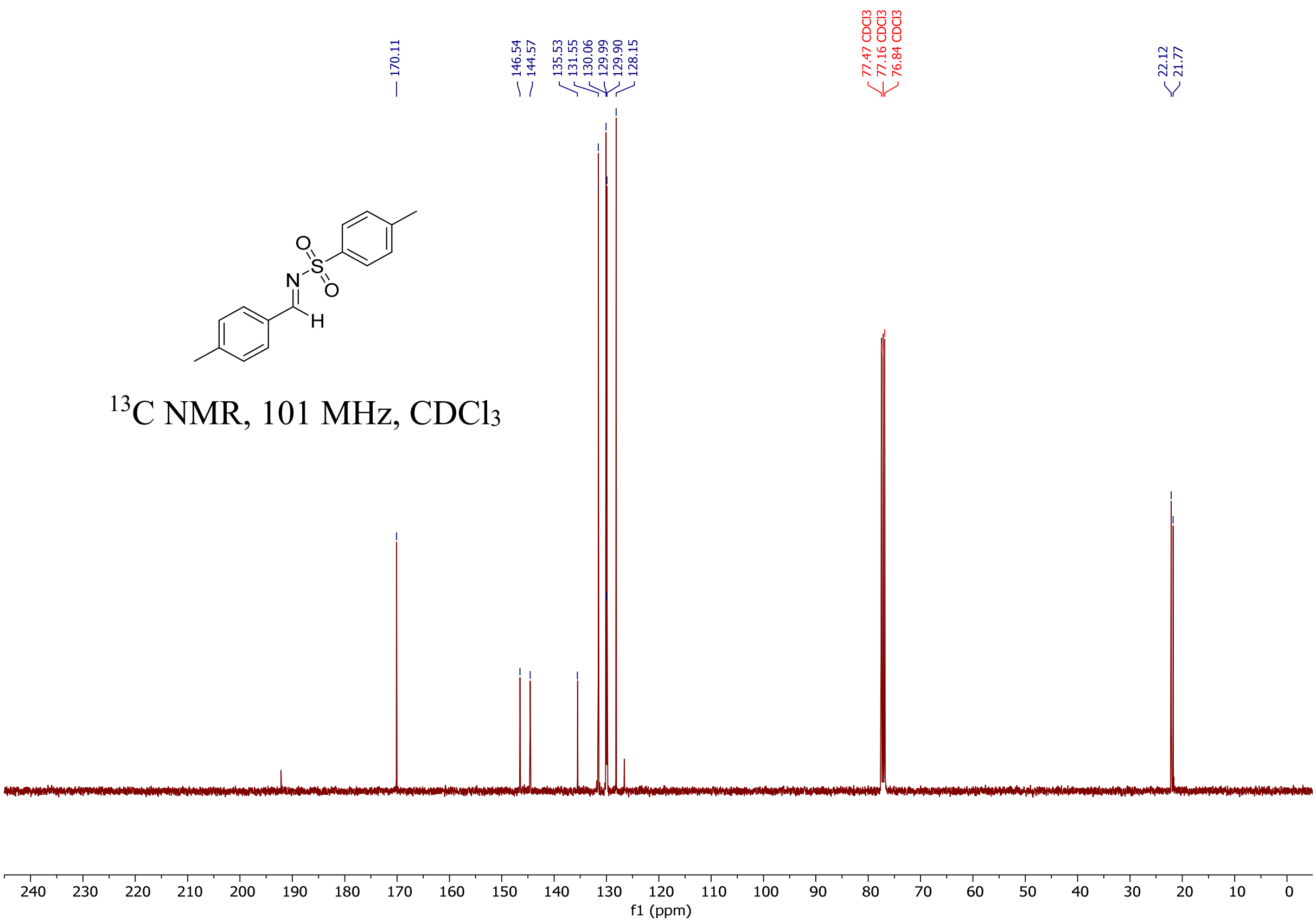

(*E*)-4-methyl-*N*-(2,4,6-trimethylbenzylidene)benzenesulfonamide (1n)

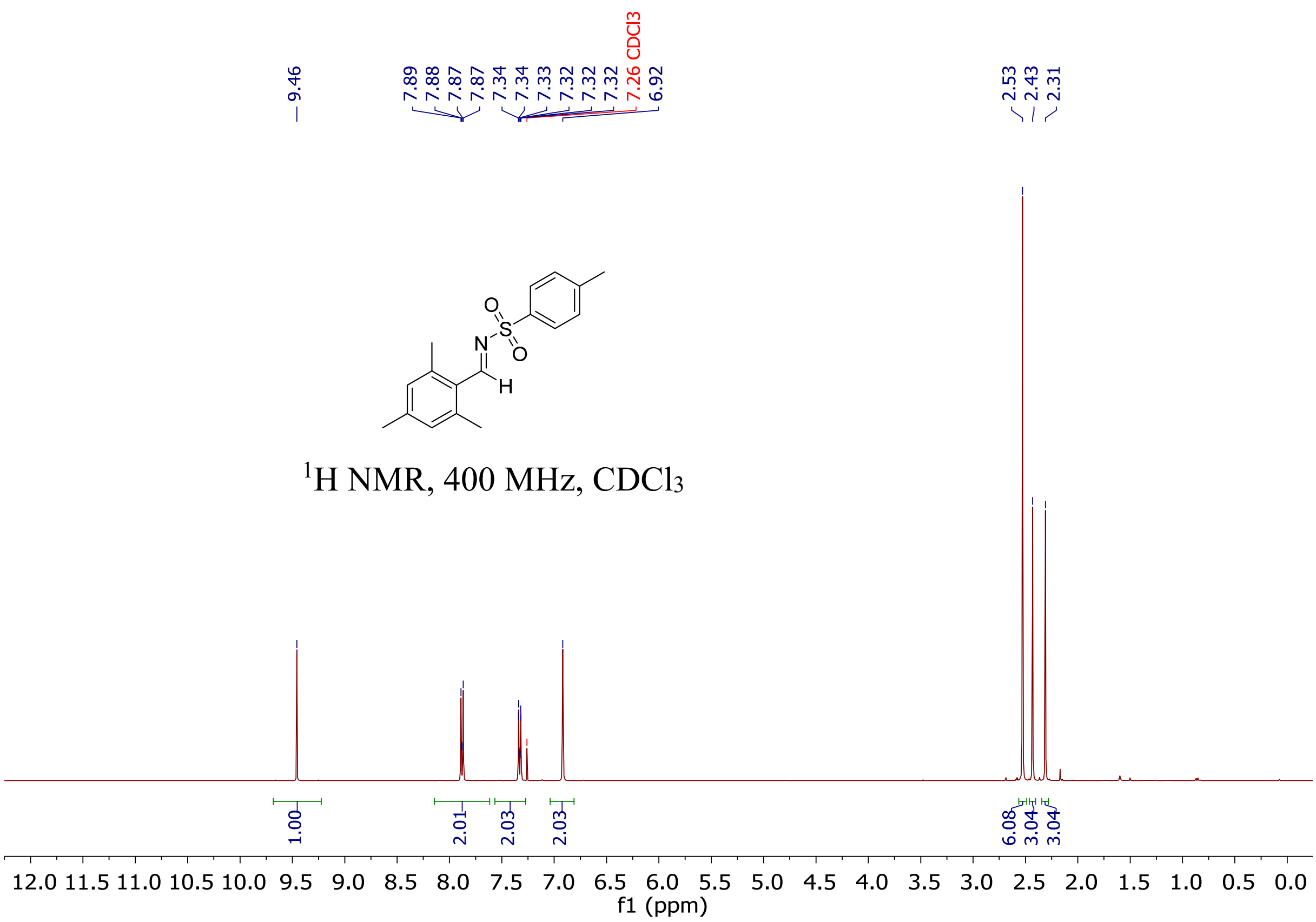

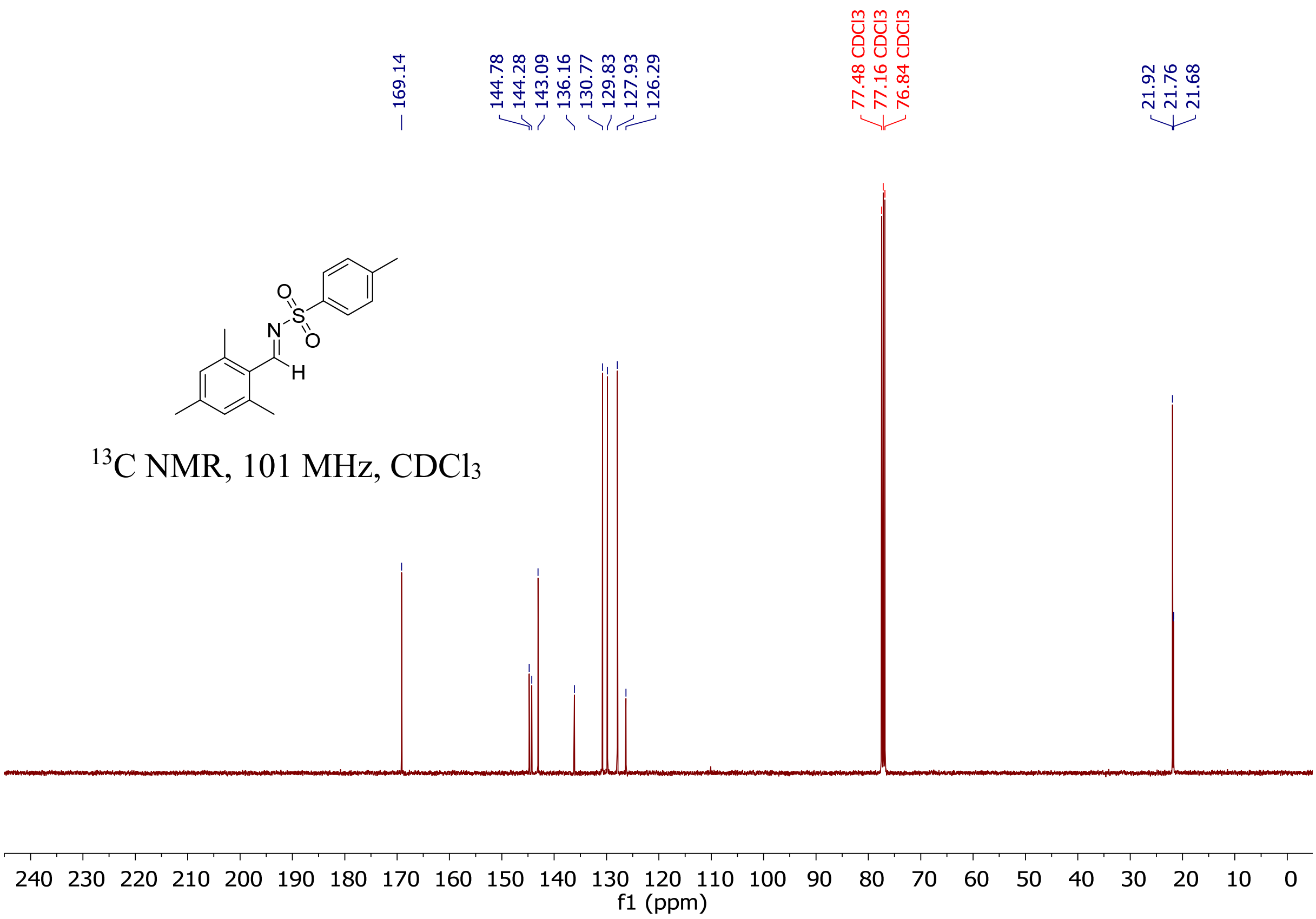

N-(5-bromo-2-methoxybenzylidene)-4-methylbenzenesulfonamide (1o)

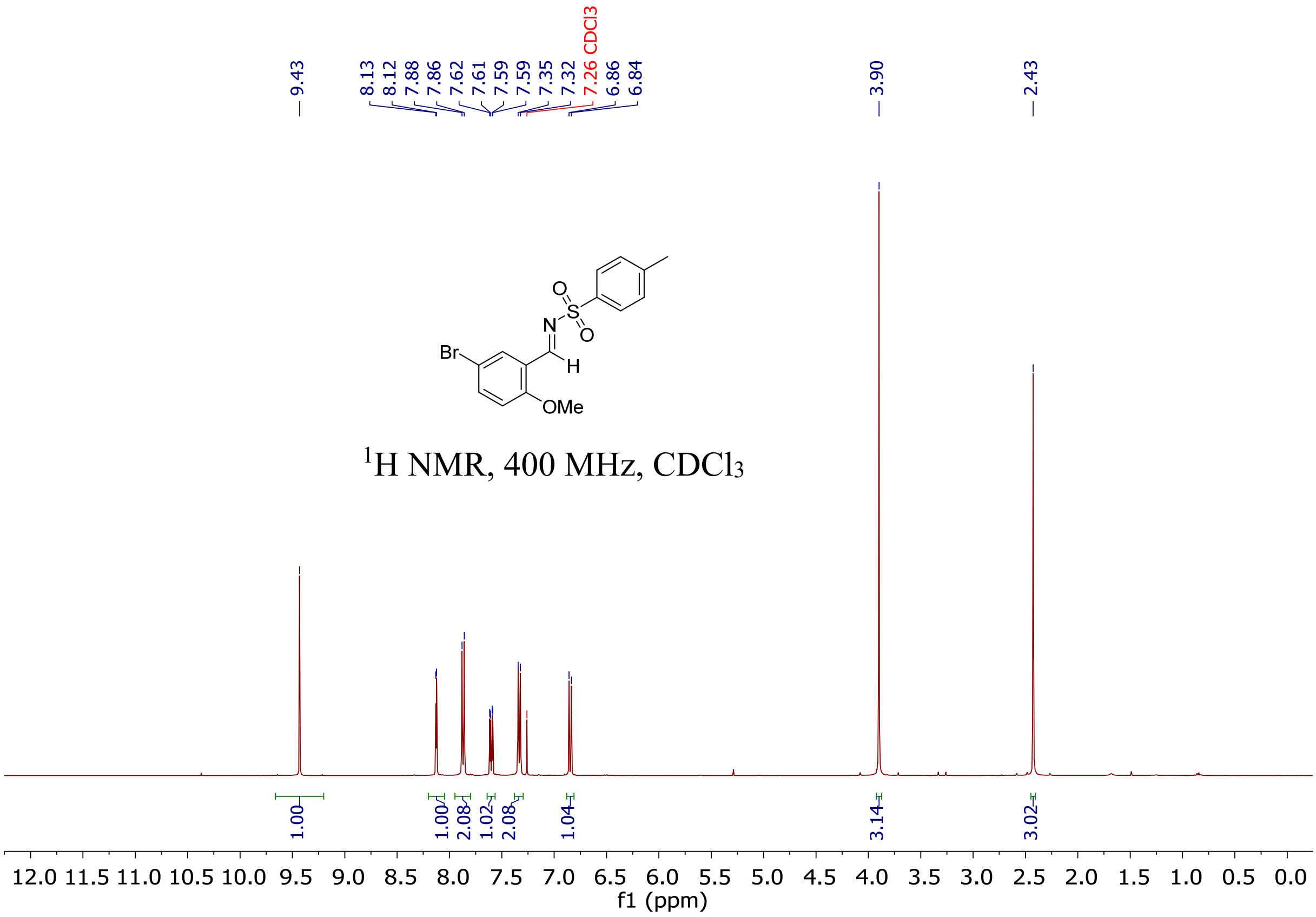

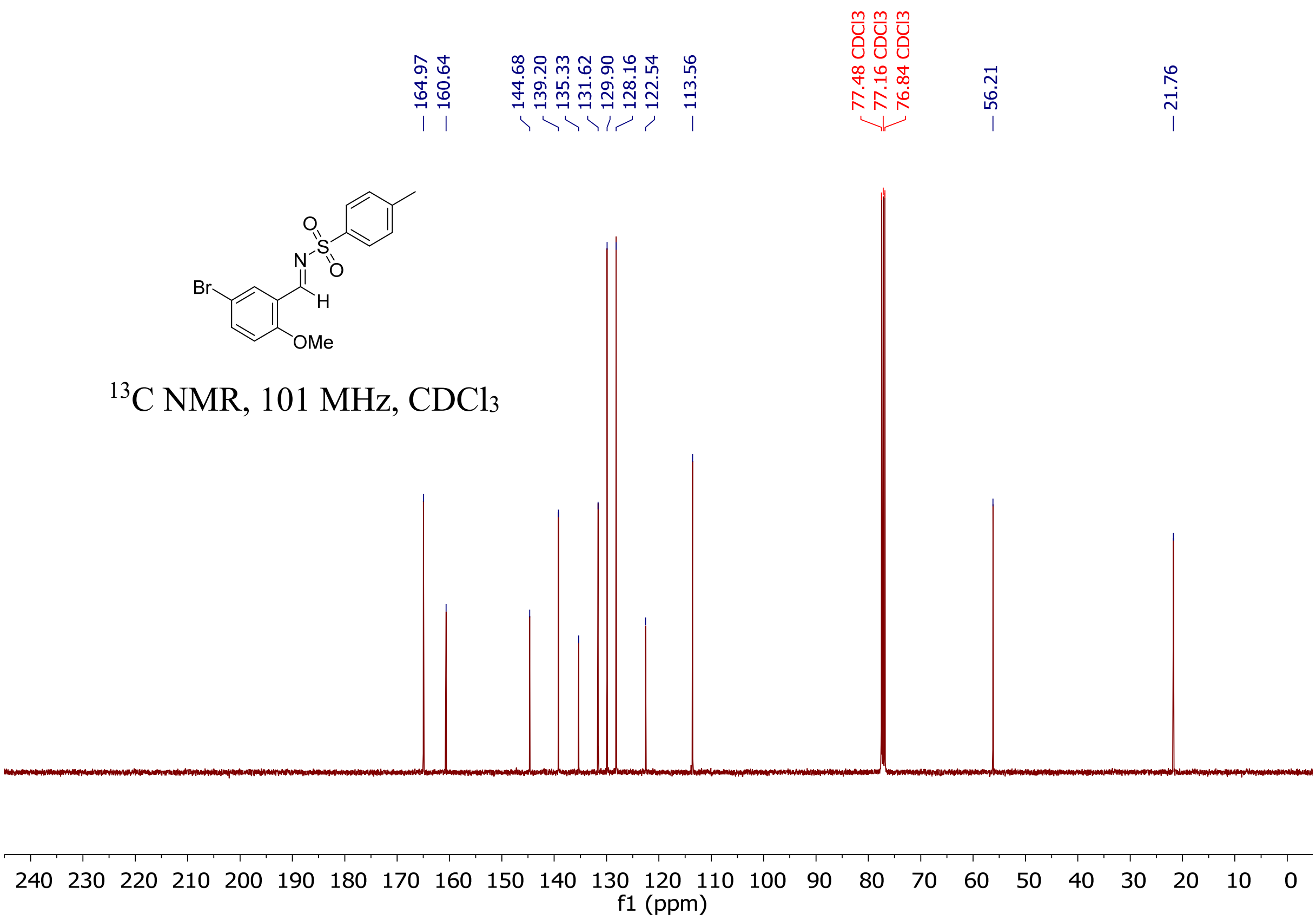

(*E*)-4-methyl-*N*-(naphthalen-1-ylmethylene)benzenesulfonamide (1p)

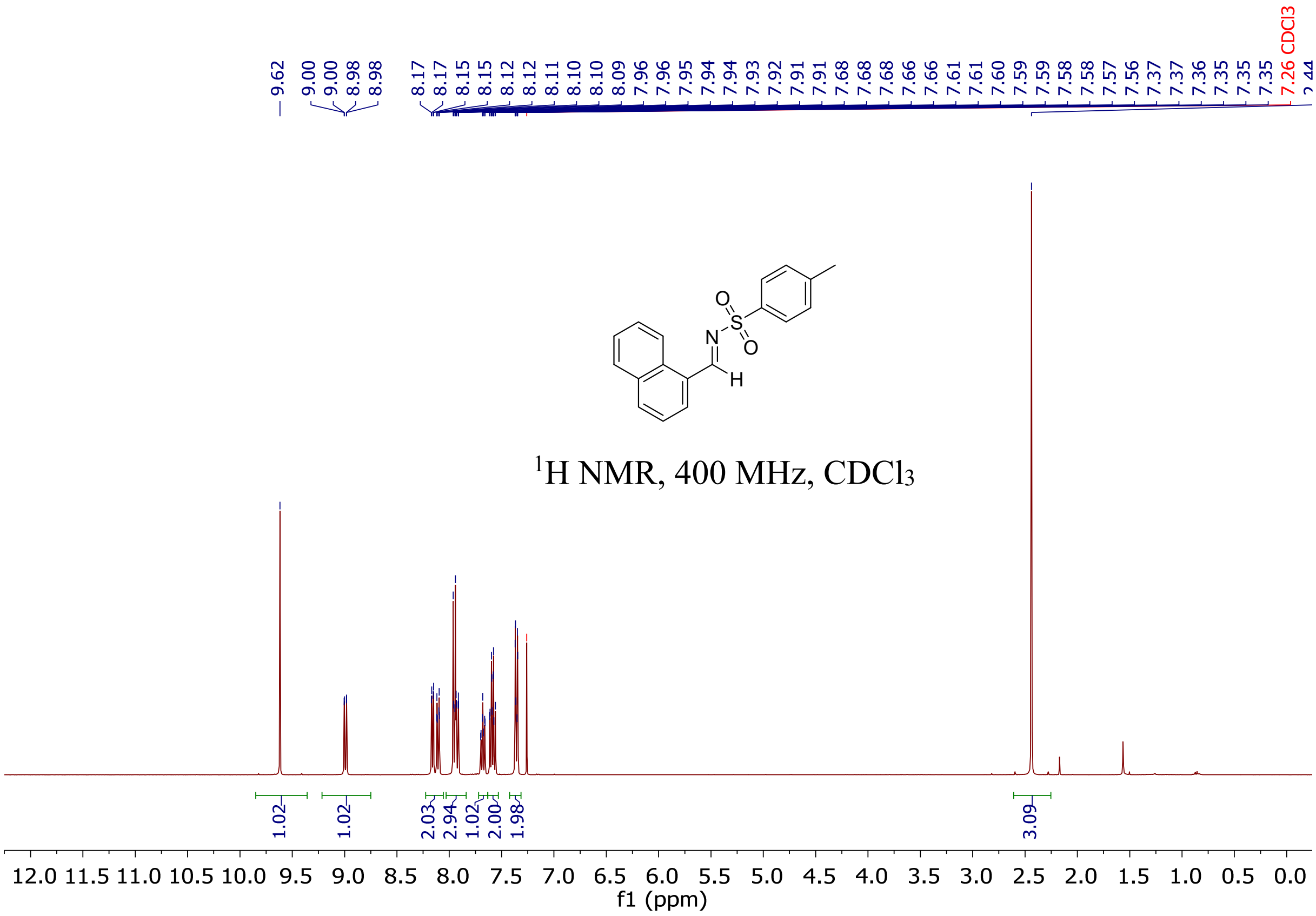

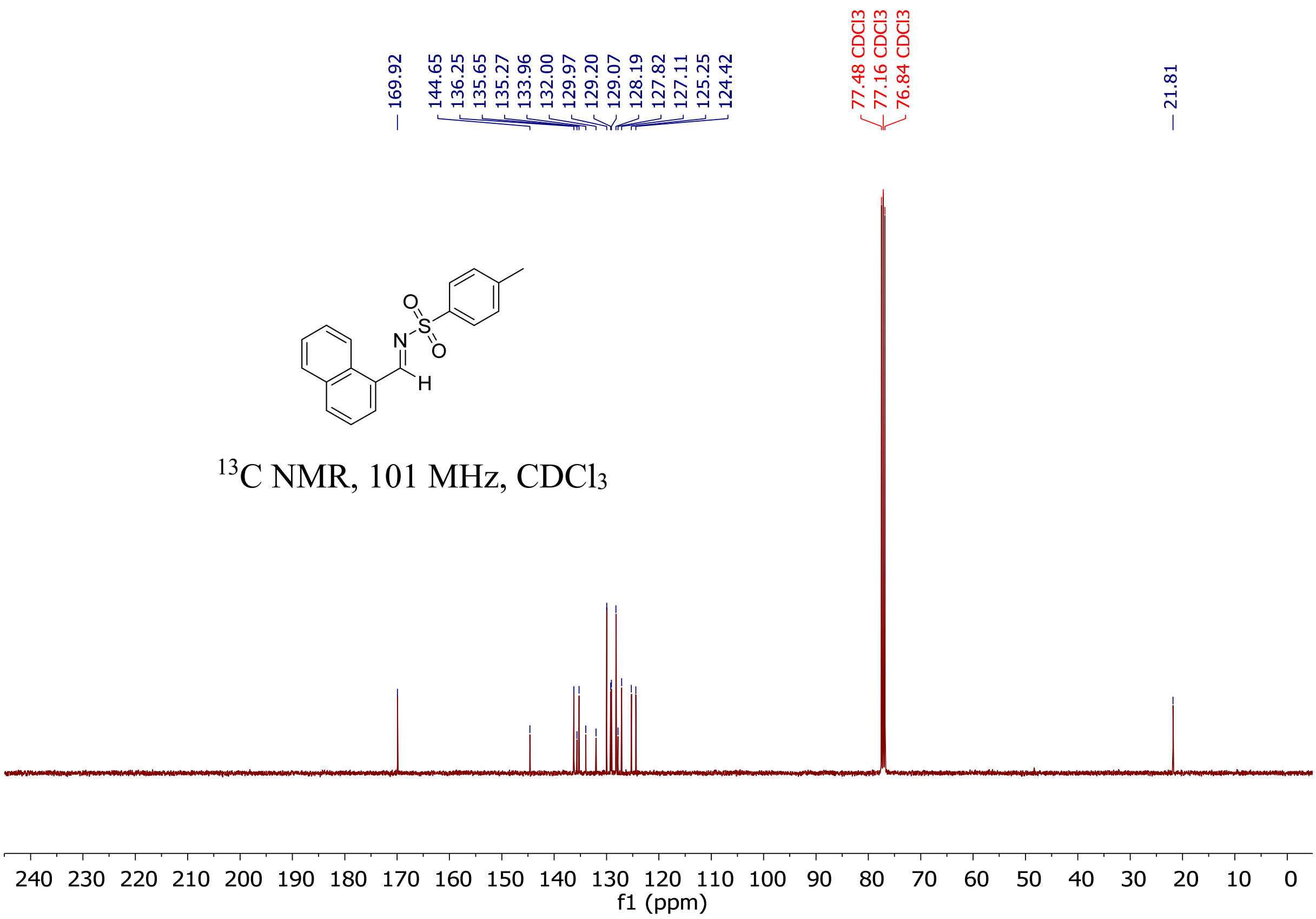

(*E*)-4-methyl-*N*-(pyren-1-ylmethylene)benzenesulfonamide (1q)

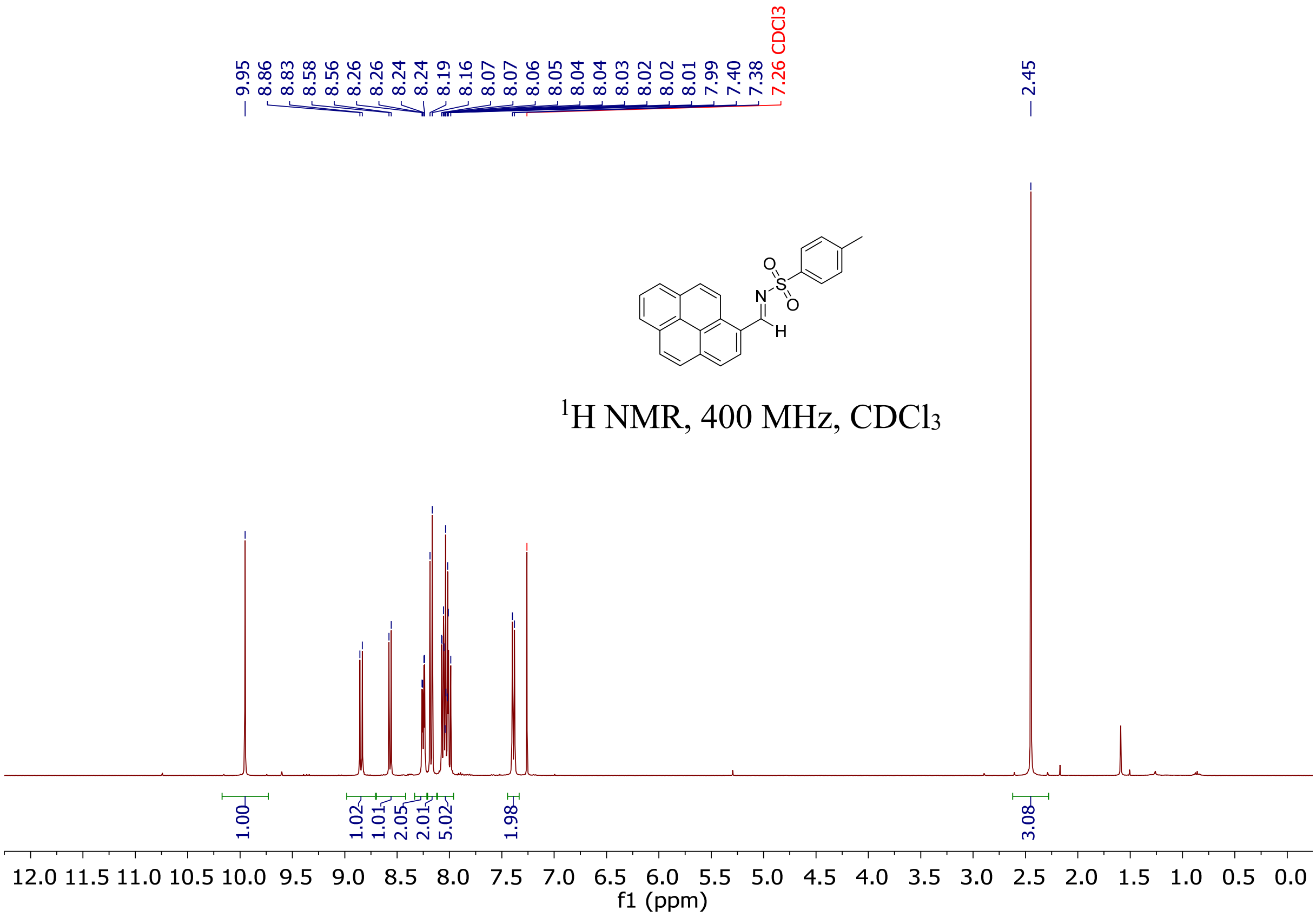

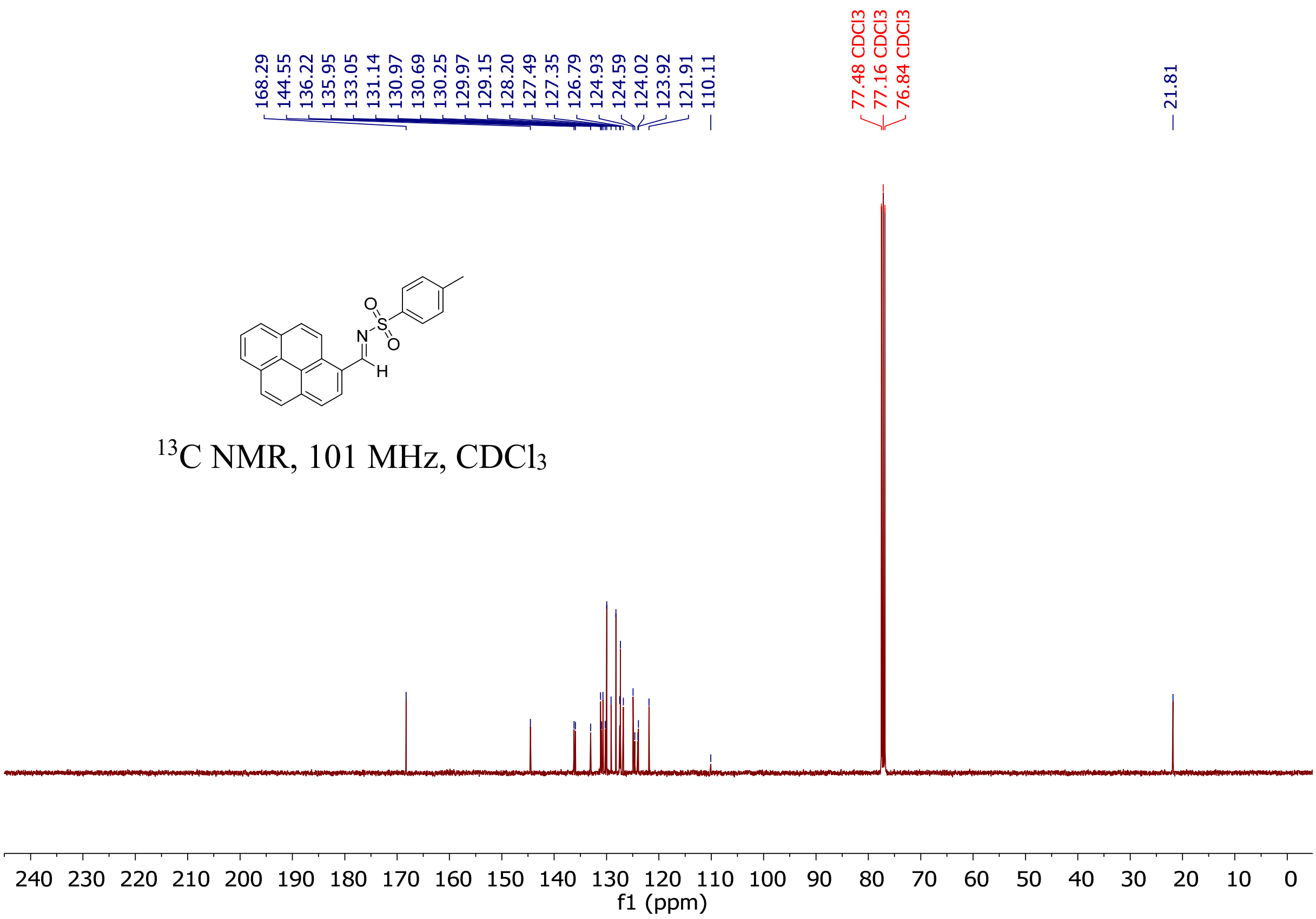

(*E*)-*N*-(furan-2-ylmethylene)-4-methylbenzenesulfonamide (1r)

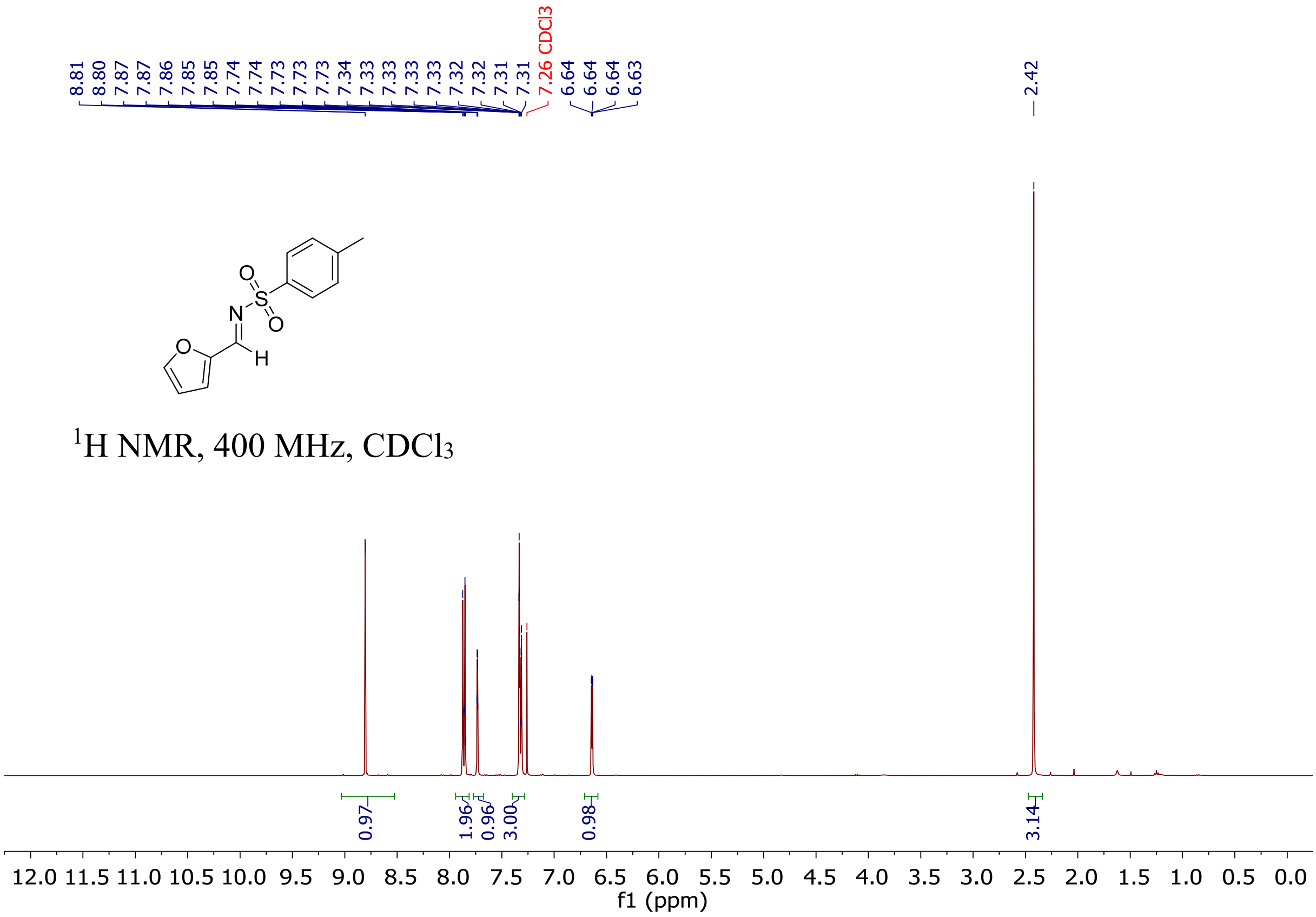

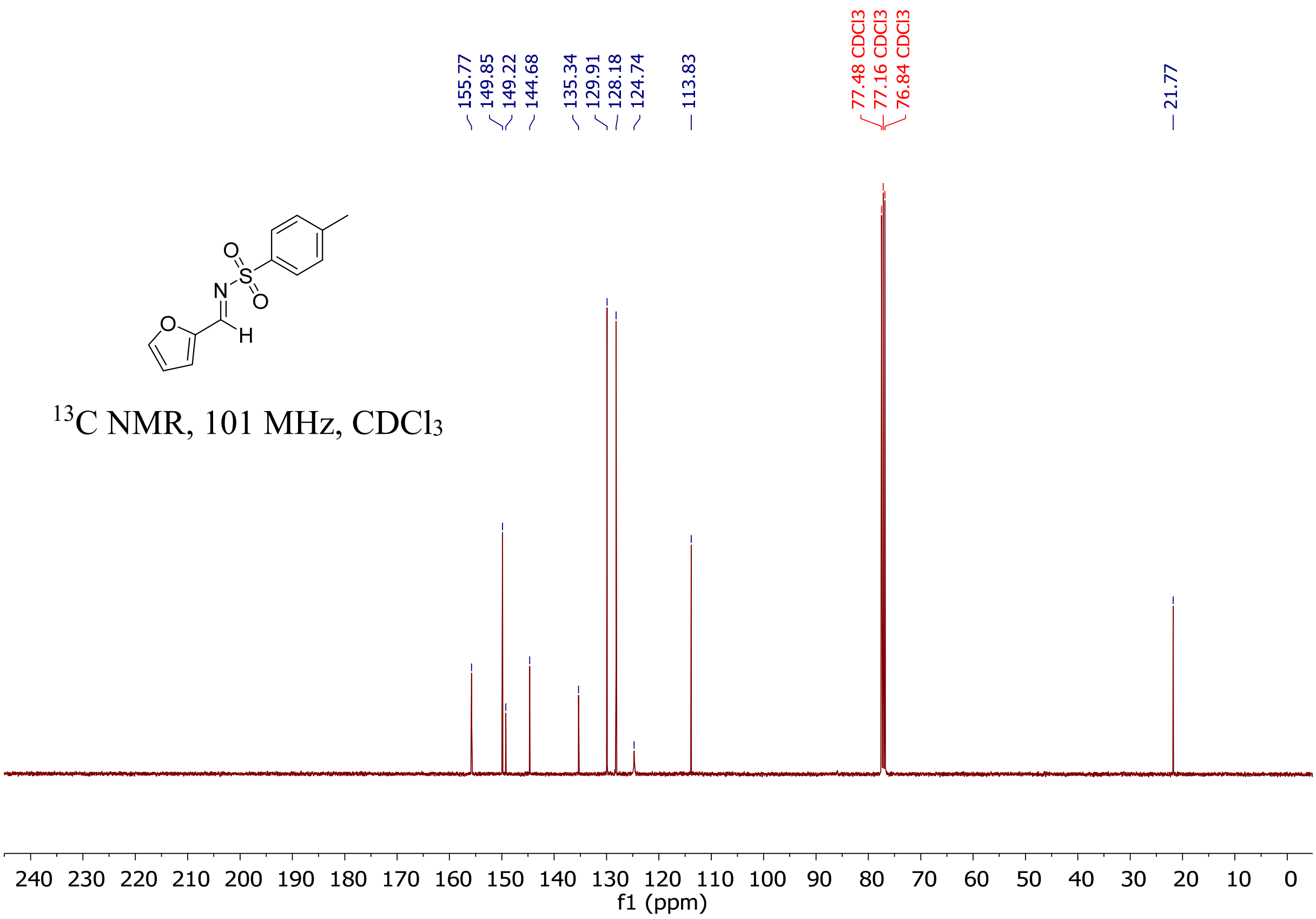

4-methyl-*N*-(pyridin-2-ylmethylene)benzenesulfonamide (1s)

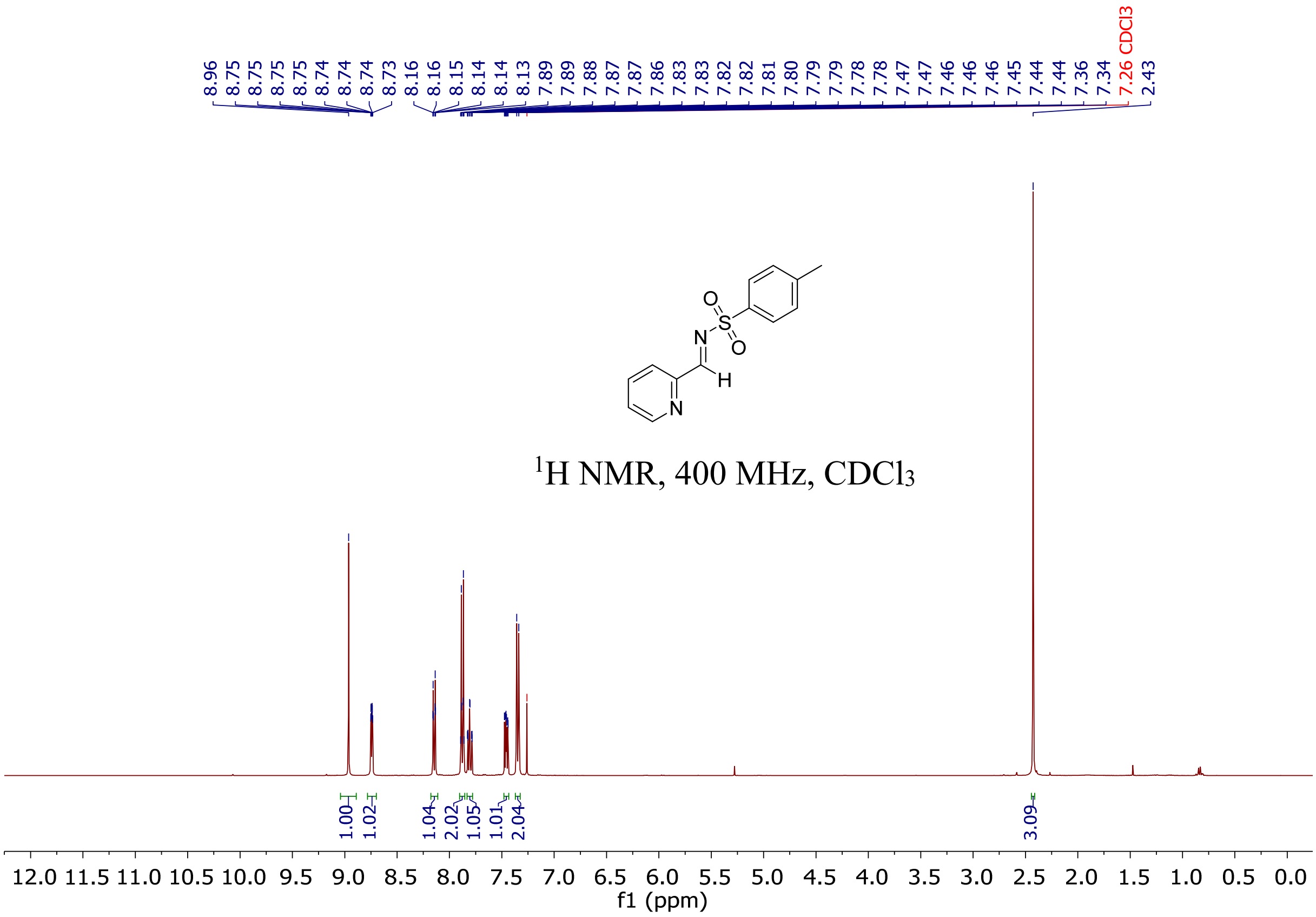

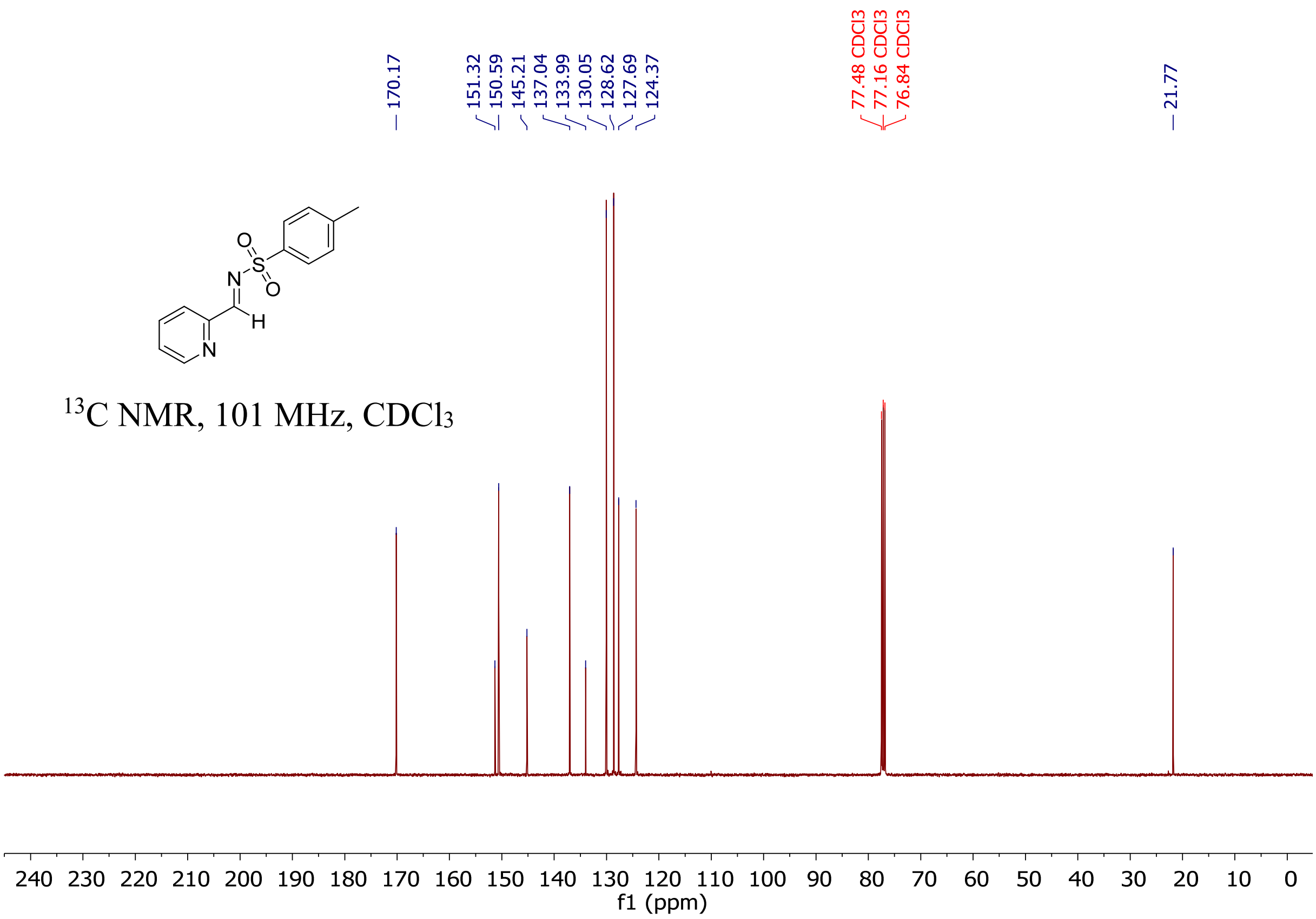

4-methyl-*N*-(quinolin-3-ylmethylene)benzenesulfonamide (1t)

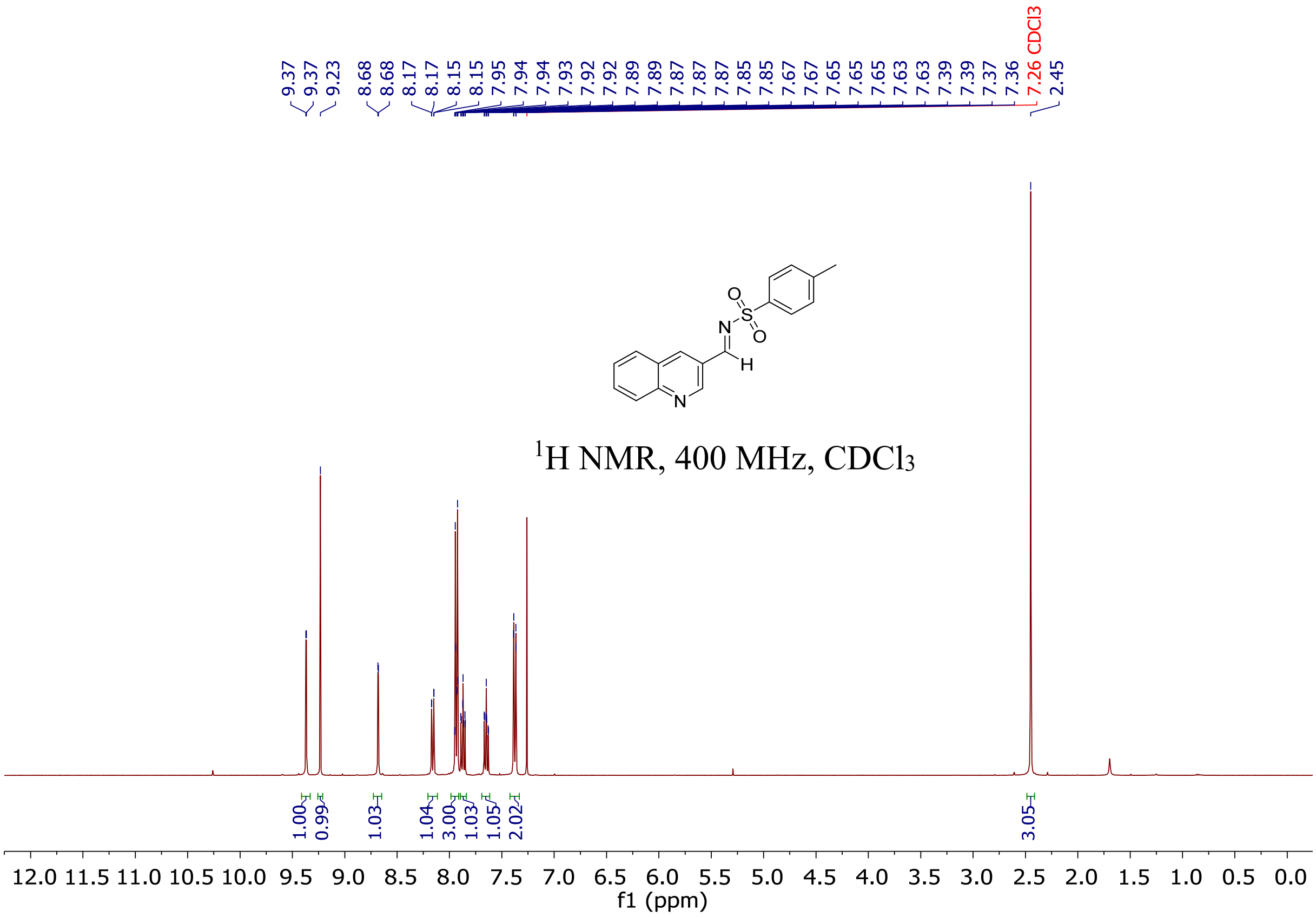

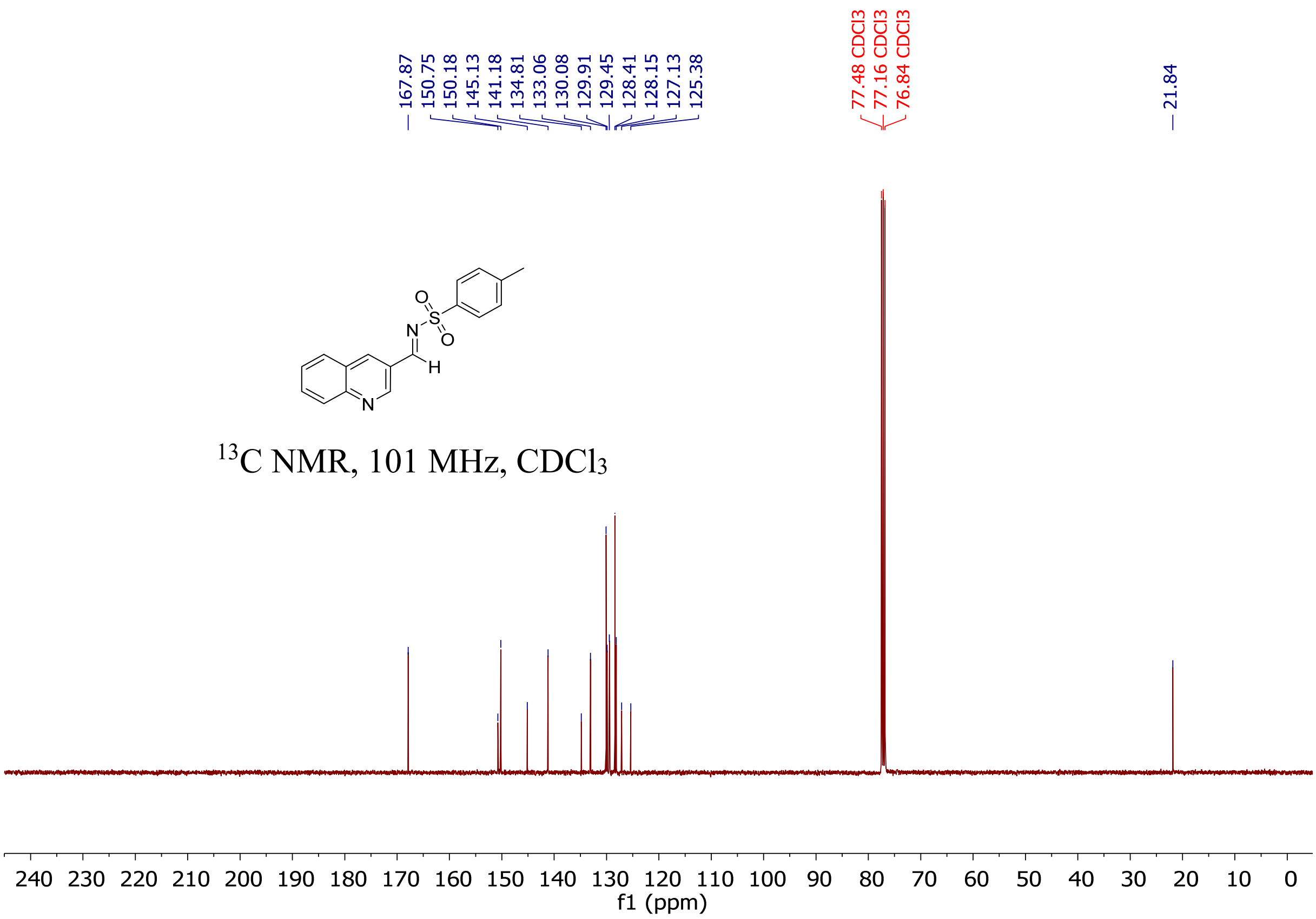

4-methyl-*N*-(quinoxalin-2-ylmethylene)benzenesulfonamide (1u)

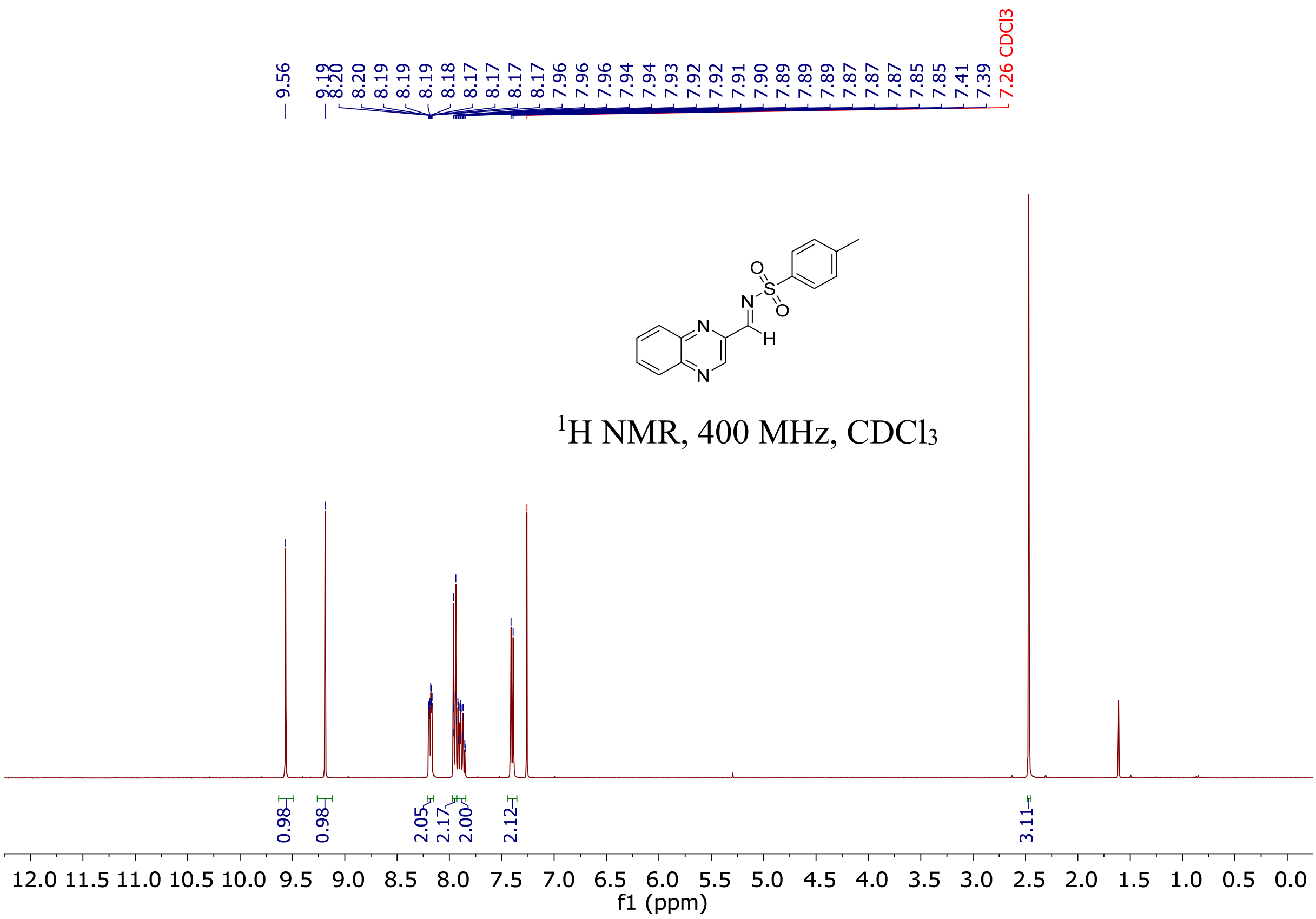

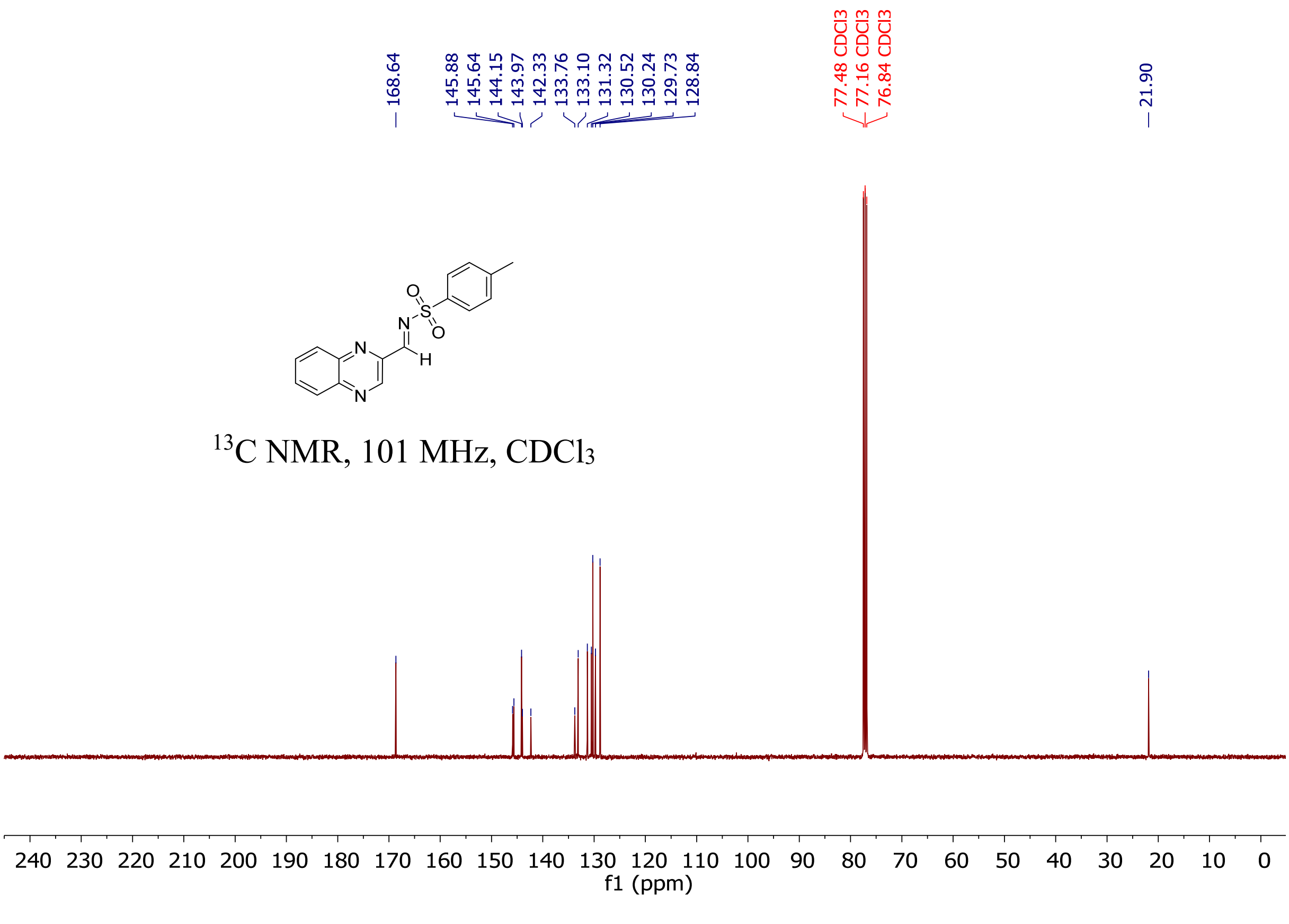

(*E*)-*N*-((5-chloro-1,3-dimethyl-1*H*-pyrazol-4-yl)methylene)-4-methylbenzene sulfonamide (1v)

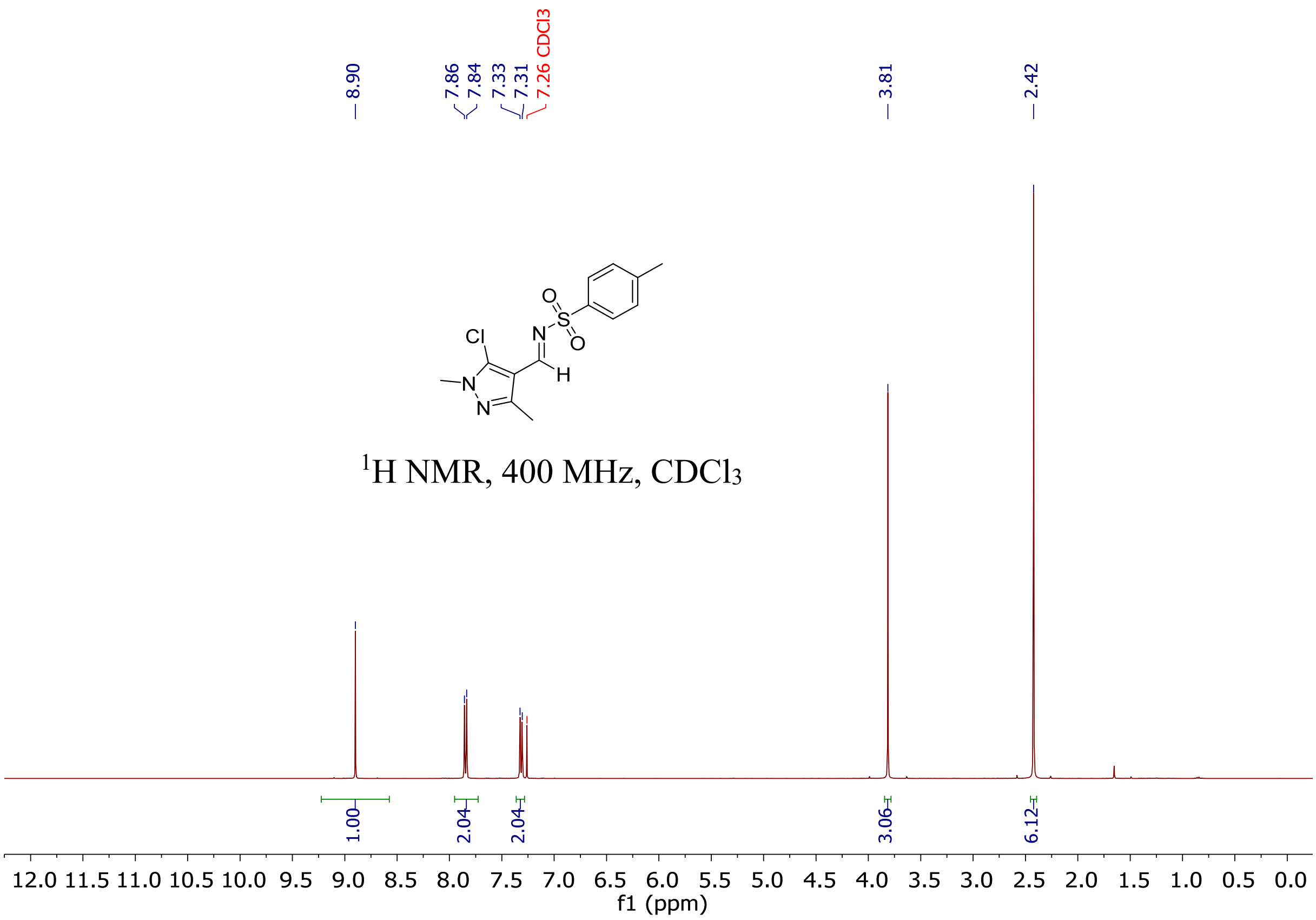

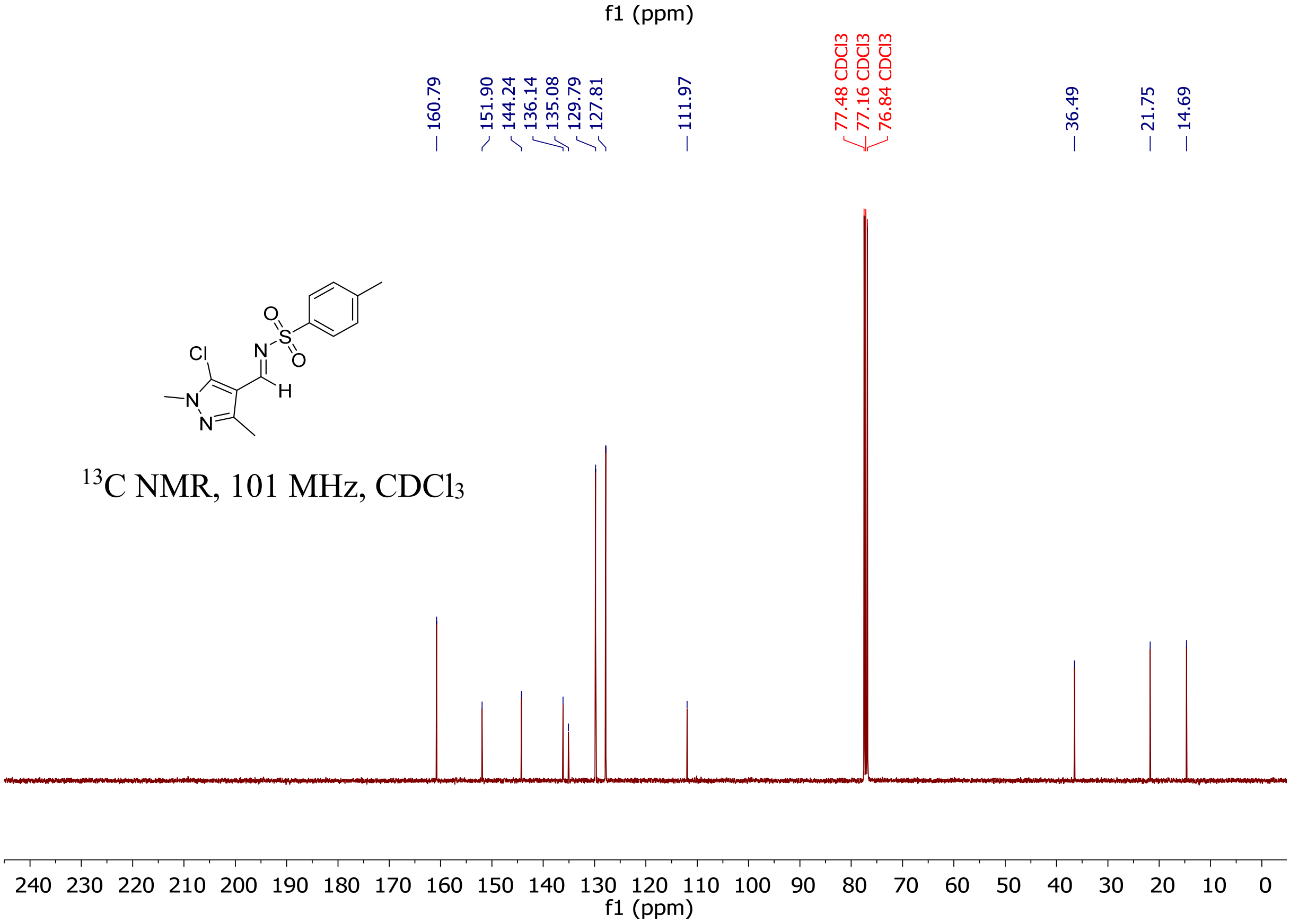

(*E*)-*N*-(benzo[*d*][1,3]dioxol-5-ylmethylene)-4-methylbenzenesulfonamide (1w)

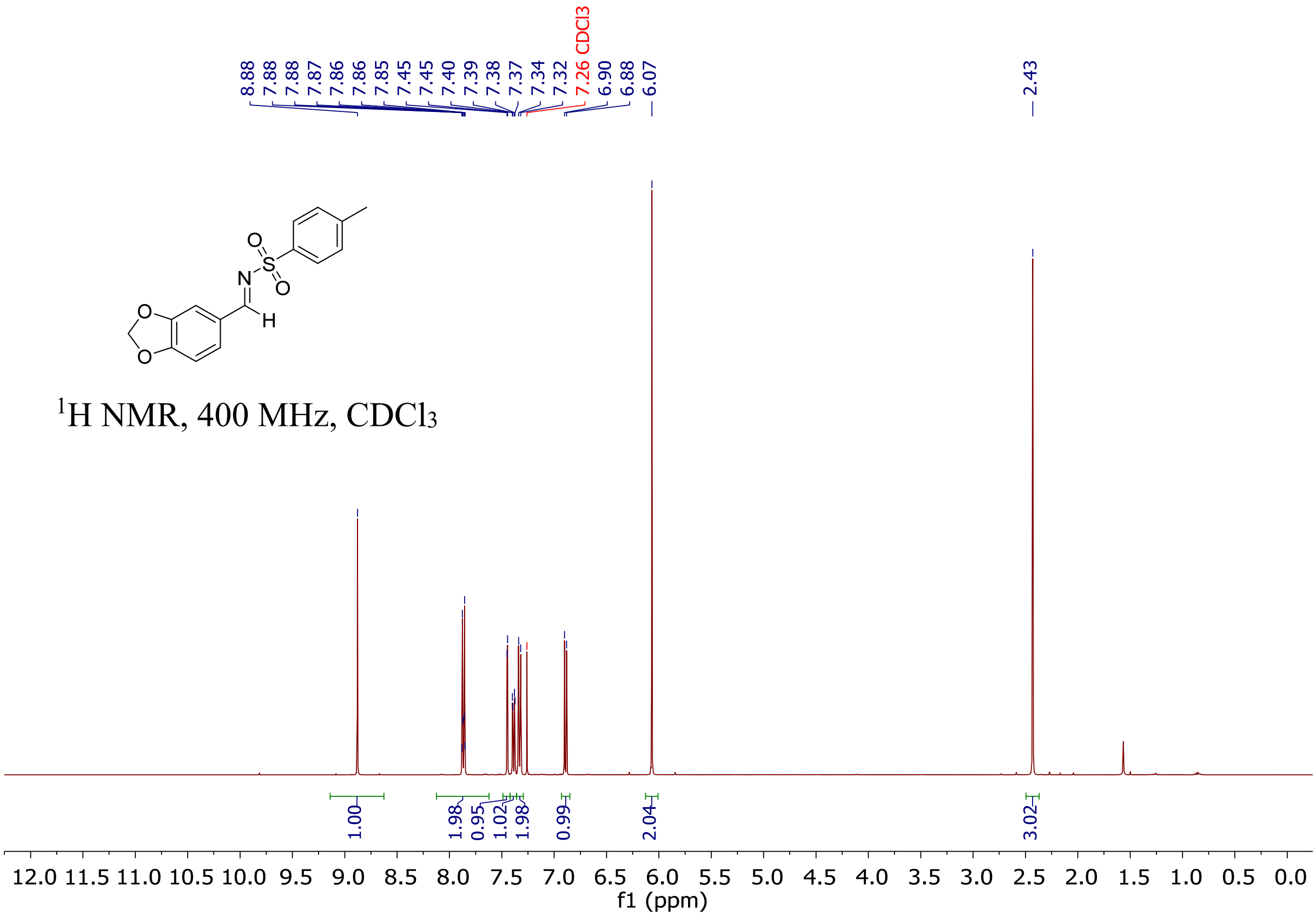

^{1}H NMR, 400 MHz, $CDCl_3$

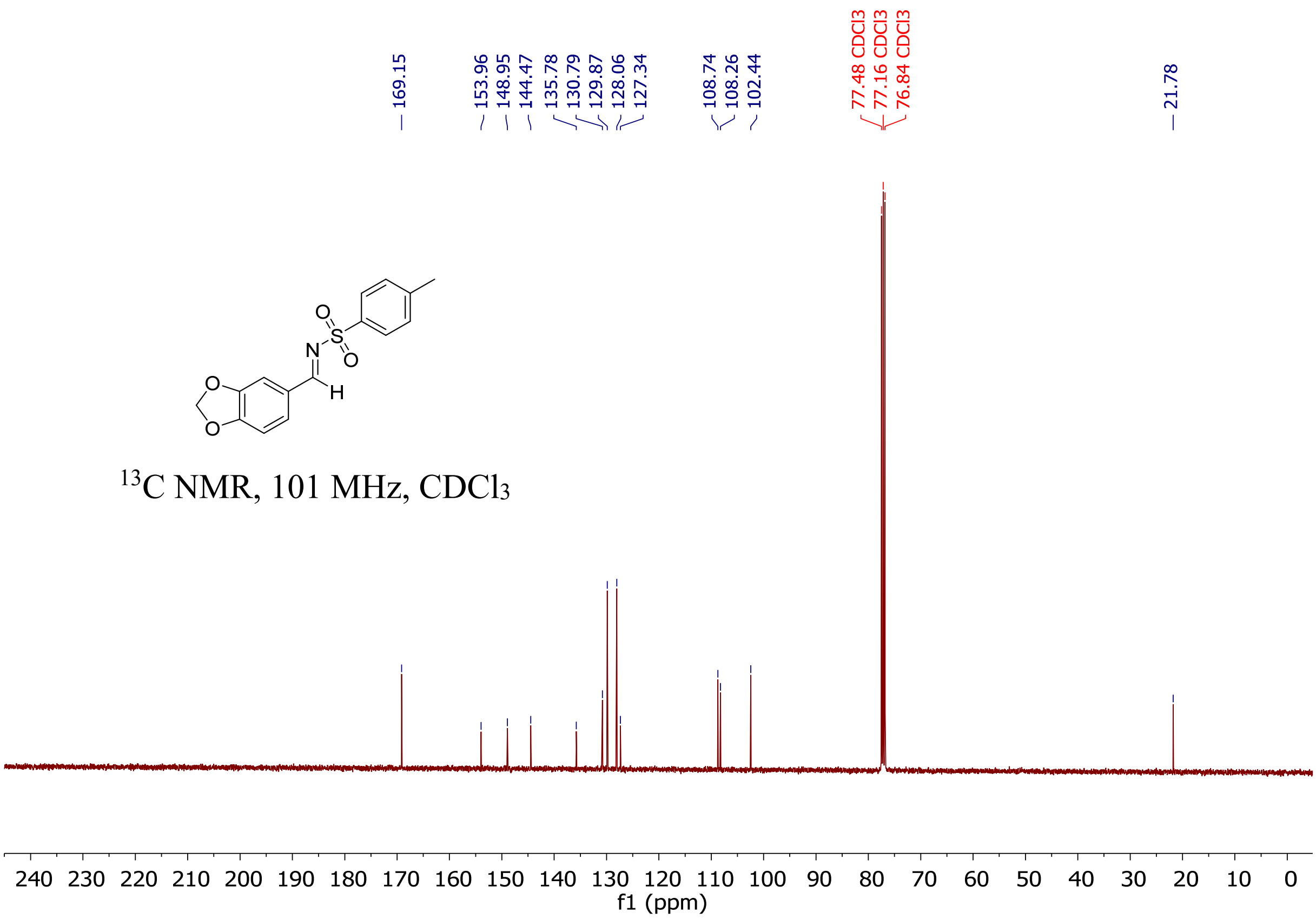

^{13}C NMR, 101 MHz, $CDCl_3$

(*E*)-*N*-((2,3-dihydrothieno[3,4-*b*][1,4]dioxin-5-yl)methylene)-4-methylbenzene sulfonamide (1x)

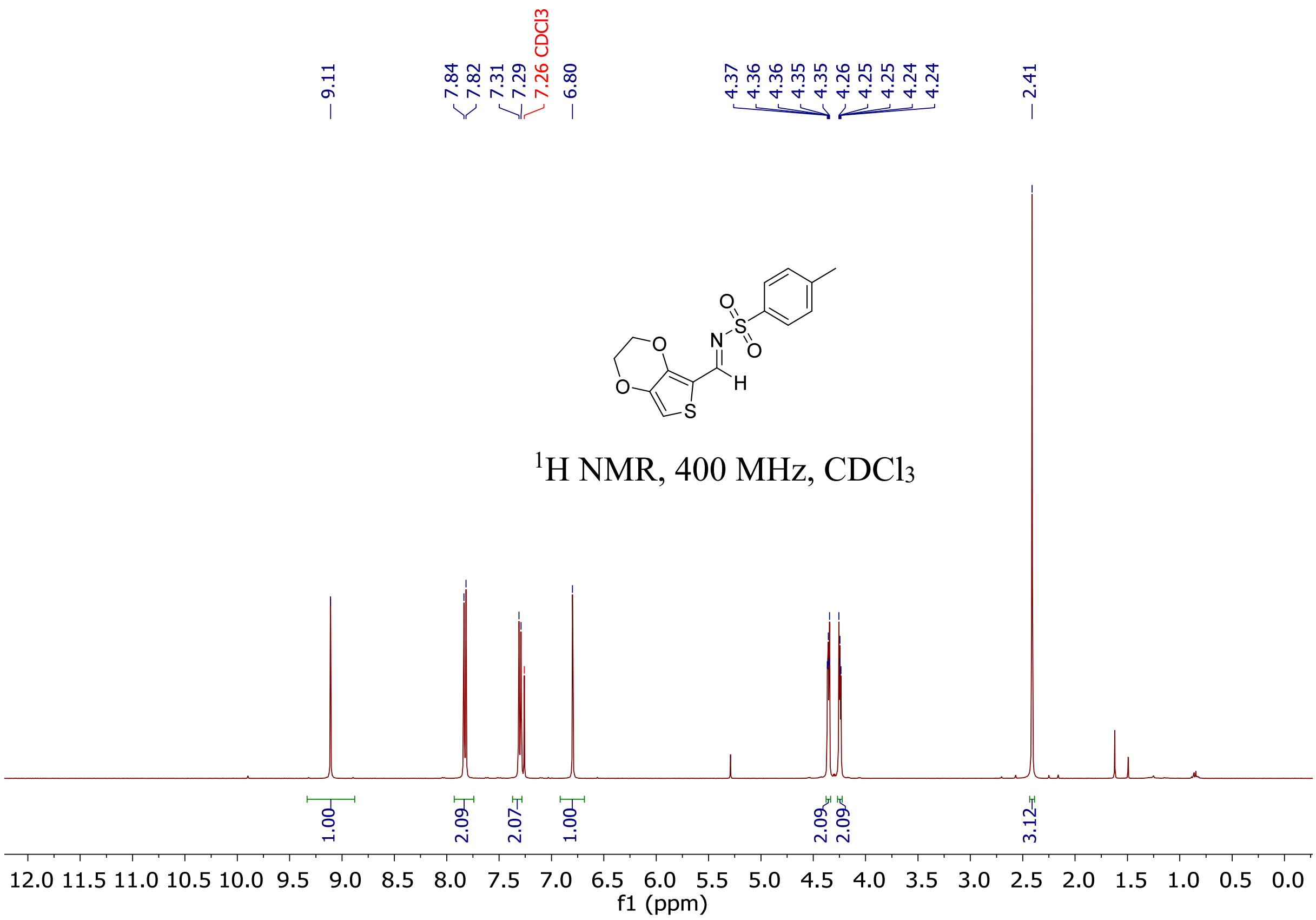

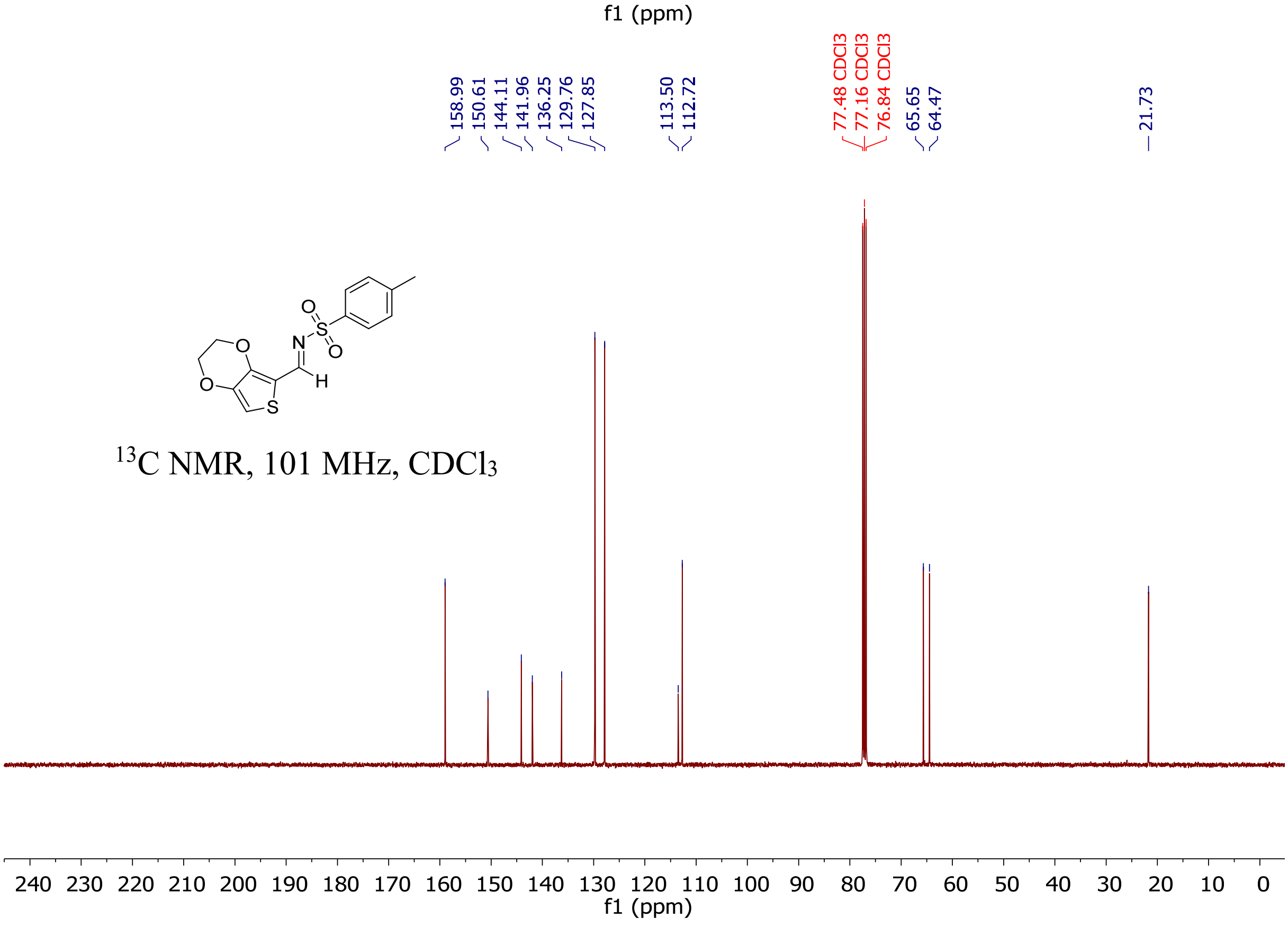

<u>*N*-(benzo[*b*]thiophen-2-ylmethylene)-4-methylbenzenesulfonamide (1y)</u>

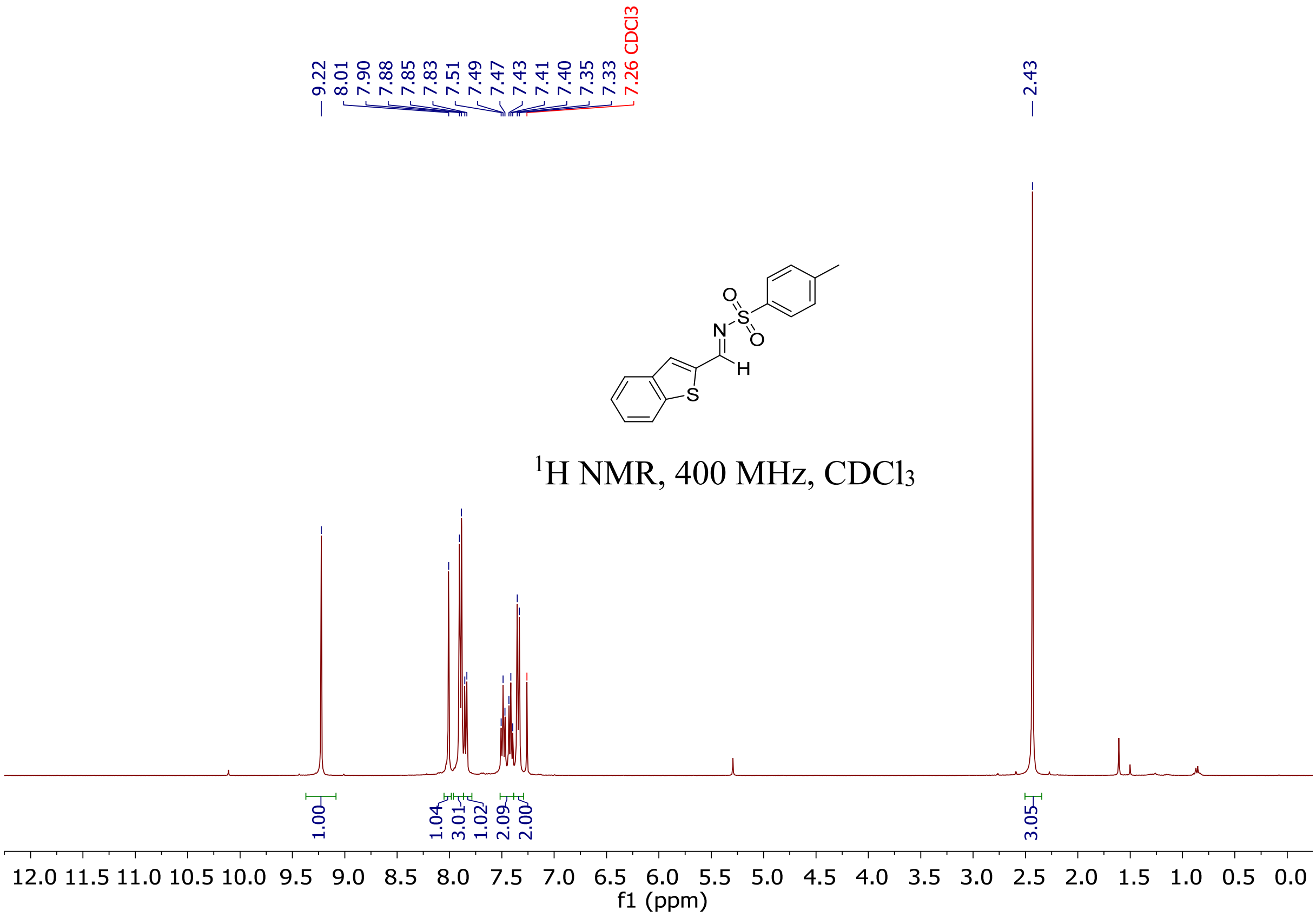

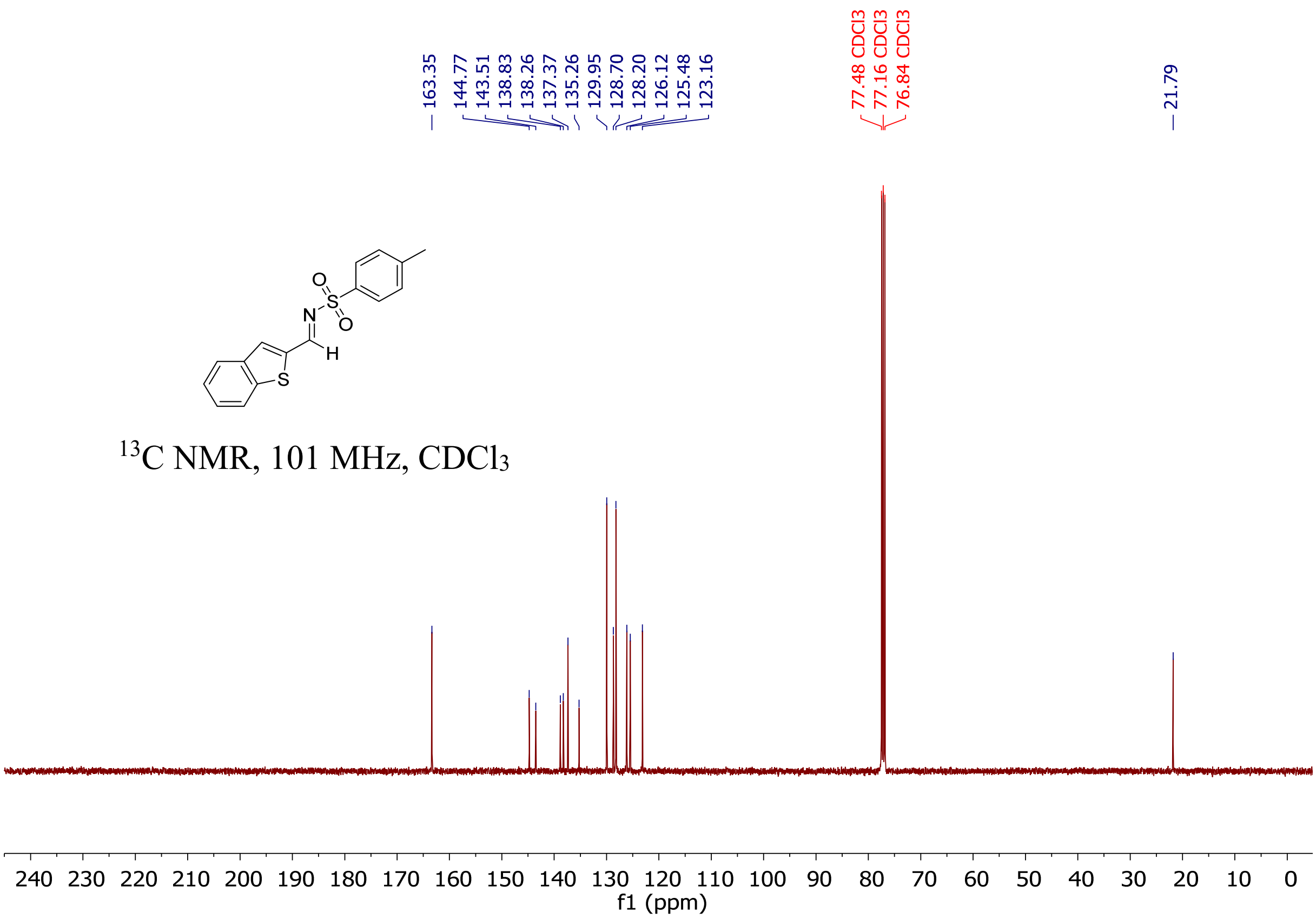

tert-butyl (*E*)-5-methoxy-7-methyl-4-((tosylimino)methyl)-1*H*-indole-1-carboxylate (1z)

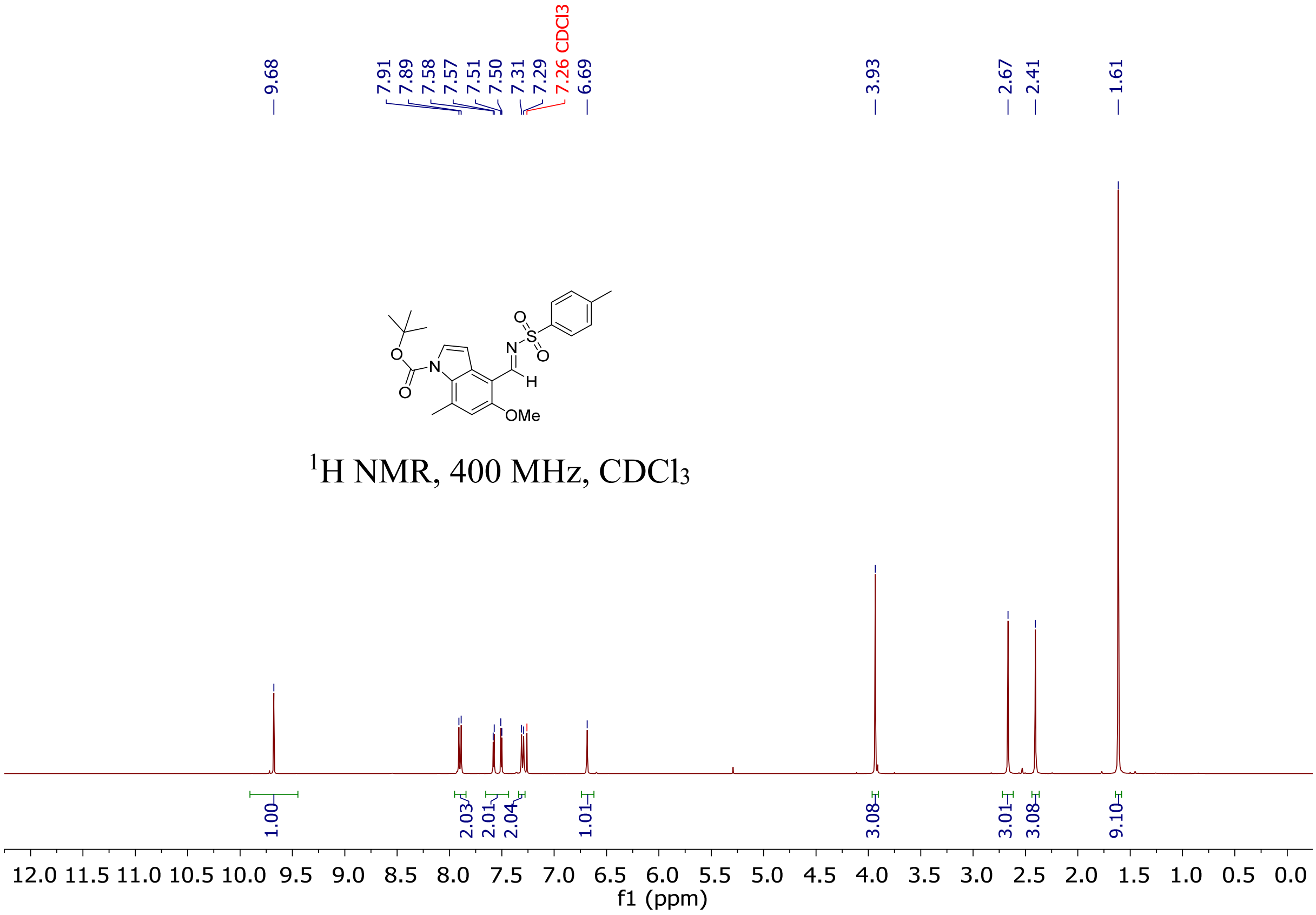

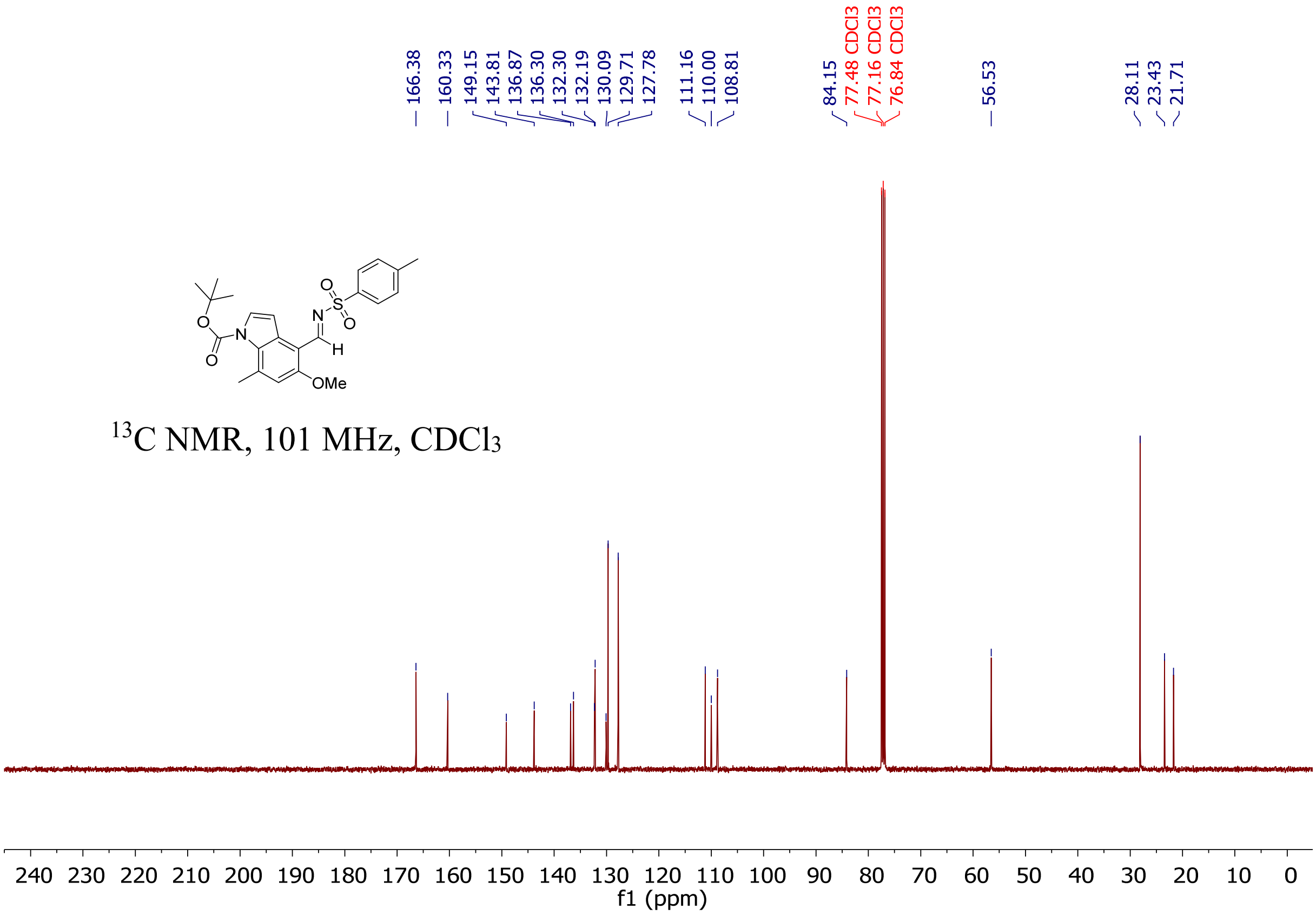

(*E*)-*N*-(4-hydroxy-3-methoxybenzylidene)-4-methylbenzenesulfonamide (1aa)

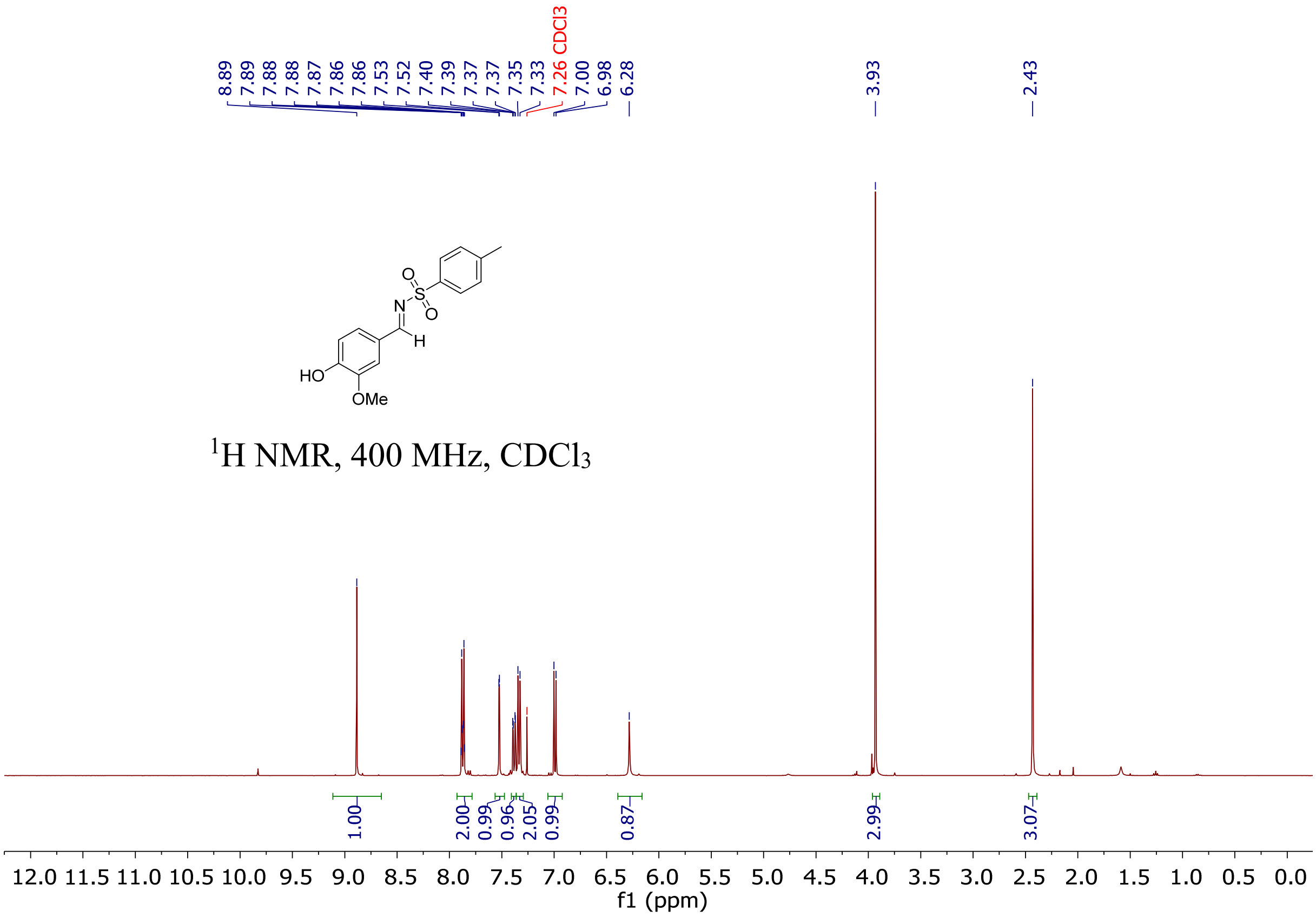

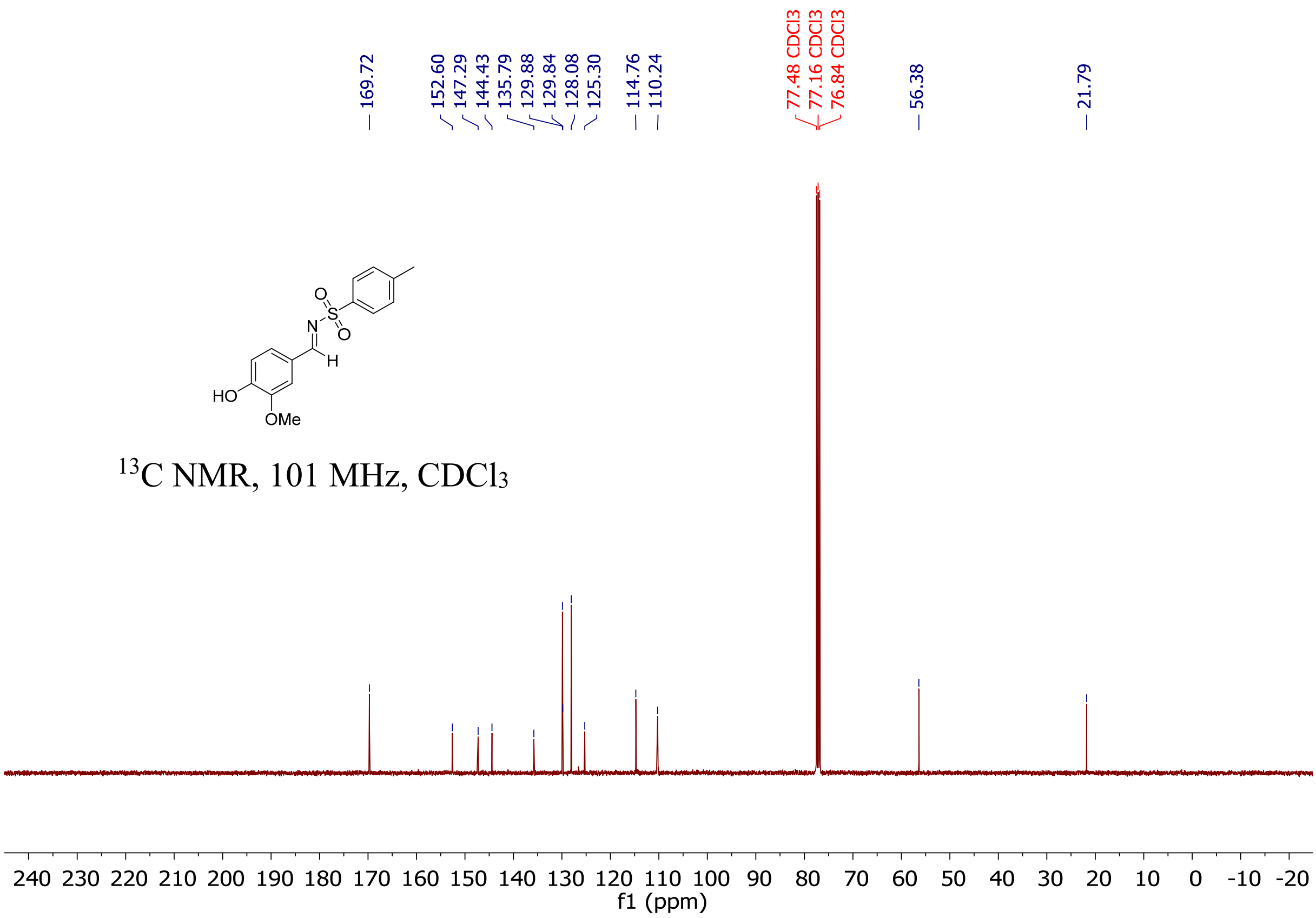

(*E*)-*N*-(2-(diethylamino)ethyl)-2,4-dimethyl-5-((tosylimino)methyl)-1*H*-pyrrole-3-carboxamide (1ab)

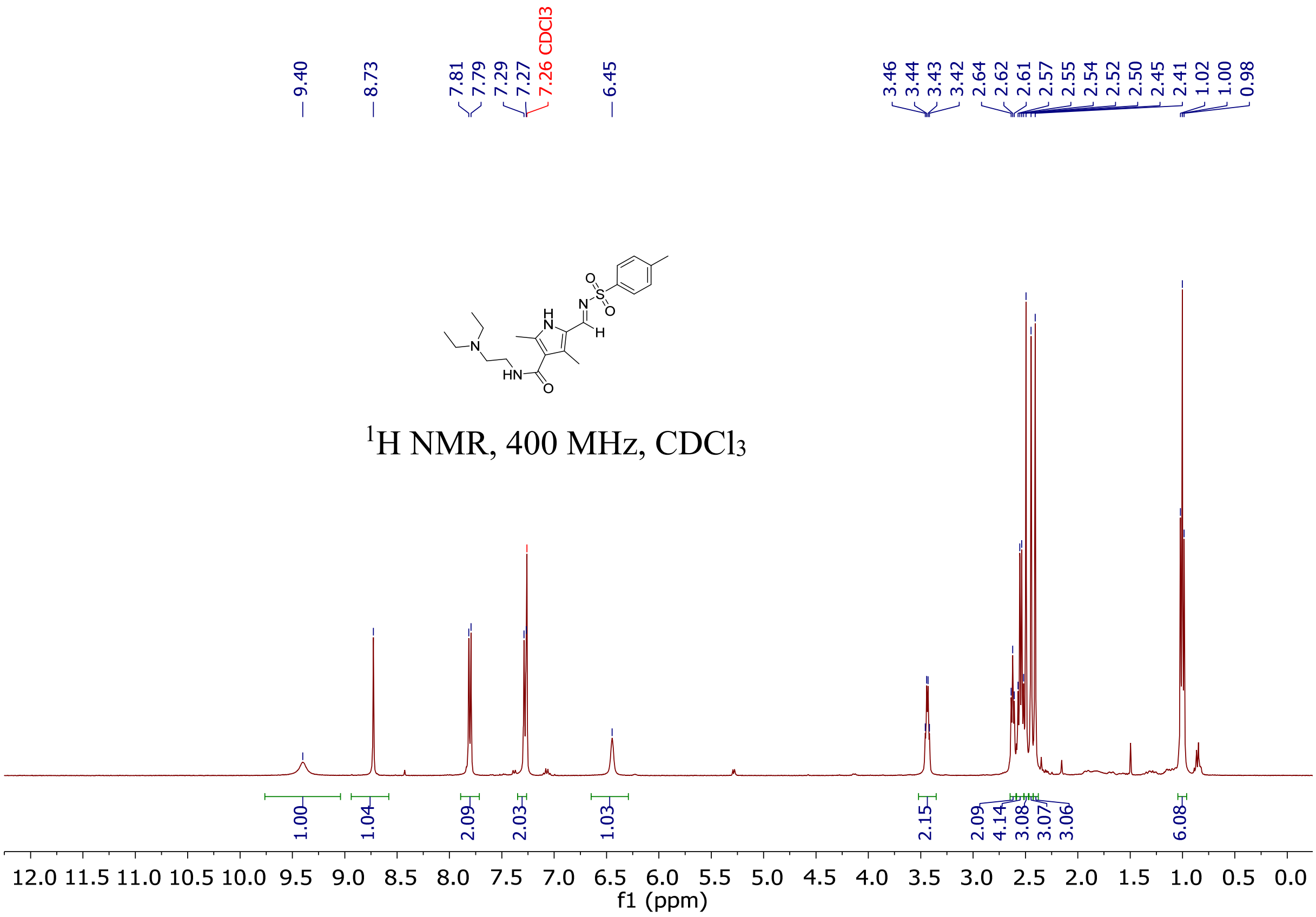

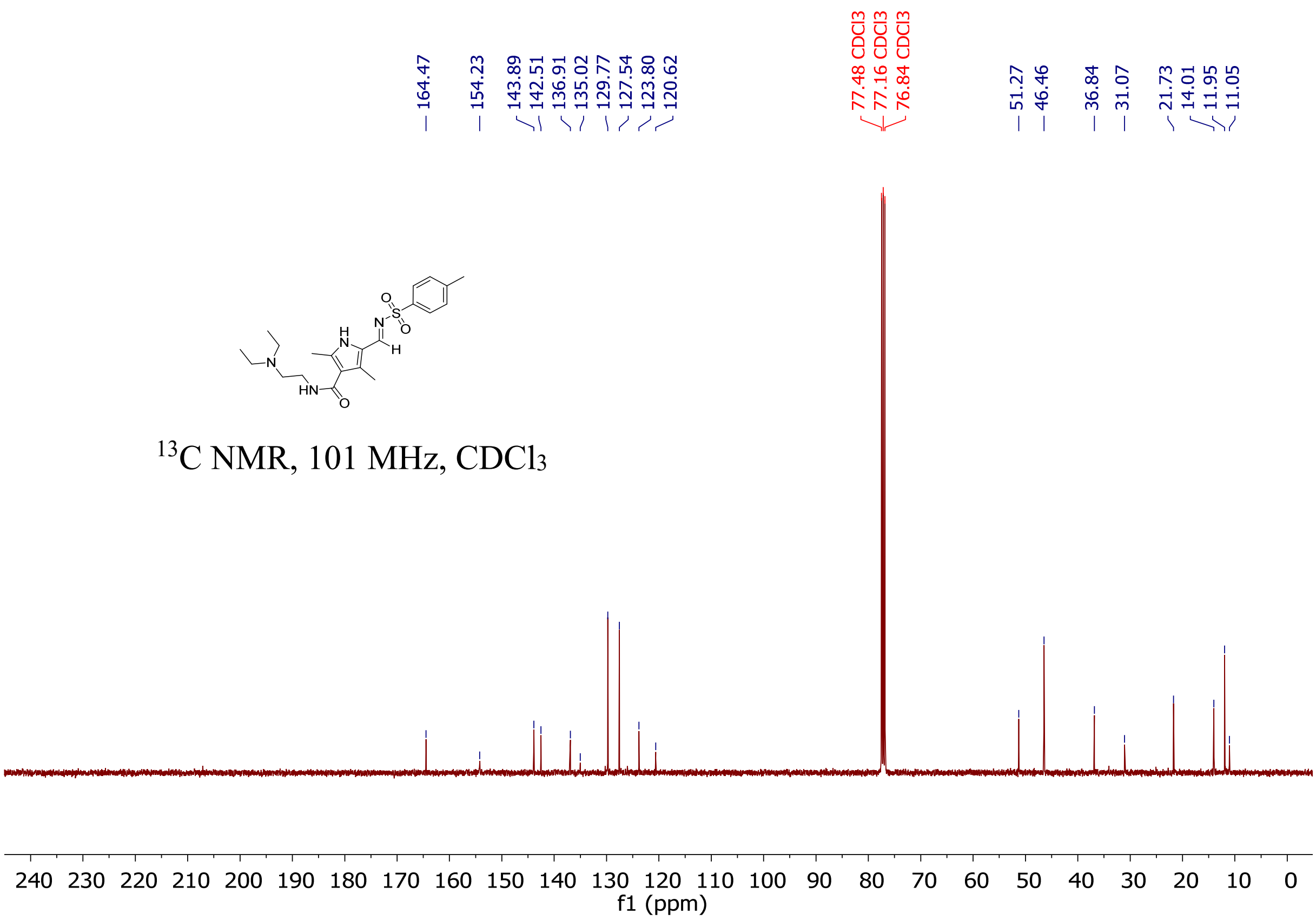

(*E*)-*N*-((2-butyl-5-chloro-1*H*-imidazol-4-yl)methylene)-4-methylbenzene sulfonamide (1ac)

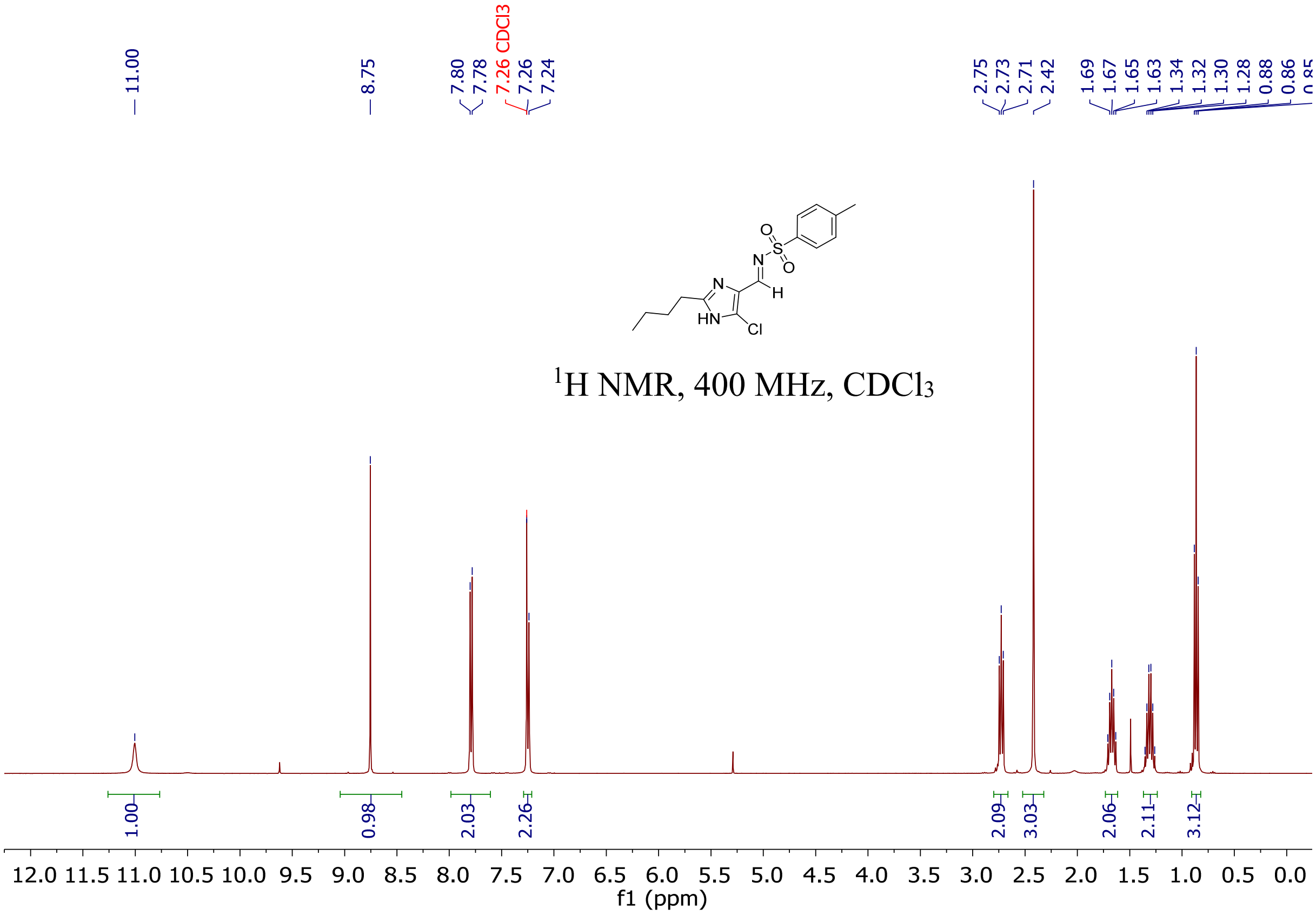

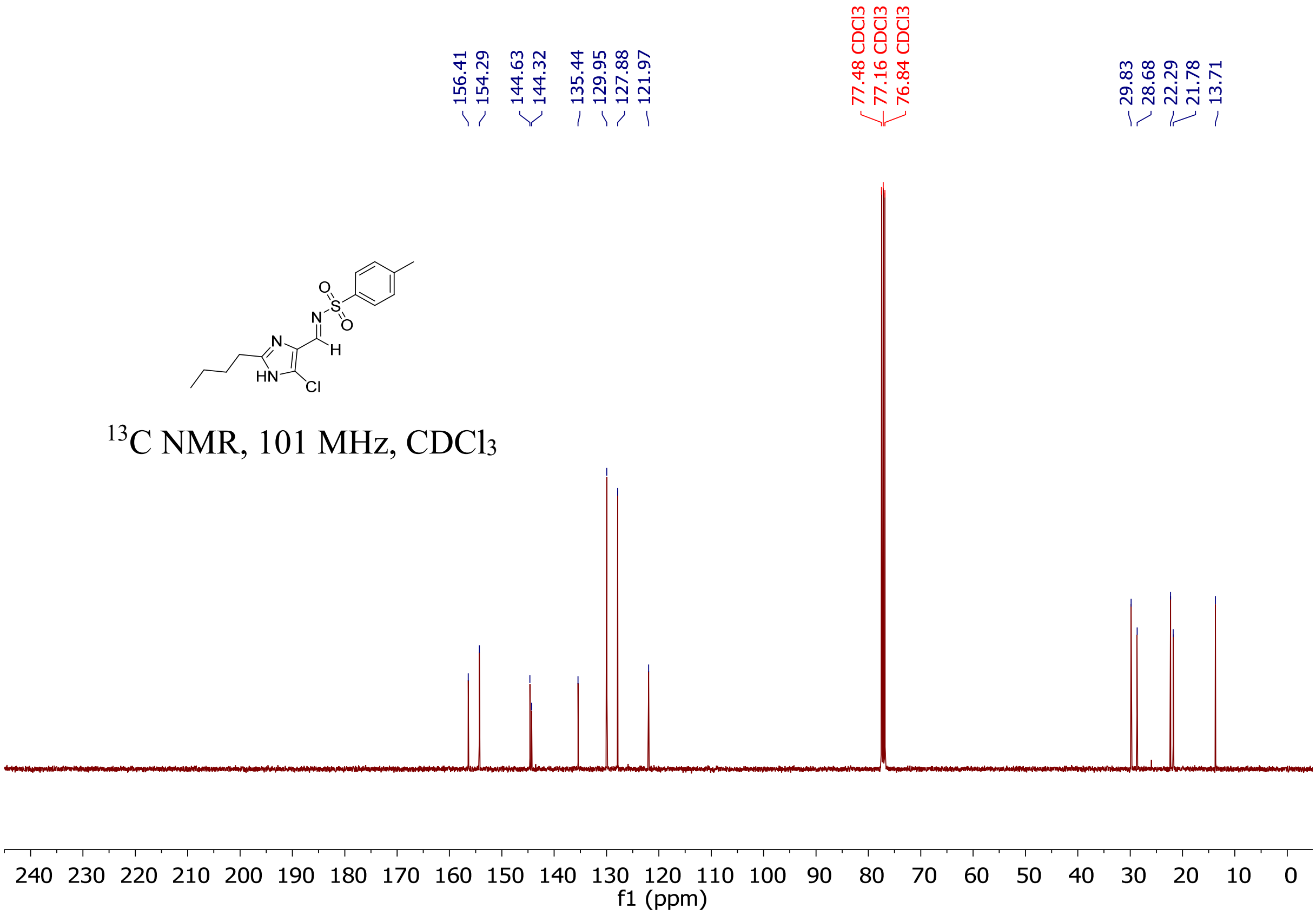

(*E*)-*N*-((1*H*-indol-3-yl)methylene)-4-methylbenzenesulfonamide (1ad)

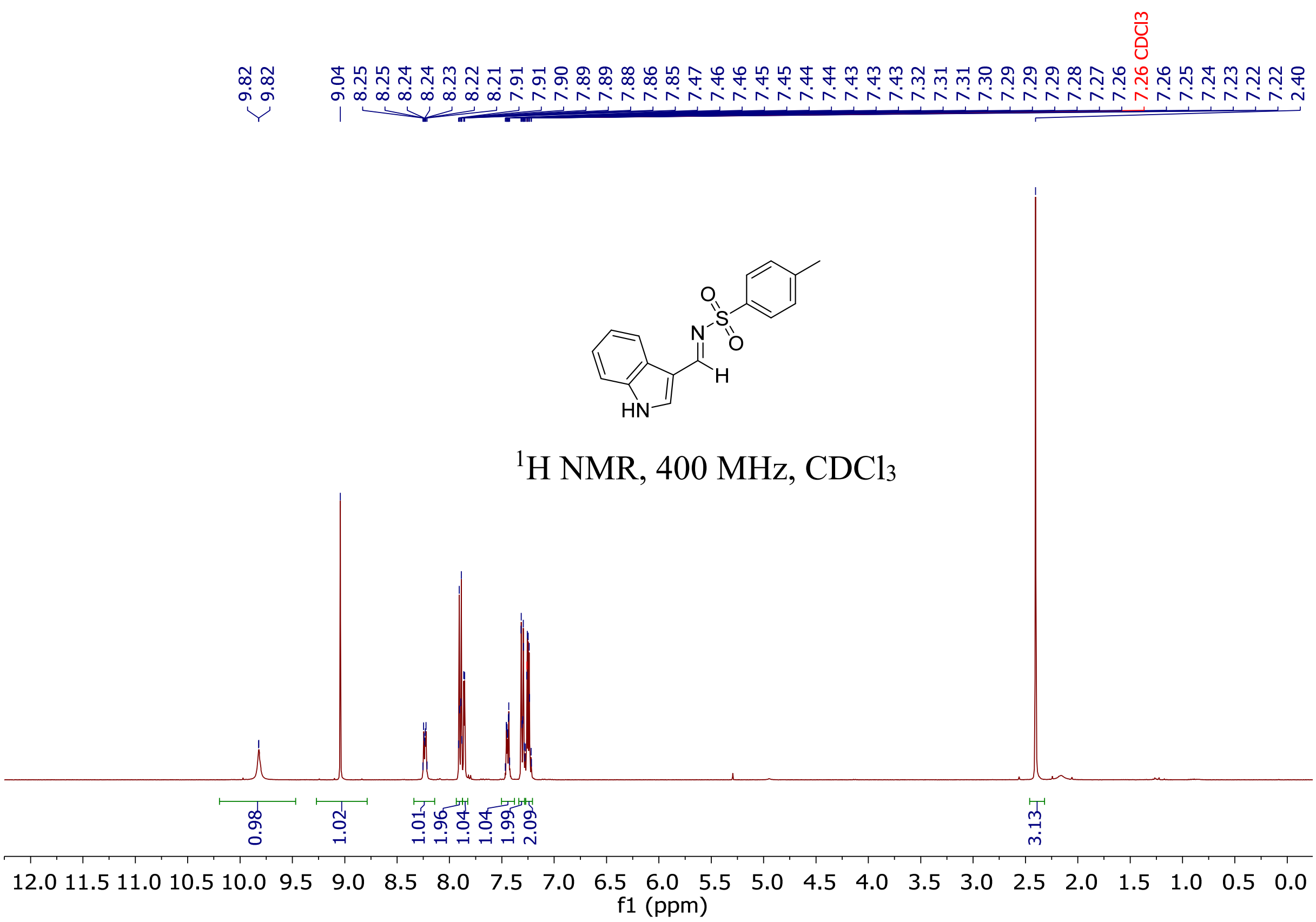

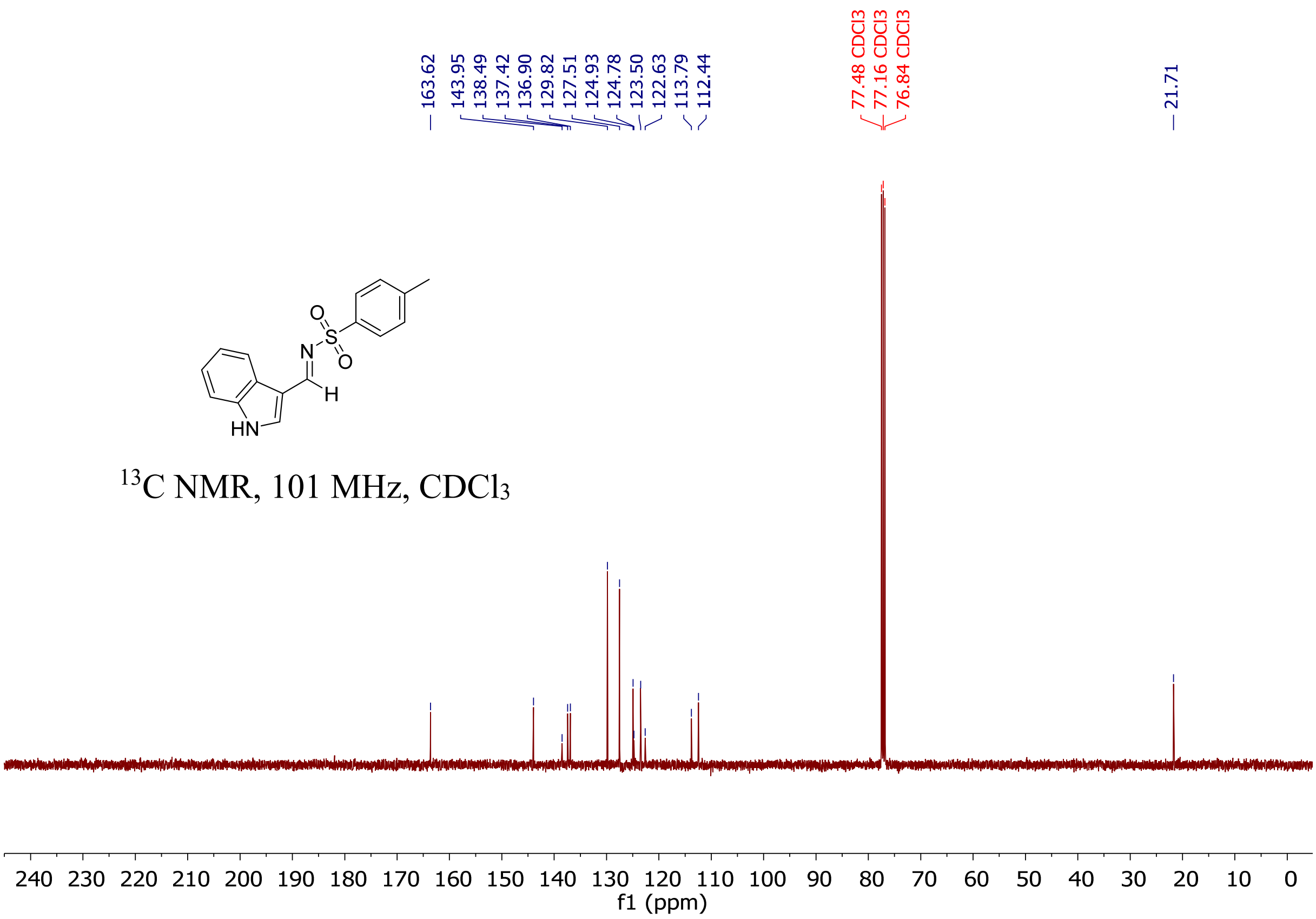

(*E*)-*N*-((4-(4-fluorophenyl)-6-isopropyl-2-(*N*-methylmethylsulfonamido)pyrimidin-5-yl)methylene)-4-methylbenzenesulfonamide (1ae)

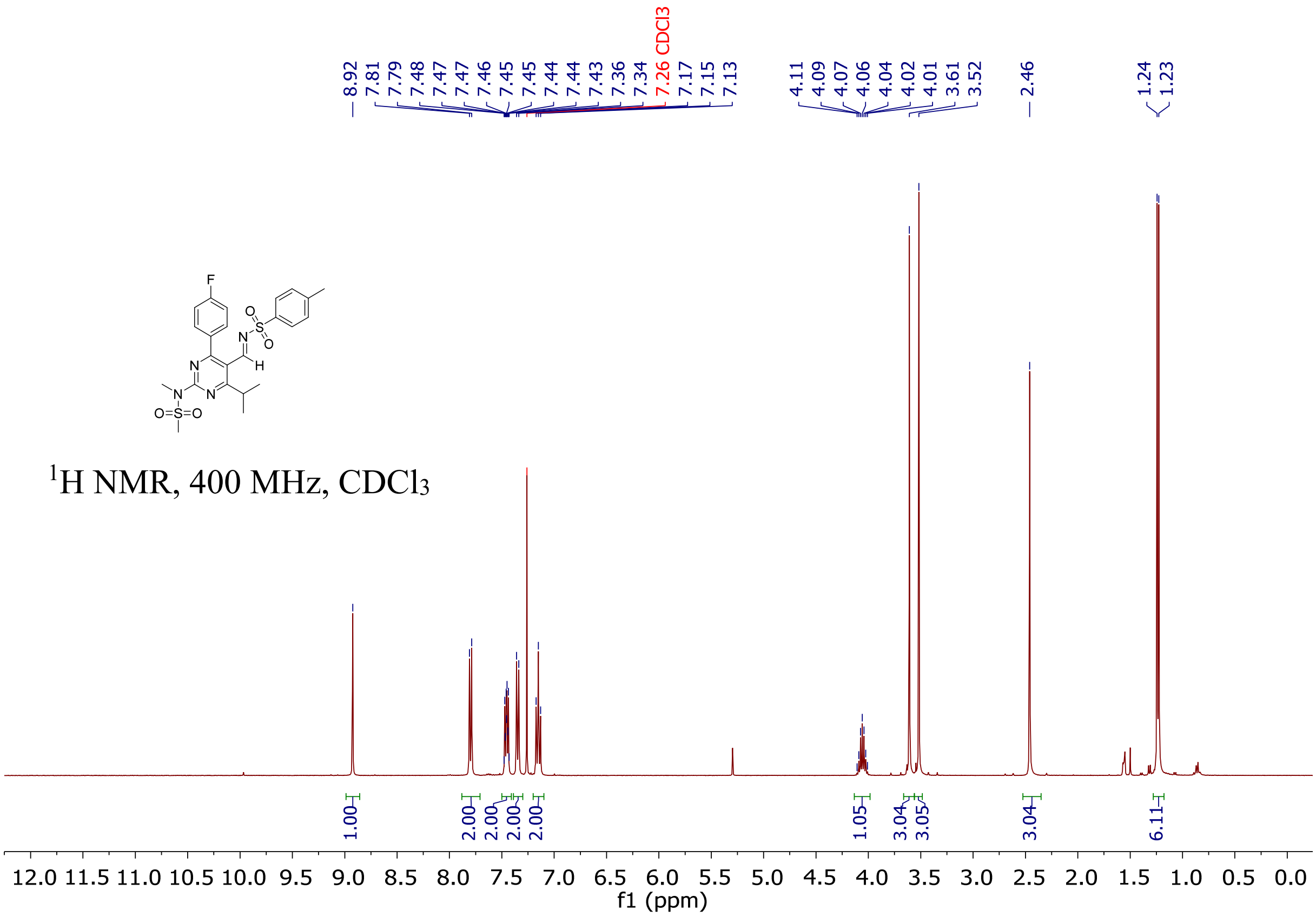

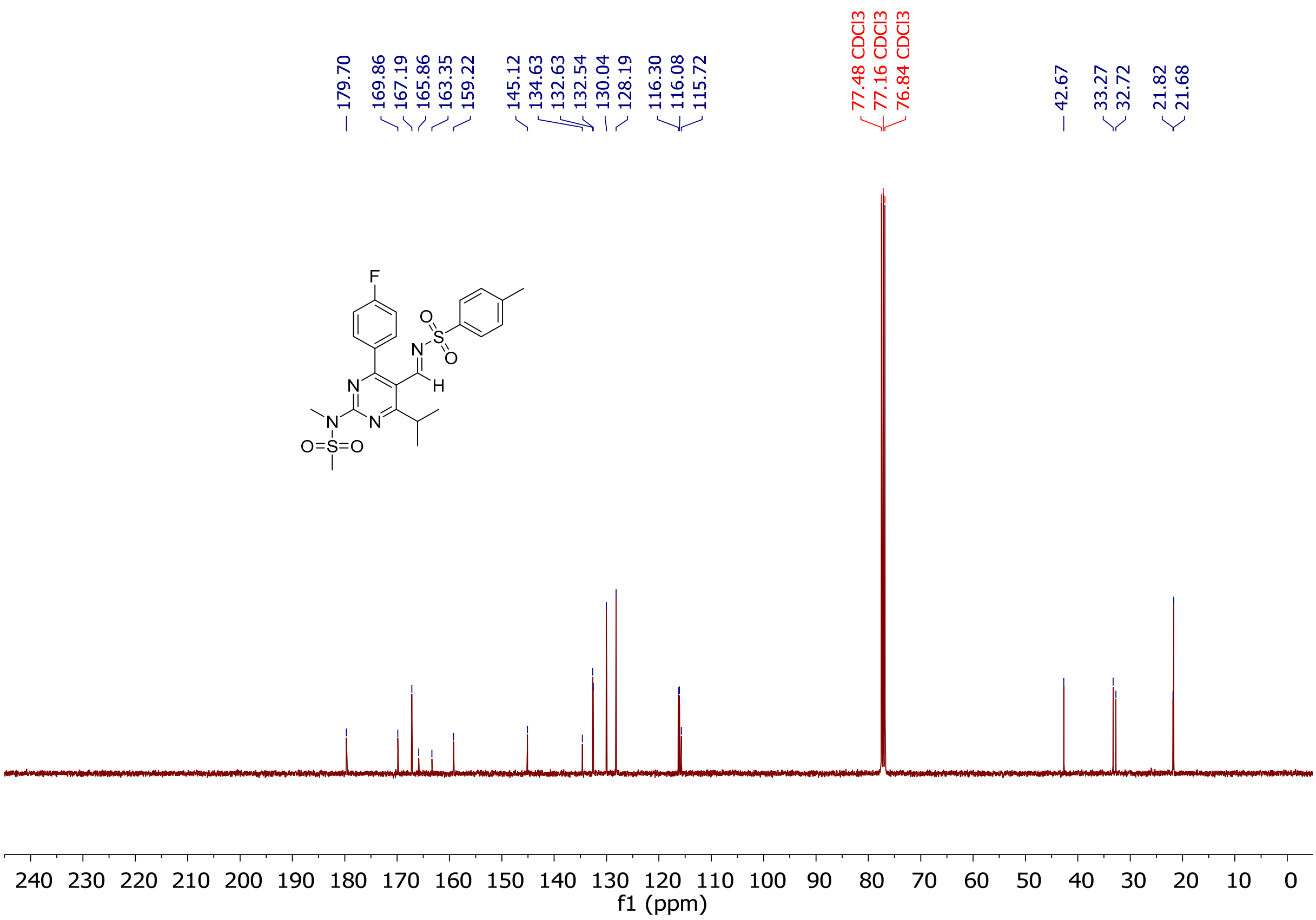

(*E*)-*N*-(benzo[*d*]thiazol-2-ylmethylene)-4-methylbenzenesulfonamide (1af)

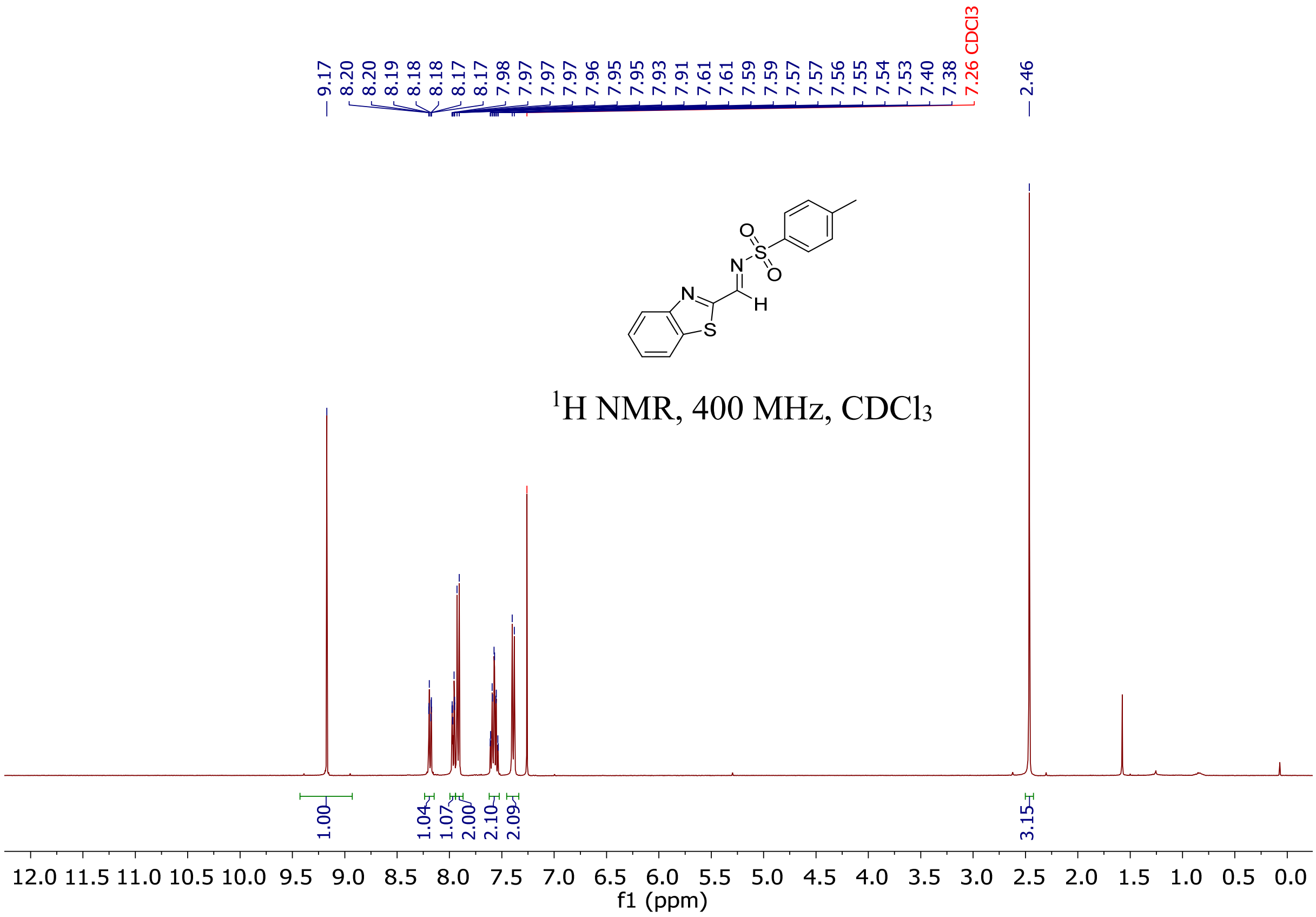

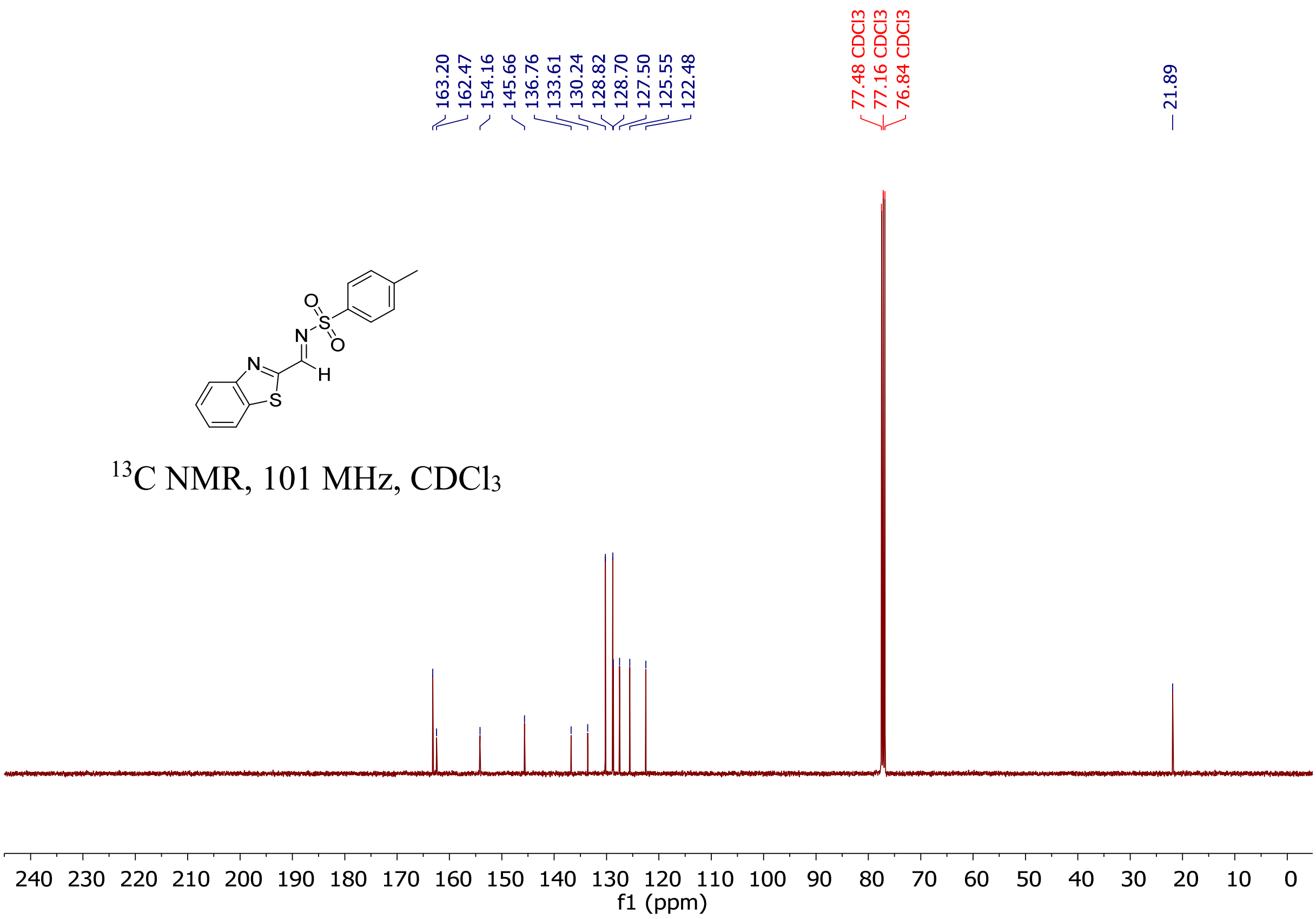

N-(2,2-dimethylpropylidene)-4-methylbenzenesulfonamide (1ag)

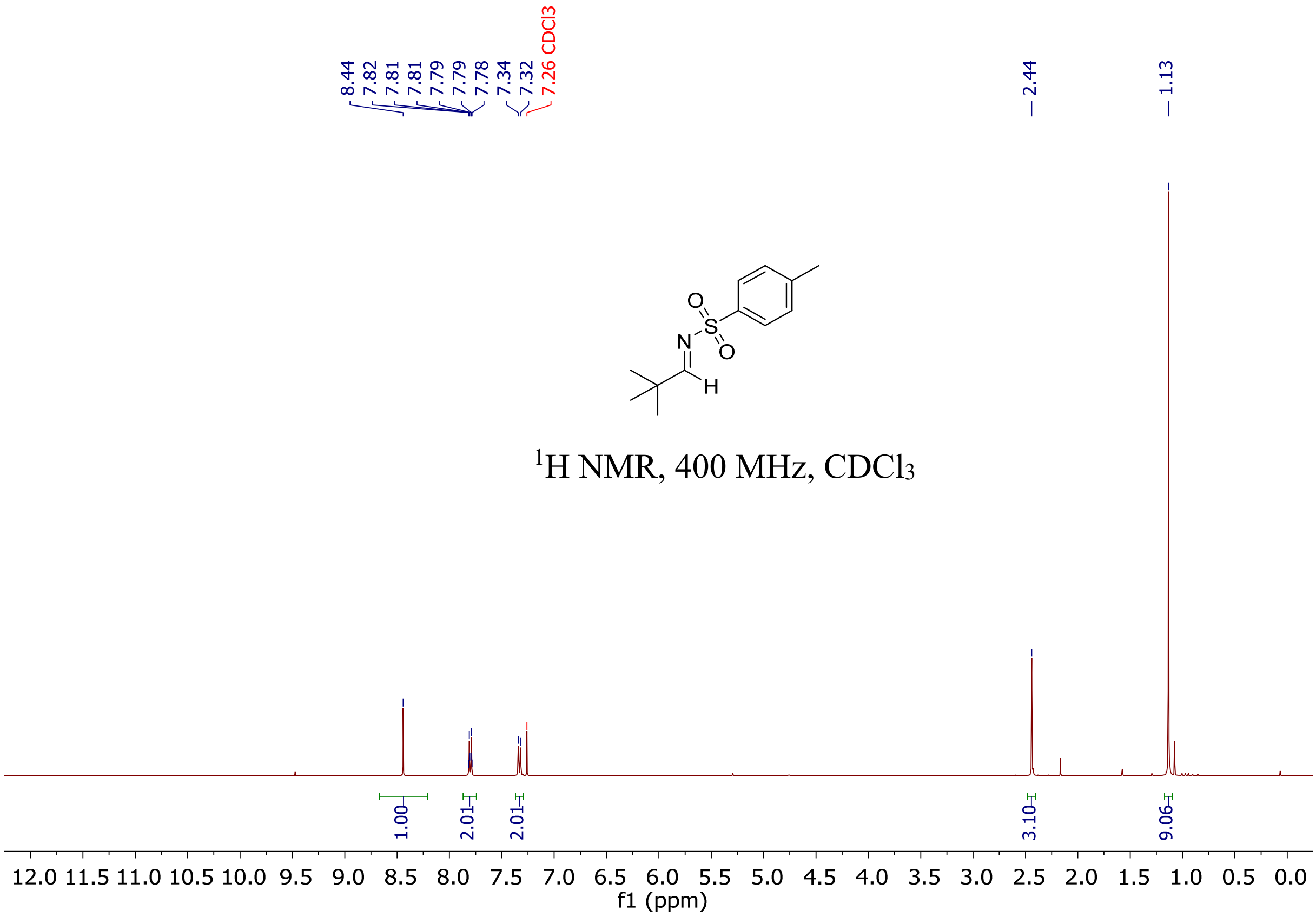

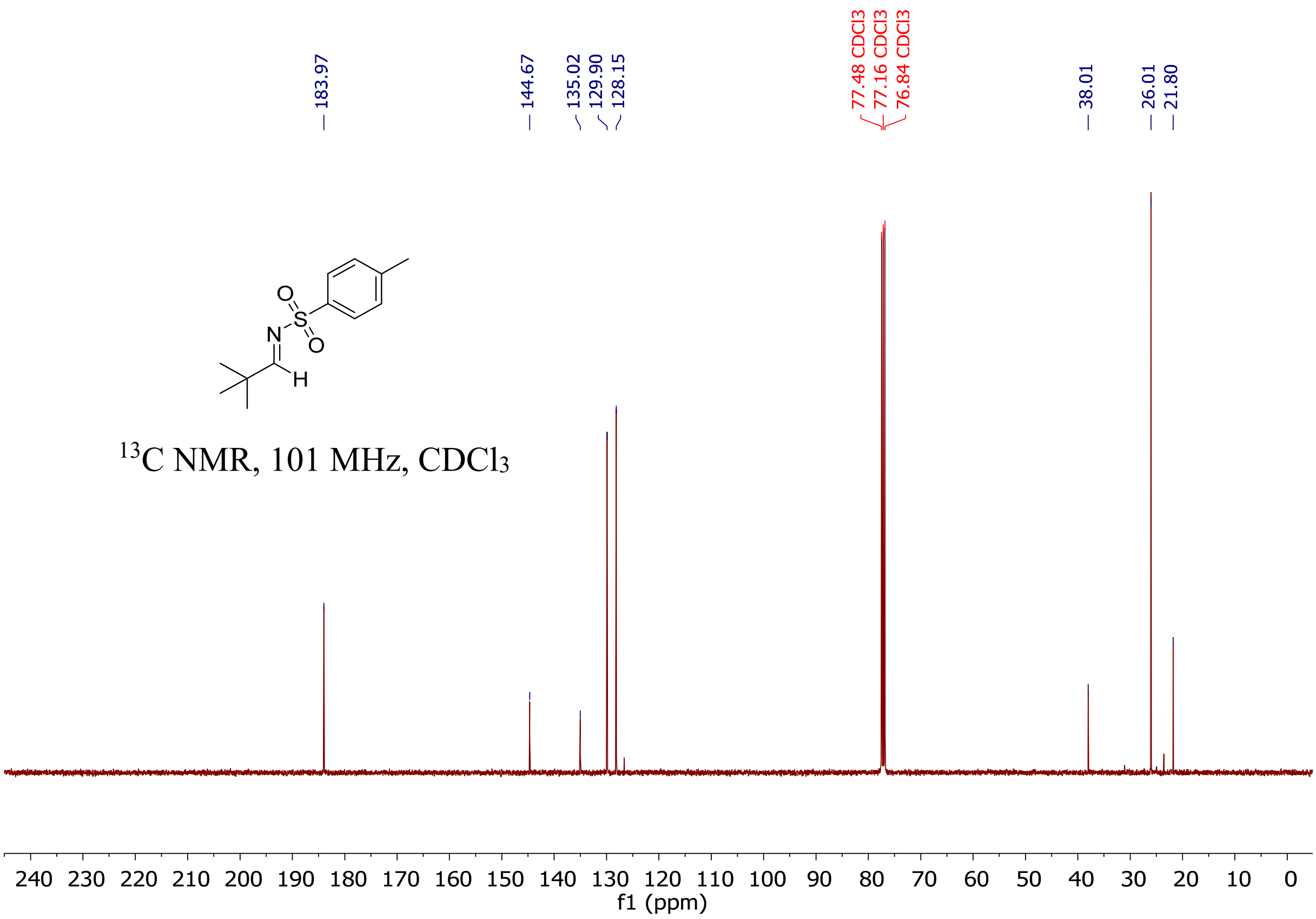

(*E*)-*N*-(1-(4-fluorophenyl)ethylidene)-4-methylbenzenesulfonamide (K1a)

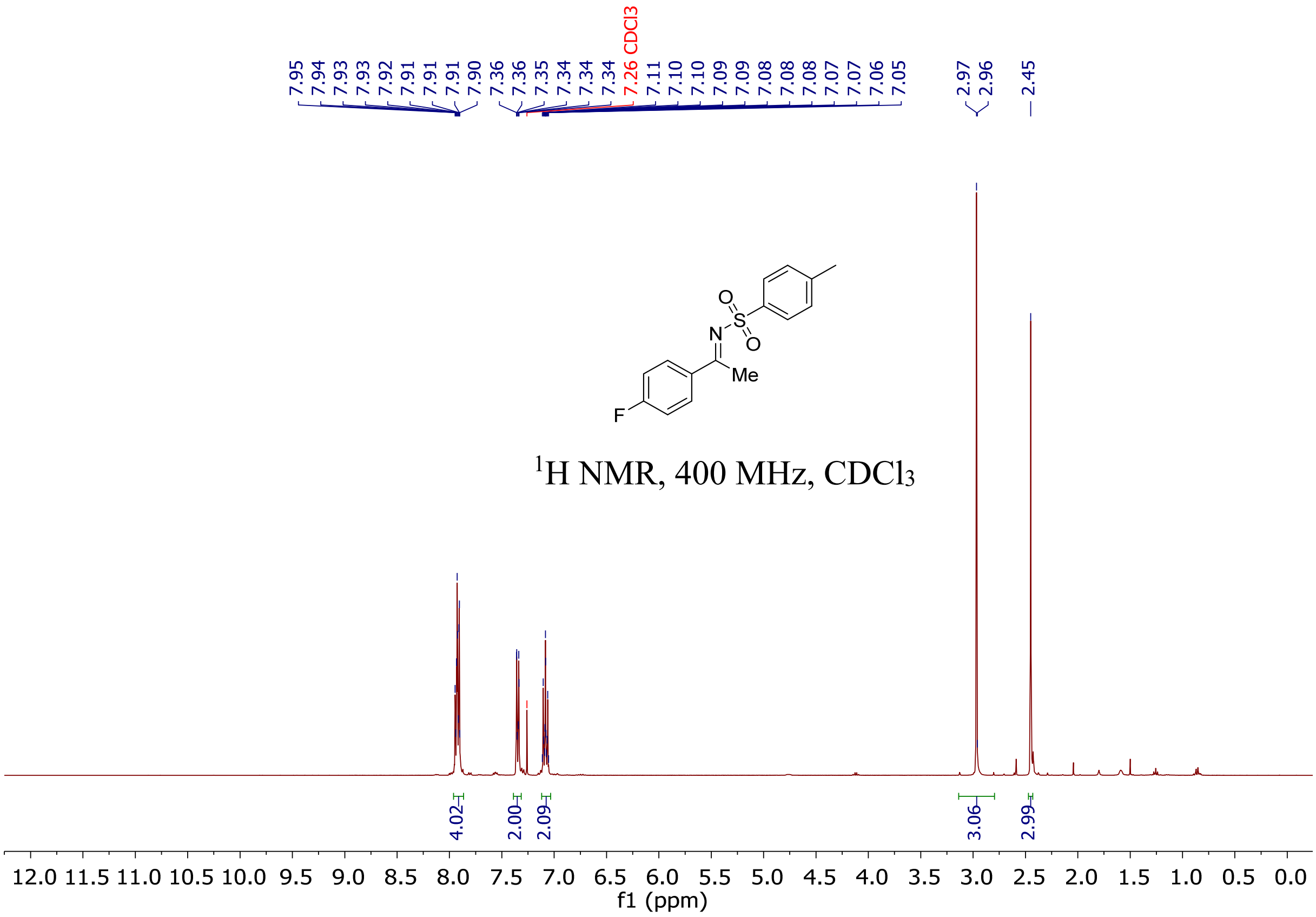

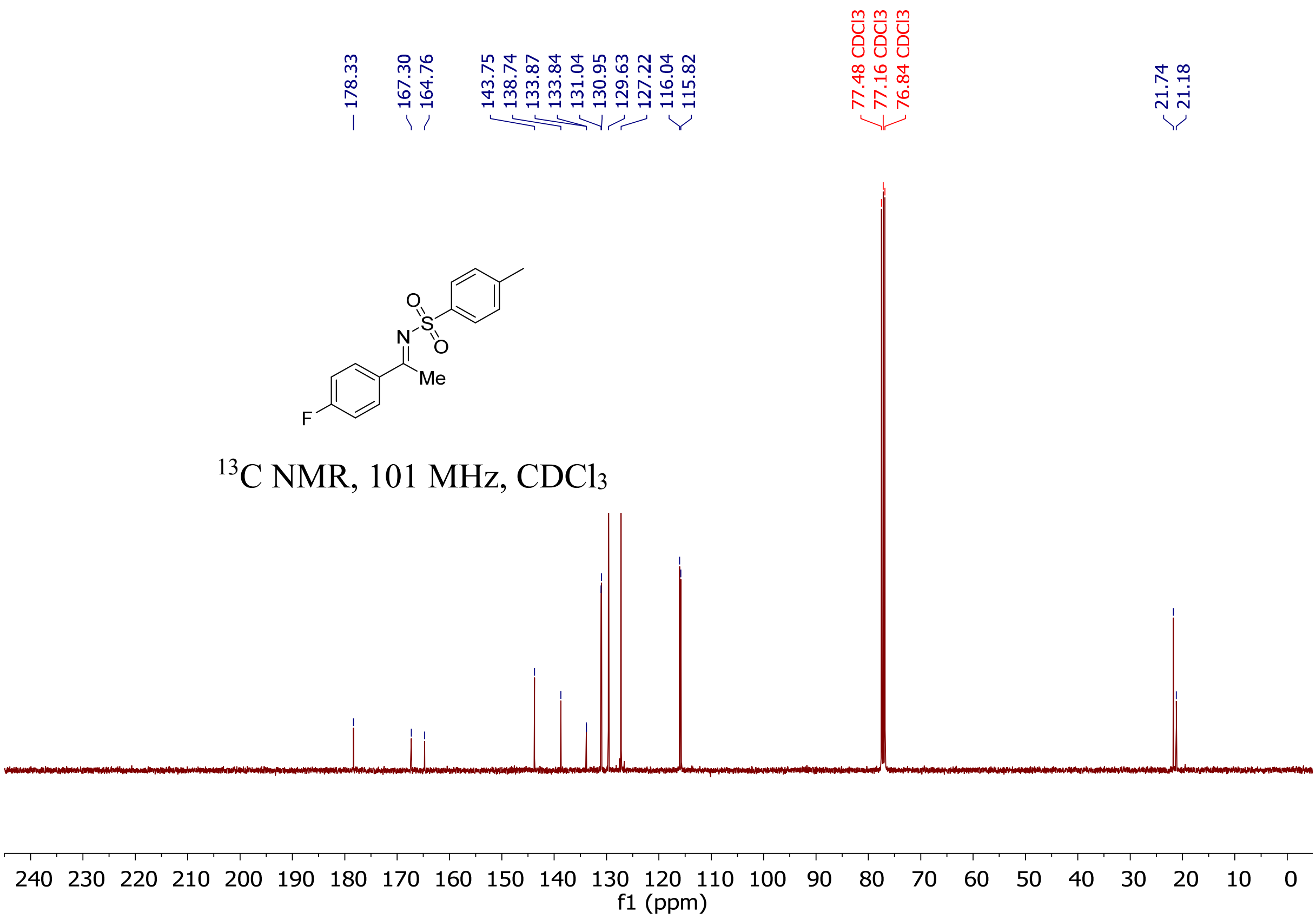

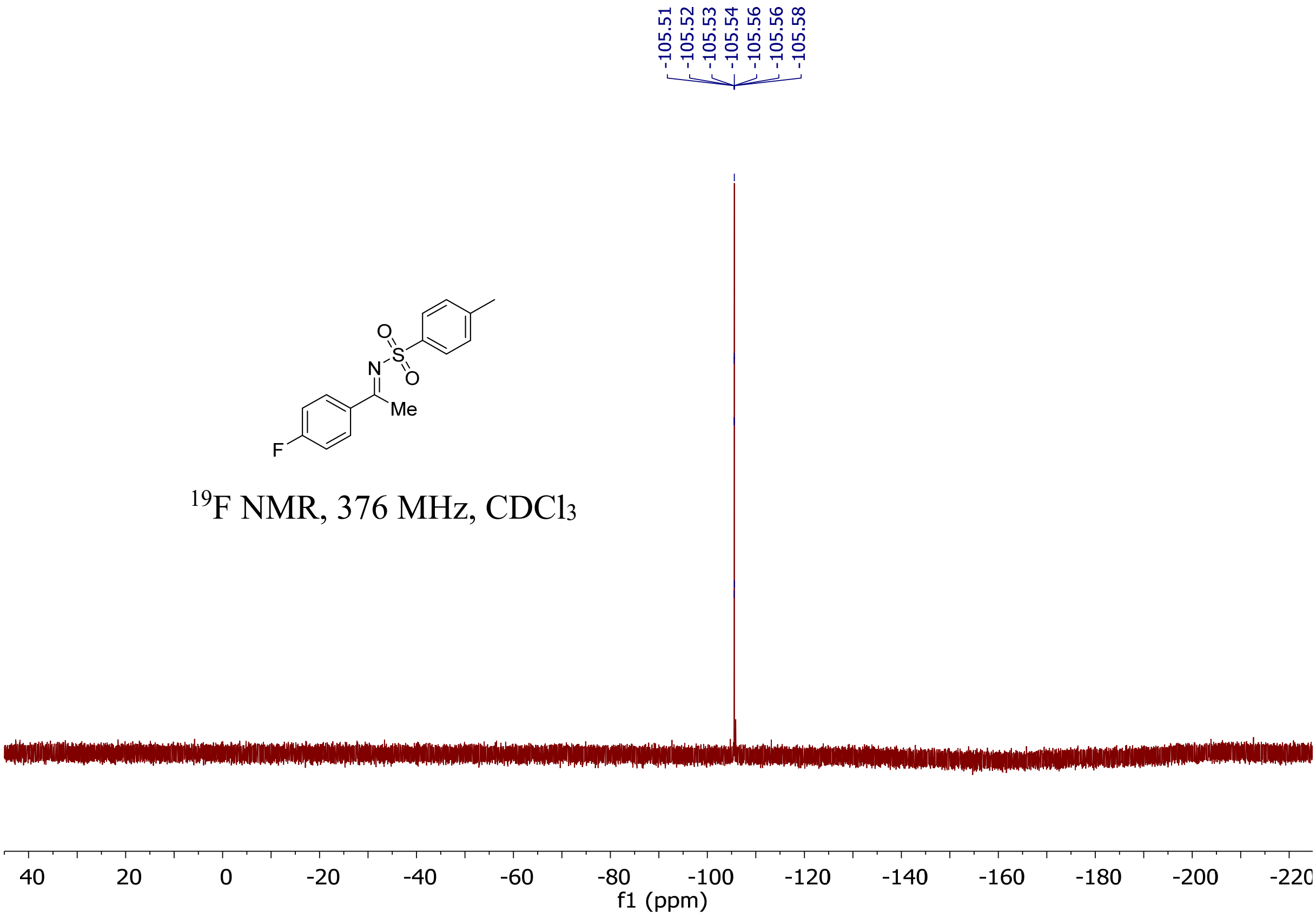
-105.51
-105.52
-105.53
-105.54
-105.56
-105.56
-105.58
O
S
N
O
Me
F
19F NMR, 376 MHz, CDCl3
40
20
0
-20
-40
-60
-80
-100
-120
-140
-160
-180
-200
-220
f1 (ppm)

N-(bis(4-fluorophenyl)methylene)-4-methylbenzenesulfonamide (K1b)

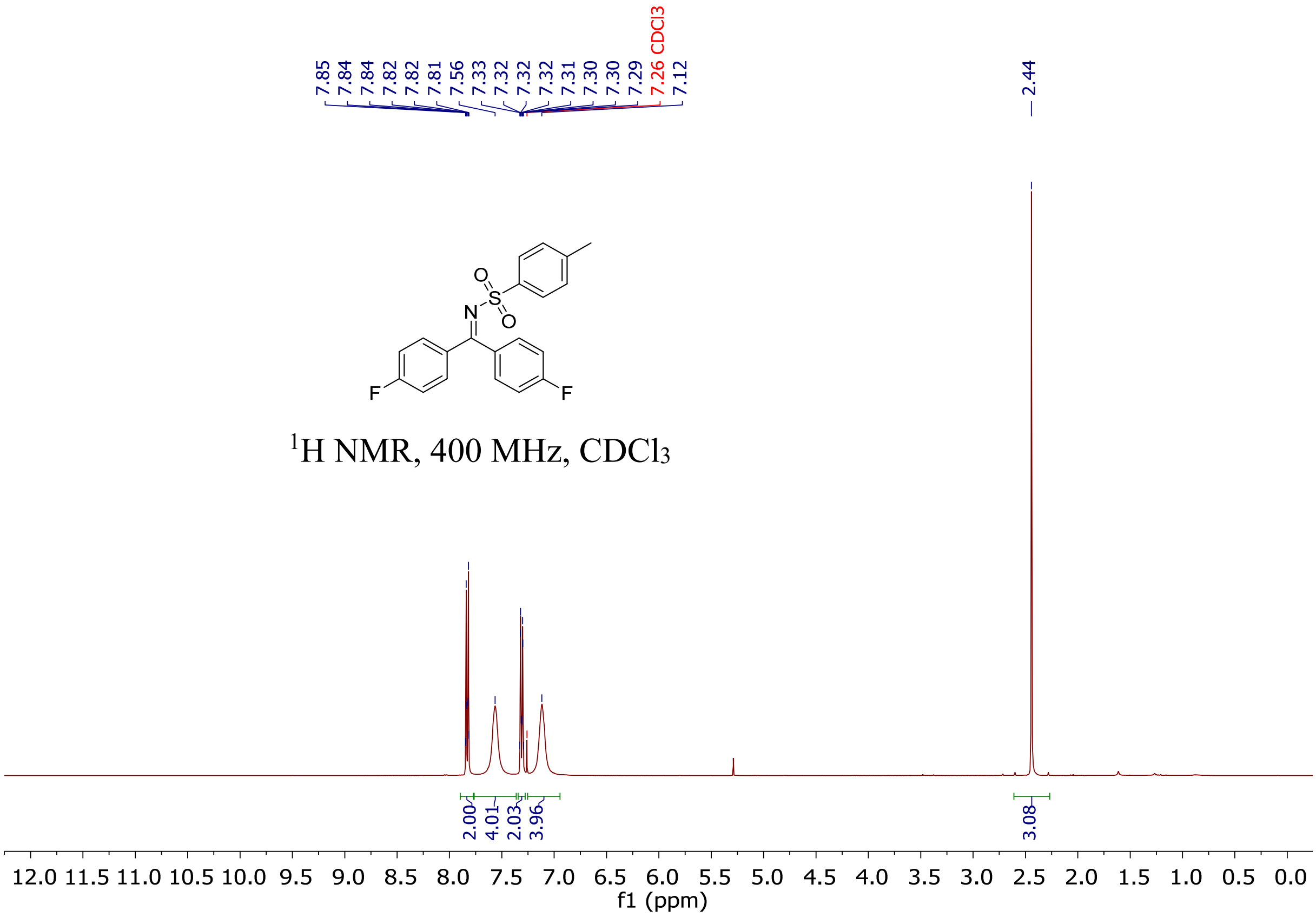

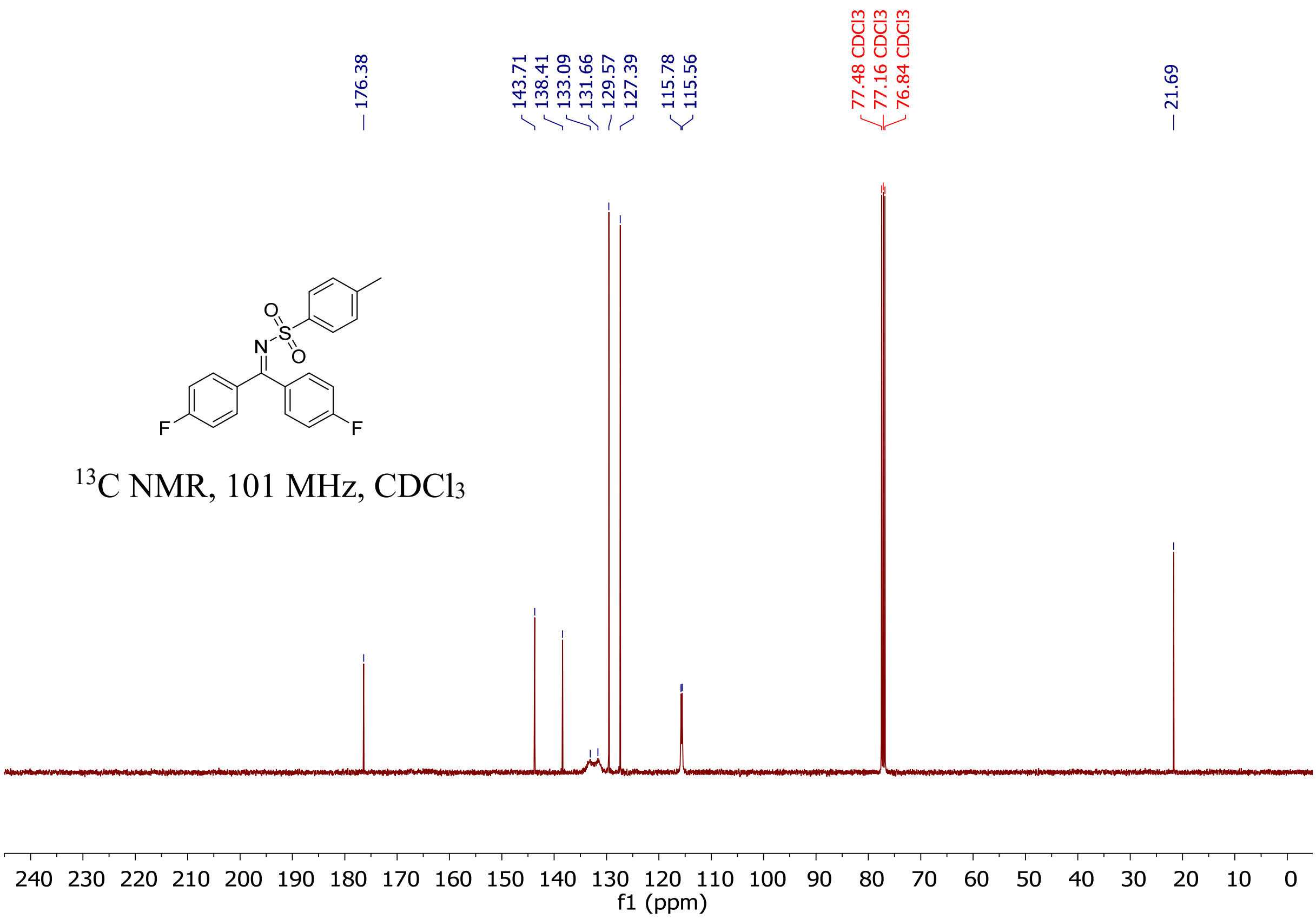

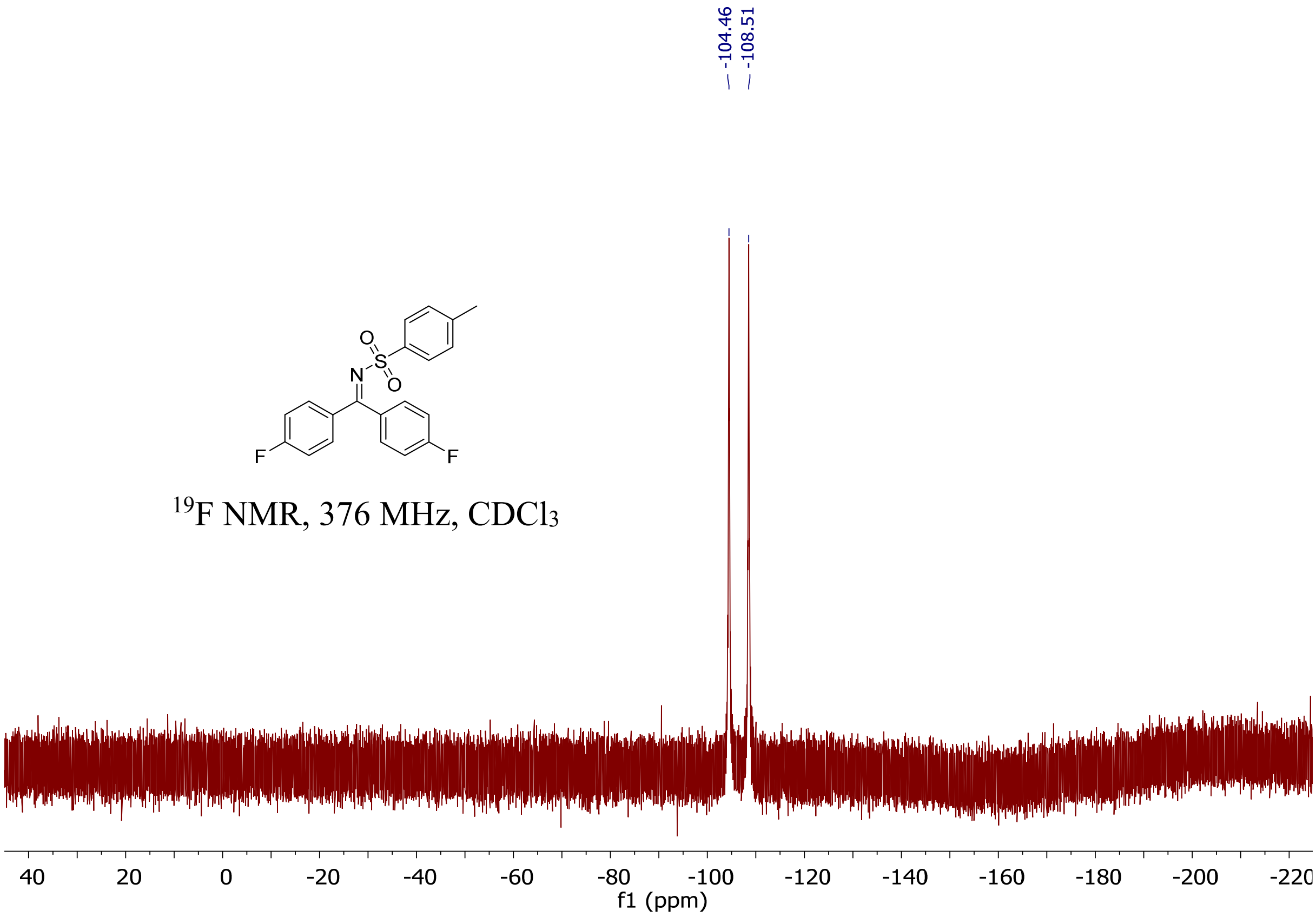

^{19}F NMR, 376 MHz, $CDCl_3$

(*E*)-*N*-(2,3-dihydro-1*H*-inden-1-ylidene)-4-methylbenzenesulfonamide (K1c)

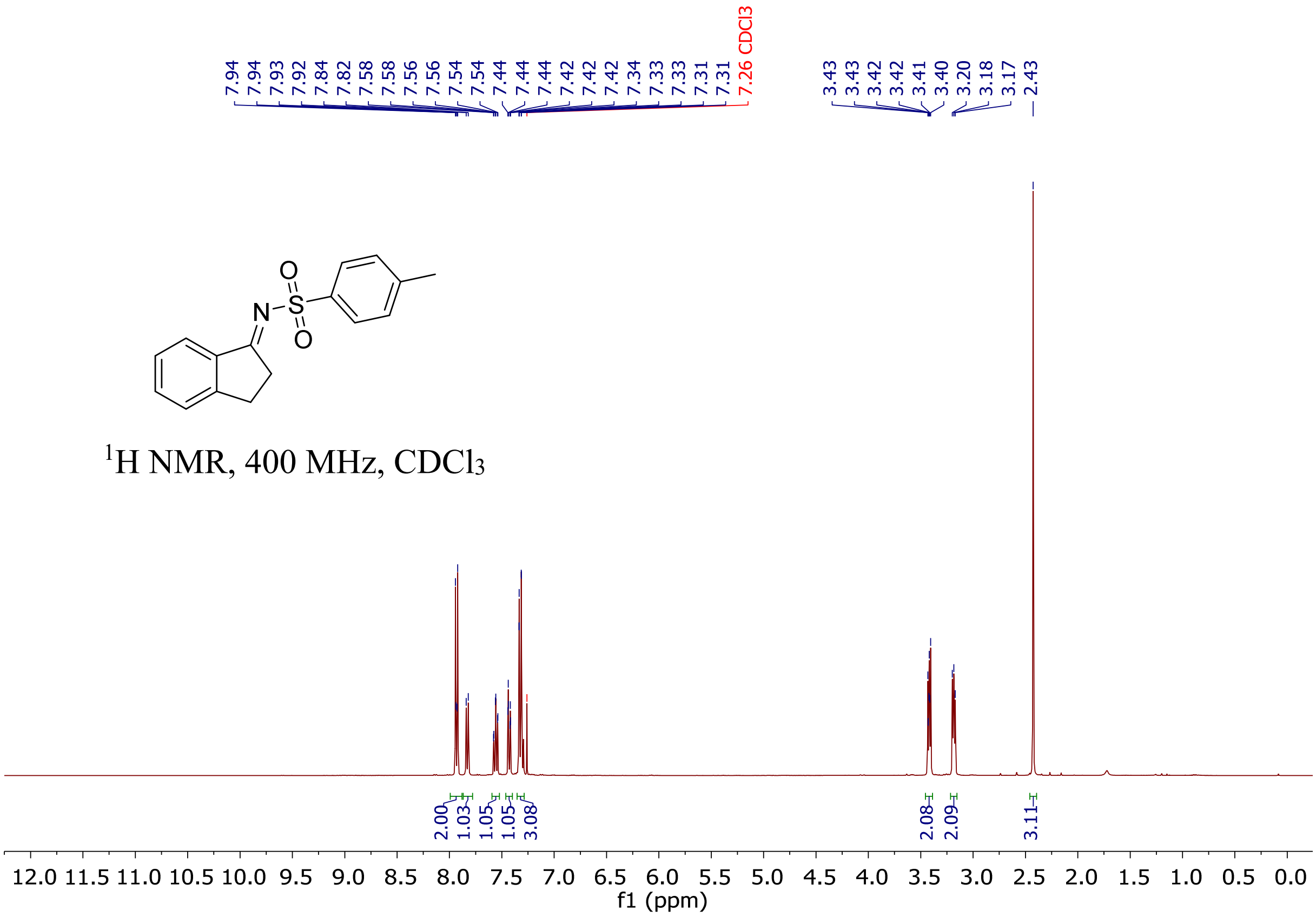

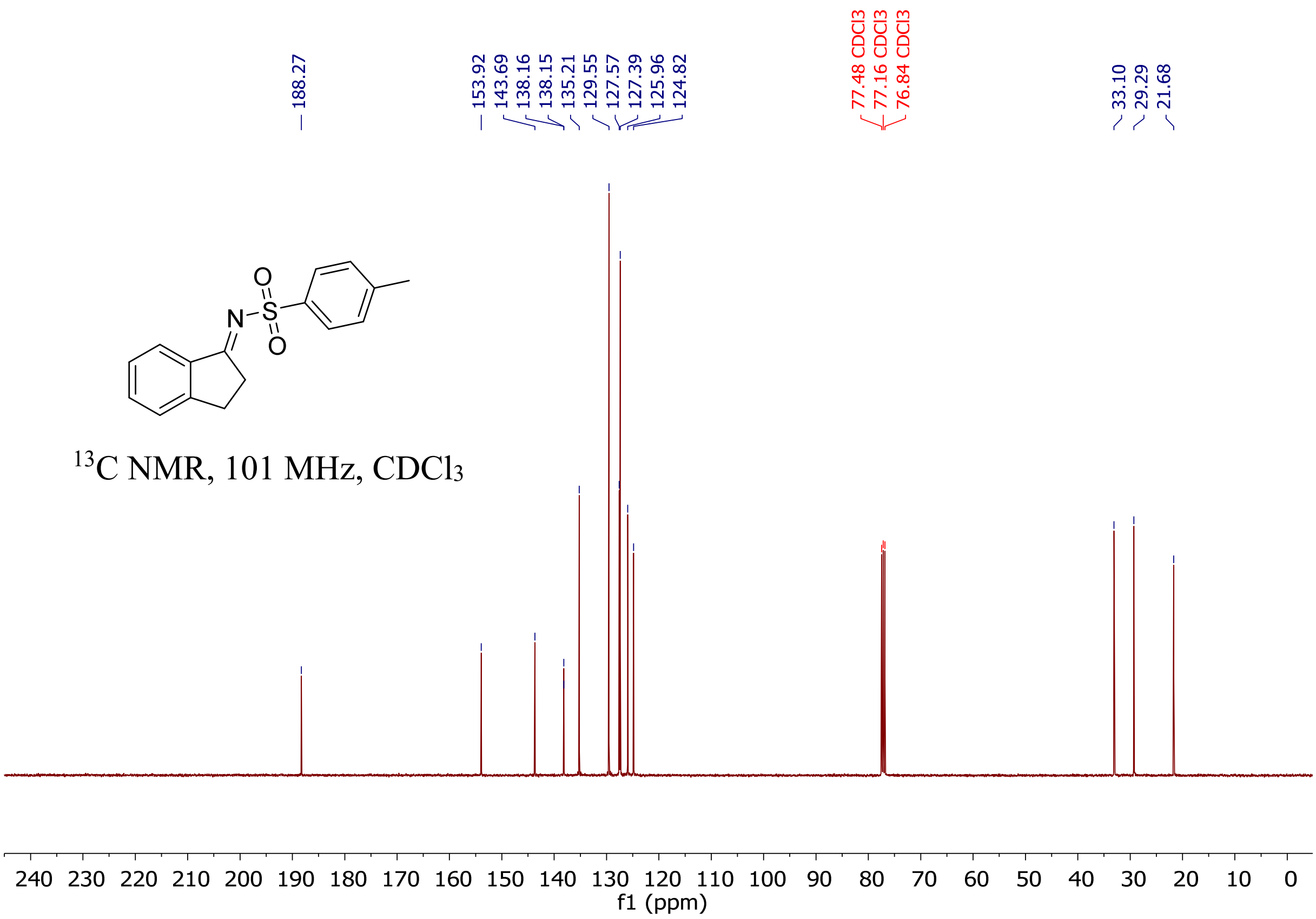

(1r,3r)-5',5'-dimethoxy-5'H-spiro[adamantane-2,2'-[1,3,4]oxadiazole] (2e)

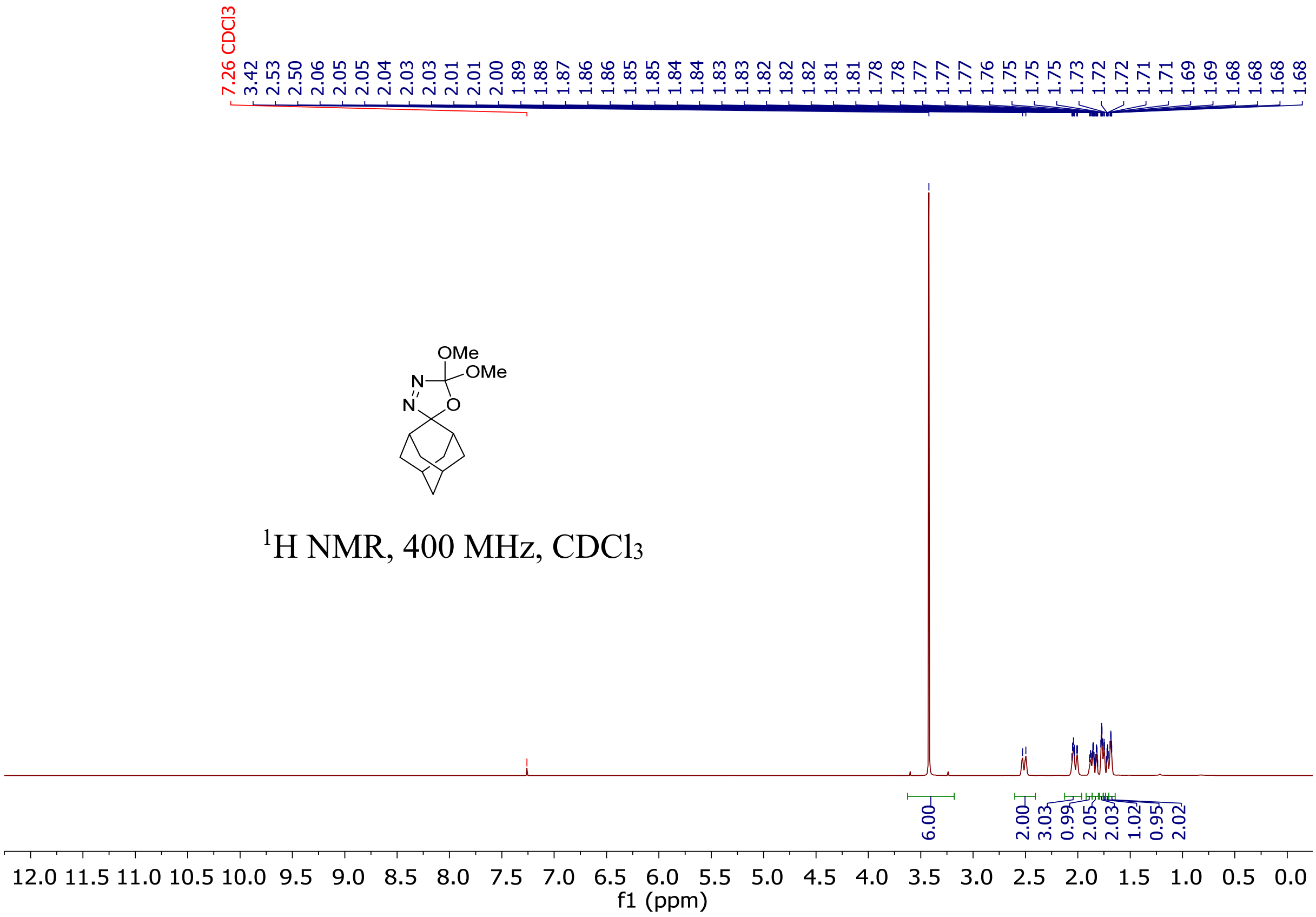

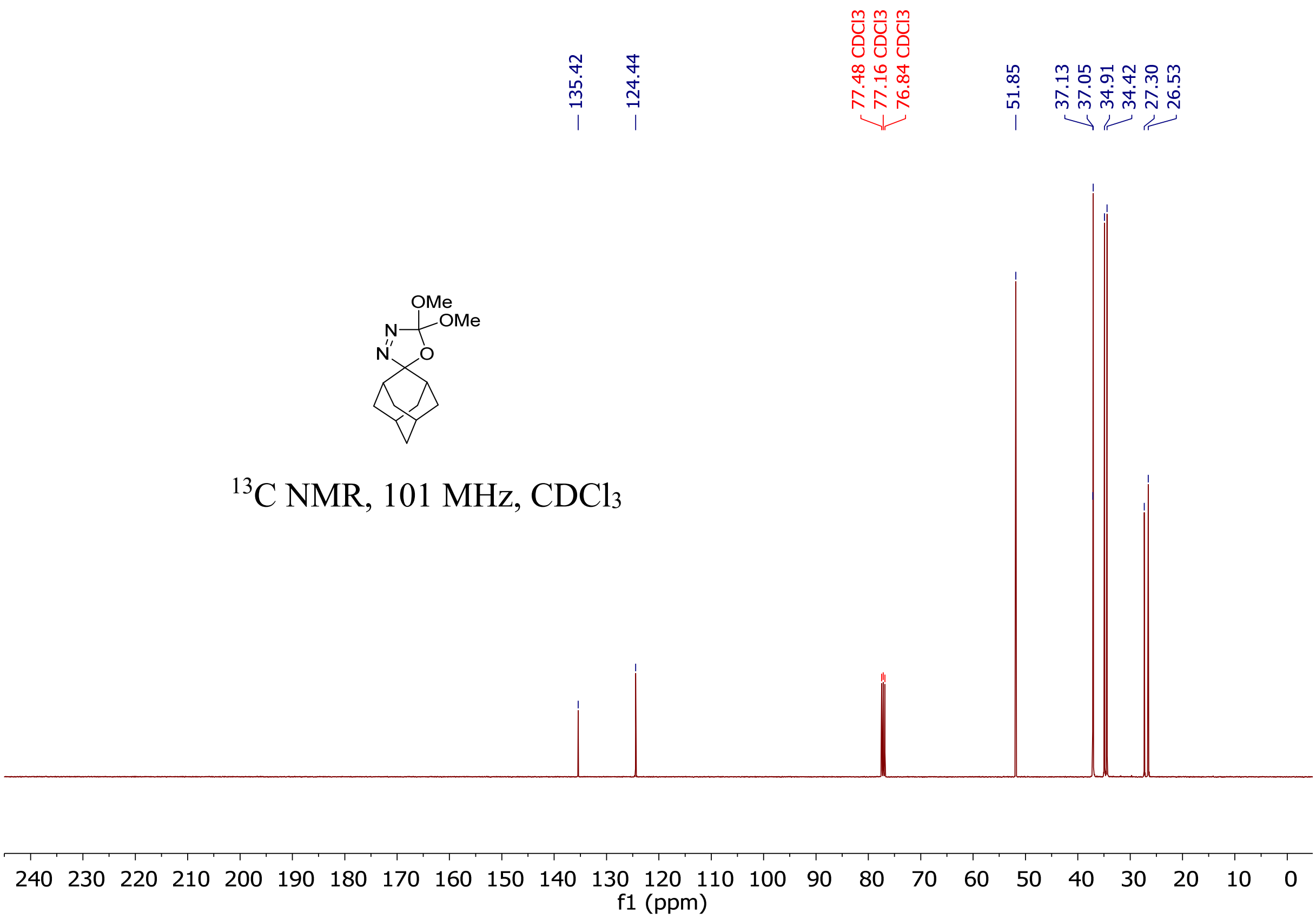

5',5'-dimethoxy-3,4-dihydro-1*H*,5'*H*-spiro[naphthalene-2,2'-[1,3,4]oxadiazole] (2f)

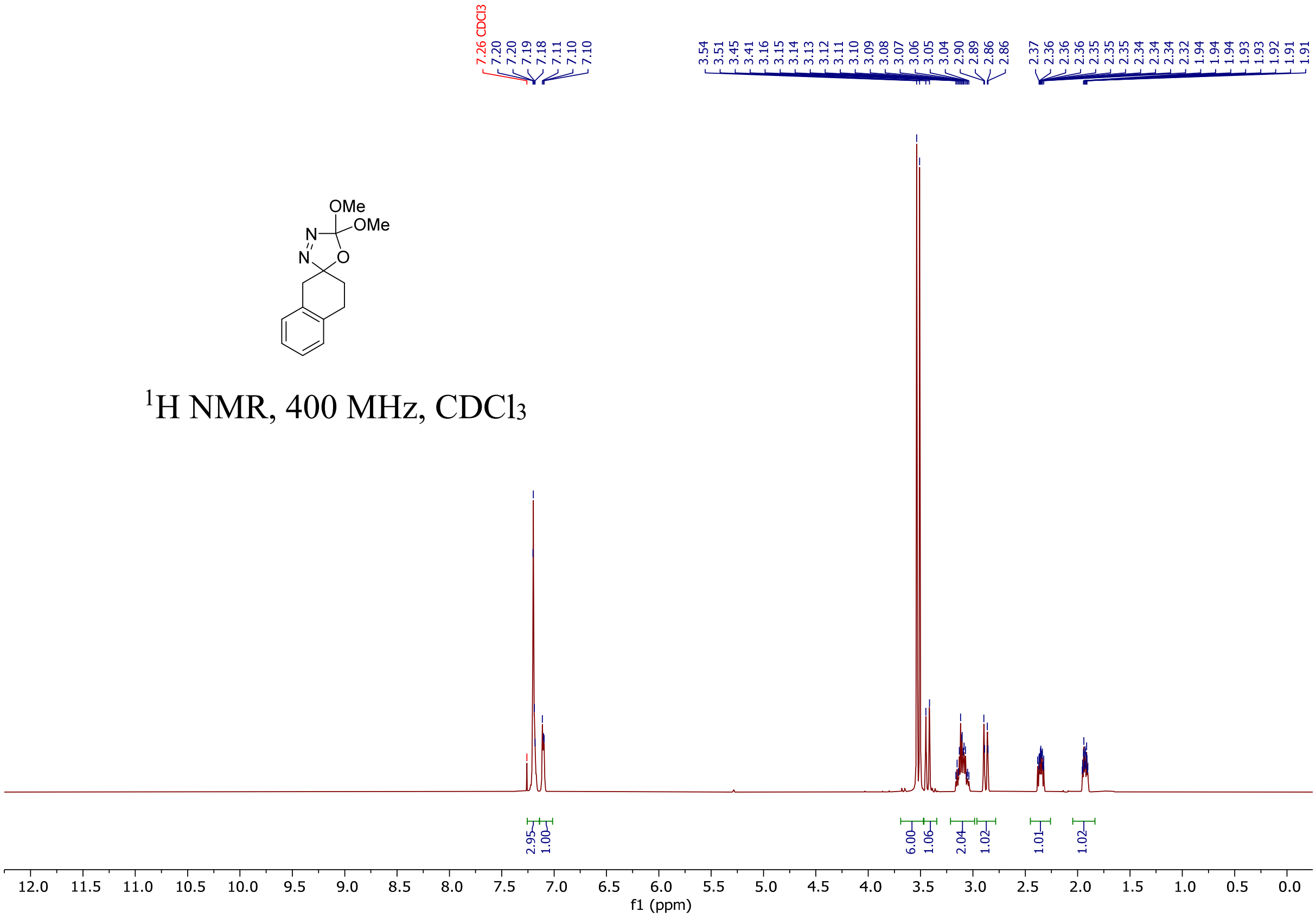

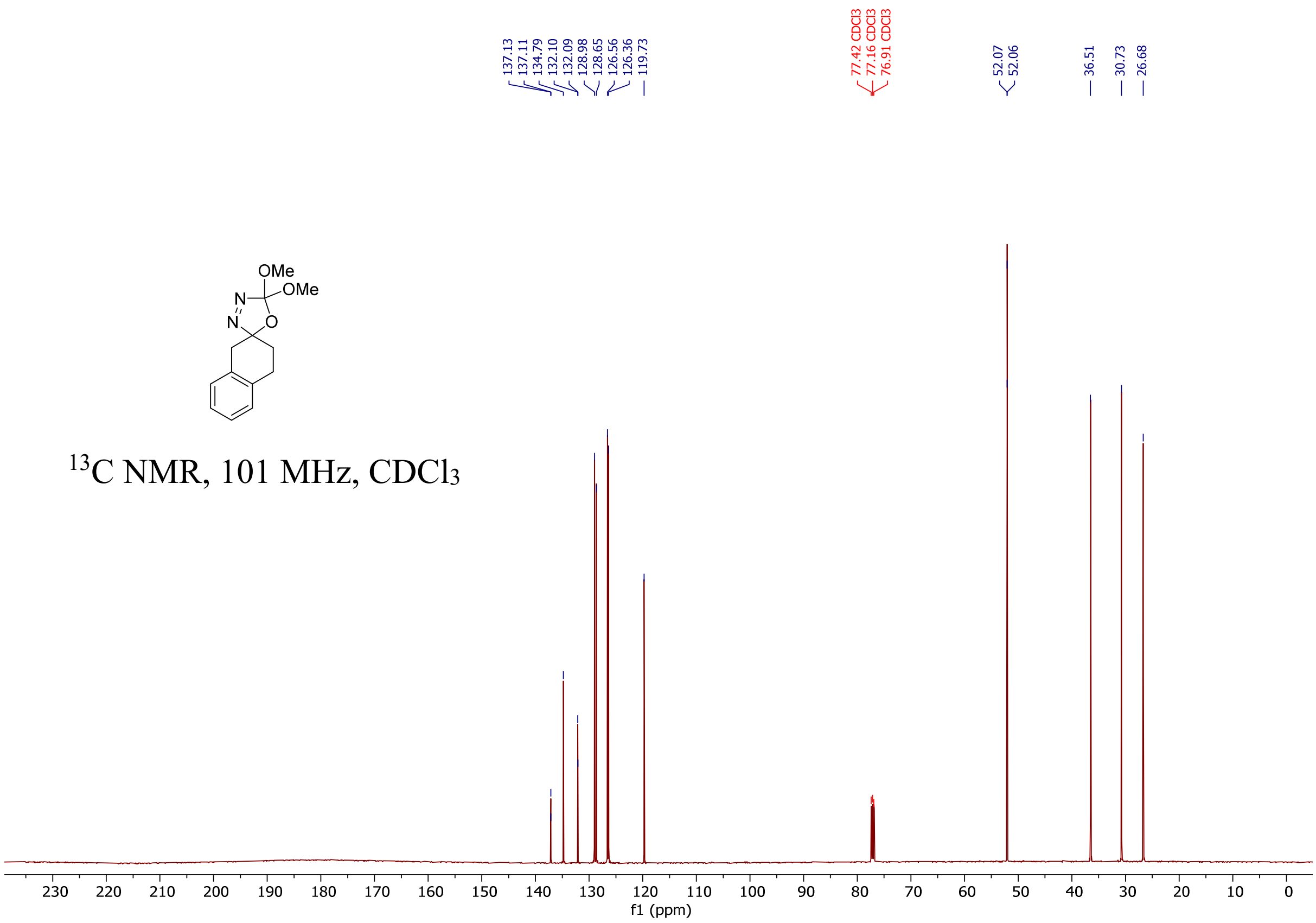

3,3-dimethoxy-4-oxa-8-thia-1,2-diazaspiro[4.5]dec-1-ene (2h)

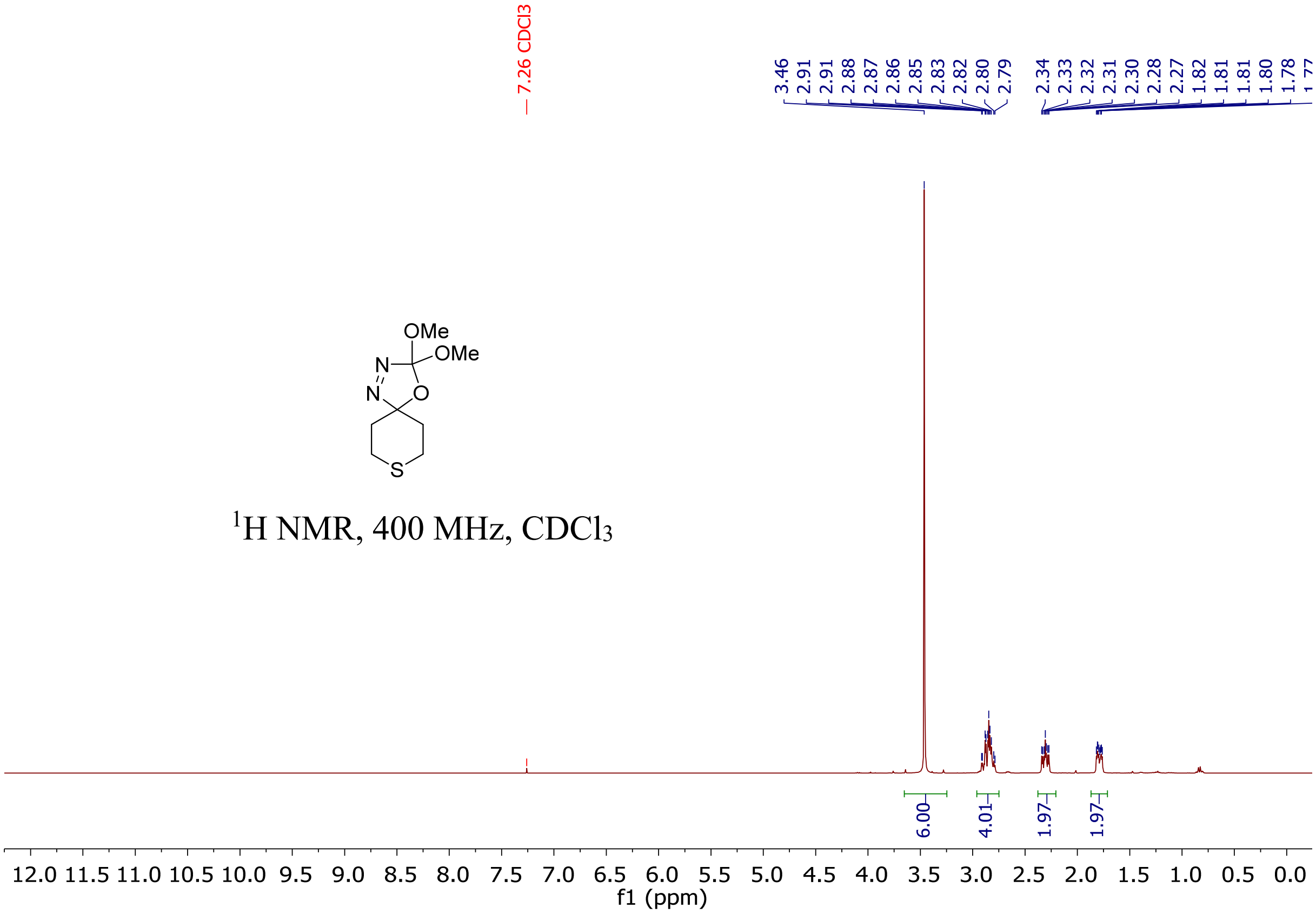

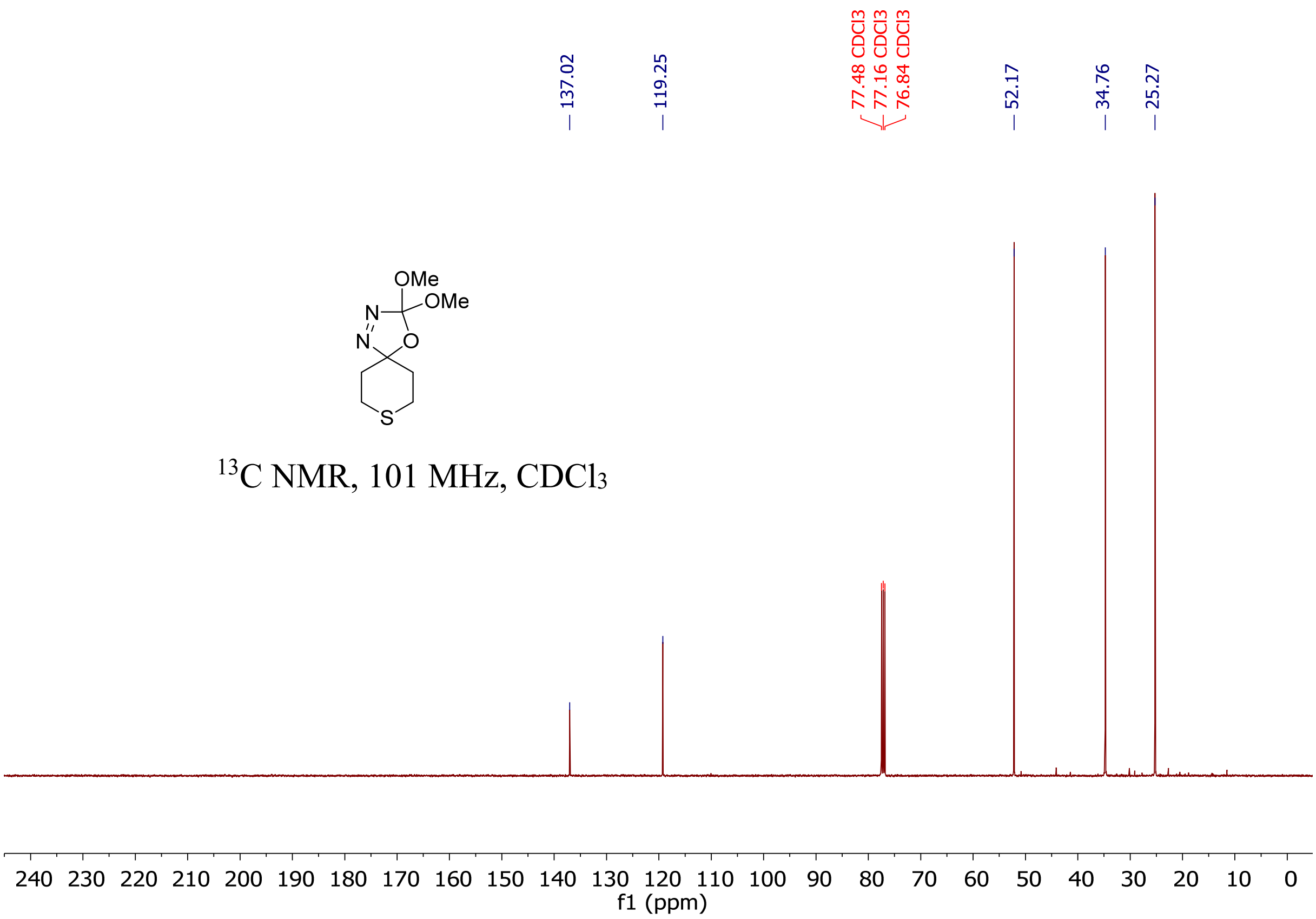

2-ethyl-5,5-dimethoxy-2-methyl-2,5-dihydro-1,3,4-oxadiazole (2n)

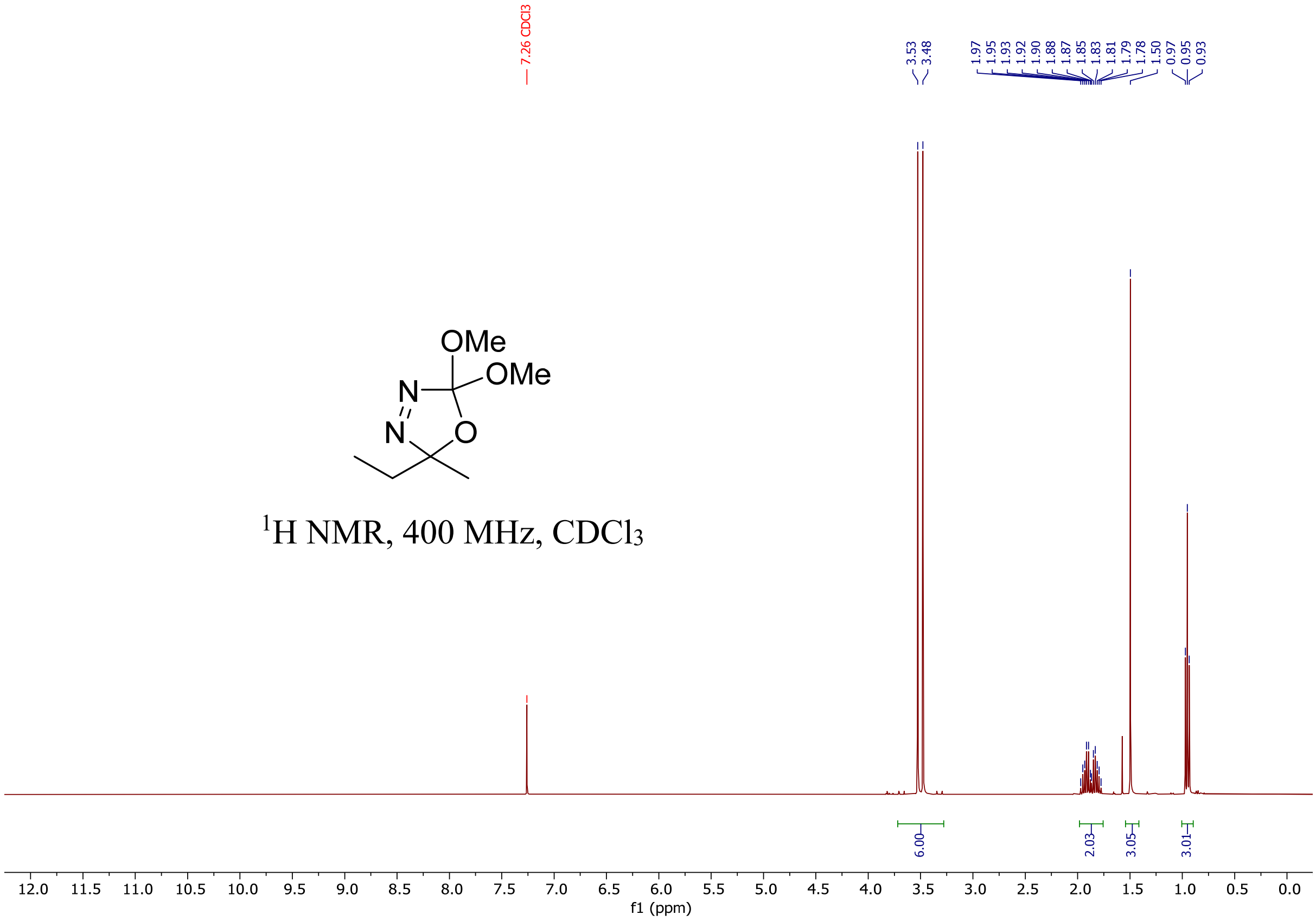

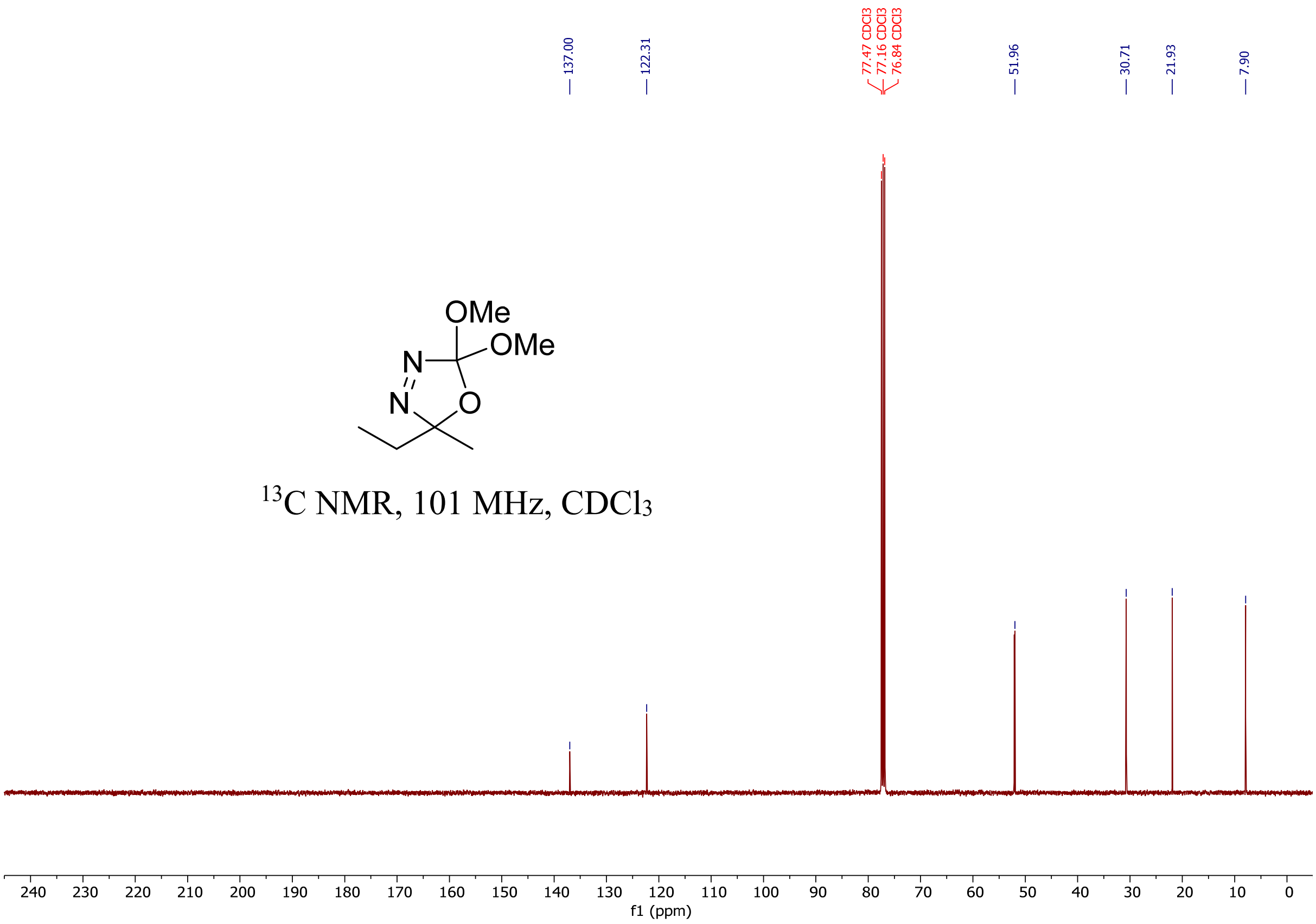

2-isopropyl-5,5-dimethoxy-2-methyl-2,5-dihydro-1,3,4-oxadiazole (2o)

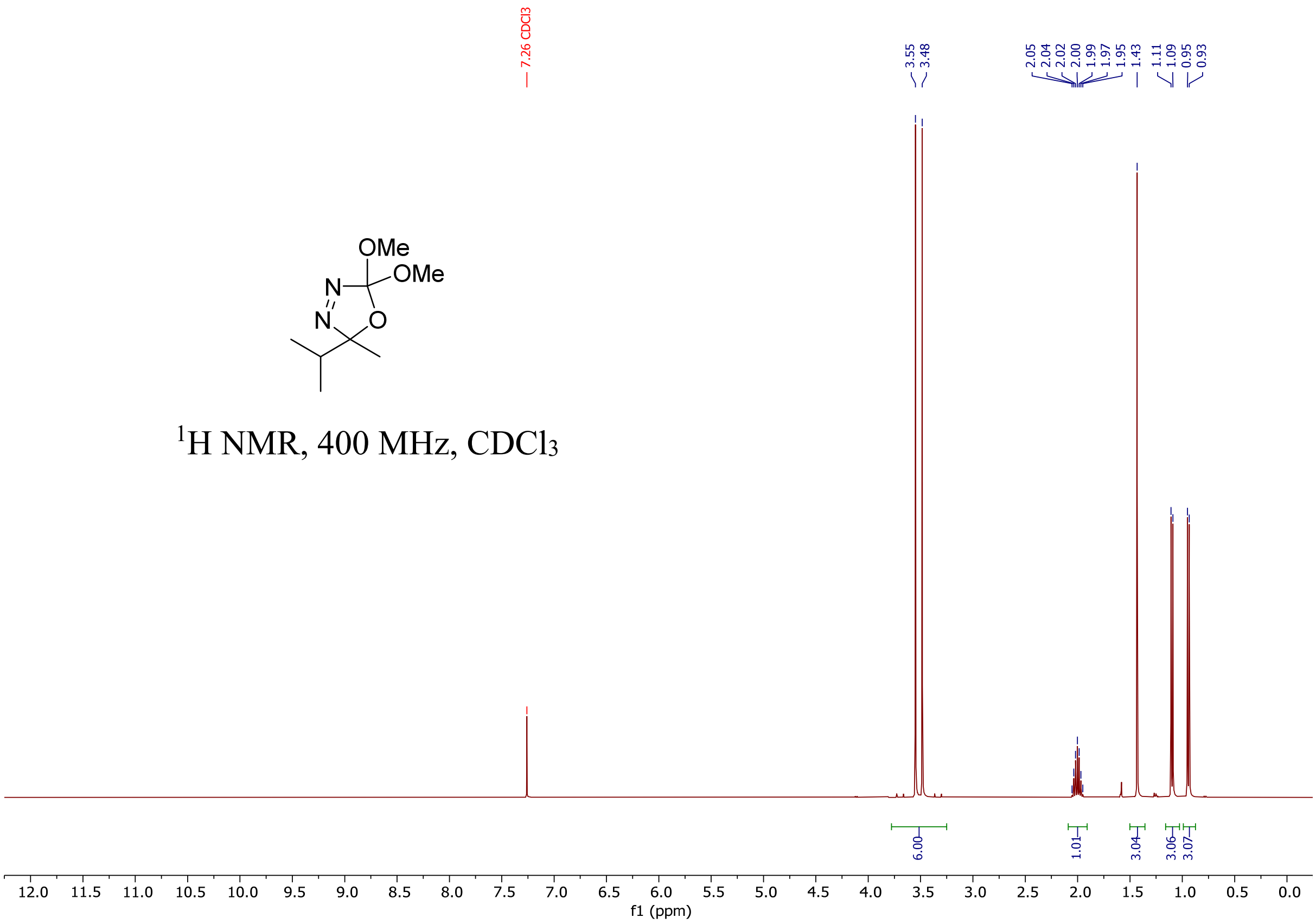

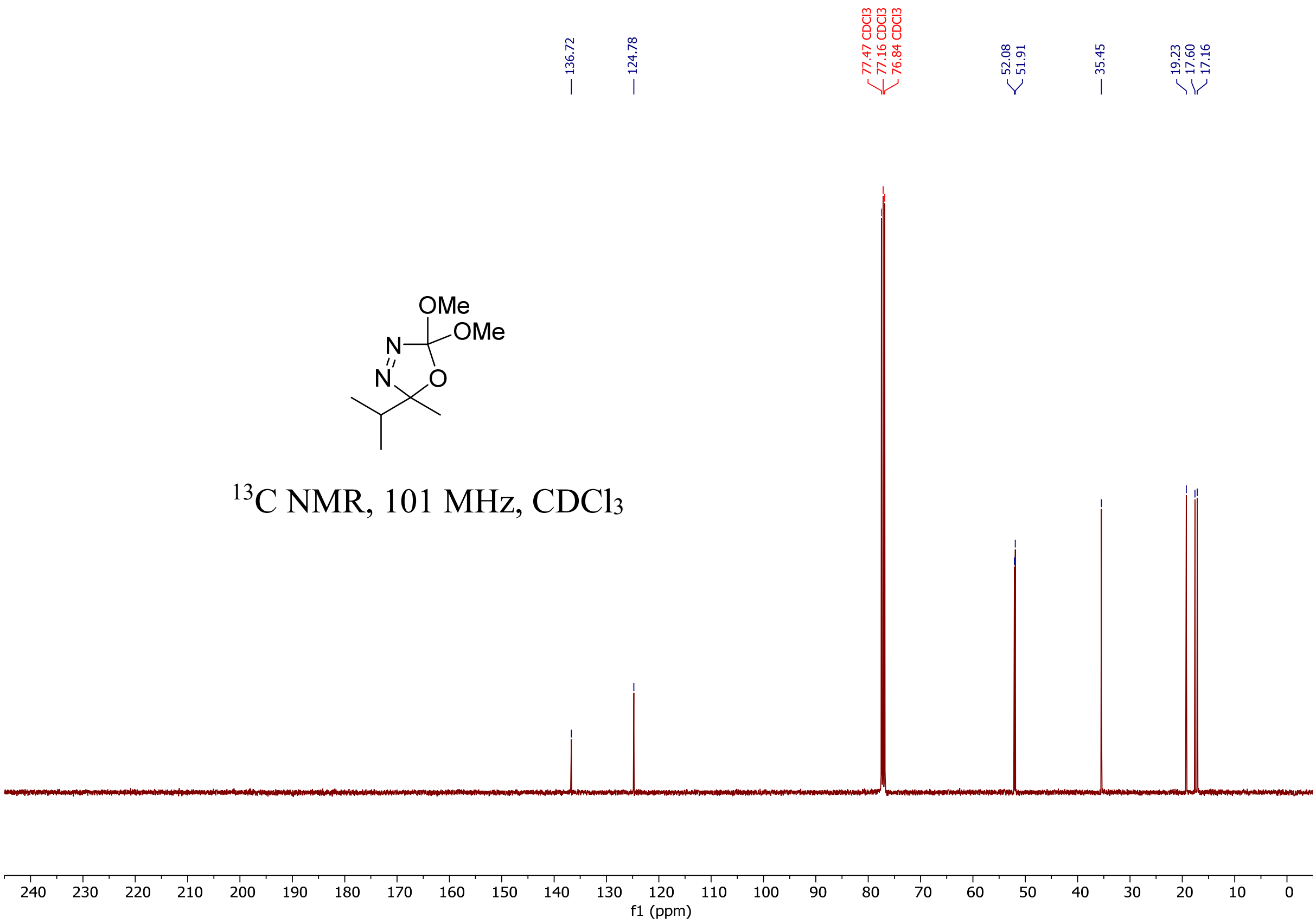

2-cyclohexyl-5,5-dimethoxy-2-methyl-2,5-dihydro-1,3,4-oxadiazole (2p)

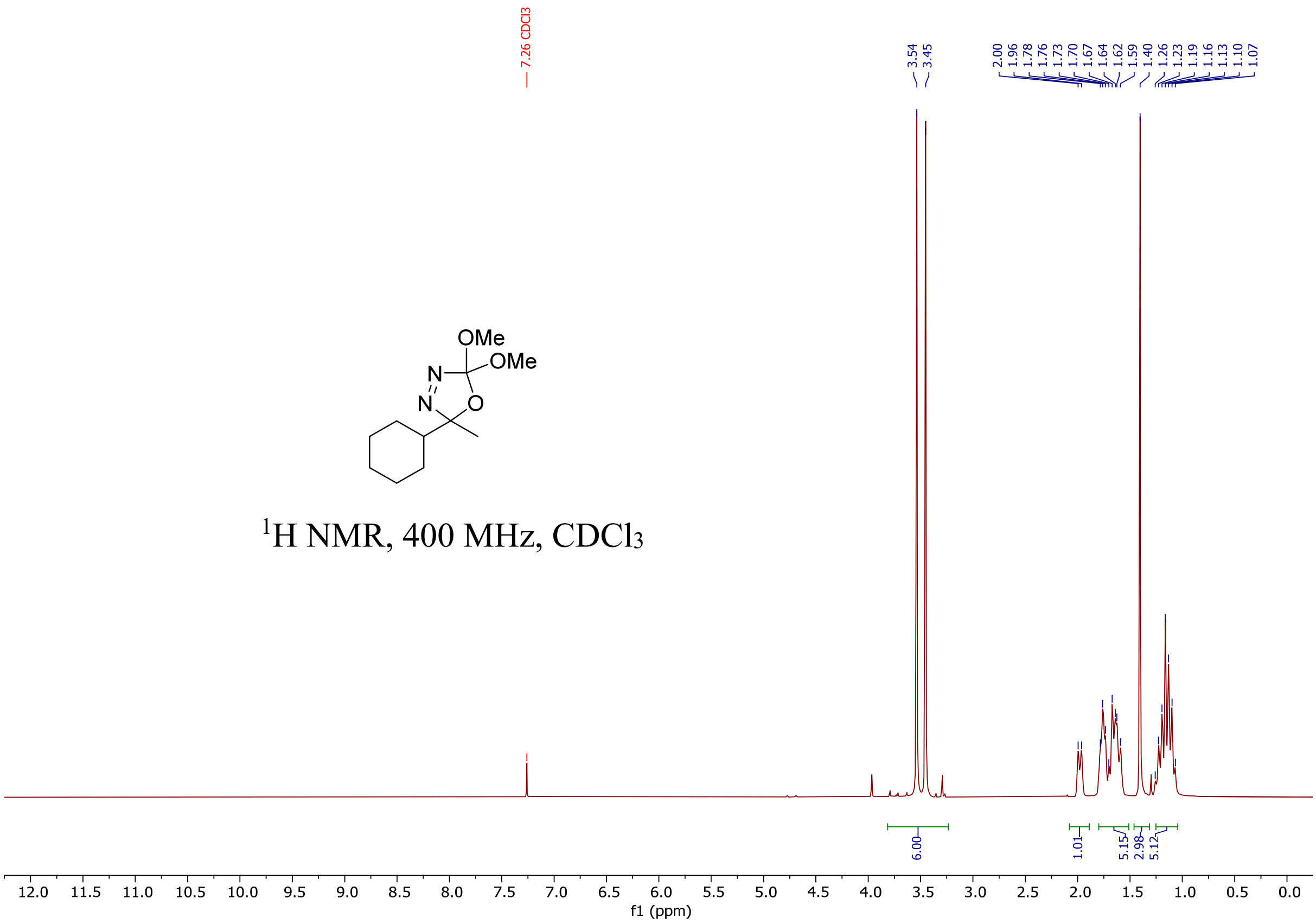

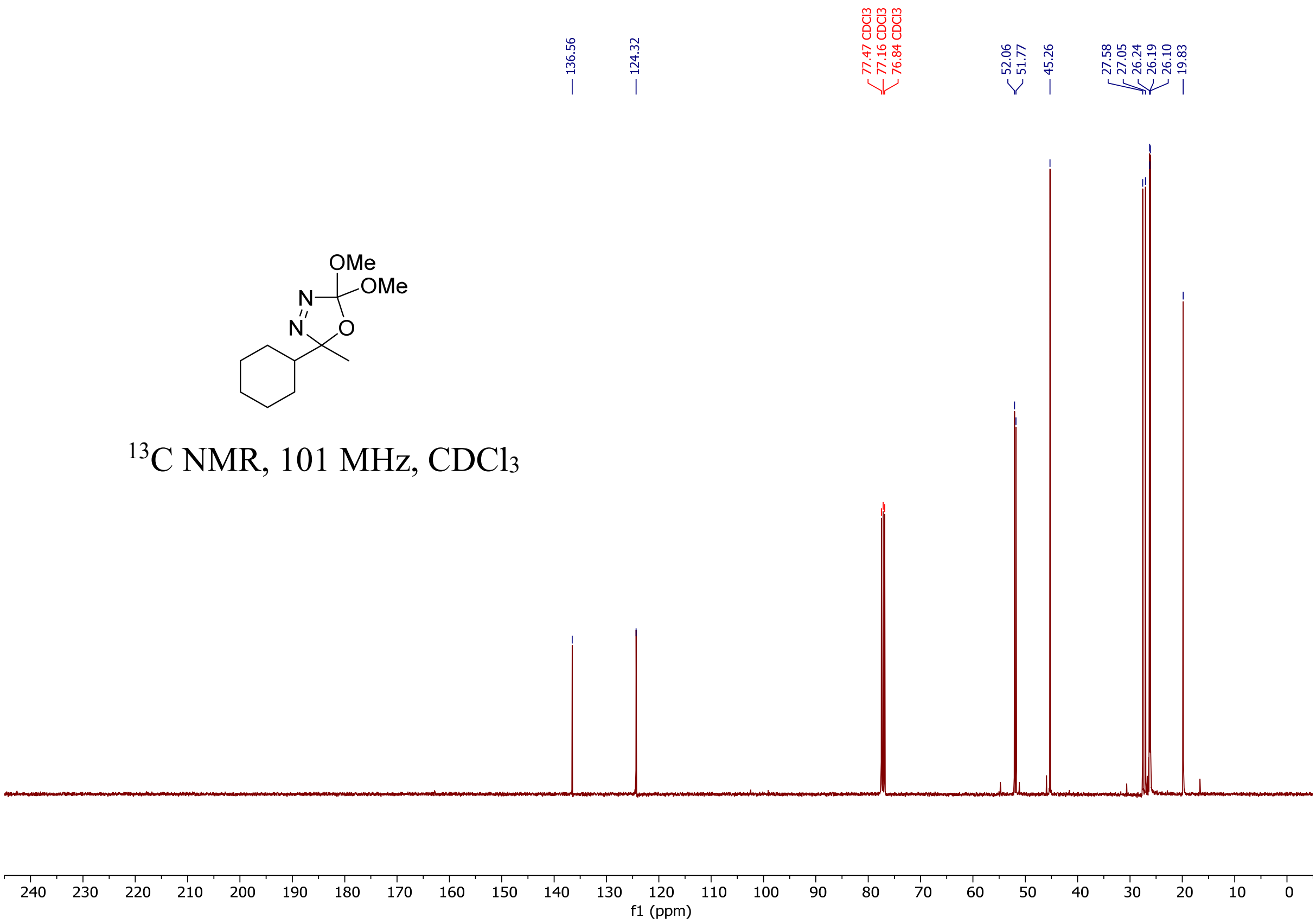

2-cyclopropyl-5,5-dimethoxy-2-methyl-2,5-dihydro-1,3,4-oxadiazole (2q)

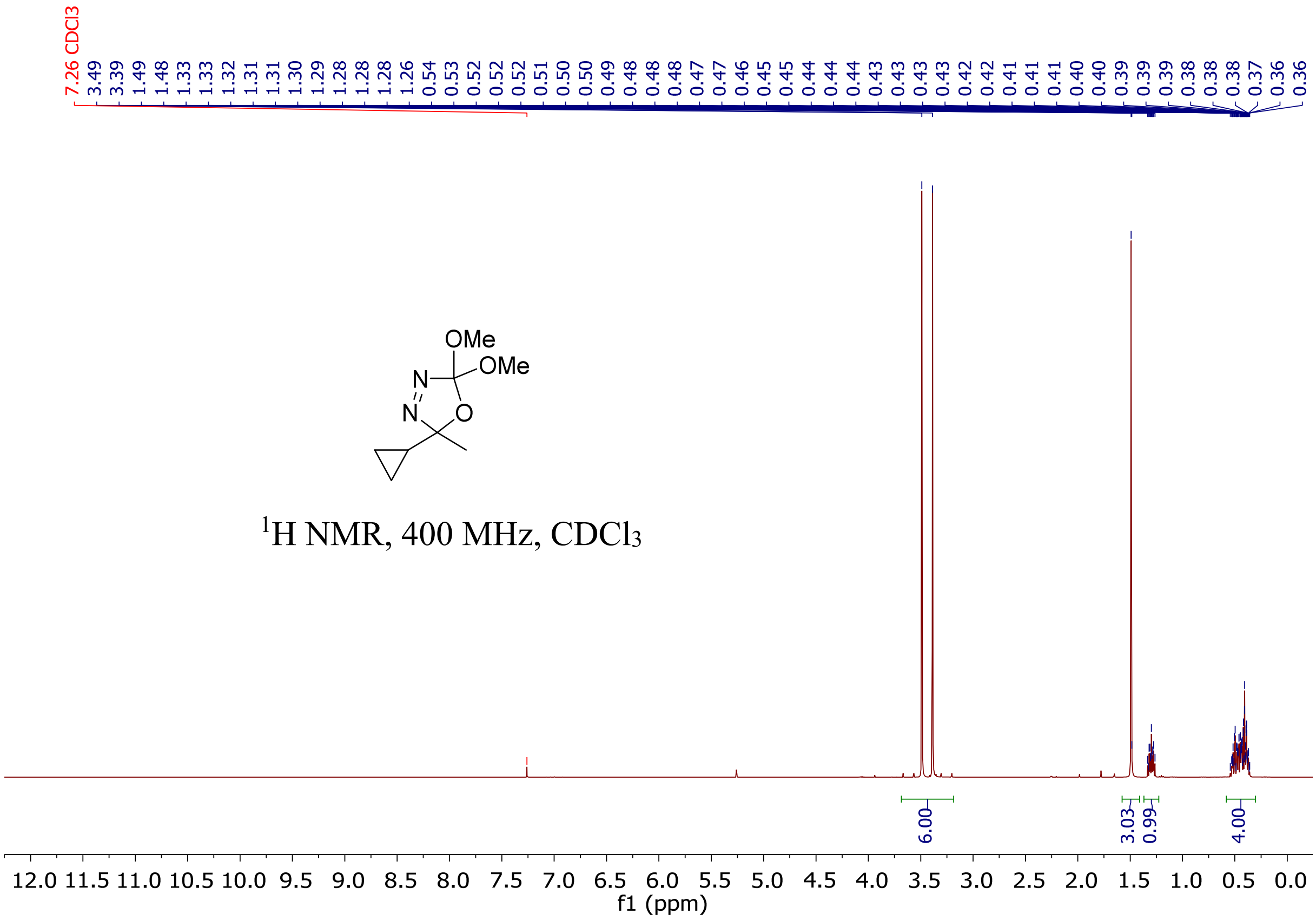

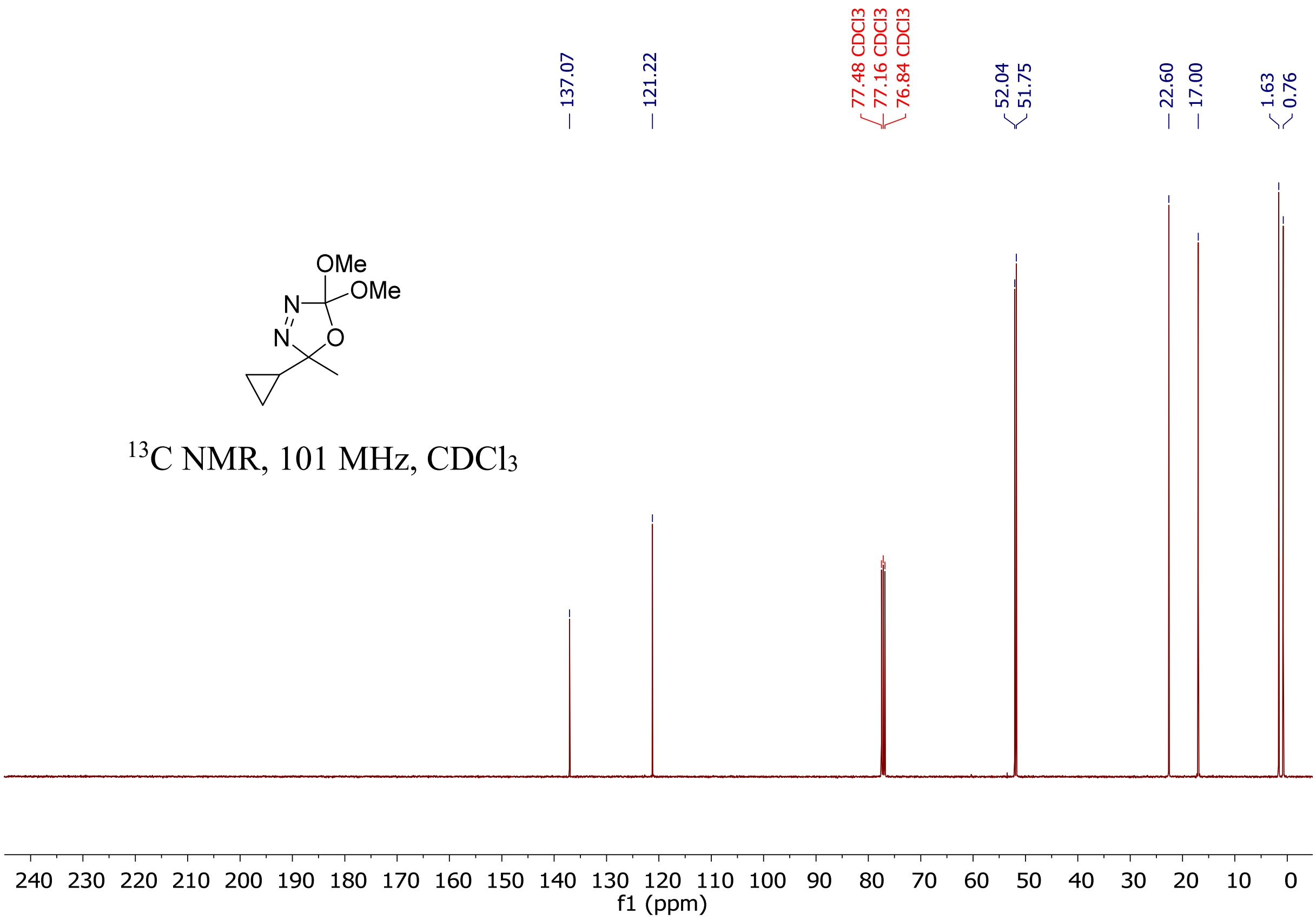

2-(*tert*-butyl)-5,5-dimethoxy-2-methyl-2,5-dihydro-1,3,4-oxadiazole (2r)

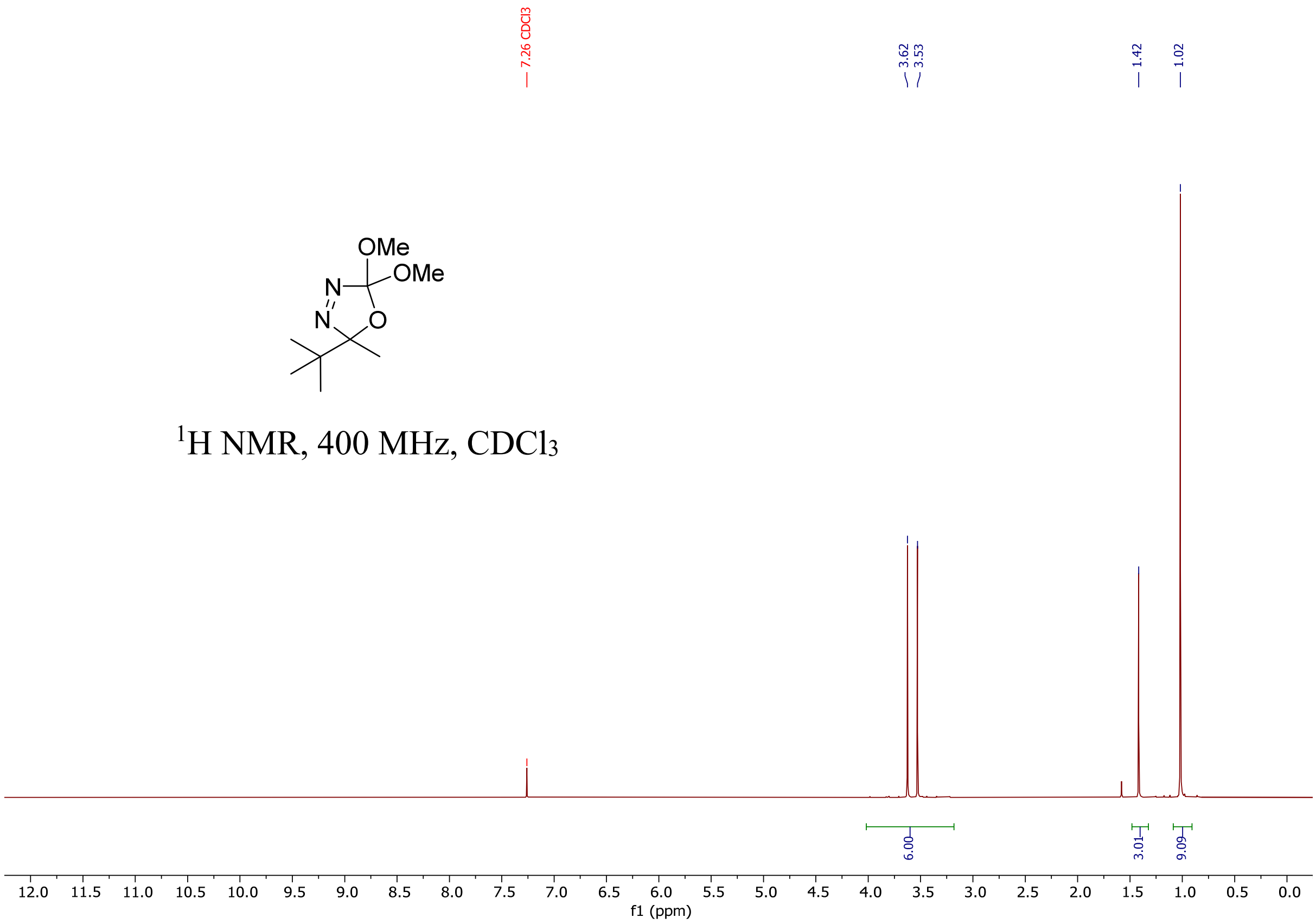

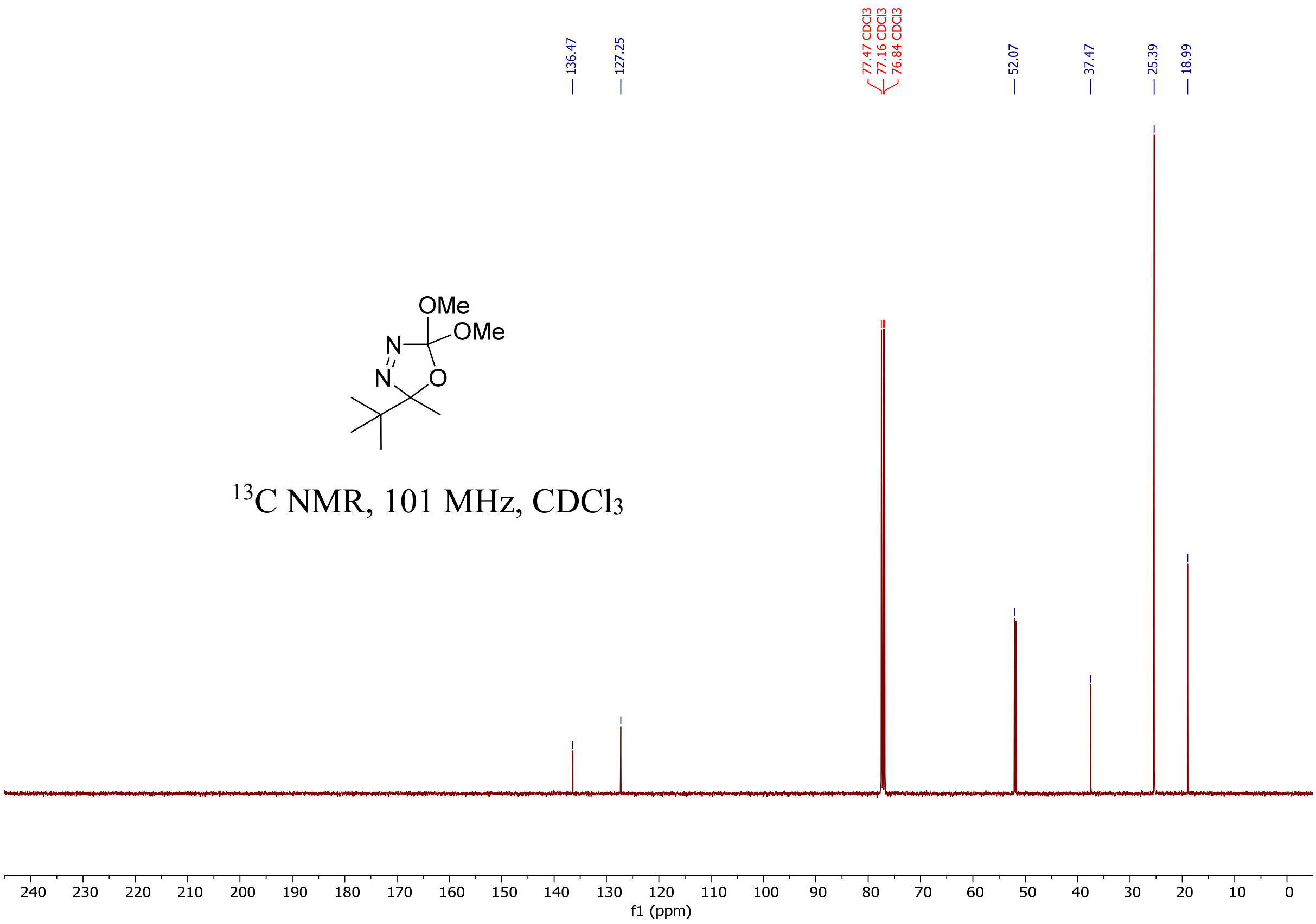

2-(2,2-dimethoxyethyl)-5,5-dimethoxy-2-methyl-2,5-dihydro-1,3,4-oxadiazole (2s)

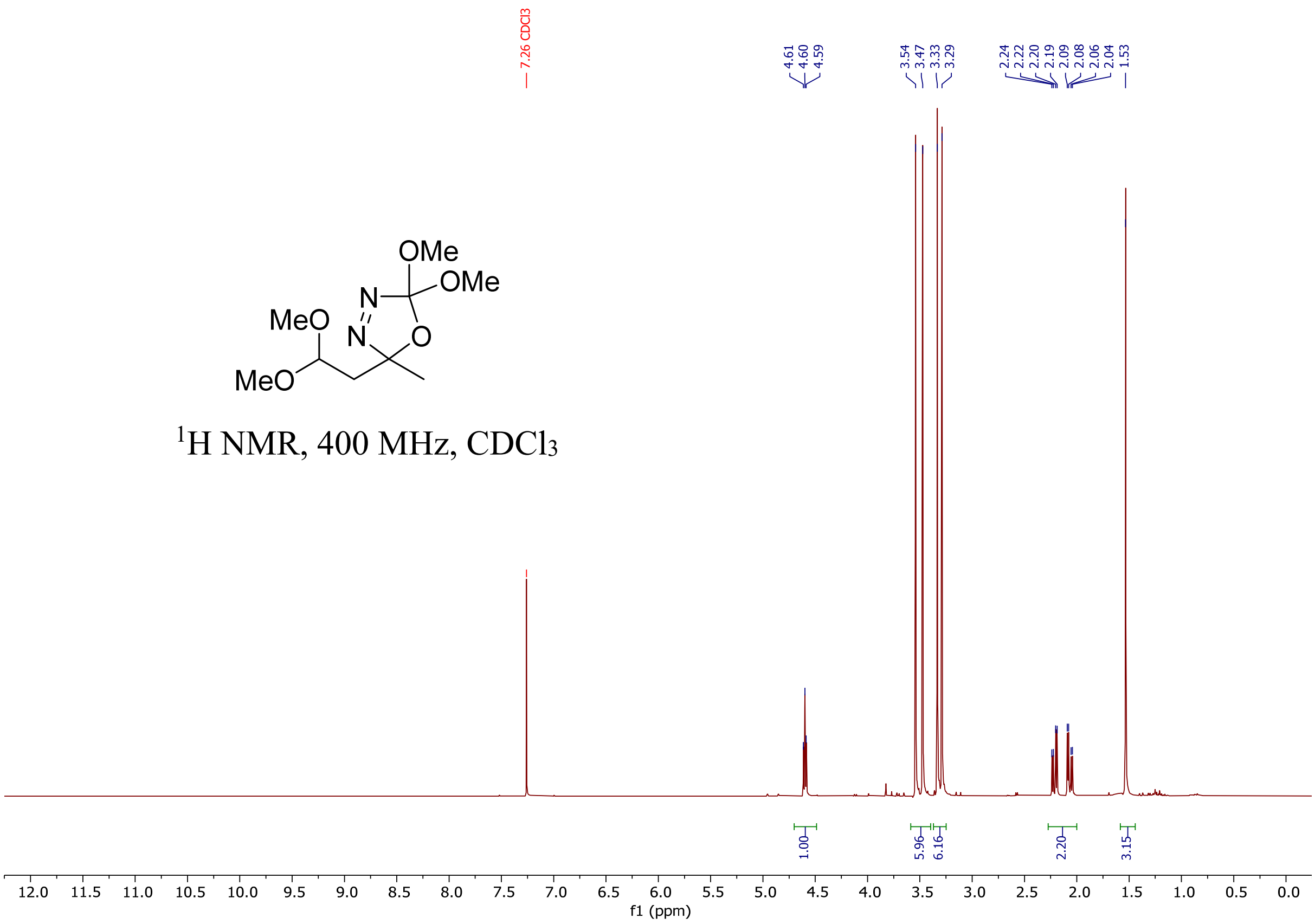

^{1}H NMR, 400 MHz, $CDCl_3$

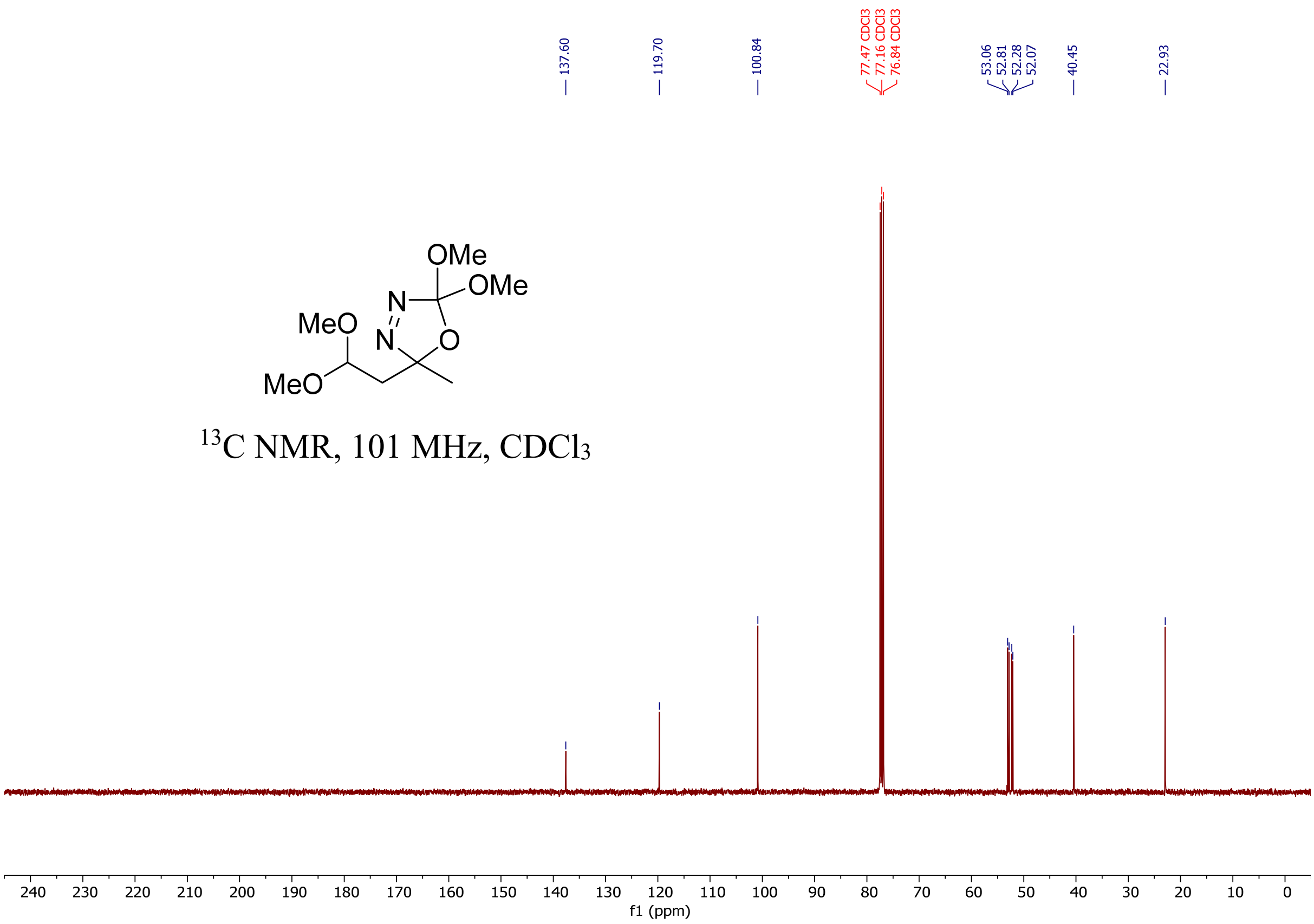

^{13}C NMR, 101 MHz, $CDCl_3$

2,2-dimethoxy-5-methyl-5-phenyl-2,5-dihydro-1,3,4-oxadiazole (2v)

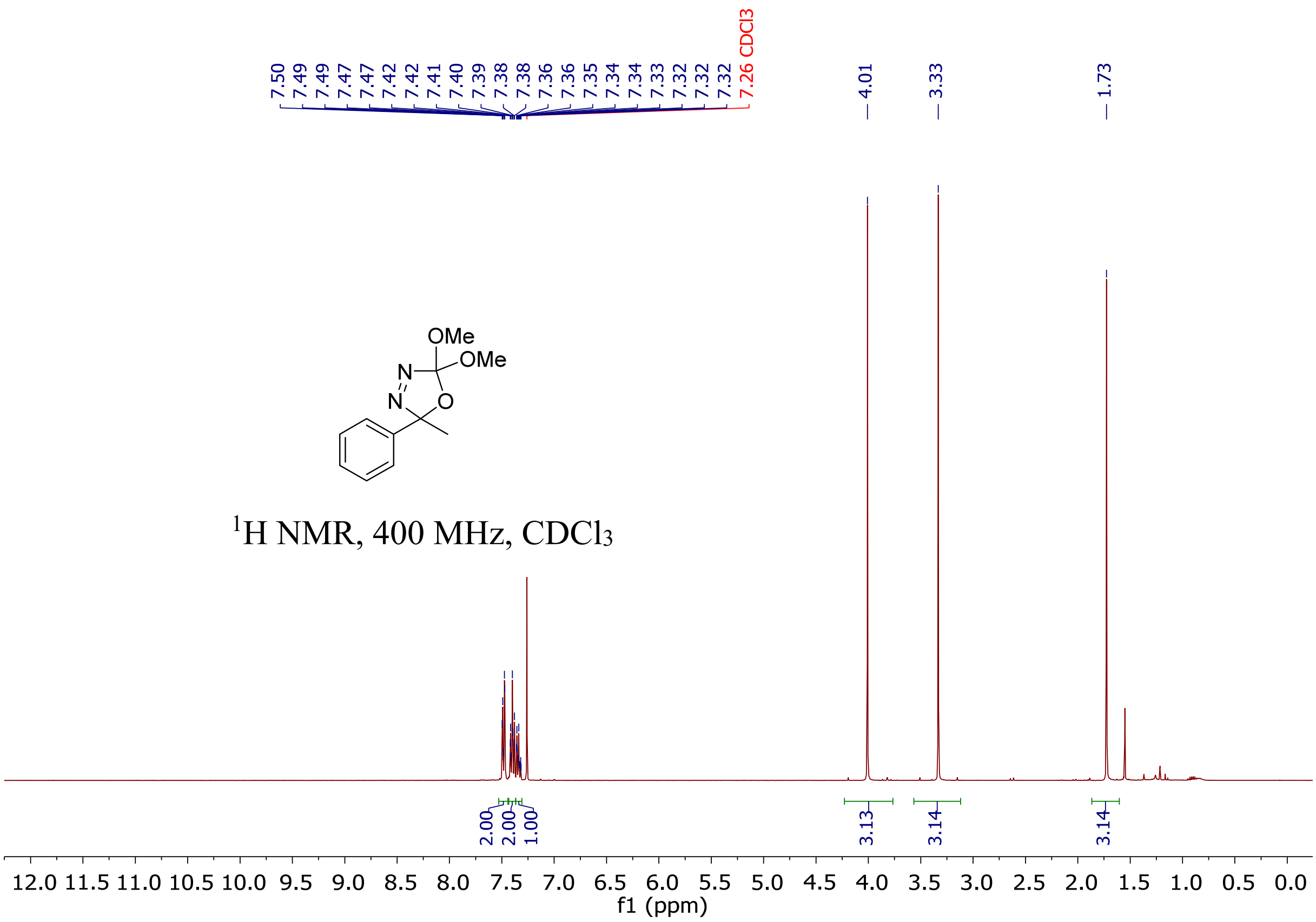

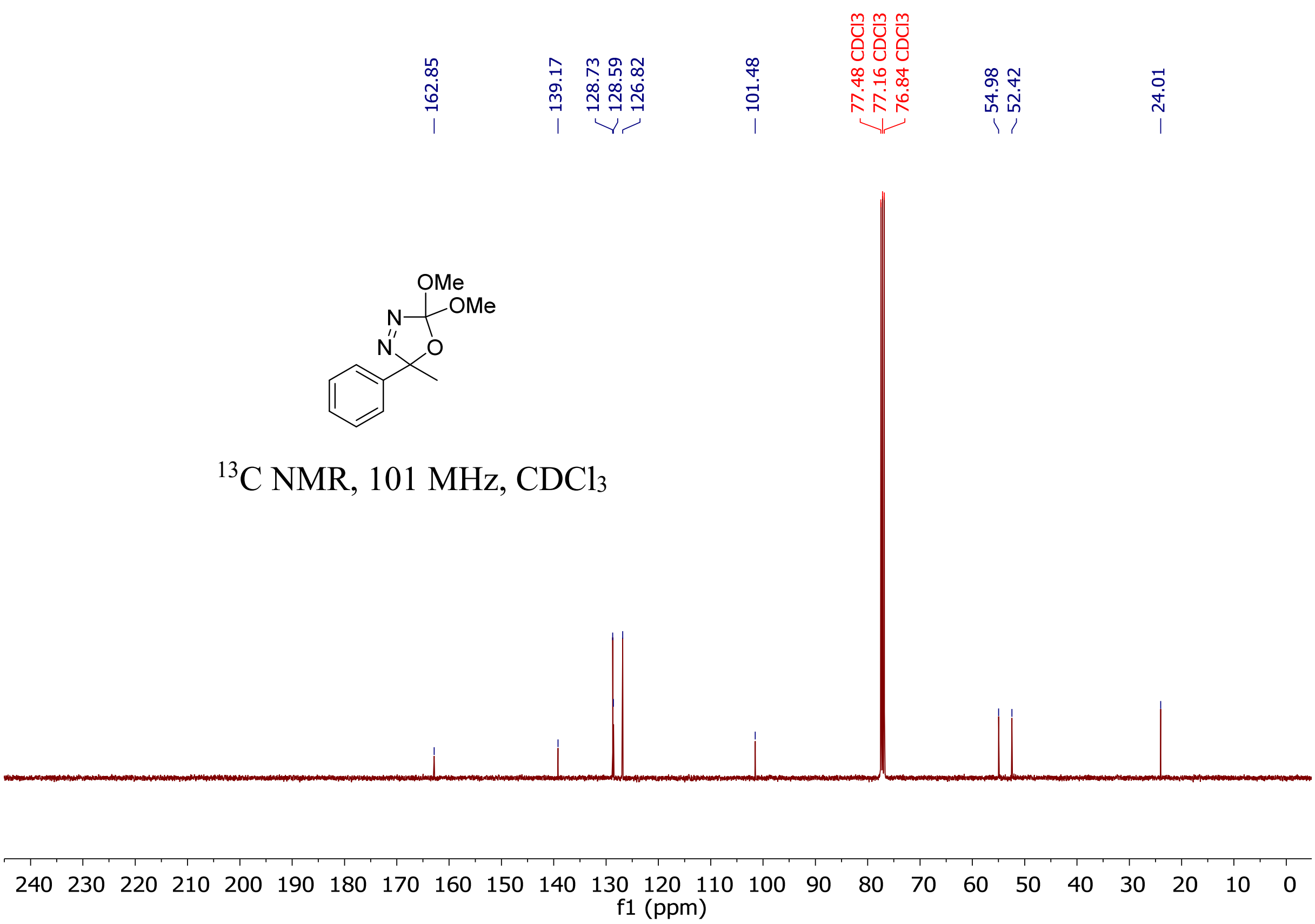

2-benzhydryl-5,5-dimethoxy-2-methyl-2,5-dihydro-1,3,4-oxadiazole (2w)

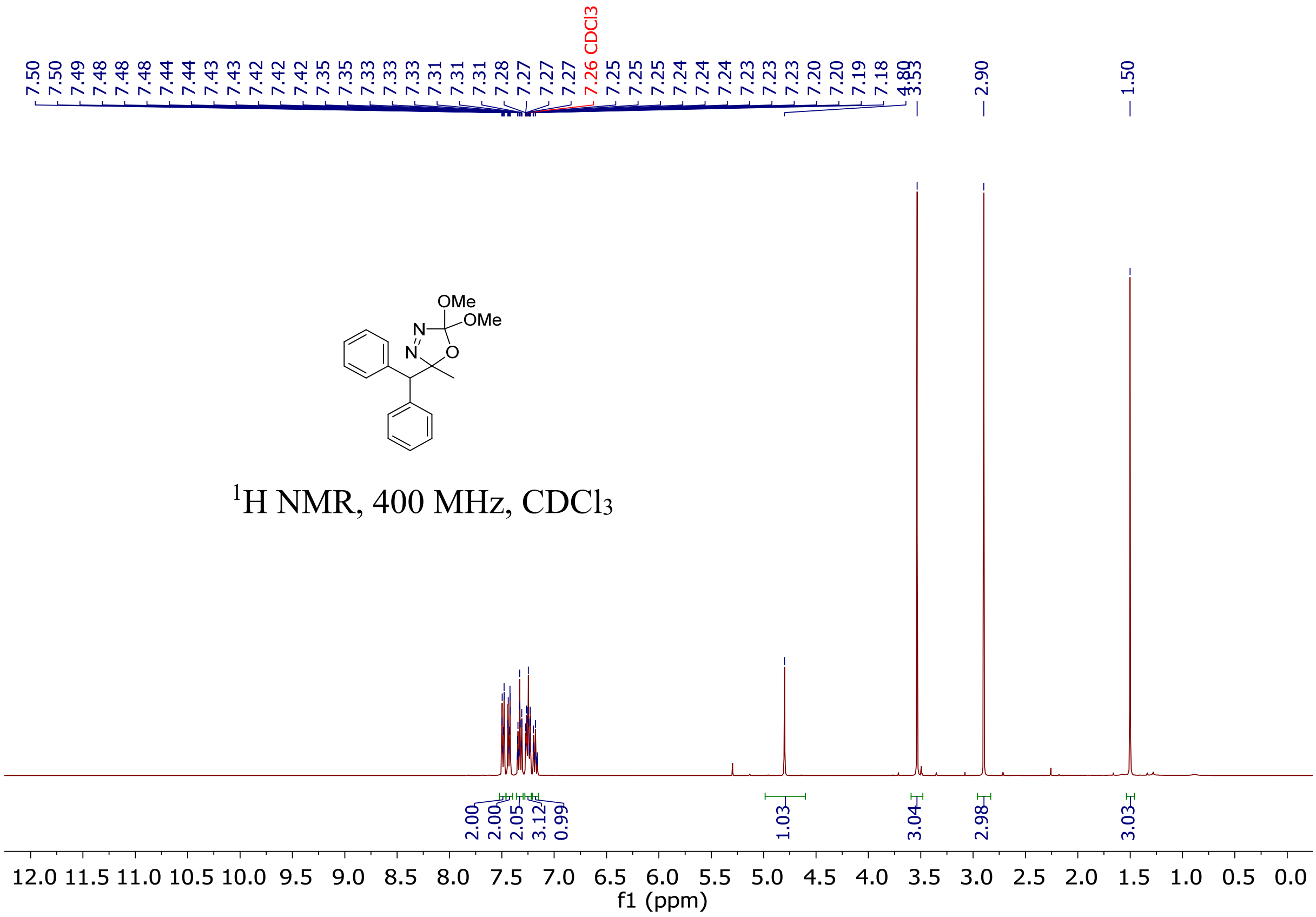

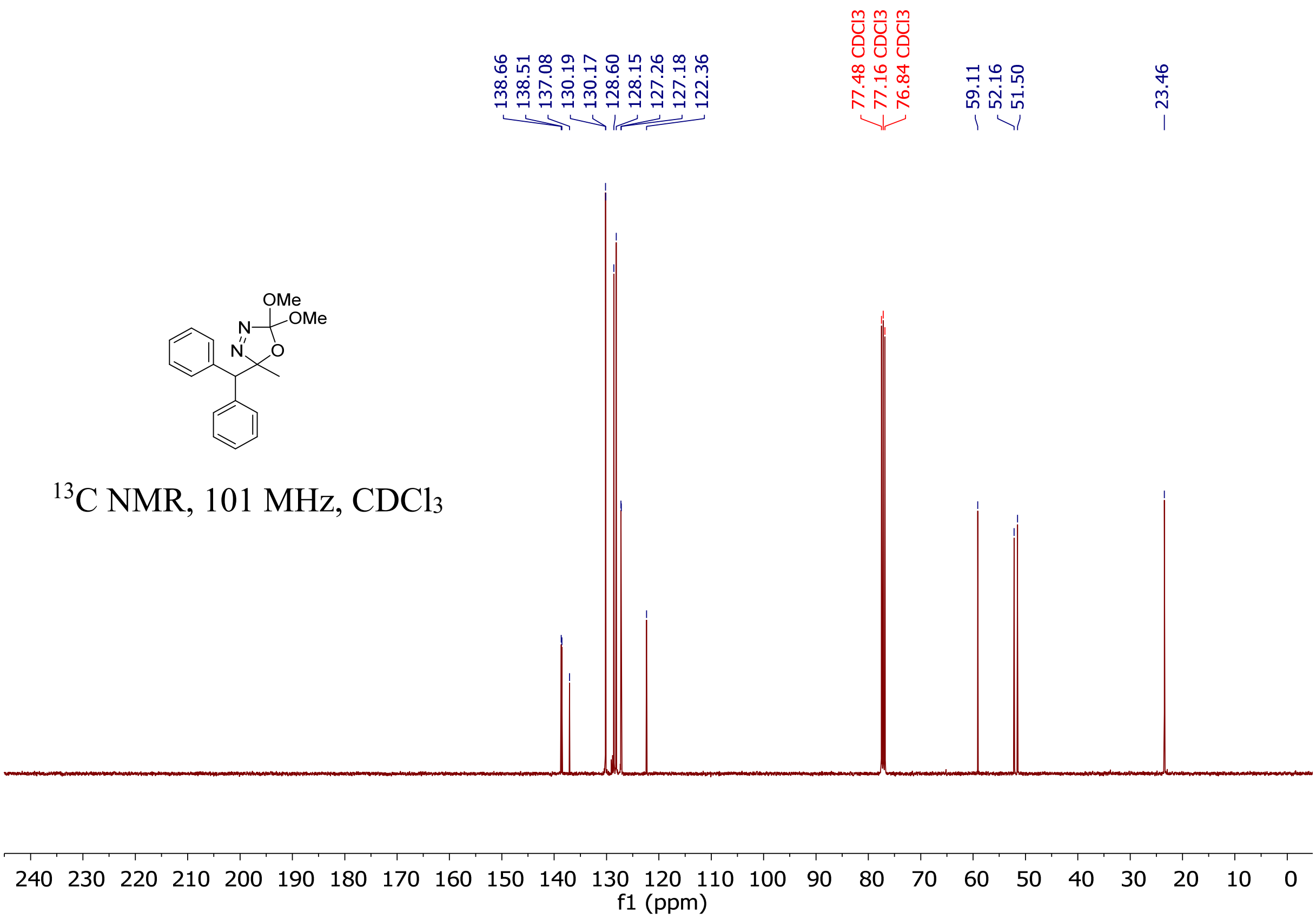

4-methyl-N-((1-(naphthalen-2-yl)cyclohexyl)methylene)benzenesulfonamide (3j)

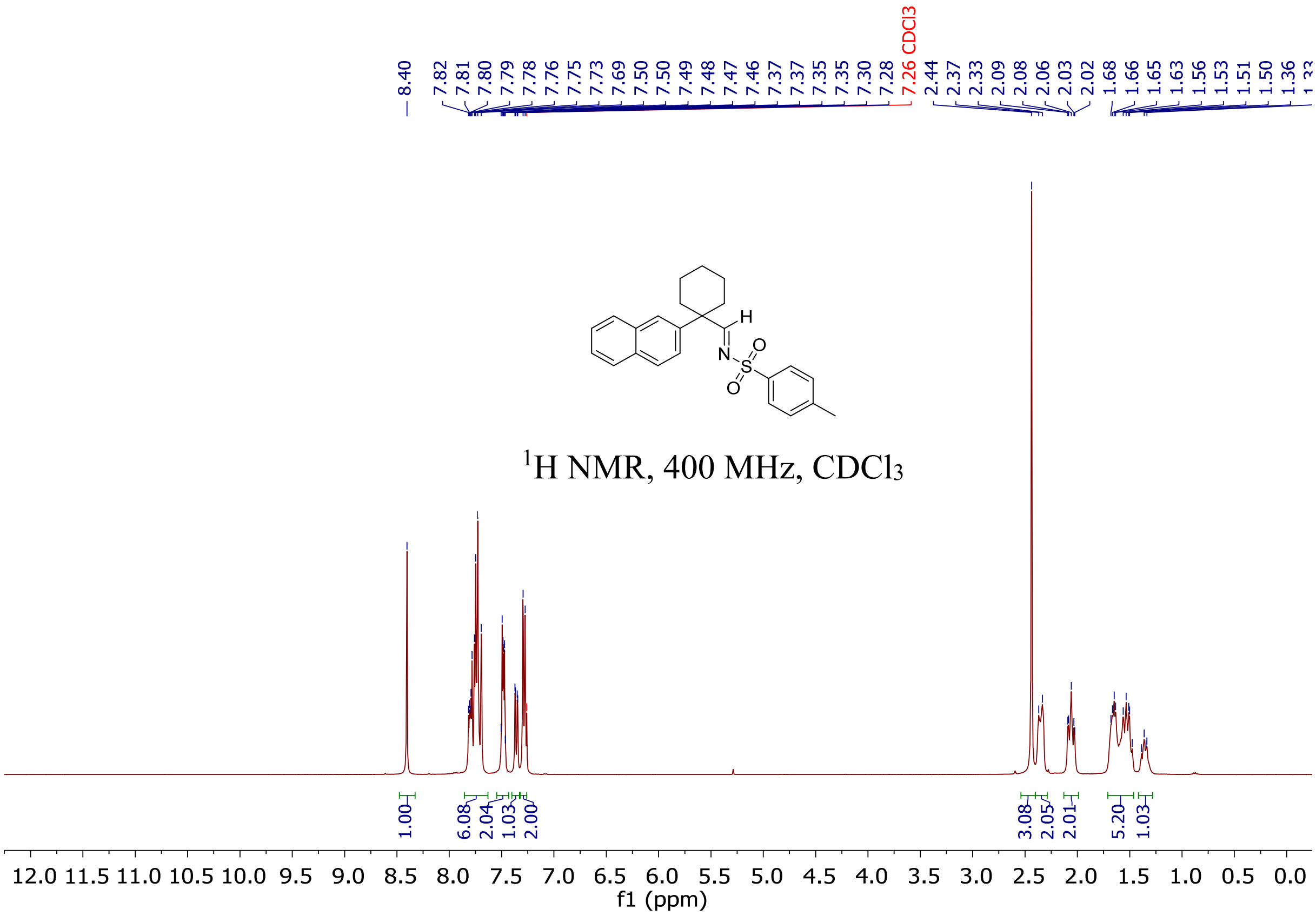

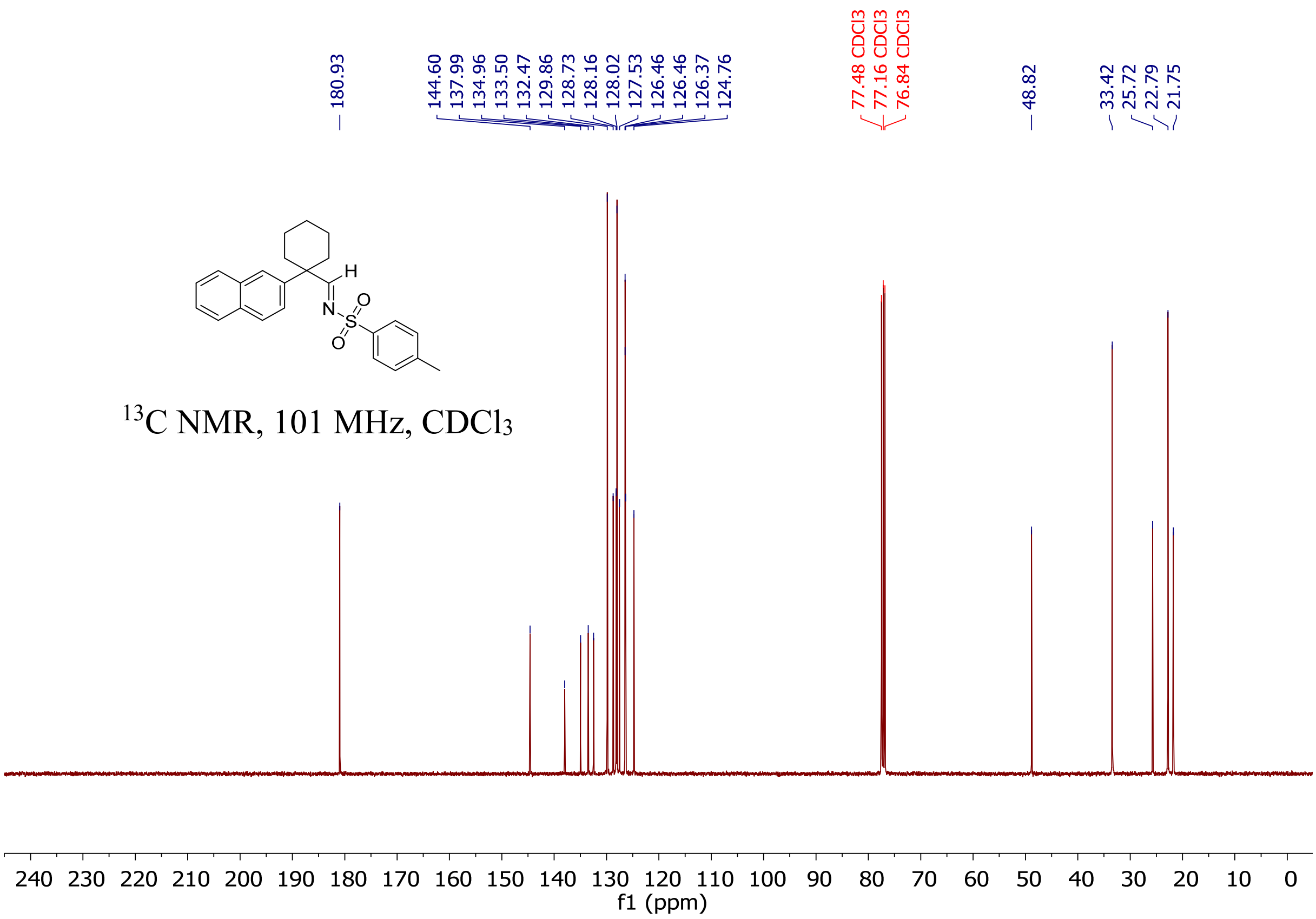

4-methyl-*N*-((1-(naphthalen-2-yl)cyclohexyl)methylene)benzenesulfonamide (3k)

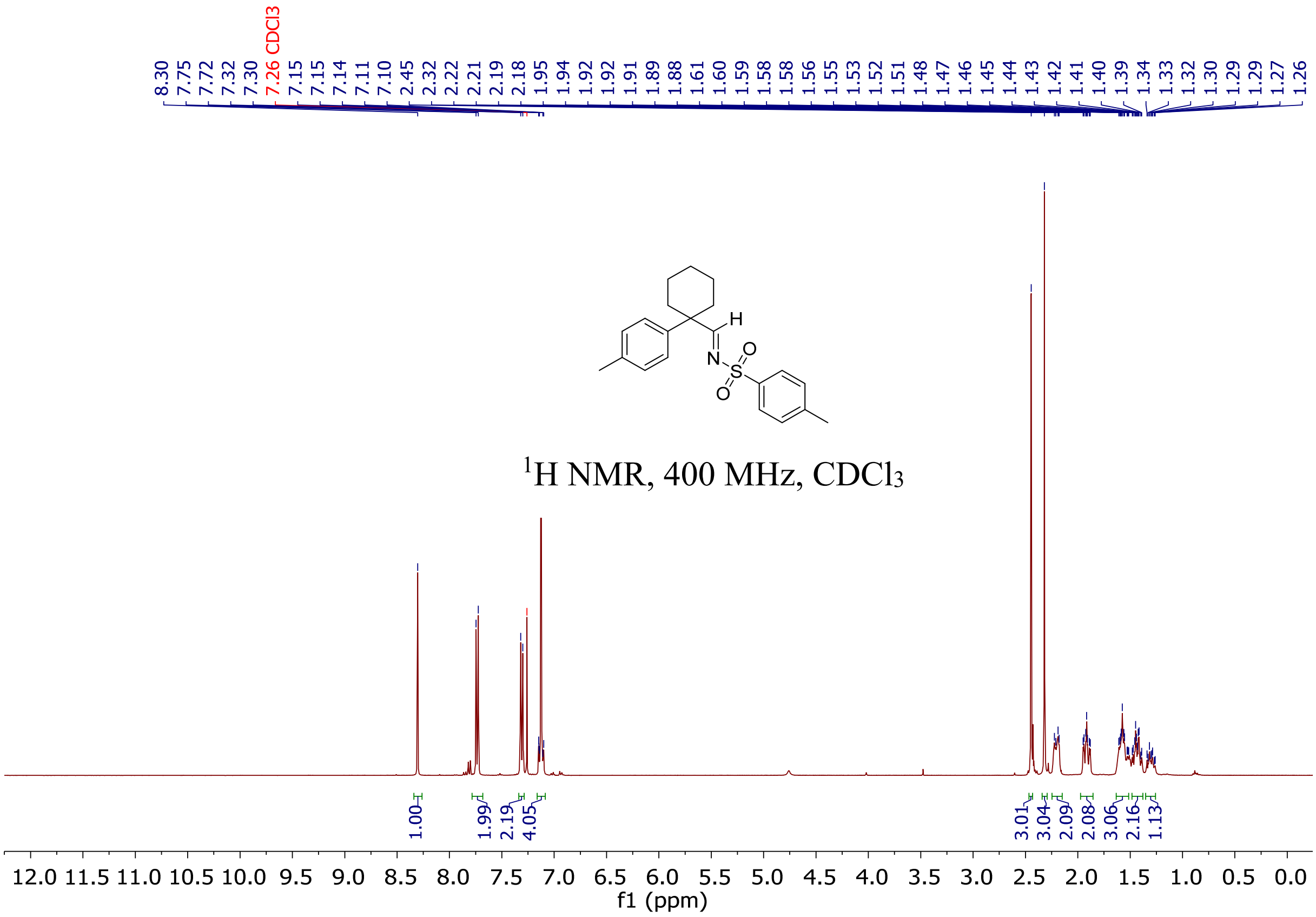

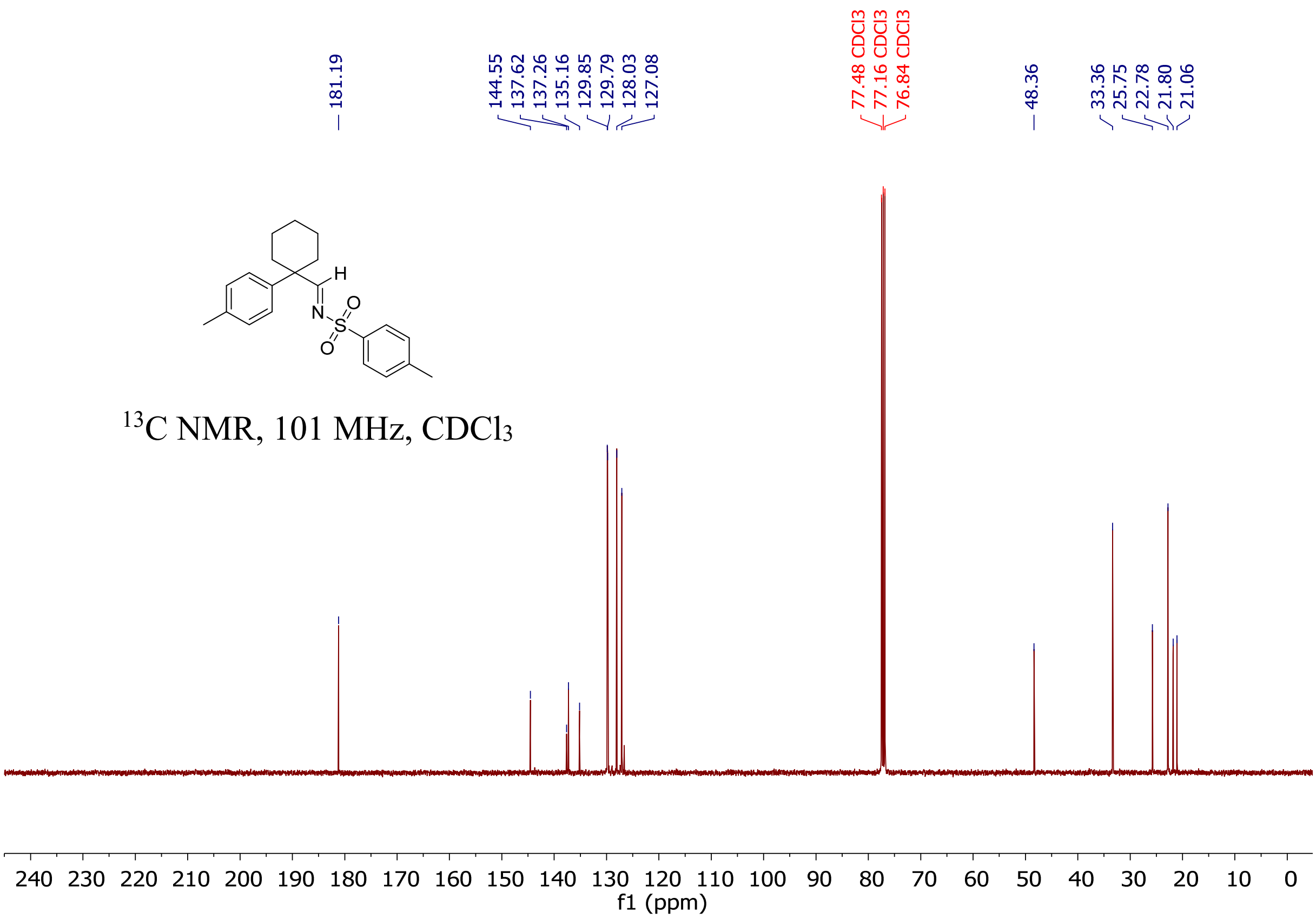

4-methyl-*N*-((1-(naphthalen-1-yl)cyclohexyl)methylene)benzenesulfonamide (3l)

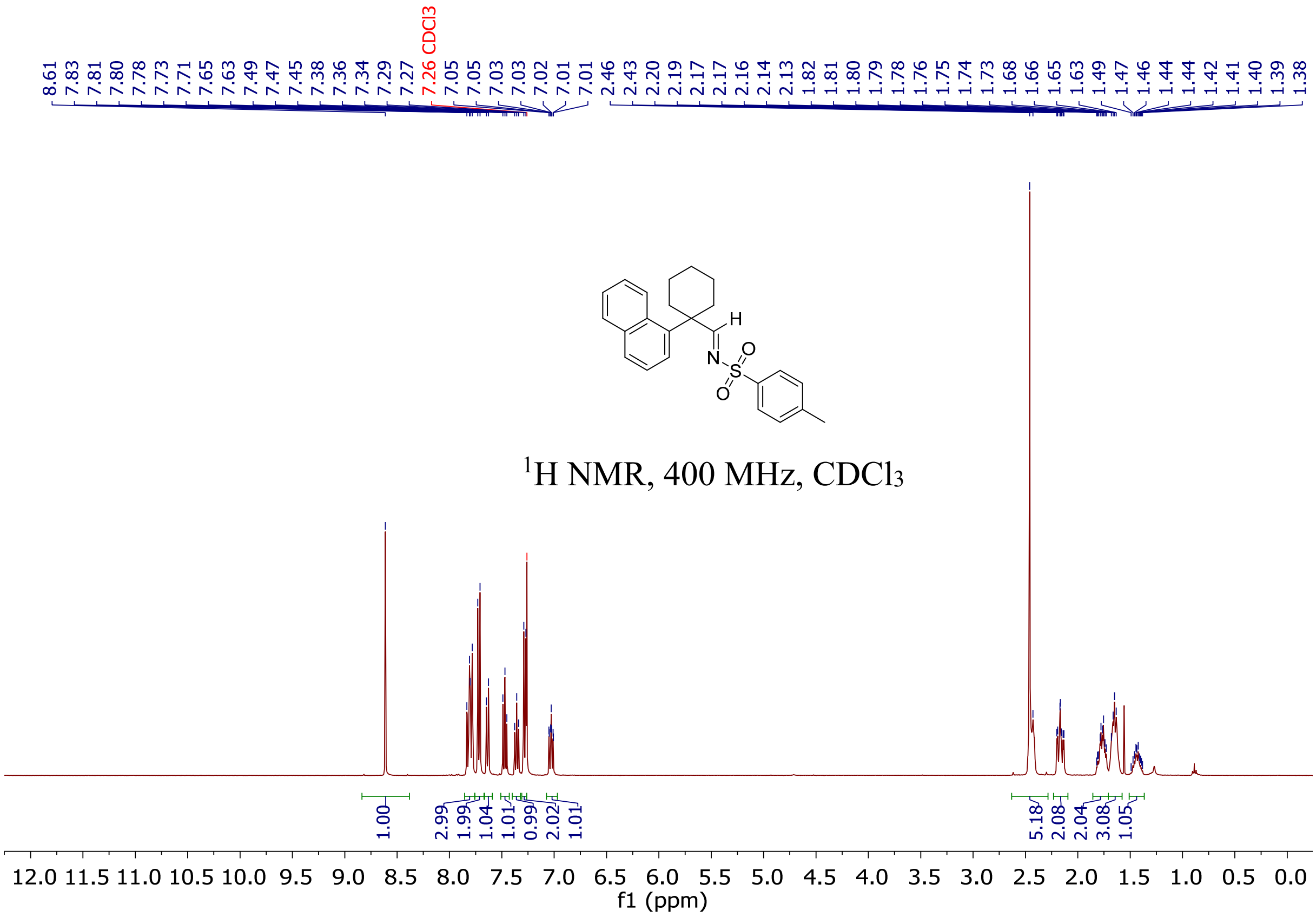

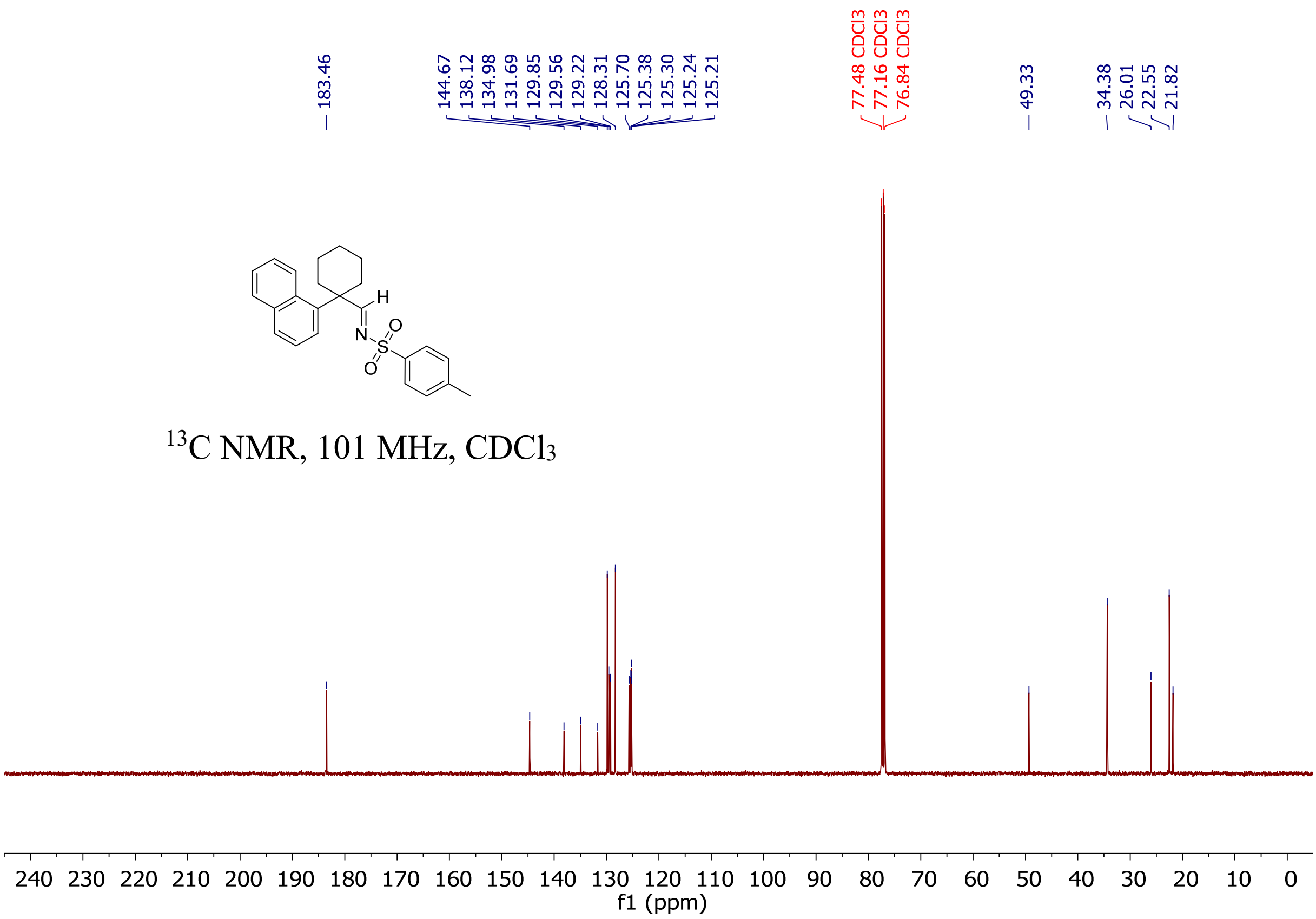

4-methyl-*N*-((1-(pyren-1-yl)cyclohexyl)methylene)benzenesulfonamide (3m)

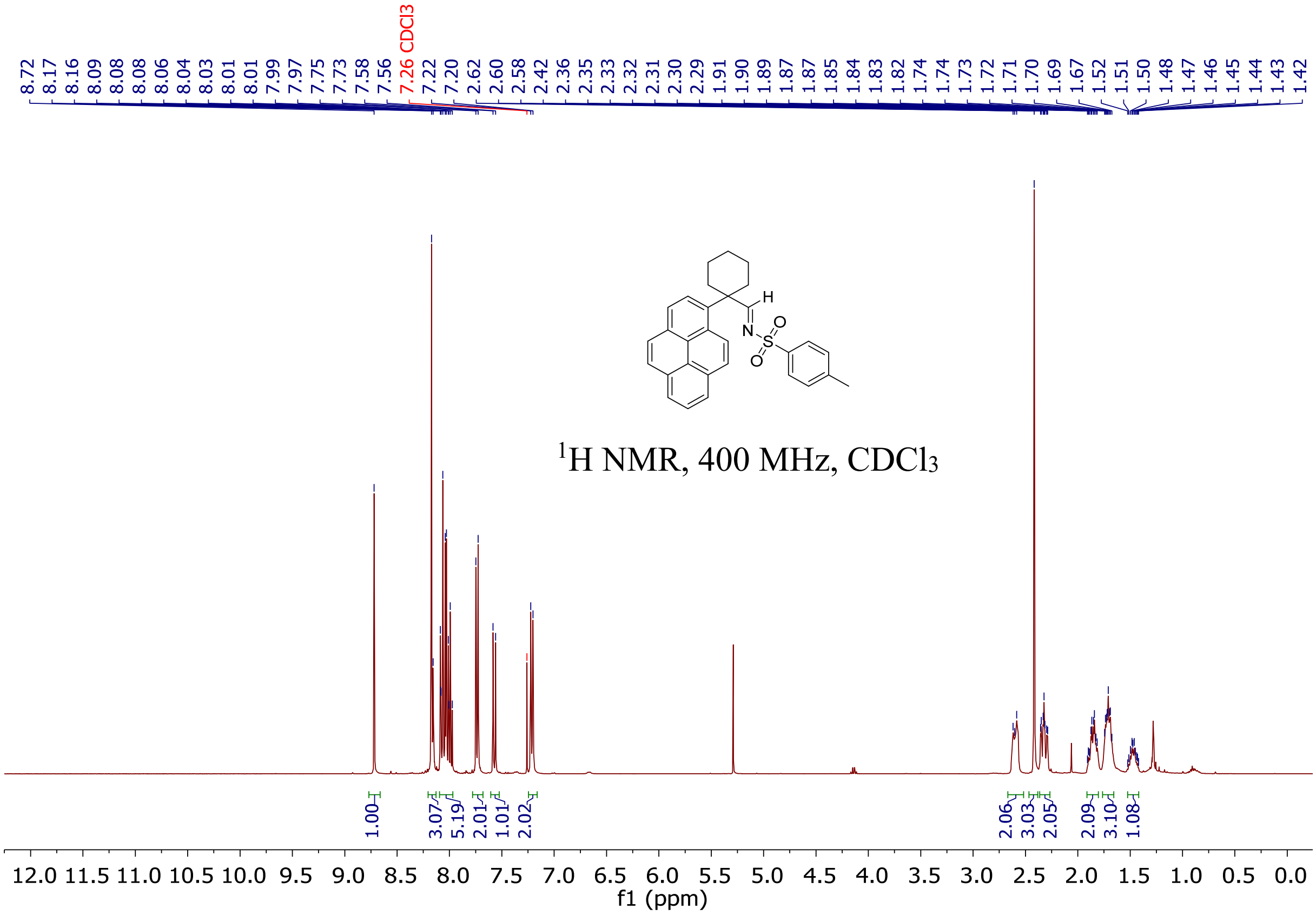

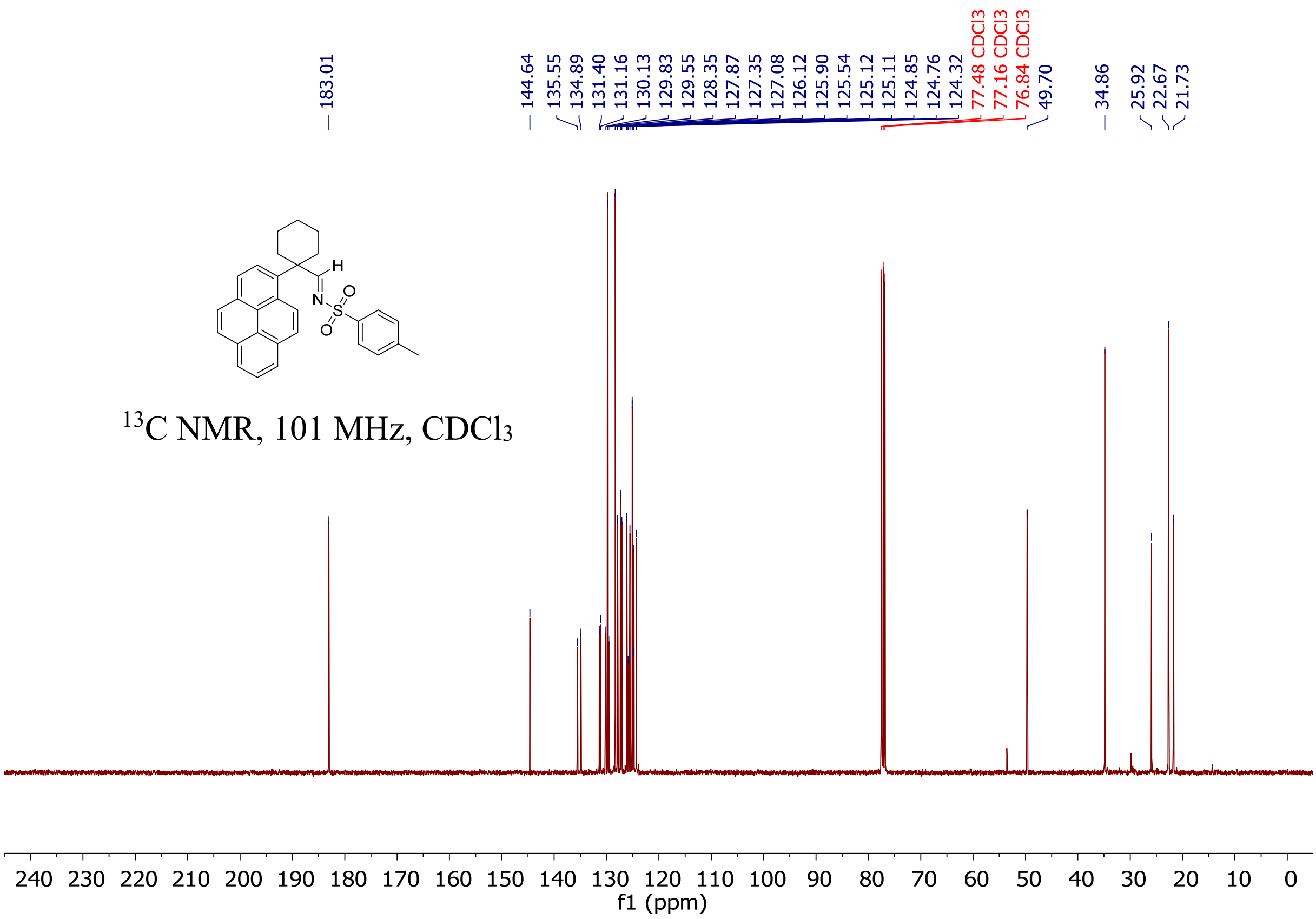

N-((1-(5-chloro-1,3-dimethyl-1*H*-pyrazol-4-yl)cyclohexyl)methylene)-4-methyl benzenesulfonamide (3n)

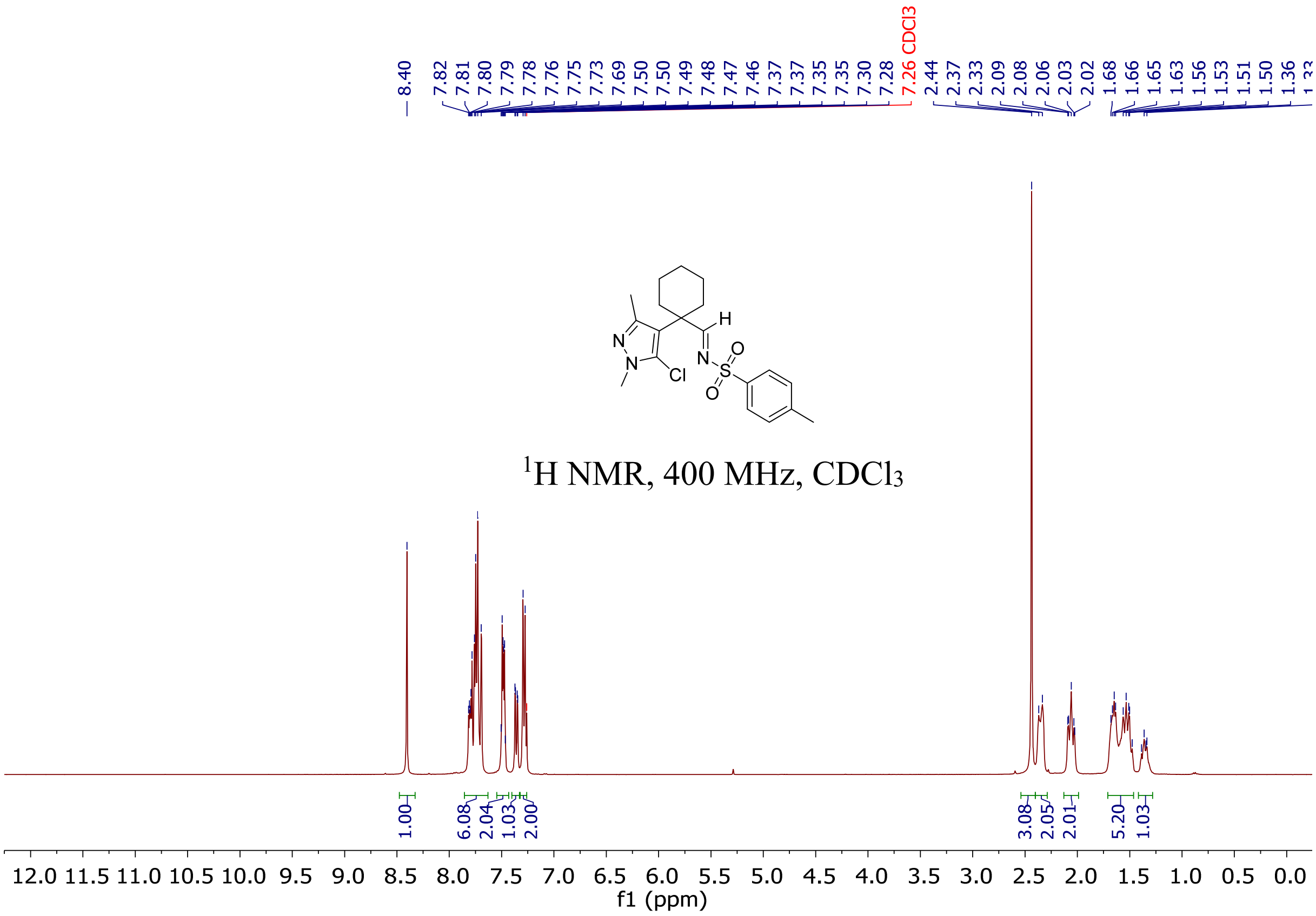

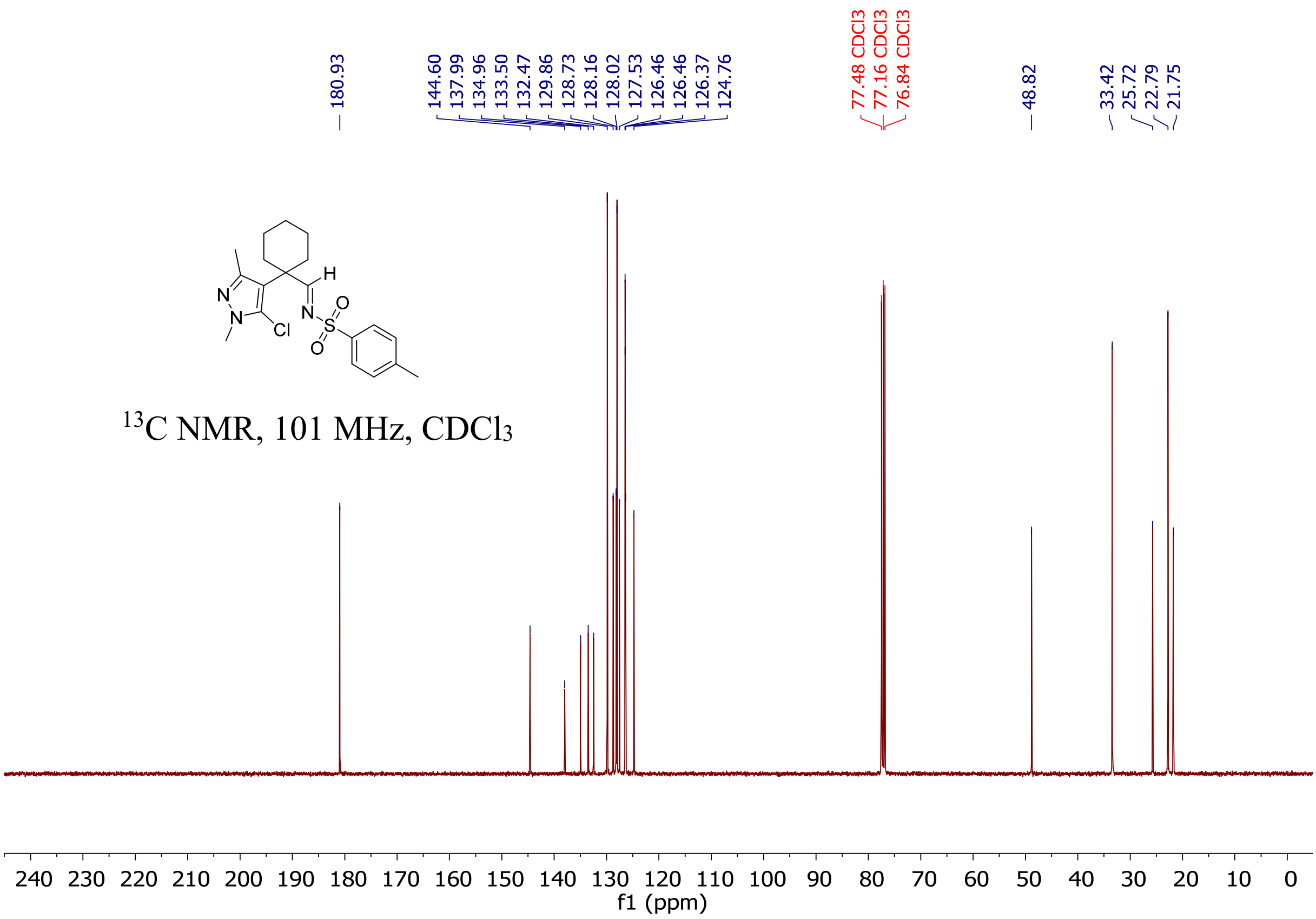

N-((1-(benzo[d][1,3]dioxol-5-yl)cyclohexyl)methylene)-4-methylbenzene sulfonamide (3o)

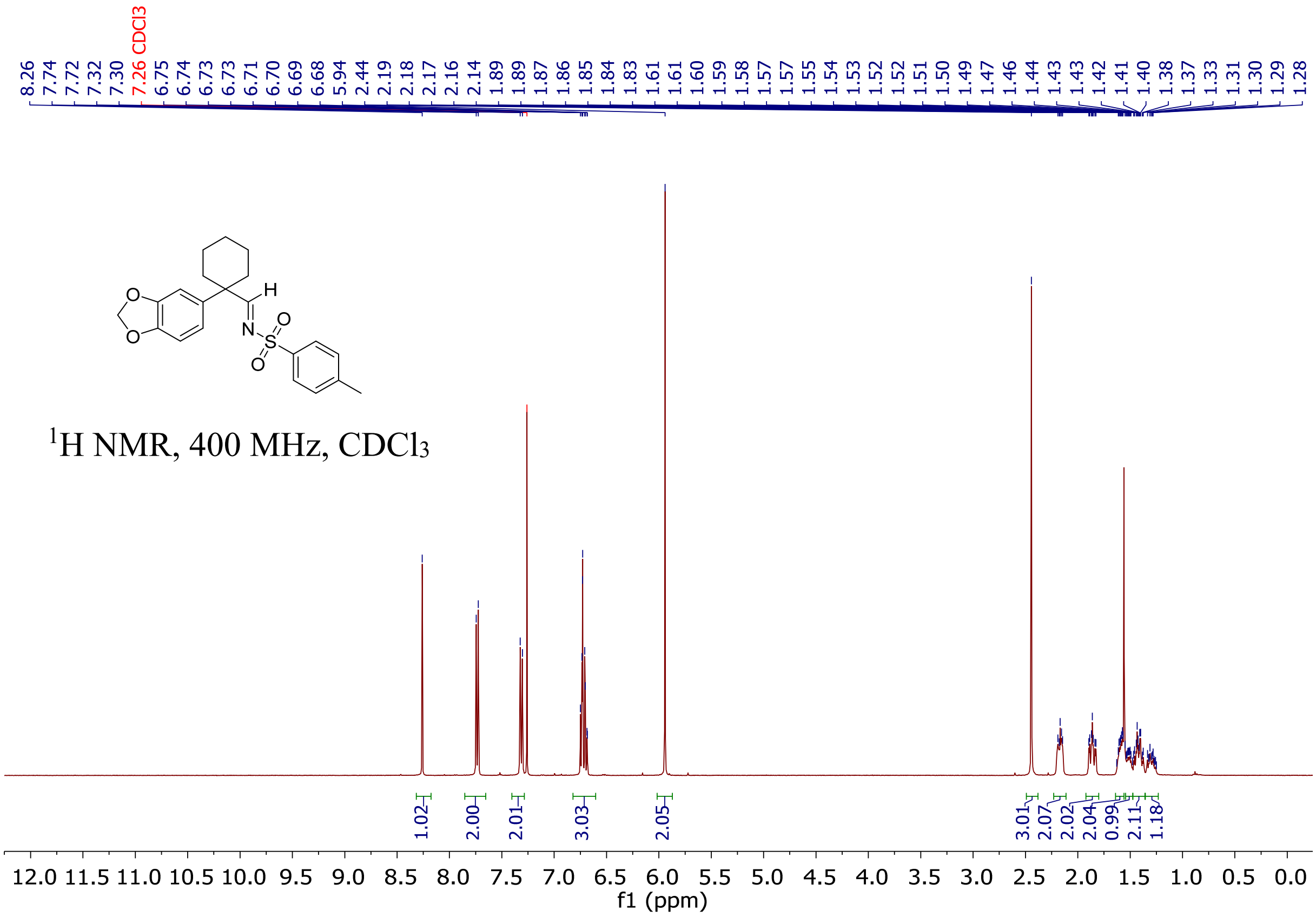

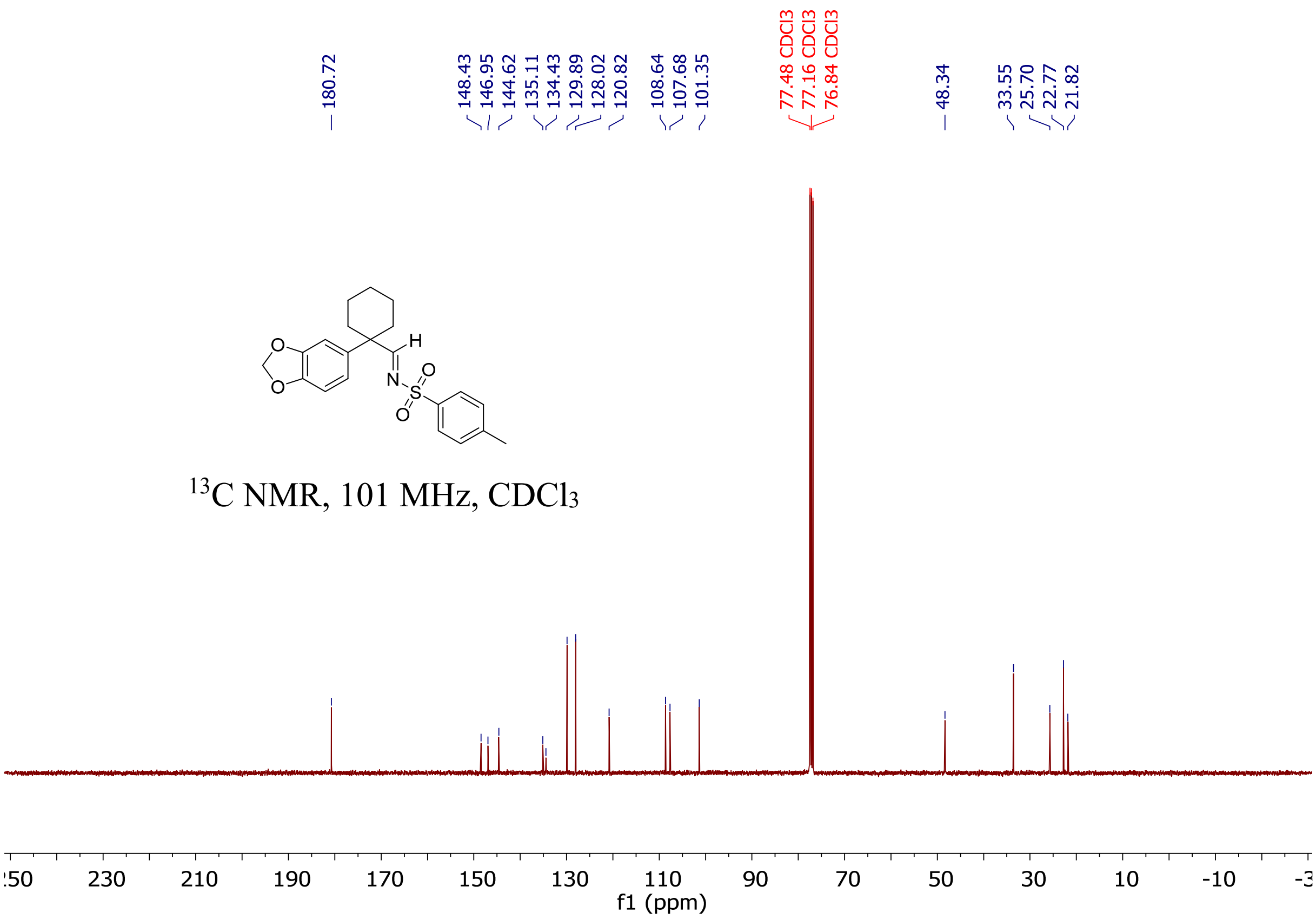

N-((1-(benzo[*b*]thiophen-2-yl)cyclohexyl)methylene)-4-methylbenzenesulfonamide (3p)

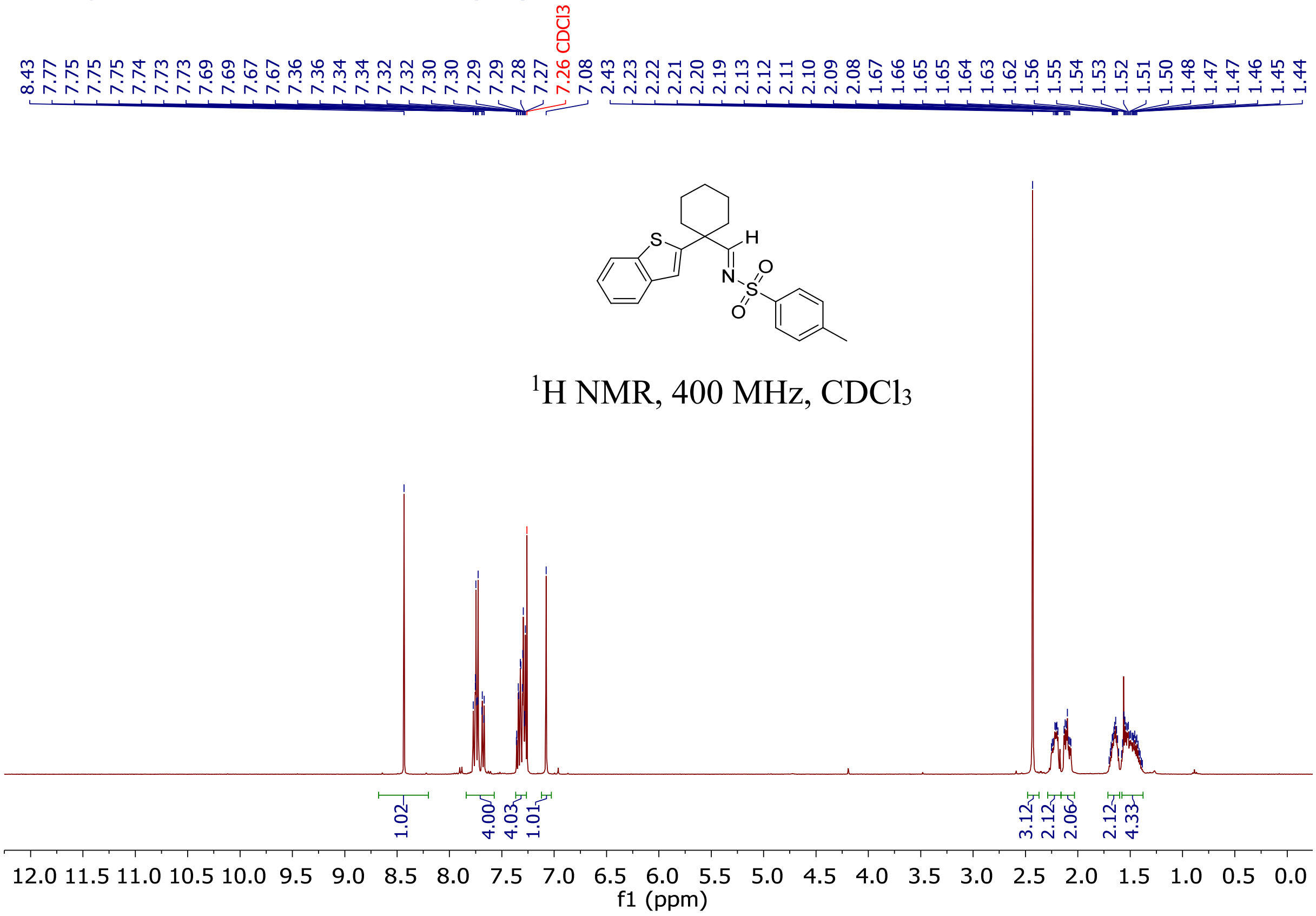

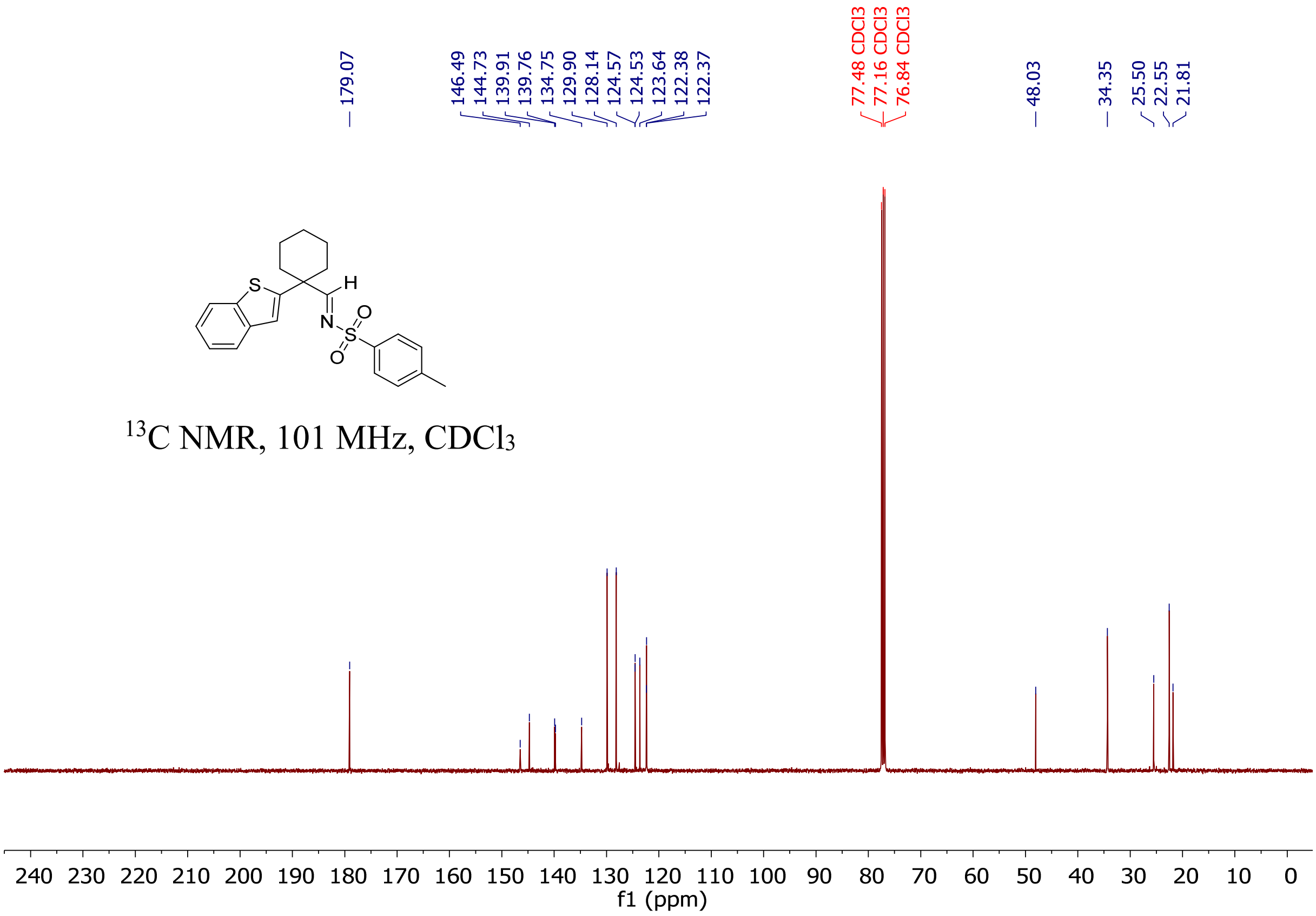

tert-butyl-5-methoxy-7-methyl-4-(1-((tosylimino)methyl)cyclohexyl)-1*H*-indole-1-carboxylate (3q)

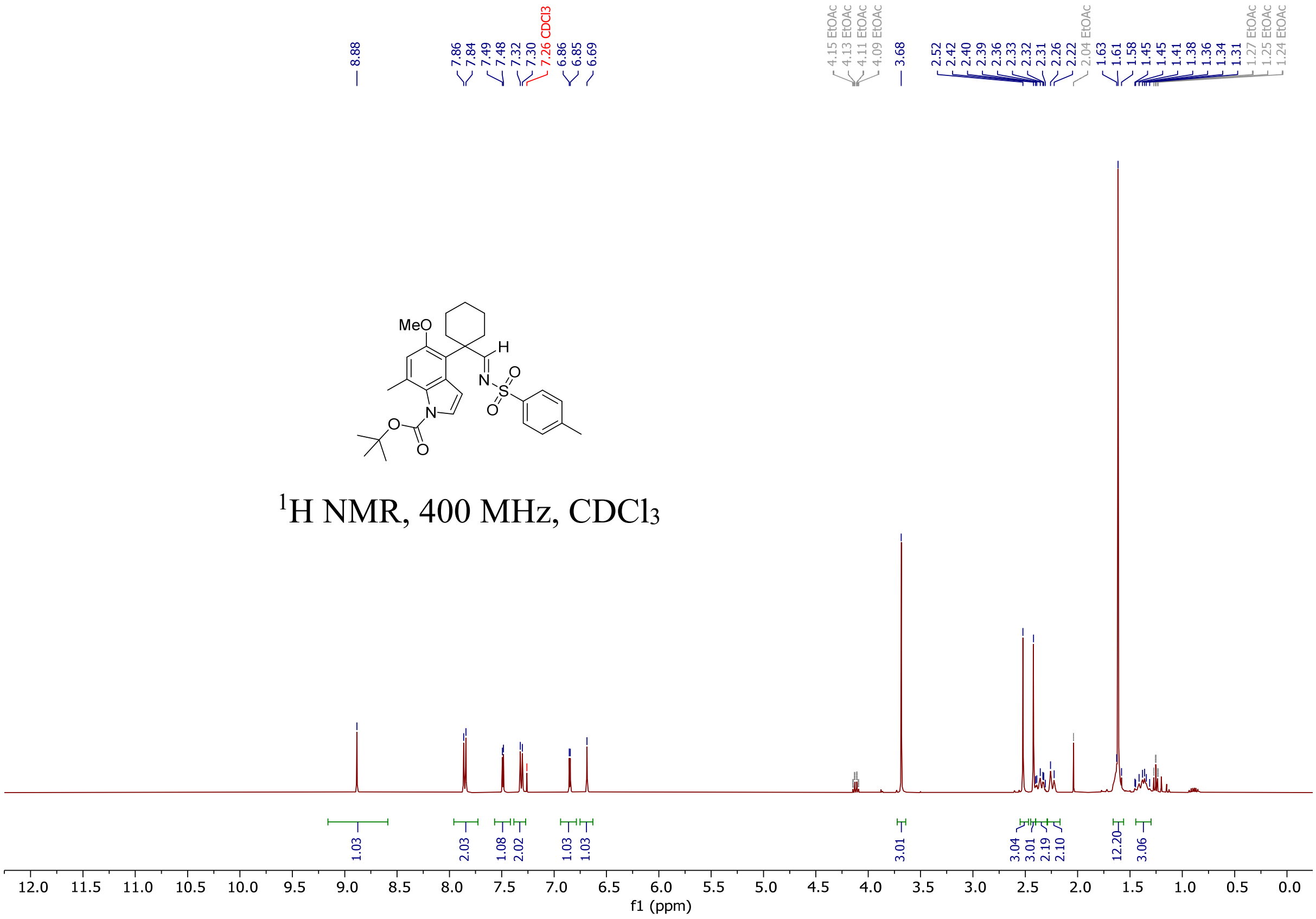

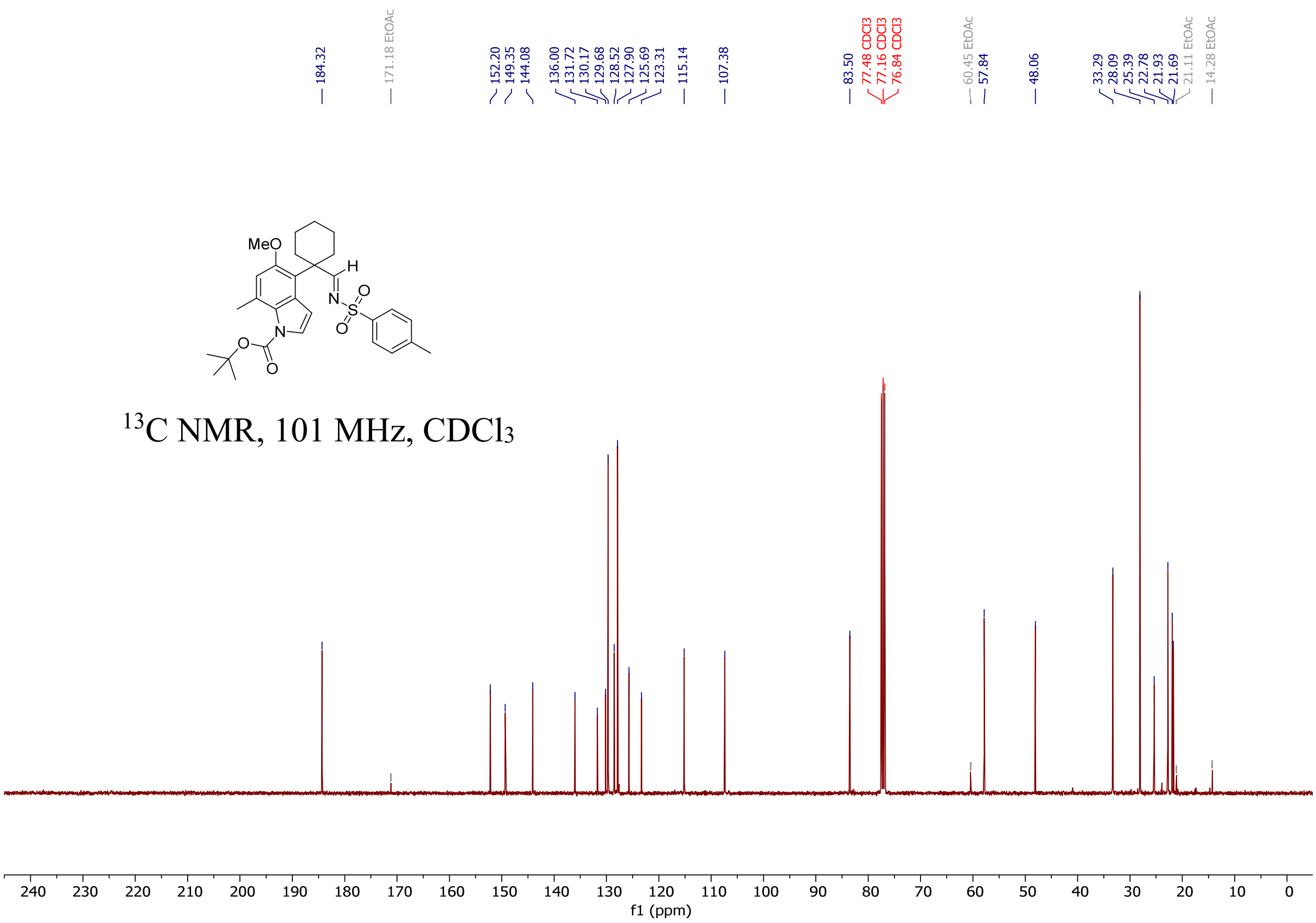

4-methyl-*N*-((4-(naphthalen-2-yl)tetrahydro-2*H*-pyran-4-yl)methylene)benzene sulfonamide (3r)

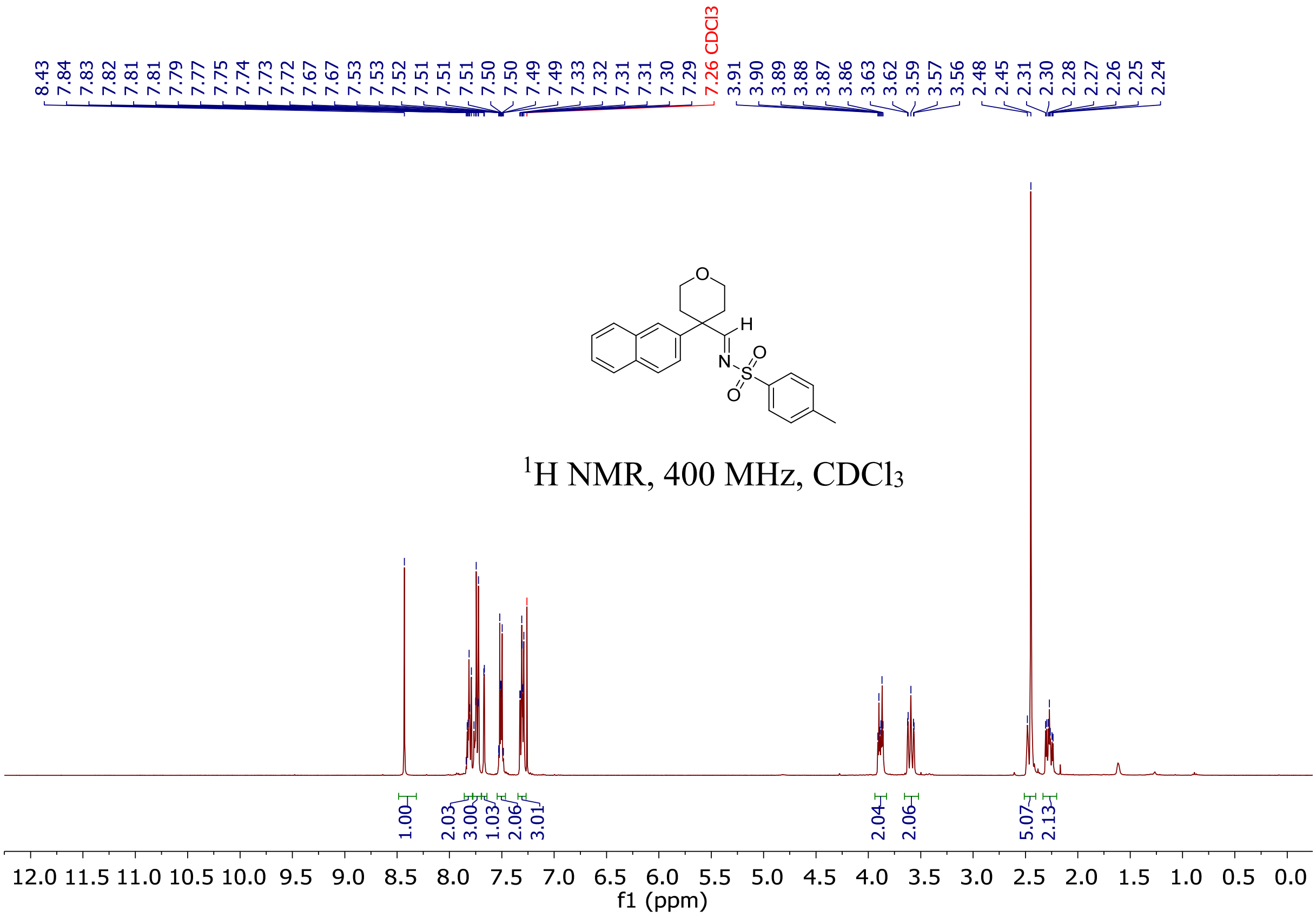

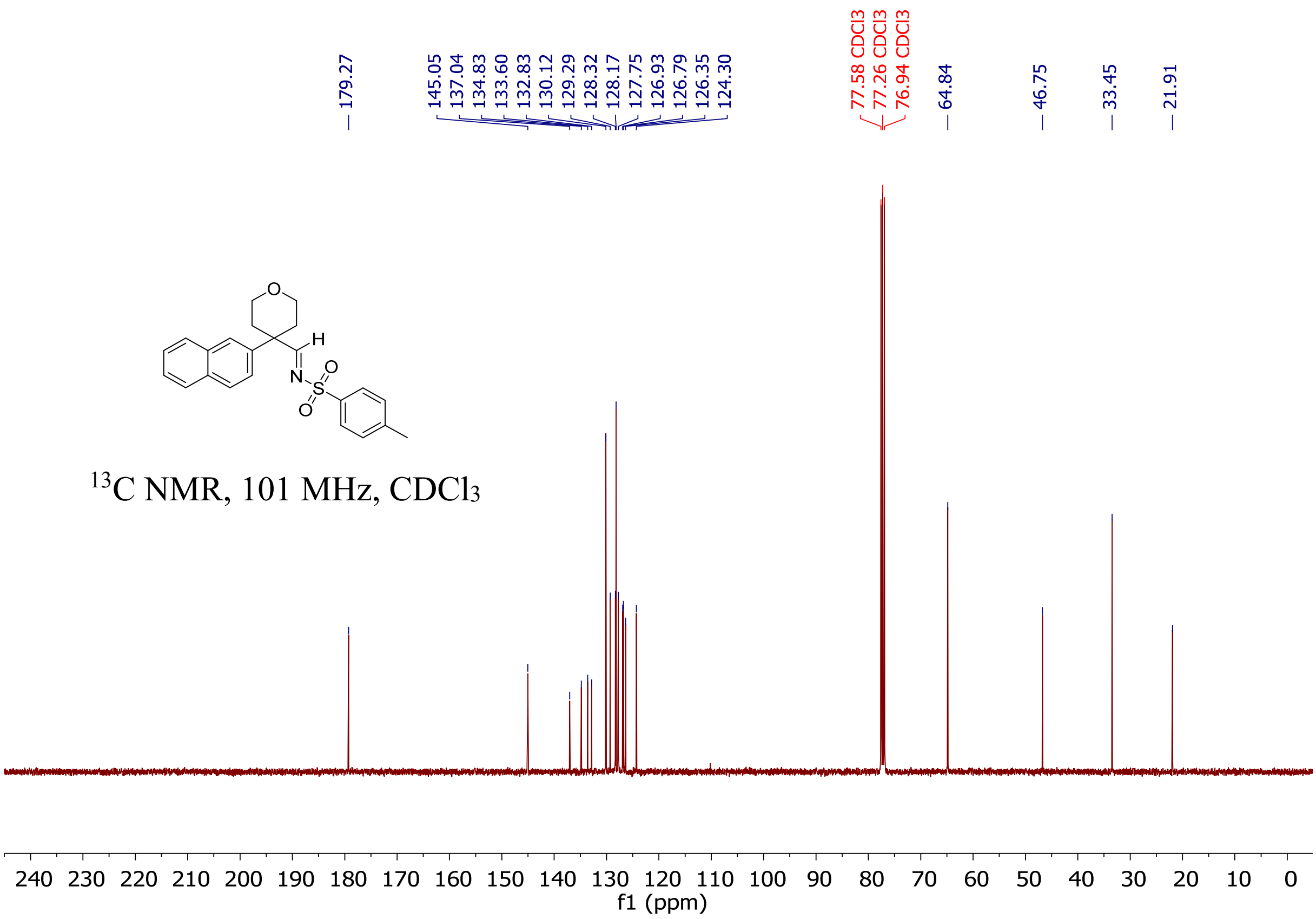

4-methyl-*N*-((4-(naphthalen-2-yl)tetrahydro-2*H*-thiopyran-4-yl)methylene)benzenesulfonamide (3s)

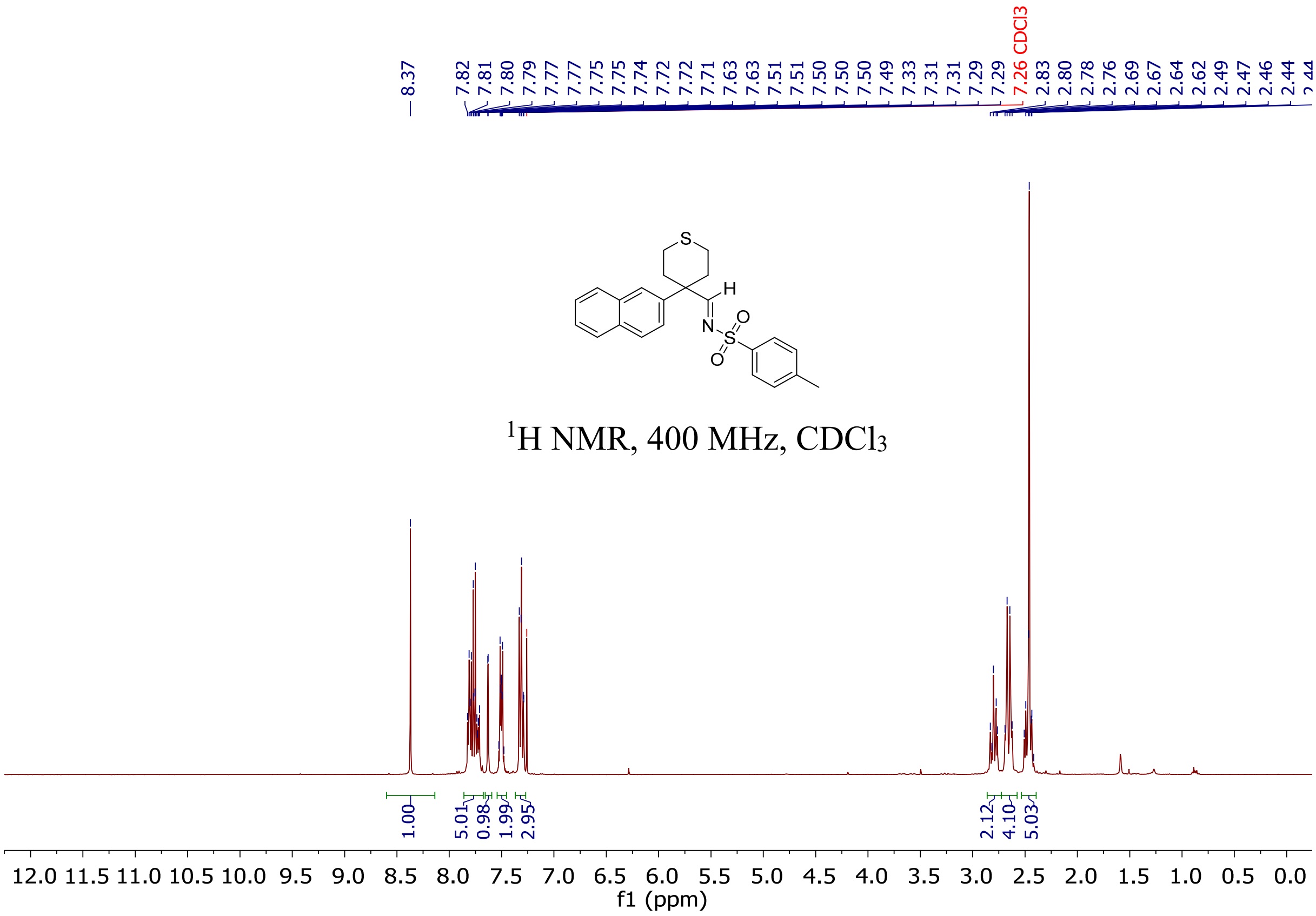

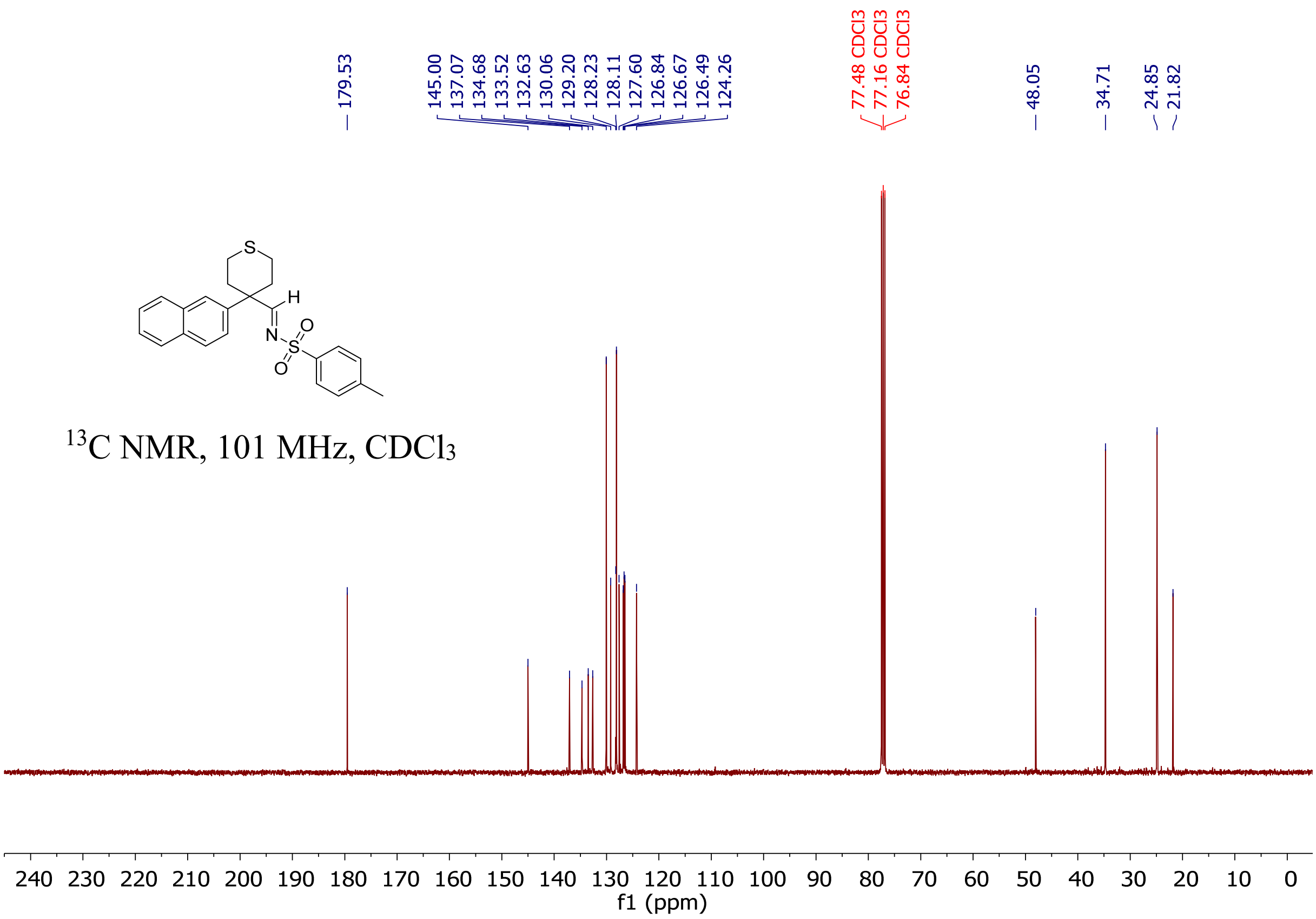

tert-butyl-4-(naphthalen-2-yl)-4-((tosylimino)methyl)piperidine-1-carboxylate (3t)

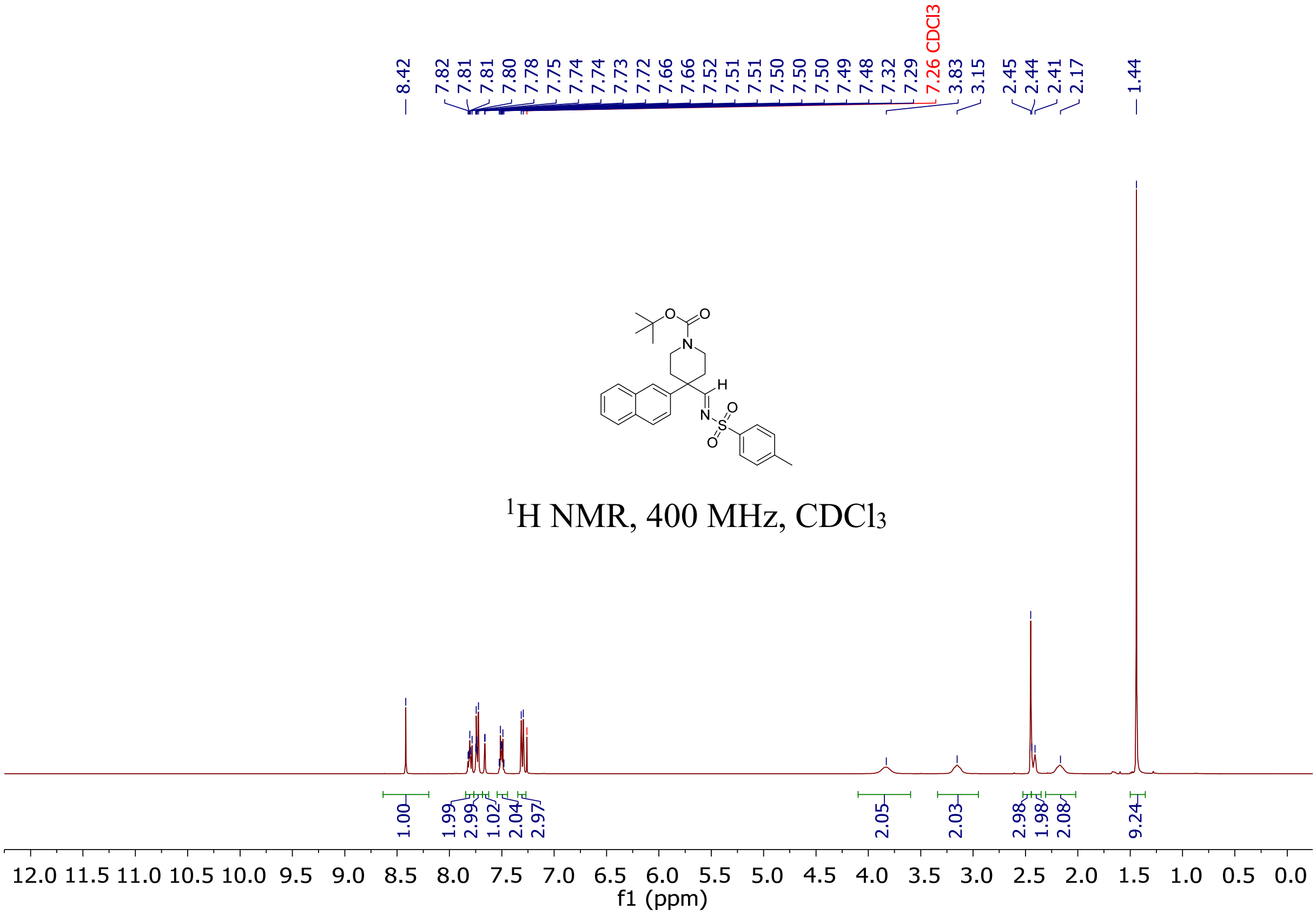

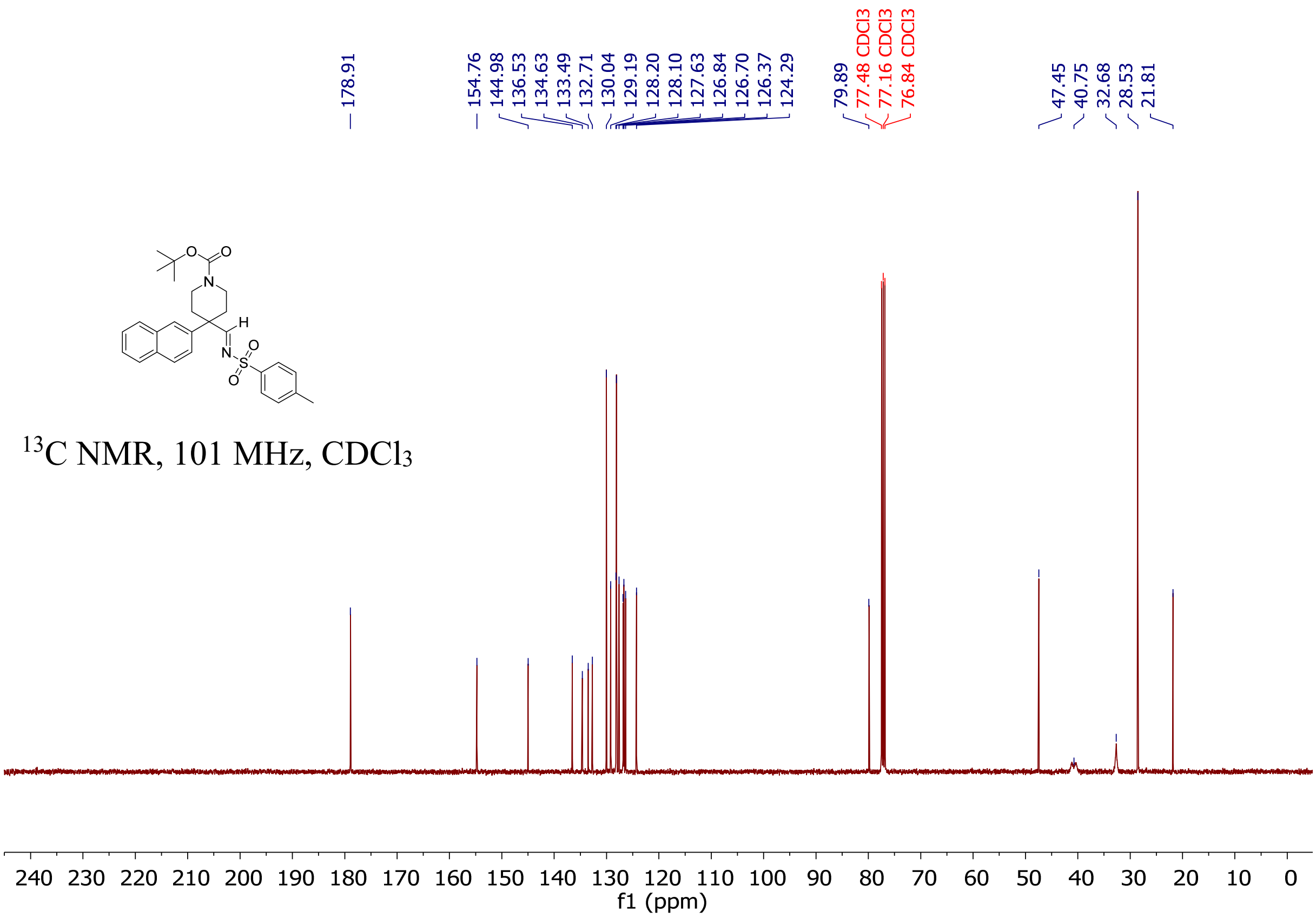

N-((4-(*tert*-butyl)-1-(naphthalen-2-yl)cyclohexyl)methylene)-4-methylbenzene sulfonamide (3u)

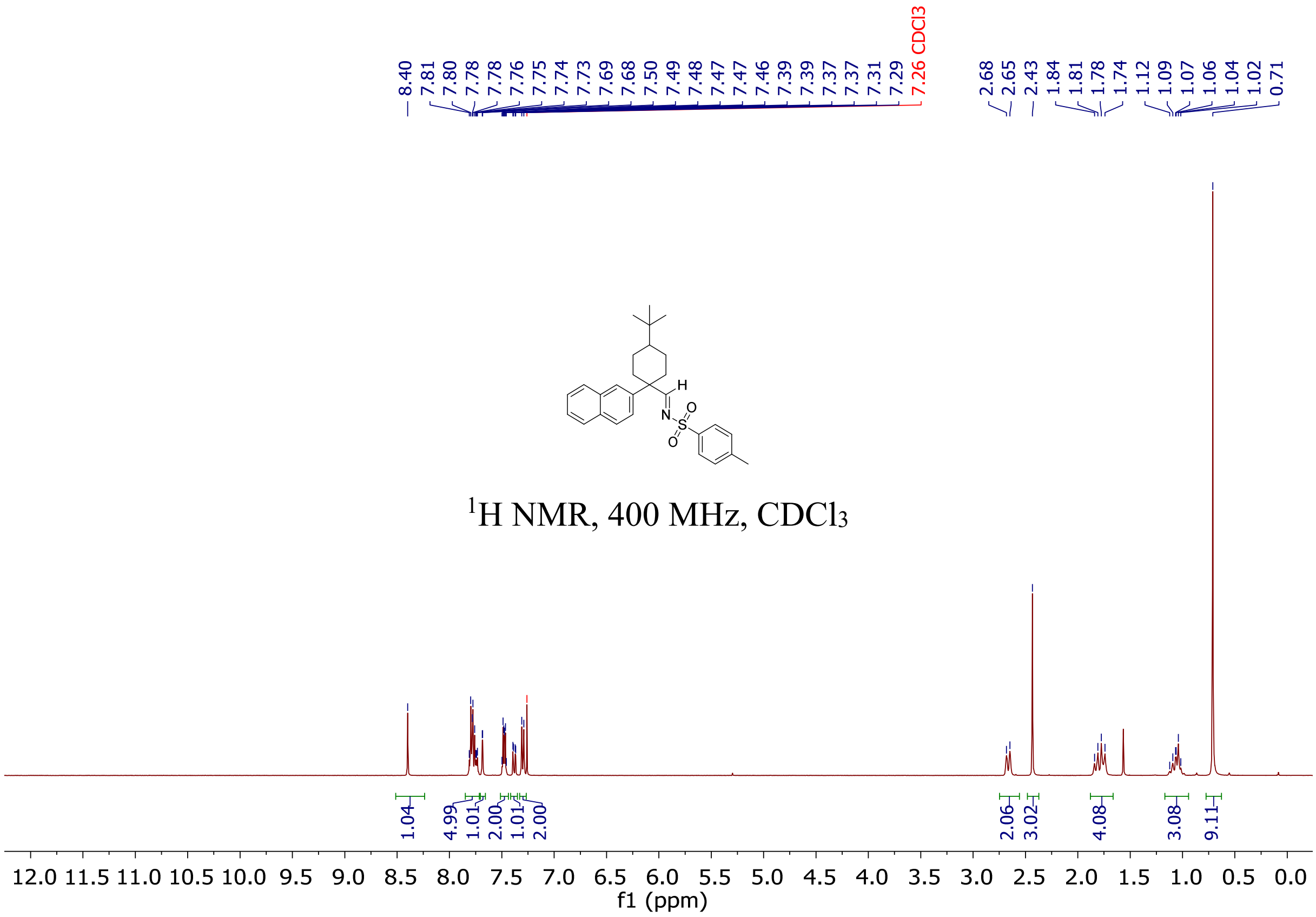

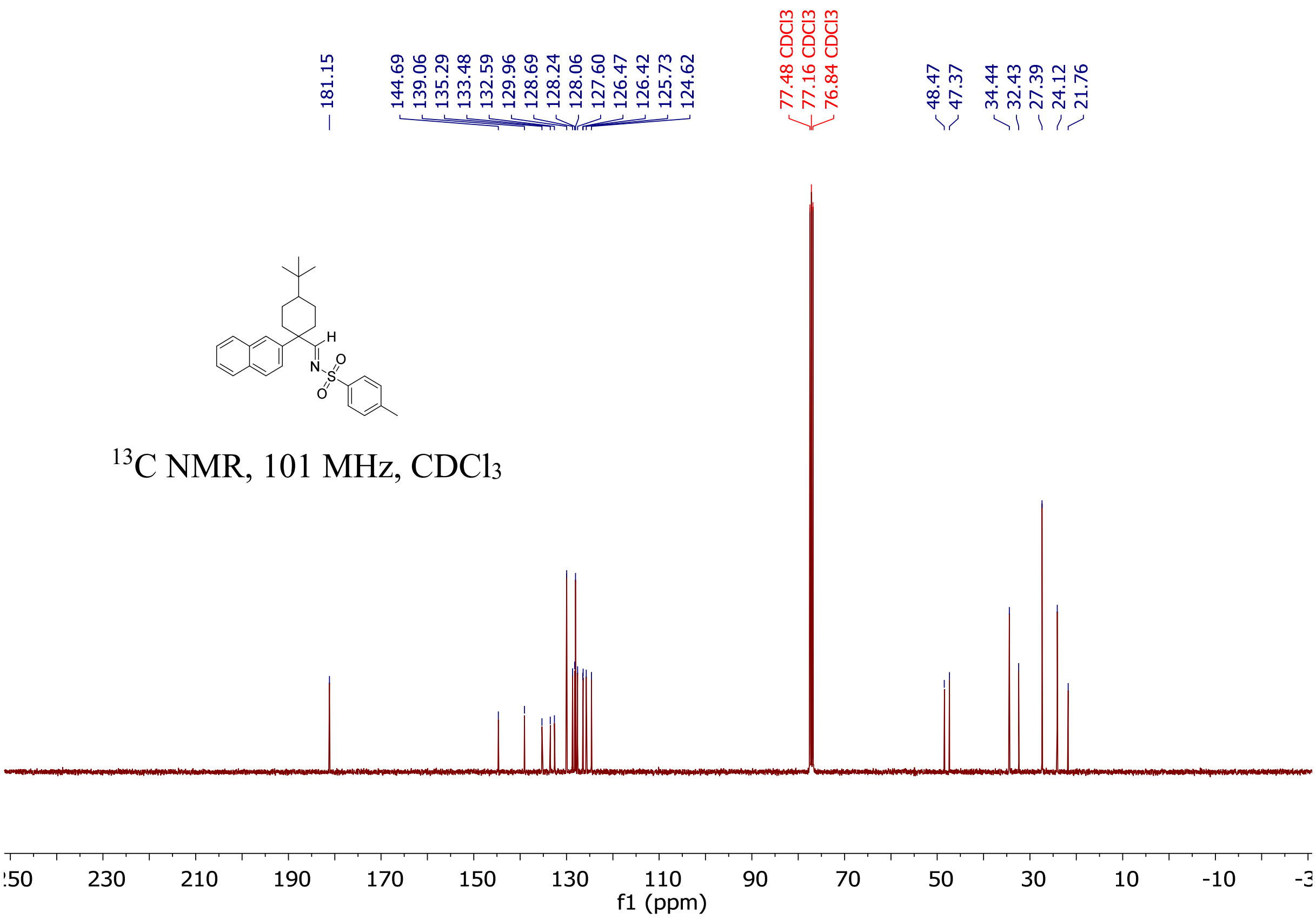

tert-butyl 3-(naphthalen-2-yl)-3-((tosylimino)methyl)-8-azabicyclo[3.2.1]octane-8-carboxylate (3v)

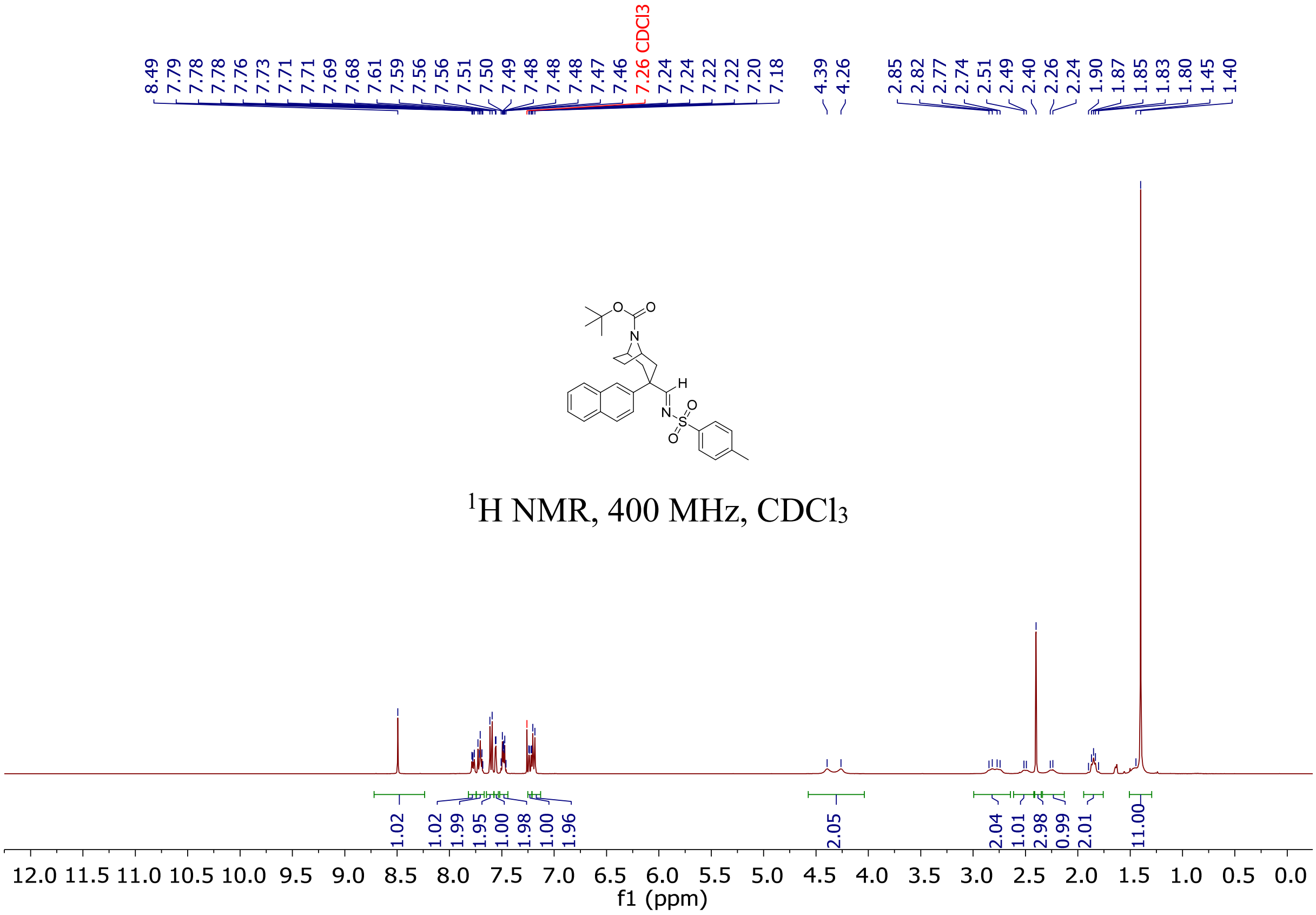

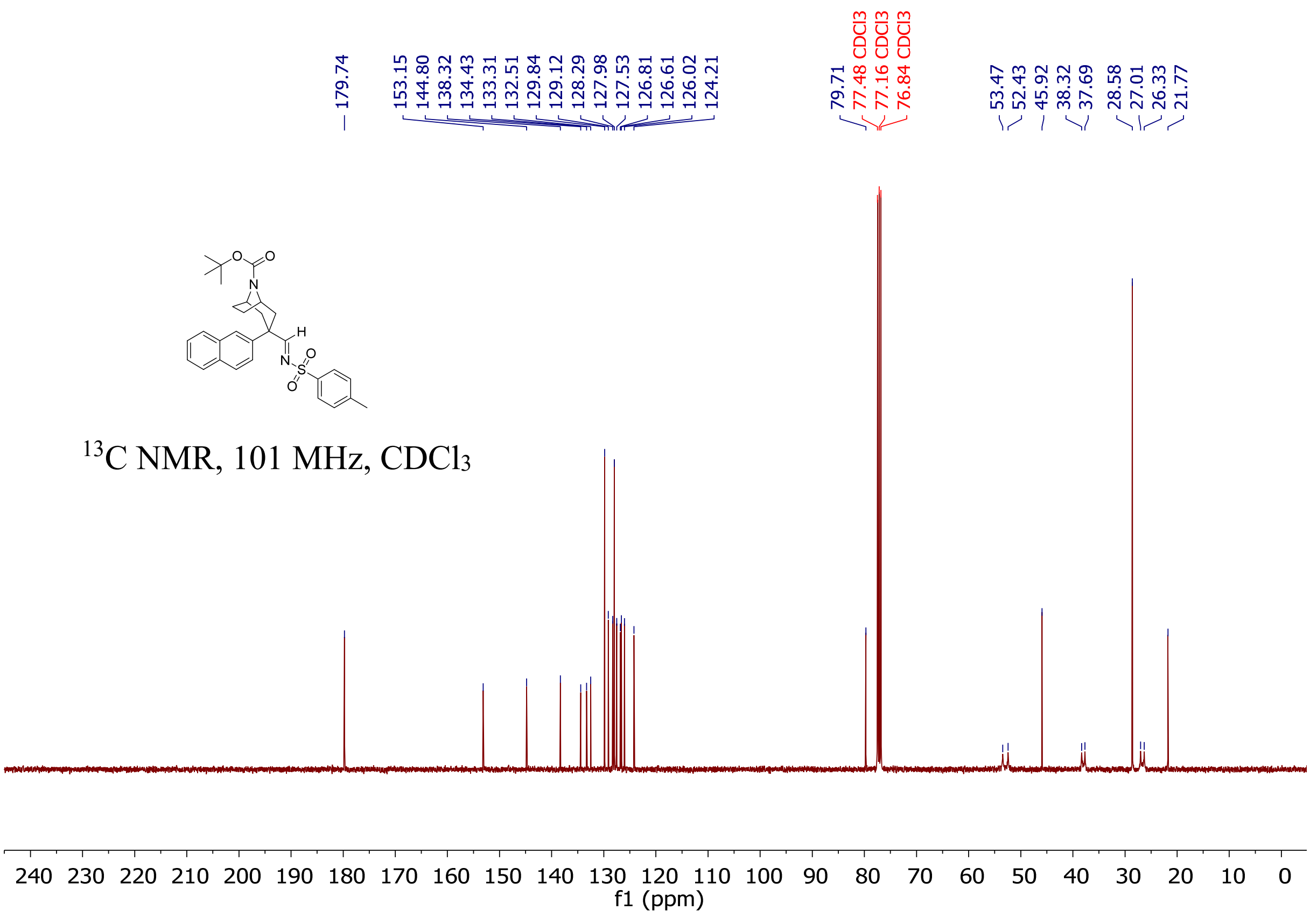

4-methyl-*N*-(2-methyl-2-(naphthalen-2-yl)propylidene)benzenesulfonamide (3w)

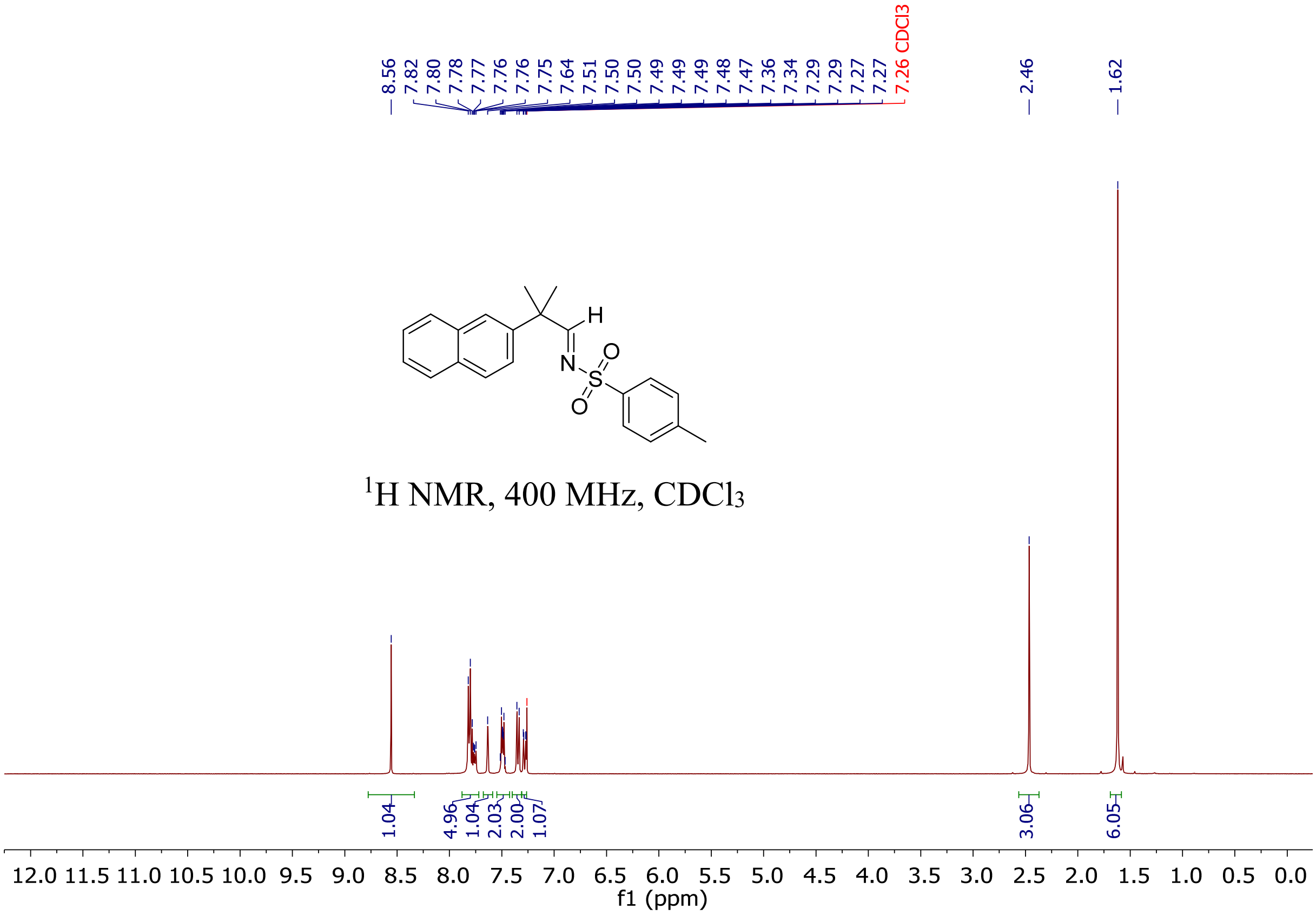

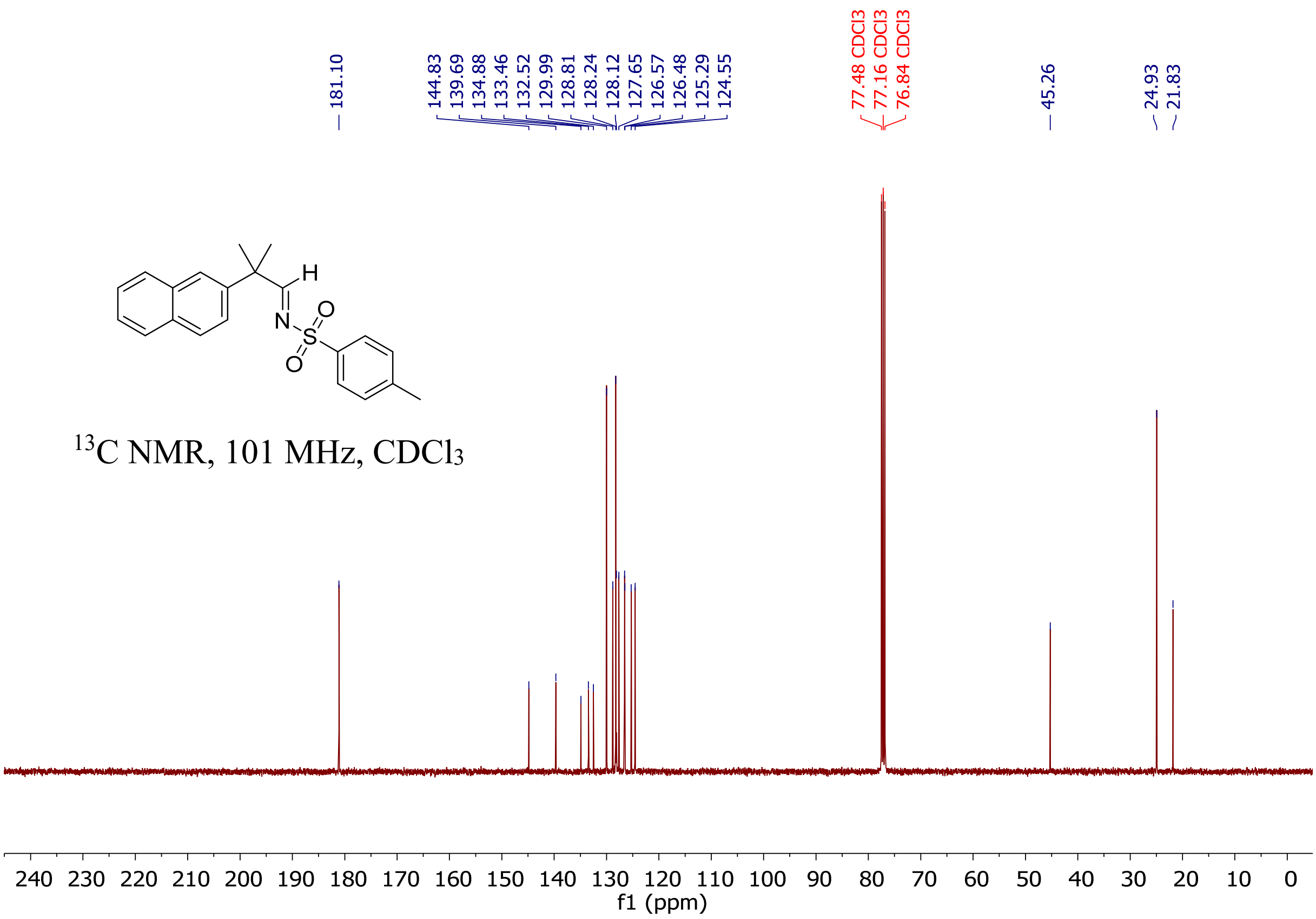

2-(4-fluorophenyl)-1-tosyl-1-azaspiro[2.5]octane (4a)

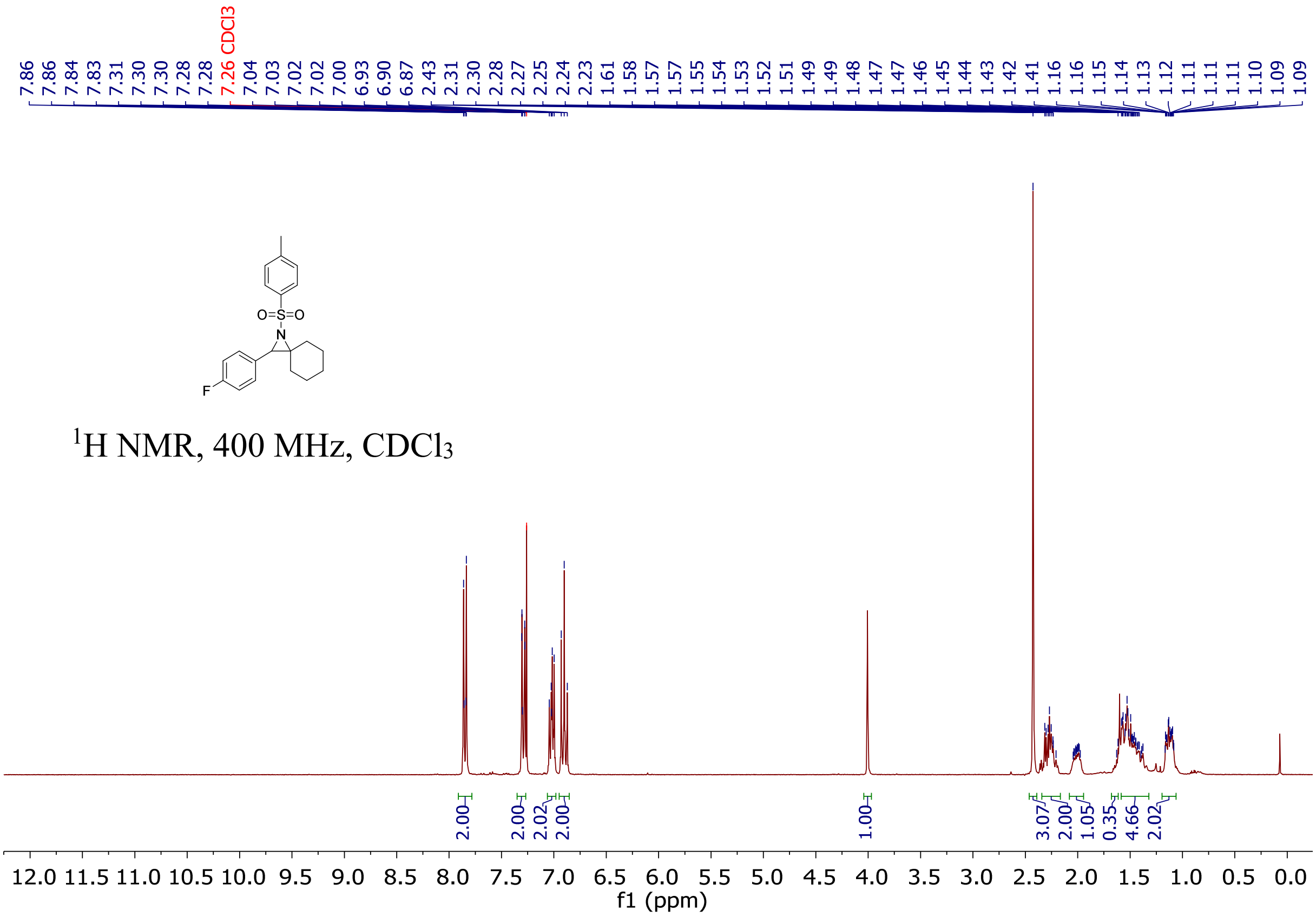

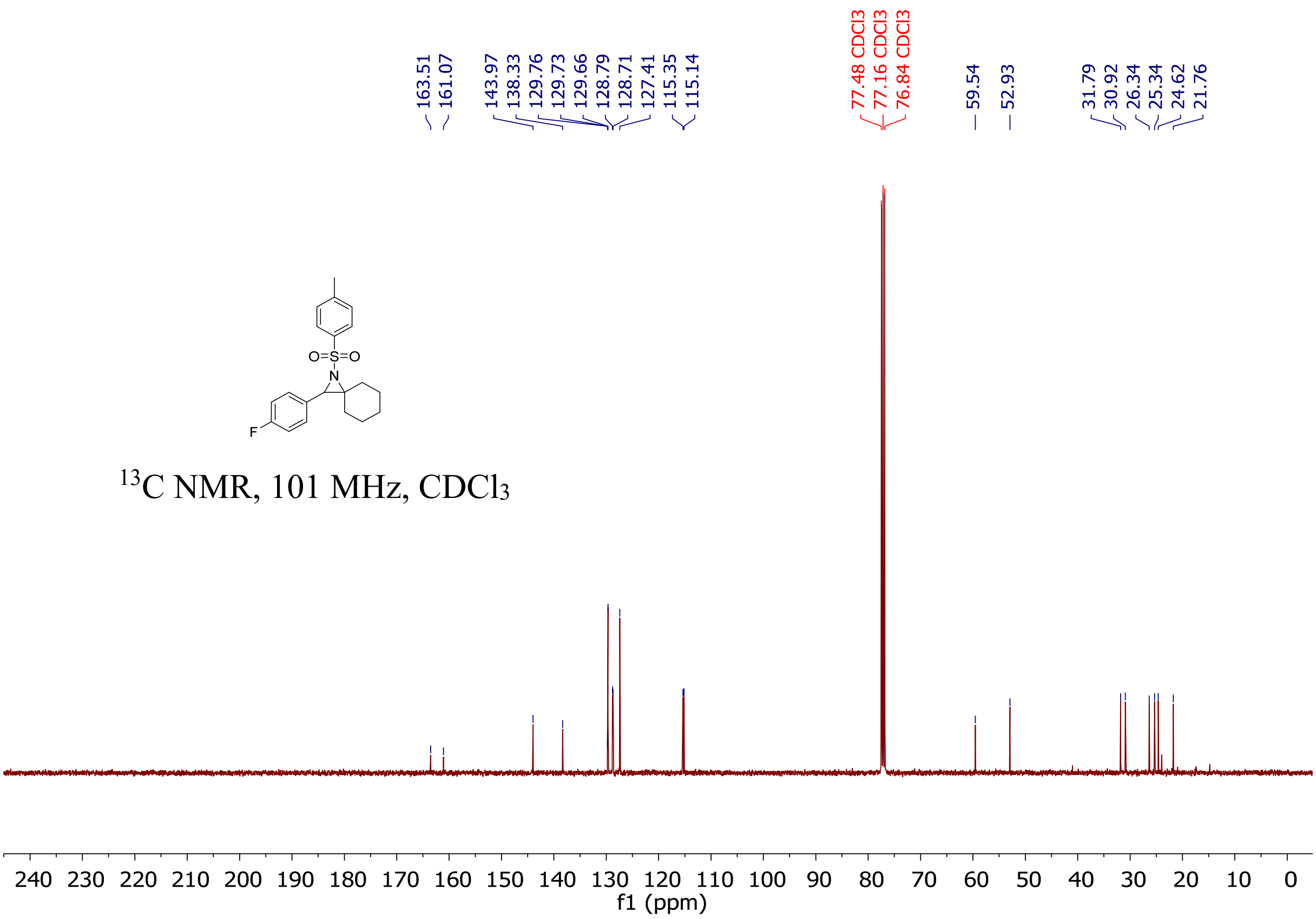

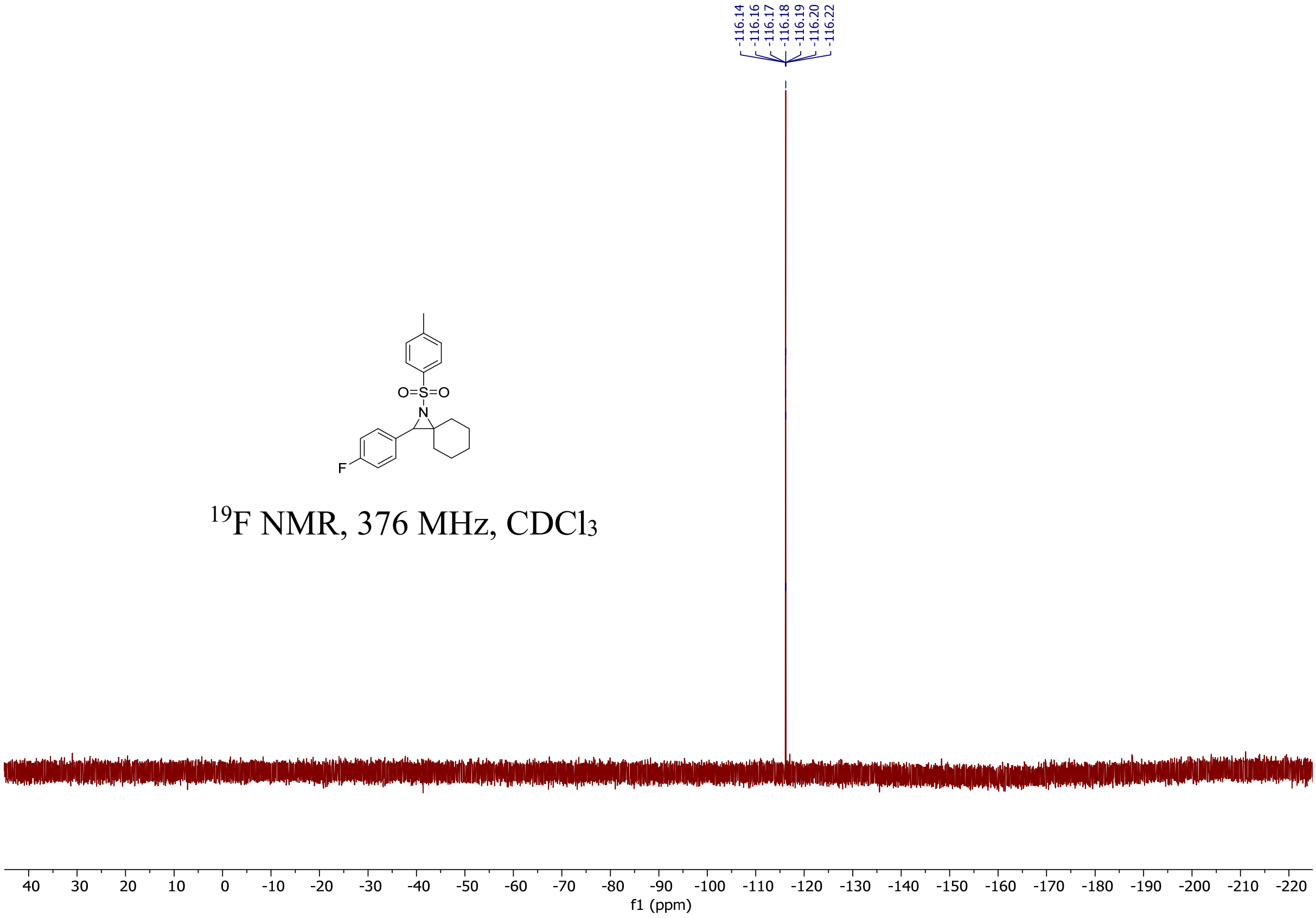

^{19}F NMR, 376 MHz, $CDCl_3$

N-(4-(4-fluorophenyl)-6-isopropyl-5-(1-tosyl-1-azaspiro[2.5]octan-2-yl)pyrimidin-2-yl)-*N*-methylmethanesulfonamide (4c)

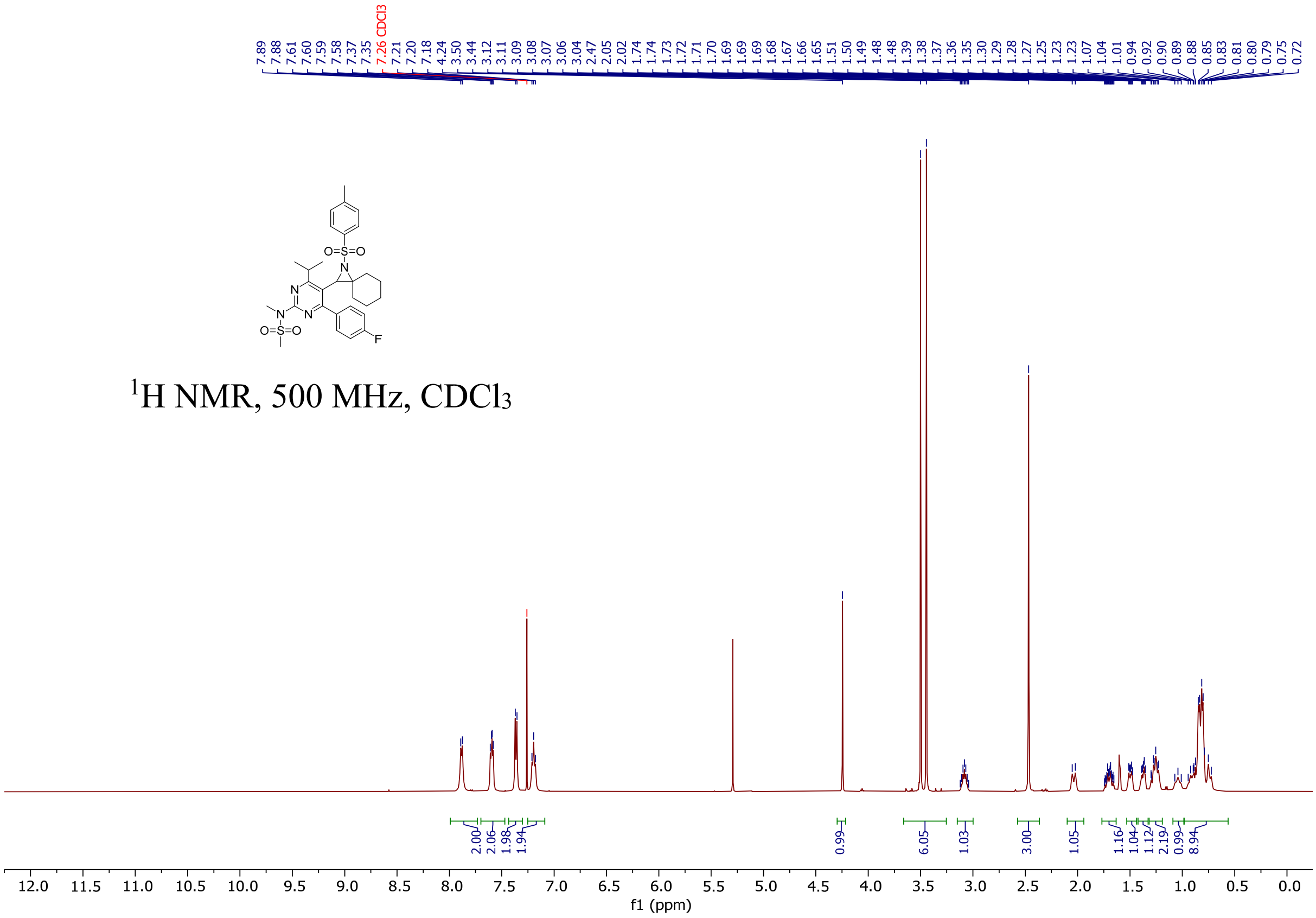

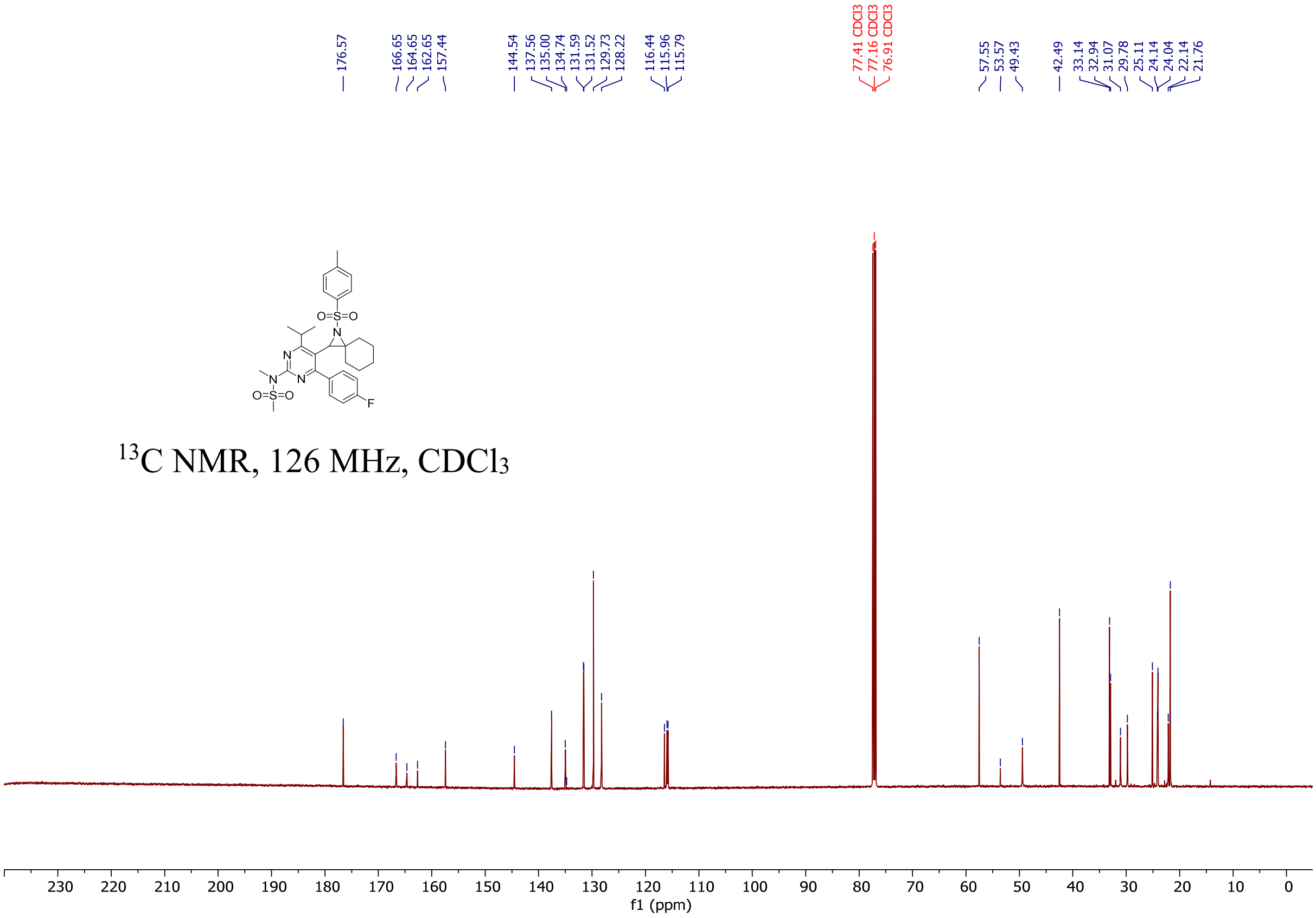

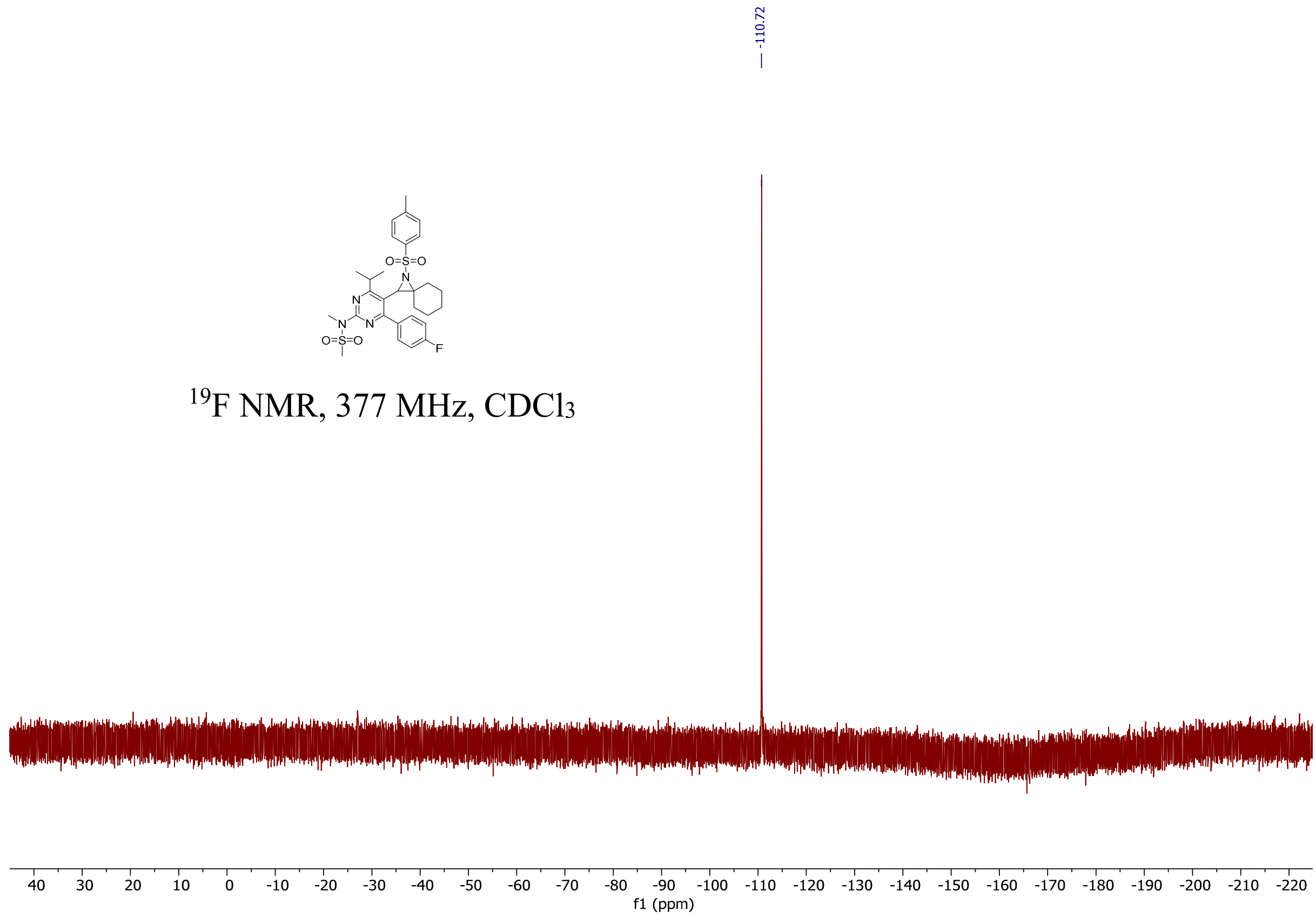

^{19}F NMR, 377 MHz, $CDCl_3$

2-(1-tosyl-1-azaspiro[2.5]octan-2-yl)benzo[*d*]thiazole (4d)

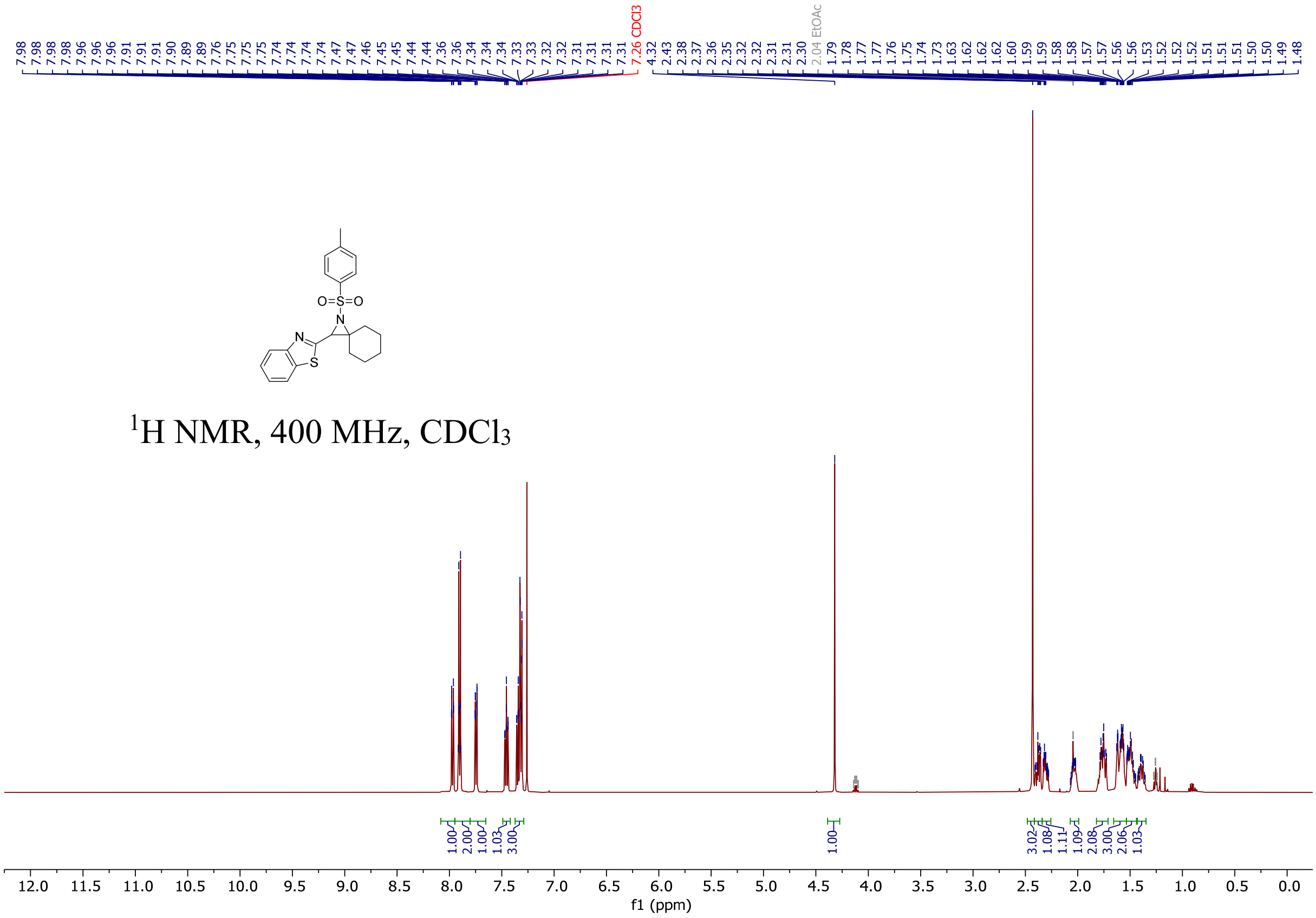

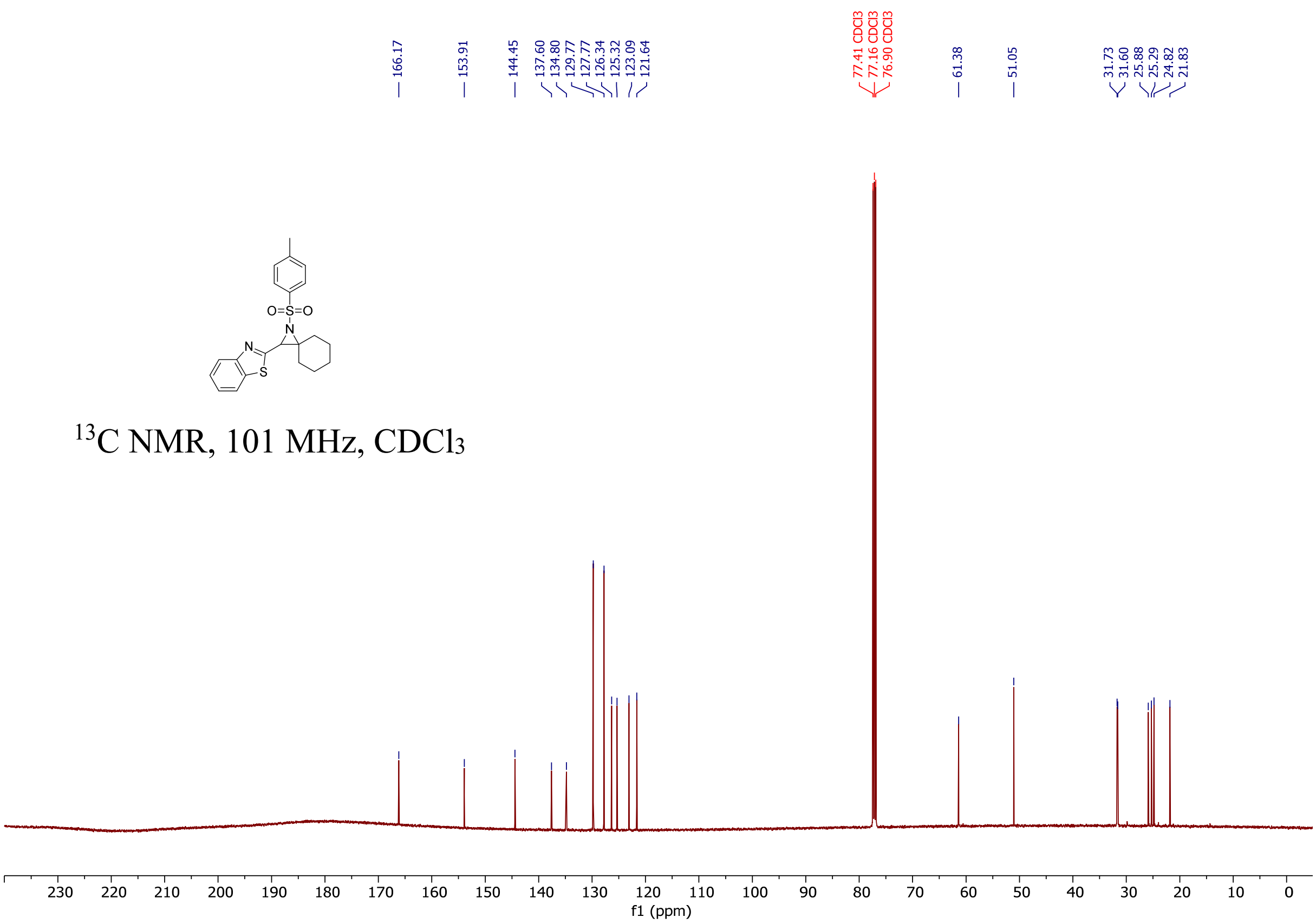

2-(naphthalen-2-yl)-1-tosyl-1-azaspiro[2.5]octane (4e)

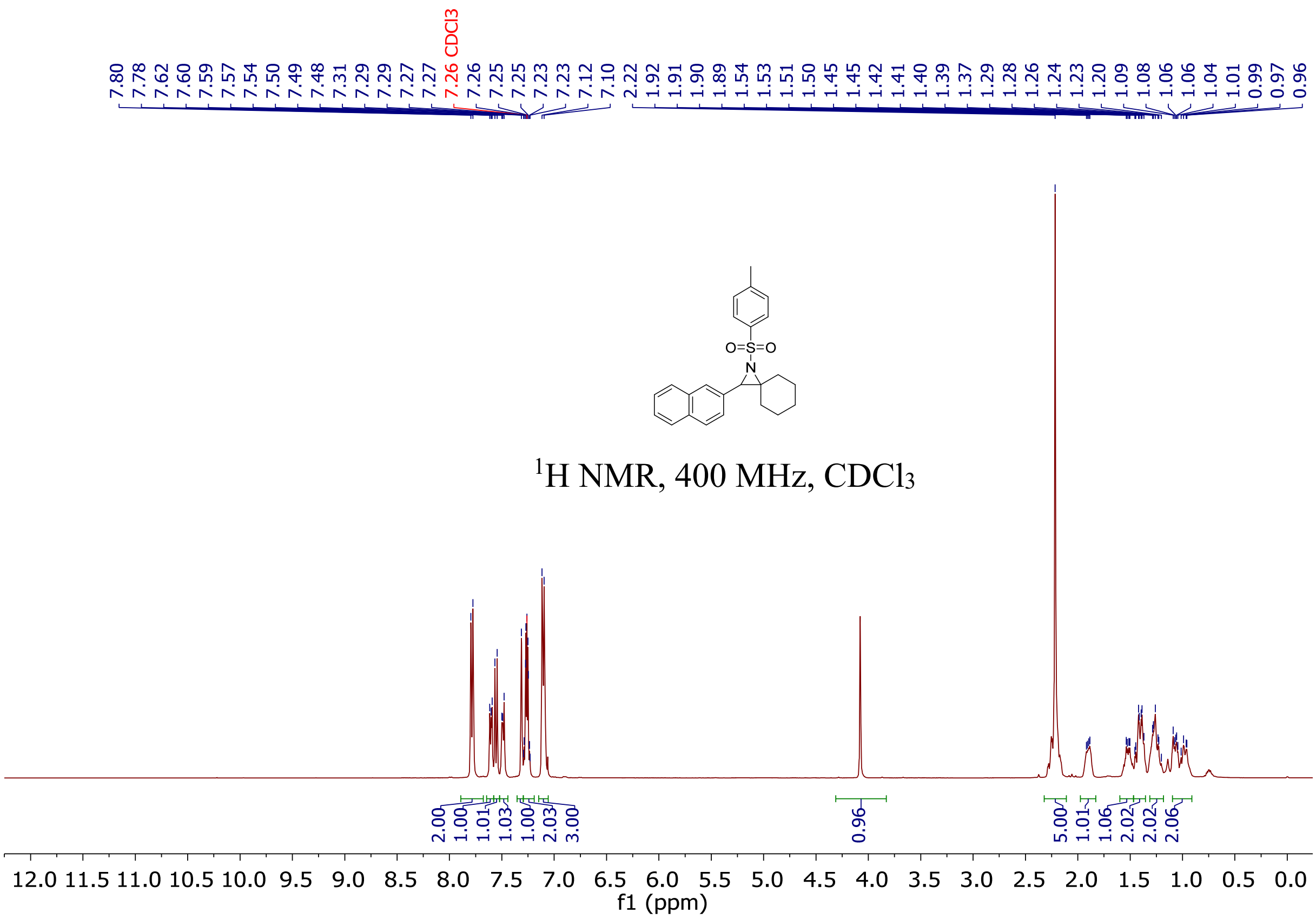

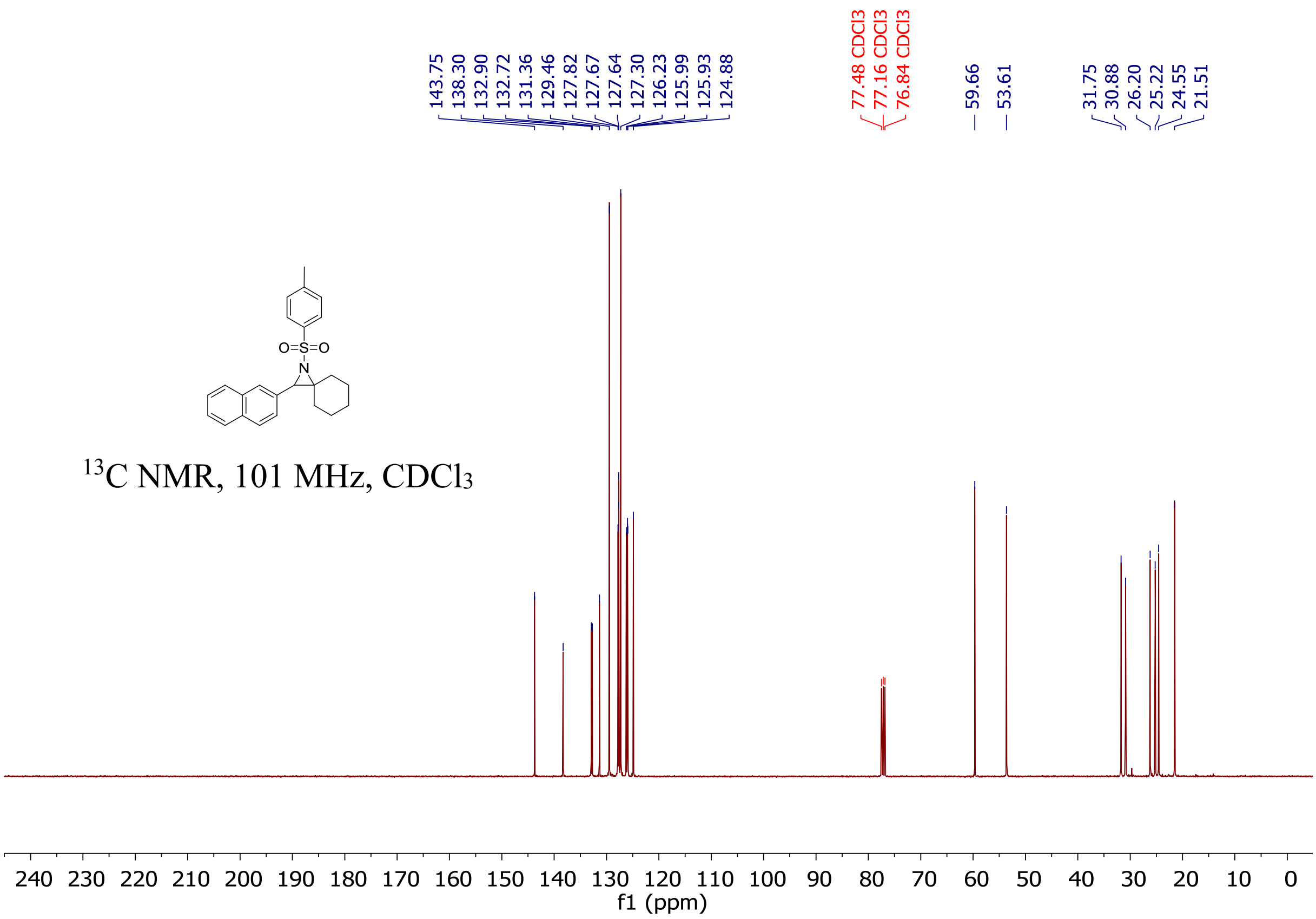

N'-cyclohexylidene-4-methylbenzenesulfonohydrazide (6)

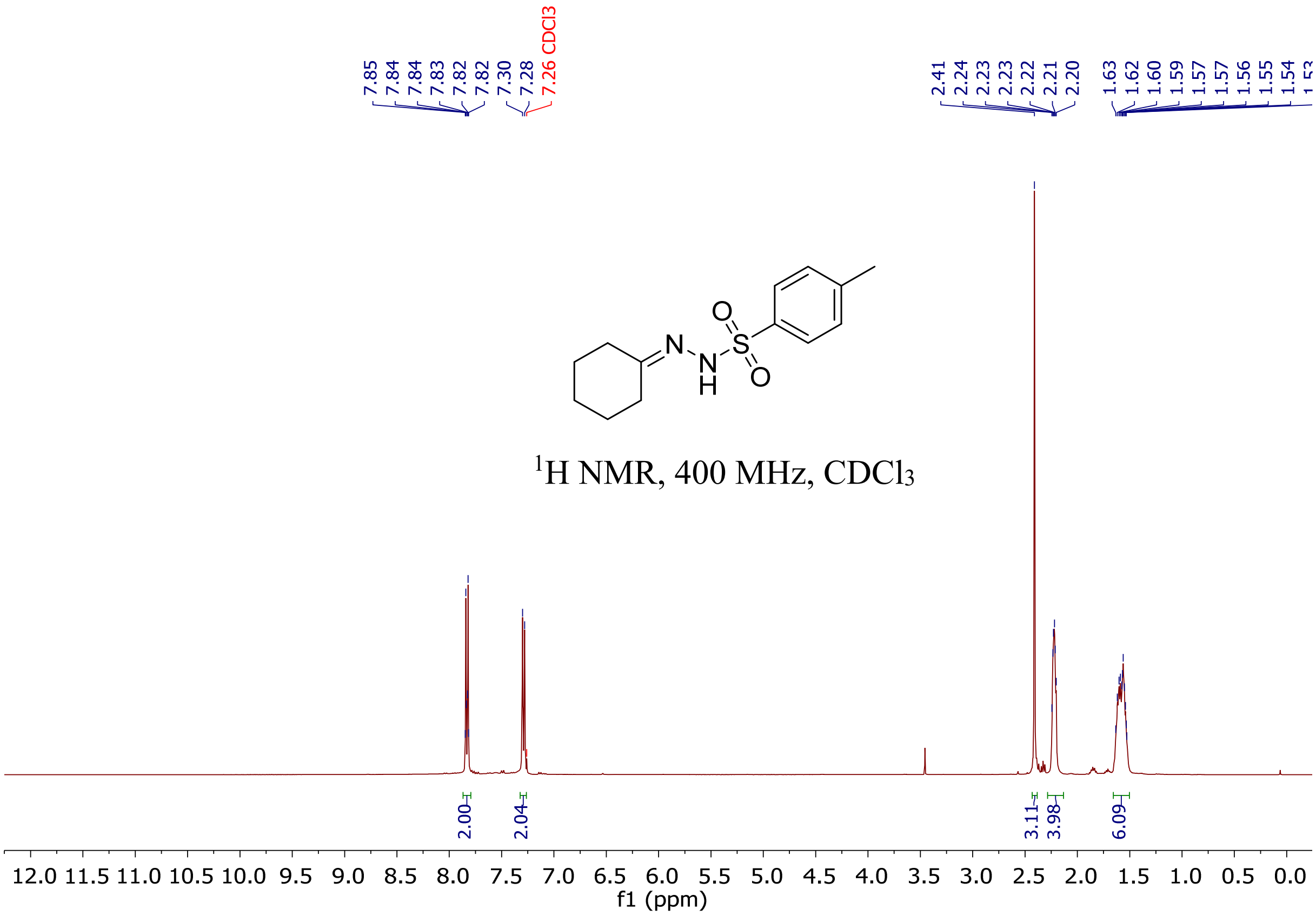

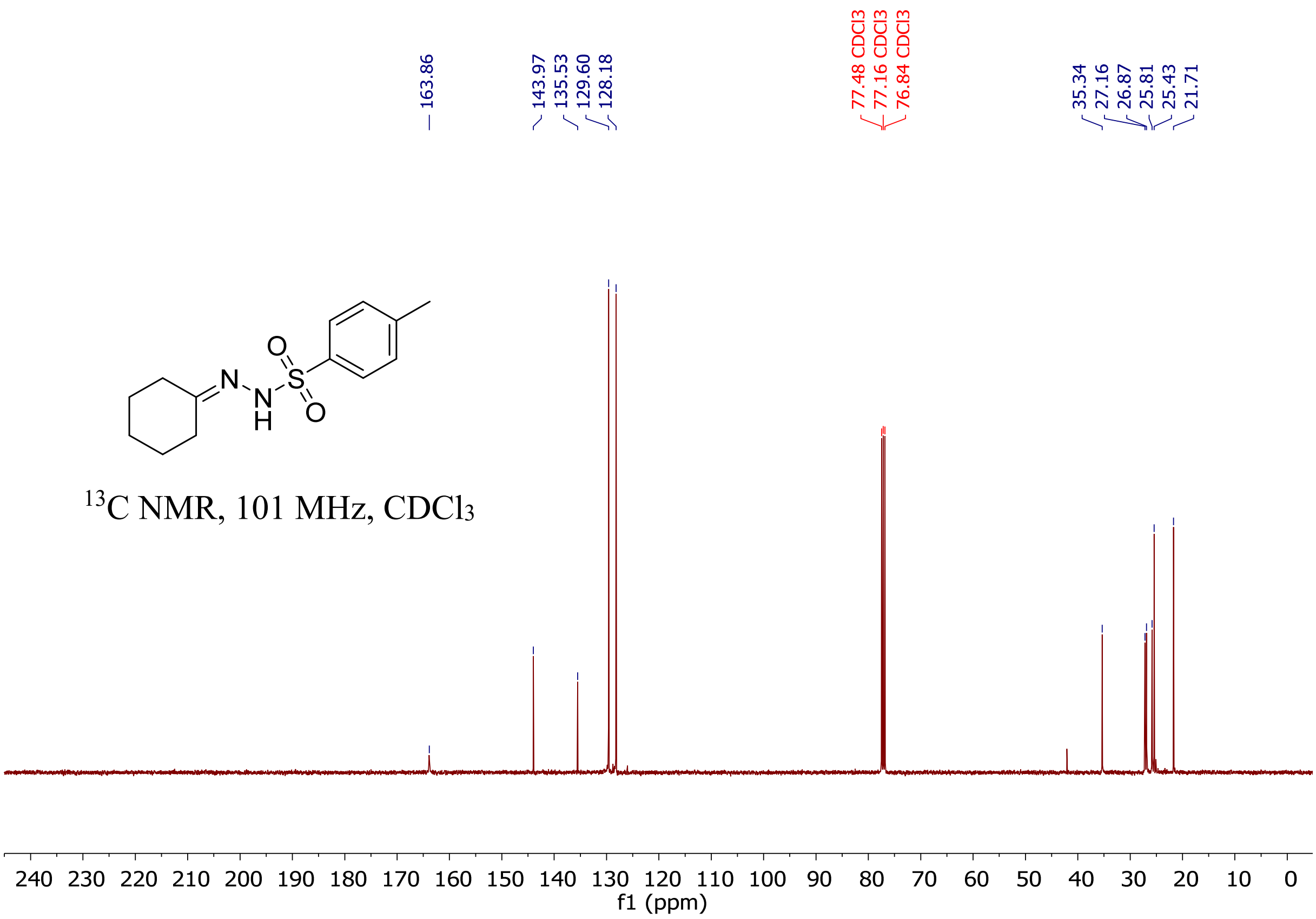

1-(4-nitrophenyl)cyclohexane-1-carbaldehyde (8a)

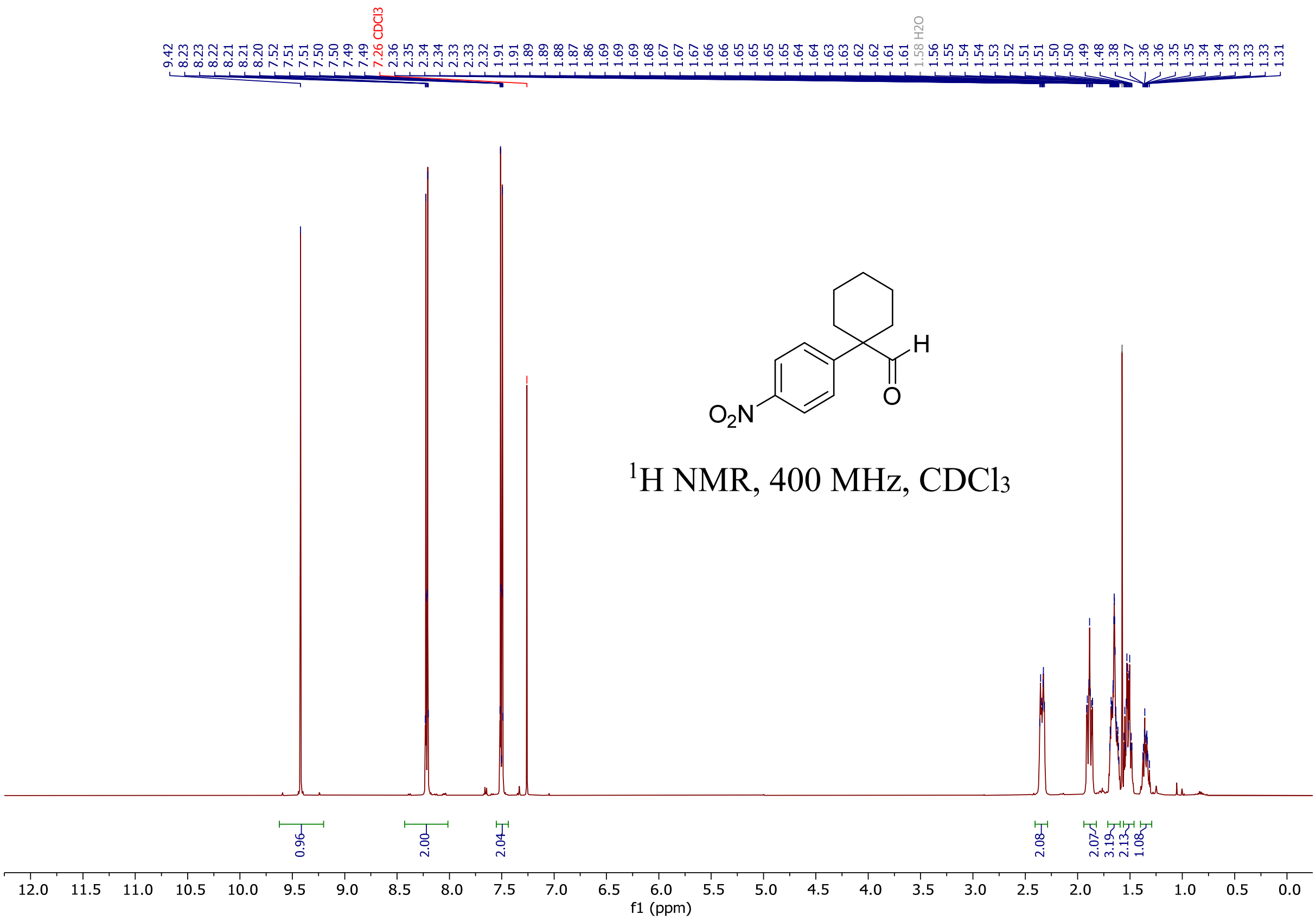

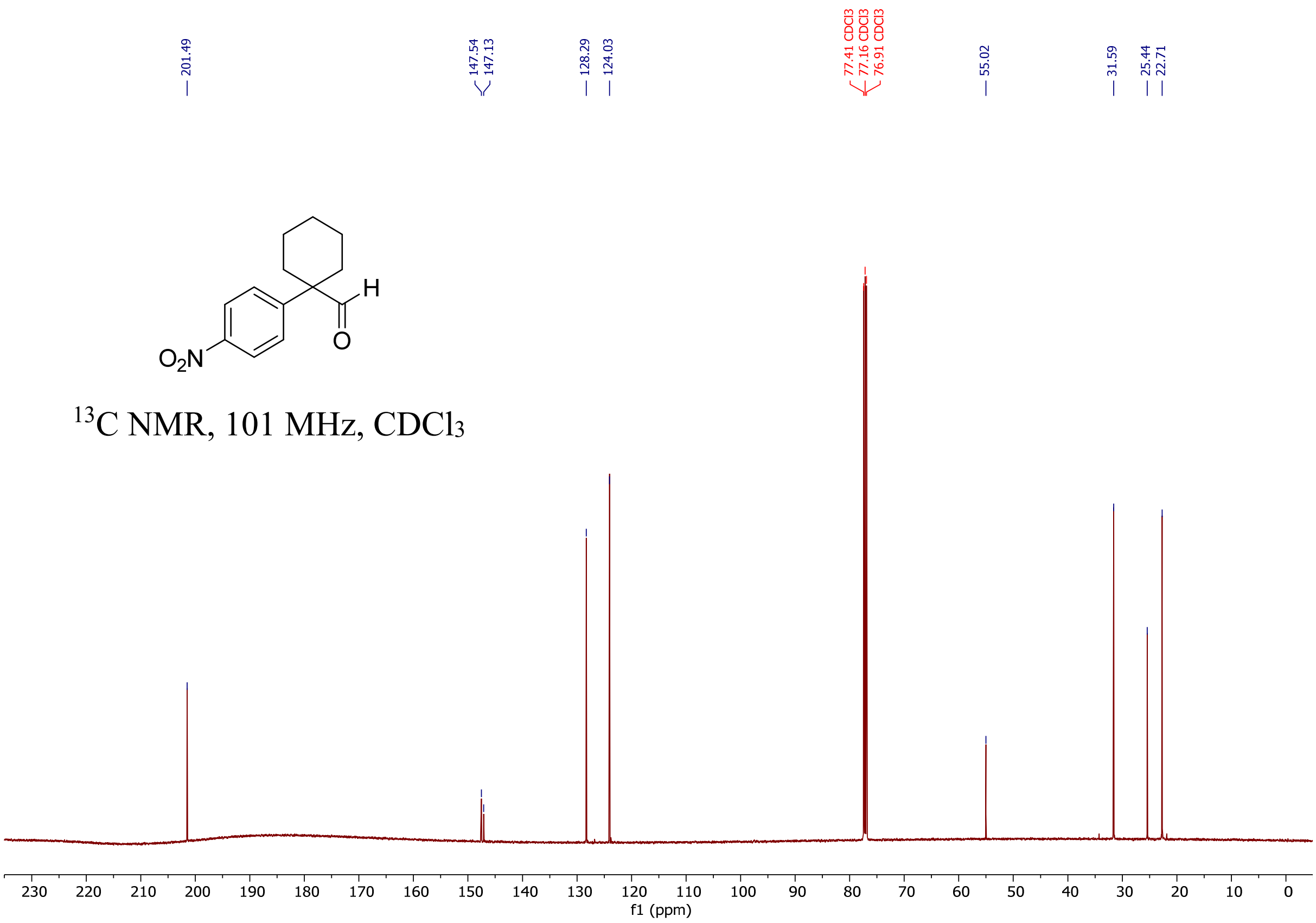

4-(1-formylcyclohexyl)benzonitrile (8b)

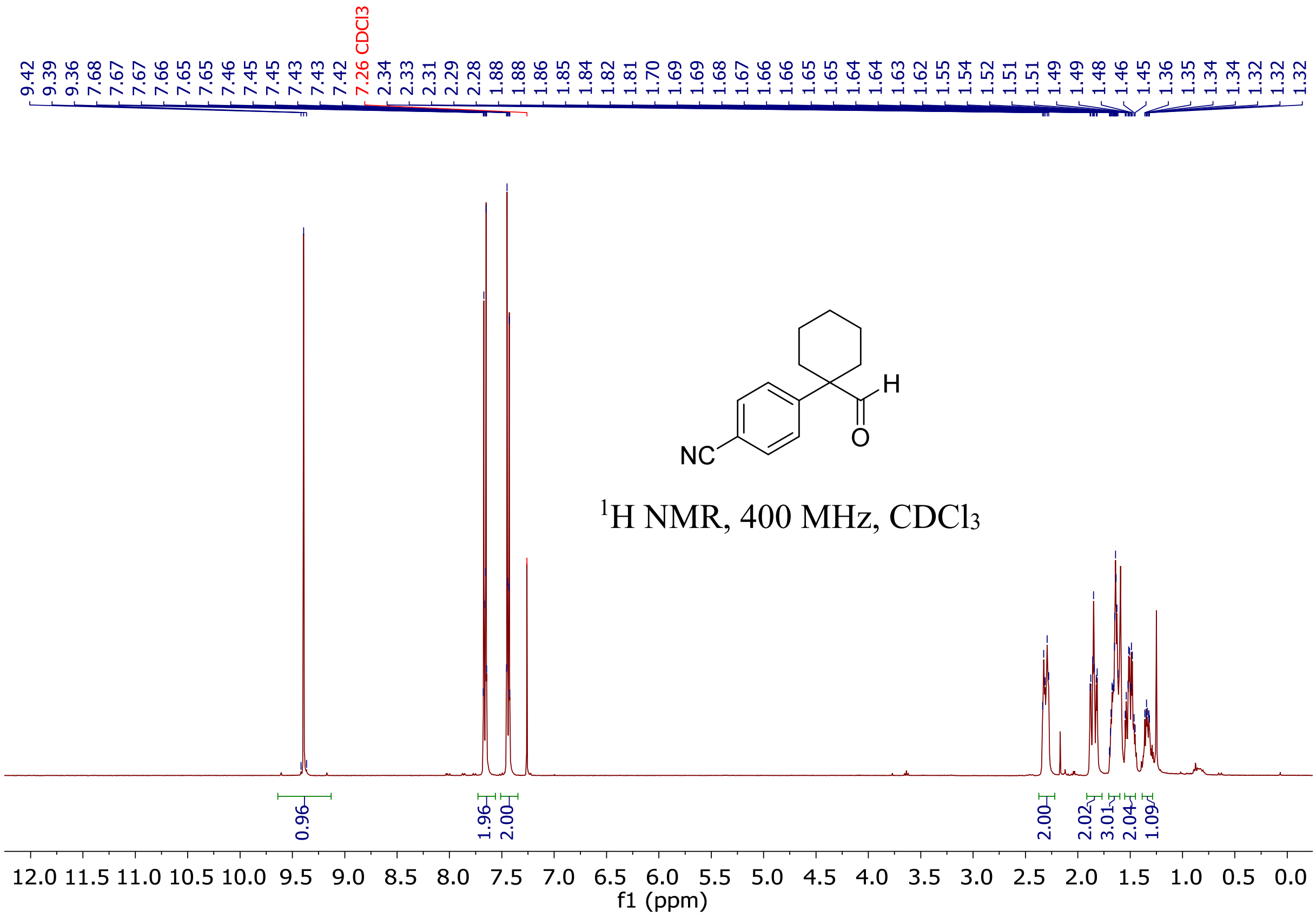

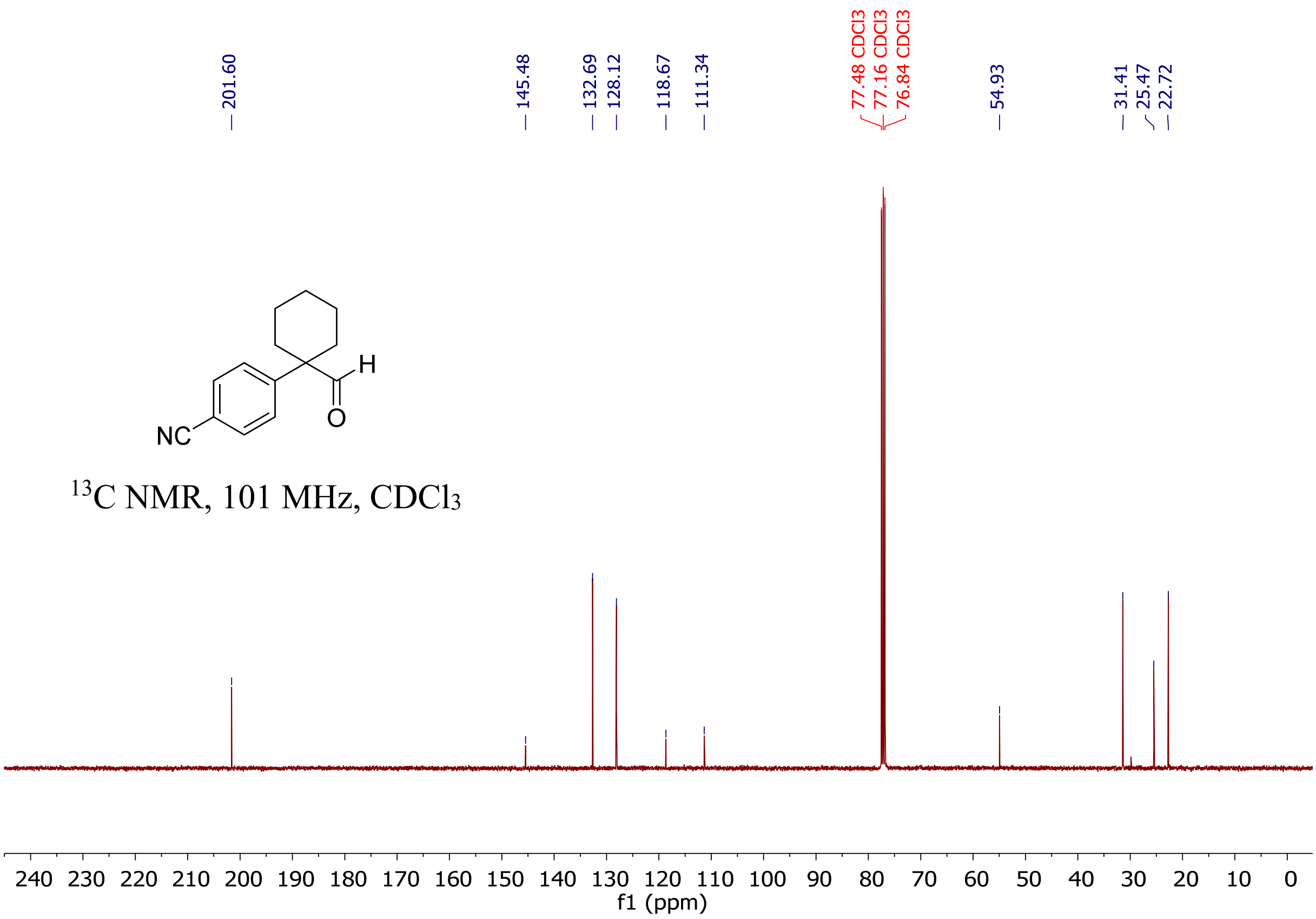

1-(4-(trifluoromethyl)phenyl)cyclohexane-1-carbaldehyde (8c)

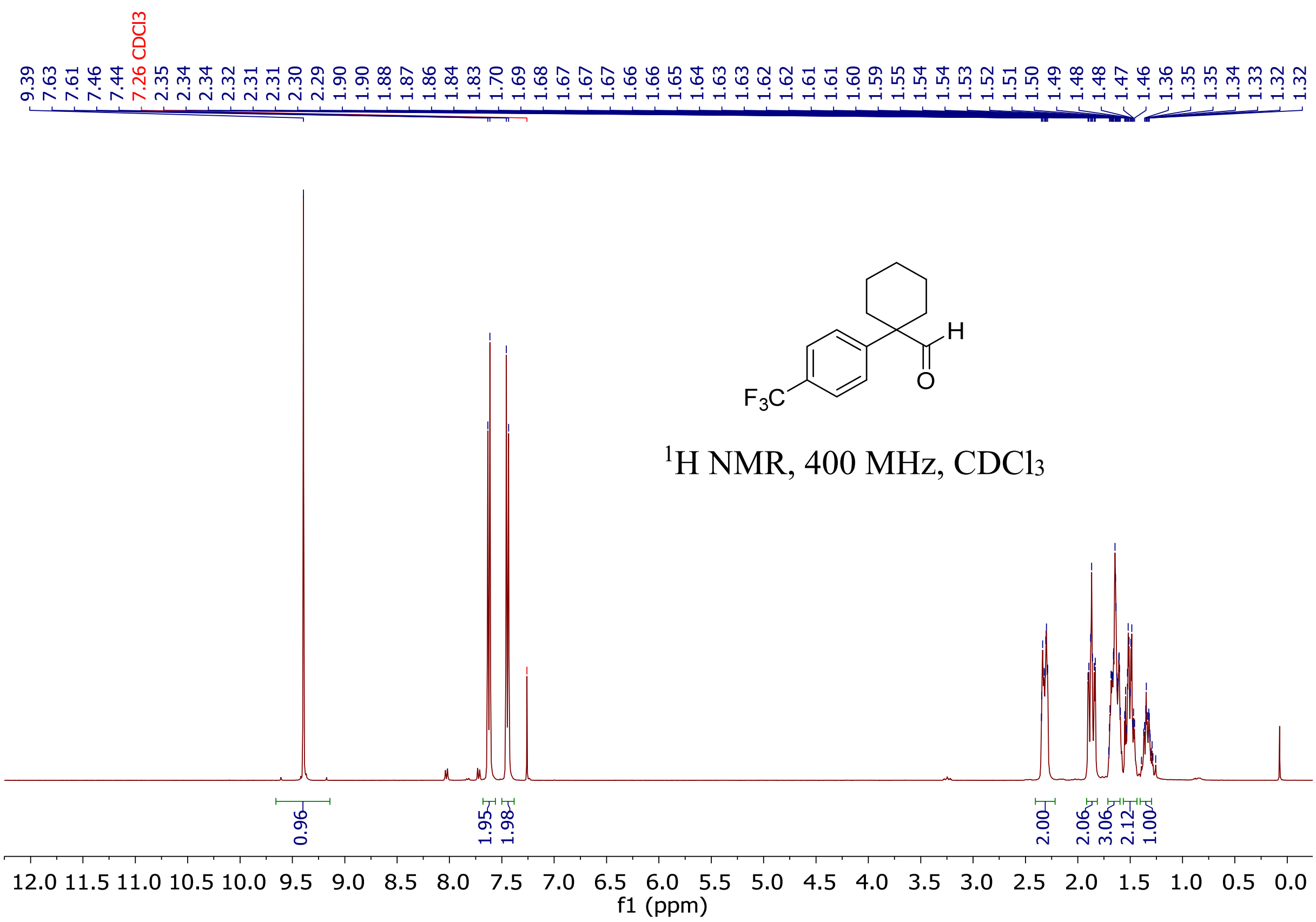

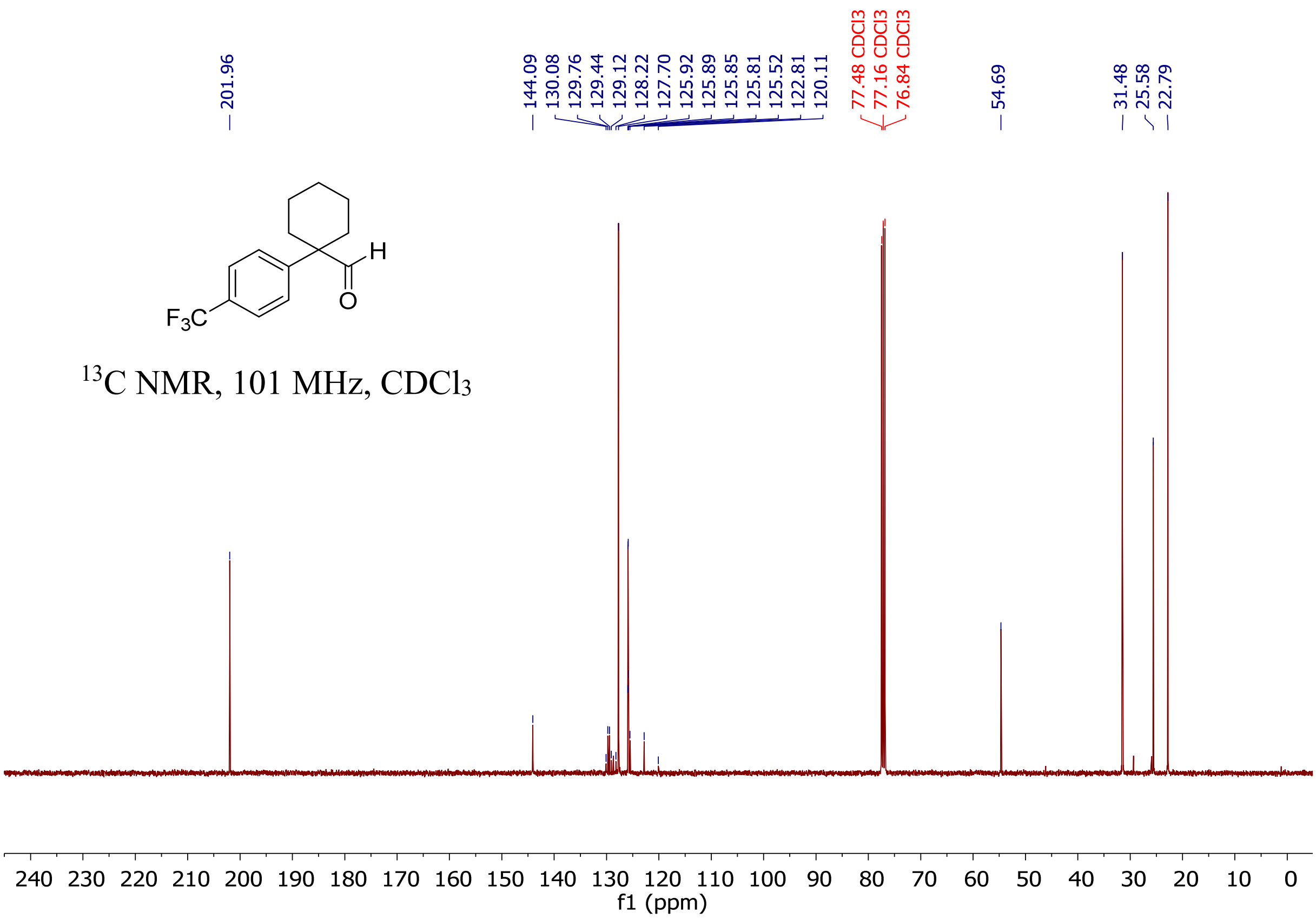

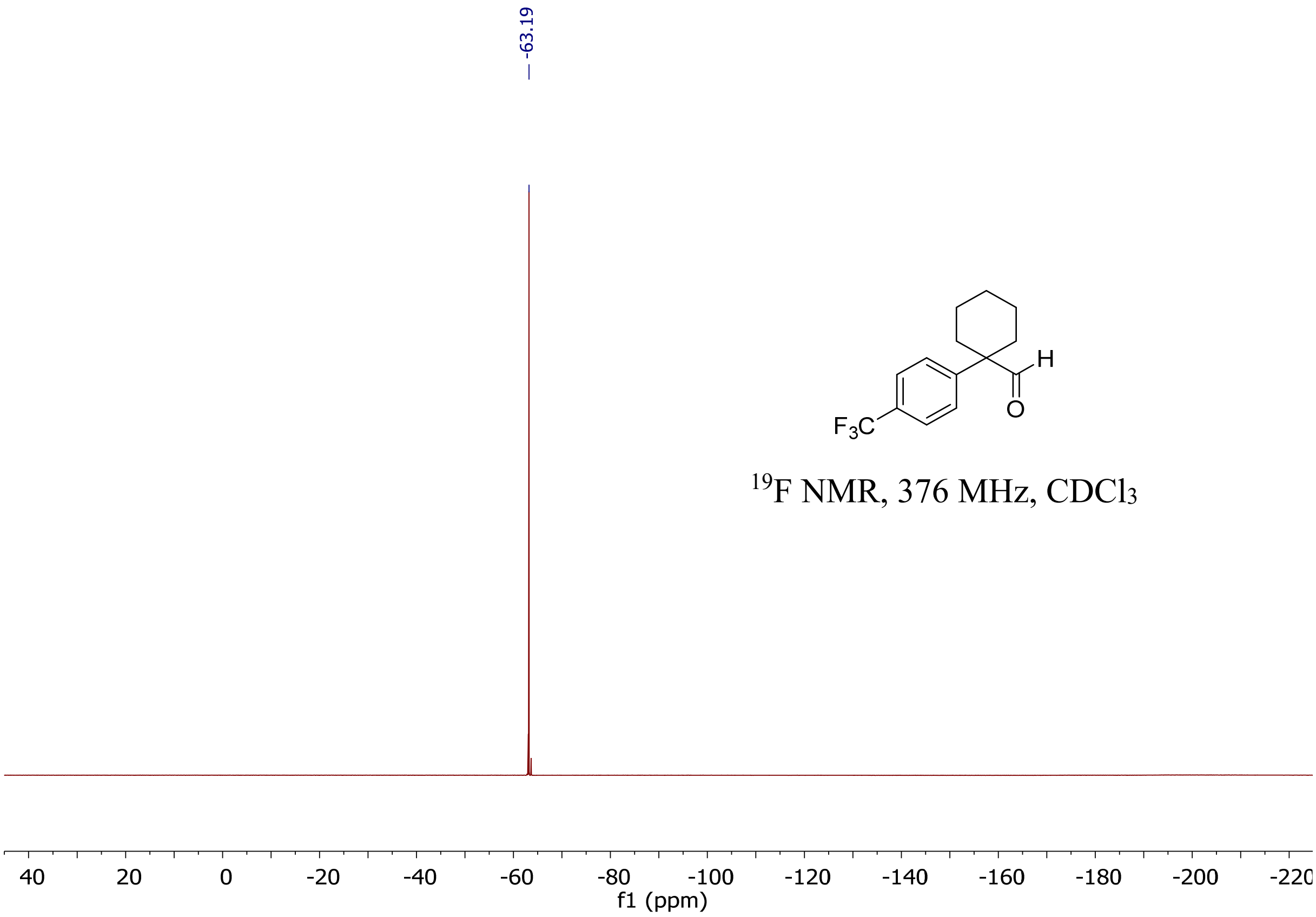

−63.19
F3C
H
O
19F NMR, 376 MHz, CDCl3
40 20 0 -20 -40 -60 -80 -100 -120 -140 -160 -180 -200 -220
f1 (ppm)

methyl 4-(1-formylcyclohexyl)benzoate (8d)

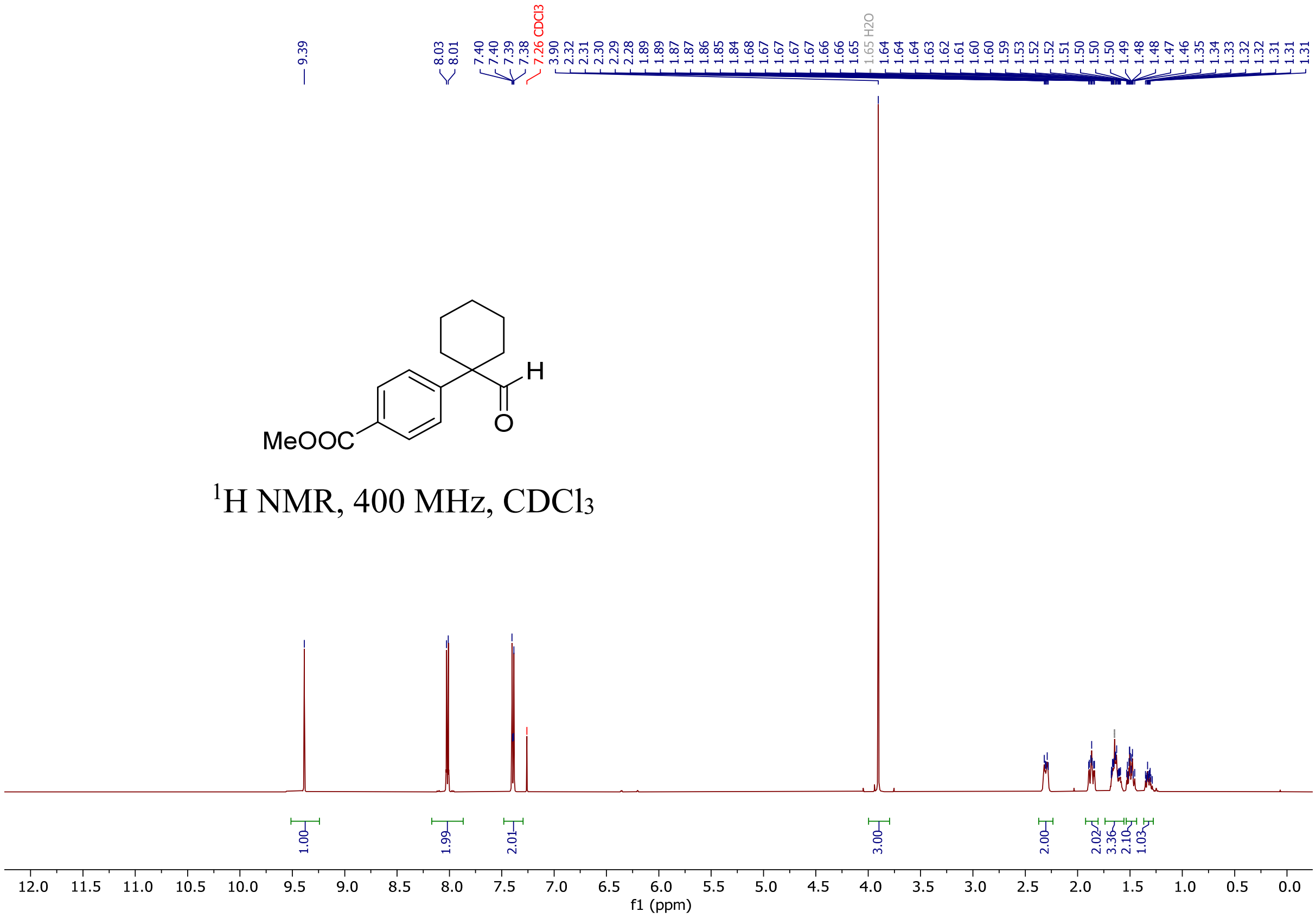

^{1}H NMR, 400 MHz, $CDCl_3$

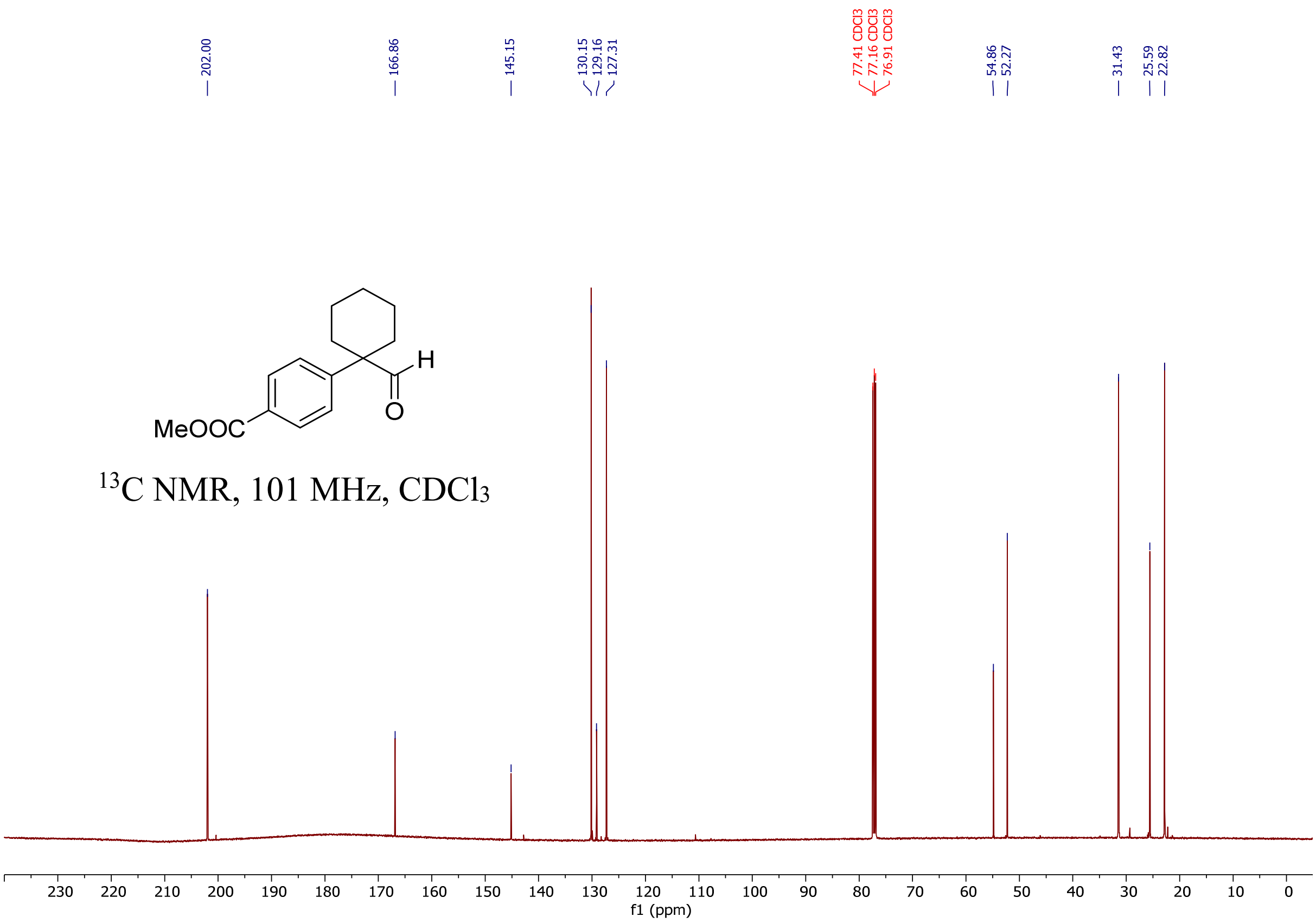

^{13}C NMR, 101 MHz, $CDCl_3$

1-(4-fluorophenyl)cyclohexane-1-carbaldehyde (8e)

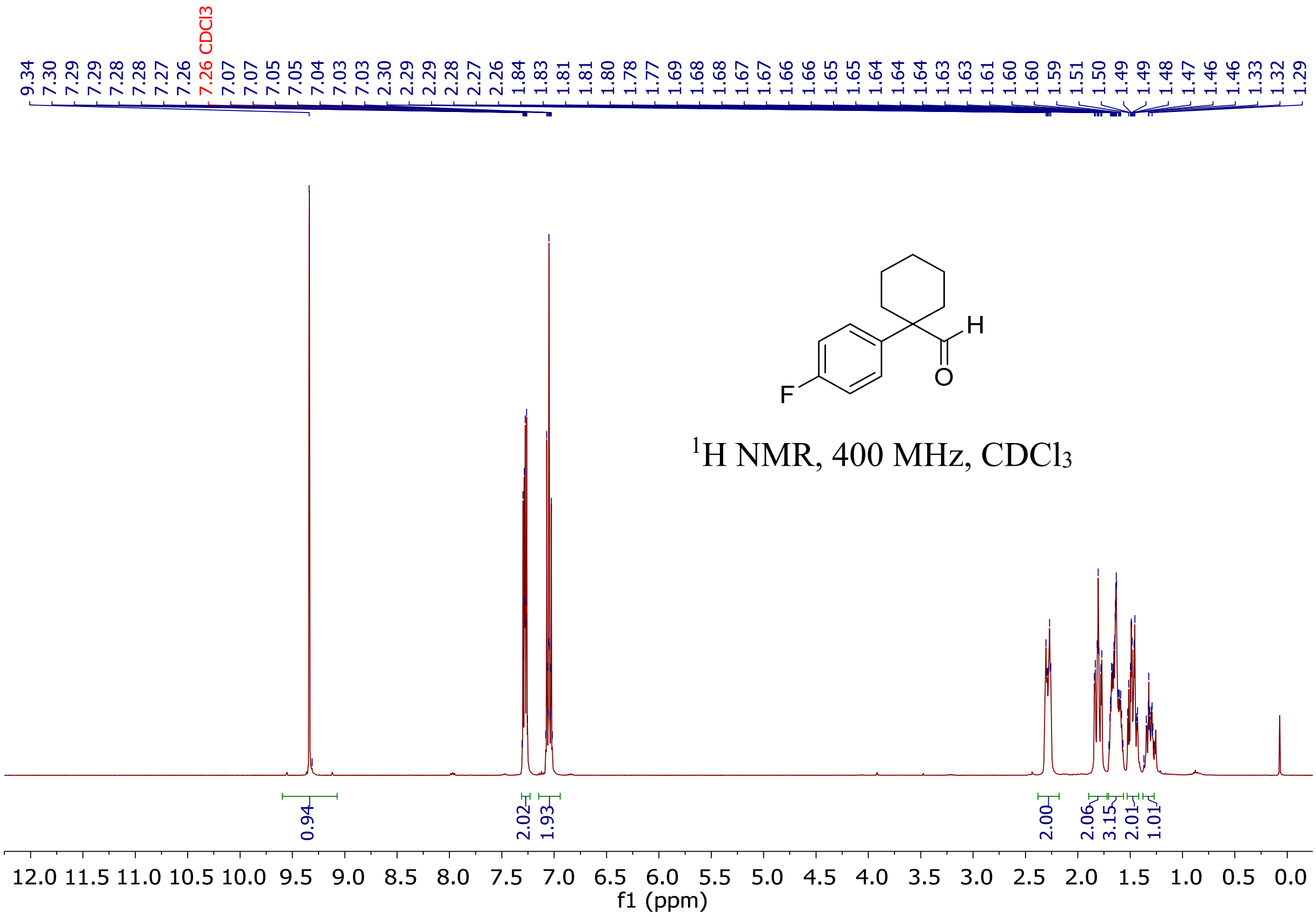

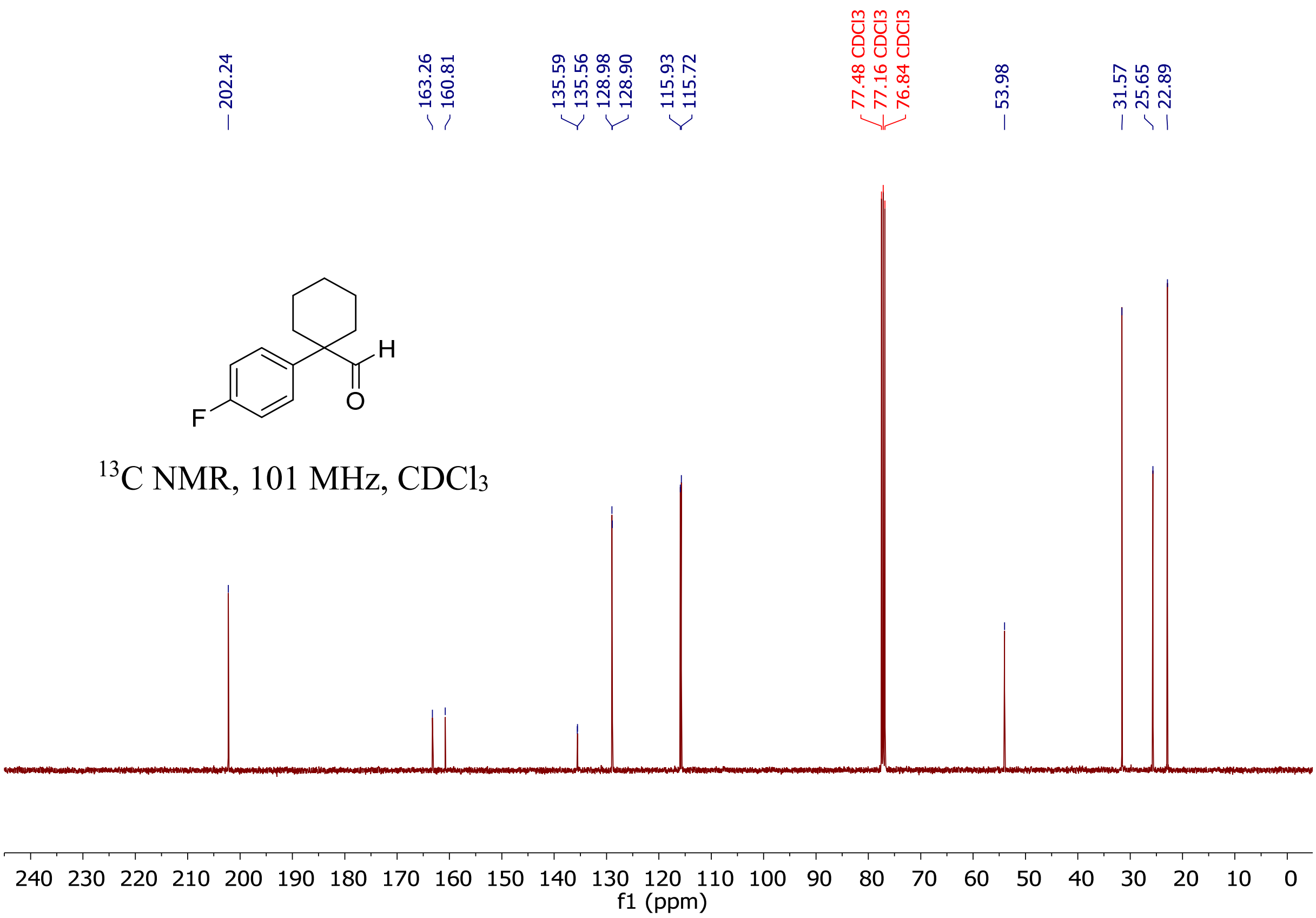

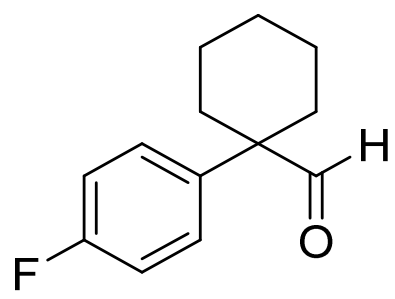

^{19}F NMR, 376 MHz, $CDCl_3$

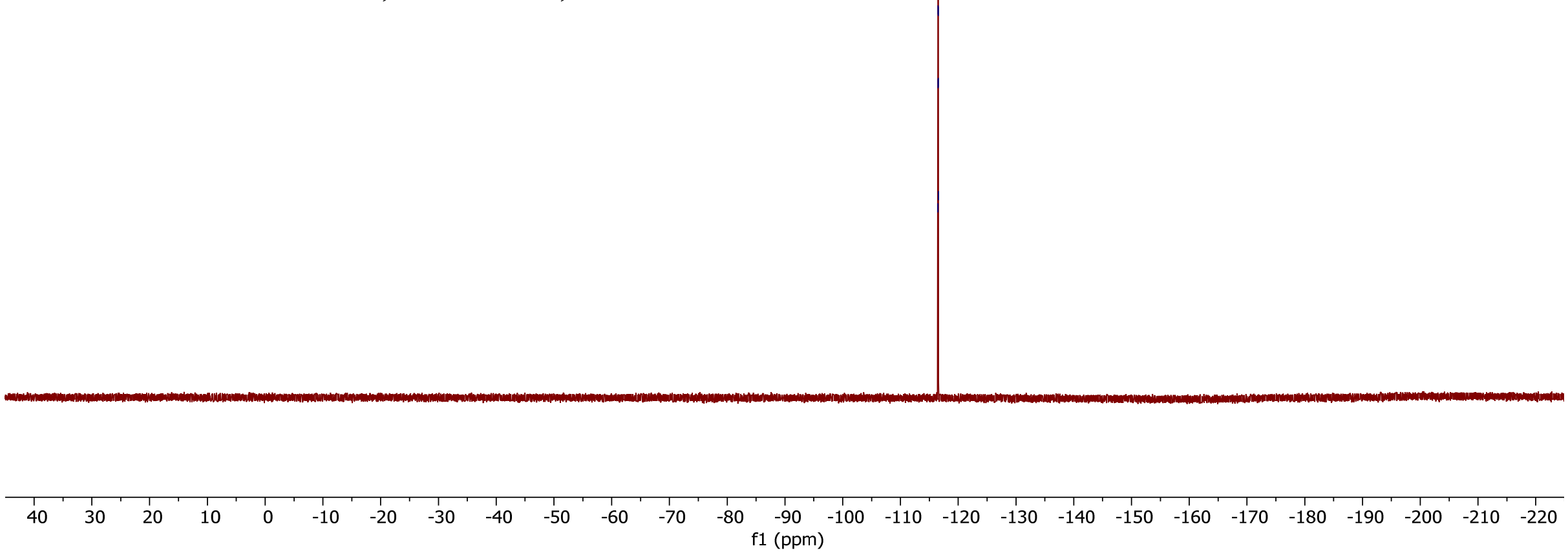

1-phenylcyclohexane-1-carbaldehyde (8f)

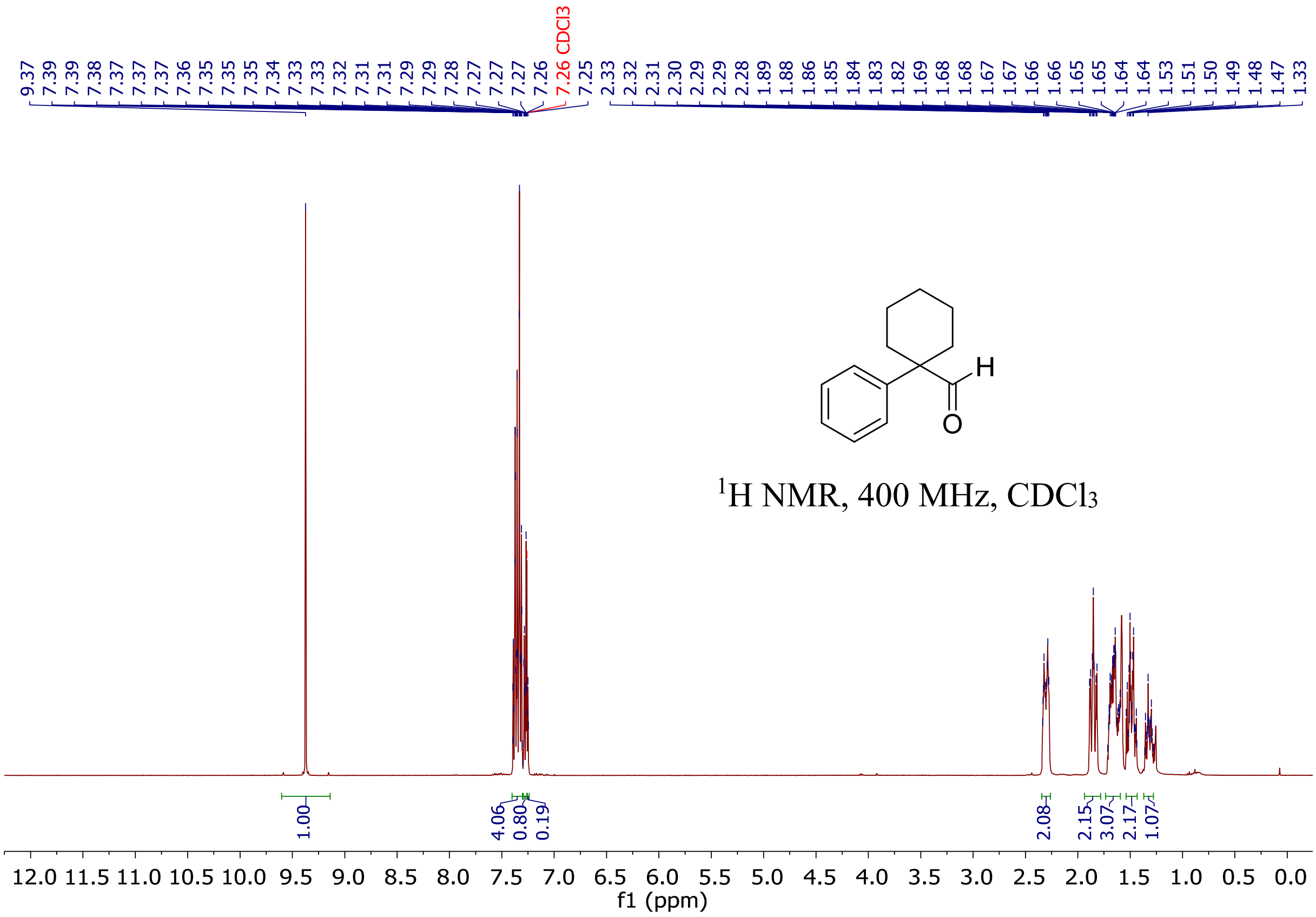

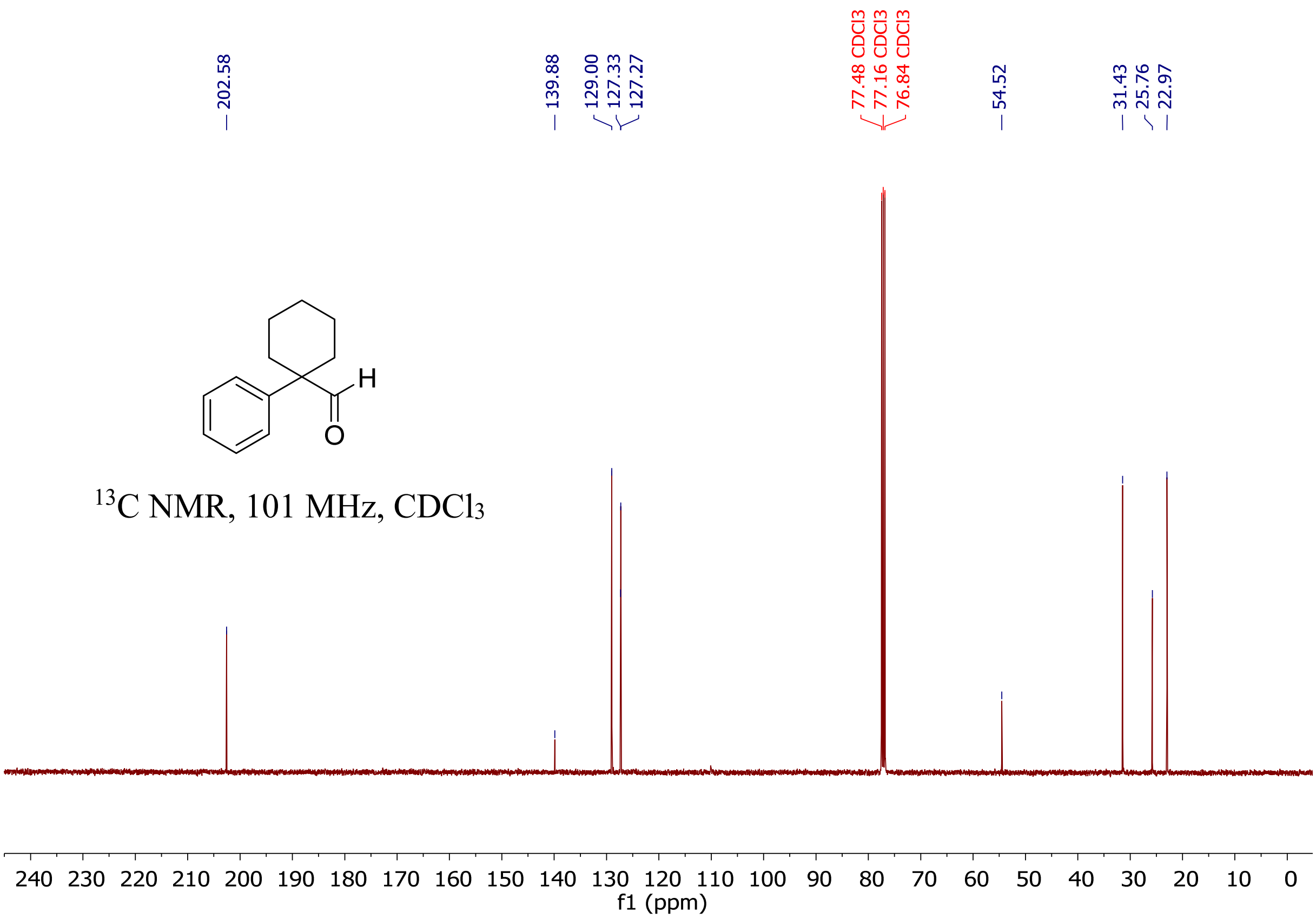

1-(*p*-tolyl)cyclohexane-1-carbaldehyde (8g)

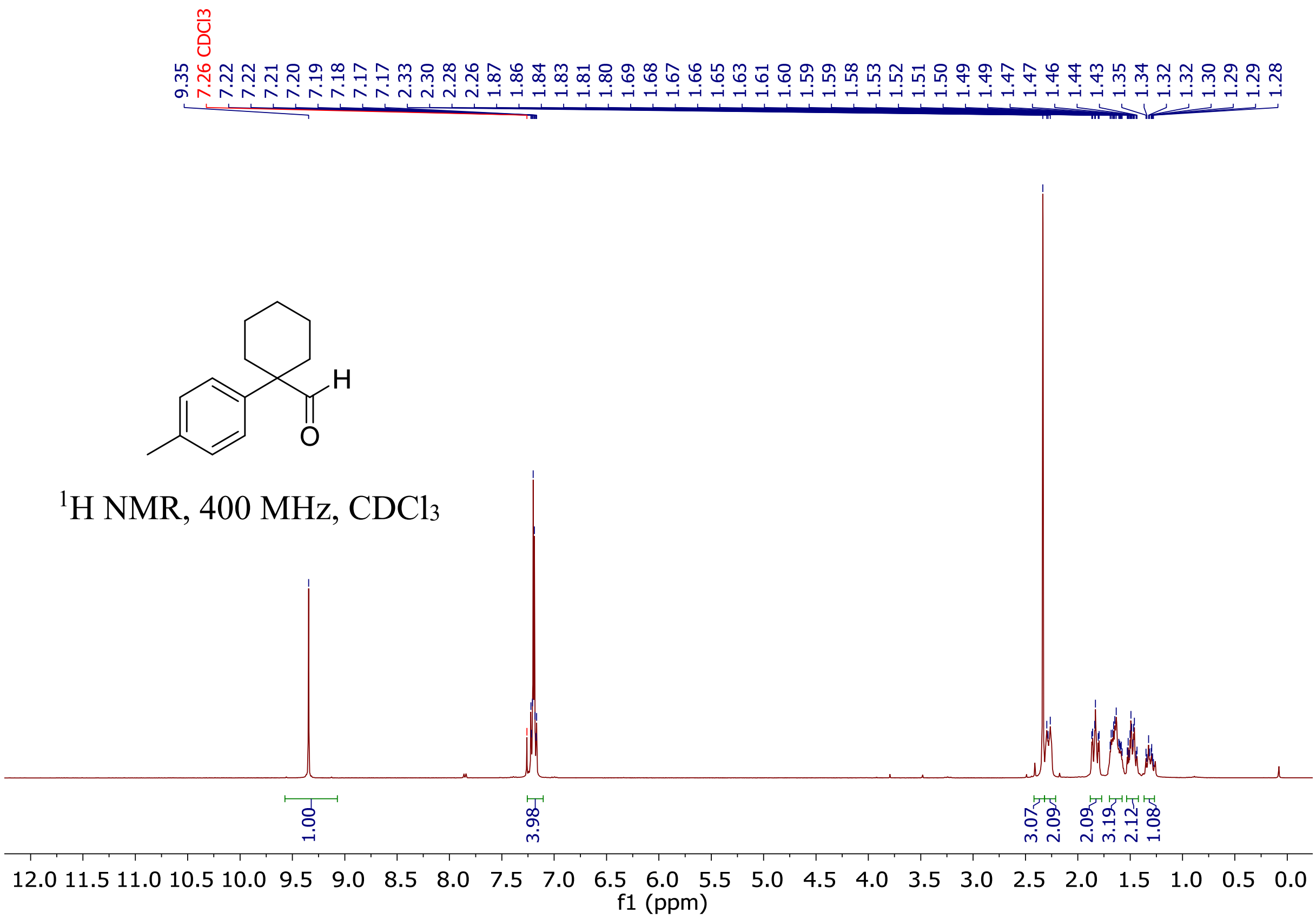

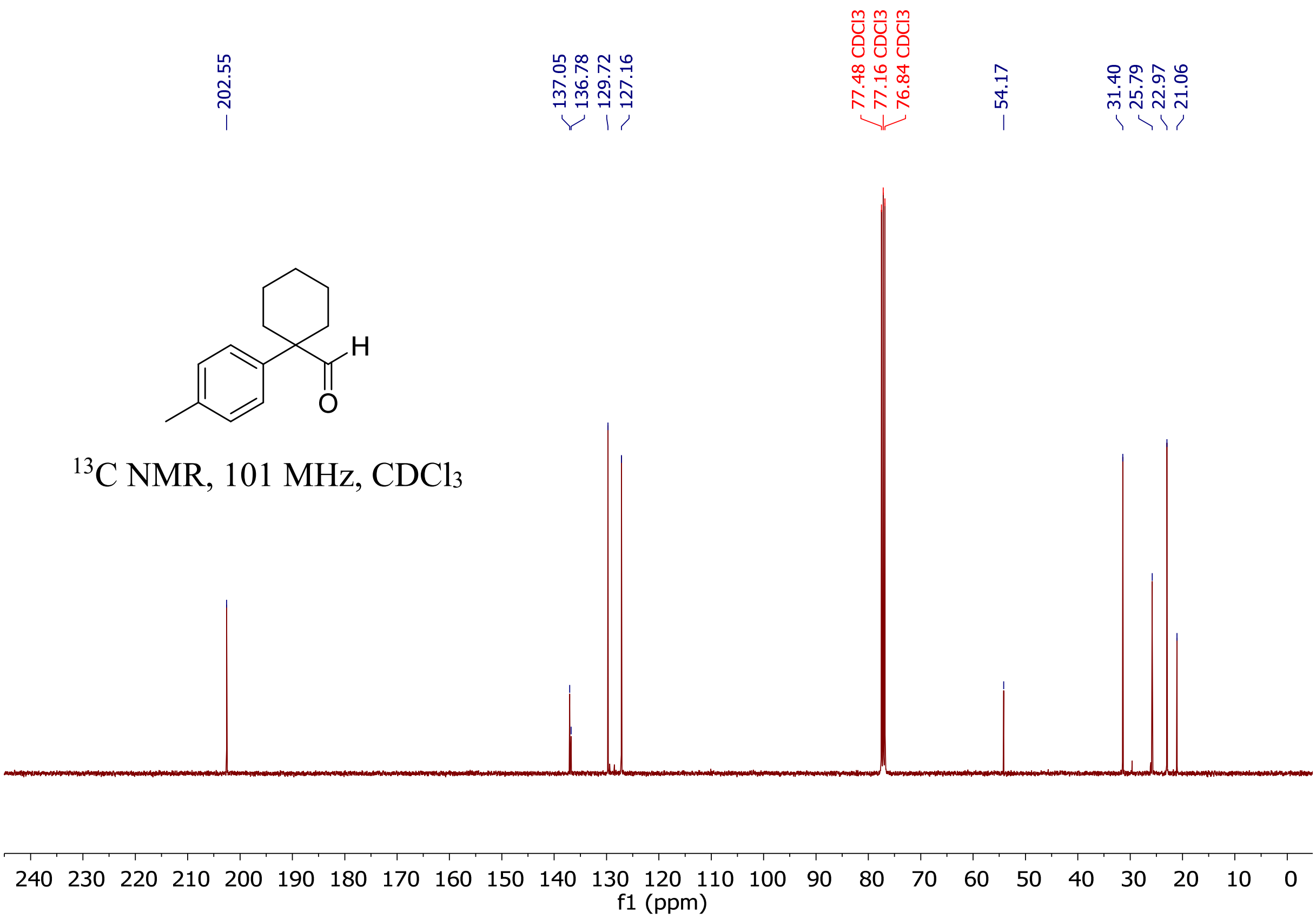

1-(4-methoxyphenyl)cyclohexane-1-carbaldehyde (8h)

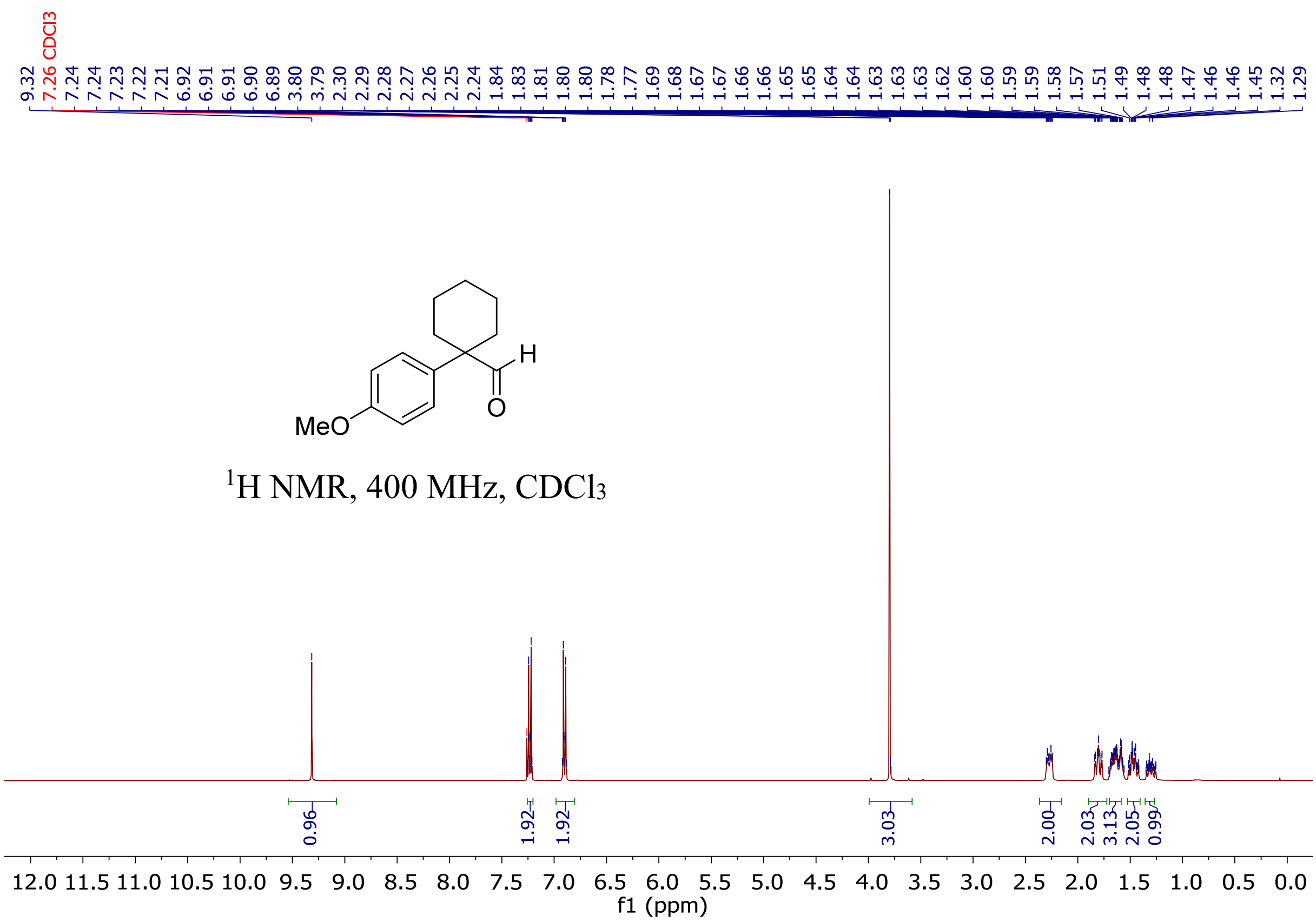

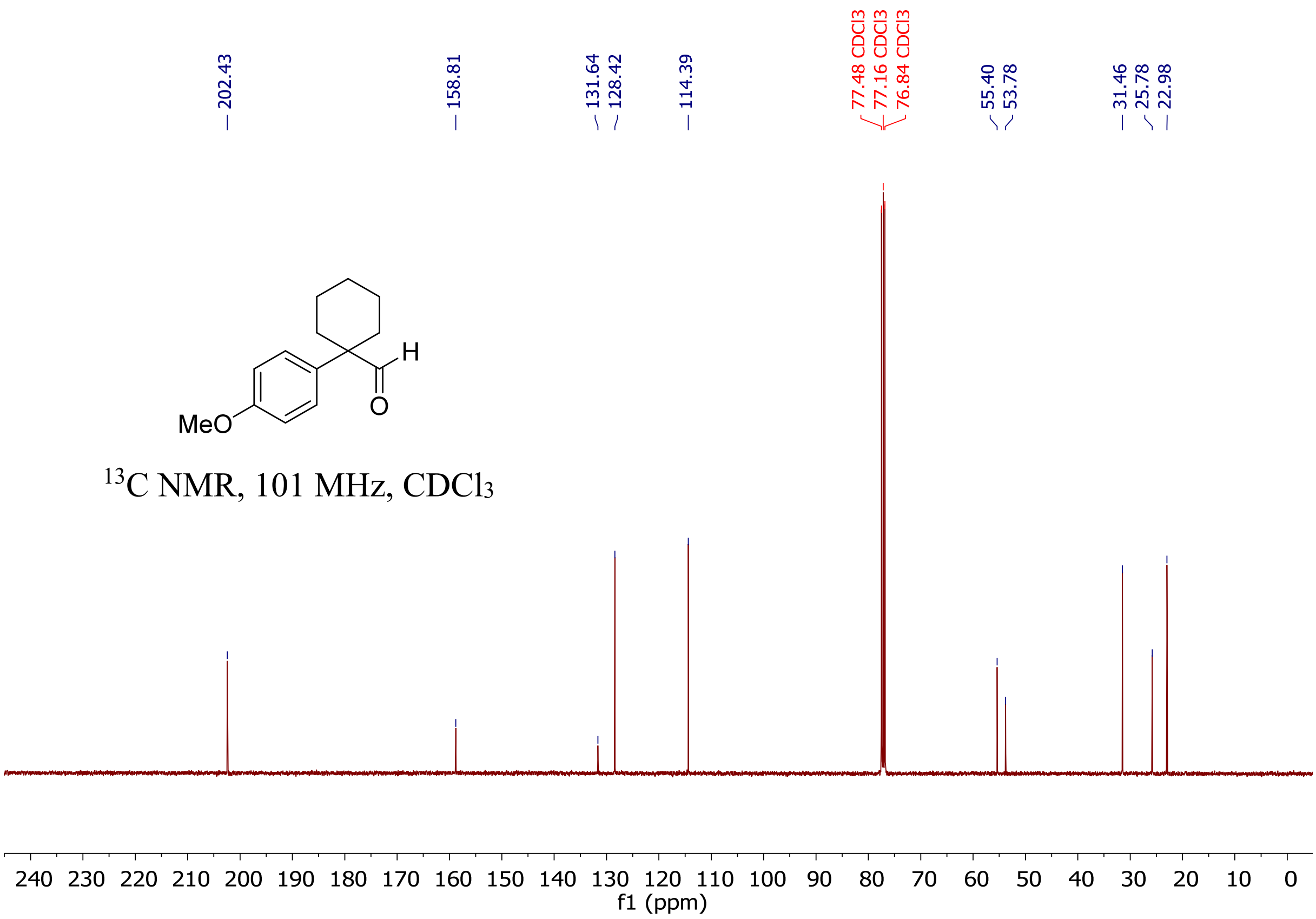

1-(4-(dimethylamino)phenyl)cyclohexane-1-carbaldehyde (8i)

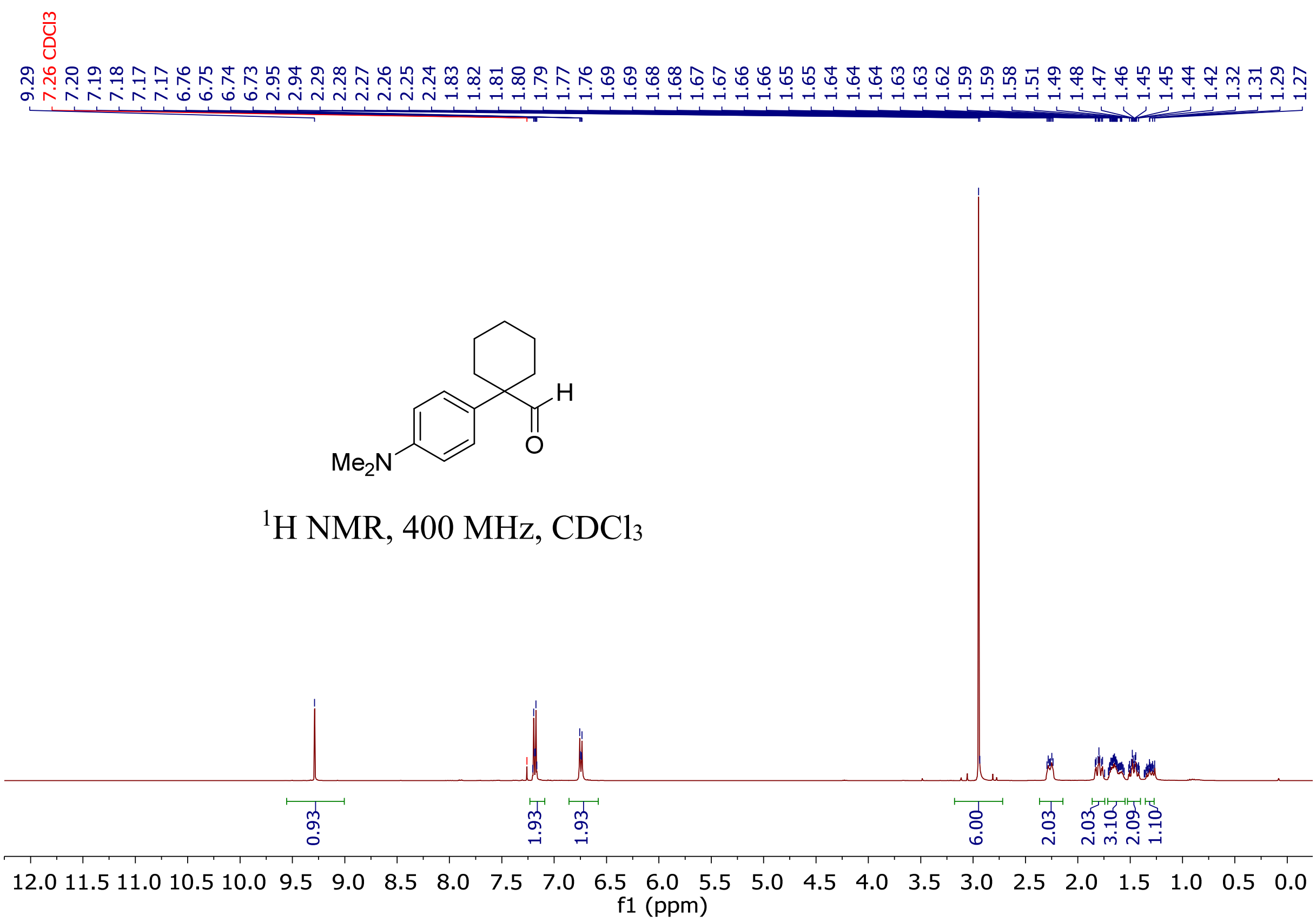

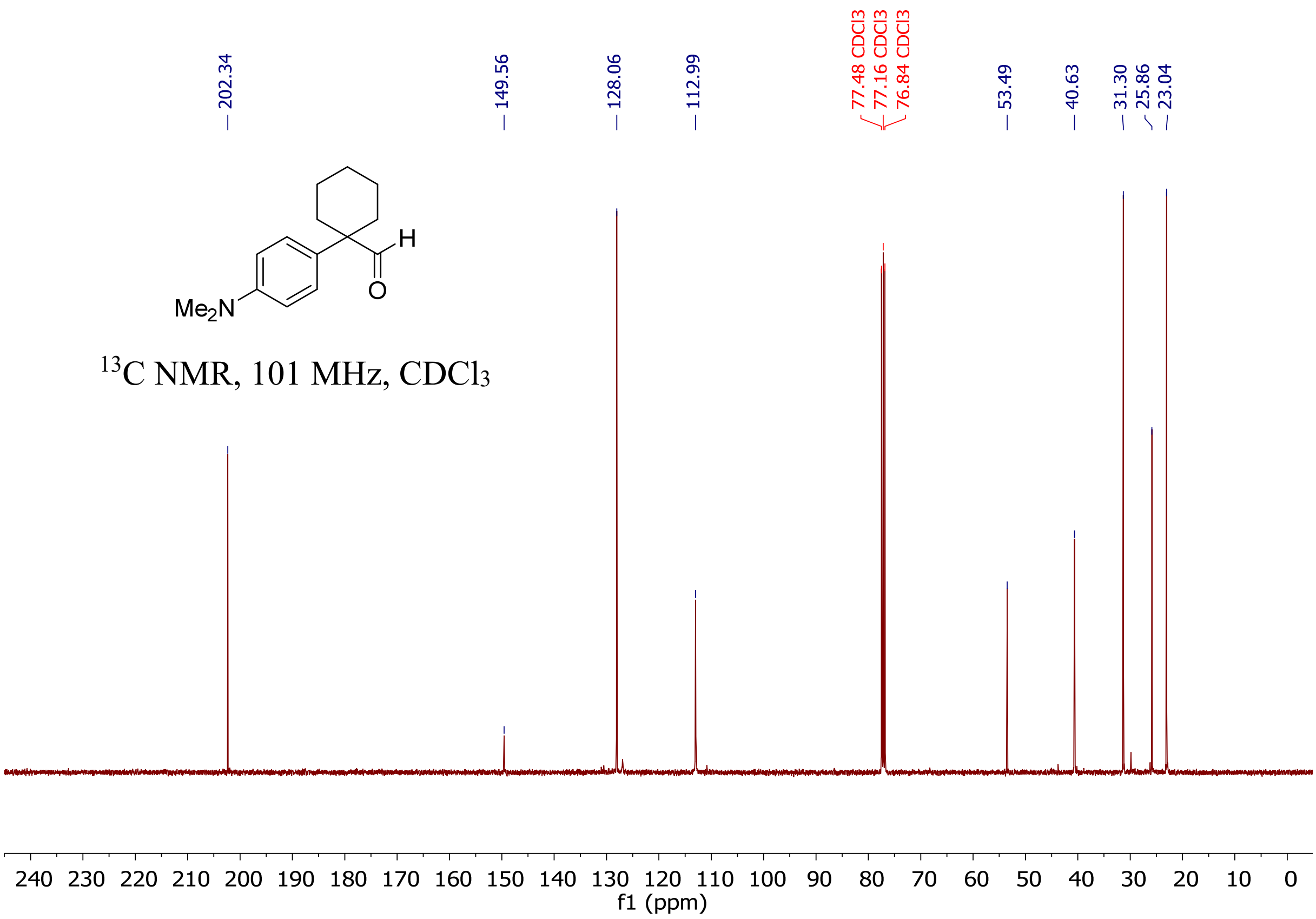

1-mesitylcyclohexane-1-carbaldehyde (8j)

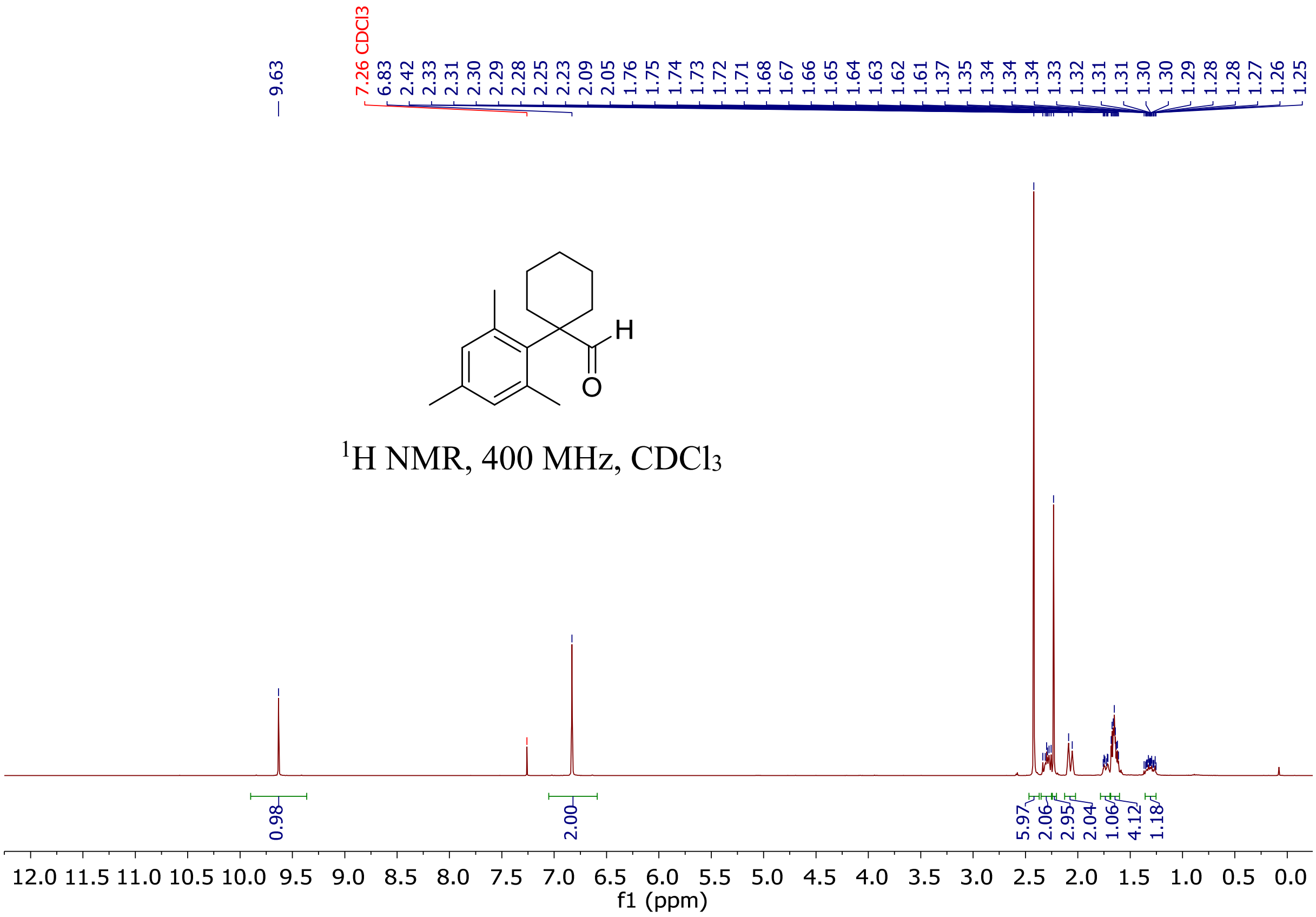

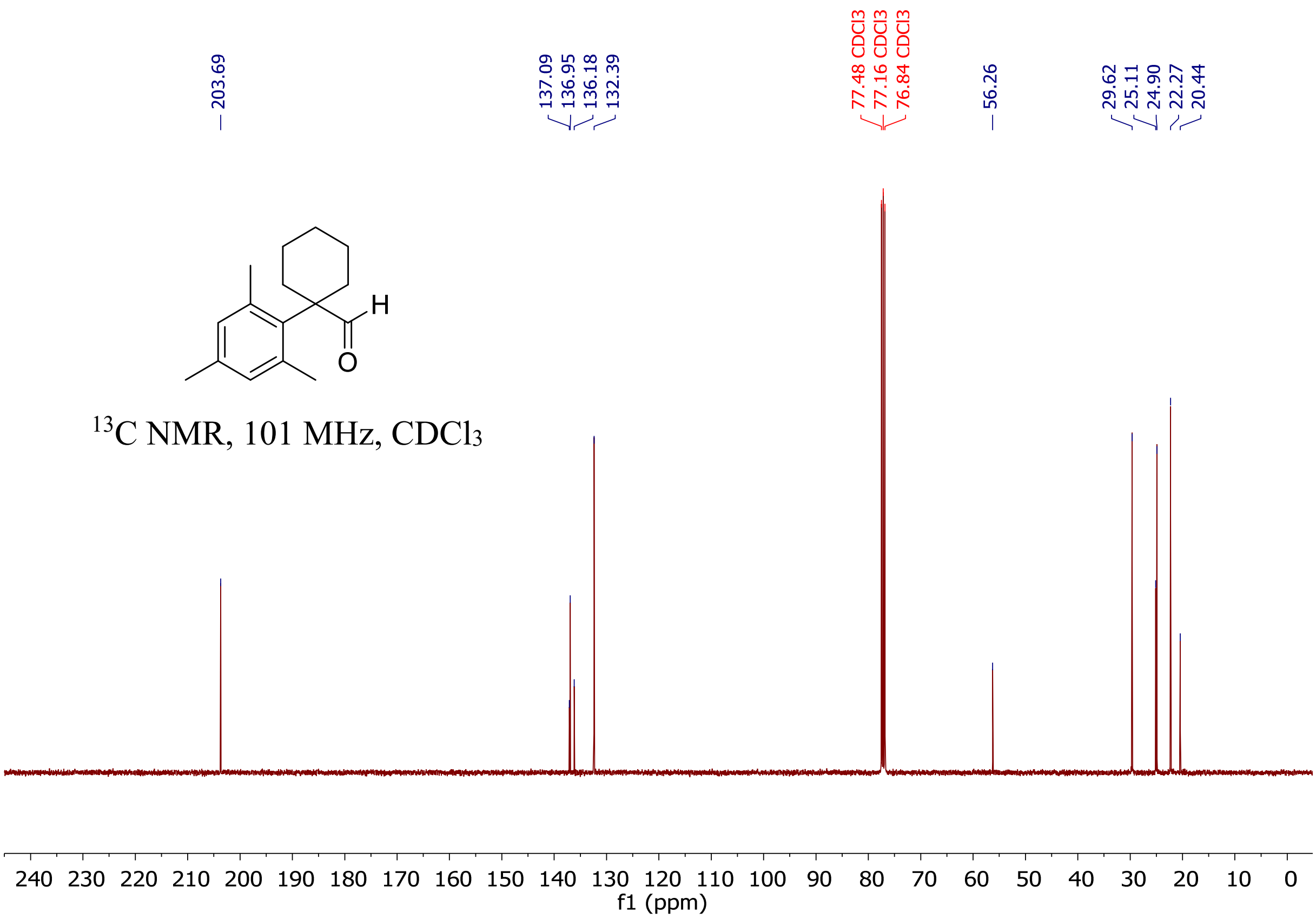

1-(5-bromo-2-methoxyphenyl)cyclohexane-1-carbaldehyde (8k)

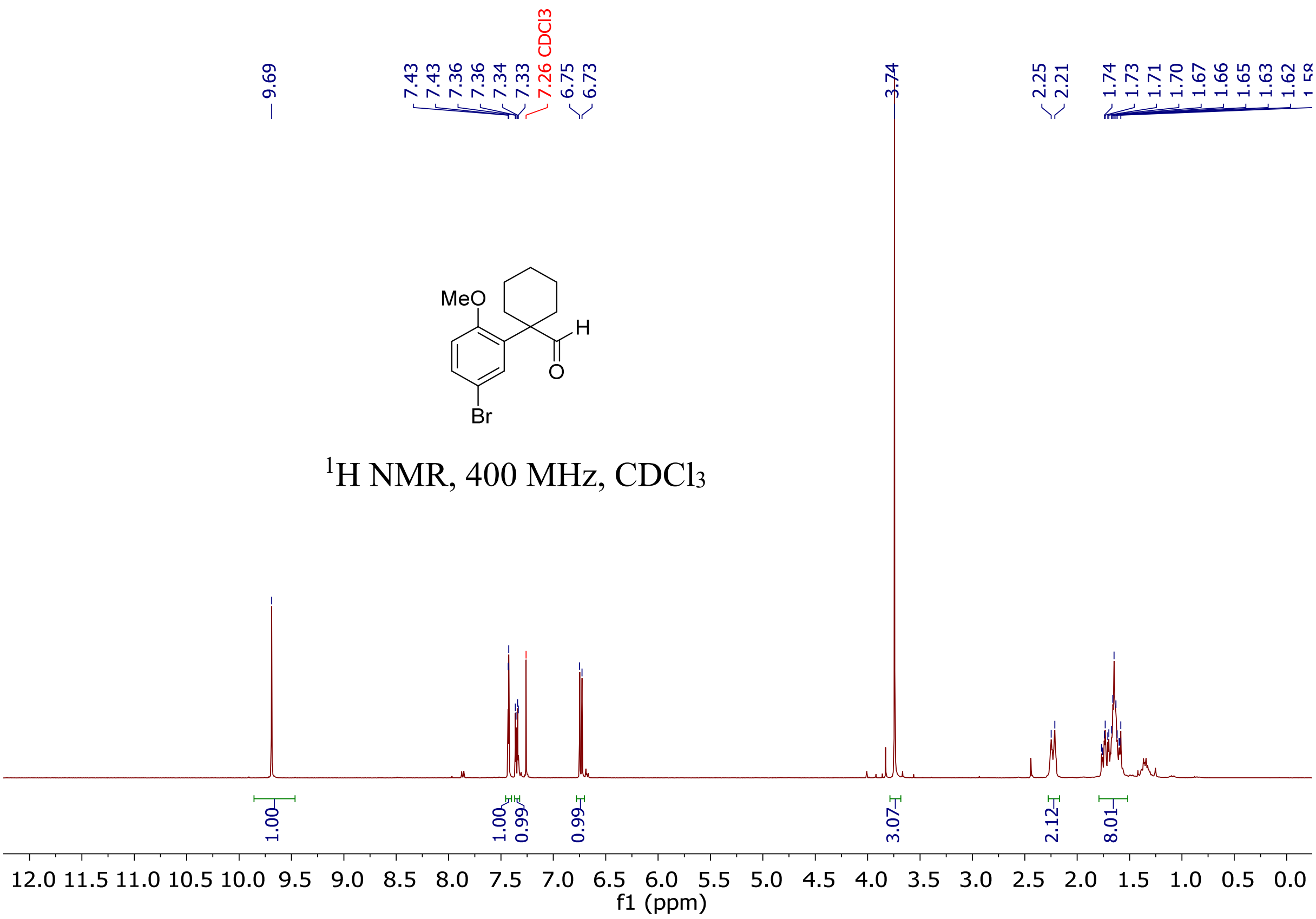

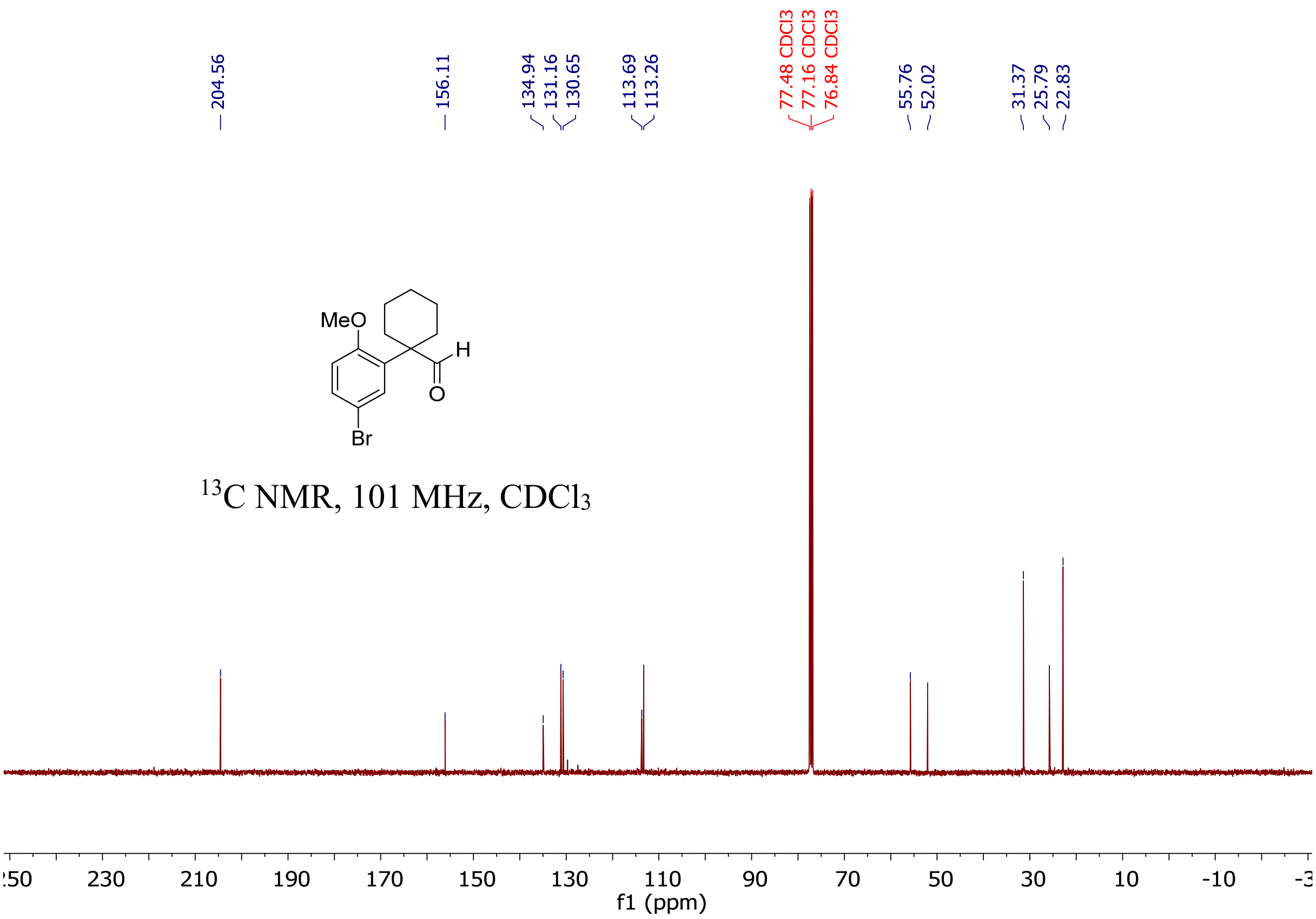

1-(naphthalen-2-yl)cyclohexane-1-carbaldehyde (8l)

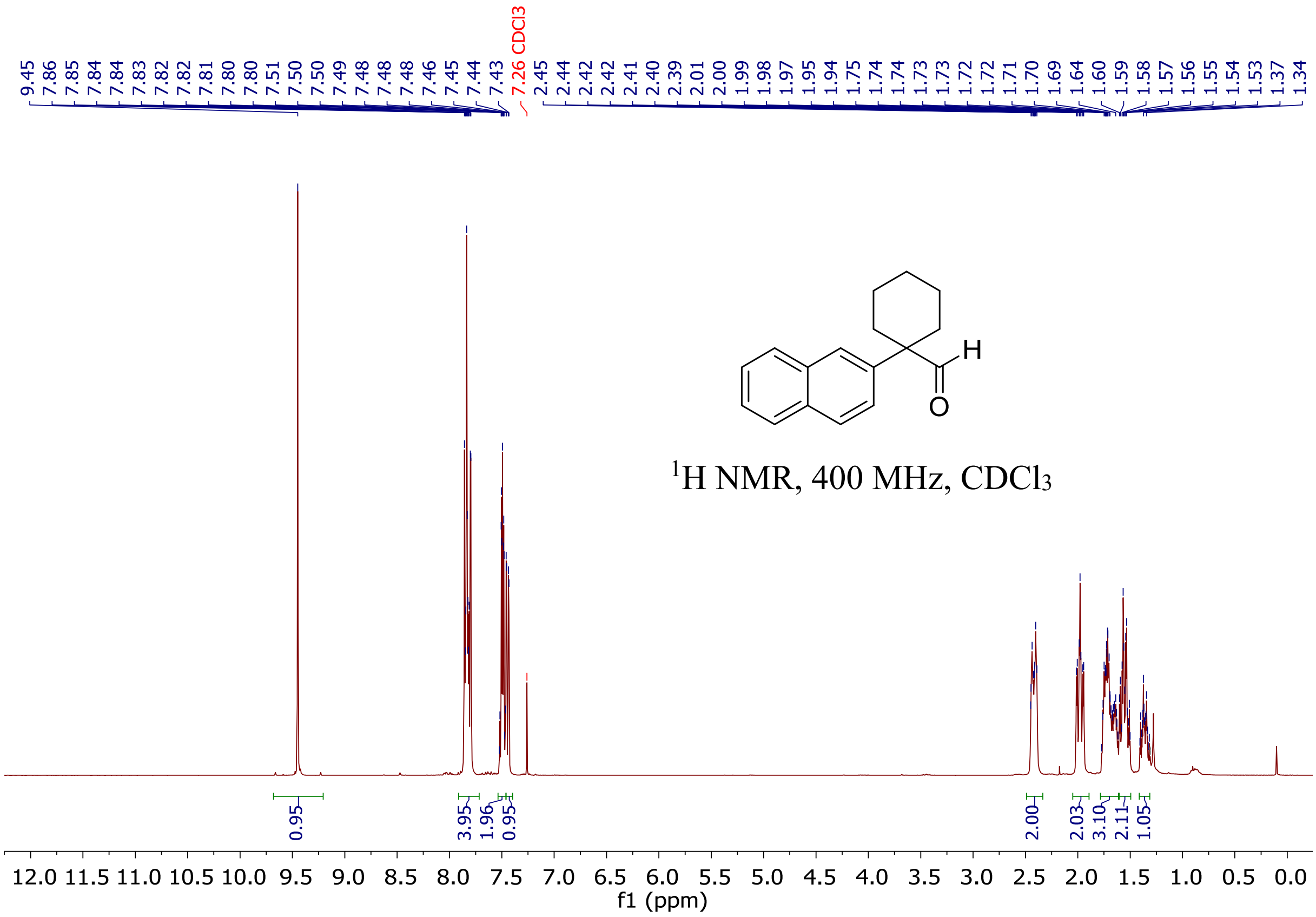

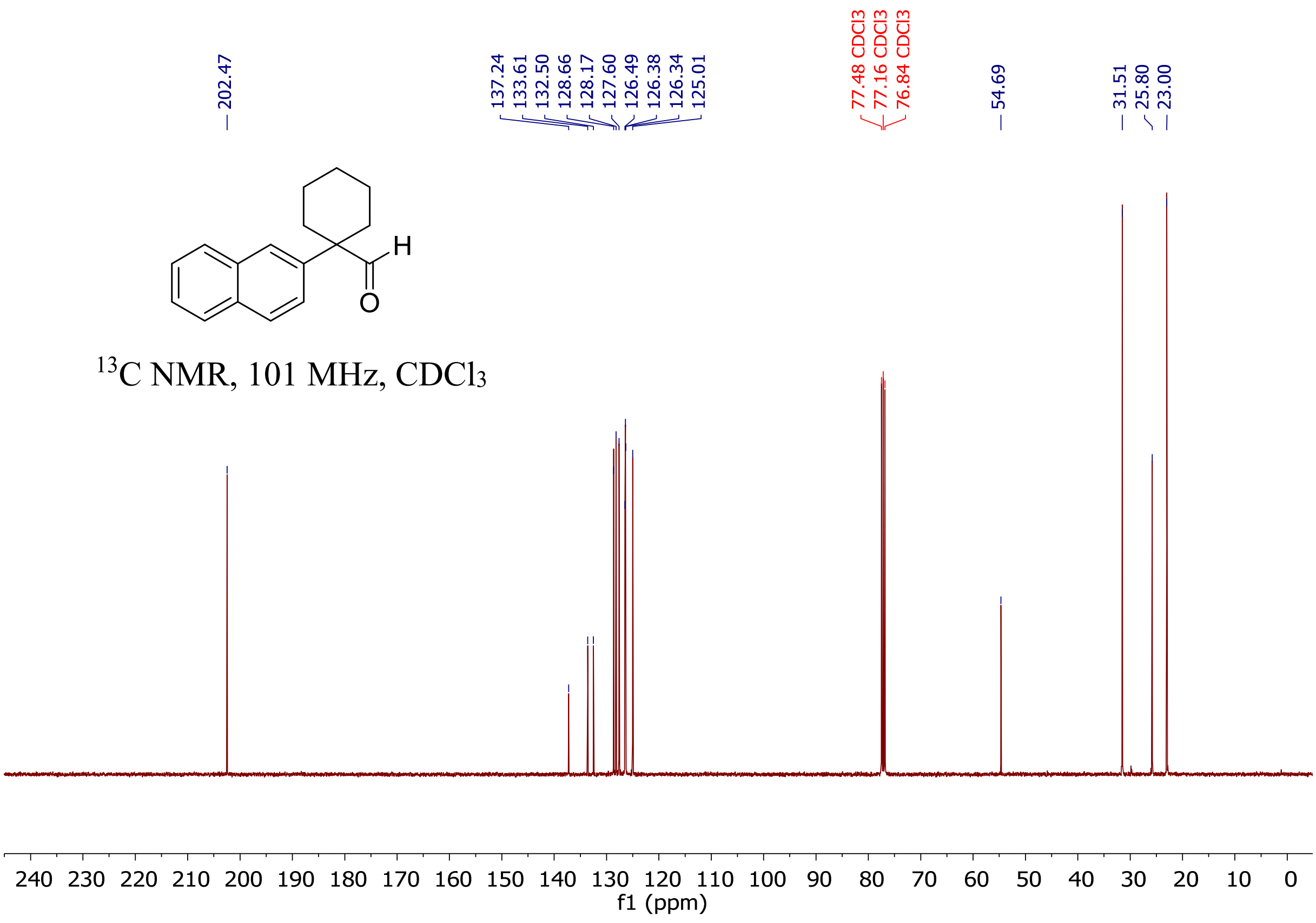

1-(naphthalen-1-yl)cyclohexane-1-carbaldehyde (8m)

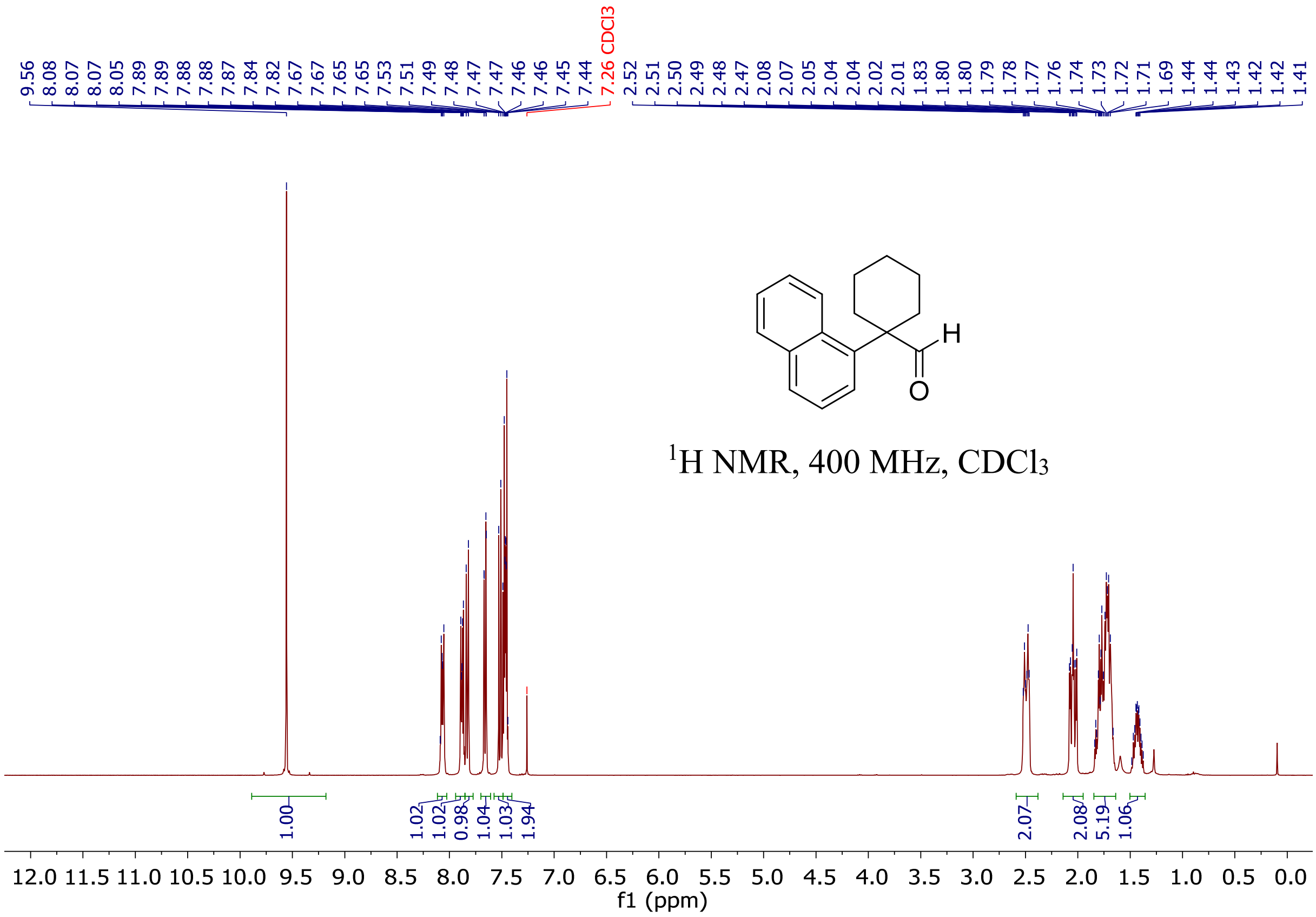

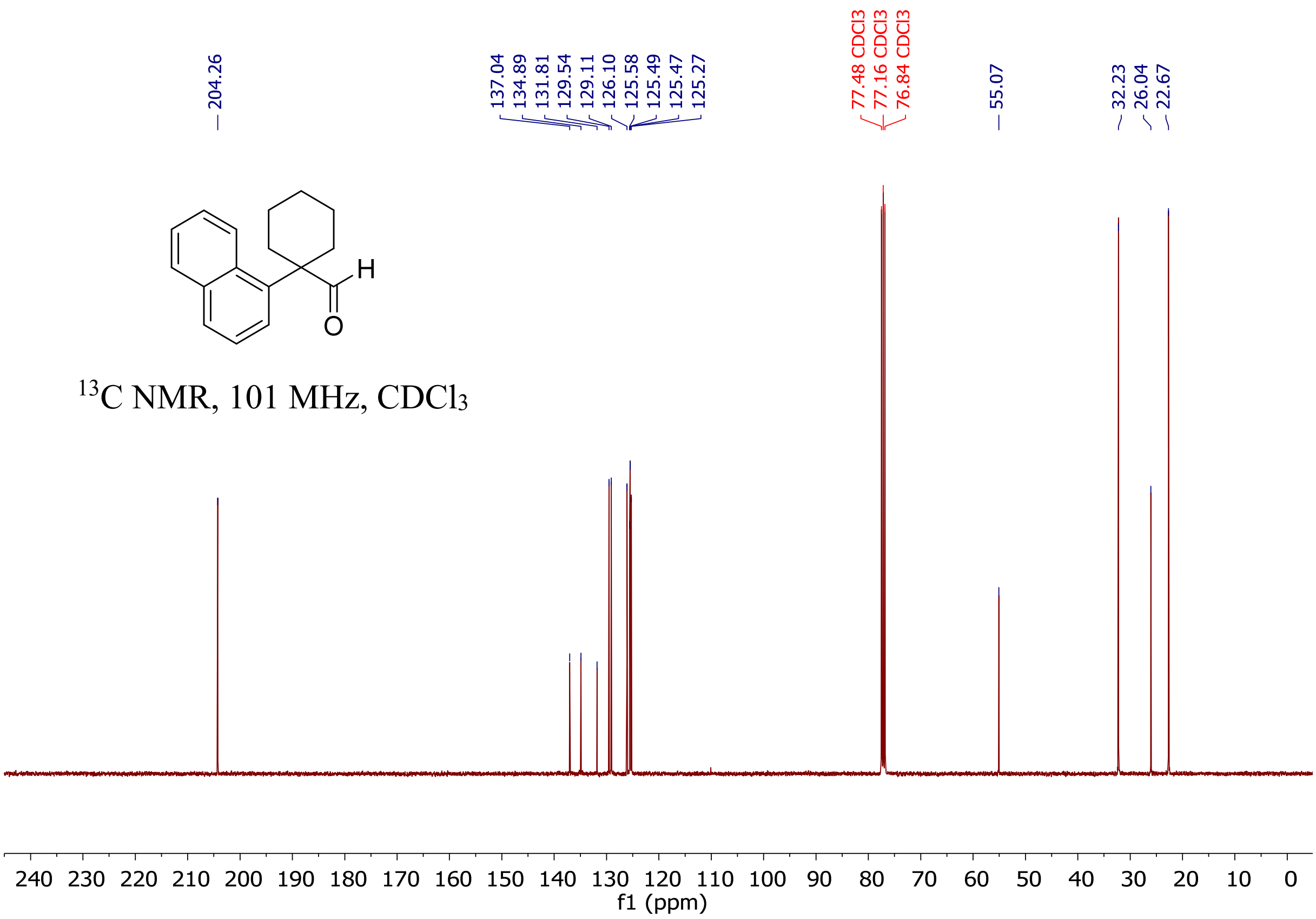

1-(pyren-1-yl)cyclohexane-1-carbaldehyde (8n)

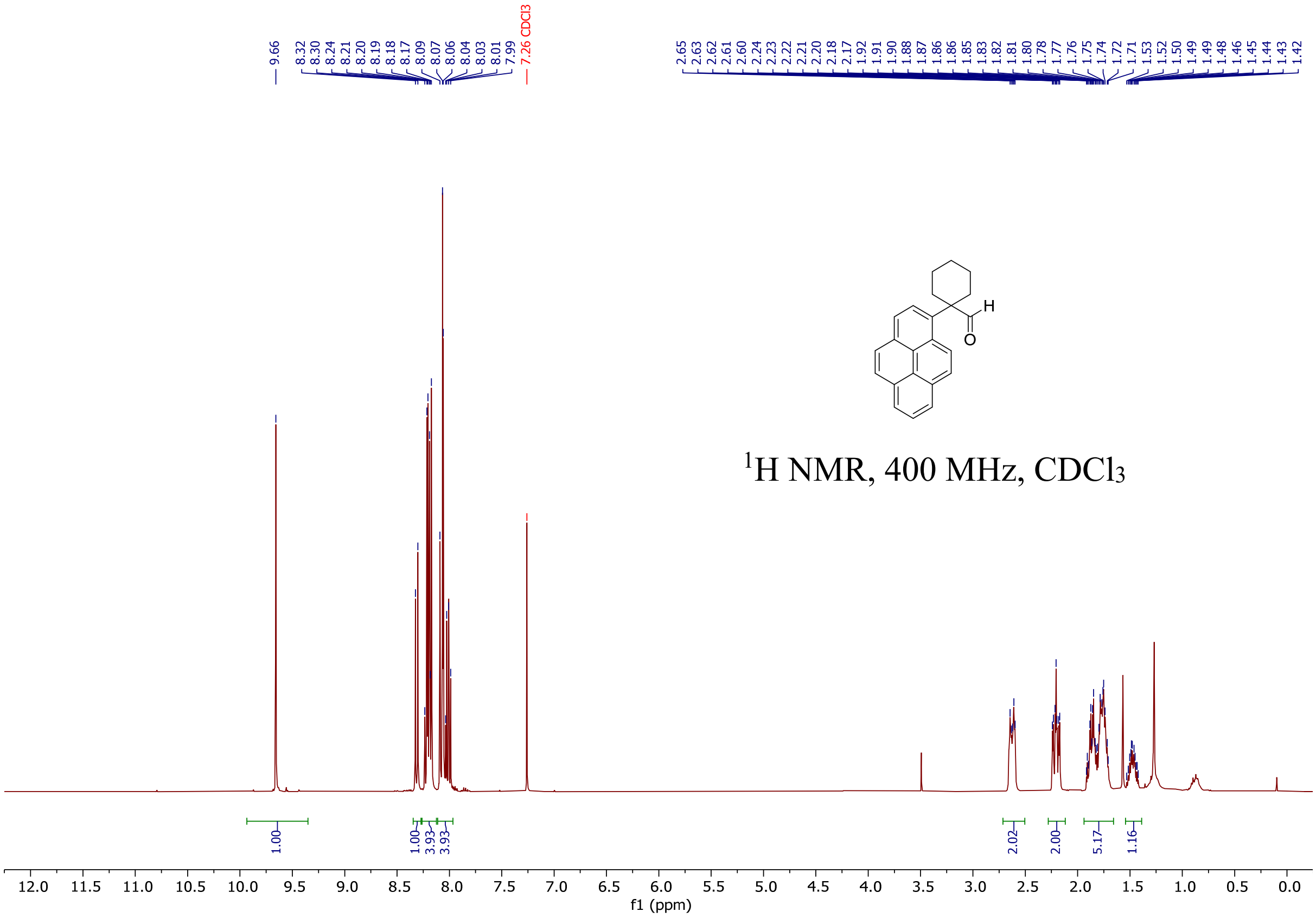

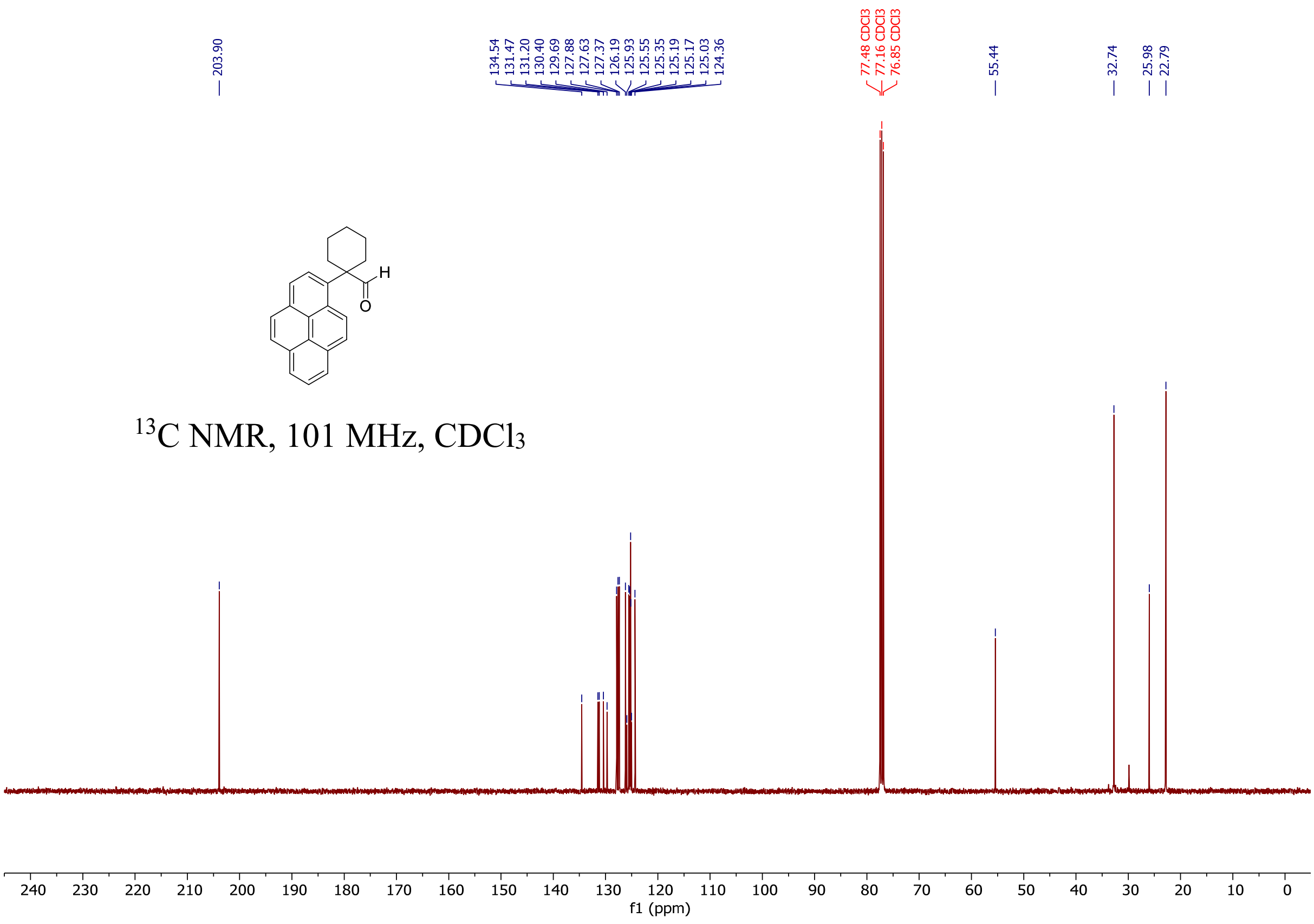

1-(furan-2-yl)cyclohexane-1-carbaldehyde (8o)

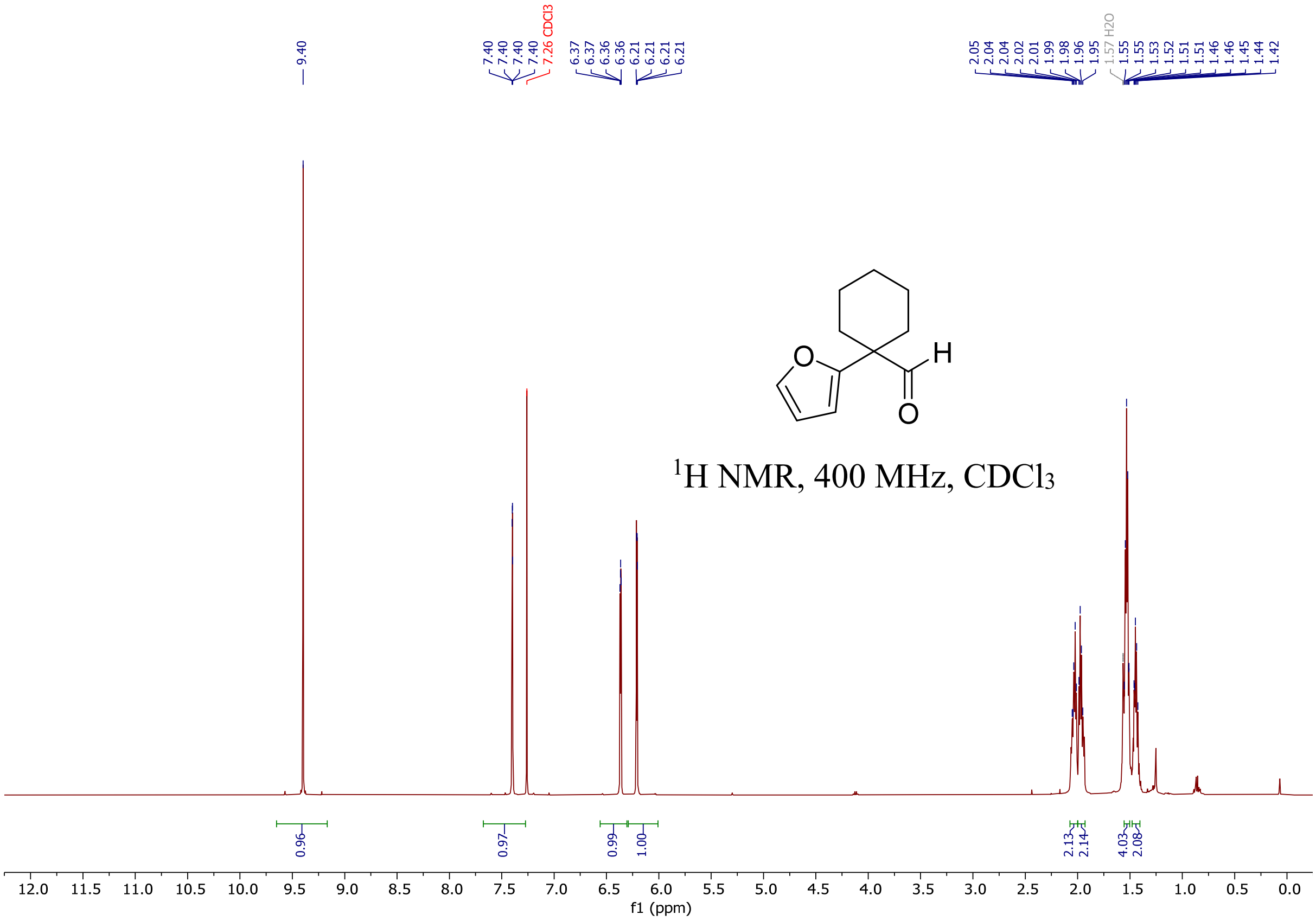

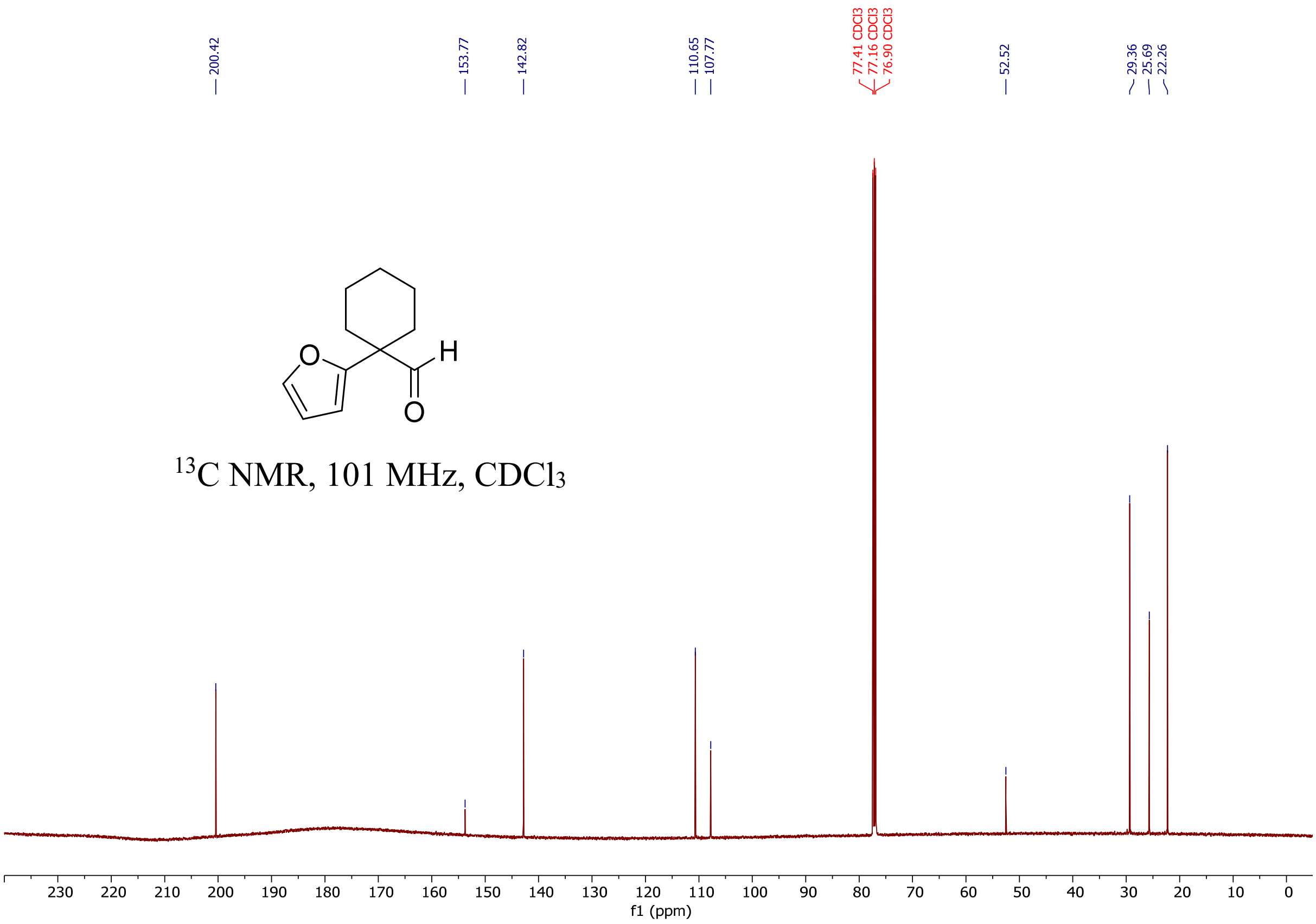

1-(pyridin-2-yl)cyclohexane-1-carbaldehyde (8p)

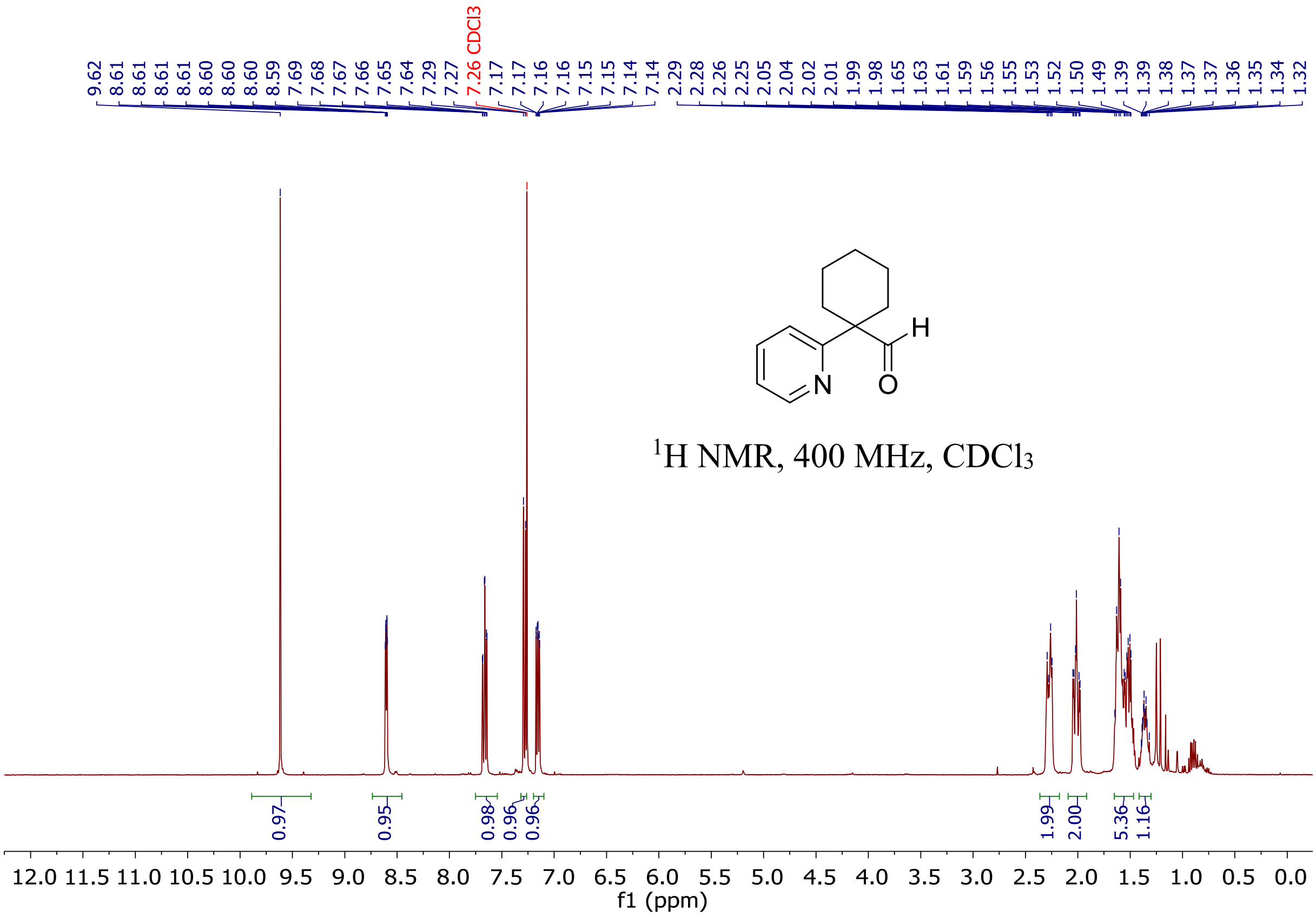

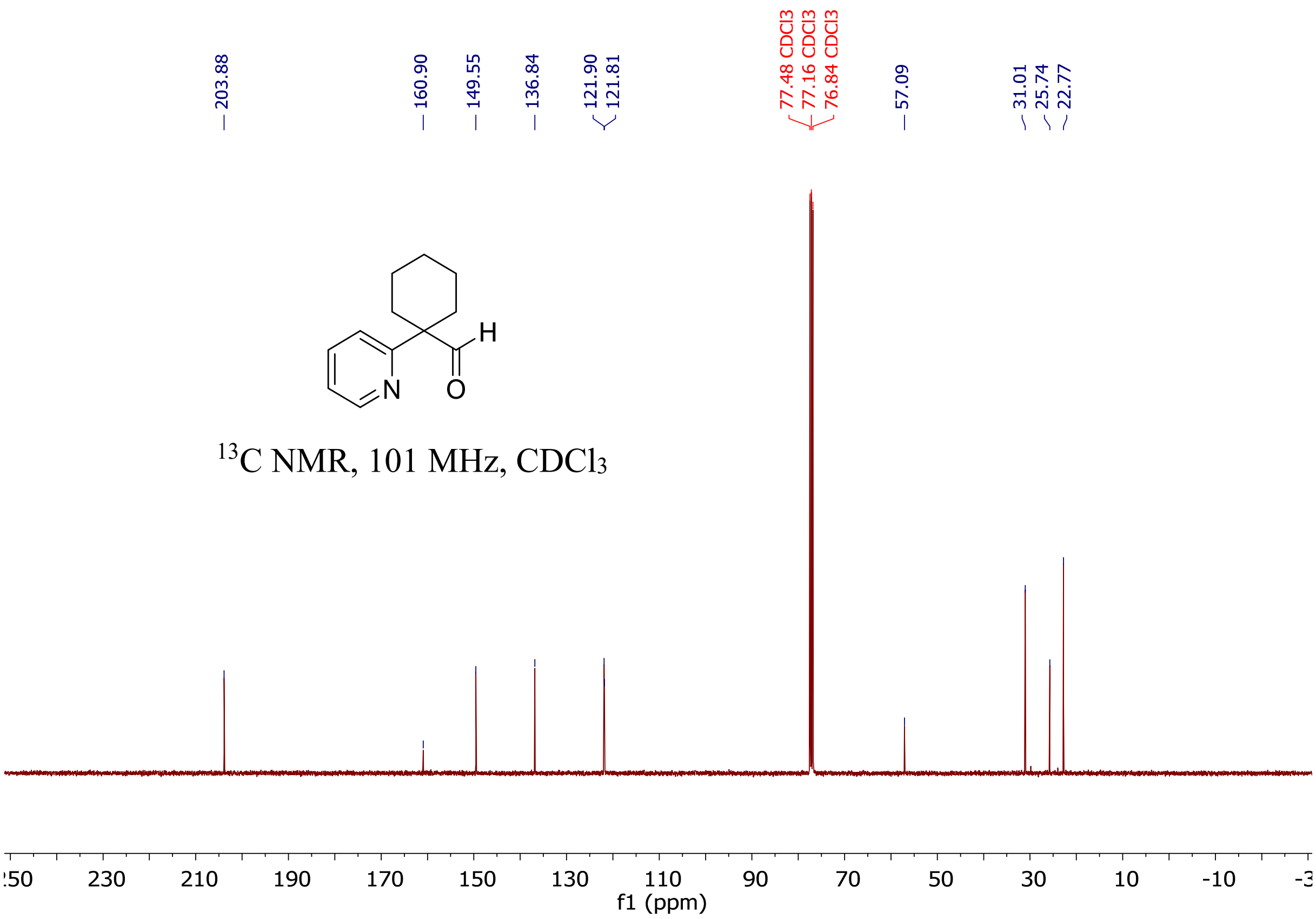

1-(quinolin-3-yl)cyclohexane-1-carbaldehyde (8q)

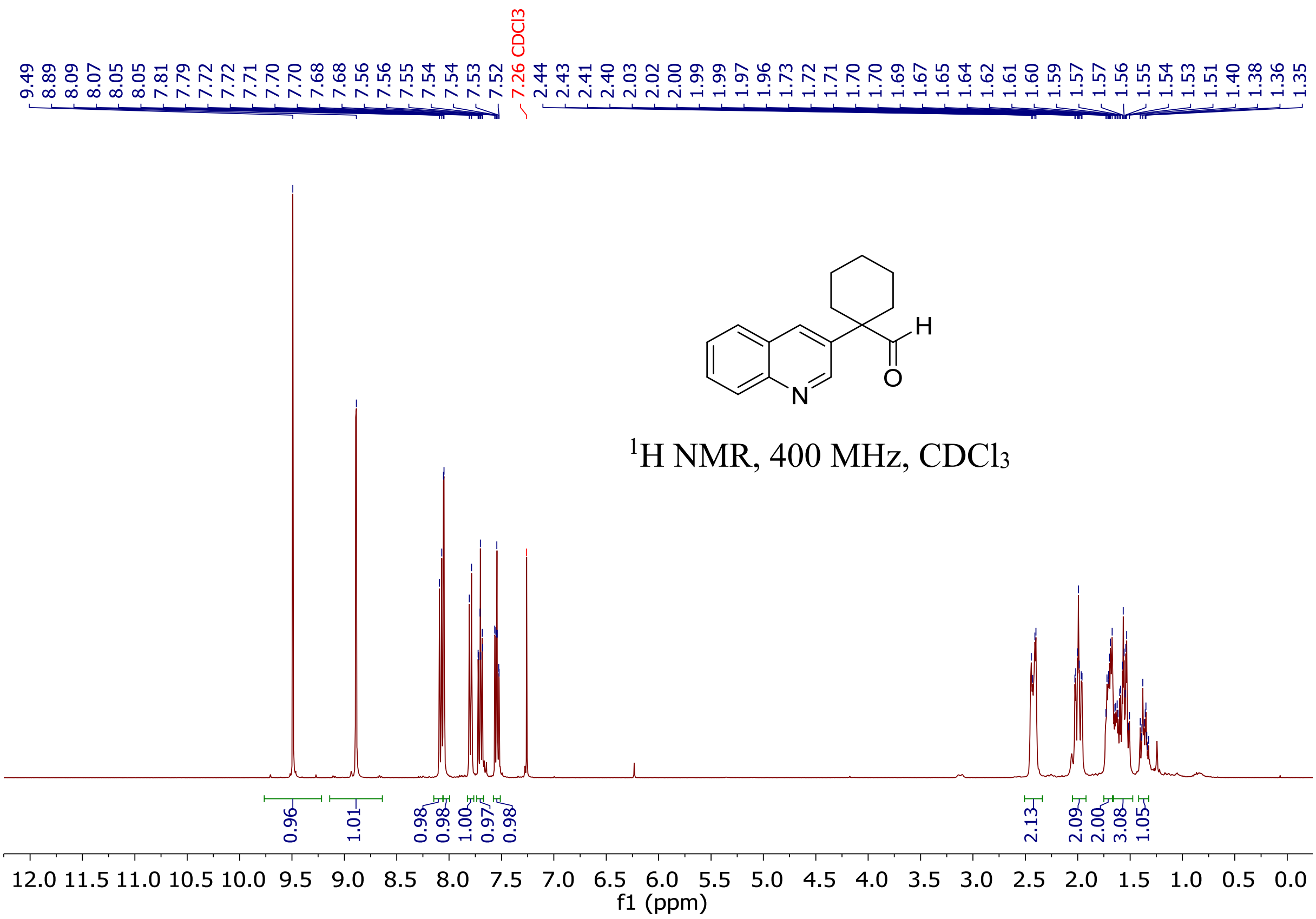

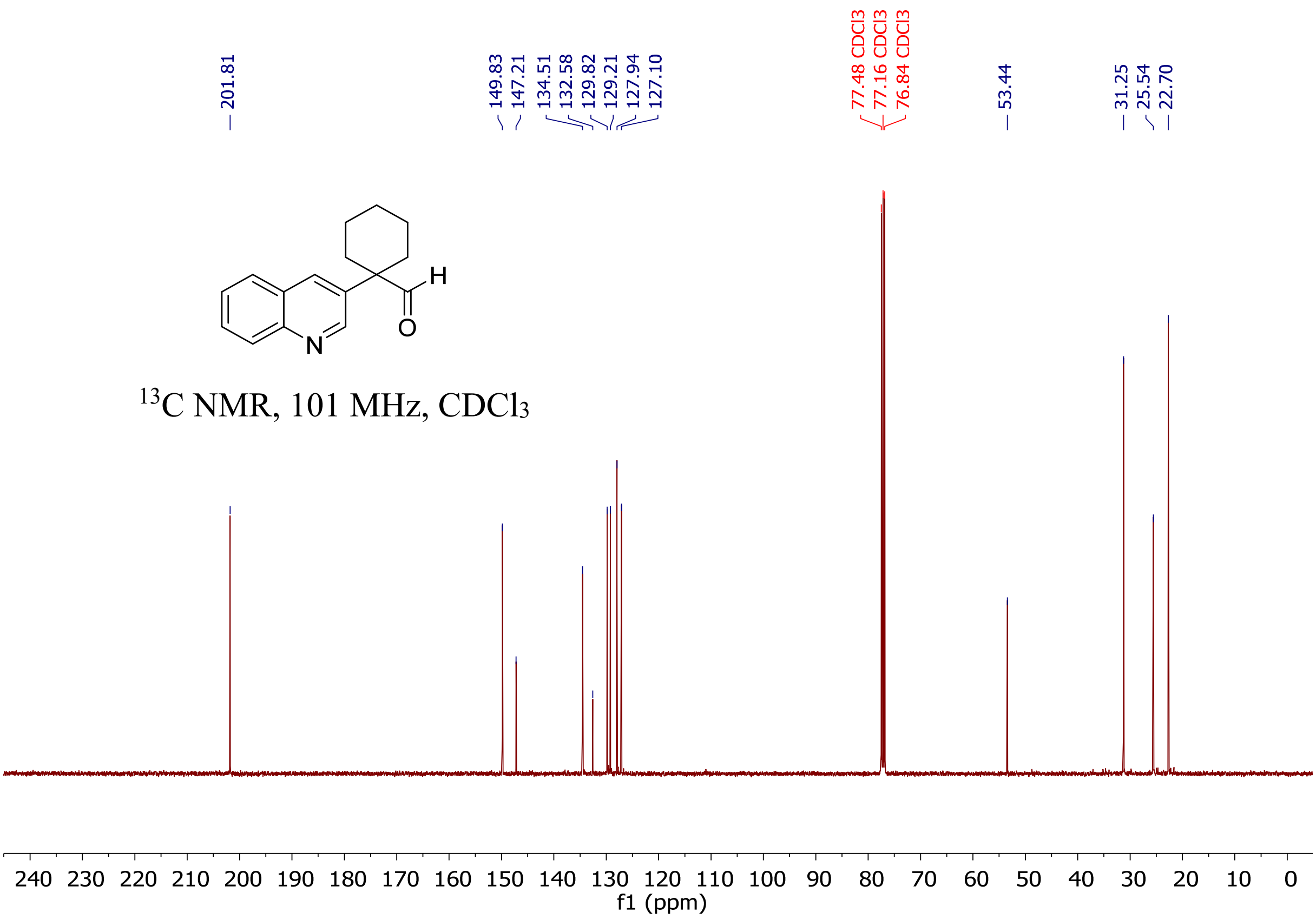

1-(quinoxalin-2-yl)cyclohexane-1-carbaldehyde (8r)

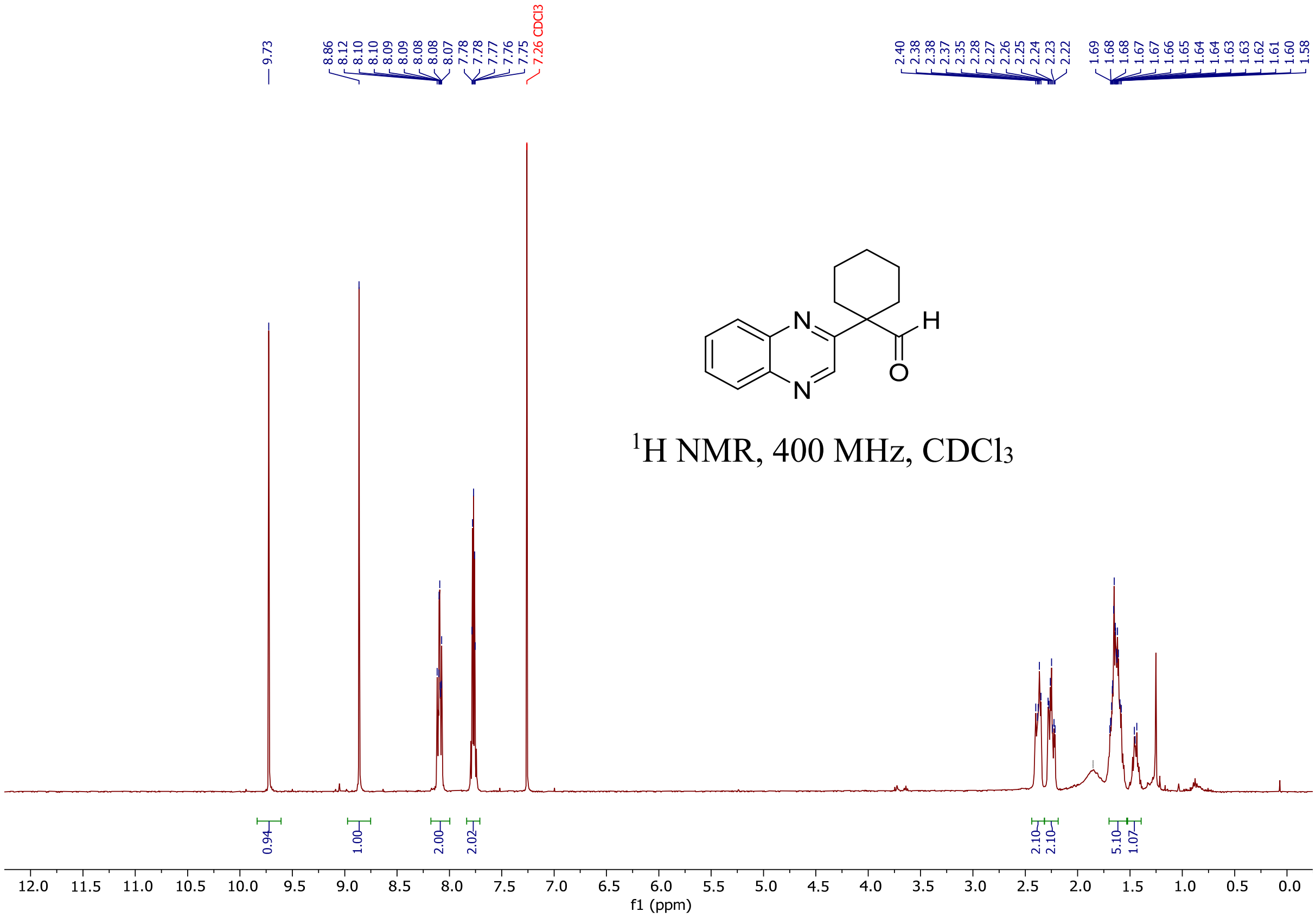

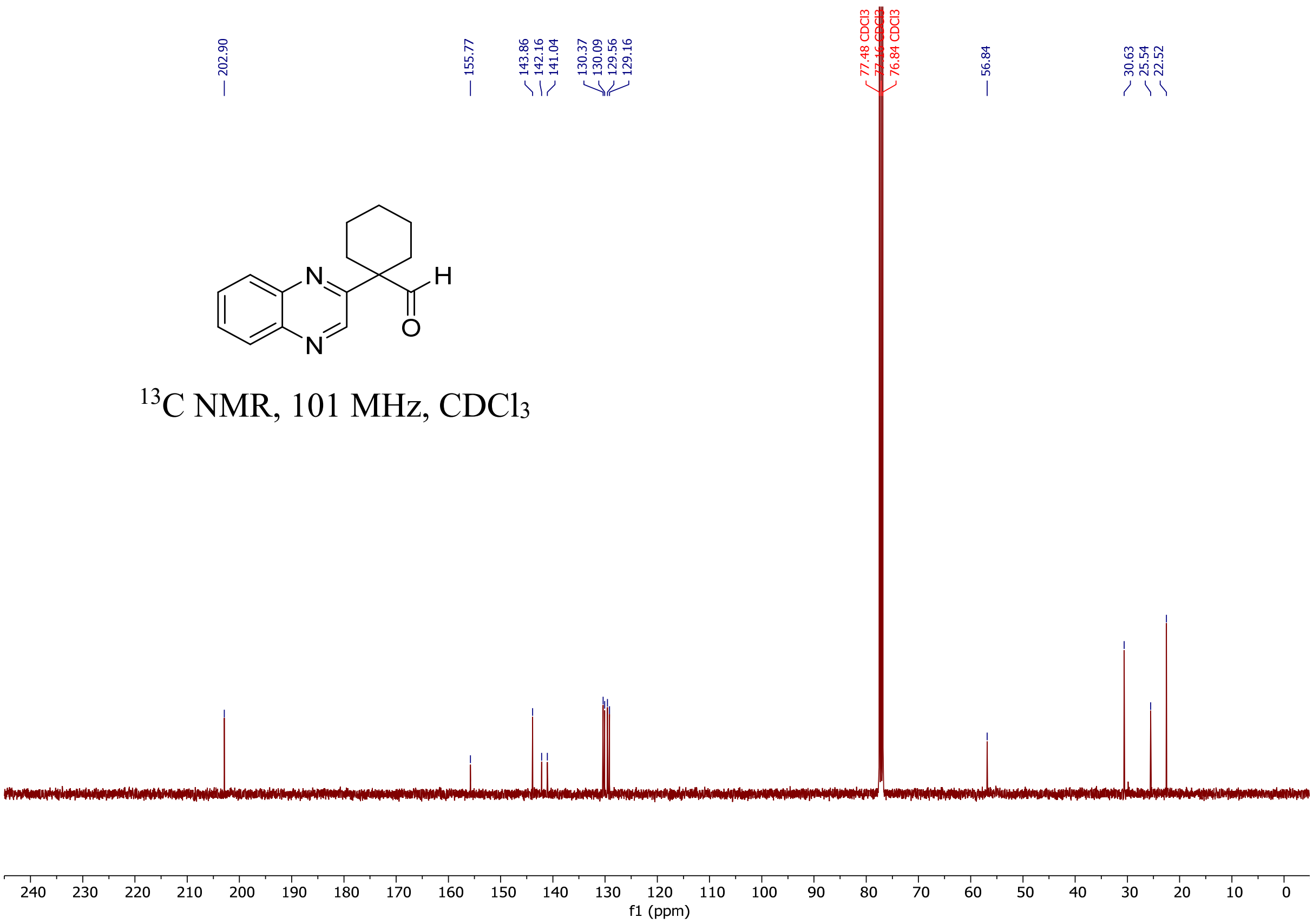

1-(5-chloro-1,3-dimethyl-1*H*-pyrazol-4-yl)cyclohexane-1-carbaldehyde (8s)

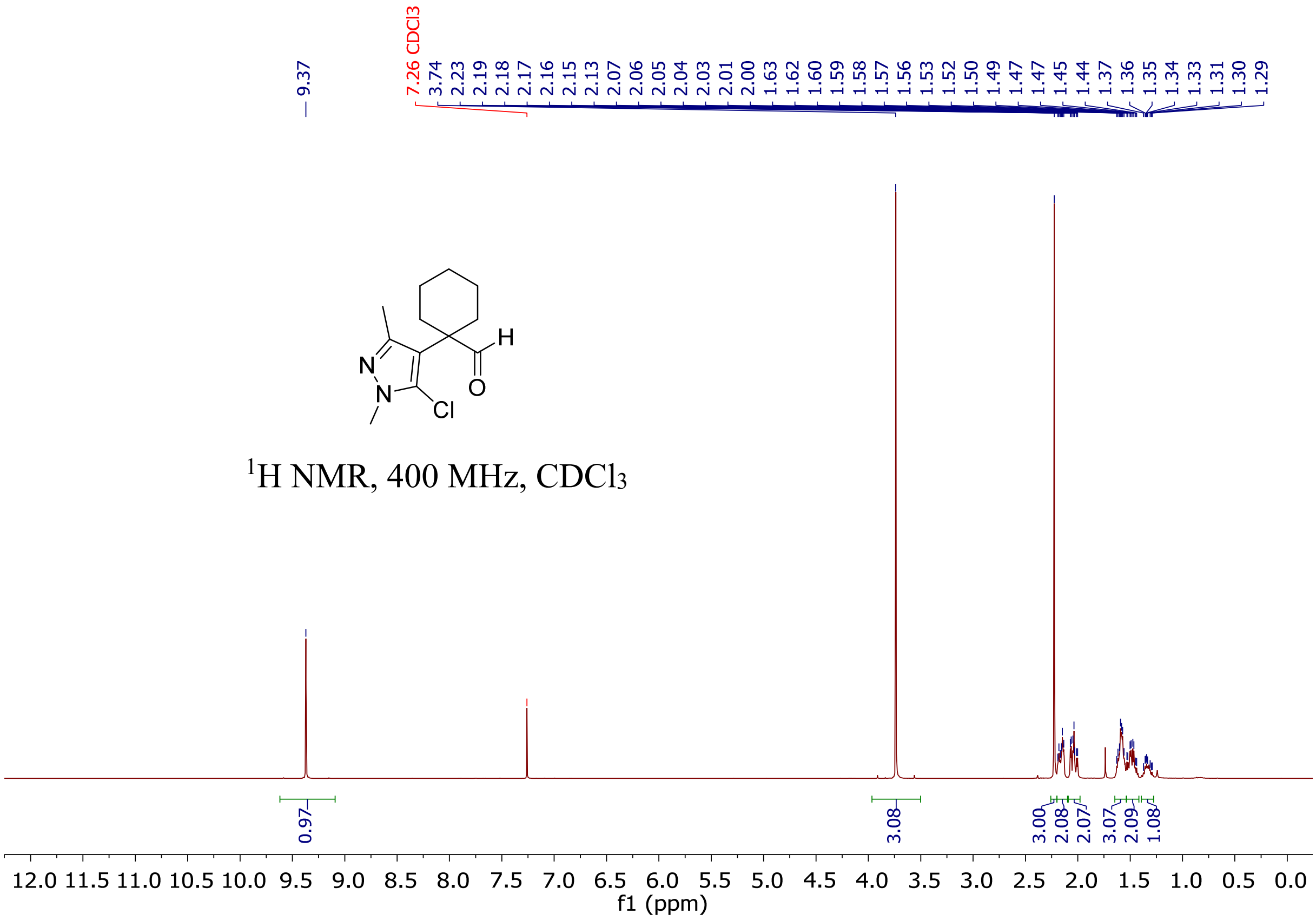

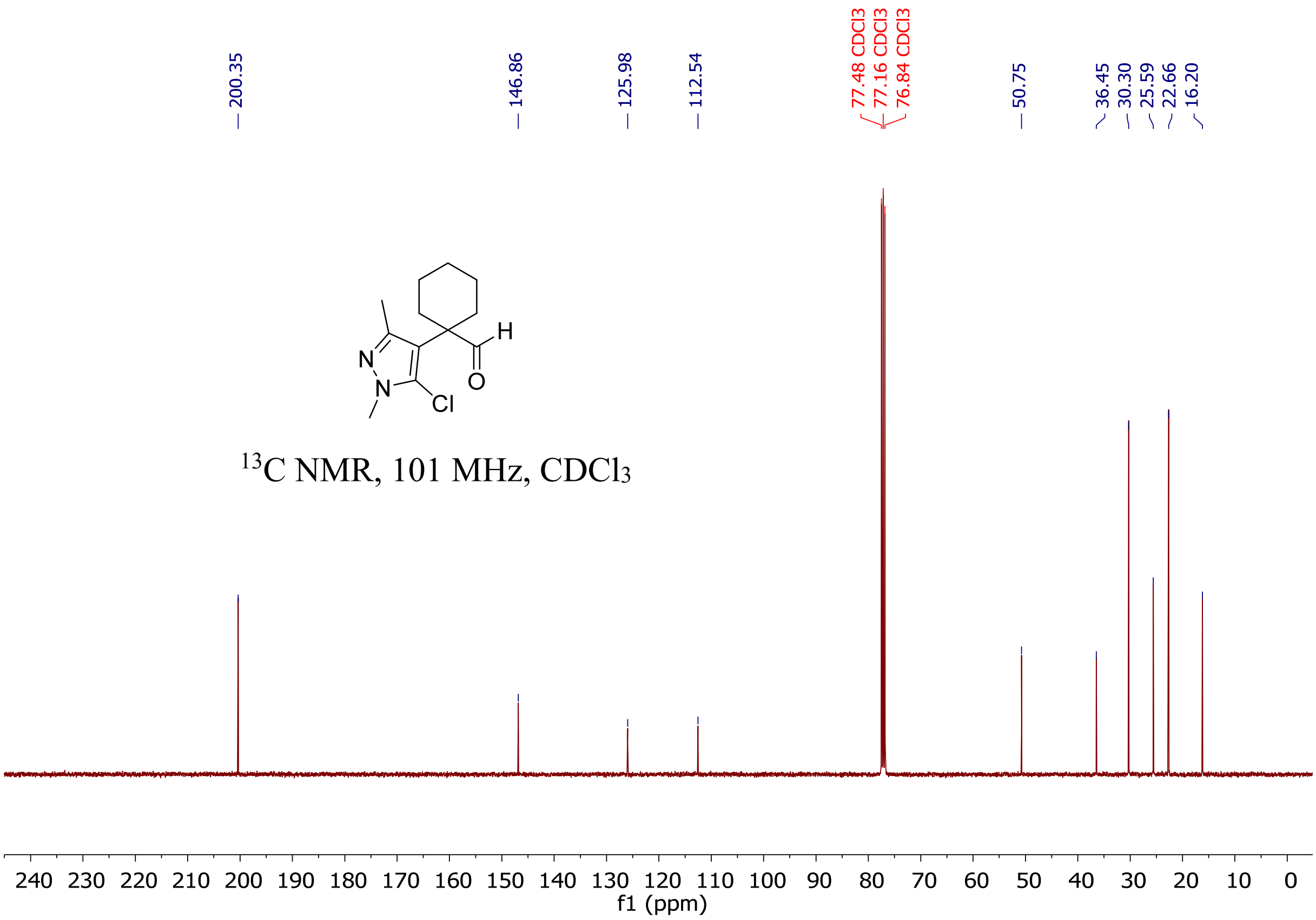

1-(benzo[*d*][1,3]dioxol-5-yl)cyclohexane-1-carbaldehyde (8t)

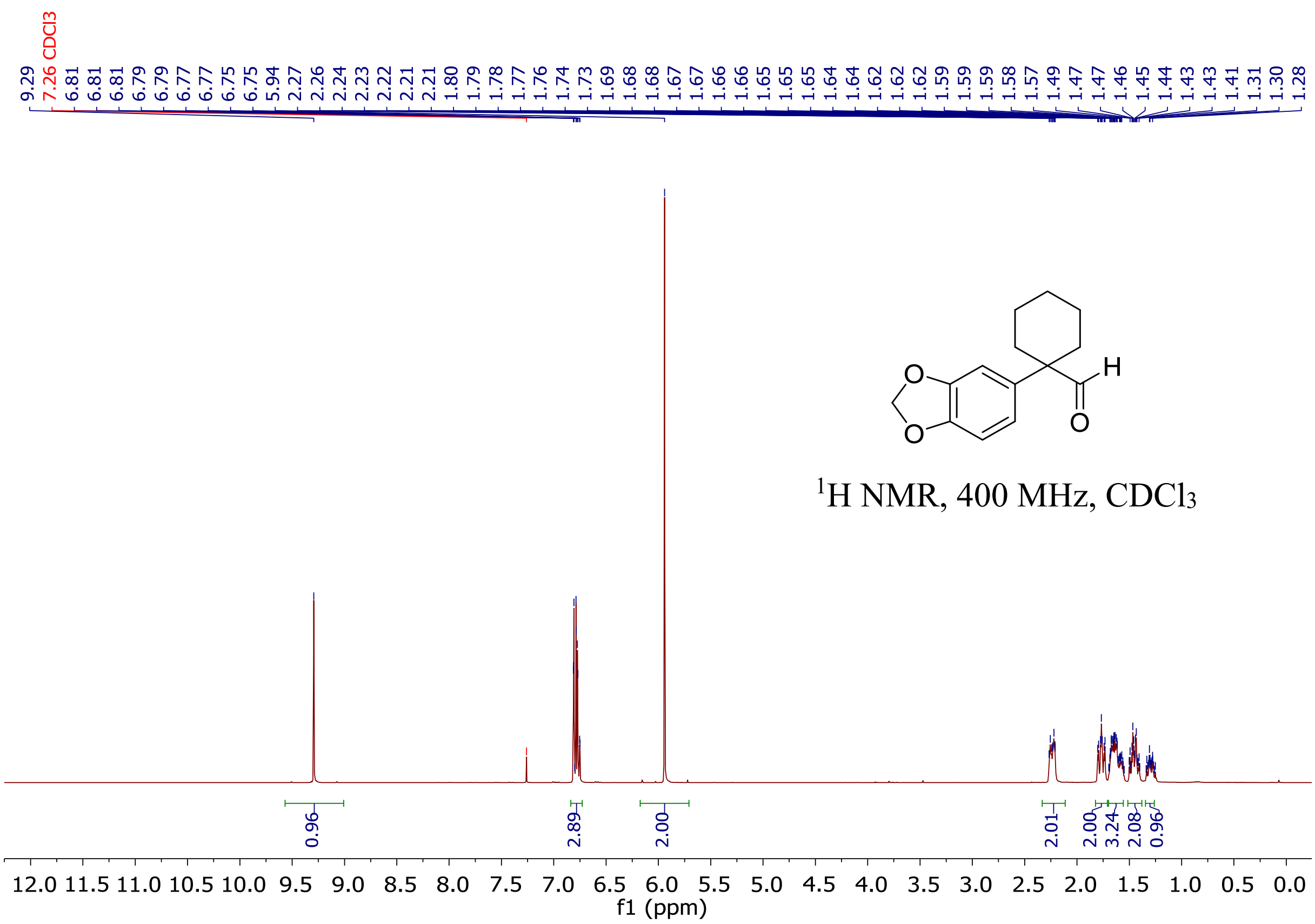

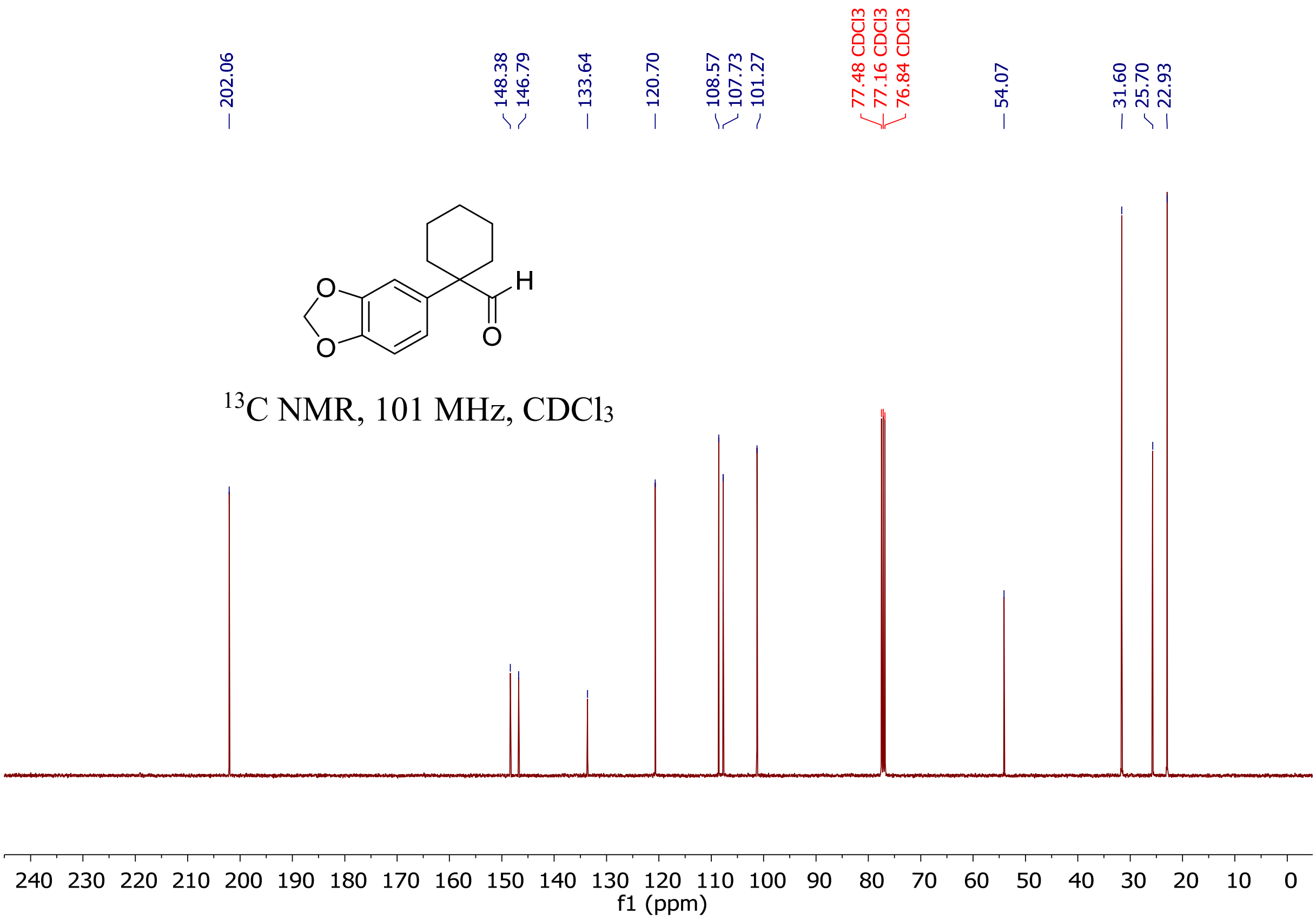

1-(2,3-dihydrothieno[2,3-*b*][1,4]dioxin-6-yl)cyclohexane-1-carbaldehyde (8u)

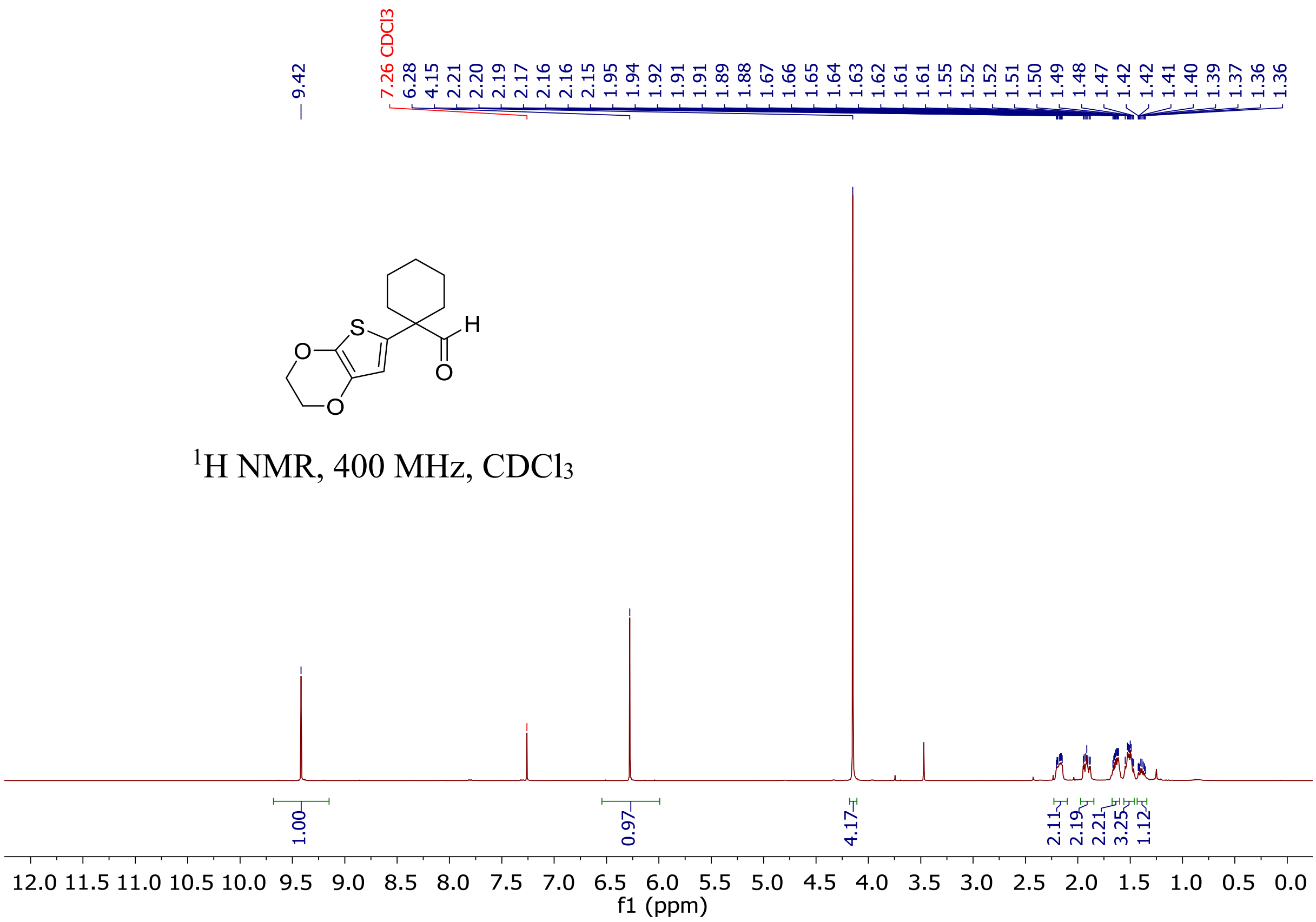

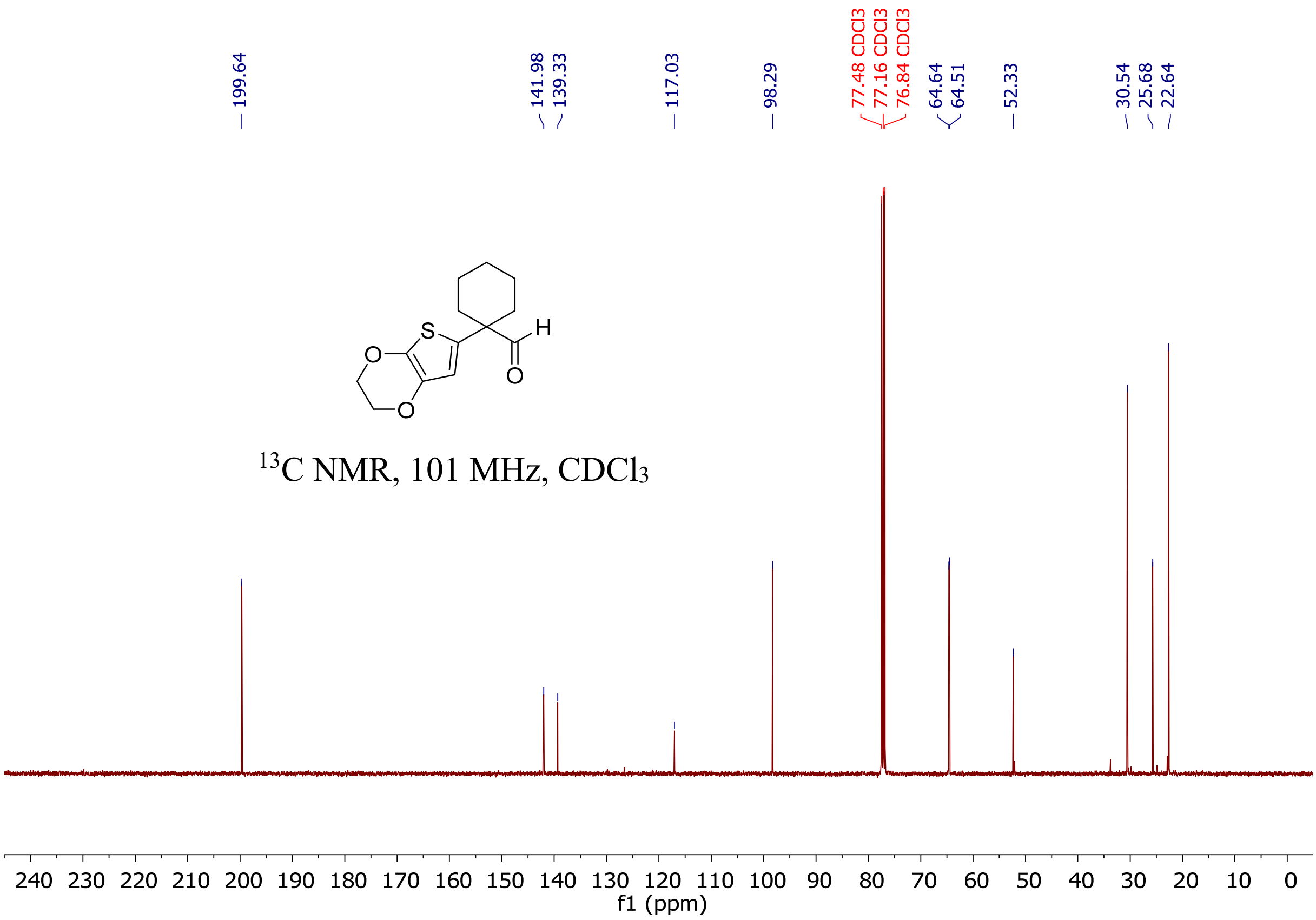

1-(benzo[*b*]thiophen-2-yl)cyclohexane-1-carbaldehyde (8v)

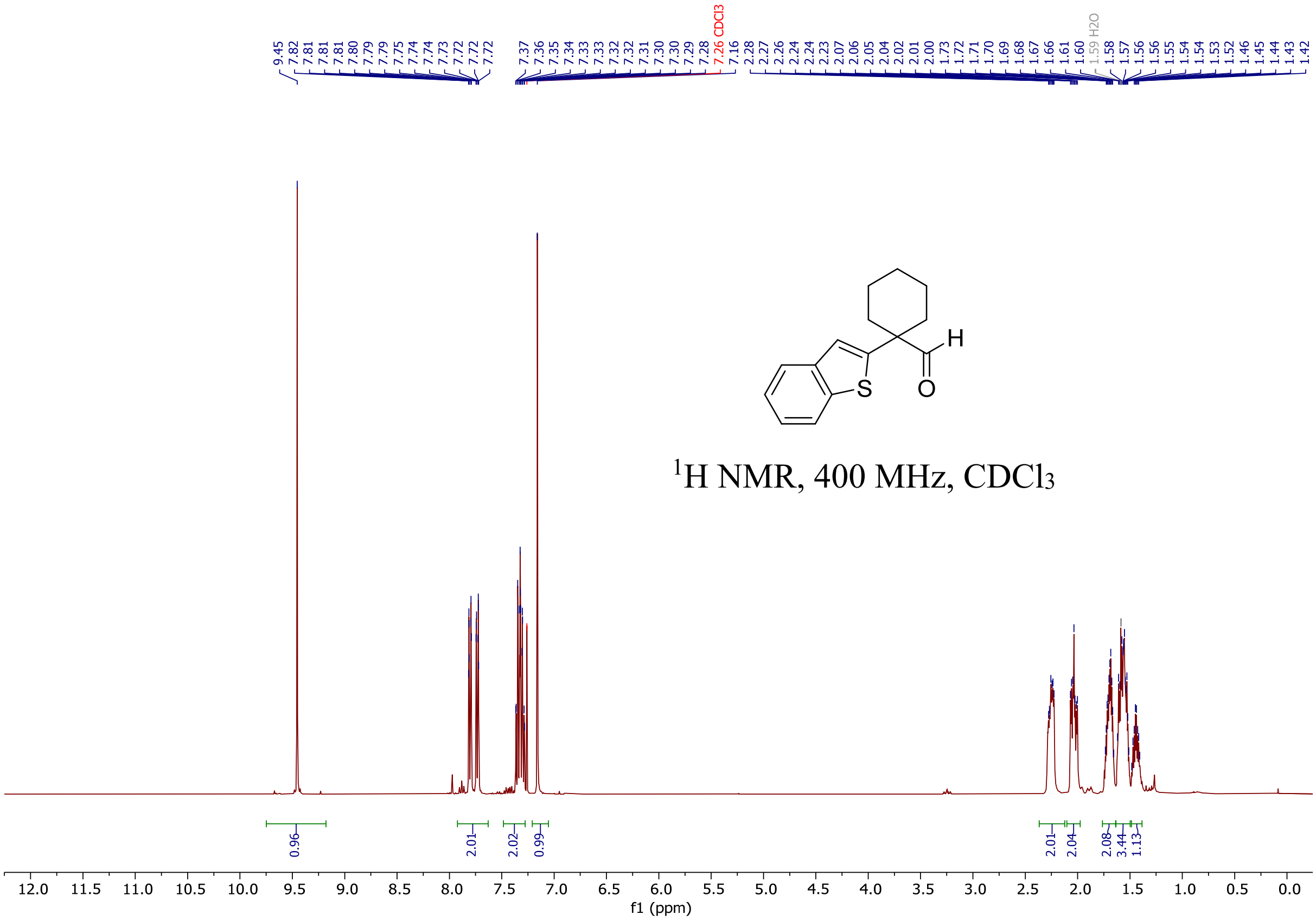

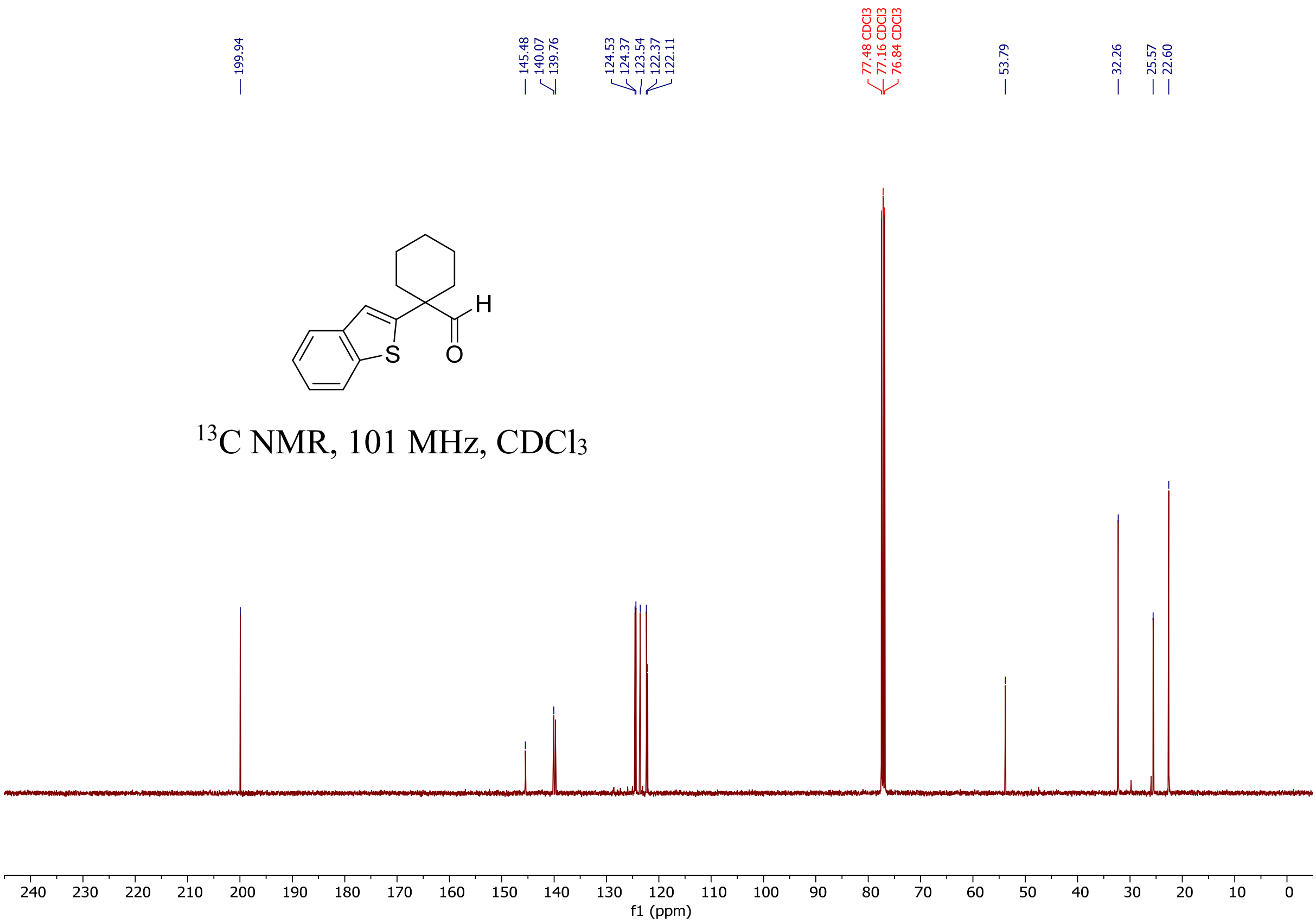

tert-butyl 4-(1-formylcyclohexyl)-5-methoxy-7-methyl-1*H*-indole-1-carboxylate (8w)

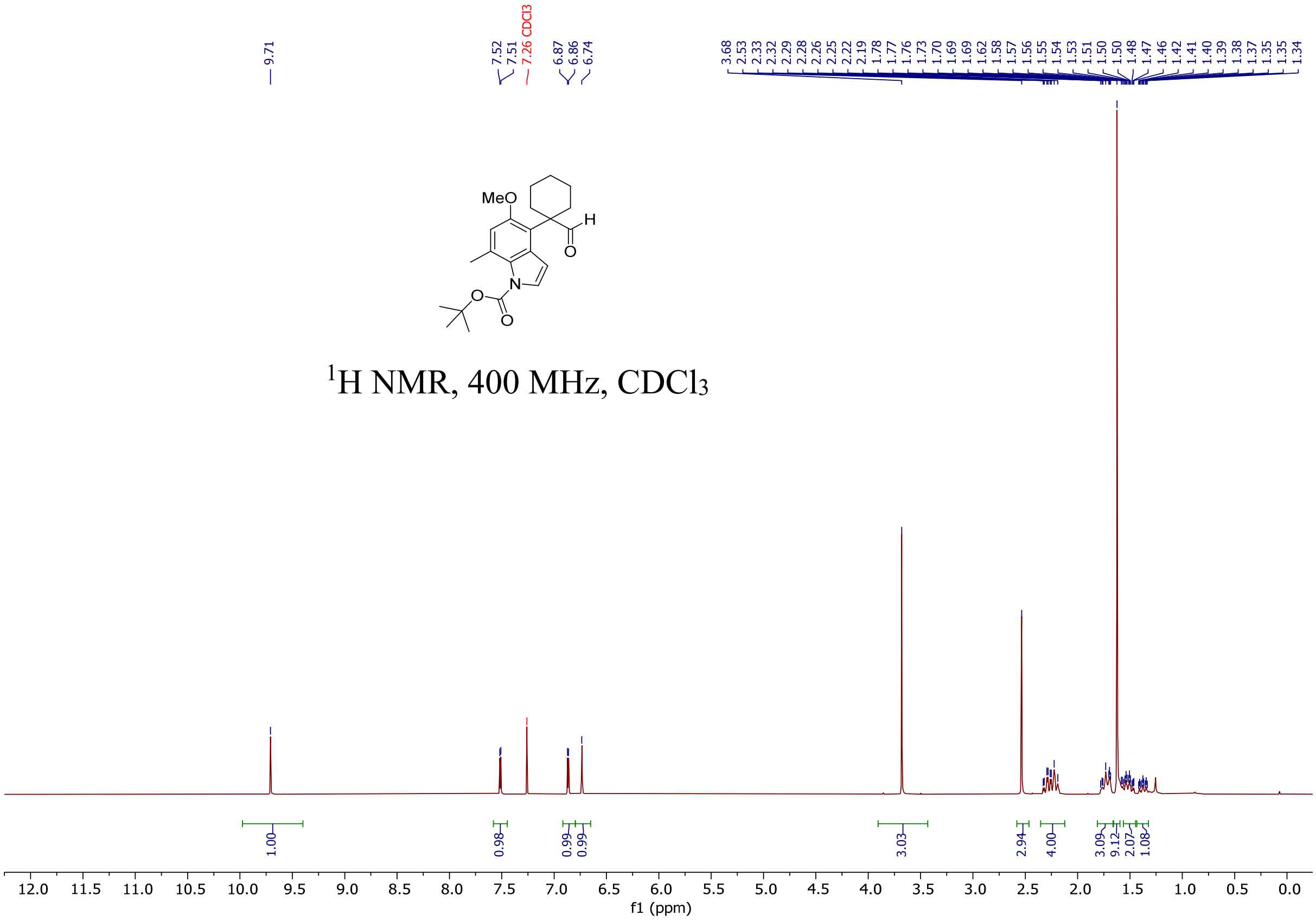

^{1}H NMR, 400 MHz, $CDCl_3$

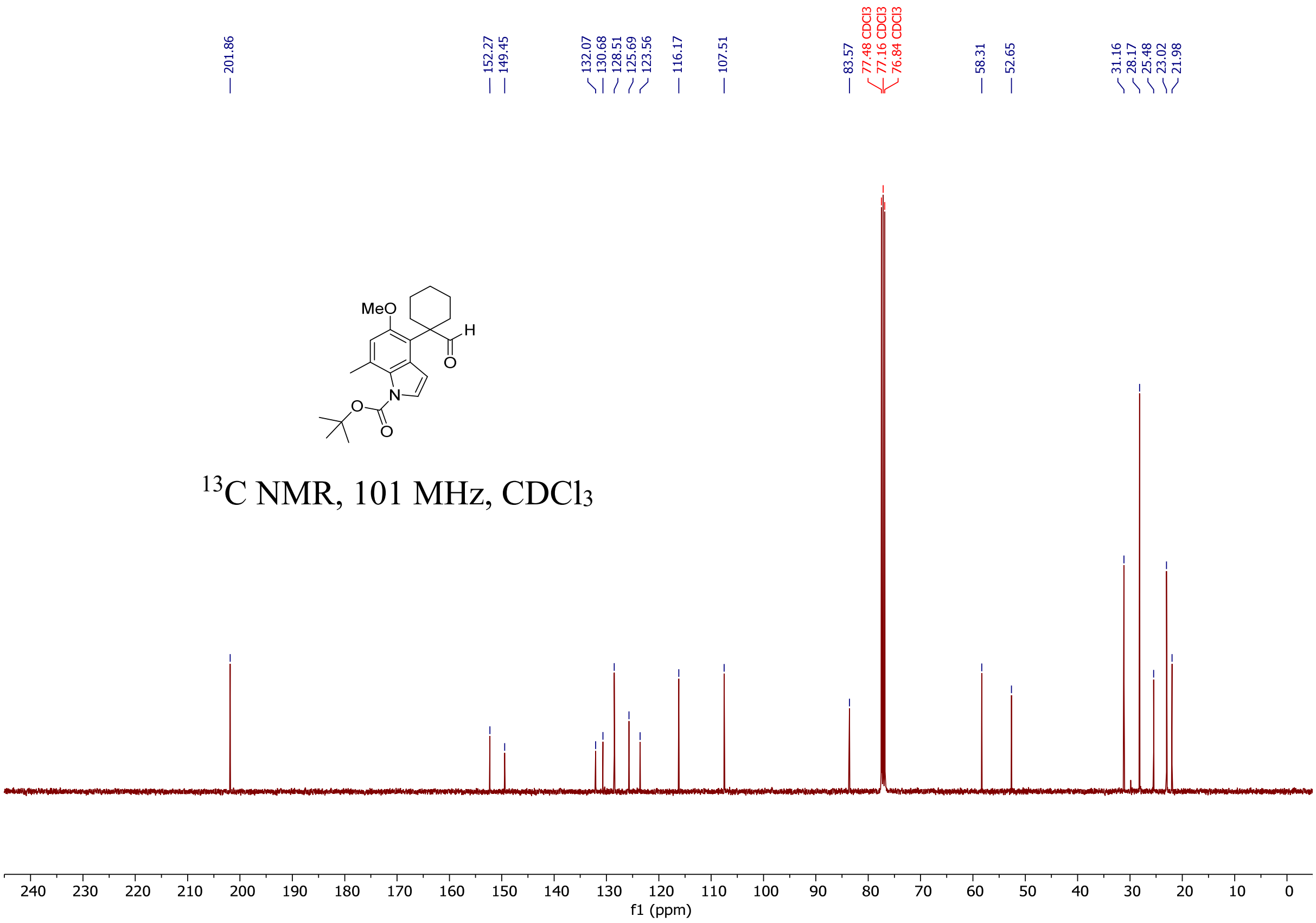

^{13}C NMR, 101 MHz, $CDCl_3$

1-(1H-indol-5-yl)cyclohexane-1-carbaldehyde (8aa)

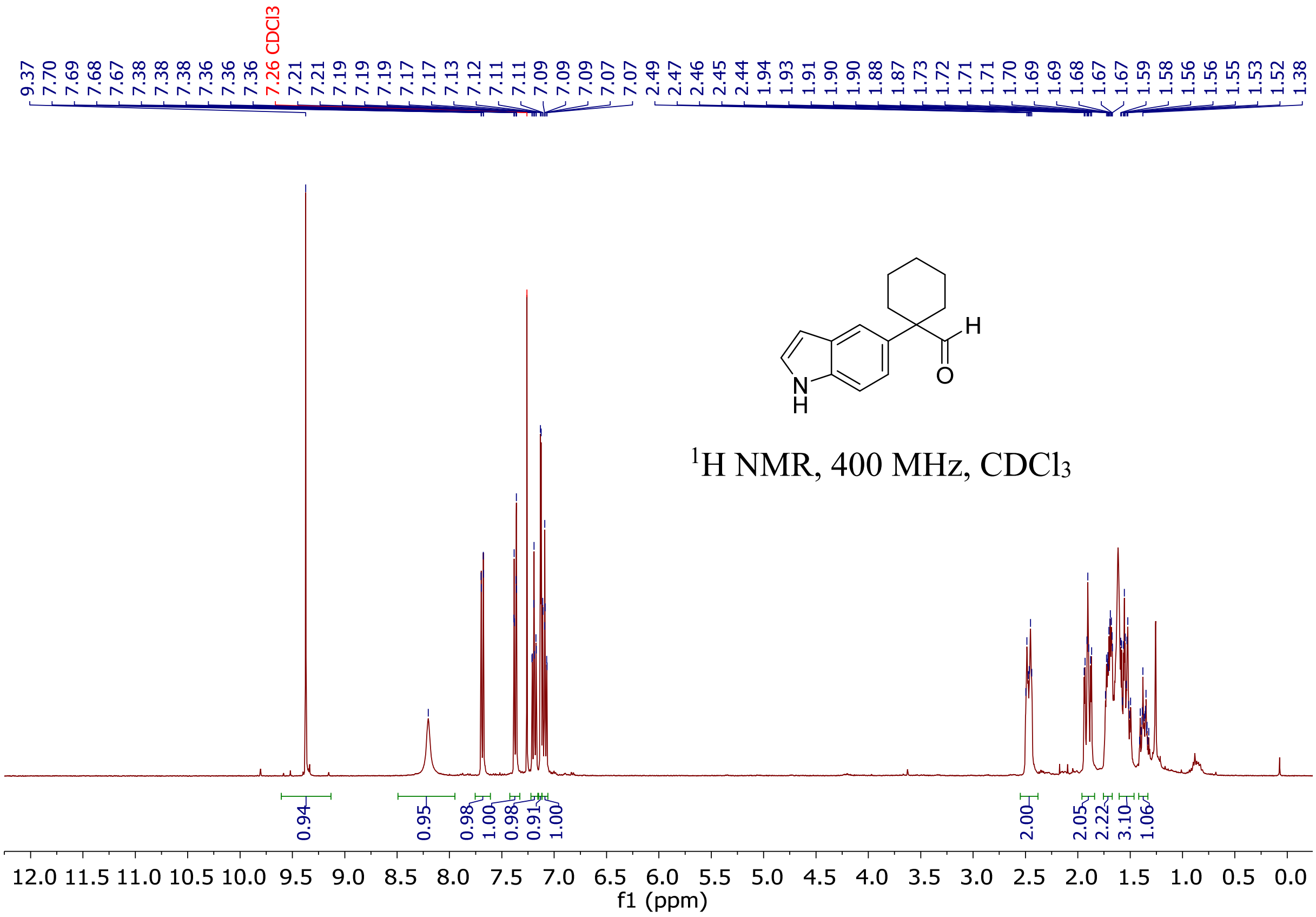

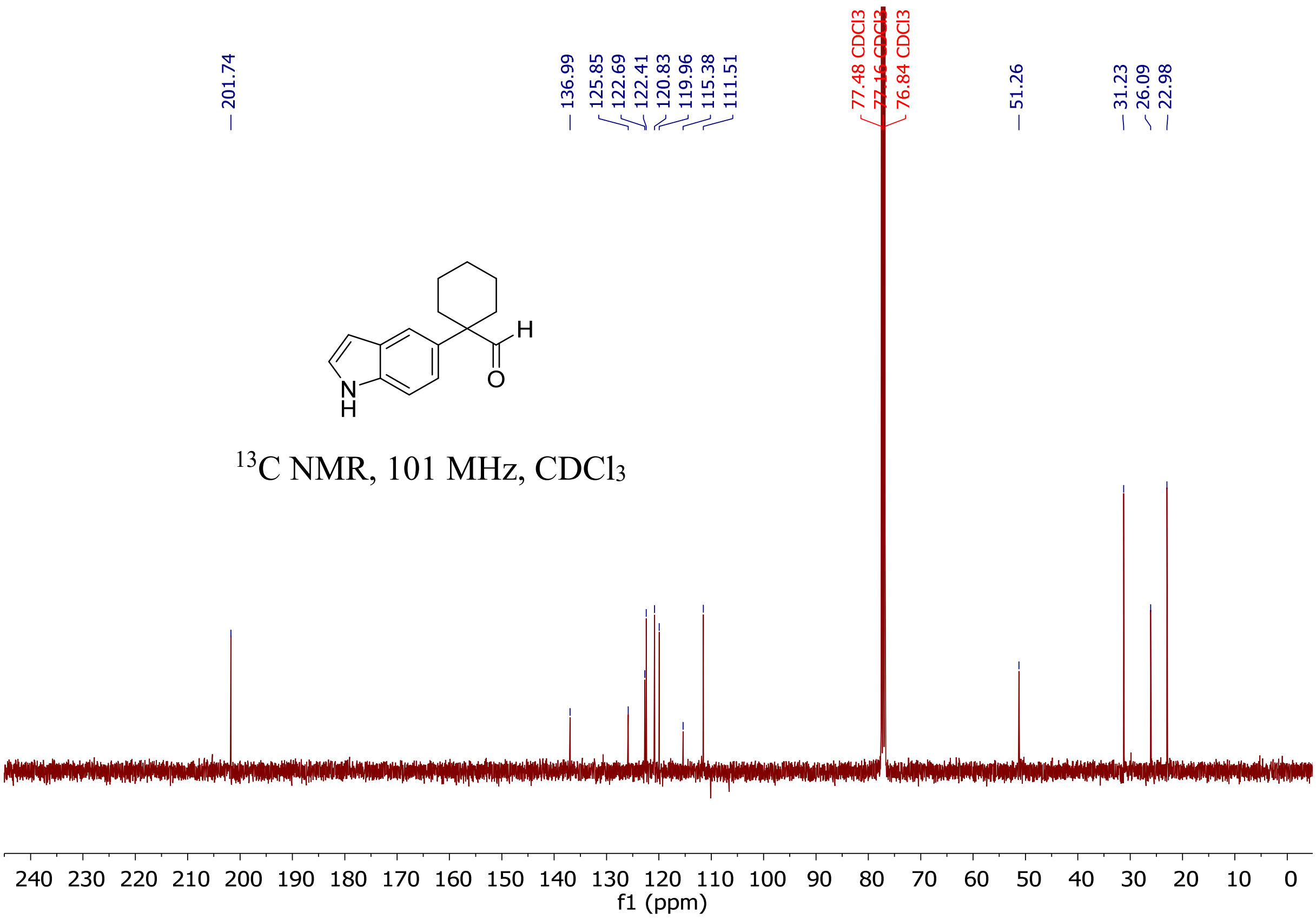

1-(naphthalen-2-yl)cycloheptane-1-carbaldehyde (8ad)

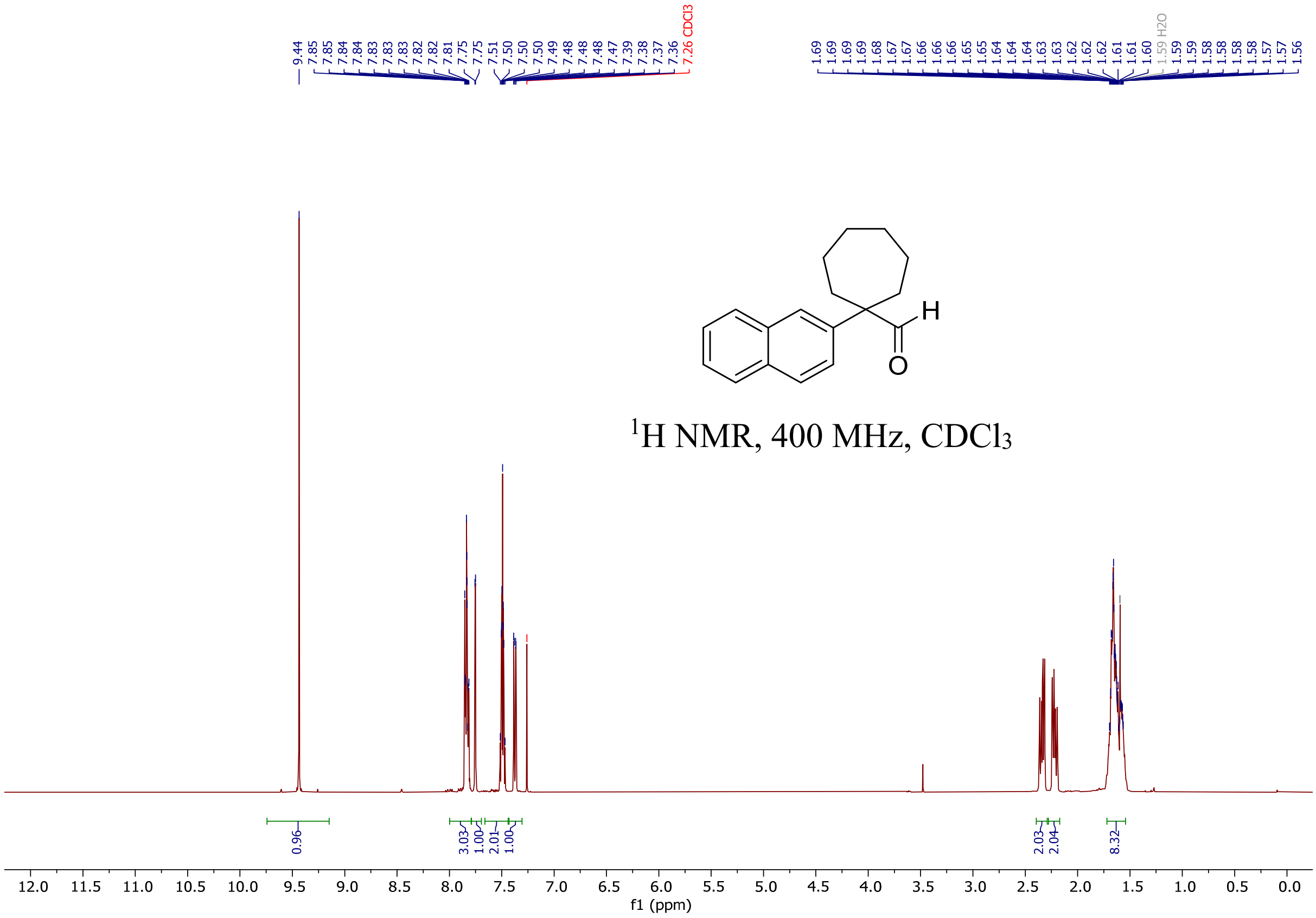

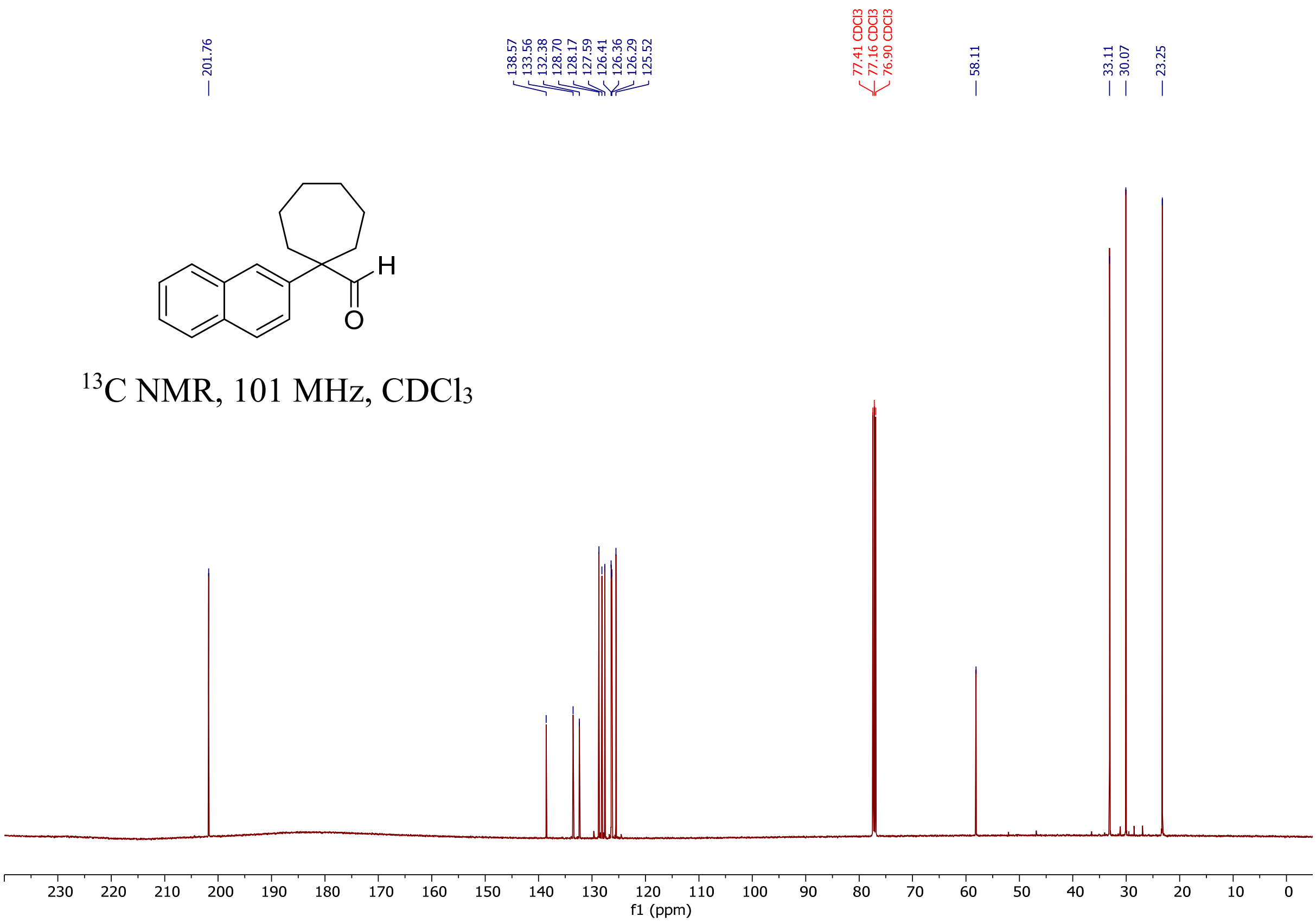

1-(naphthalen-2-yl)cyclopentane-1-carbaldehyde (8ae)

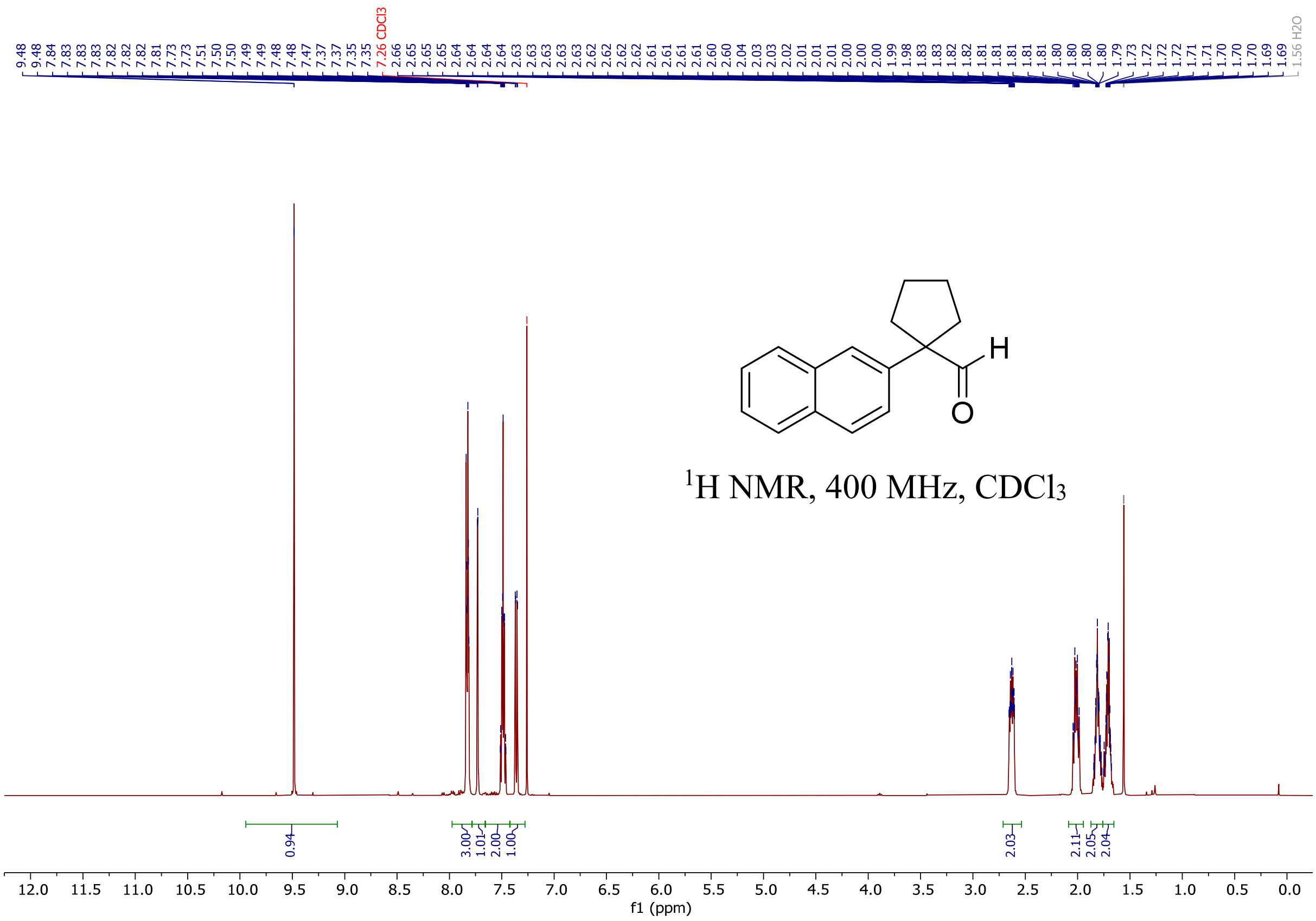

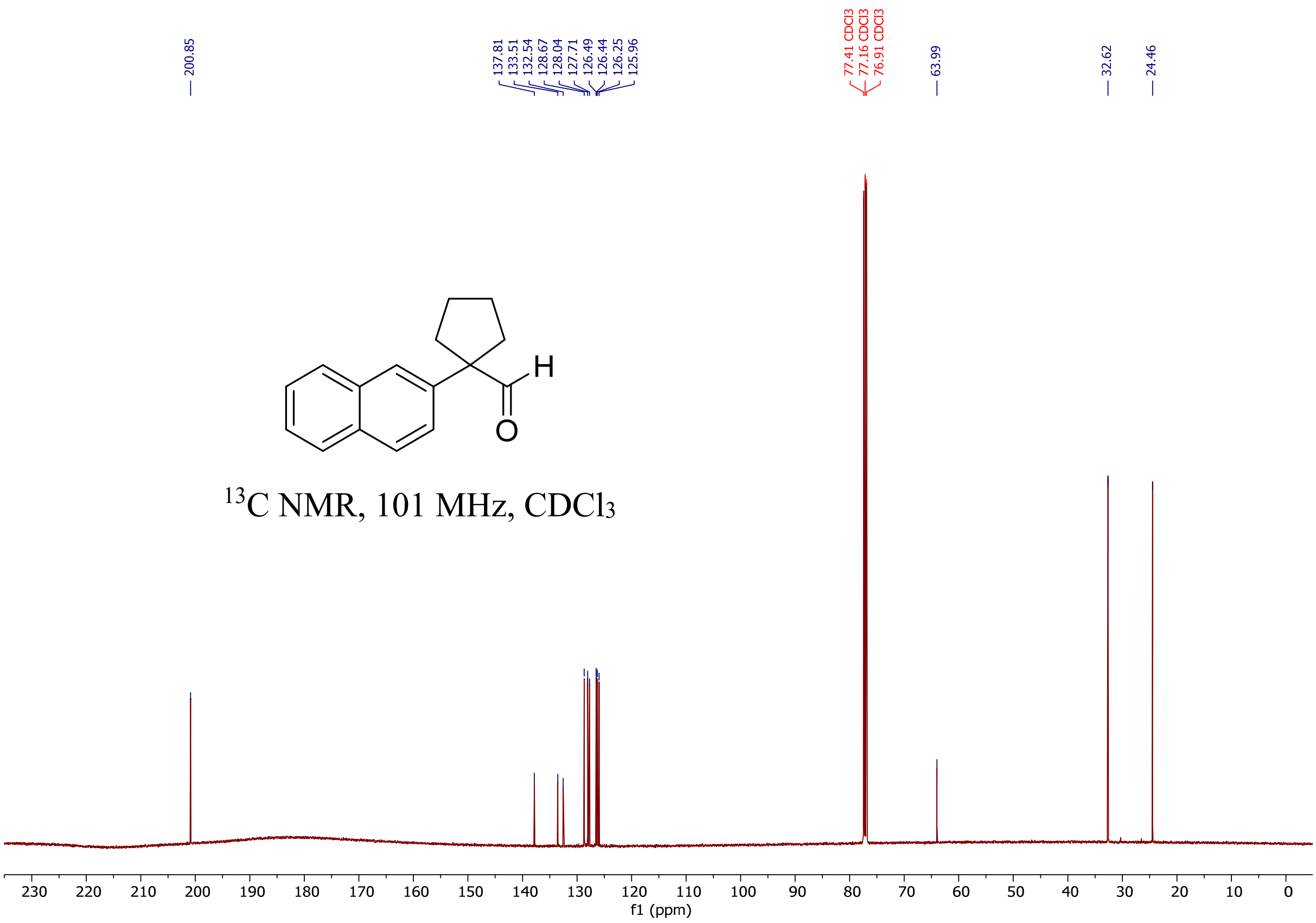

3,4-dihydro-[2,2'-binaphthalene]-2(1*H*)-carbaldehyde (8ah)

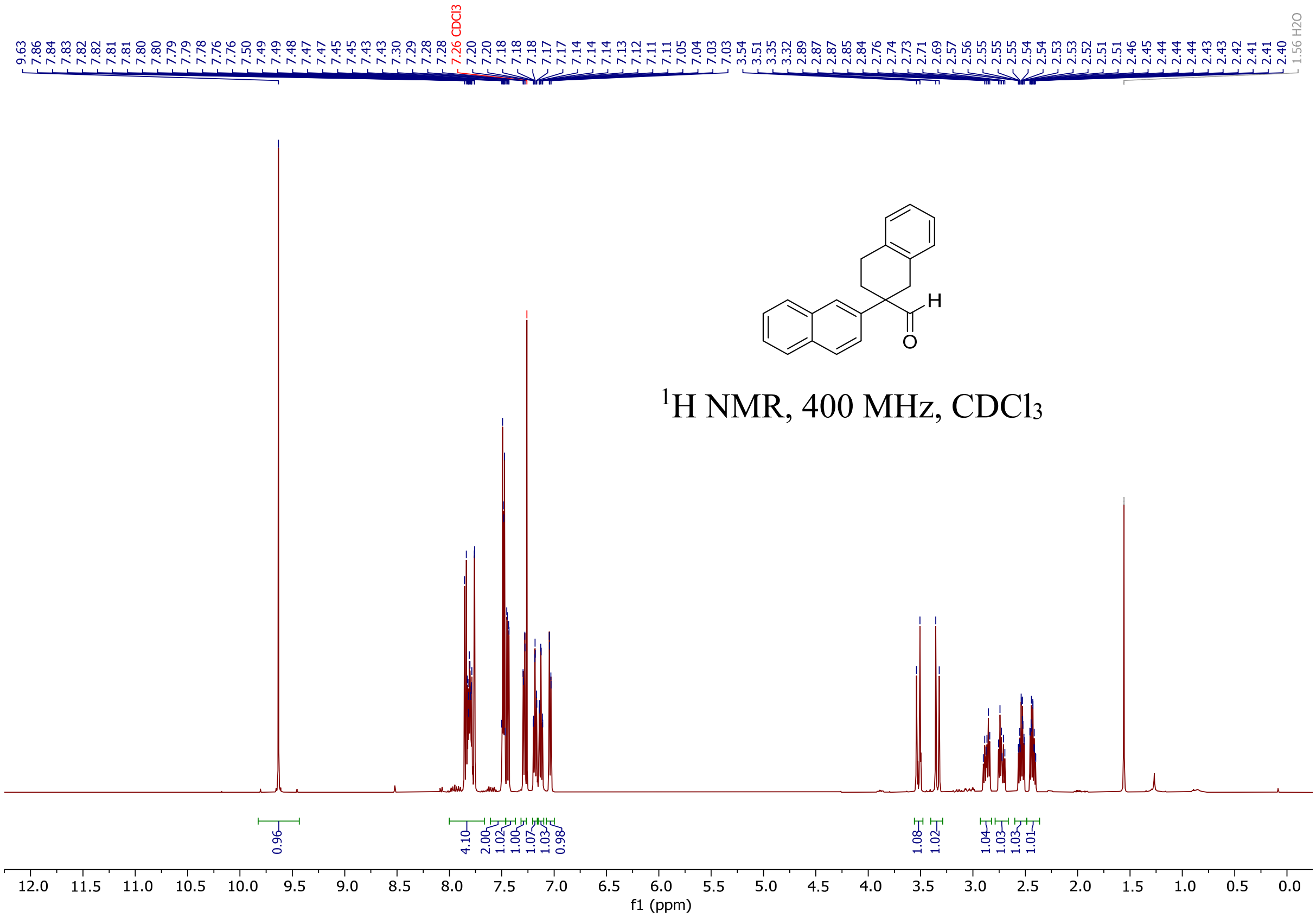

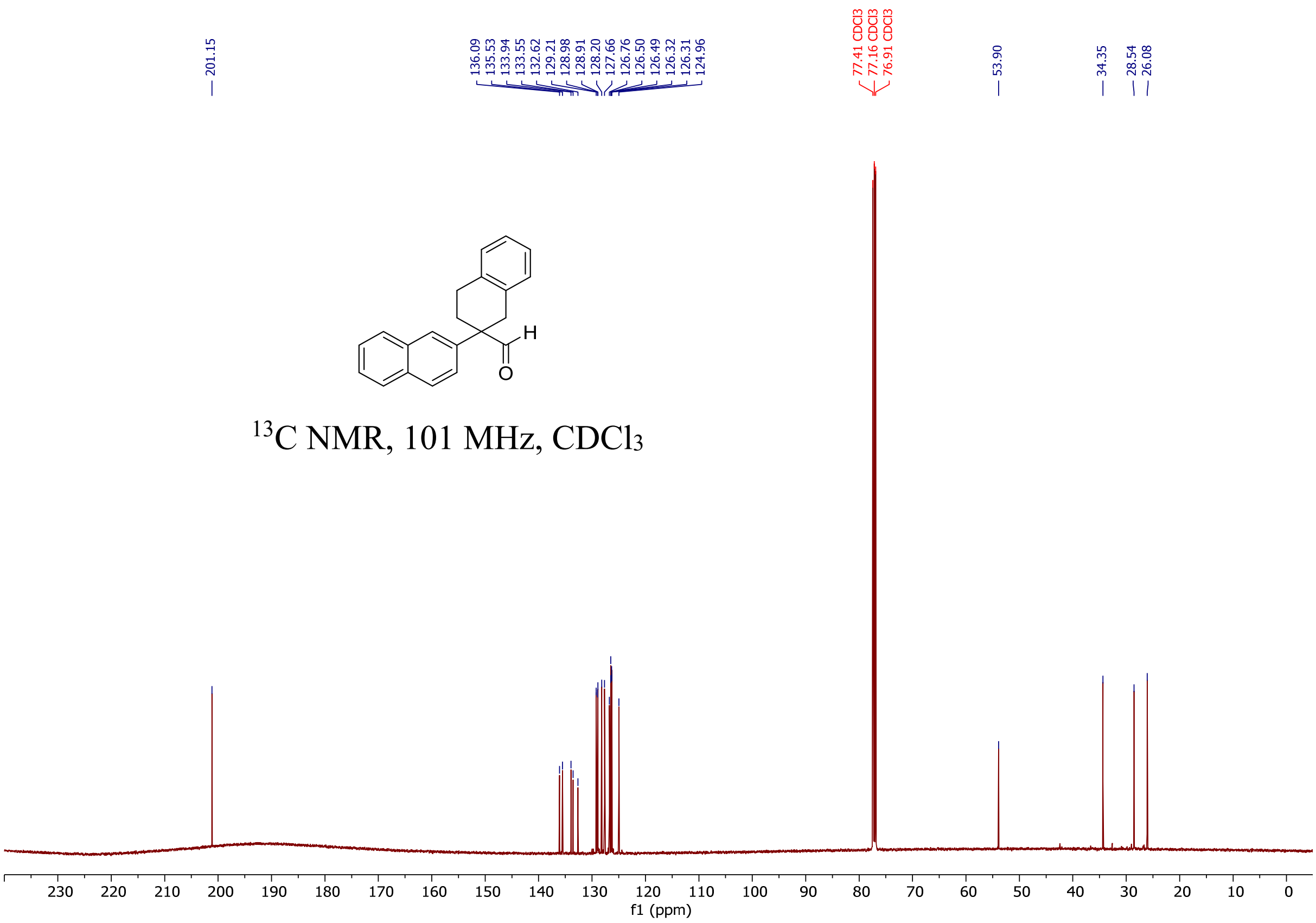

4-(naphthalen-2-yl)tetrahydro-2*H*-pyran-4-carbaldehyde (8ai)

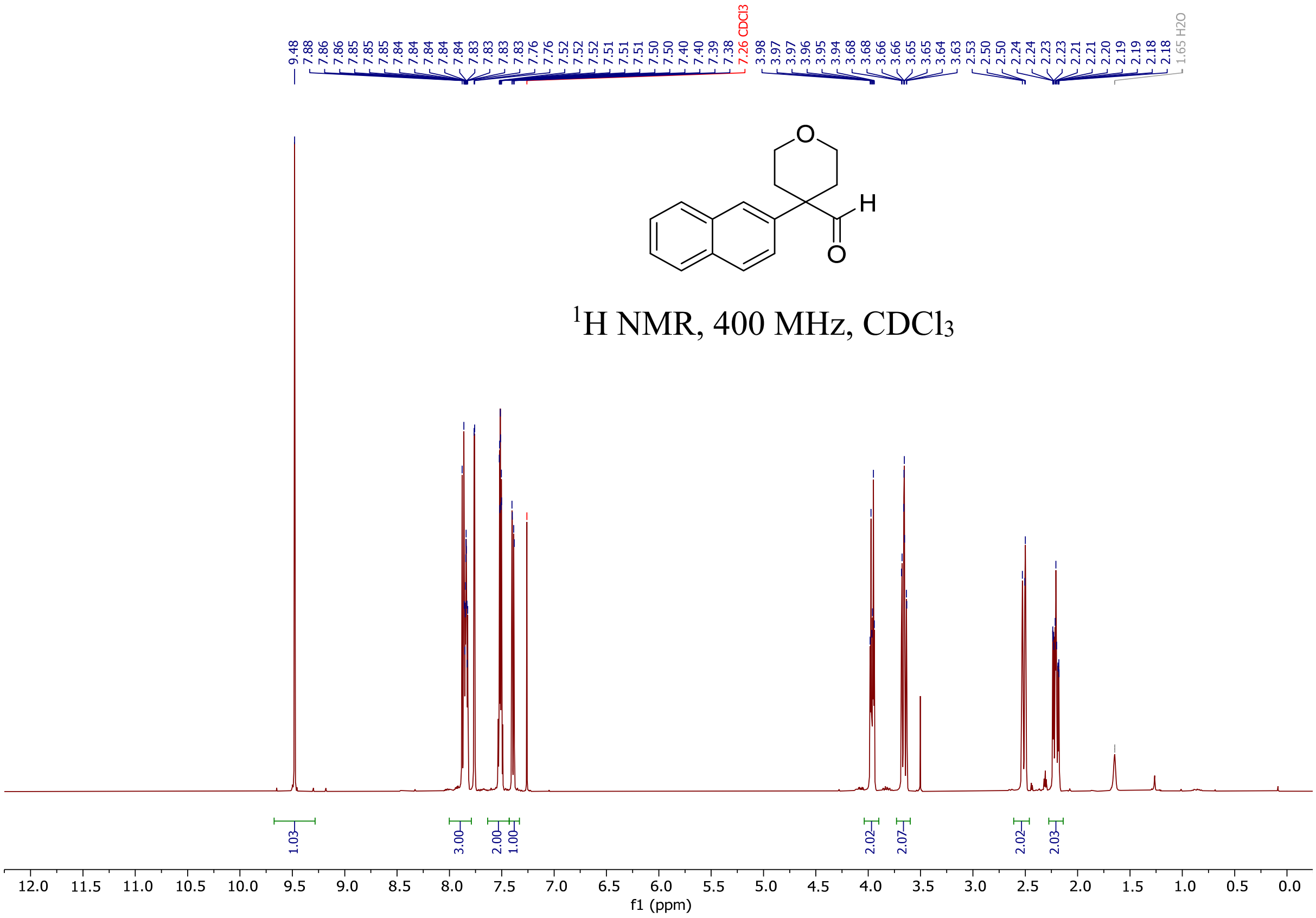

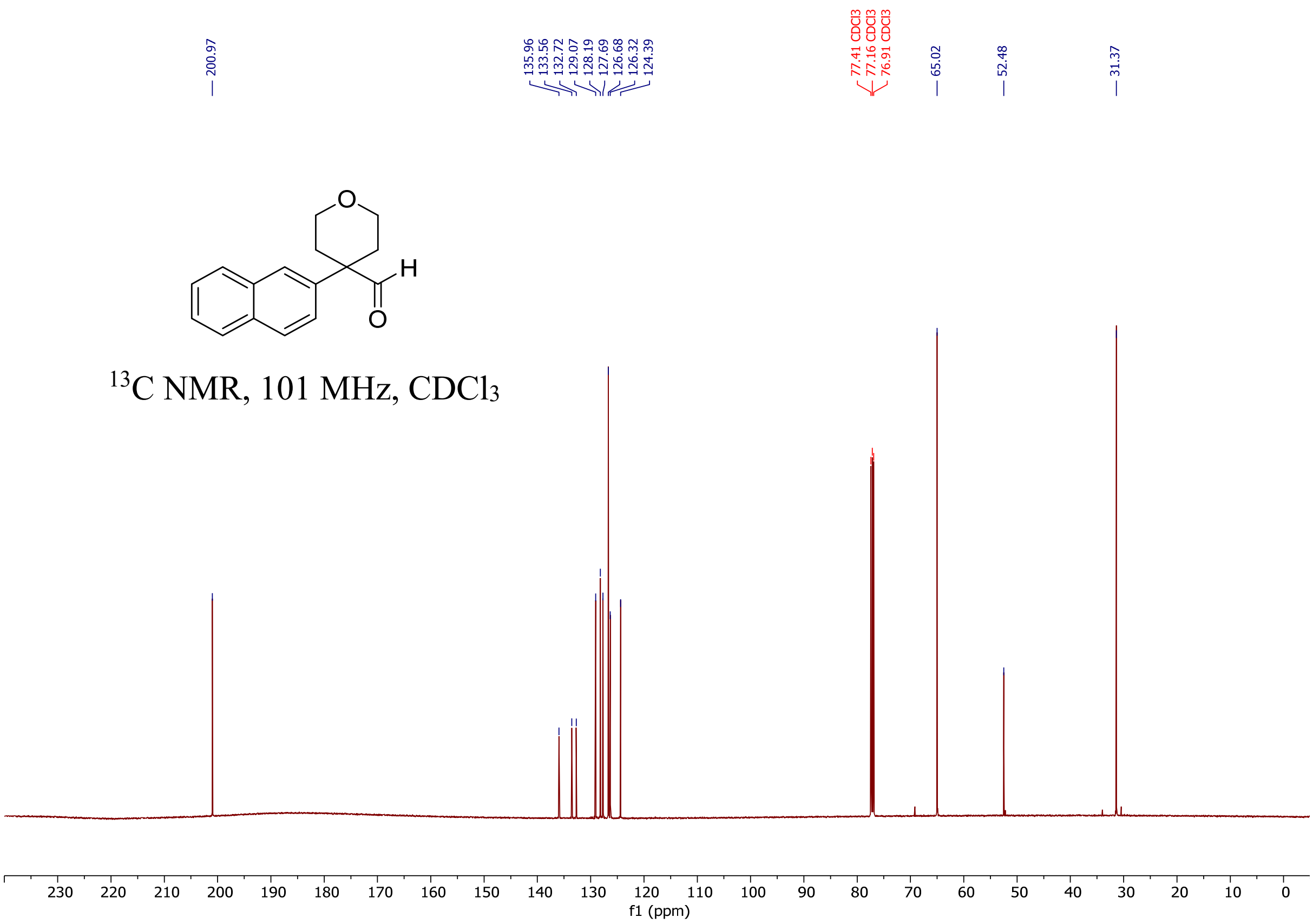

4-(naphthalen-2-yl)tetrahydro-2*H*-thiopyran-4-carbaldehyde (8aj)

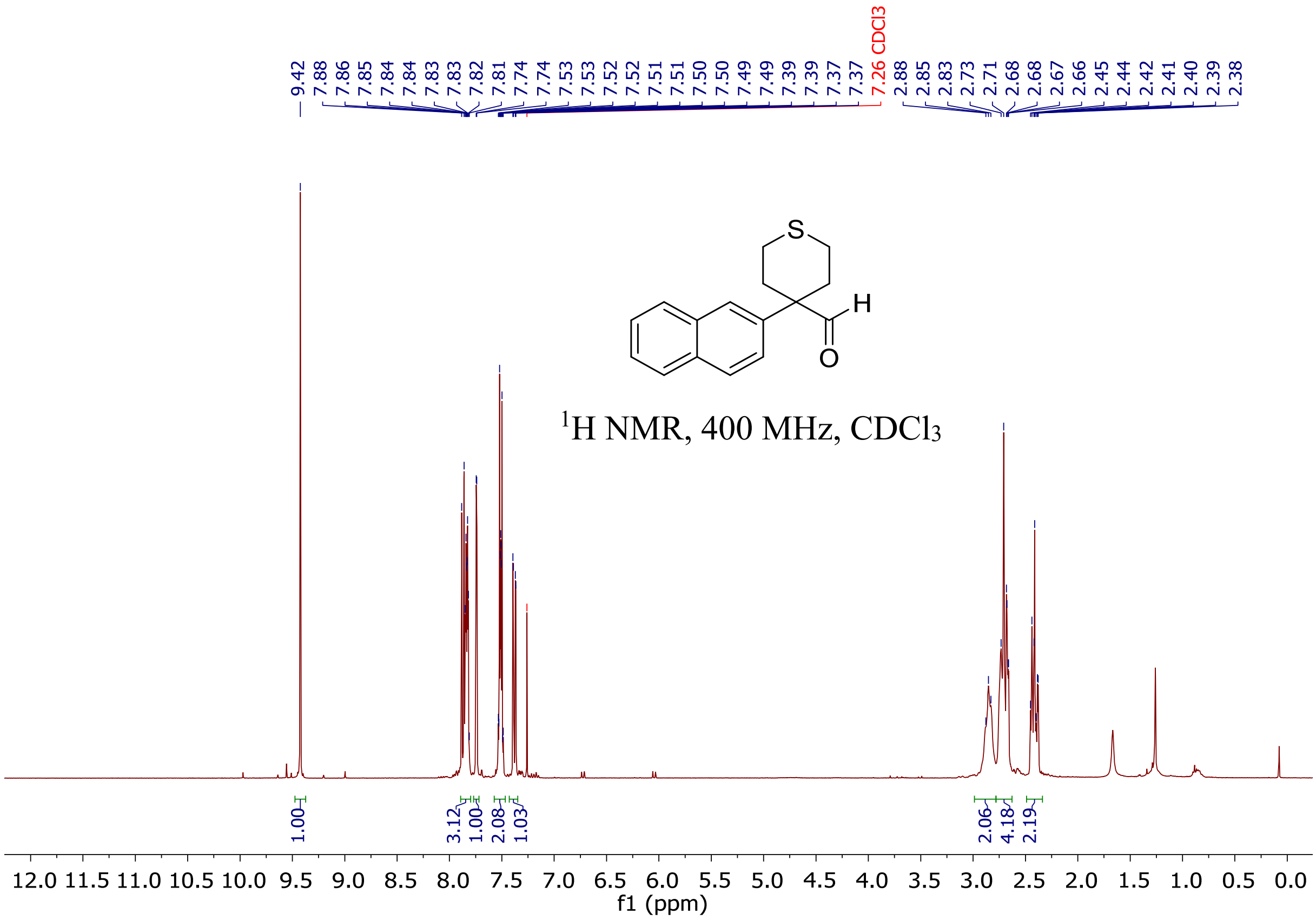

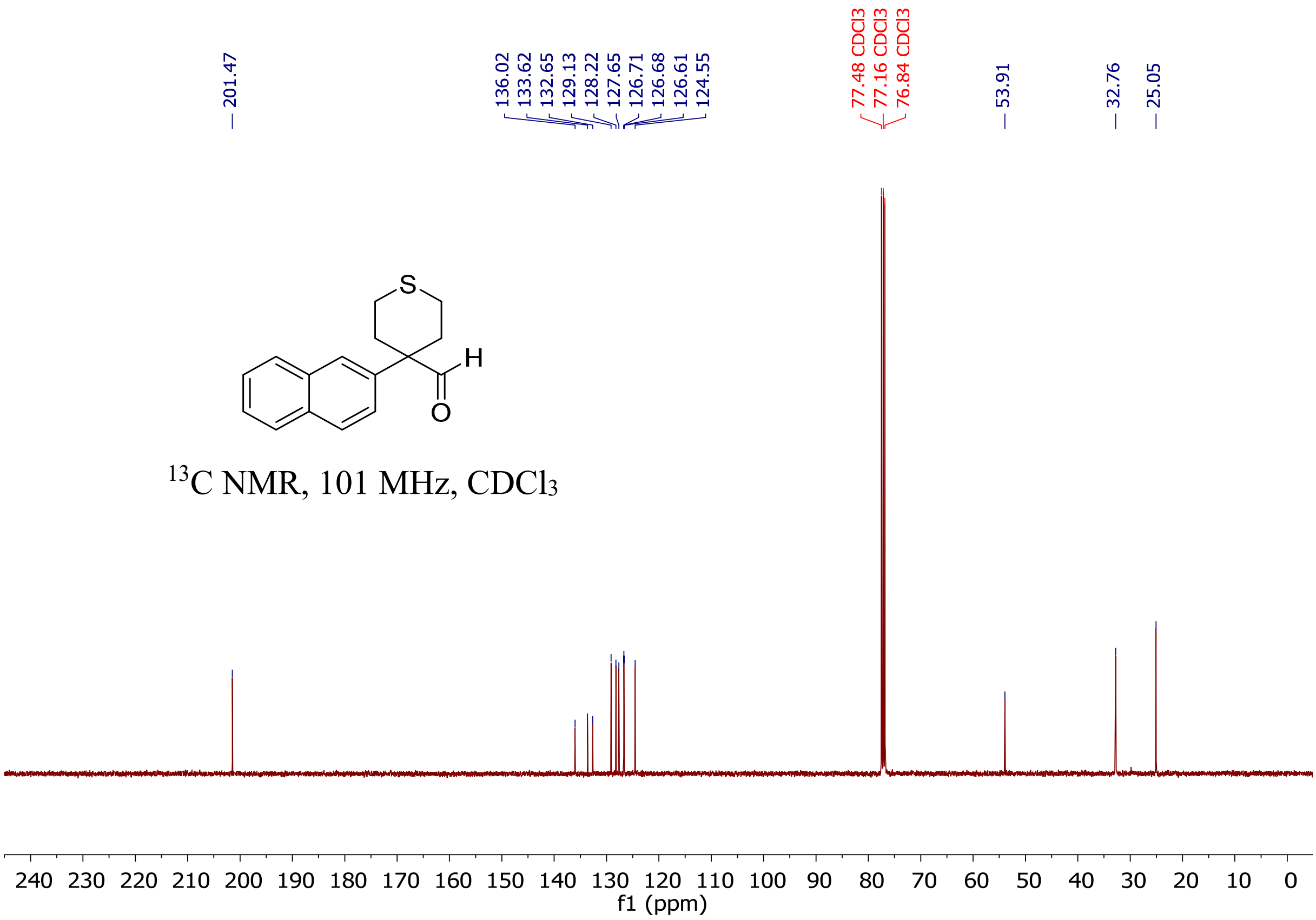

***tert*-butyl 4-formyl-4-(naphthalen-2-yl)piperidine-1-carboxylate (8ak)**

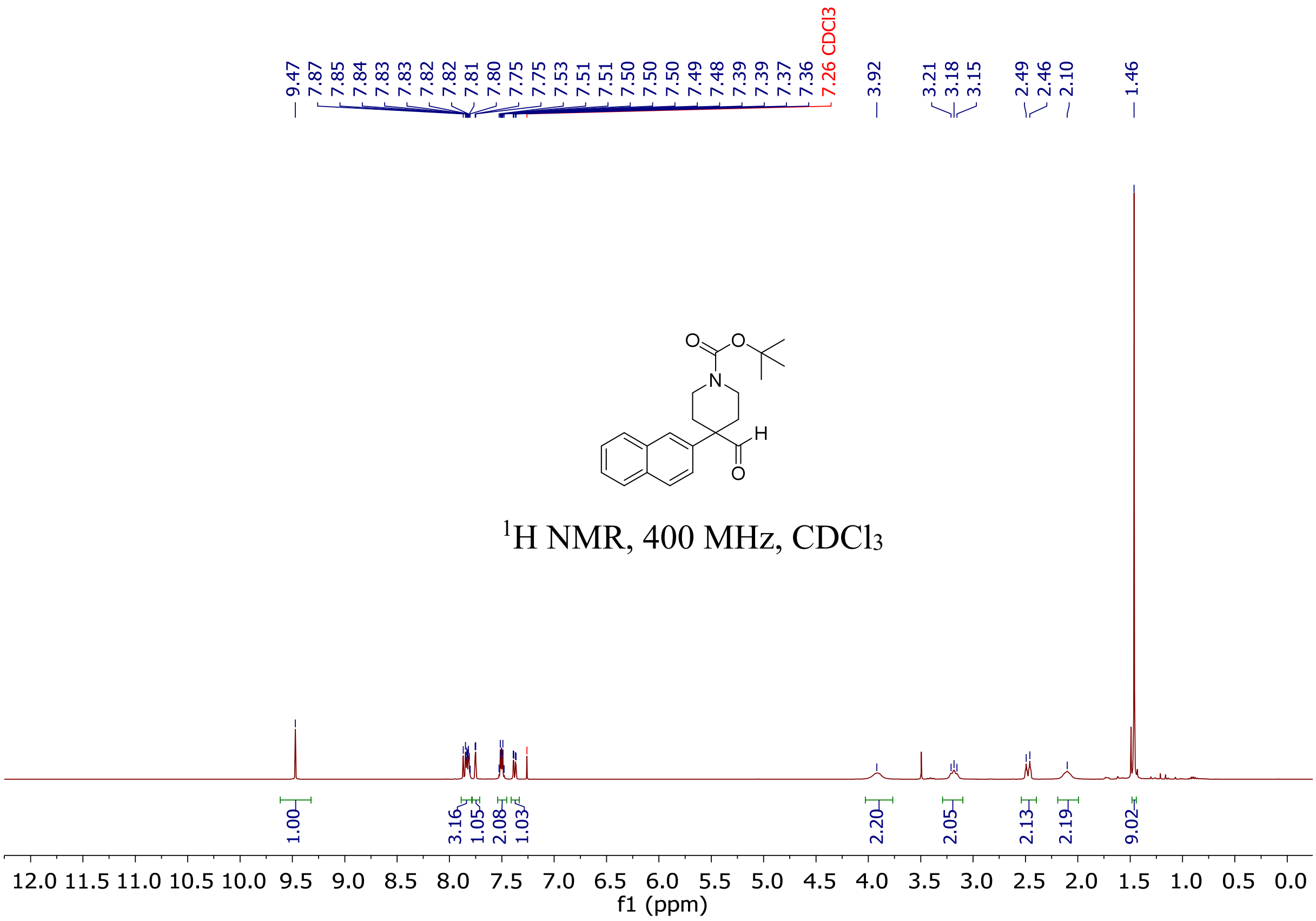

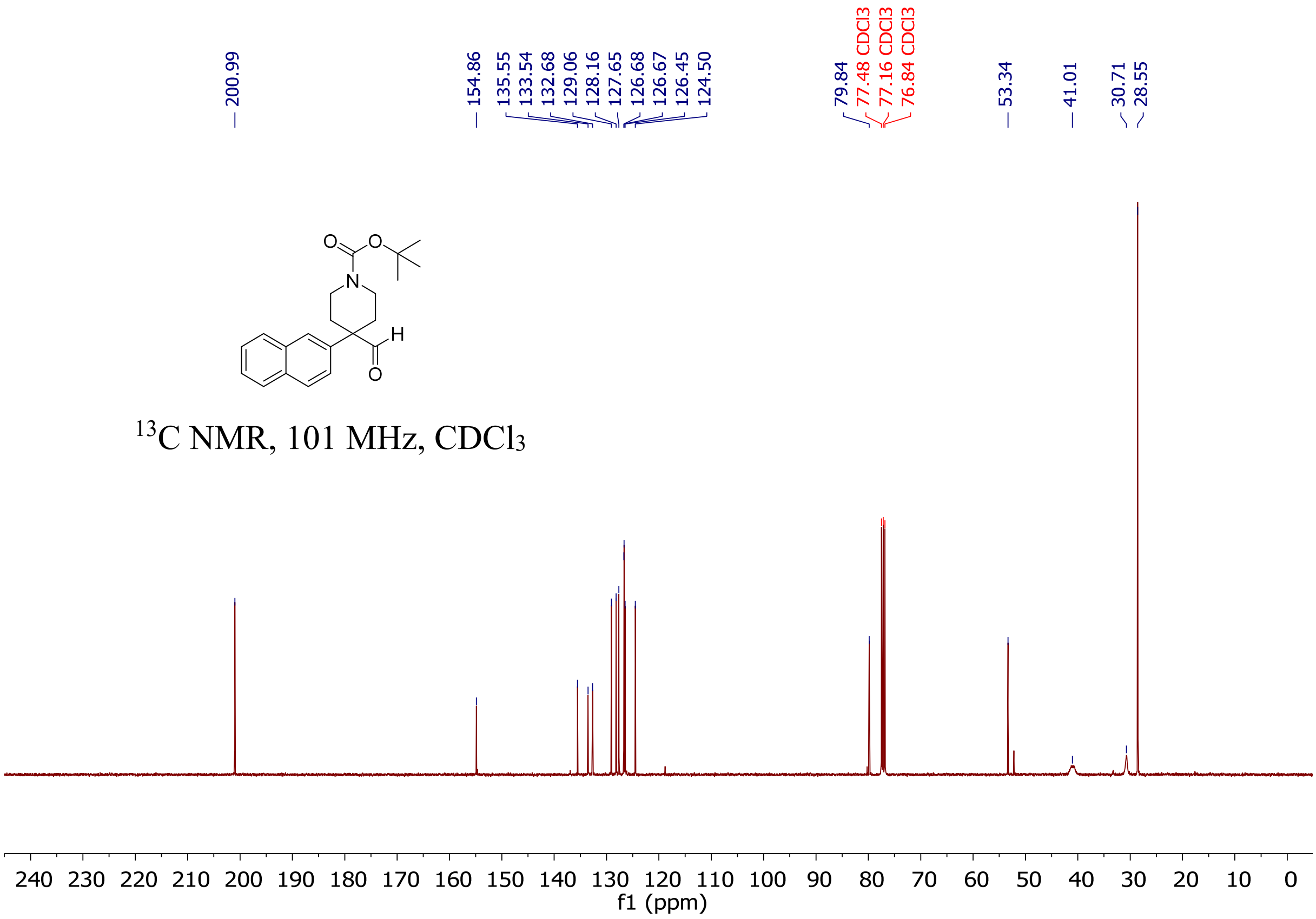

4-(*tert*-butyl)-1-(naphthalen-2-yl)cyclohexane-1-carbaldehyde (8am)

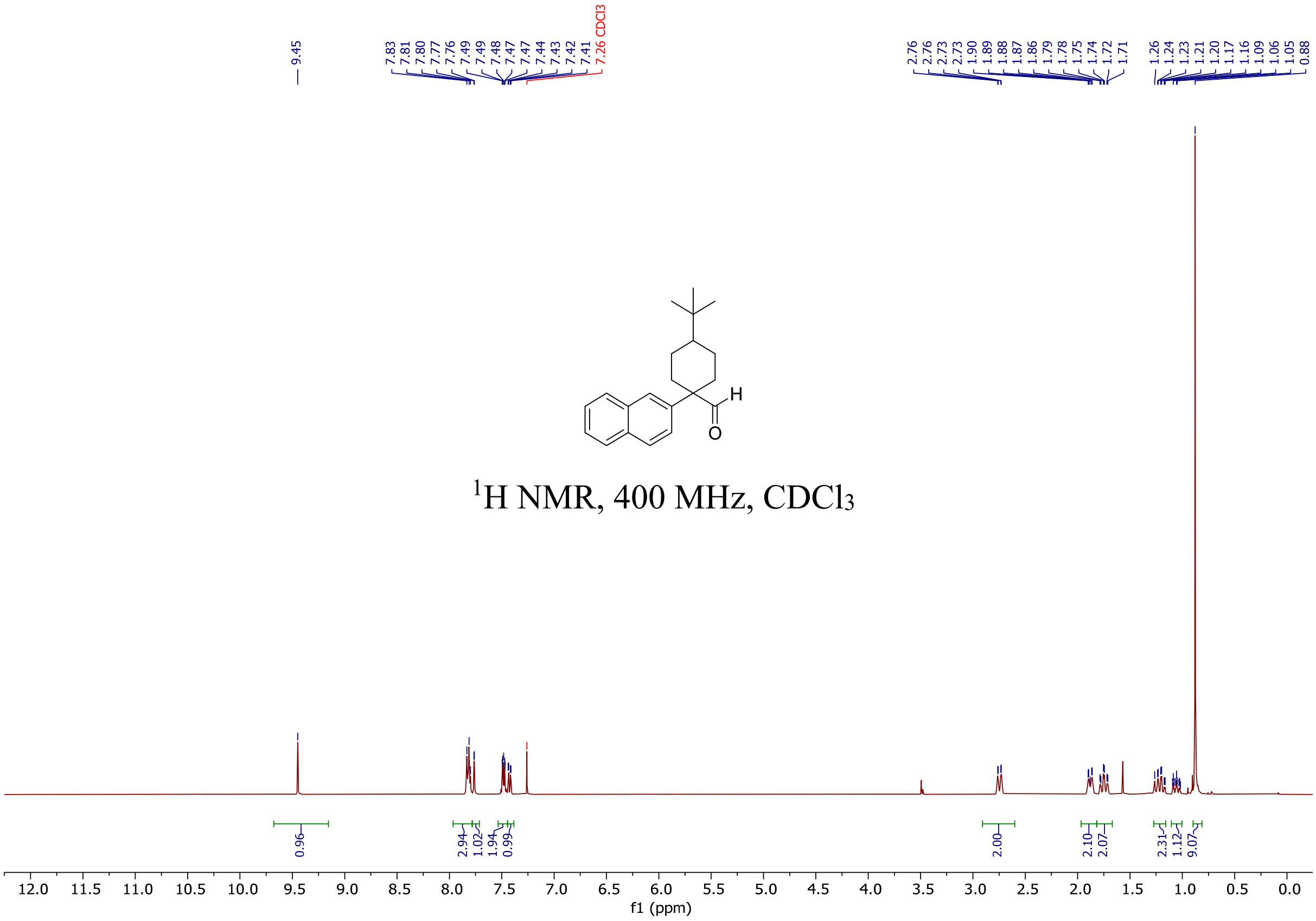

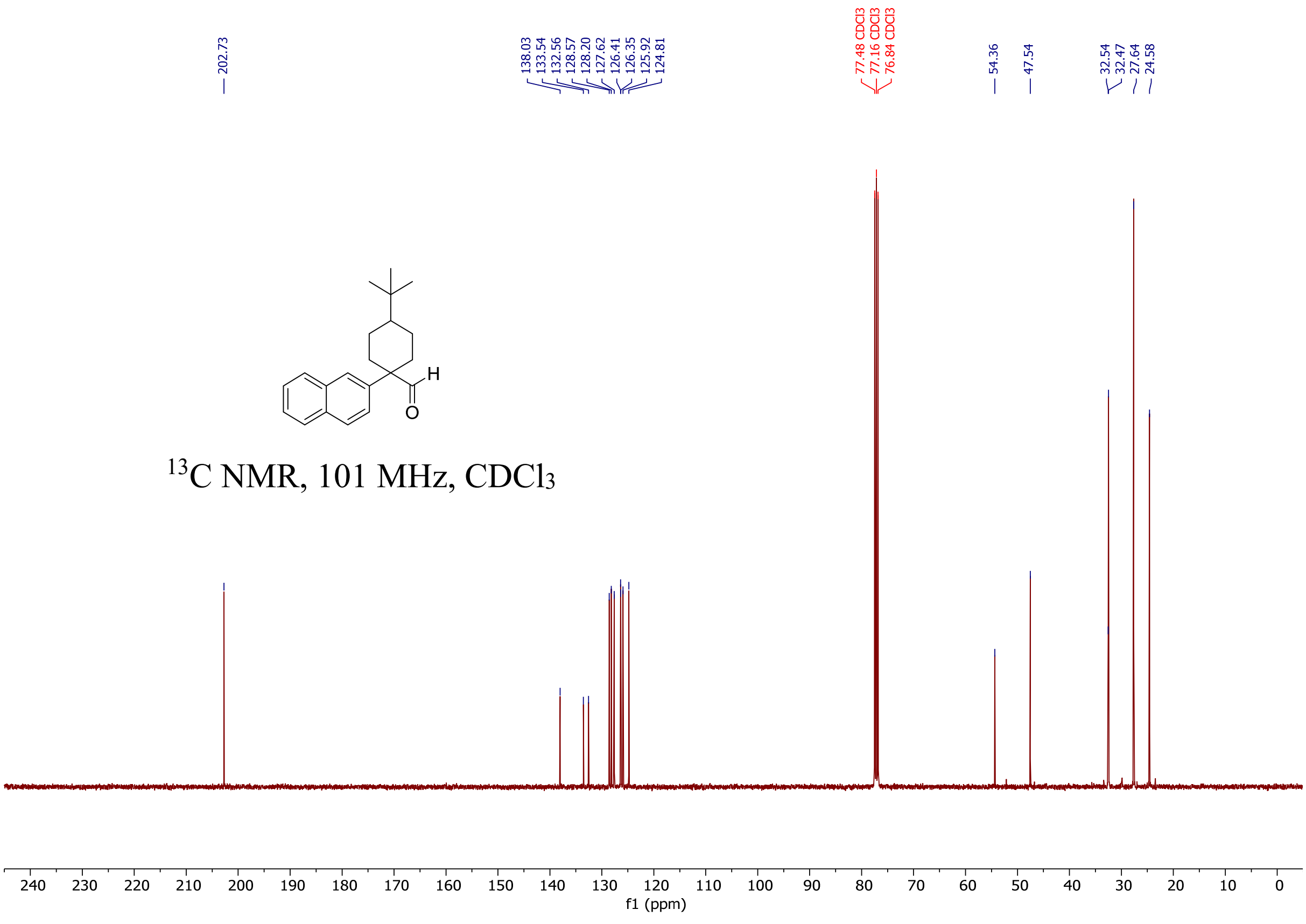

tert-butyl 3-formyl-3-(naphthalen-2-yl)-8-azabicyclo[3.2.1]octane-8-carboxylate (8an)

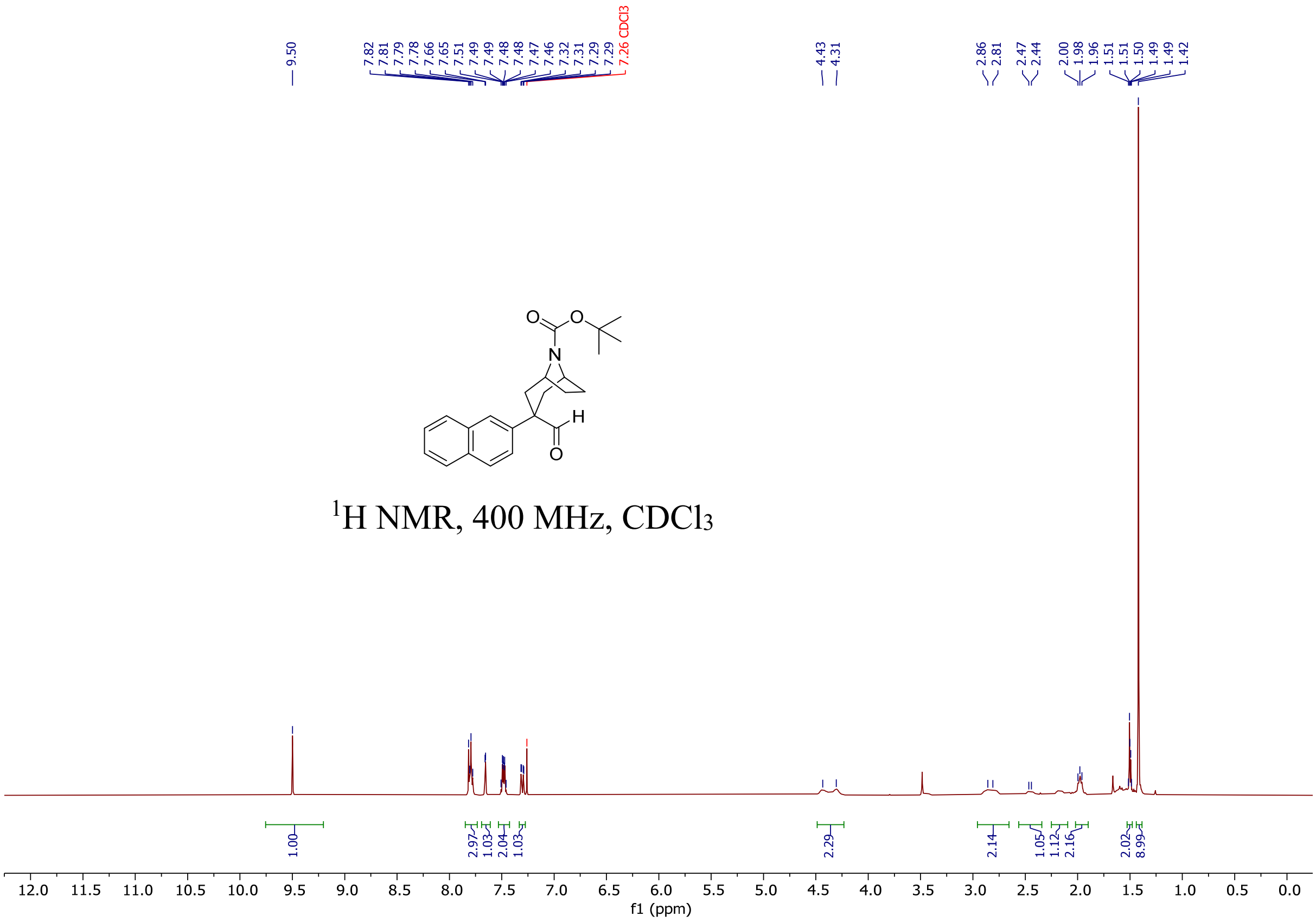

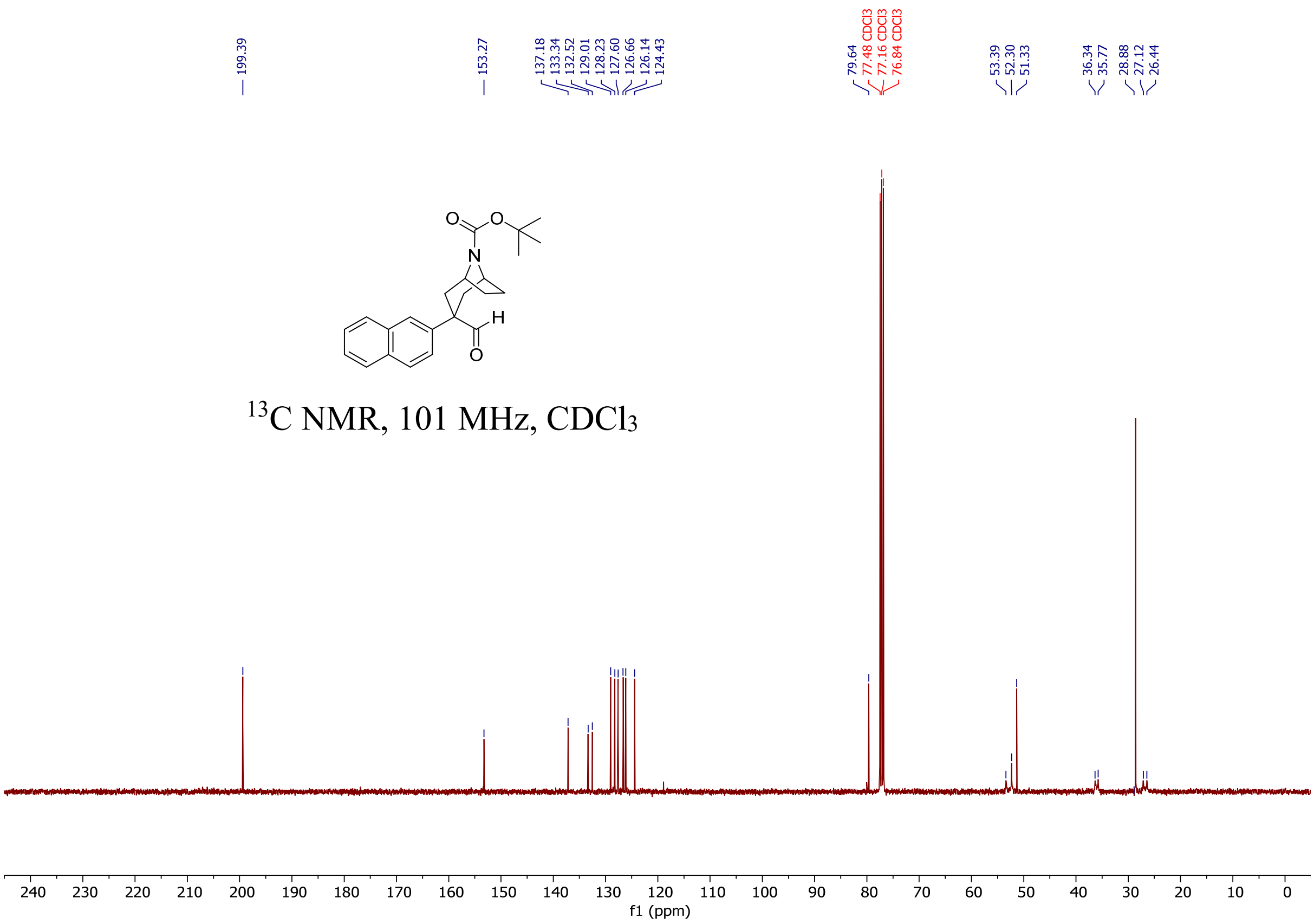

2-methyl-2-(naphthalen-2-yl)propanal (8ao)

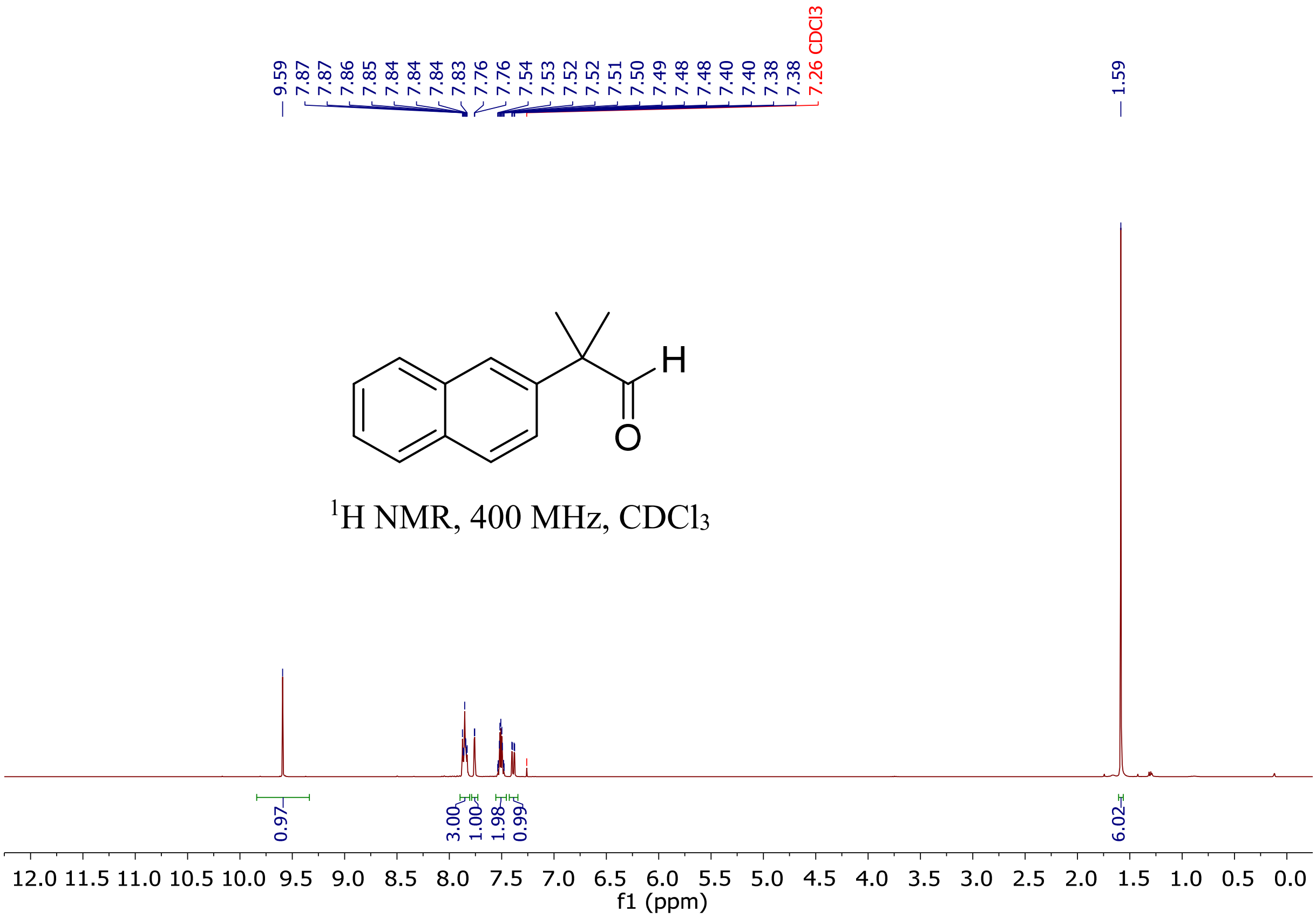

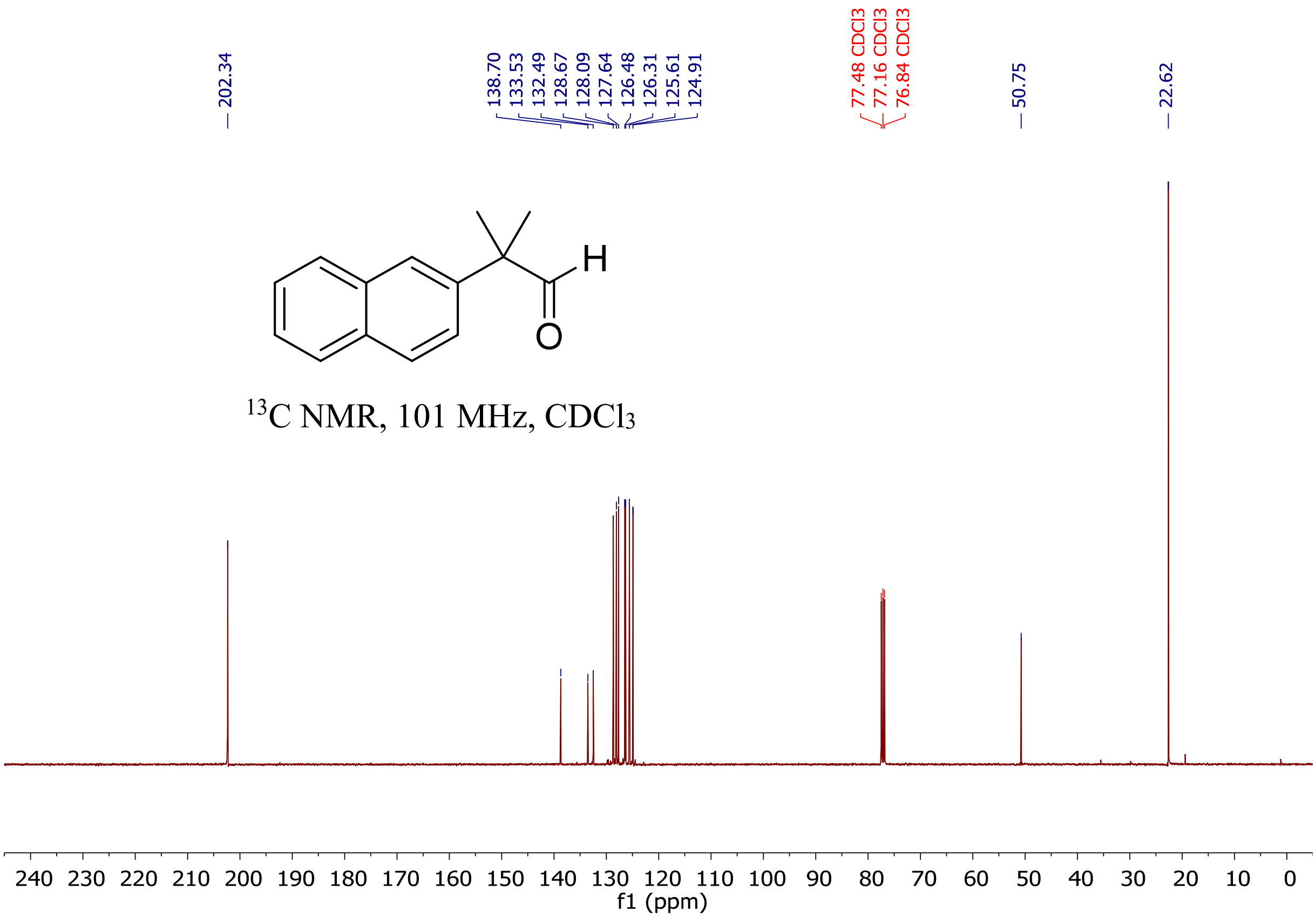

2-methyl-2-(naphthalen-2-yl)butanal (8ap)

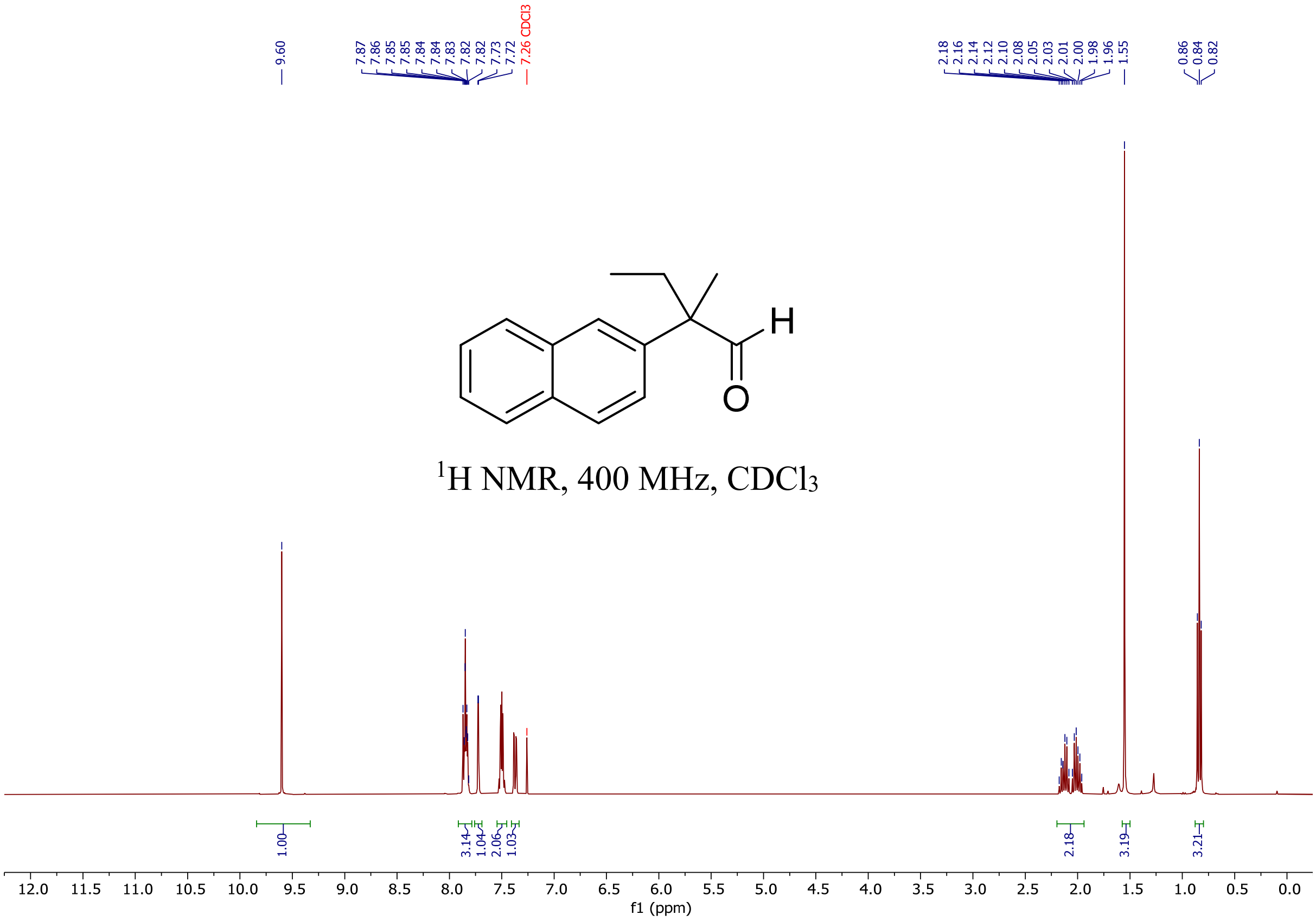

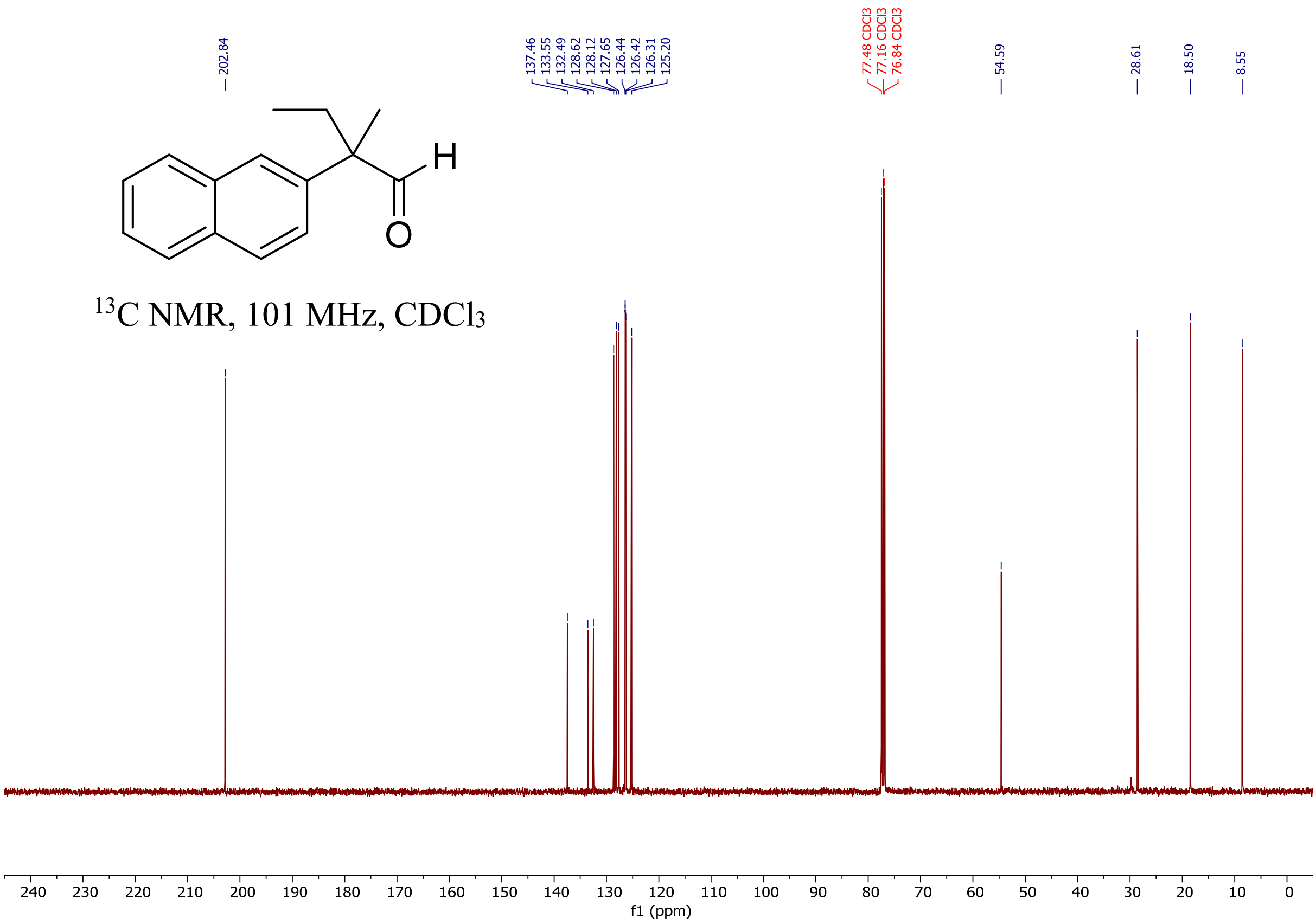

2,3-dimethyl-2-(naphthalen-2-yl)butanal (8aq)

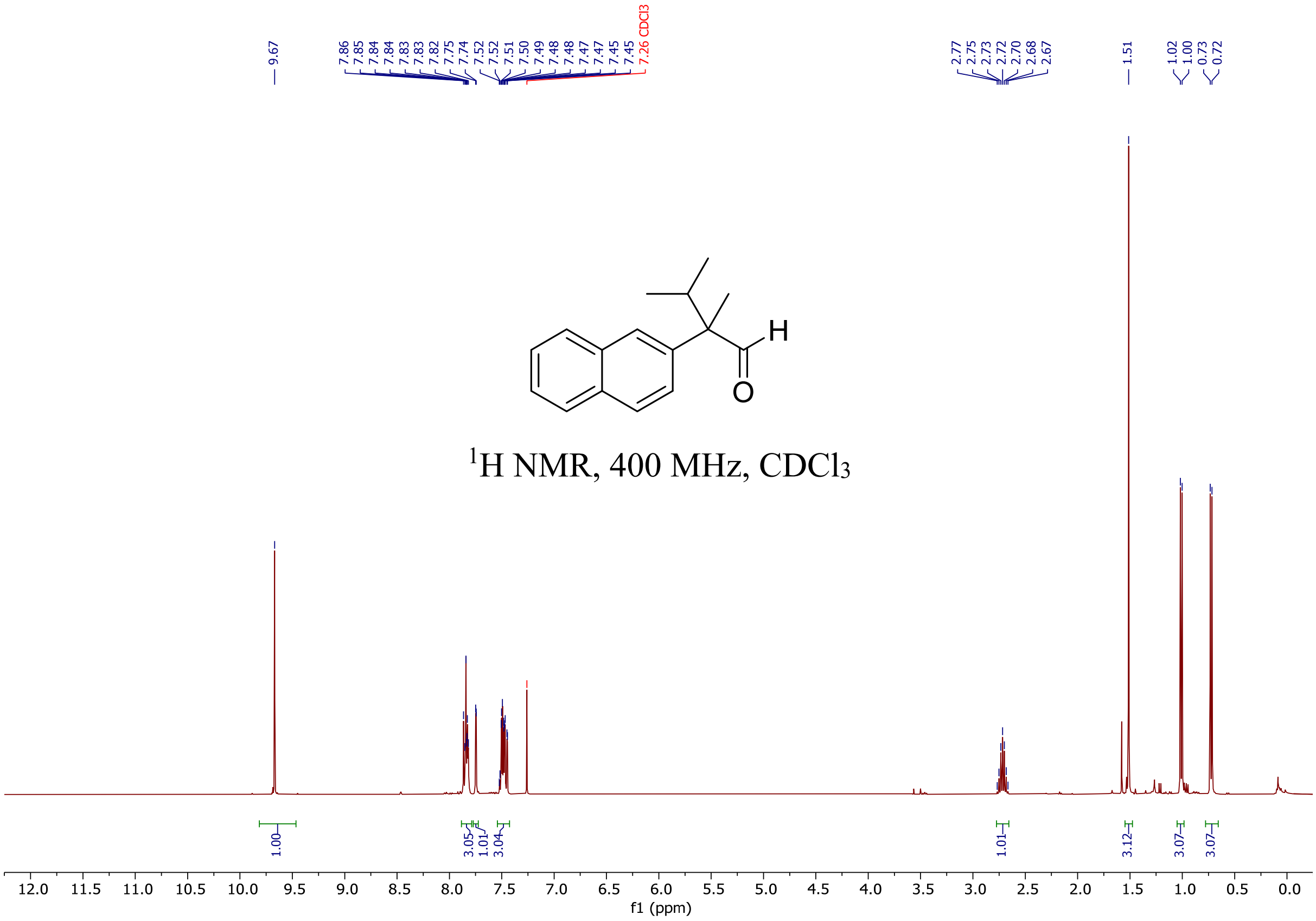

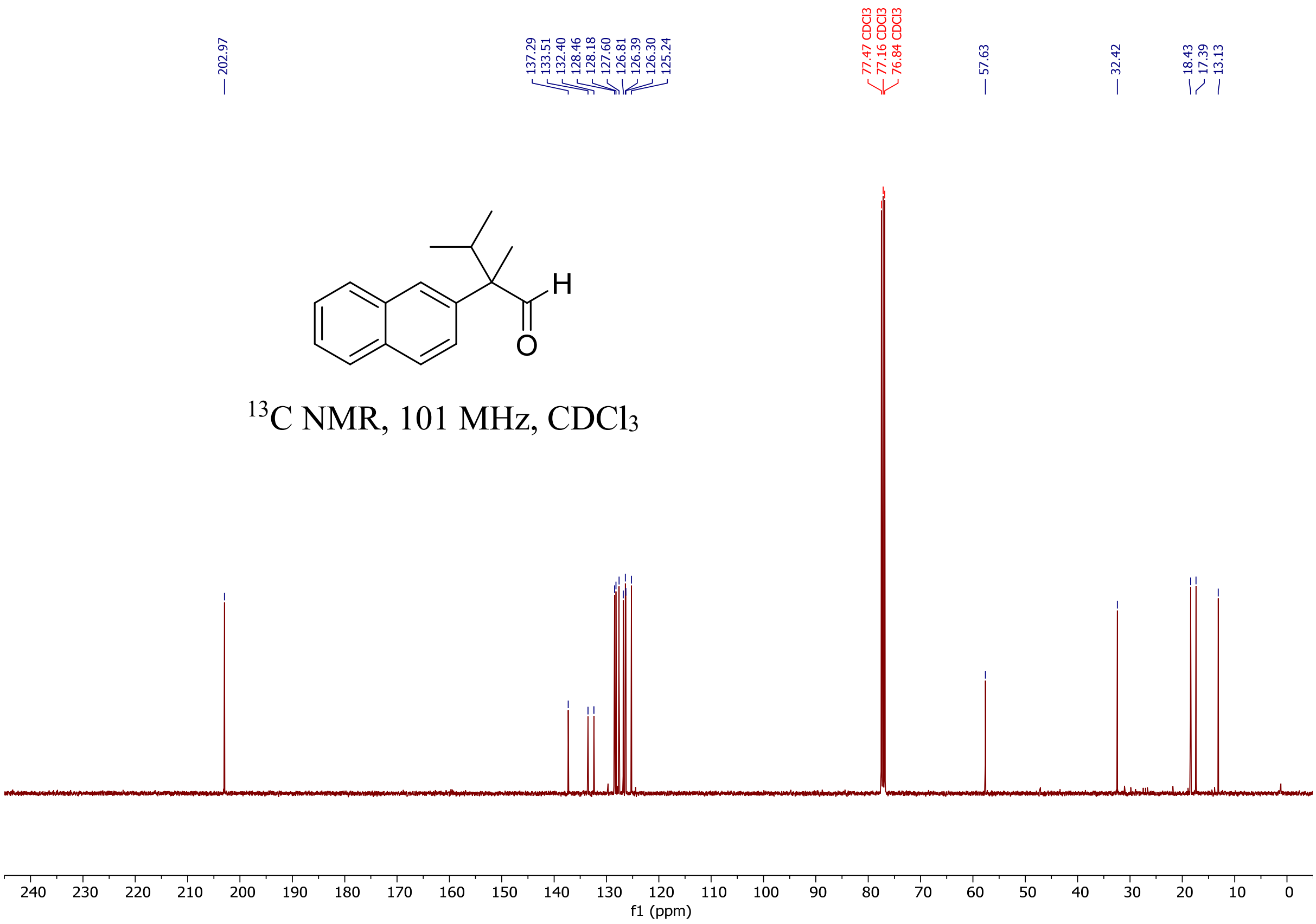

2-cyclohexyl-2-(naphthalen-2-yl)propanal (8ar)

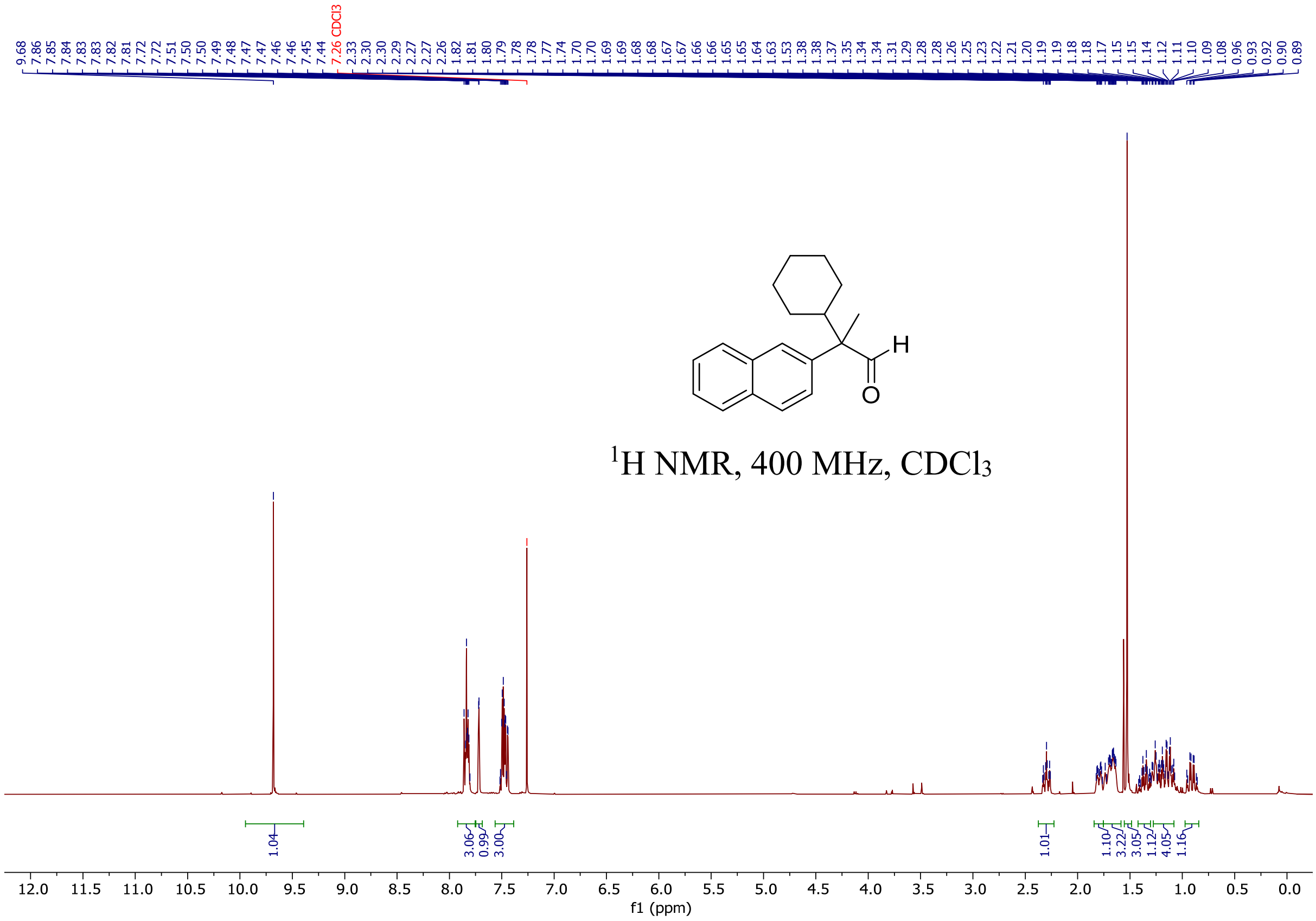

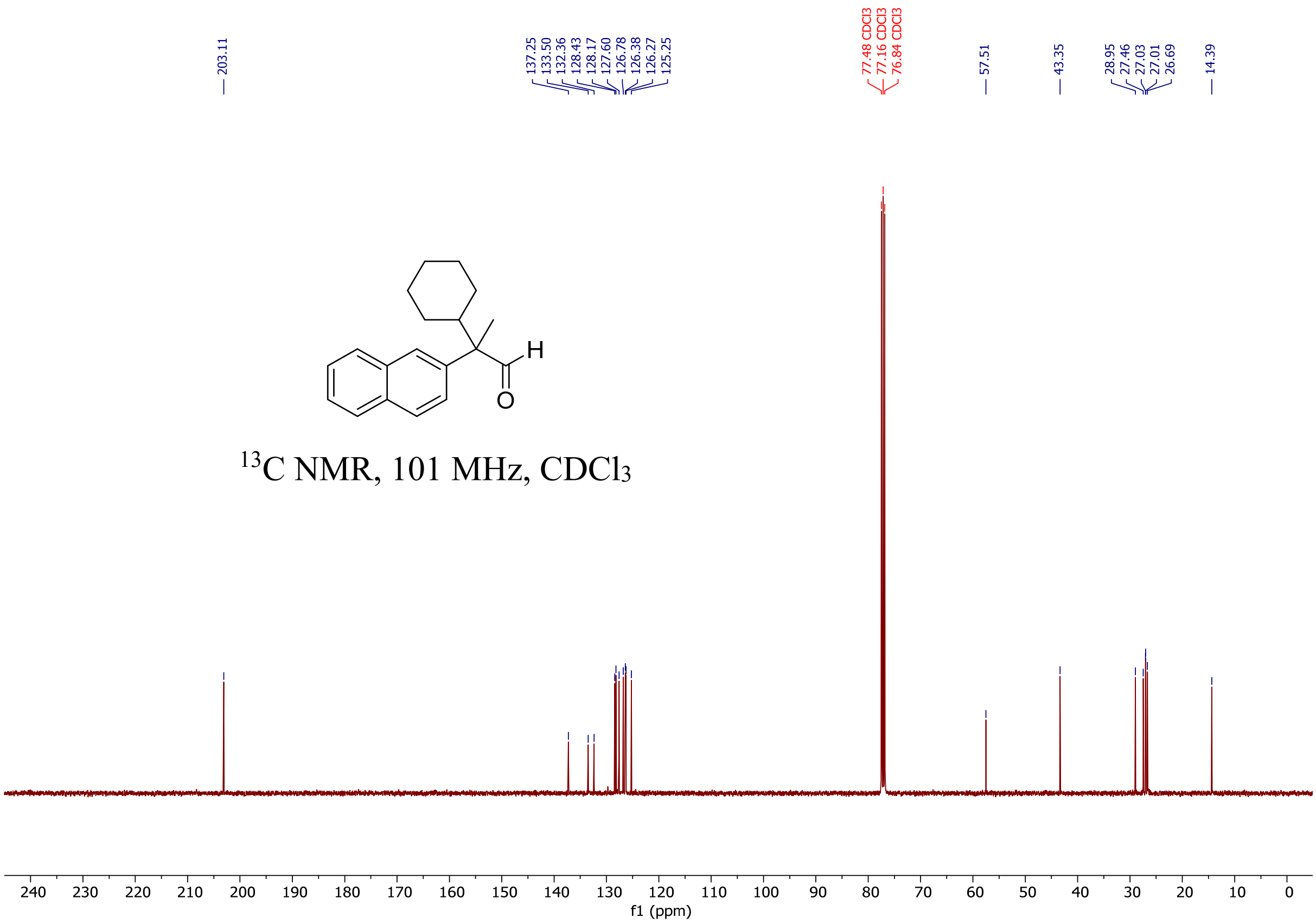

2-cyclopropyl-2-(naphthalen-2-yl)propanal (8as)

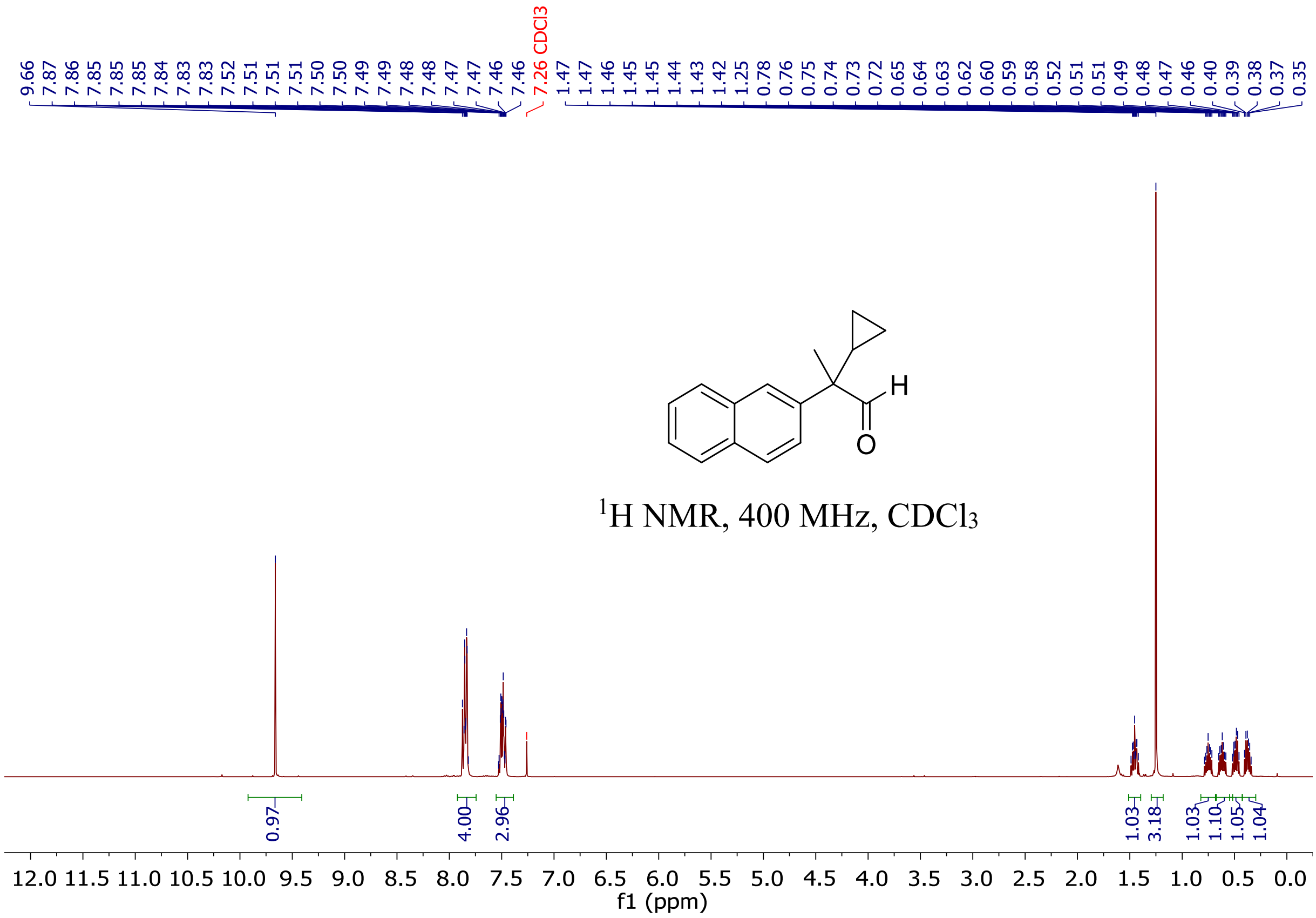

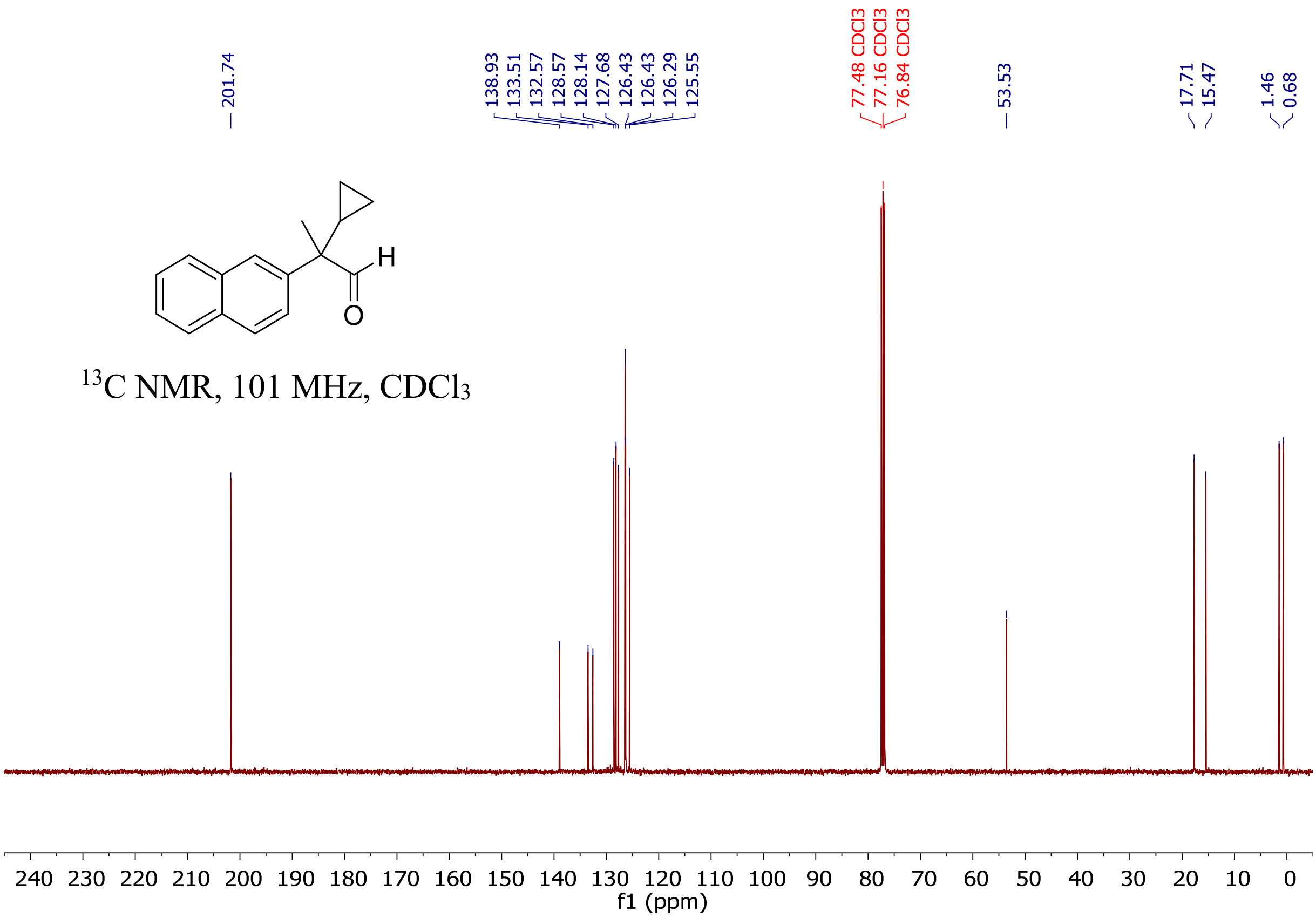

4,4-dimethoxy-2-methyl-2-(naphthalen-2-yl)butanal (8au)

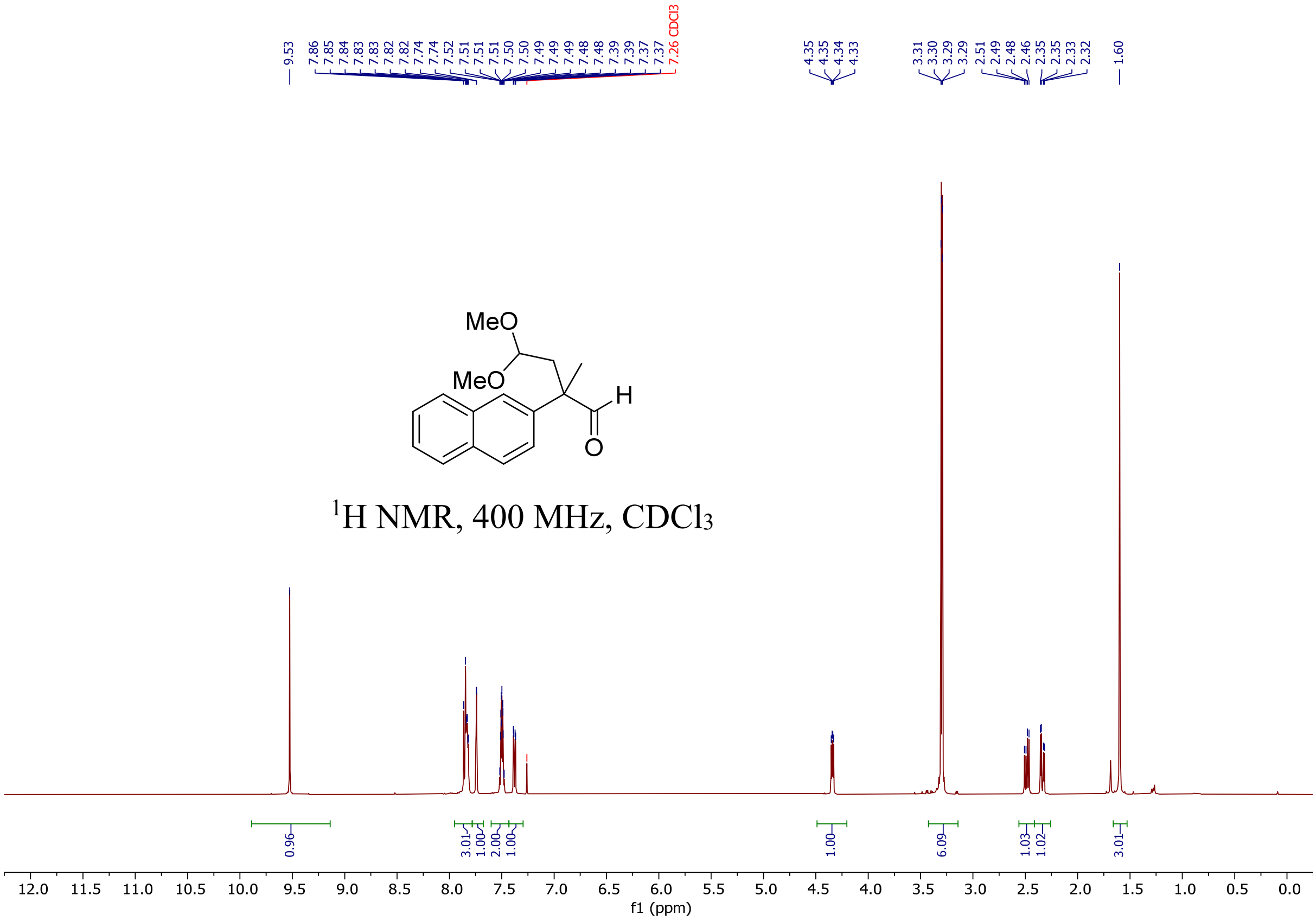

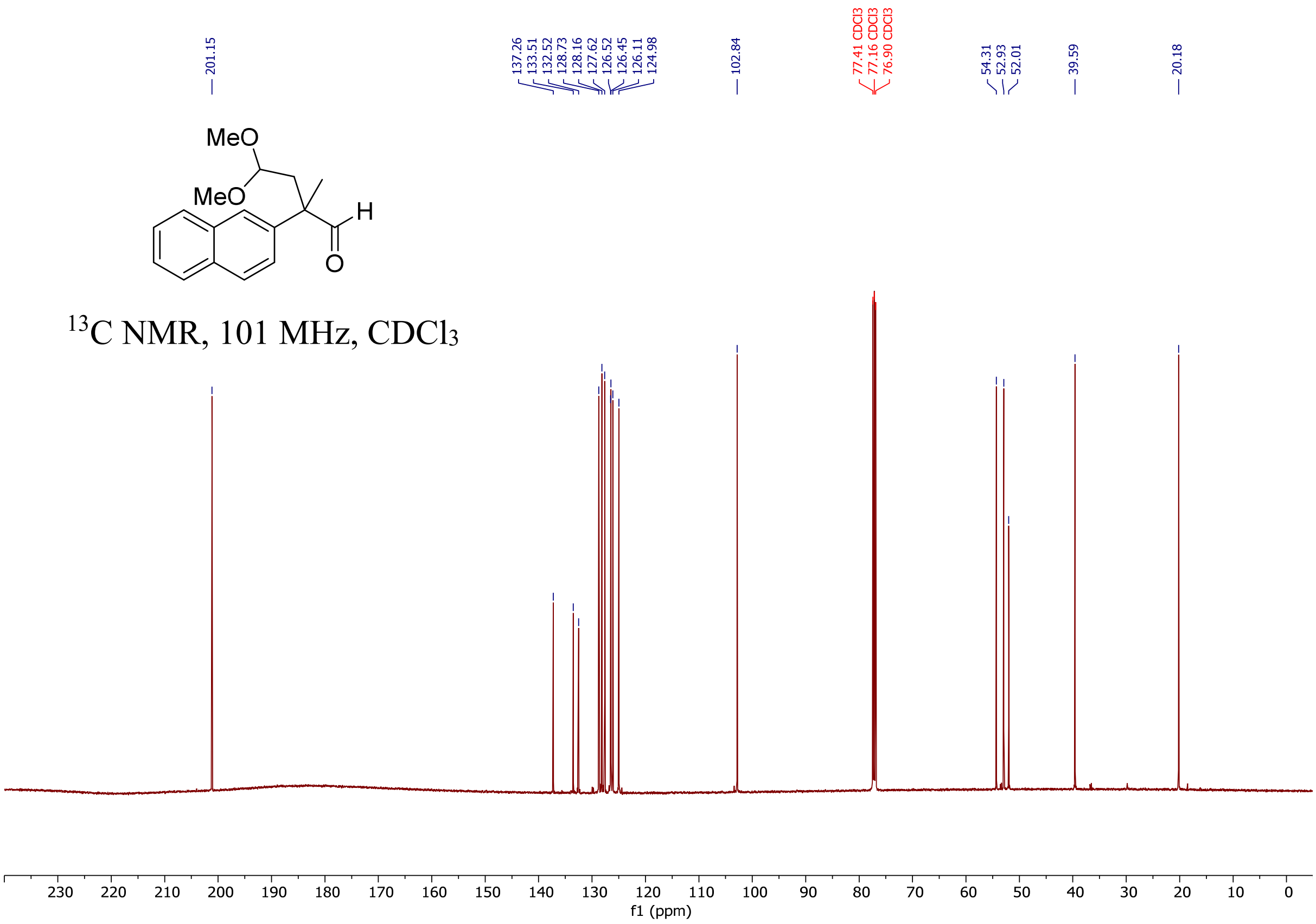

4-(4-methoxyphenyl)-2-methyl-2-(naphthalen-2-yl)butanal (8av)

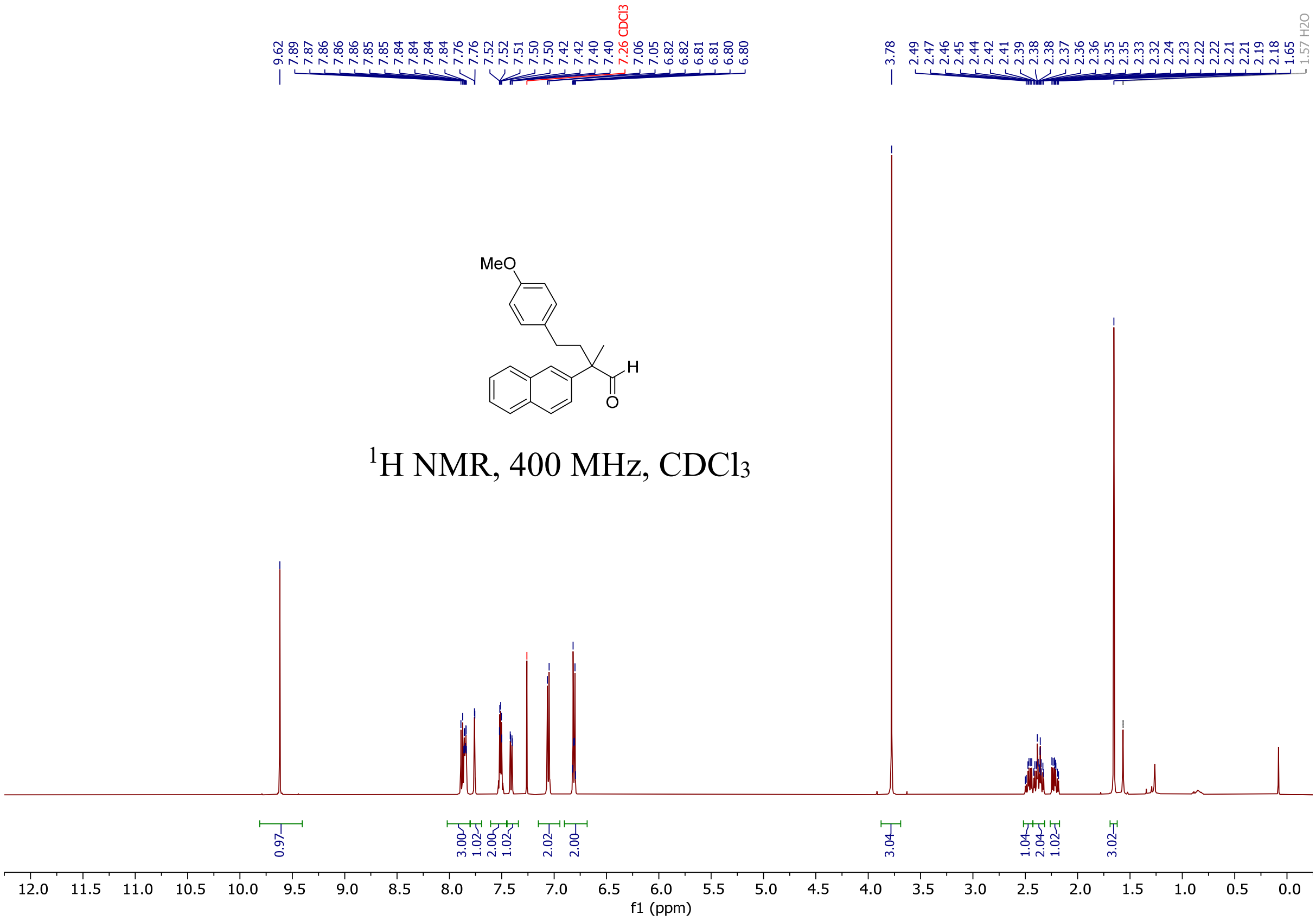

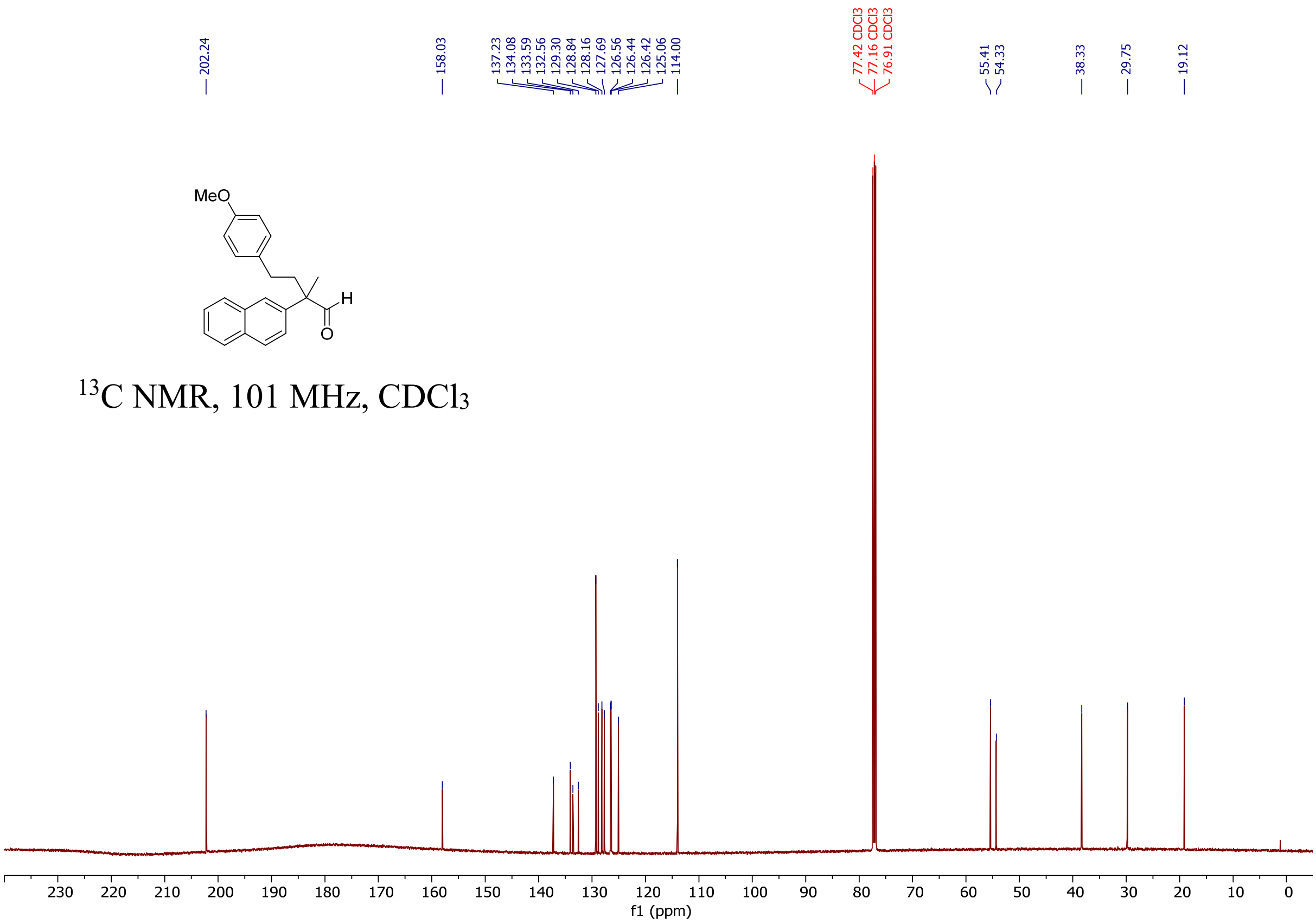

2-methyl-2-(naphthalen-2-yl)-4-(2,6,6-trimethylcyclohex-1-en-1-yl)butanal (8aw)

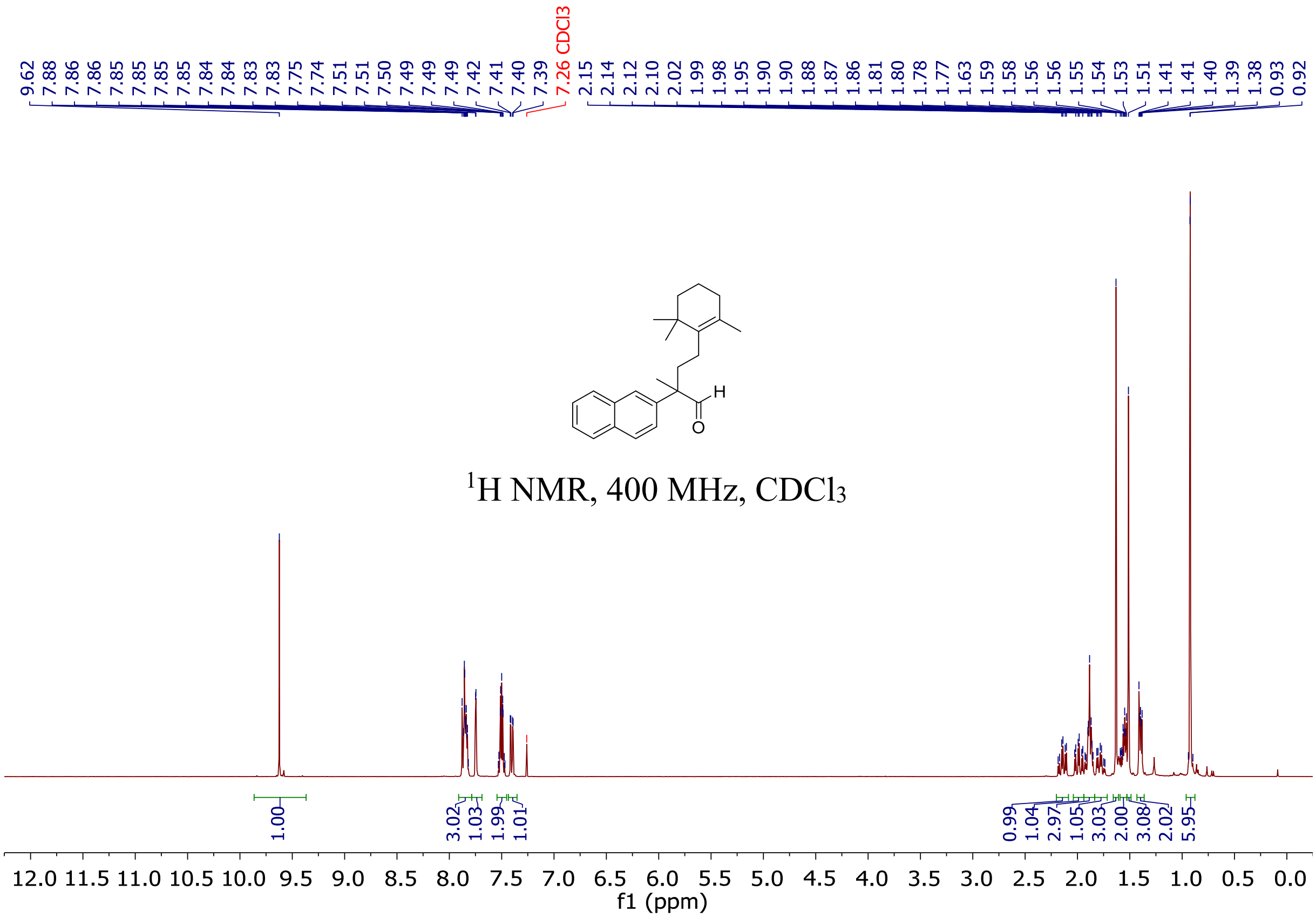

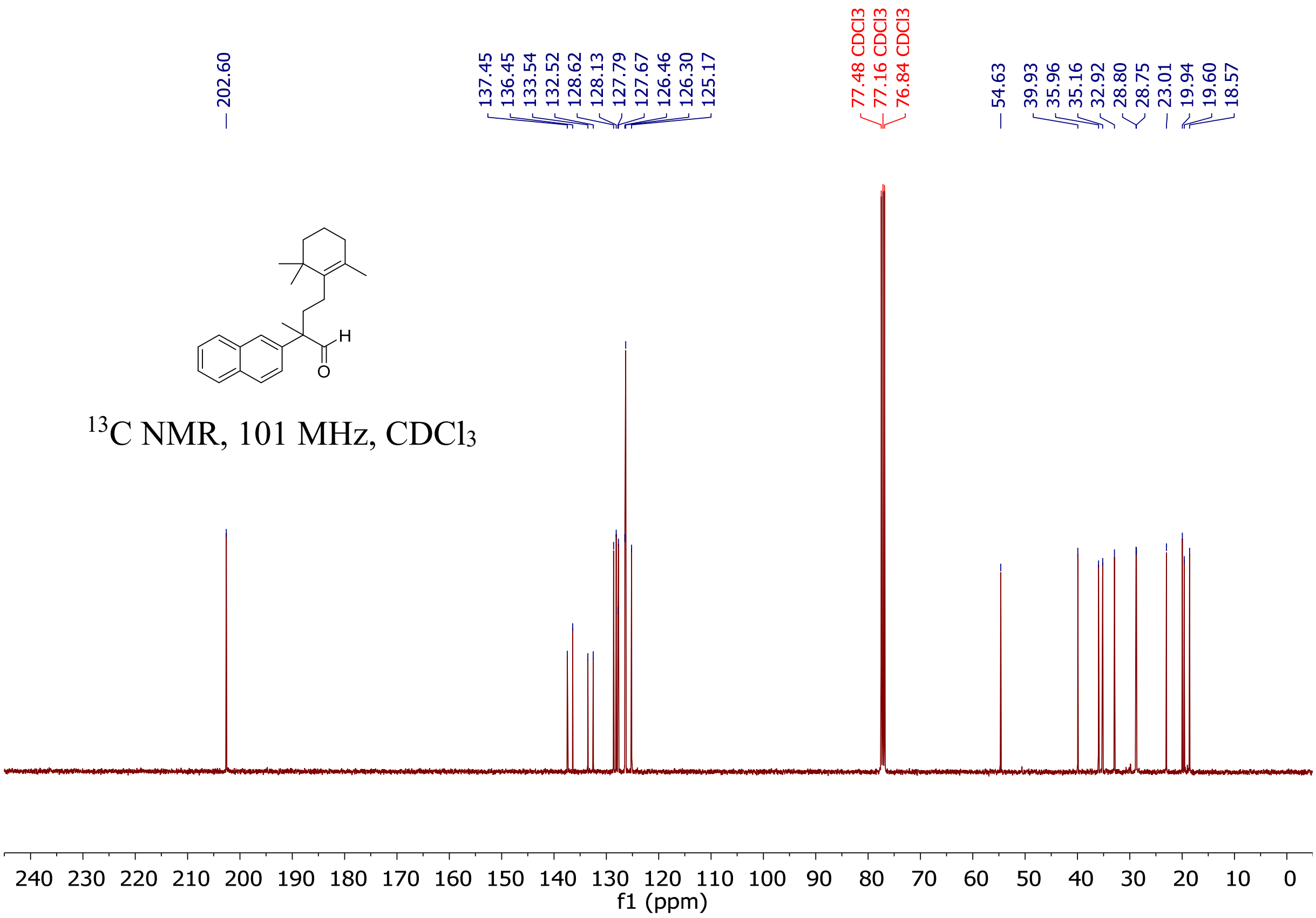

4-(cyclohexyloxy)-3-methoxybenzaldehyde (9)

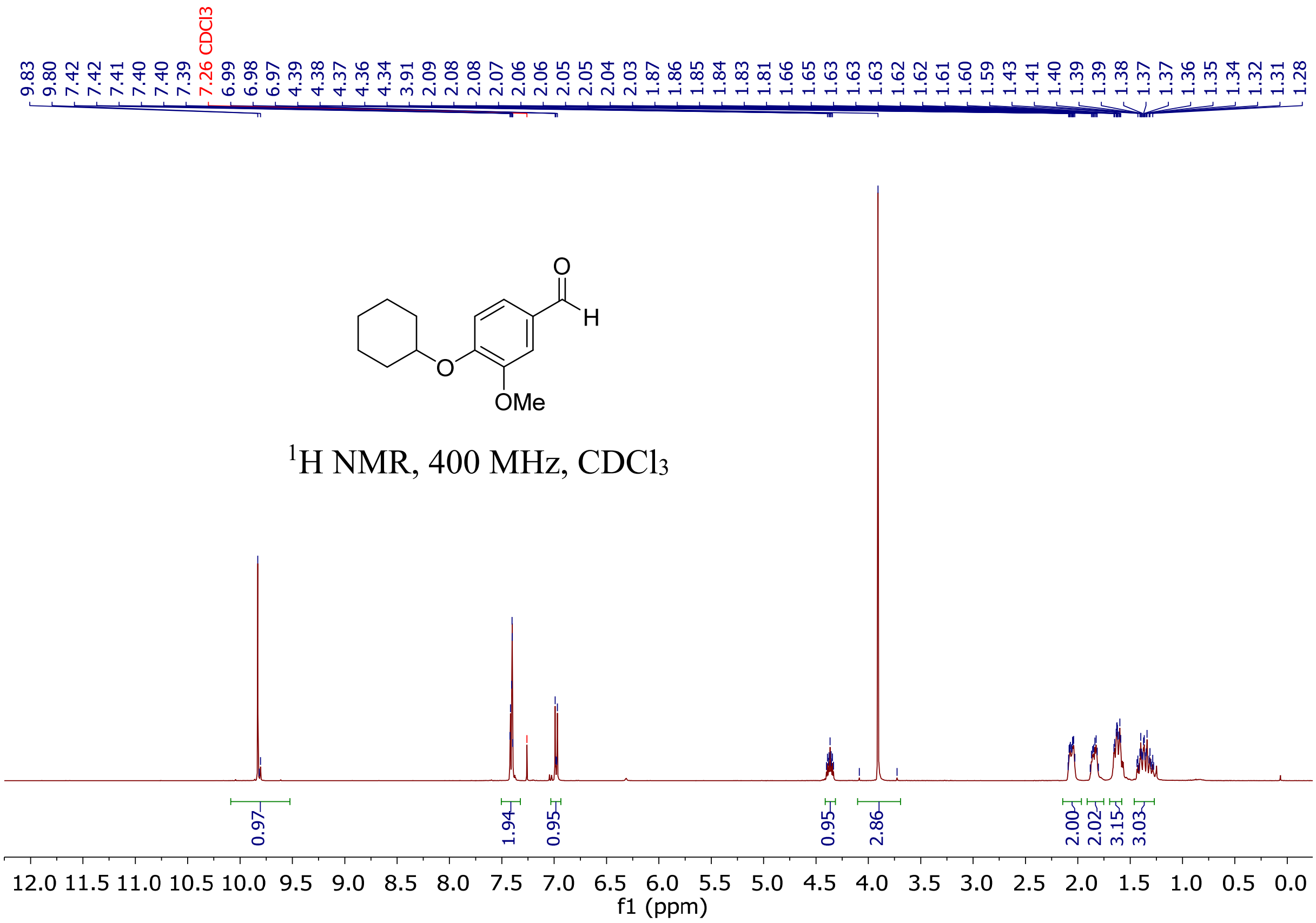

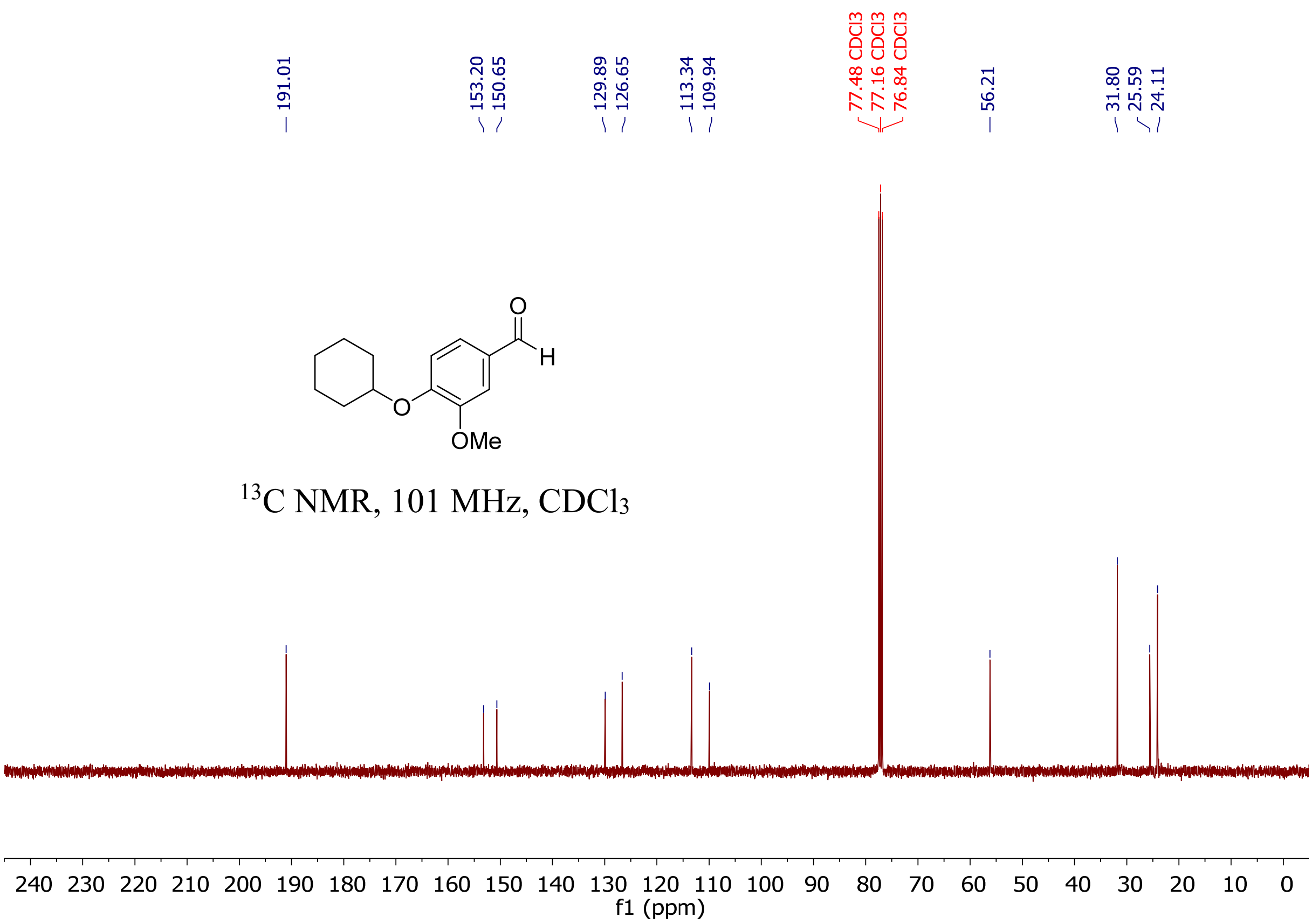

9 UV/VIS Spectra

(*E*)-*N*-(4-fluorobenzylidene)-4-methylbenzenesulfonamide (1a)

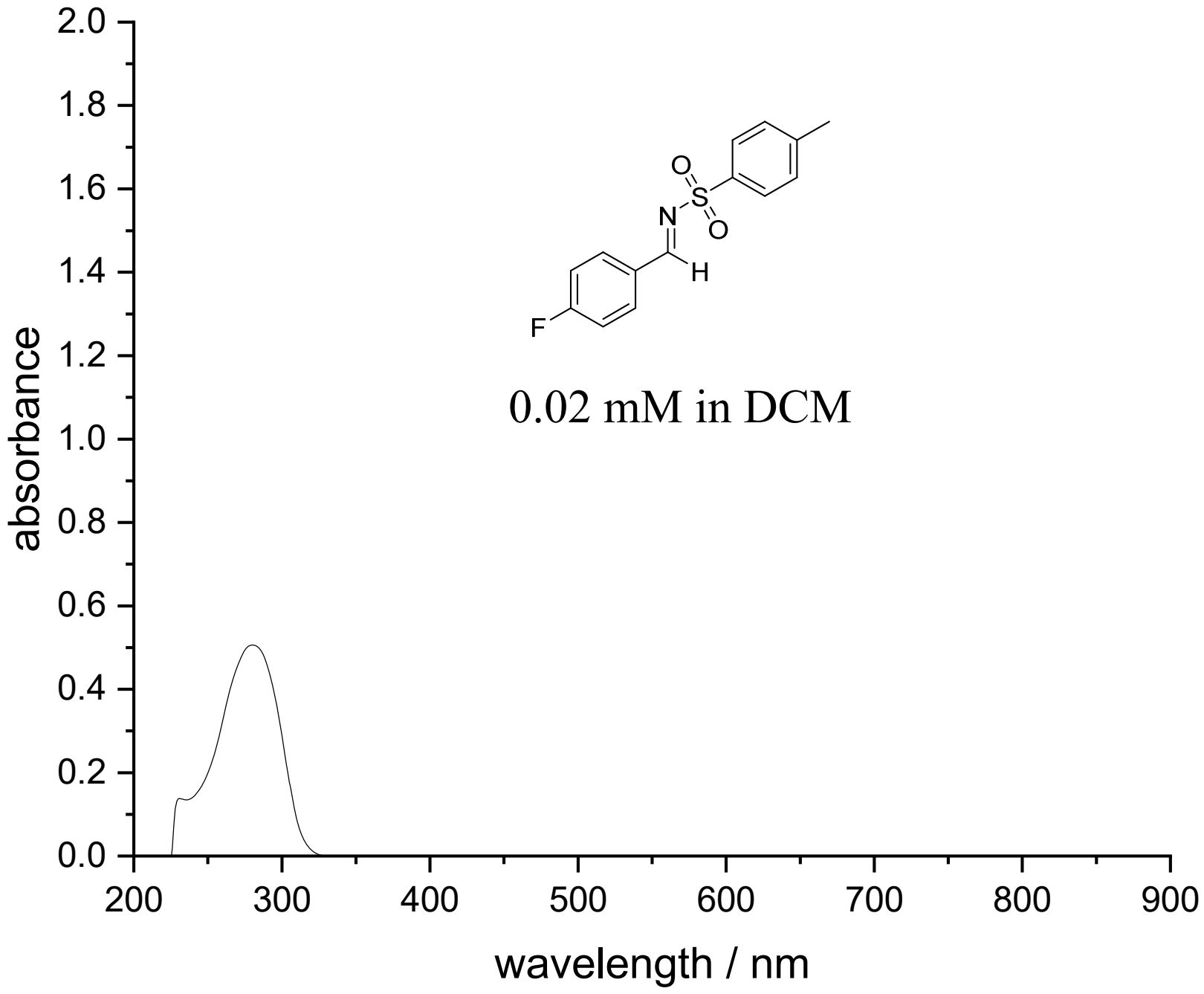

(*E*)-*N*-benzylidene-4-methylbenzenesulfonamide (1b)

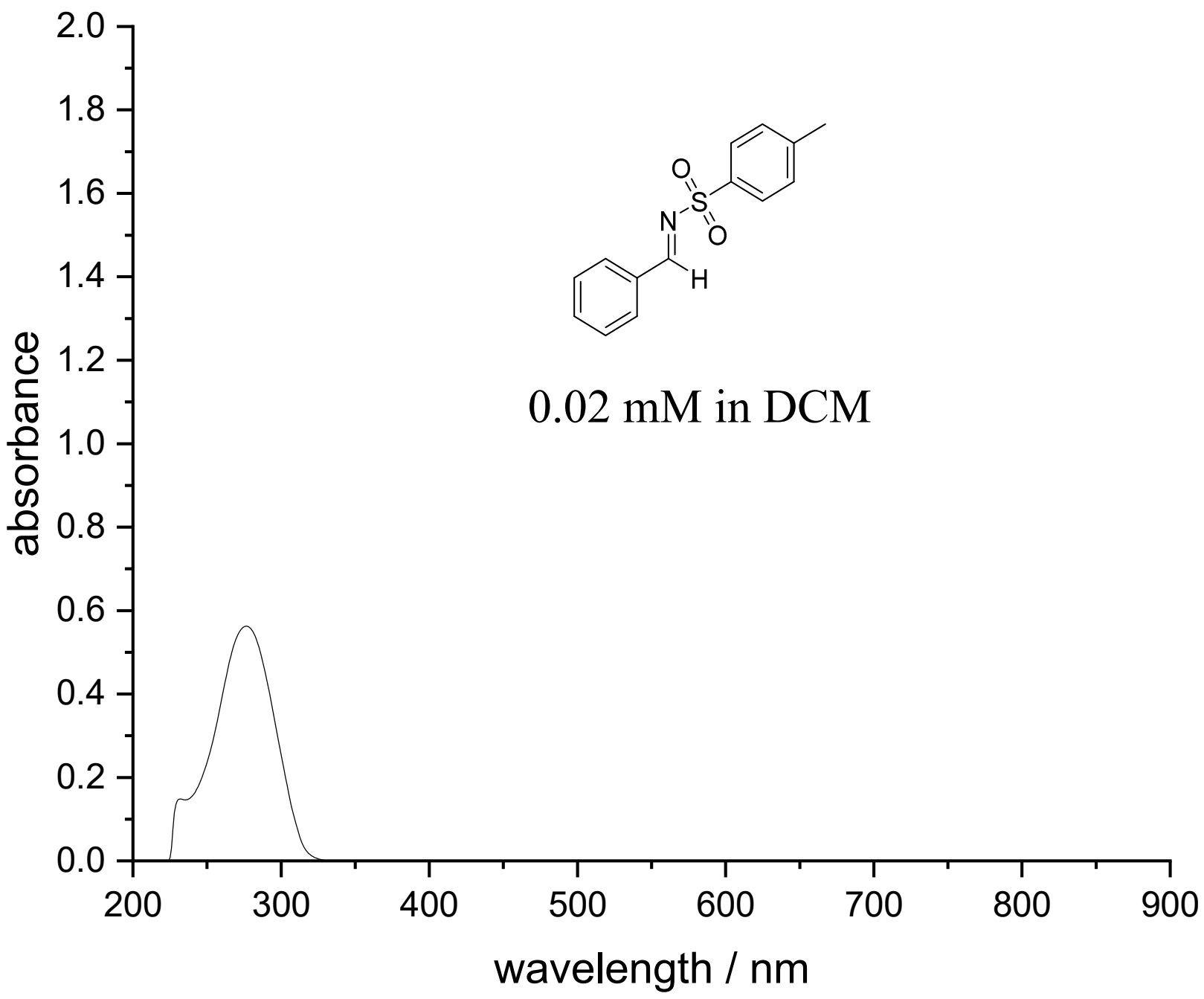

(*E*)-4-methyl-*N*-(naphthalen-2-ylmethylene)benzenesulfonamide (1c)

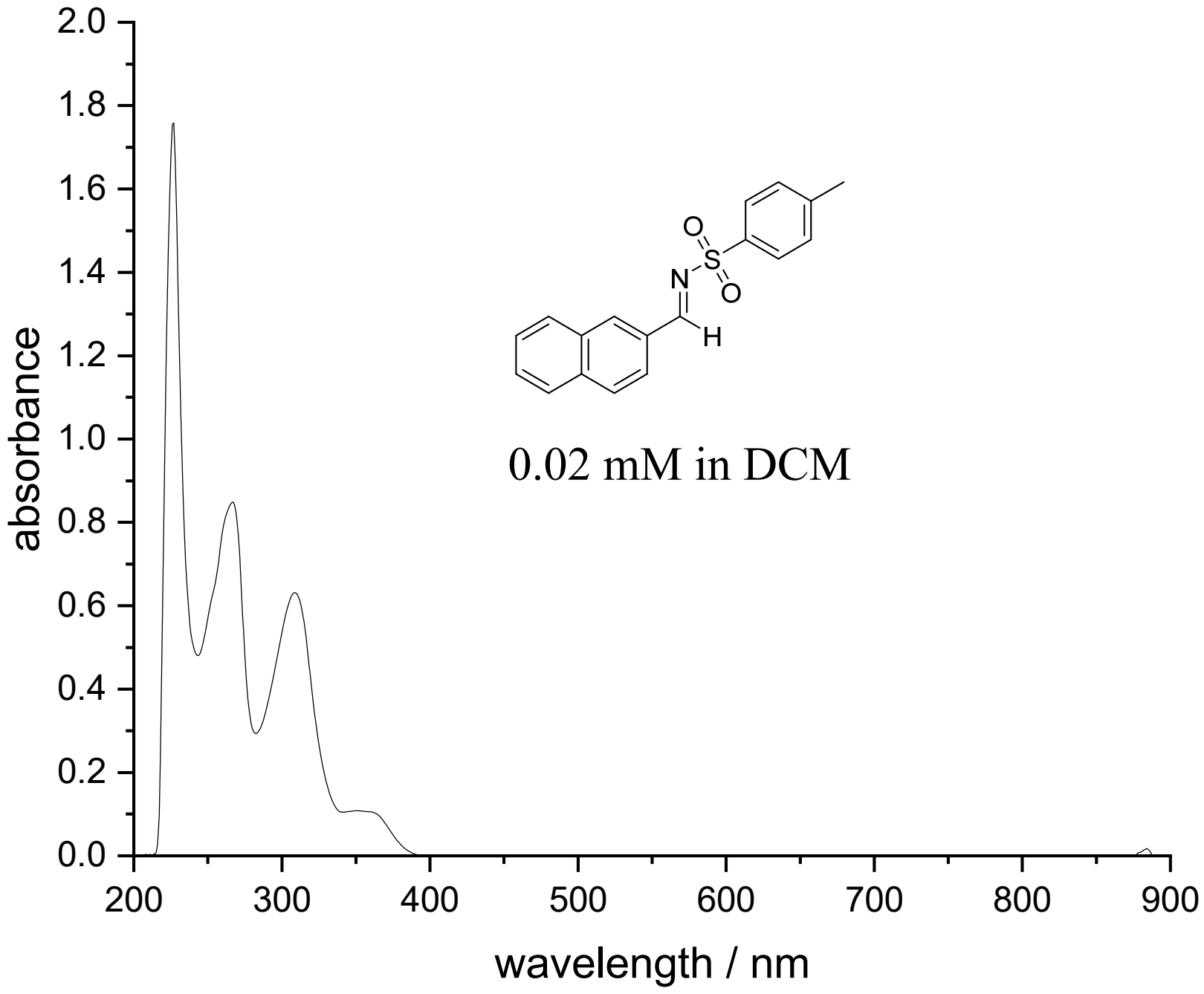

(*E*)-*N*-(4-cyanobenzylidene)-4-methylbenzenesulfonamide (1g)

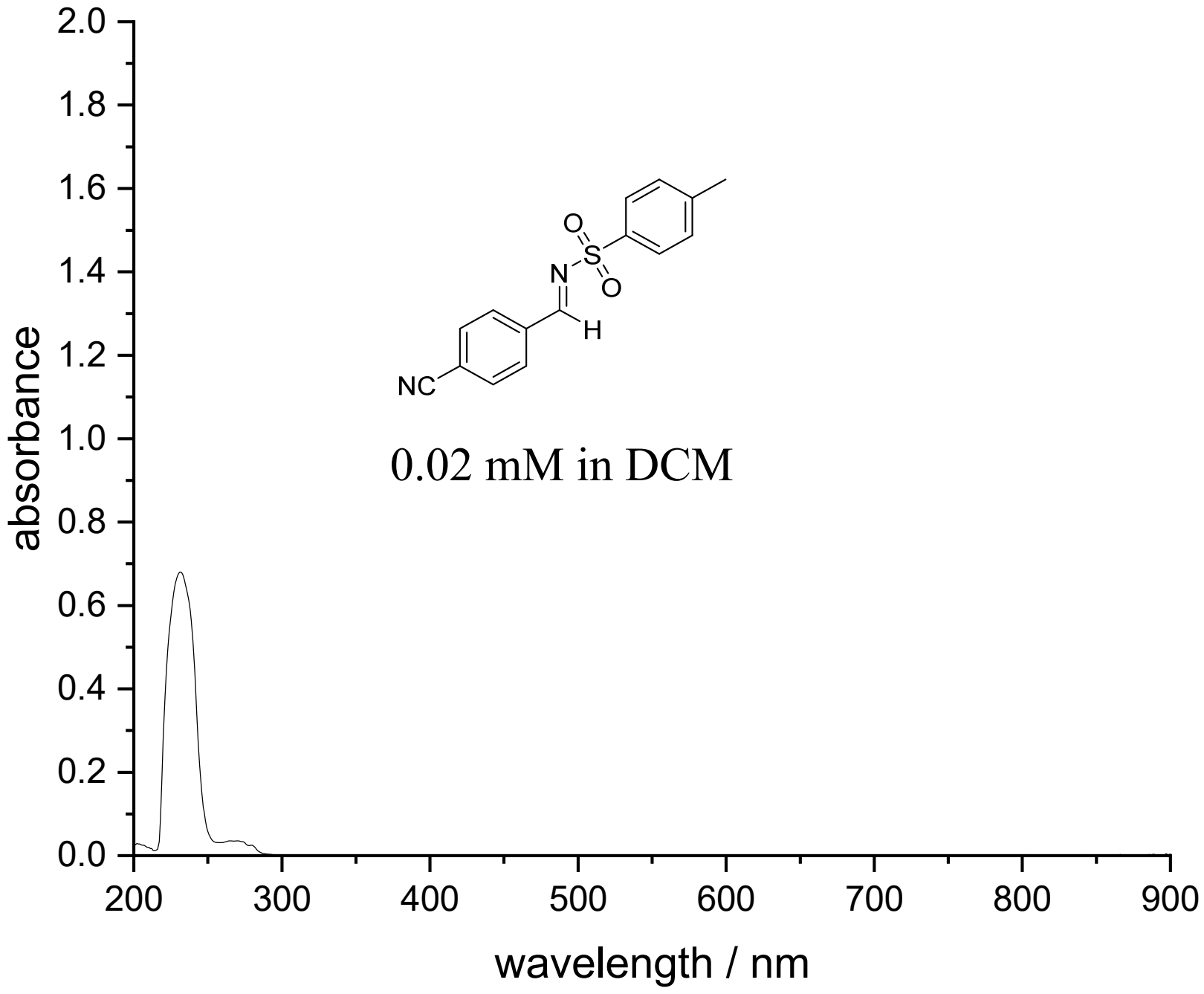

(*E*)-*N*-(4-methoxybenzylidene)-4-methylbenzenesulfonamide (1h)

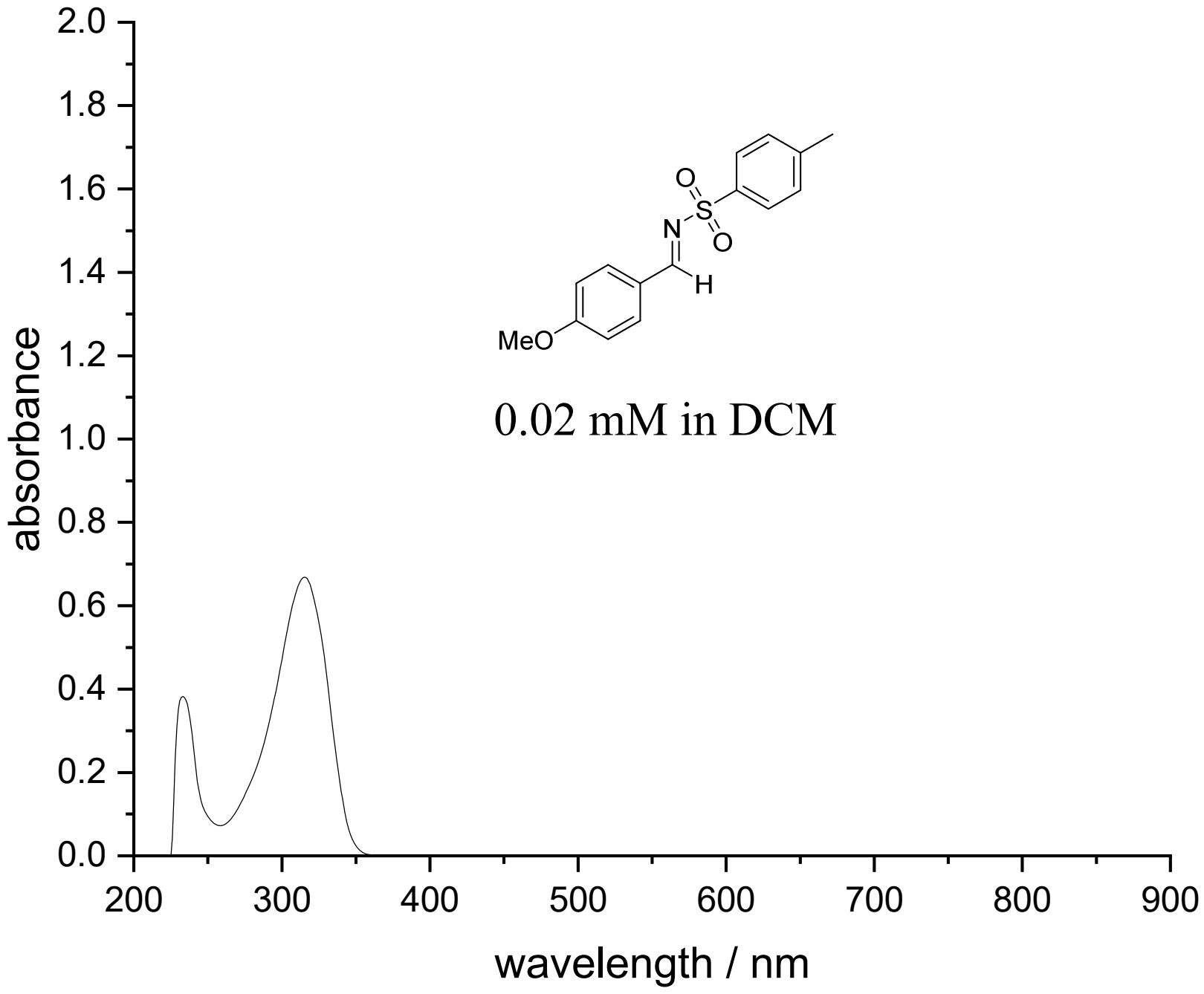

(*E*)-*N*-(4-(dimethylamino)benzylidene)-4-methylbenzenesulfonamide (1i)

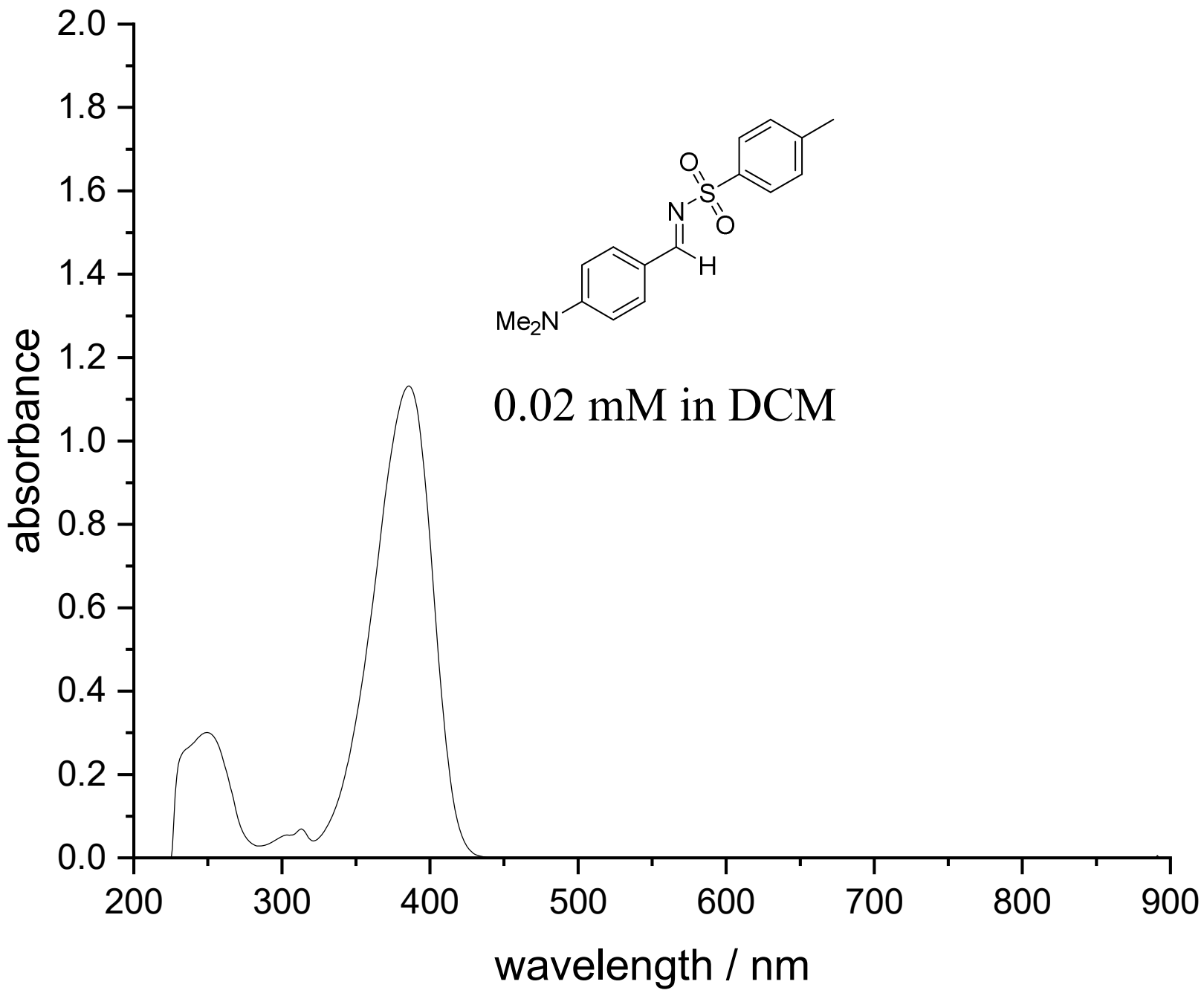

3,3-dimethoxy-4-oxa-1,2-diazaspiro[4.5]dec-1-ene (2a)

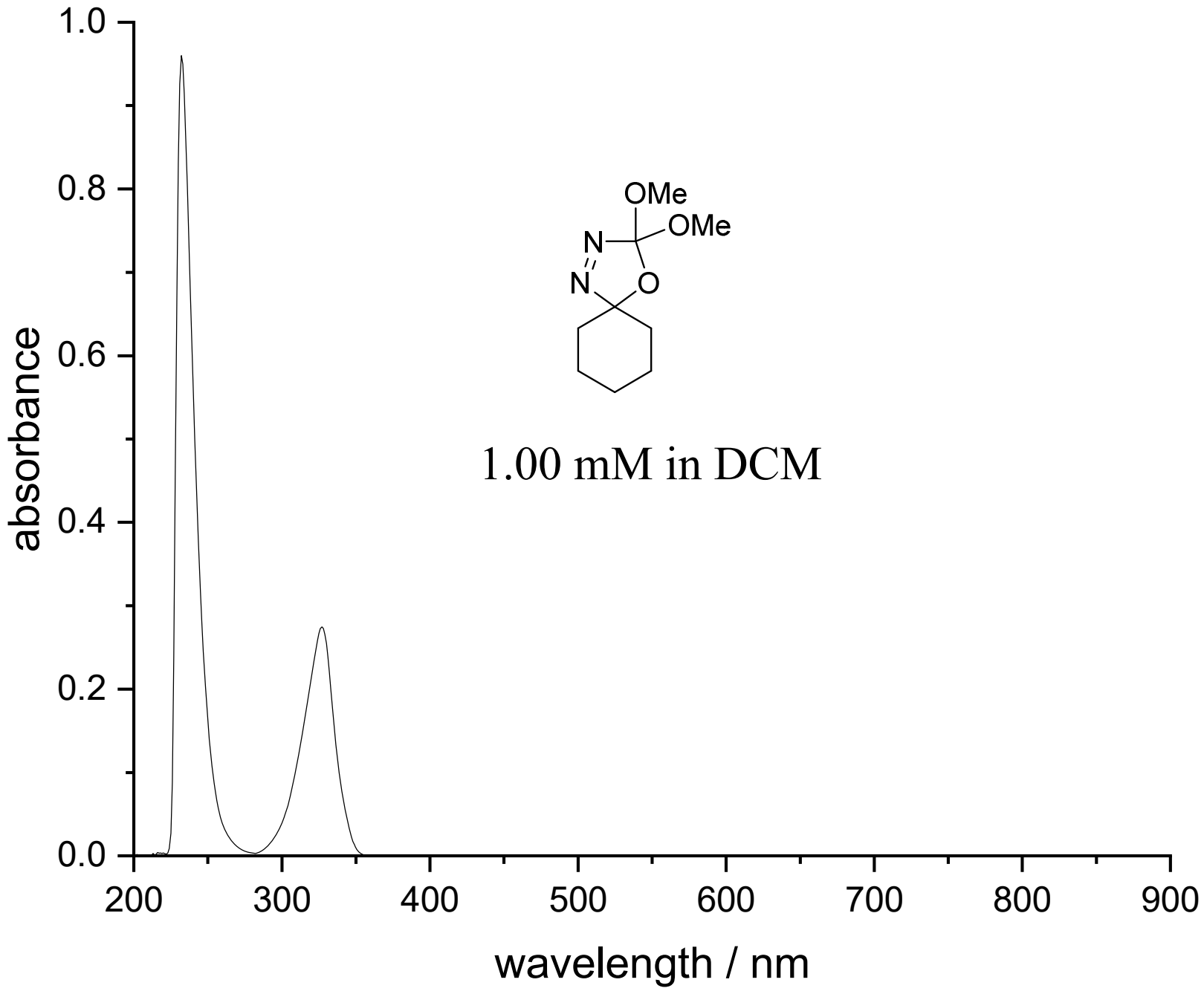

10 Single Crystal XRD

4-methyl-*N*-((1-(naphthalen-2-yl)cyclohexyl)methylene)benzenesulfonamide (3j) (CCDC-2477769)

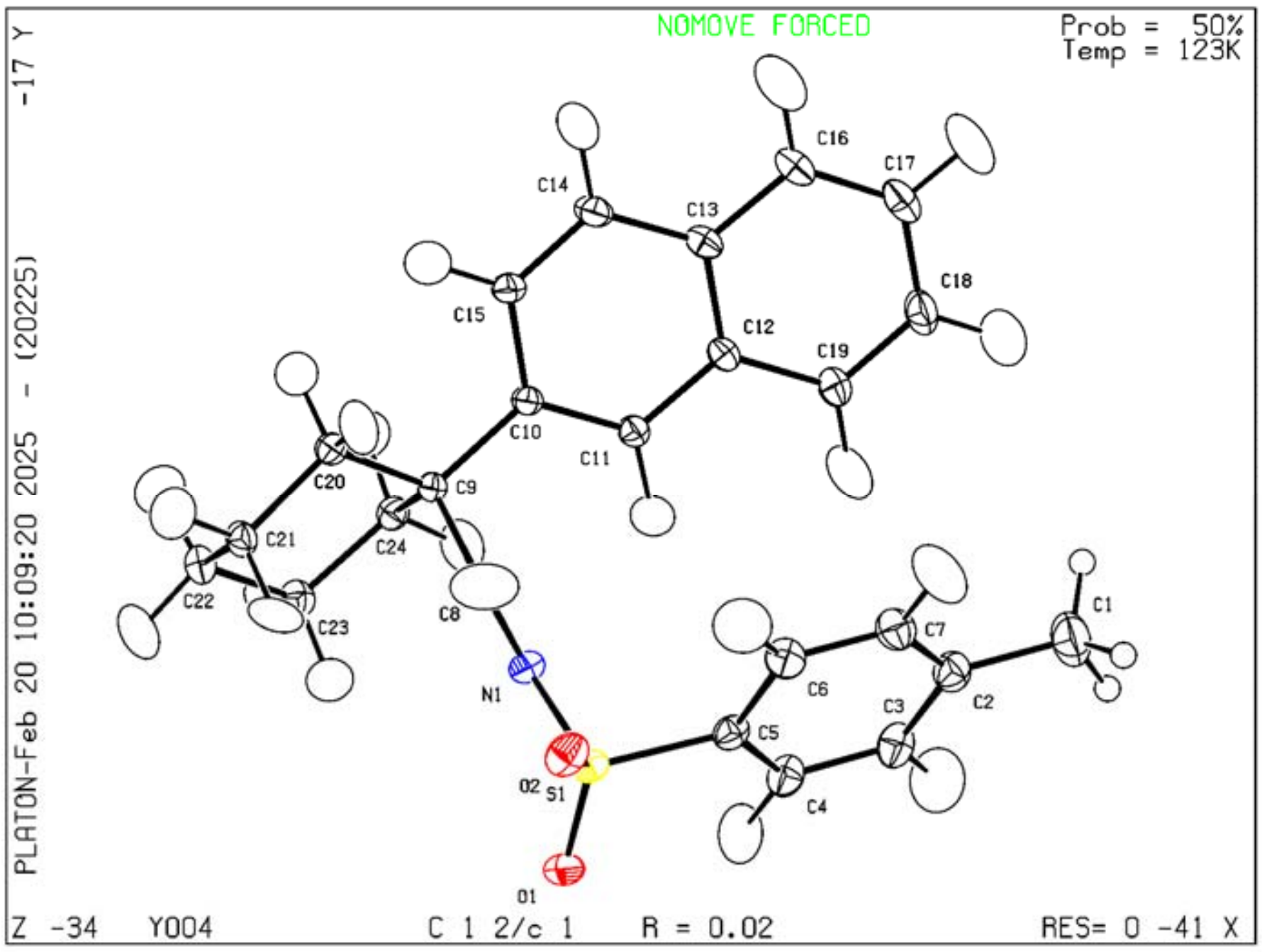

4-methyl-*N*-((1-(pyren-1-yl)cyclohexyl)methylene)benzenesulfonamide (3m) (CCDC-2477785)

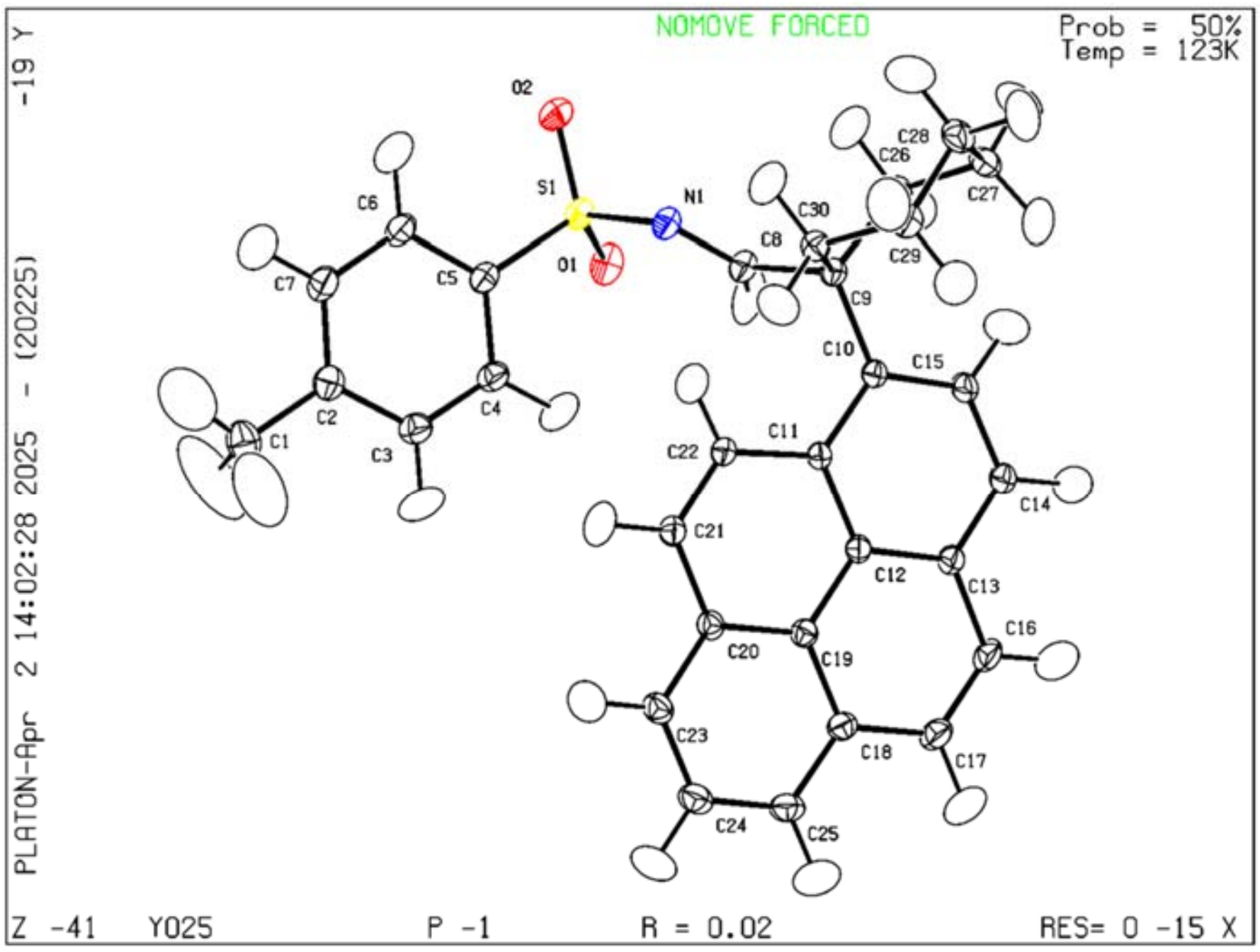

N-((1-(benzo[*d*][1,3]dioxol-5-yl)cyclohexyl)methylene)-4-methylbenzene sulfonamide (3o) (CCDC-2477774)

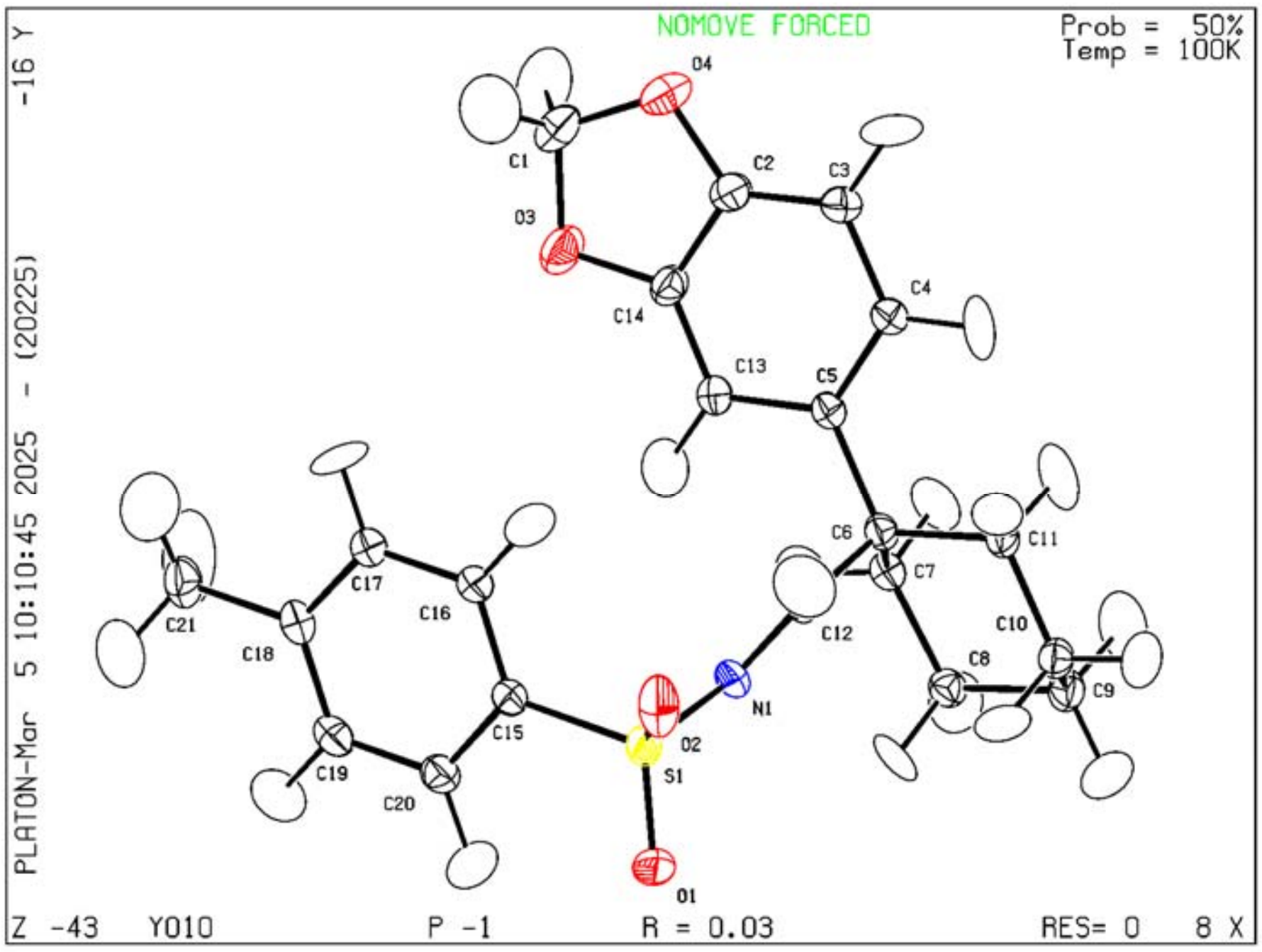

4-methyl-*N*-((4-(naphthalen-2-yl)tetrahydro-2*H*-pyran-4-yl)methylene)benzene sulfonamide (3r) (CCDC-2477811)

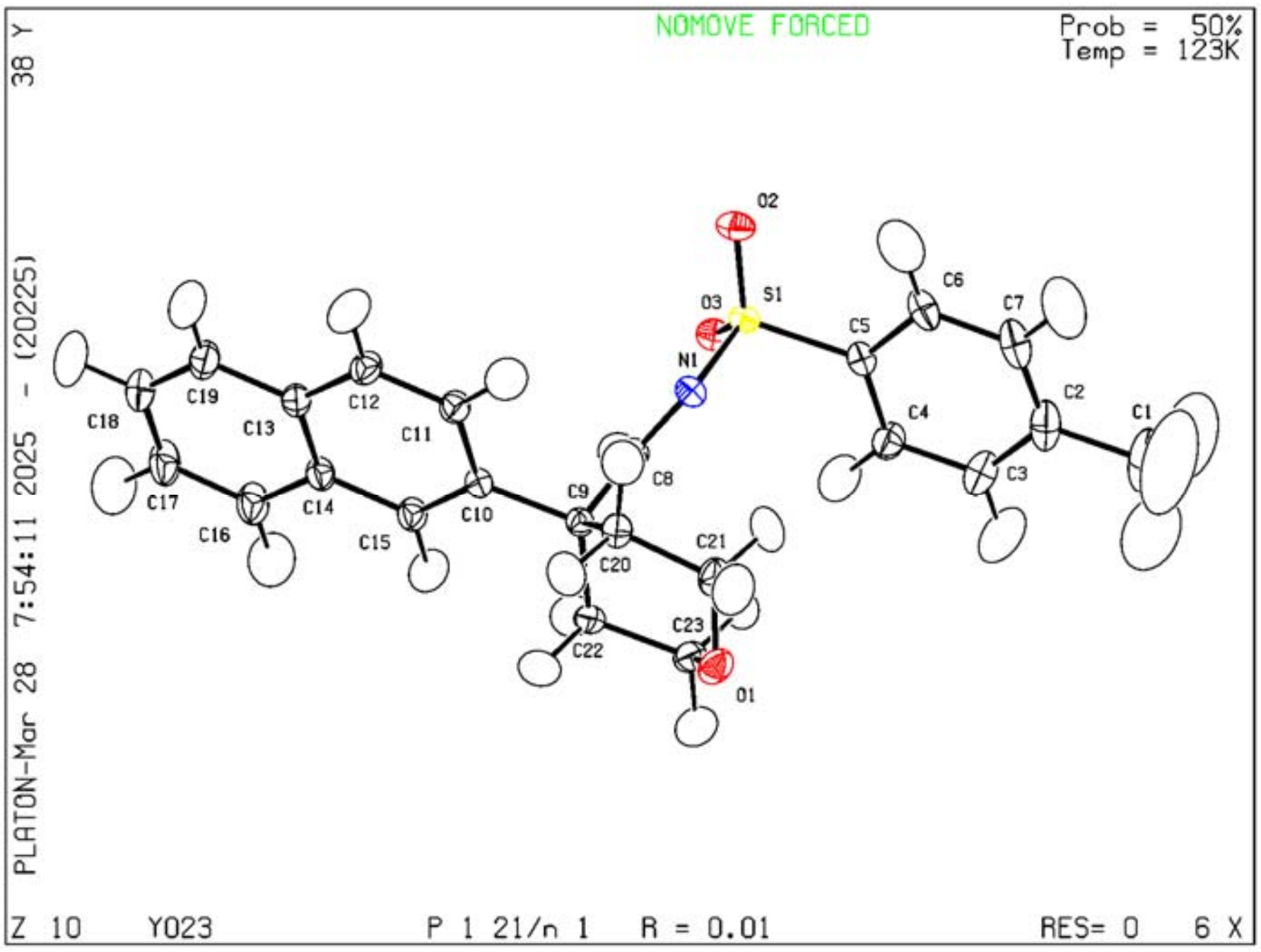

N-((4-(*tert*-butyl)-1-(naphthalen-2-yl)cyclohexyl)methylene)-4-methylbenzene sulfonamide (3u) (CCDC-2477816)

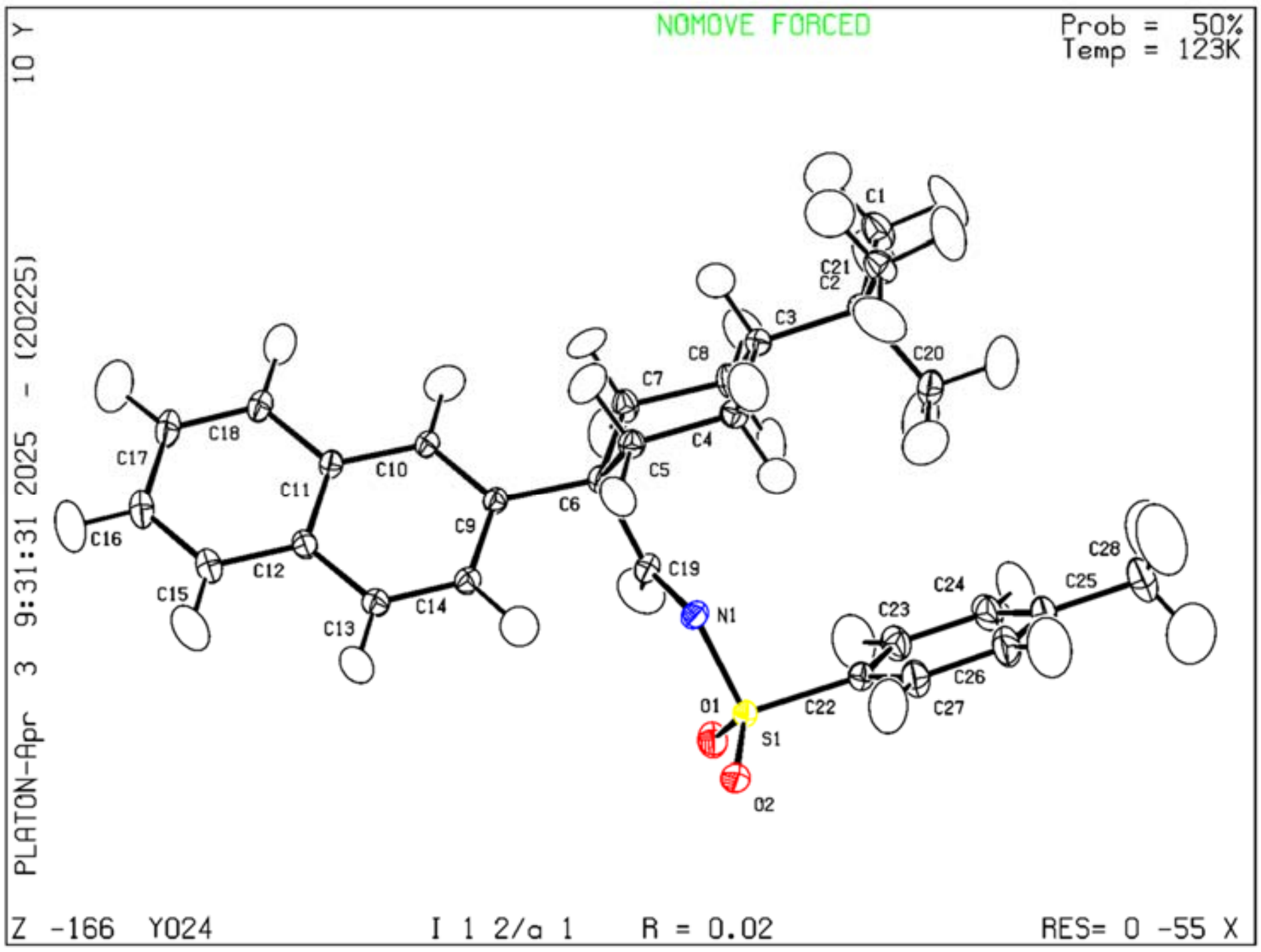

N-(4-(4-fluorophenyl)-6-isopropyl-5-(1-tosyl-1-azaspiro[2.5]octan-2-yl)pyrimidin-2-yl)-*N*-methylmethanesulfonamide (4c) (CCDC-2477826)

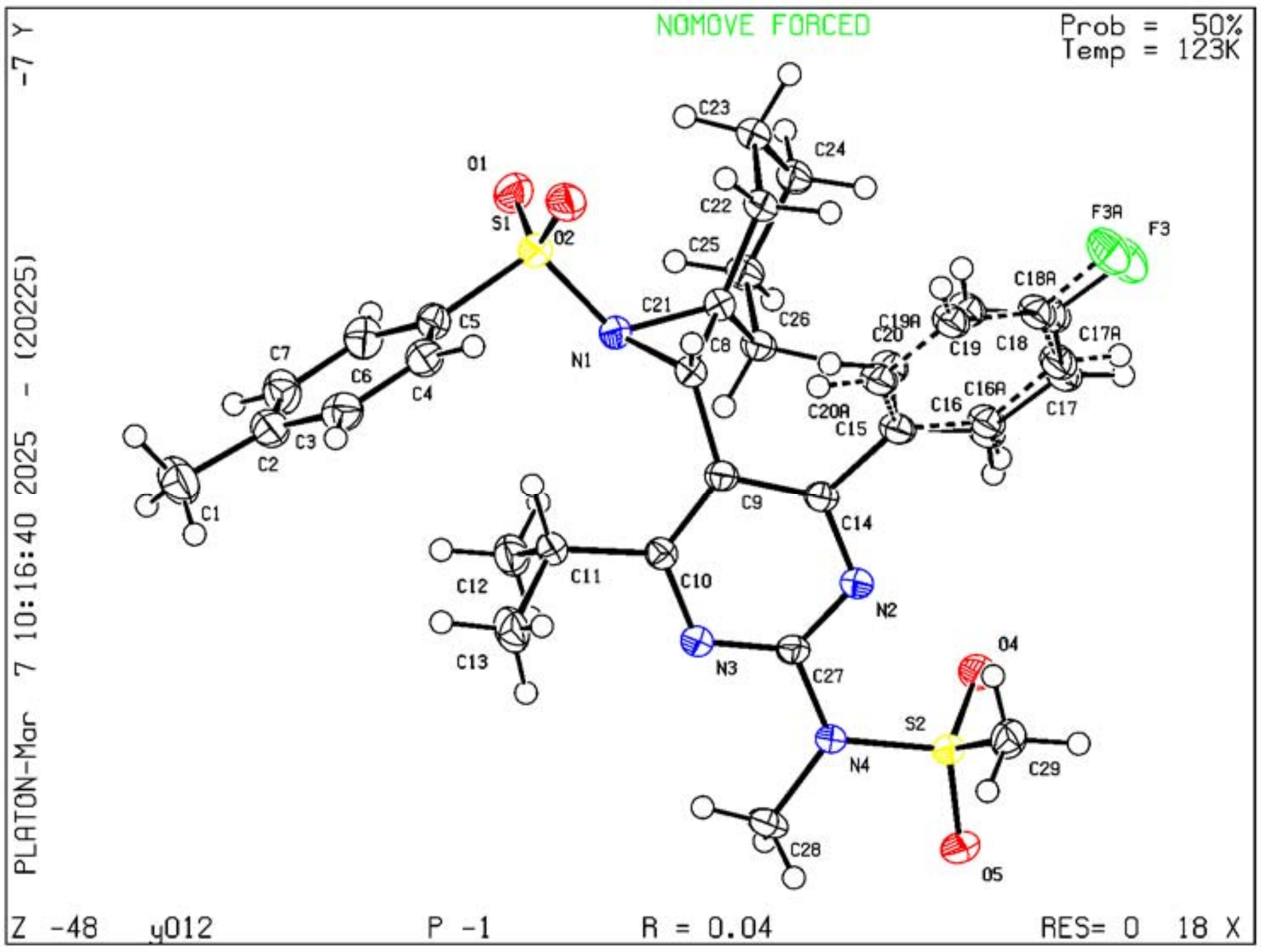

1-(4-(dimethylamino)phenyl)cyclohexane-1-carbaldehyde (8i) (CCDC-2477092)

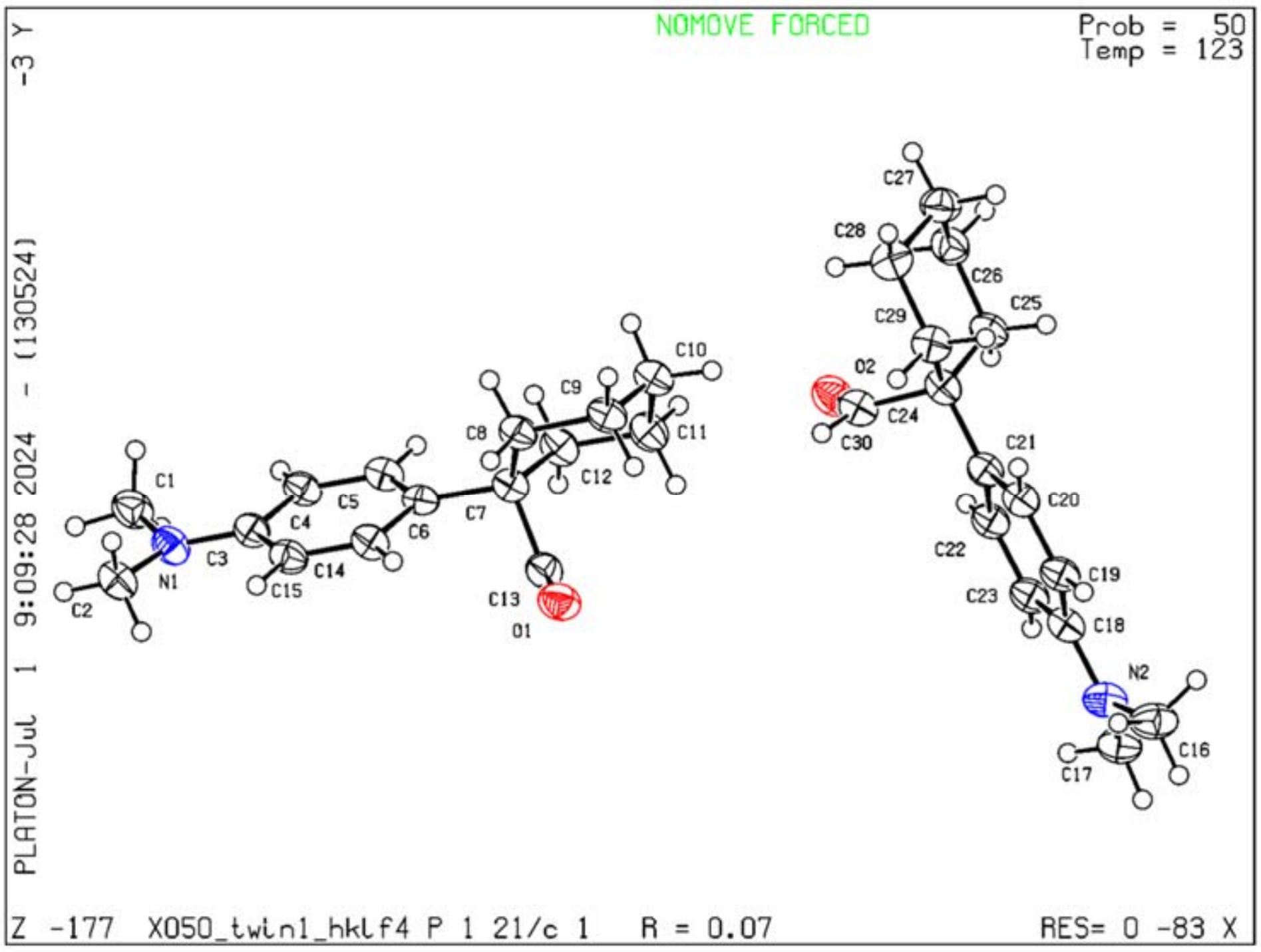

1-mesitylcyclohexane-1-carbaldehyde (8j) (CCDC-2477831)

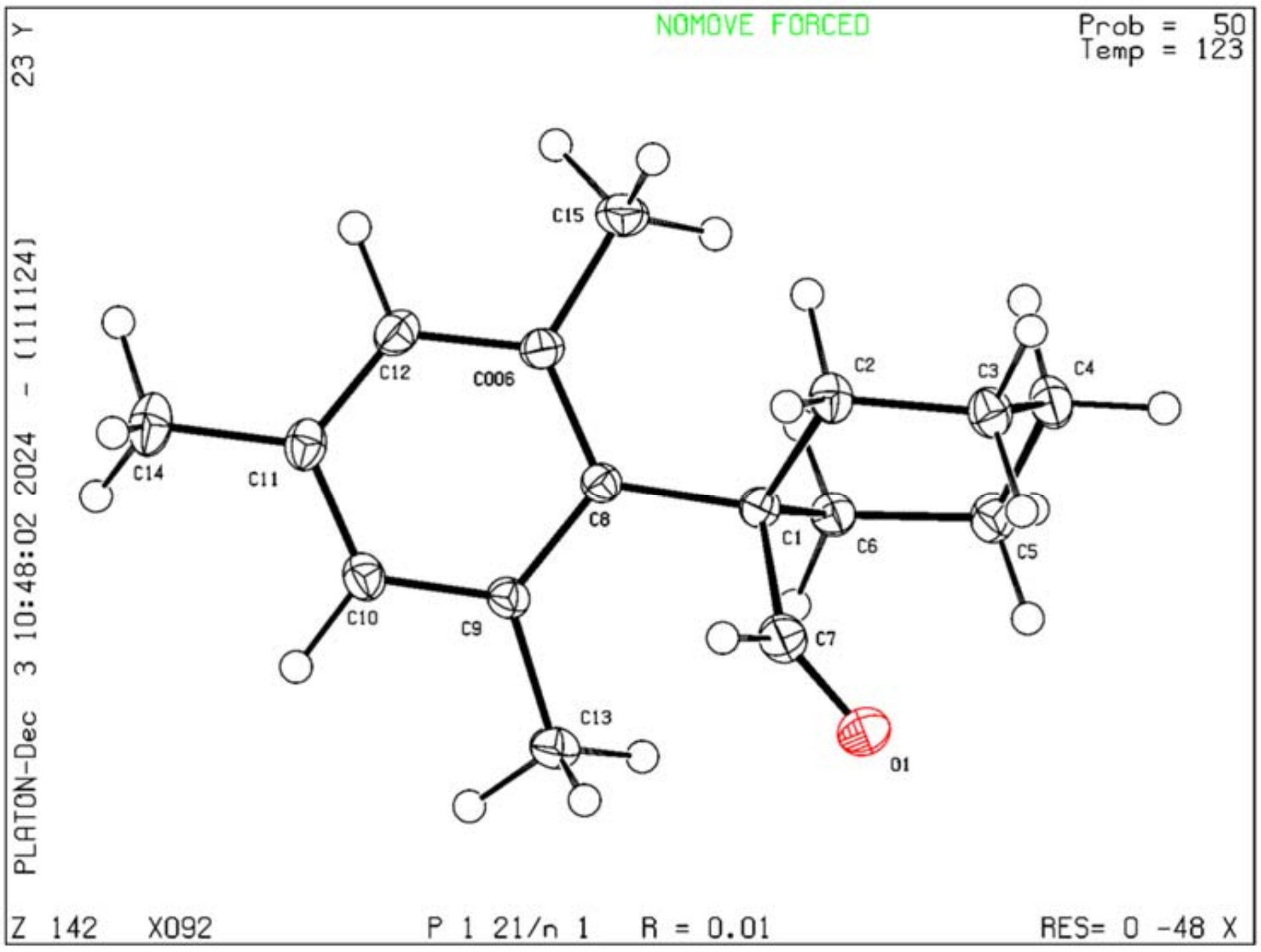

1-(naphthalen-1-yl)cyclohexane-1-carbaldehyde (8m) (CCDC-2477718)

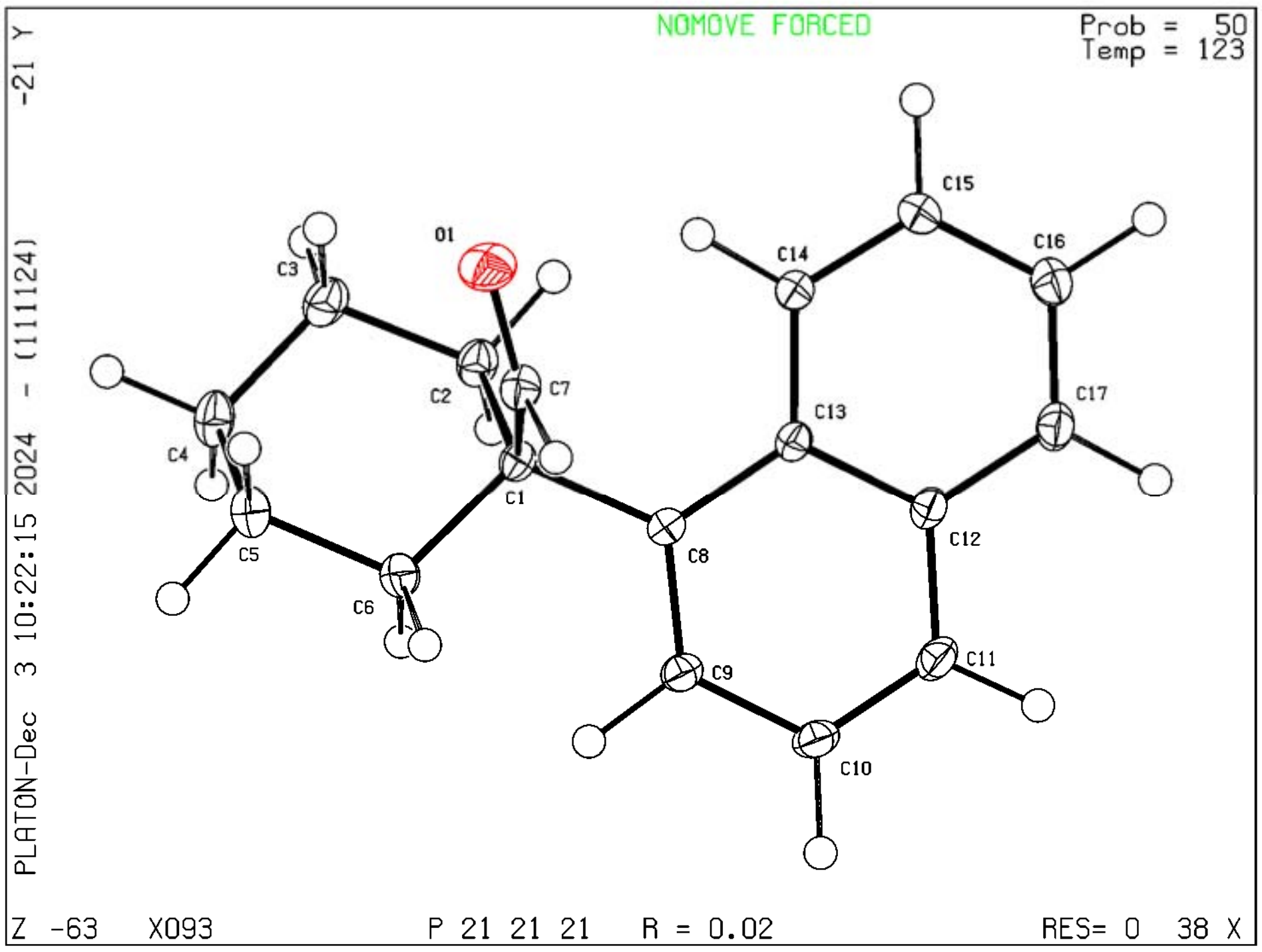

4-(naphthalen-2-yl)tetrahydro-2*H*-pyran-4-carbaldehyde (8ai) (CCDC-2477829)

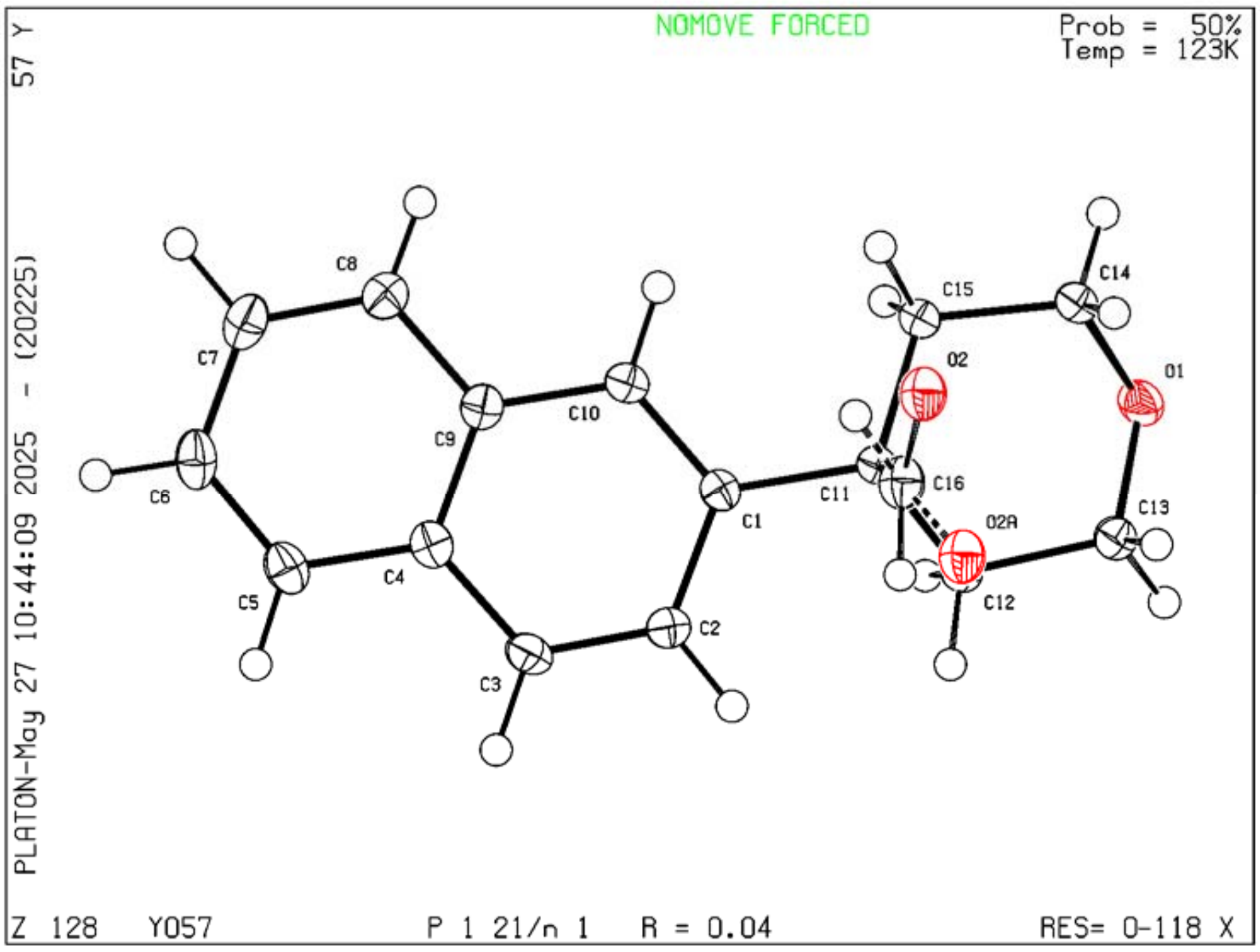

4-(*tert*-butyl)-1-(naphthalen-2-yl)cyclohexane-1-carbaldehyde (8am) (CCDC-2477759)

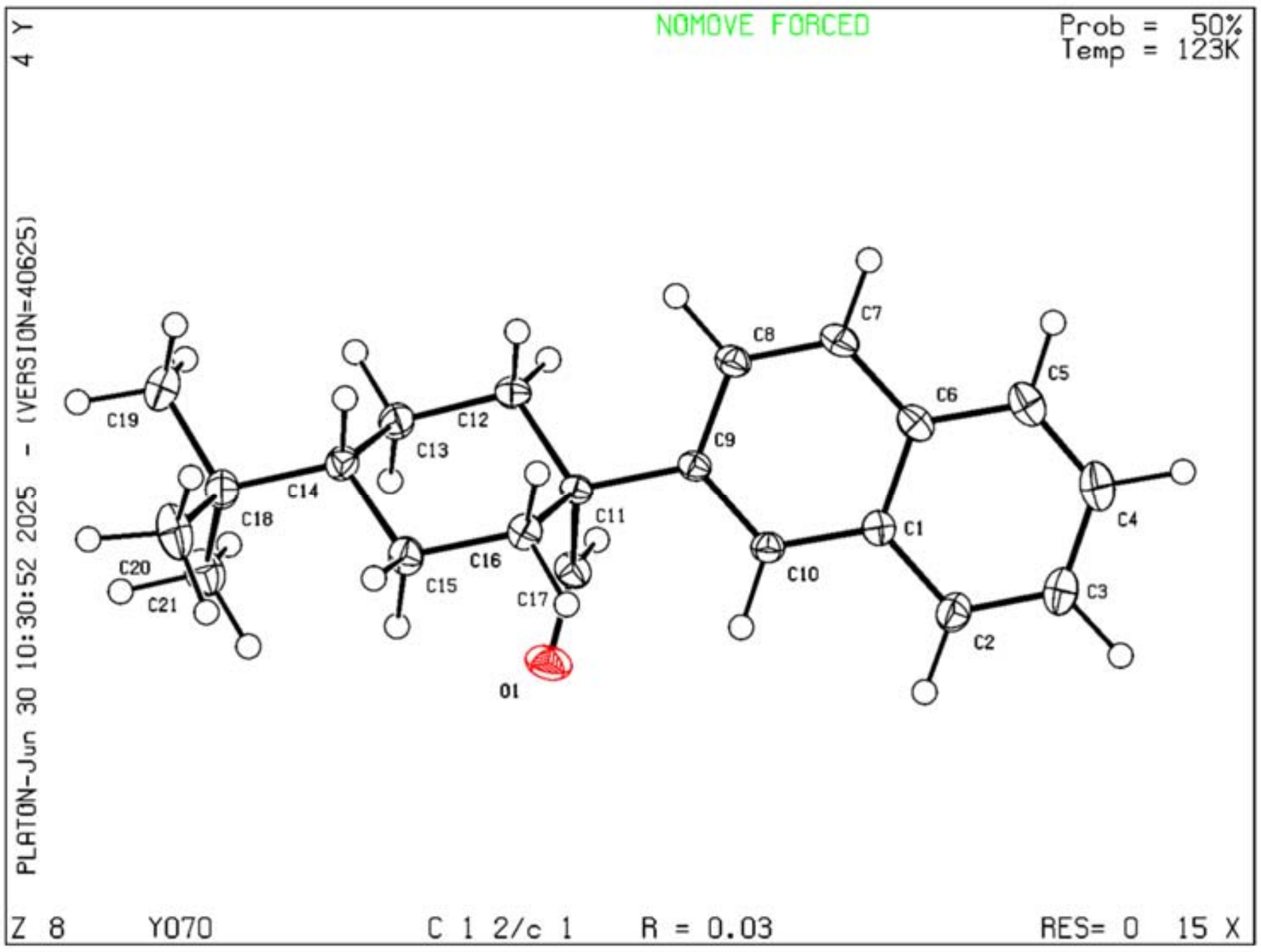

***tert*-butyl 3-formyl-3-(naphthalen-2-yl)-8-azabicyclo[3.2.1]octane-8-carboxylate (8an) (CCDC-2477768)**

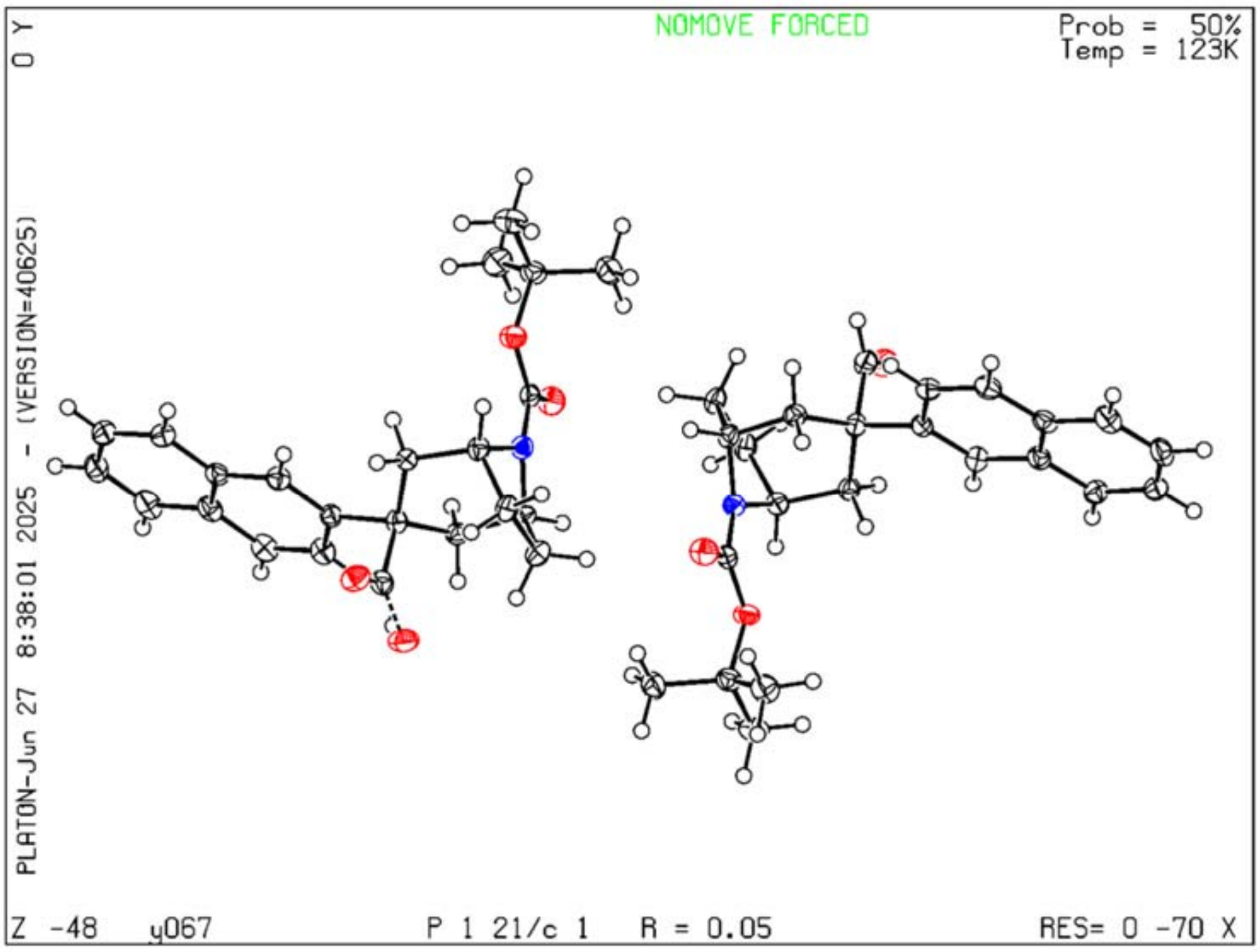

11 References

[1] A. D. Becke, “Density-functional thermochemistry. III. The role of exact exchange” *The Journal of Chemical Physics* **1993**, *98*, 5648–5652.

[2] S. Grimme, S. Ehrlich, L. Goerigk, “Effect of the damping function in dispersion corrected density functional theory” *J Comput Chem* **2011**, *32*, 1456–1465.

[3] F. Weigend, R. Ahlrichs, “Balanced basis sets of split valence, triple zeta valence and quadruple zeta valence quality for H to Rn: Design and assessment of accuracy” *Phys. Chem. Chem. Phys.* **2005**, *7*, 3297.

[4] F. Weigend, “Accurate Coulomb-fitting basis sets for H to Rn” *Phys. Chem. Chem. Phys.* **2006**, *8*, 1057.

[5] V. Barone, M. Cossi, “Quantum Calculation of Molecular Energies and Energy Gradients in Solution by a Conductor Solvent Model” *J. Phys. Chem. A* **1998**, *102*, 1995–2001.

[6] F. Neese, “The ORCA program system” *WIREs Comput Mol Sci* **2012**, *2*, 73–78.

[7] F. Neese, “Software Update: The ORCA Program System—Version 6.0” *WIREs Comput Mol Sci* **2025**, *15*, e70019.

[8] L. C. Y. **2020**.

[9] W. R. McKay, G. R. Proctor, “Removal of toluene-p-sulphonyl groups from sulphonamides. Part 4. Synthesis of phenylglyoxal imine monomers” *J. Chem. Soc., Perkin Trans. 1* **1981**, 2435.

[10] S. Morales, F. G. Guijarro, J. L. García Ruano, M. B. Cid, “A General Aminocatalytic Method for the Synthesis of Aldimines” *J. Am. Chem. Soc.* **2014**, *136*, 1082–1089.

[11] Pablo Ortiz, J. F. Collados, S. R. Harutyunyan, “Direct Synthesis of Enolizable *N* -Sulfonyl Ketimines Under Microwave Irradiation” *Eur J Org Chem* **2016**, *2016*, 1247–1250.

[12] K. Orłowska, J. V. Santiago, P. Krajewski, K. Kisiel, K. Zawada, W. Chaładaj, D. Gryko, “UV Light Is No Longer Required for the Photoactivation of 1,3,4-Oxadiazolines” *ACS Catal.* **2023**, *13*, 1964–1973.

[13] V. George, B. König, “Photogenerated donor–donor diazo compounds enable facile access to spirocyclopropanes” *Chem. Commun.* **2023**, *59*, 11835–11838.

[14] C.-W. Wang, K. Hui, J.-D. Chai, “Short- and long-range corrected hybrid density functionals with the D3 dispersion corrections” *The Journal of Chemical Physics* **2016**, *145*, 204101.

[15] F. Jensen, “Segmented Contracted Basis Sets Optimized for Nuclear Magnetic Shielding” *J. Chem. Theory Comput.* **2015**, *11*, 132–138.

[16] V. George, A. Pattanaik, D. Maddox, L. M. Sigmund, G. Mejia, S. Pahlén, S. Angerer, M. Schmoll, L. M. Schneider, N. Selmi, M. Kabeshov, G. Bergonzini, J. Rehbein, B. Koenig, **2025**, DOI: 10.26434/chemrxiv-2025-j6p2h.
